# Structure–Activity Relationship Study Reveals ML240 and ML241 as Potent and Selective Inhibitors of p97 ATPase

**DOI:** 10.1002/cmdc.201200520

**Published:** 2013-01-11

**Authors:** Tsui-Fen Chou, Kelin Li, Kevin J Frankowski, Frank J Schoenen, Raymond J Deshaies

**Affiliations:** a Division of Biology and Howard Hughes Medical Institute, California Institute of Technology1200 East California Boulevard, Pasadena, CA 91125 (USA); b University of Kansas Specialized Chemistry Center2034 Becker Drive, Structural Biology Center, West Campus, Lawrence, KS 66047 (USA); c Division of Medical Genetics, Department of Pediatrics, Harbor-UCLA Medical Center and Los Angeles Biomedical Research Institute1124 West Carson Street, Torrance, CA 90502 (USA)

**Keywords:** AAA ATPase, autophagy, cancer, structure–activity relationships, ubiquitin proteasome

## Abstract

To discover more potent p97 inhibitors, we carried out a structure–activity relationship study of the quinazoline scaffold previously identified from our HTS campaigns. Two improved inhibitors, ML240 and ML241, inhibit p97 ATPase with IC_50_ values of 100 nm. Both compounds inhibited degradation of a p97-dependent but not a p97-independent proteasome substrate in a dual-reporter cell line. They also impaired the endoplasmic-reticulum-associated degradation (ERAD) pathway. Unexpectedly, ML240 potently stimulated accumulation of LC3-II within minutes, inhibited cancer cell growth, and rapidly mobilized the executioner caspases 3 and 7, whereas ML241 did not. The behavior of ML240 suggests that disruption of the protein homeostasis function of p97 leads to more rapid activation of apoptosis than is observed with a proteasome inhibitor. Further characterization revealed that ML240 has broad antiproliferative activity toward the NCI-60 panel of cancer cell lines, but slightly lower activity toward normal cells. ML240 also synergizes with the proteasome inhibitor MG132 to kill multiple colon cancer cell lines. Meanwhile, both probes have low off-target activity toward a panel of protein kinases and central nervous system targets. Our results nominate ML240 as a promising starting point for the development of a novel agent for the chemotherapy of cancer, and provide a rationale for developing pathway-specific p97 inhibitors.

## Introduction

The hexameric p97 protein belongs to the type II AAA (***A***TPase ***a***ssociated with diverse cellular ***a***ctivities) ATPase protein family and is conserved across all eukaryotes and is essential for life.^1^,^2^ p97 is known as VCP (valosin-containing protein) in mammals and Cdc48p in yeast. p97 plays critical roles in a broad array of cellular processes including homotypic fusion of endoplasmic reticulum and Golgi membranes,^3^ degradation of misfolded membrane and secretory proteins,^4^ Golgi membrane reassembly,^5^ membrane transport,^6^ regulation of myofibril assembly,^7^ cell division,^8^ regulation of protein aggregates,^9^ and autophagosome maturation.^10^, ^11^ The broad range of cellular functions for p97 is thought to derive from its ability to unfold proteins or disassemble protein complexes using the energy derived from ATP hydrolysis, but the detailed mechanism of how p97 works remains largely elusive.

The mechanochemical activity of p97 is linked to substrate proteins by a large number of interacting proteins (p97 cofactors) including Npl4 (nuclear protein localization homologue 4), Ufd1 (ubiquitin fusion degradation 1) heterodimer,^12^ and an array of 13 UBX (ubiquitin regulatory X) domain cofactors.^13^ The physiological functions and mechanisms of action of these different p97–cofactor complexes remain largely unknown. Our research group reported a proteomic analysis of UBX cofactors and revealed their interactions with a large number of E3 ligases.^14^ Whereas all UBX proteins interact with p97, only those containing a ubiquitin associated (UBA) domain associate with high-molecular-weight ubiquitin conjugates.^14^ Thus, some p97–UBX complexes may have functions that do not involve polyubiquitination of their substrates. In support of this observation, Ubxd1, a non-UBA containing UBX protein, associates with monoubiquitinated caveolin-1 and regulates its endolysosomal sorting.^15^

p97 exhibits a tripartite structure, with an N-terminal domain that recruits cofactor/substrate specificity factors followed by two AAA ATPase domains, D1 and D2.^16^, ^17^ p97 protomers assemble to form a homohexamer that is thought to provide a platform for transduction of chemical energy into mechanical force, which is then applied to substrate proteins. The D1 domain mediates hexamerization^18^ and has very low basal ATPase activity.^19^ The majority of the ATPase activity detected in vitro appears to arise from the D2 domain.^20^ Interestingly, D1 ATPase mutations affect D2 ATPase activity, and it has been suggested that a functional D1 domain and positive cooperativity between the D1 and D2 domains is essential for cell growth.^21^

We recently reported two high-throughput screening (HTS) campaigns that yielded a specific small-molecule inhibitor of p97 ATPase activity, *N*^2^,*N*^4^-dibenzylquinazoline-2,4-diamine (DBeQ).^22^ DBeQ affects multiple p97-dependent processes including ubiquitin fusion degradation (UFD), endoplasmic-reticulum-associated degradation (ERAD), and autophagy. DBeQ also potently inhibits cancer cell growth. Interestingly, DBeQ mobilizes the executioner caspases 3 and 7 and induces apoptosis more rapidly than the proteasome inhibitor MG132,^22^ thus highlighting p97 as a potentially suitable target for cancer chemotherapy.

## Results and Discussion

### SAR for p97 inhibition by 1 and DBeQ

**Figure 1:**
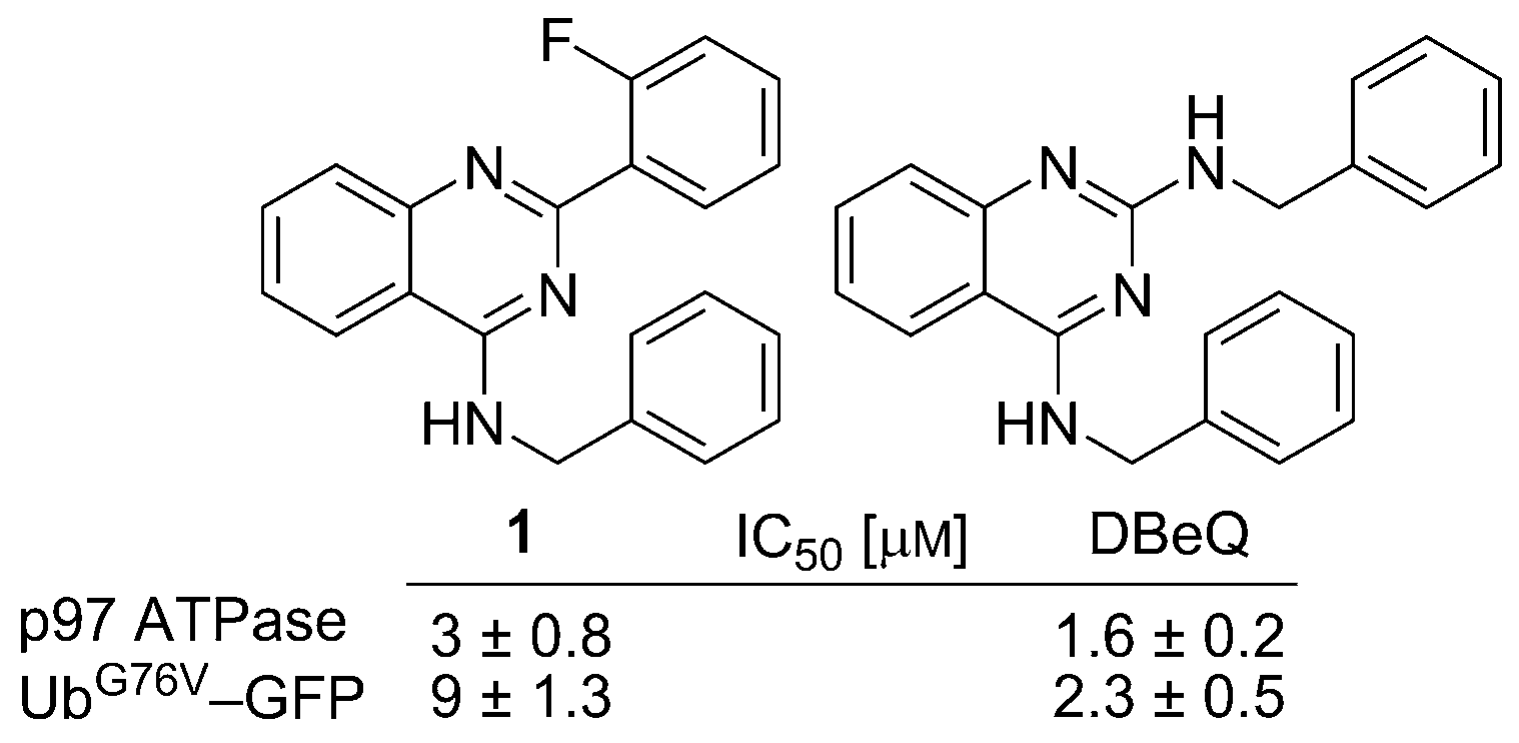
Structures and p97 inhibitory activities for the HTS hit compounds 1 and DBeQ. IC_50_ values for inhibition of p97 ATPase activity and degradation of p97-dependent reporter (Ub^G76V^–GFP) are shown.

**Figure 2:**
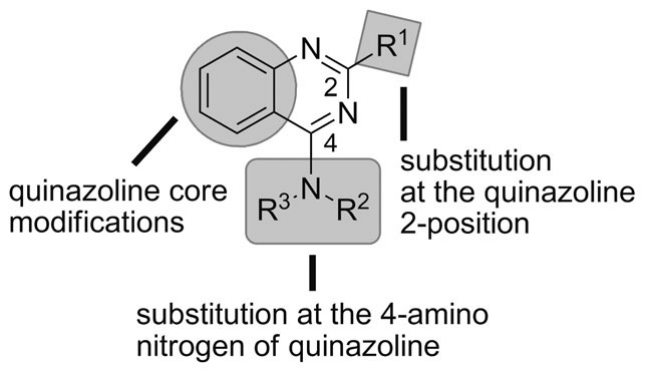
Summary of the SAR leading to the discovery of ML240 and ML241: 200 analogues were tested, mainly exploring changes to R^1^ and R^2^.

In addition to DBeQ, the HTS screen identified *N*-benzyl-2-(2-fluorophenyl)quinazolin-4-amine **1** (Figure 1) as a promising hit warranting further investigation.^22^ With the aim to discover more potent p97 inhibitors, we carried out a structure–activity relationship (SAR) study of these two quinazoline hits identified during the HTS phase (Figure 2).^22^ In total, we examined 200 analogues with the majority focused on changes at R^1^ and R^2^. R^1^ replacement afforded the most dramatic potency gains, and the benzyl group from the HTS hits remained the preferred R^2^ substitution. Any substitution for R^3^ other than hydrogen was not well tolerated, and further analogues were not explored. Modification to the core quinazoline scaffold typically required de novo synthesis of the quinazoline, and although not exhaustively investigated, alternate core scaffolds and substituted quinazolines provided a significant boost in potency when applied to an already potent R^1^ and R^2^ combination.

We first purchased analogues of **1**, and the activities of these compounds in the in vitro ATPase and cell-based Ub^G76V^–GFP degradation assays^23^ are shown in full in Supporting Information tables S1 and S2. We began by examining the aromatic substitution, with the greatest number of analogues focused on substitution of the aryl ring at the 2-position of the quinazoline core. A few analogues exhibited modestly greater potency in vitro (e.g. compounds **2** and **3**, Table 1), but none of the derivatives performed better than the parent compound in both the biochemical and cell-based assays. The most dramatic effects arose from chloro substitution at the 7-position on the quinazoline core structure, which caused a 10–23-fold decrease in biochemical potency (e.g. **4** and **5**, Table 1). Replacing the aryl group with a selection of aliphatic heterocycles consistently decreased activity by 10–20-fold (e.g. **6** and **7**, Table 1). The *N*-methyl analogue **8** is typical for alkyl-substituted R^3^ analogues, possessing potency far below that of the HTS hit compounds.

**Table 1:** Selected SAR for the initial analogues of the HTS hits 1 and DBeQ.

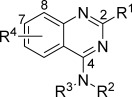					IC_50_ [μm]^a^	
Compd	R^1^	R^2^	R^3^	R^4^	ATPase	Ub^G76V^–GFP
**2**	2-chlorophenyl	benzyl	H	H	1.2±0.6	8±3
**3**	3-nitrophenyl	benzyl	H	H	5.9±3	4.0±1.6
**4**	phenyl	benzyl	H	7-Cl	70±24	12±4
**5**	*p*-tolyl	benzyl	H	7-Cl	39±13	24±5
**6**	piperidnyl	benzyl	H	H	26±4	7.3±2
**7**	morpholinyl	benzyl	H	H	23±6	36±9
**8**	phenyl	benzyl	Me	H	49±17	17±5
**9**	*N*-aminophenyl	phenyl	H	H	2.6±0.8	7.2±1
**10**^b^	*N*-aminophenyl	benzyl	H	H	2.3±1	3.1±0.4
**11**	*N*-amino(3-chlorophenyl)	benzyl	H	H	0.48±0.16	7.8±1.3
**12**	*N*-amino(3-chlorophenyl)	4-fluorobenzyl	H	H	1.5±0.3	5.7±1.3
**13**	*N*-amino(4-methoxybenzyl)	benzyl	H	H	3.0±0.5	1.3±0.2
**14**	*N*-aminobenzyl	benzyl	H	8-OMe	0.6±0.06	10±2

a Measurements were carried out in triplicate, and results are expressed as the mean ±SD.

b Screened as the HCl salt.

**Scheme 1:**
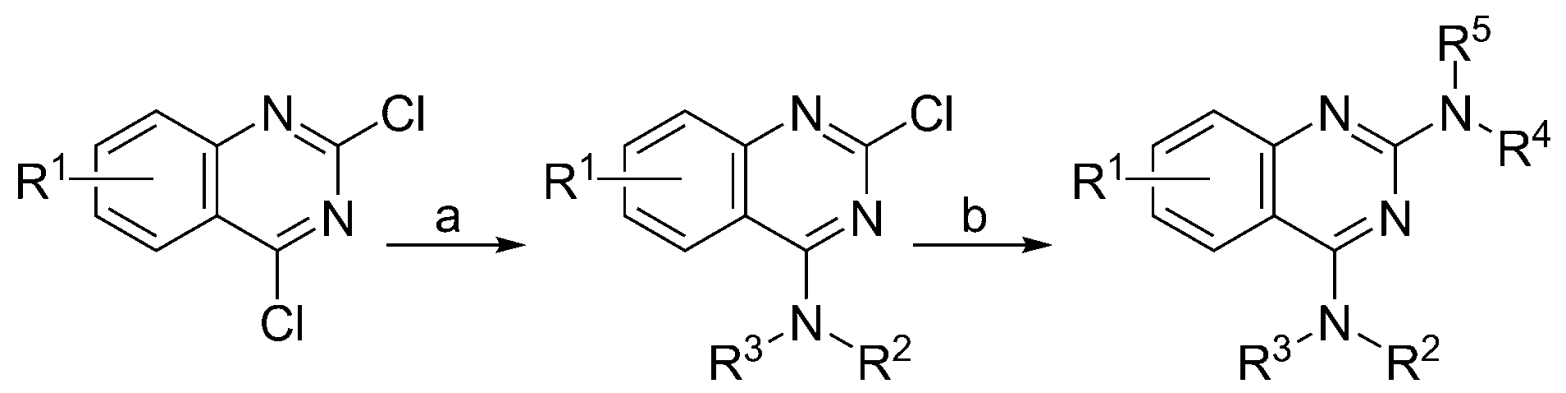
General synthetic route to DBeQ analogues. *Reagents and conditions:* a) R^2^R^3^NH, Et_3_N, CH_3_CN, RT, 16 h; b) R^4^R^5^NH, CH_3_CN, microwave irradiation, 180 °C, 1 h.

We next focused on purchasing and synthesizing compounds related to DBeQ (see Supporting Information tables S3–S6 for a complete listing of analogues and corresponding p97 inhibition values). The target analogues were prepared via the general synthetic route in Scheme 1, which features a modular approach where N2 and N4 can be varied independently (individual experimental details and characterization for all new compounds can be found in the Experimental Section below, or in the Supporting Information). We first replaced the N2 and N4 benzyl groups with phenyl groups, resulting in a slight decrease in potency (Table 1, **9**). Incorporation of substituents on the phenyl rings had a more substantial negative effect on p97 inhibition (Supporting Information table S3). Replacing only the N2 benzyl group of DBeQ with an N2 phenyl group also resulted in decreased potency (Table 1, **10**); however, in this case substitution on the N2 phenyl did not have the serious deleterious effect as before. In fact, a number of halide-containing analogues possessed improved potency in the enzymatic ATPase assay (e.g. compound **11**, Table 1, and Supporting Information table S4). The ATPase potency gains of these analogues were offset by a slight decrease in potency in the Ub^G76V^–GFP degradation assay. Nonetheless, encouraged by these initial results, we next explored the effect of substitution on the N4 benzyl group while incorporating the 3-chlorophenyl substitution of **11**. Neither electron-donating- nor electron-withdrawing-group-substituted analogues were able to match the potency of DBeQ in the cell-based Ub^G76V^–GFP degradation assay (Table 1 and Supporting Information table S5). Substitution of the N2 benzyl aromatic ring of DBeQ was also surveyed, affording several analogues possessing marginally better cell-based potency, most notably **13** (Table 1 and Supporting Information table S6).

In a complementary approach, we investigated the effect of substitution on the quinazoline core (Supporting Information table S7). The most potent compound incorporated a methoxy group at the 8-position of the quinazoline ring (Table 1, **14**) and exhibited a threefold improvement in ATPase inhibition counterbalanced with a fourfold erosion in the Ub^G76V^–GFP assay.

**Figure 3:**
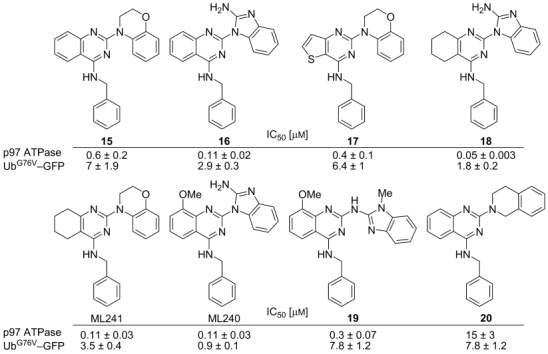
Structures and p97 inhibitory activities for key analogues in the development of ML240 and ML241. IC_50_ values for inhibition of p97 ATPase activity and degradation of p97-dependent reporter Ub^G76V^–GFP are shown.

Based on the results from varying the substitutions on the HTS hits **1** and DBeQ, we decided to explore more diverse moieties at the N2 position. Several constrained analogues were synthesized (for complete results, see Supporting Information tables S8 and S9), yielding two potent p97 inhibitors **15** and **16** (Figure 3) possessing in vitro ATPase IC_50_ values in the sub-micromolar range. Holding the N2 position substitution constant for each of these lead compounds, we turned our attention toward optimizing the quinazoline core. Initial efforts led to analogues with markedly different core structures possessing even better ATPase potency (e.g. **17** and **18**, Figure 3); however, these potency gains did not translate to improvements in the cell-based potency. Further modifications to the quinazoline core ultimately afforded two probe compounds ML241 and ML240 bearing different N2 position substitutions on distinct quinazoline core scaffolds (Figure 3 and Supporting Information tables S9 and S10). Although ML240 and ML241 exhibited similar potencies in the ATPase assay (IC_50_∼0.1 μm), ML240 was modestly more potent in the Ub^G76V^–GFP stabilization assay (IC_50_ 0.9 versus 3.5 μm). Exploration into replacements for the benzimidazole moiety of ML240 failed to yield superior analogues and was not pursued further (e.g. **19** and **20**, Figure 3 and Supporting Information tables S11 and S12). A survey of ML240 analogues examining substitution on the benzimidazole moiety (Table 2) revealed three compounds with improved ATPase potency (**27**, **29**, and **30**), although no analogues were found with improved cell-based potency. A survey of ML241 analogues covering substitution at the N4 position as well as modification of the quinazoline core is summarized in Table 3. Analogue **33** possessed activity approaching ML241 and several analogues with more radical modifications retained most of the ML241 activity (e.g. **17** and **41**). Even the severely truncated analogues **31** and **32** retained a portion of the in vitro inhibition. Analogous to the ML240 series, introduction of a methoxy group at the C8 position of the quinazoline core (Table 3, **38**) afforded an analogue of improved potency in the ATPase and Ub^G76V^–GFP assays (relative to **15**, Figure 3). A number of analogues for this series were synthesized with the specific aim to improve the aqueous solubility by introducing hydrophilic groups tethered to the phenol at the 8-position (e.g. **33**–**35** and **39**, Table 3). These efforts were largely successful, as the analogues retained most if not all of the potency observed in the probe molecule ML241.

**Table 2:** Selected SAR related to the optimization of ML240.

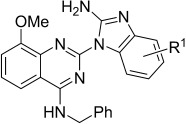		IC_50_ [μm]^a^	
Compd	R^1^	ATPase	Ub^G76V^–GFP
ML240	H	0.11±0.03	0.9±0.1
**21**	5,6-dichloro	25±8	>20
**22**	5,6-dimethyl	2±0.4	89±39
**23**	mixture of 5- and 6-chloro	0.7±0.3	31±7
**24**	mixture of 5- and 6-bromo	0.8±0.3	30±10
**25**	mixture of 5- and 6-methyl	0.24±0.06	2.5±0.5
**26**	mixture of 5- and 6-methoxy	0.48±0.09	0.96±0.2
**27**	4-fluoro	0.043±0.01	5.4±1.2
**28**	7-fluoro	0.5±0.1	5.4±0.8
**29**	4-methyl	0.045±0.006	2.6±0.3
**30**	mixture of 5- and 6-fluoro	0.08±0.02	6.8±1

a Measurements were carried out in triplicate, and results are expressed as the mean ±SD.

**Table 3:** Selected SAR related to the optimization of ML241.

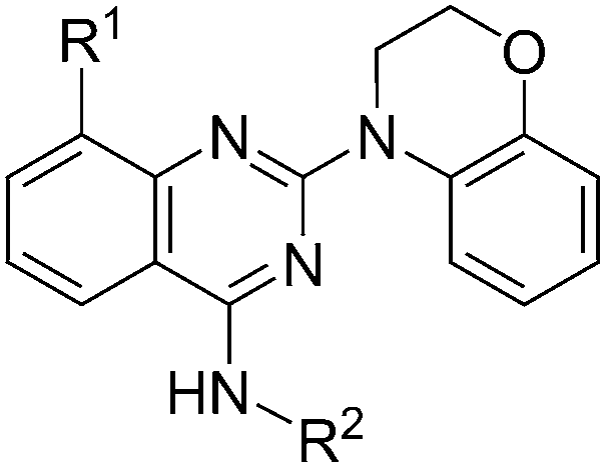			IC_50_ [μm]^a^	
Compd	R^1^	R^2^	ATPase	Ub^G76V^–GFP
ML241	see Figure 3		0.11±0.03	3.5±0.4
**31**	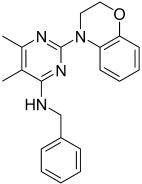		1.1±0.2	10±1
**32**	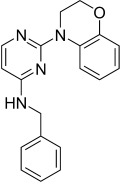		2.9±0.2	27±3
**33**	OCH_2_CH_2_OH	benzyl	0.17±0.05	3.8±0.8
**34**	OCH_2_CH_2_OMe	benzyl	0.6±0.03	6.5±0.7
**35**	OCH_2_CH_2_NEt_2_	benzyl	0.4±0.08	5.3±0.6
**36**	4-methoxyphenyl	benzyl	1.1±0.1	9±1
**37**	*n*-butoxy	benzyl	2.63±0.7	28±3
**38**	methoxy	benzyl	0.2±0.02	3.3±0.4
**39**	OCH_2_CN	benzyl	0.4±0.08	7.7±0.7
**40**	methoxy	4-fluorobenzyl	1.9±0.4	3.7±0.6
**41**	methoxy	thiophen-2-ylmethyl	1.2±0.2	3±0.6
**42**	methoxy	cyclohexylmethyl	7.4±0.9	8.2±1
**43**	methoxy	2-hyrdoxylbenzyl	4.6±1	6±0.6

a Measurements were carried out in triplicate, and results are expressed as the mean ±SD.

### Cell-based on-target, off-target, and antiproliferative activities of the top 22 compounds

The top compounds that emerged from the main SAR effort were next tested for their ability to retard degradation of the p97-independent proteasome substrate ODD-Luc^23^ (Table 4). ODD-Luc is targeted to the proteasome via the CRL2^VHL^ ubiquitin ligase pathway. To confirm that compounds did not interfere with measurement of luciferase activity, western blot analysis of ODD-Luc degradation was performed in parallel (Supporting Information figure S1). Both probe compounds and DBeQ were more than 10-fold less potent at stabilizing this substrate. A similar trend was observed for most of the ML240/ML241 analogues tested, with only **19** and, to a lesser extent, **17** appreciably blocking ODD-Luc degradation. The DBeQ analogues were slightly less selective, most notably **14** and **52**. Compound **52** is likely to block other components within the ubiquitin proteasome system.

**Table 4:** Compilation of further characterization experiments on promising p97 inhibitor analogues.

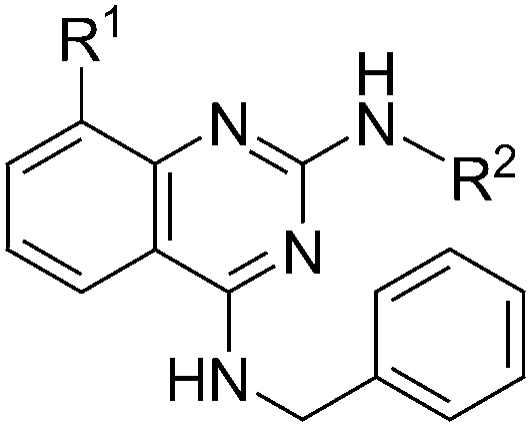			IC_50_ [μm]^a^			Cytotoxicity GI_50_ [μm]^a^			
						HCT15		SW403	
Compd	R^1^	R^2^	ATPase	Ub^G76V^–GFP	ODD-Luc	24 h	72 h	24 h	72 h
DBeQ	see Figure 1		1.6	2.3	56±14	3.2±1.0	1.8±0.7	2.2±0.6	1.4±0.5
**11**^c^	see Table 1		0.4	7.8	NM^b^	5±1	2.5±0.6	5.4±2	4.1±1.7
**14**^c^	see Table 1		0.6	10	6.5±1.1	1.1±0.3	0.8±0.3	0.8±0.3	0.5±0.2
**44**^c^	H	3-fluorophenyl	1.6	2.7	NM^b^	5.8±1.6	3.1±0.9	5.6±2.4	4.3±1.8
**45**^c^	H	4-methylbenzyl	3	1.5	16±5	3.6±1.2	3.5±1.2	3.3±1.1	2±0.7
**46**^c^	H	4-trifluoromethylbenzyl	2.6	2.5	15±3	3.4±1.1	2±1	3.0±1	1.6±0.7
**47**^c^	H	3-methoxybenzyl	4.5	1.7	>20	4.4±1.6	3.9±1.5	4.7±2.2	3±0.9
**48**^c^	H	3-fluorobenzyl	3.1	1.1	11±3	3.4±1	3.5±1.2	4.6±2	2.6±0.7
**49**^c^	H	3-bromobenzyl	3.7	1.3	12±3	3.4±1.2	3.5±1.2	4.3±1.9	2.8±0.7
**50**^c^	methoxy	3-chlorophenyl	0.5	5.4	18±4	29±6	19±4	12±5	11±5
**51**^c^	methoxy	3-fluorophenyl	0.5	4.8	22±4	8.0±2.7	3.7±1.3	4.9±2.0	4±1.6
**52**^c^	methoxy	thiophen-2-ylmethyl	1.7	1.8	4±1	0.82±0.17	0.7±0.3	0.5±0.1	0.35±0.1
ML240	see Figure 3		0.1	0.9	28±7	0.76±0.14	0.54±0.19	0.5±0.07	0.5±0.1
**19**^d^	see Figure 3		0.3	7.8	10±2	1.7±0.8	1.4±0.5	1.7±0.7	1.4±0.5
ML241	see Figure 3		0.1	3.5	46±8	53±5	13±4	33±7	12±3
**15**^e^	see Figure 3		0.6	7	>20	24±3	10±3	21±5	13±4
**17**^e^	see Figure 3		0.4	6.4	17±2	37±7	16±5	18±10	9.7±3.7
**33**^e^	see Table 3		0.1	3.8	>20	28±3	17±5	42±9	9.4±2.9
**34**^e^	see Table 3		0.6	6.5	27±5	12±4	9±3	15±7	7.9±2.7
**35**^e^	see Table 3		0.4	5.3	61±6	13±5	7.1±2.5	11±5	3.9±1
**38**^e^	see Table 3		0.2	3.3	>20	25±5	12±3	43±11	13±4
**40**^e^	see Table 3		1.9	3.7	>20	27±4	8.9±2.3	21±6	9.4±2.6

a Measurements were carried out in triplicate, and results are expressed as the mean ±SD.

b Not measured due to interference with luciferase assay.

c Analogue of DBeQ.

d Analogue of ML240.

e Analogue of ML241.

**Figure 4:**
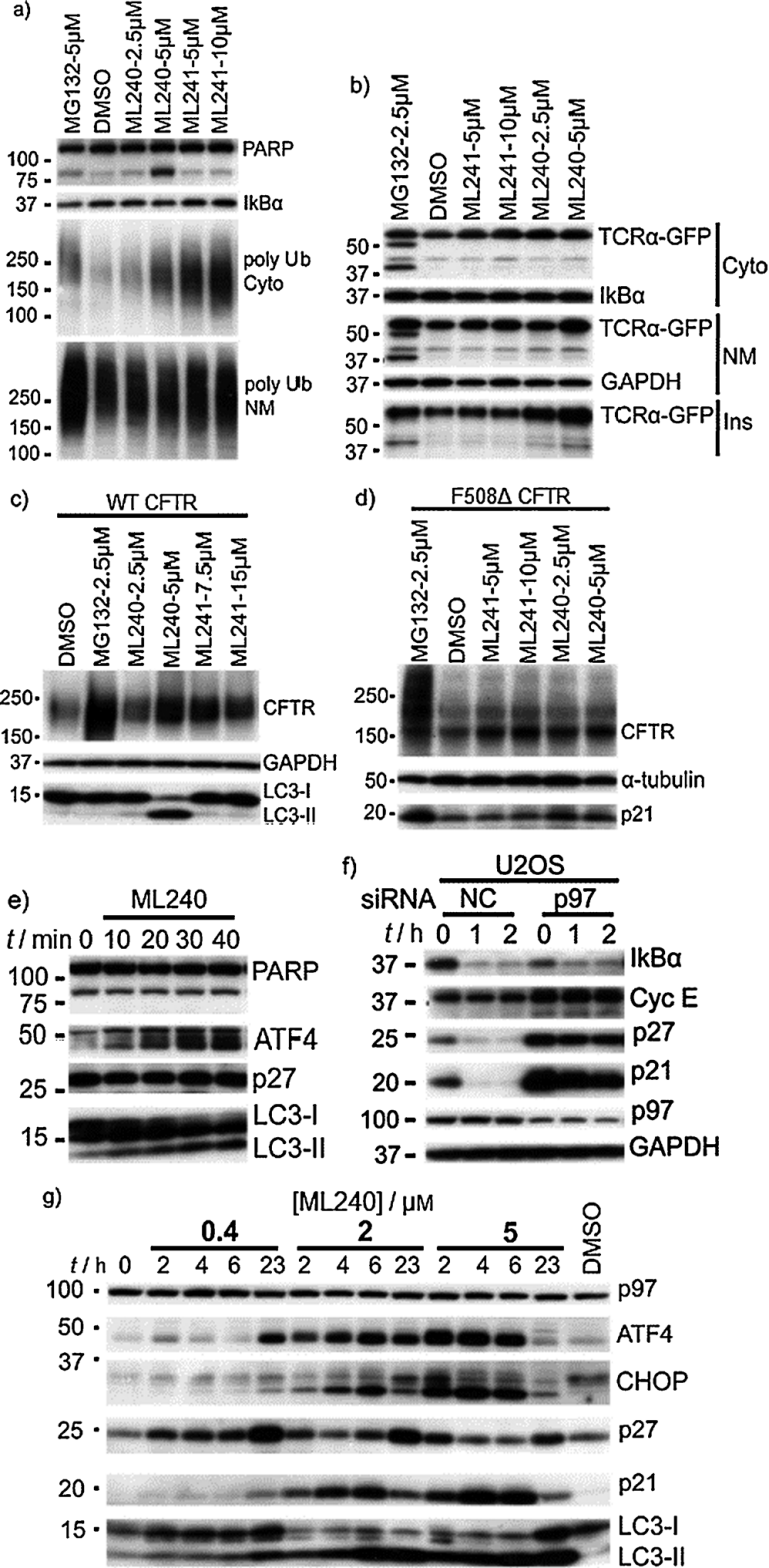
ML240 and ML241 impair the endoplasmic-reticulum-associated degradation (ERAD) pathway, and only ML240 impairs the autophagy pathway and induces apoptosis. a) SW403 cells were treated with DMSO or compounds for 2 h. The indicated proteins were evaluated by immunoblotting cytosolic (Cyto) and nuclear plus membrane (NM) fractions. b) HEK293 cells stably expressing TCRα–GFP were used to determine the effect of ML240 and ML241 on the ERAD pathway. Cells were treated with MG132, washed, and then incubated in the presence of cycloheximide plus compounds for 2.5 h. The indicated proteins were evaluated by immunoblotting cytosolic (Cyto), nuclear plus membrane (NM) and insoluble (Ins) fractions. c) HEK293 cells were transfected with wild-type CFTR cDNA and treated with DMSO or compounds for 5 h. Cytosolic fractions were immunoblotted to detect CFTR, GAPDH, and LC3. d) HEK293 cells were transfected with F508Δ CFTR cDNA and treated with DMSO or compounds for 5 h. Cytosolic fractions were immunoblotted to detect CFTR, α-tubulin, and p21. e) HCT116 cells were treated with ML240 (10 μm) for 0–40 min, and samples were immunoblotted to detect the indicated proteins (ATF4: nuclear plus membrane fraction, p27 and LC3: cytosolic fraction). f) U2OS cells were transfected with negative control siRNA (NC) or p97 siRNA (10 nm) for 72 h, and degradation of the indicated proteins was determined by immunoblotting of total cell extracts after addition of cycloheximide (CHX) for 0, 1, and 2 h. g) HT29 cells were incubated with ML240 (0.4, 2, or 5 μm) for 0 to 23 h. The levels of the indicated proteins were determined by immunoblotting. (ATF4, CHOP, p27, and p21: nuclear plus membrane fraction, and LC3: cytosolic fraction).

We next assayed the antiproliferative activity of the compounds on two colon cancer cell lines (HCT15 and SW403) after 24 or 72 h treatment (Table 4). To our surprise, ML240 and ML241 exhibited strikingly different antiproliferative activities. Whereas ML240 was only fourfold more potent than ML241 at stabilizing Ub^G76V^–GFP, it was 24- to 70-fold more potent at blocking cell proliferation. ML240, but not ML241, also induced efficient cleavage of the caspase substrate, poly(ADP-ribose) polymerase (PARP; Figure 4A), suggesting that its effects on cell proliferation were accompanied by induction of apoptosis.

### Inhibition of p97 by ML240 and ML241 is ATP competitive

**Figure 5:**
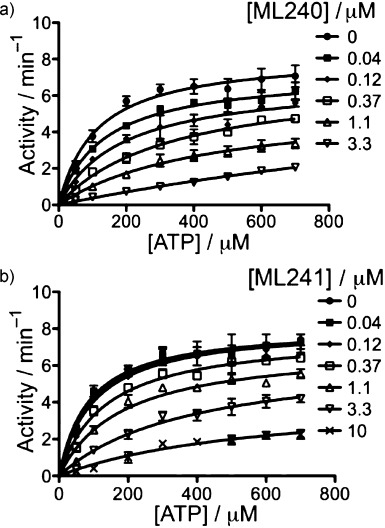
Michaelis–Menten plots for inhibition of p97 ATPase activity by a) ML240 and b) ML241.

To determine the mechanism by which ML240 and ML241 inhibited p97 ATPase, we evaluated rates of ATP hydrolysis at different concentrations of ATP. ML240 and ML241 inhibited p97 competitively with respect to ATP with a *K*_i_ values of 0.22 μm (Figure 5a) and 0.35 μm (Figure 5b), respectively.

### Evaluation of the effects of ML240 and ML241 on the ERAD and autophagy pathways

Consistent with the role of p97 in the ubiquitin–proteasome system (UPS), ML240 and ML241, like the parent compound DBeQ, caused accumulation of ubiquitin conjugates in the nuclear plus membrane and cytosolic compartments at concentrations of 5–10 μm (Figure 4a). Interestingly, ML241 caused stronger accumulation than ML240, even though ML240 was more potent at stabilizing Ub^G76V^–GFP. This, along with the cell proliferation data, points to unexpected complexity in the mechanism of action of these compounds. In addition to its effect on ubiquitin conjugates, DBeQ inhibits both the ERAD and autophagy pathways.^22^ We therefore sought to evaluate the impact of ML240 and ML241 on the ERAD reporters TCRα–GFP (α chain of the T-cell receptor fused to GFP) and F508Δ CFTR (cystic fibrosis transmembrane conductance regulator), as well as the autophagy reporter LC3-II. TCRα–GFP overexpressed in non-T-cells inserts into the endoplasmic reticulum (ER), but behaves as an unfolded protein and is degraded by the proteasome in a p97-dependent manner.^24^ ML241 had a modest effect on TCRα–GFP, but ML240 caused more substantial accumulation of this reporter, particularly in the insoluble (Ins) fraction, and to a lesser extent in the “nucleus plus membrane” (NM) fraction (Figure 4b). Furthermore, both compounds promoted accumulation of wild-type and the F508Δ mutant form of CFTR (albeit less strongly than MG132), which is normally rapidly degraded via the ERAD pathway (Figure 4c,d). Interestingly, only ML240 induced accumulation of the lipidated LC3-II species, which is indicative of a defect in autophagosome maturation (Figure 4c). Overall, ML240 behaved similarly to DBeQ in impinging on all of the p97-dependent processes we tested and inducing caspase activation and a decrease in cell proliferation.^22^ The lack of effect of ML241 on the autophagy pathway may explain why it did not cause rapid cell death. Most importantly, these data suggest that it is possible to develop pathway-specific inhibitors that inhibit distinct p97 functions.

### ML240 induces multiple markers diagnostic of p97 inhibition

The blockade in autophagosome maturation induced by ML240 was not an indirect consequence of apoptosis activation,^25^ because LC3-II accumulated as early as 10 min after addition of drug, whereas the drug-induced PARP cleavage shown in Figure 4a was not observed even after 40 min (Figure 4e). In parallel with the nearly immediate imposition of a block to autophagosome maturation, we observed rapid activation of the unfolded protein response (UPR) upon addition of ML240, as judged by accumulation of the ER stress-induced transcription factor, activating transcription factor 4 (ATF4; Figure 4e). Together, these data indicate that inhibition of p97 profoundly disrupts protein homeostasis within minutes.

To identify other markers for p97 inhibition, we surveyed the degradation of several UPS substrates—including IkBα, cyclin E, p21, and p27—in cells depleted of p97 by siRNA. The most dramatic effects were observed for p27 and p21, which were strongly stabilized in p97-depleted cells (Figure 4f). Consistent with the effects of p97 depletion,^22^ ML240 increased the levels of ATF4, CHOP, p27, p21, and LC3-II in concentration- and time-dependent manners (Figure 4g). The decreases in protein levels observed upon exposure to 5 μm ML240 for 23 h were potentially caused by cell death.

### ML240 induces executioner caspases 3 and 7 and triggers cell death independently of apical caspases 8 and 9

**Figure 6:**
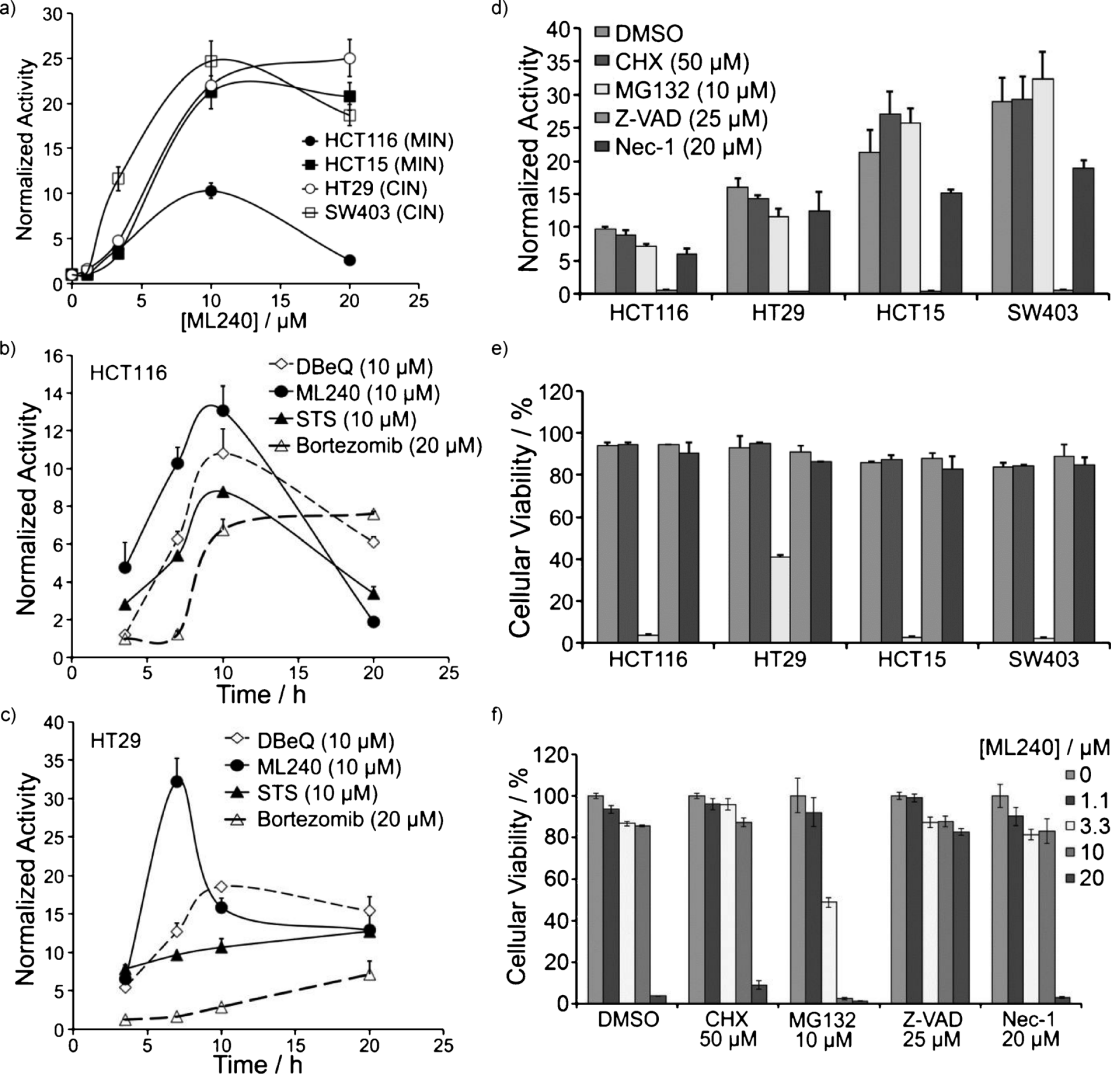
ML240 induces activation of caspases 3 and 7 and apoptosis. a) MIN and CIN colon cancer lines were treated with ML240 (1.1, 3.3, 10, or 20 μm) for 7 h prior to determination of caspase 3 and 7 activities in cell extract. Caspase 3 and 7 activities were normalized to the intensity of DMSO treated cells. b) HCT116 cells were incubated with 10 μm DBeQ, ML240, STS (staurosporine) or 20 μm bortezomib for 3, 7, 10, or 20 h prior to determination of caspase 3 and 7 activities in the cell extract. c) Same as panel b, except HT29 cells were used. d) Colon cancer cell lines were treated with ML240 (10 μm) plus either DMSO, CHX (50 μm), MG132 (10 μm), Z-VAD (25 μm), or Nec-1 (20 μm) for 7 h prior to determination of caspase 3 and 7 activities in the cell extract. e) Same as panel d, except cellular viability was determined using CellTiter-Glo. f) Viability of HCT15 cells was determined using CellTiter-Glo after co-treatment with various concentrations of ML240 (0–20 μm) plus either DMSO, CHX (50 μm), MG132 (10 μm), Z-VAD (25 μm), or Nec-1 (20 μm) for 7 h.

In our prior study, 10 μm DBeQ rapidly promoted activation of the “executioner” caspases 3 and 7 in HeLa cells, which mimicked p97 depletion by siRNA.^22^ ML240 induced caspase activity in a dose- (Figure 6) and time-dependent (Figure 6b,c) manner in multiple colon cancer cell lines. By comparison, ML240 was more effective at inducing caspase activity than DBeQ (Figure 6b,c). Activation of caspases 3 and 7 by ML240 was specific, because it was blocked by the caspase inhibitor Z-VAD(OMe)FMK (Figure 6d). Interestingly, although the ability of ML240 to activate caspases 3 and 7 was not potentiated by MG132, these two compounds had a synergistic effect on cell proliferation (Figure 6e,f). The caspase inhibitor Z-VAD(OMe)FMK had a protective effect on cell proliferation, whereas the necroptosis inhibitor Necrostatin-1 (Nec-1)^26^ did not (Figure 6f). Therefore, the rapid cell death induced by ML240 is most likely elicited by the apoptotic pathway.

**Figure 7:**
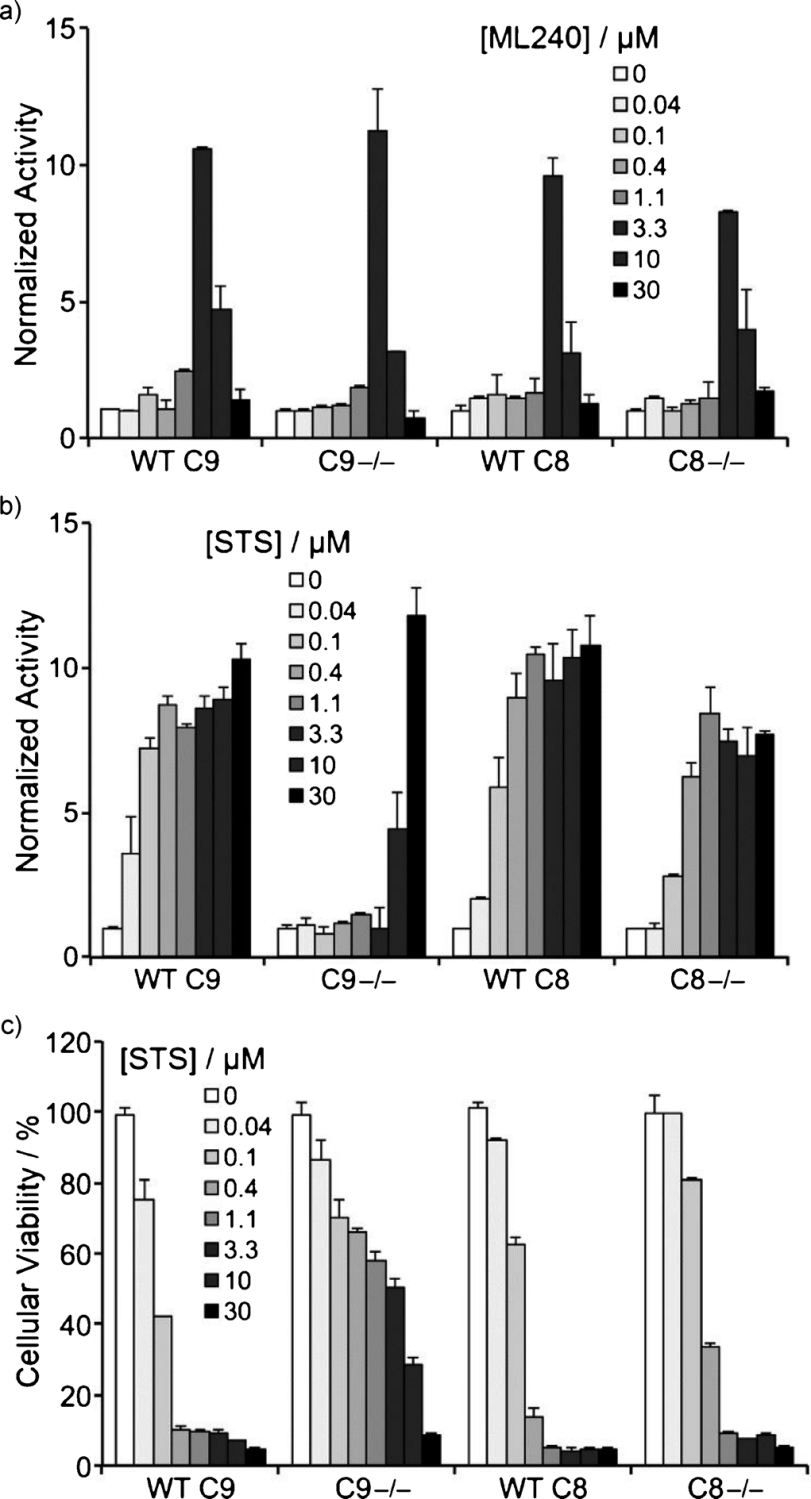
Cell death induced by ML240 is independent of caspases 8 and 9. Jurkat cells deficient in caspase 9 (C9−/−), C9−/− reconstituted with caspase 9 cDNA (WT C9), deficient in caspase 8 (C8−/−), or the parental clone (WT C8) were exposed to the indicated concentrations of a) ML240 or b) staurosporine (STS) for 8.5 h prior to determination of caspase 3 and 7 activities in the cell extract, and caspase 3 and 7 activities were normalized to the intensity of DMSO treated cells. c) Same as panel b, except incubation was for 24 h prior to determining cellular viability.

To evaluate the role of initiator caspases in activation of caspases 3 and 7 by ML240, we compared caspases 3 and 7 activation in four human Jurkat cell lines: caspase-9-deficient cells (C9−/−), C9−/− reconstituted with caspase 9 cDNA (WT C9), caspase-8-deficient cells (C8−/−), or the parental clone (WT C8) (Figure 7). ML240 (3.3 μm) activated caspases 3 and 7 by 10-fold within 8.5 h regardless of the status of initiator caspases 8 or 9 (Figure 7a). In contrast, staurosporine (STS) was much more effective at inducing caspases 3 and 7 (Figure 7b) and restricting cell proliferation (Figure 7c) when the intrinsic caspase 9 apoptotic pathway was intact. Taken together, our data suggest that ML240 does not induce caspases 3 and 7 via the apical caspases, but more directly impinges on the executioner caspases or their immediate regulators (e.g. IAP proteins). More work is required to understand how ML240 activates caspases 3 and 7, and how this relates to the inhibition of p97.

**Figure 8:**
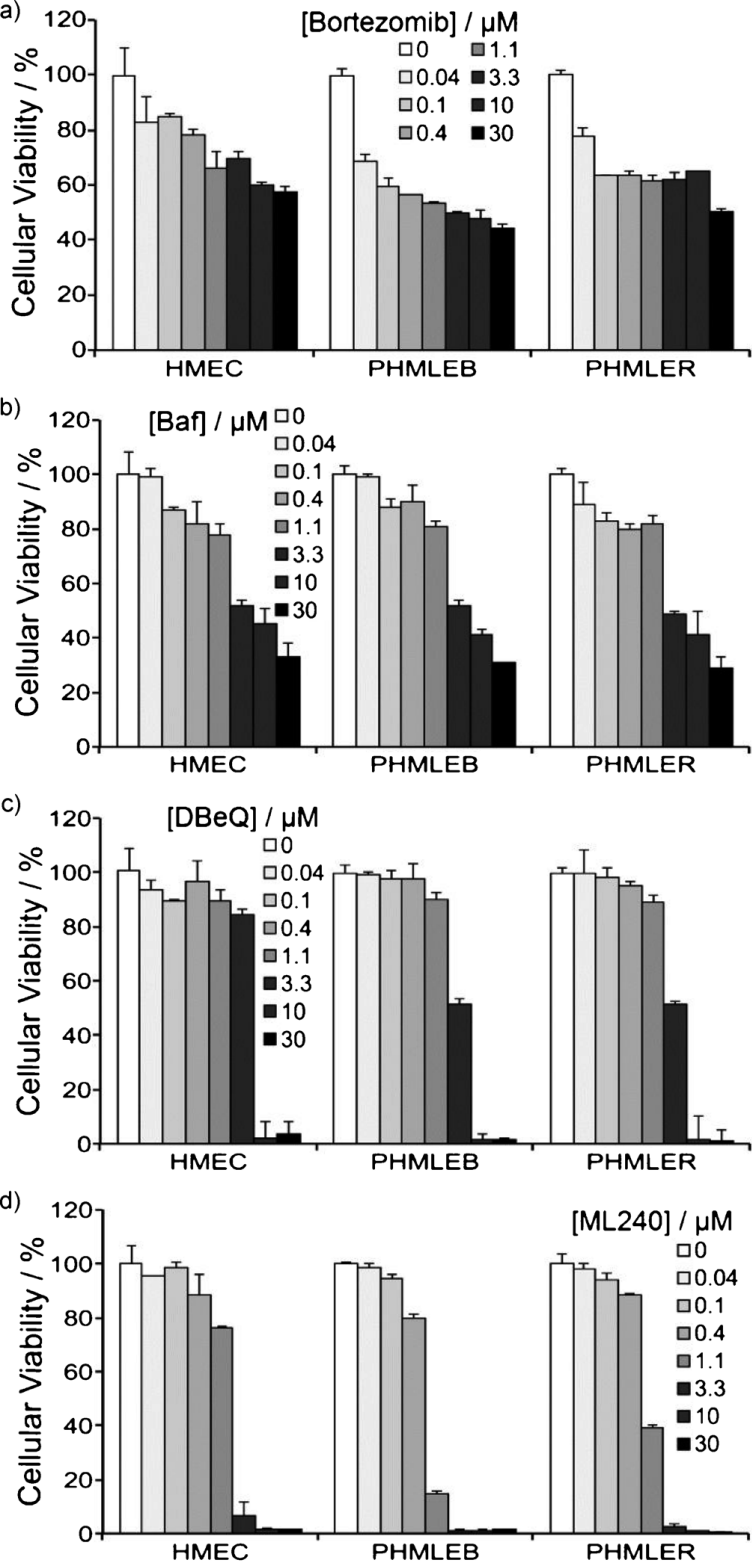
ML240 and DBeQ exhibit a modest therapeutic index. HMEC (primary human mammary epithelial cells), PHMLEB (sv40 and hTert immortalized), and PHMLER (H-Ras tumorigenic) cells were incubated with the indicated concentrations of a) bortezomib, b) bafilomycin (Baf), c) DBeQ, or d) ML240 for 24 h. Cellular viability was determined using CellTiter-Glo. Cellular viability was normalized to DMSO treated cells.

To determine whether the antiproliferative activity of ML240 might be selective for cancer cell lines, we employed HMEC (primary human mammary epithelial cells), PHMLEB (HMEC immortalized with SV40 and hTert), and PHMLER (PHMLEB transformed by H-Ras) cells.^27^ Both the proteasome inhibitor bortezomib and the autophagy inhibitor bafilomycin blocked proliferation of the normal, immortalized, and tumorigenic cell lines with equal potency (Figure 8a,b). In contrast, DBeQ (Figure 8c) and ML240 (Figure 8d) exhibited slightly greater potency toward the immortalized and transformed cells.

### Evaluation of ML240 and ML241 in the NCI 60-cell-line panel

The probe molecules ML240 and ML241 were assessed for inhibition of cell growth in the National Cancer Institute (NCI) 60-cell-line screen. The aggregate results for all 60 cell lines are summarized in Table 5 (see Supporting Information figures S2 and S3 for individual cell line results). The mean growth percent for ML240 was negative, indicating a decrease in tumor cell density, whereas ML241 was positive, indicating an increase in tumor cell density during the 48 h assay time period, although ML241 still displayed a slight decrease in overall growth rate relative to control. The striking difference in antiproliferative activity is likely a consequence of the aforementioned findings that ML240 activates the executioner caspases 3 and 7, while ML241 does not. A notable feature of the NCI-60 screen is that the growth inhibitory effects of ML240 cluster in a narrow range, suggesting that specific genotypes do not greatly influence cellular sensitivity to this compound.

**Table 5:** Aggregate NCI 60-cell-line screening results.

Compd	Mean *GP* [%]^a^	Lowest *GP* [%]^b^	Highest *GP* [%]^c^
ML240	−84.5±15.5	−100.0 (OVCAR-4)	−43.8 (HS 578T)
ML241	92.8±22.8	111.9 (HS 578T)	69.9 (MDA-MB-231/ATCC)

a Growth percent (*GP*) for individual cell lines calculated as follows: for negative growth, *GP*=100×(*OD*_test_−*OD*_T=0_)/*OD*_T=0_; for positive growth, *GP*=100×(*OD*_test_−*OD*_T=0_)/(*OD*_control_−*OD*_T=0_), for which *GP* is growth percent, *OD*_test_ is the optical density of the test culture after 48 h, *OD*_T=0_ is the optical density of the test culture before addition of compound, and *OD*_control_ is the optical density of the control culture after 48 h; values represent the mean of 60 NCI cell lines ±SD.

b Growth percent of the most inhibited individual cancer cell line.

c Growth percent of the least inhibited individual cancer cell line.

### Profiling of ML240 and ML241 in kinase/CNS panels and in vitro PK evaluation

The quinazoline scaffold at the heart of ML240 and ML241 is found in compounds that inhibit protein kinases.^28^ To uncover potential off-target effects of these compounds on protein kinases, kinase profiling was performed by using an activity-based proteomics platform (KiNativ, ActivX Biosciences, Inc., San Diego, CA, USA). This assay measures the ability of small molecules to inhibit the covalent labeling of protein kinases in native cell lysates by a broadly reactive ATP acyl-phosphate probe.^29^ As a positive control, we carried out a parallel analysis with the protein kinase inhibitor pyrazolopyrimidine (ACJI-47).^30^ The full results from these analyses are presented in Supporting Information table S14. Remarkably, ML241 (20 μm) did not appreciably inhibit labeling of any of the ∼170 kinases that were evaluated, whereas ML240 inhibited labeling of only three protein kinase domains by >50% when tested at 20 μm: PIP5 K3 (a member of the phosphoinositide-3 kinase family), JAK1 JH2 (N-terminal pseudokinase domain of JAK1), and DNA-dependent protein kinase (DNAPK). In contrast, pyrazolopyrimidine exhibited >50% inhibition of 56 protein kinases when tested at the same concentration. We therefore concluded that both ML240 and ML241 are quite specific, showing little activity against the targets assessed here.

**Figure 9:**
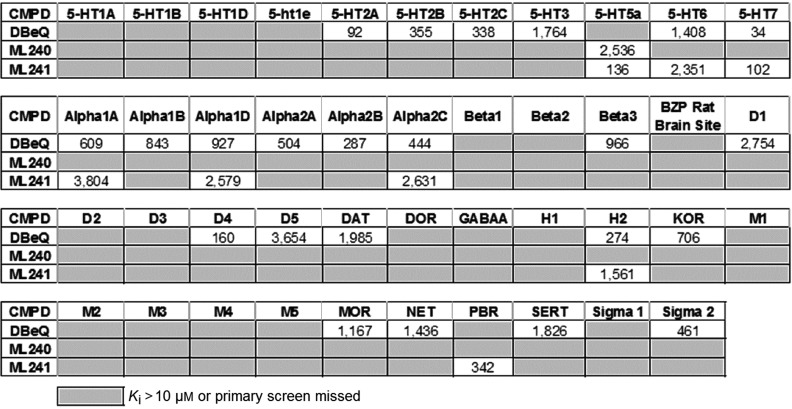
Binding affinity (*K*_i_ [nm]) for DBeQ, ML240, and ML241 toward 43 CNS-relevant targets.

In an effort to gauge the potential for off-target effects of these compounds, the binding affinity (*K*_i_ values) of DBeQ, ML240, and ML241 were determined for a panel of 43 CNS-relevant receptors and targets (Figure 9). While DBeQ was found to possess significant binding affinity (*K*_i_ values of <10 μm) for 23 targets (with 15 targets found to be <1 μm), ML240 was found to only have significant binding affinity for the 5HT5a receptor (*K*_i_=2.5 μm), and ML241 was found to possess significant binding affinity for eight targets (with only three targets <1 μm). The substantial improvement in selectivity for ML241 and the remarkably clean profile for ML240 bode well for their utility as in vivo probes and the therapeutic potential of this chemotype in general.

Finally, ML240 and ML241 were subjected to a panel of standard in vitro pharmacokinetic (PK) assays (Table 6). Encouragingly, both compounds possessed excellent plasma stability and acceptable human microsomal stability, with ML240 less susceptible to degradation than ML241. ML241 was also nontoxic toward Fa2N-4 immortalized human hepatocytes, although ML240 was less benign. In the parallel artificial membrane permeability assay (PAMPA), ML241 was again found to perform better than ML240, although this could be attributed to the adjusted assay conditions (addition of 20% acetonitrile). The aqueous solubility of ML241 was decent at low pH (pH 5.0) and poor at other pH values tested, whereas ML240 possessed poor solubility at all tested pH levels. Finally, both compounds were highly bound in the presence of plasma protein. The poor solubility and high plasma protein binding activity of these probes will need to be taken into consideration by researchers who use these compounds to investigate p97 function. To overcome these issues, we suggest that assays using these compounds should be carried out at a final concentration of 1% DMSO following 100-fold dilution from a DMSO stock. Cell-based experiments should be carried out in 2.5–5% FBS.

**Table 6:** Summary of in vitro pharmacokinetic properties for ML240 and ML241.

Compd	Aqueous solubility [μg mL^−1^]^a^	PAMPA *P*_e_ [×10^−6^ cm s^−1^]^b^	PPB [%]^c^		PS [%]^d^	HMS [%]^e^	LC_50_ [μm]^f^
			human	mouse			
ML240	0.35 (5.0)/0.33 (6.2)/0.27 (7.4)	357 (5.0)/628 (6.2)/<60.3 (7.4)	99.53/99.71	99.73/99.63	100/100	54.28/0.63	8.5
ML241	28 (5.0)/0.13 (6.2)/0.20 (7.4)	1164 (5.0)^g^/2504 (6.2)^g^/2278 (7.4)^g^	99.97/99.95	99.95/99.91	100/100	18.97/2.57	>50

a In aqueous buffer, pH 5.0/6.2/7.4.

b In aqueous buffer, donor compartment pH 5.0/6.2/7.4; acceptor compartment pH 7.4.

c Plasma protein bound, 1 μm/10 μm.

d Plasma stability, percent remaining at 3 h; human/mouse.

e Hepatic microsome stability, percent remaining at 1 h; human/mouse.

f Hepatic toxicity toward Fa2N-4 immortalized human hepatocytes.

g In the presence of 20% CH_3_CN.

## Conclusions

We developed two probes, ML240 and ML241, for the further study of p97 functions. The probes arose from a focused SAR campaign based on the 2,4-disubstituted quinazoline chemotype mined from the NIH compound collection (AID 1794, http://pubchem.ncbi.nlm.nih.gov/).^22^ The wide range of known cellular functions involving the p97 enzyme underscore the importance of developing selective p97 inhibitors, which would be highly useful to better understand the precise function of p97 in normal and aberrant physiological systems. Both probes are useful inhibitors of the p97 enzyme and share many similar structural elements; however, ML240, like the parent compound DBeQ,^22^ is capable of inhibiting the autophagic degradation pathway and activating caspases 3 and 7 and inducing rapid cell death, whereas ML241 is not. The differential ability of these compounds to induce apoptosis suggests either that rapid cell death was due to inhibition of the autophagic function of p97, or required simultaneous blockade of p97 function in both the autophagy and ubiquitin-proteasome pathways. Alternatively, apoptosis may be triggered by inhibition of a novel p97 function that has not been evaluated in our studies. Regardless of the precise explanation for the differential activity of these compounds, the more selective behavior of ML241 suggests that developing pathway-specific p97 inhibitors may prove to be a valuable approach to study the role of p97 in regulating a variety of cellular pathways.^31^ Pathway-selective p97 inhibitors with differential effects on apoptosis induction may enable the development of therapies that target p97 activity in diverse clinical settings, including IBMPFD (inclusion body myopathy associated with Paget disease of bone and frontotemporal dementia),^22^, ^11^ retinitis pigmentosa,^32^ and cancer.^33^ Understanding the basis of the divergent activities of ML240 and ML241 as well as potentially exploiting such effects toward the development of novel therapeutics are the aim of ongoing studies.

## Experimental Section

### Biology

*ATPase assay*: The detailed methodology was described previously.^22^ To avoid compounds forming colloids that cause nonspecific enzyme inhibition,^34^ inhibition of p97 was carried out in the assay buffer (50 mm Tris pH 7.4, 20 mm MgCl_2_, 1 mm EDTA, 0.5 mm TCEP) containing 0.01% Triton X-100. ATPase activity was determined by the addition of biomol green reagent (Enzo Life Sciences).

*Reporter degradation assay*: The dual reporter stable HeLa cell line that expresses the UFD reporter Ub^G76V^–GFP^35^ and the oxygen-dependent degradation domain of HIF1α fused to luciferase (ODD-Luc)^36^ was described previously.^23^ Cellular caspase activity, cellular viability, and western blot analysis were described previously.^22^

*Cell culture*: Colon cancer cell lines were maintained in DMEM supplemented with 5% FBS and antibiotics (Invitrogen). The human Jurkat T-cell line was maintained in RPMI 1640 medium supplemented with 5% FBS and antibiotics (Invitrogen). PHMLEB, PHMLER, and primary HMEC (Lonza) were propagated in MEGM mammary epithelial cell medium with mammary epithelial growth supplement (Invitrogen). Kinase profiling was described previously^22^ except that PC3 cell lysate was used.

### Chemistry

**General**: All reagents were used as received. CH_3_CN, CH_2_Cl_2_, toluene and THF were purified using the Innovative Technology PureSolv solvent purification system. ^1^H and ^13^C spectra were recorded on a Bruker Avance 400 or 500 MHz spectrometer. Chemical shifts (*δ*) are reported in parts per million and were referenced to residual proton solvent signals. Flash column chromatography separations were performed using the Teledyne Isco CombiFlash *R*_f_ using RediSep *R*_f_ silica gel columns. TLC was performed on Analtech Uniplate silica gel GHLF (gypsum inorganic hard layer with fluorescence) plates. TLC plates were developed using iodine vapor. Automated preparative RP HPLC purification was performed using an Agilent 1200 mass-directed fractionation system (prep pump G1361 with gradient extension, make-up pump G1311A, pH modification pump G1311A, HTS PAL autosampler, UV-DAD detection G1315D, fraction collector G1364B, and Agilent 6120 quadrupole spectrometer G6120A). The preparative chromatography conditions included a Waters X-Bridge C_18_ column (19×150 mm, 5 μm, with 19×10 mm guard column), elution with a H_2_O and CH_3_CN gradient, which increases to 20% CH_3_CN content over 4 min at a flow rate of 20 mL min^−1^ (modified to pH 9.8 through the addition of NH_4_OH by auxiliary pump), and sample dilution in DMSO. The preparative gradient, triggering thresholds, and UV wavelength were selected according to the analytical RP HPLC analysis of each crude sample. The analytical method used an Agilent 1200 RRLC system with UV detection (Agilent 1200 DAD SL) and mass detection (Agilent 6224 TOF). The analytical method conditions included a Waters Aquity BEH C_18_ column (2.1×50 mm, 1.7 μm) and elution with a linear gradient of 5% CH_3_CN in pH 9.8 buffered aqueous ammonium formate to 100% CH_3_CN at a flow rate of 0.4 mL min^−1^. Compound purity was measured on the basis of peak integration (area under the curve) from UV/Vis absorbance (*λ* 214 nm), and compound identity was determined on the basis of mass analysis. All compounds used for biological studies have purity >95% with the following exceptions: **18** (81.2%), **20** (83.1%), and **35** (68.2%). DBeQ was synthesized as previously described,^22^ and compounds **1**–**8**, **10**, and **11** were purchased from commercial vendors.

**General procedure A**: Representative protocol for the synthesis of quinazoline analogues, synthesis of **16**.

***N*****-Benzyl-2-chloroquinazolin-4-amine**: 2,4-dichloroquinazoline (2.4 g, 12.3 mmol) was suspended in THF (20 mL). Et_3_N (2.1 mL, 14.7 mmol) was added, followed by the addition of benzylamine (1.4 mL, 12.9 mmol). The mixture was stirred at room temperature for 16 h. The mixture was diluted with EtOAc, filtered, and the filtrate was concentrated. The residue was purified by silica gel chromatography to give the product as a white solid (1.6 g, 47%): *R*_f_=0.5 (EtOAc/hexanes, 1:3); ^1^H NMR (400 MHz, CDCl_3_): *δ*=7.87–7.73 (m, 2 H), 7.68 (d, *J*=8.2 Hz, 1 H), 7.54–7.32 (m, 6 H), 6.10 (s, 1 H), 4.90 ppm (d, *J*=5.3 Hz, 2 H).

**2-(2-Amino-1*H*-benzo[*d*]imidazol-1-yl)-*N*-benzylquinazolin-4-amine (16)**: A suspension of *N*-benzyl-2-chloroquinazolin-4-amine (0.015 g, 0.056 mmol) and 1*H*-benzo[*d*]imidazol-2-amine (0.015 g, 0.111 mmol) in CH_3_CN (1 mL) was heated in a synthesis microwave at 180 °C for 1 h. The solvent was removed in vacuo and the crude sample was purified via reversed-phase preparative HPLC to give **16** as a white solid (6.8 mg, 33%): ^1^H NMR (400 MHz, [D_6_]DMSO): *δ*=9.42 (t, *J*=5.8 Hz, 1 H), 8.41 (d, *J*=8.1 Hz, 1 H), 8.12 (d, *J*=7.5 Hz, 1 H), 7.94–7.79 (m, 4 H), 7.54–7.50 (m, 1 H), 7.45 (d, *J*=7.1 Hz, 2 H), 7.37 (t, *J*=7.6 Hz, 2 H), 7.27 (t, *J*=7.3 Hz, 1 H), 7.15 (d, *J*=7.1 Hz, 1 H), 7.03 (td, *J*=1.2, 7.6 Hz, 1 H), 6.88–6.76 (m, 1 H), 4.91 ppm (d, *J*=5.8 Hz, 2 H); ^13^C NMR (126 MHz, [D_6_]DMSO): *δ*=160.7, 154.5, 154.1, 148.9, 142.8, 138.4, 133.7, 131.7, 128.5, 127.0, 126.6, 125.1, 123.0, 122.5, 118.7, 114.8, 112.5, 44.4 ppm; HRMS-ESI: *m*/*z* [*M*+H]^+^ calcd for C_22_H_19_N_6_: 367.1671, found: 367.1685; HPLC purity: 96.1%.

**General procedure B**: Representative protocol for the synthesis of 8-*O*-alkylation analogues, synthesis of **39**.

**2-(2*H*-Benzo[*b*][1,4]oxazin-4(3*H*)-yl)-4-(benzylamino)quinazolin-8-ol**: To a solution of 2-(2*H*-benzo[*b*][1,4]oxazin-4(3*H*)-yl)-*N*-benzyl-8-methoxyquinazolin-4-amine (2.6 mg, 6.5 mmol) in CH_2_Cl_2_ (0.5 mL) at 0 °C, was added BBr_3_ (39 mL, 39 mmol, 1 m in CH_2_Cl_2_). The mixture was stirred at room temperature for 16 h. The reaction was quenched with slow addition of H_2_O. After extraction with CH_2_Cl_2_, the organic layer was dried over MgSO_4_, evaporated under vacuum to give the product as a brown oil (19.0 mg, 62%): ^1^H NMR (400 MHz, CDCl_3_): *δ*=7.88 (s, 1 H), 7.30 (d, *J*=4.6 Hz, 4 H), 7.28–7.21 (m, 1 H), 7.09–7.06 (m, 2 H), 7.02–6.88 (m, 2 H), 6.86 (d, *J*=6.5 Hz, 1 H), 6.79–6.74 (m, 1 H), 4.76 (d, *J*=5.4 Hz, 2 H), 4.23 ppm (s, 4 H); HRMS-ESI: *m*/*z* [*M*+H]^+^ calcd for C_23_H_21_N_4_O_2_: 385.1665, found: 385.1657.

**2-((2-(2*H*-Benzo[*b*][1,4]oxazin-4(3*H*)-yl)-4-(benzylamino)quinazolin-8-yl)oxy)acetonitrile (39)**: To a suspension of 2-(2*H*-benzo[*b*][1,4]oxazin-4(3*H*)-yl)-4-(benzylamino)quinazolin-8-ol (0.012 g, 0.031 mmol) and K_2_CO_3_ (0.013 g, 0.094 mmol) in DMF (1 mL), was added 2-chloroacetonitrile (3.0 μL, 0.047 mmol). The mixture was stirred at 80 °C for 16 h. The reaction was monitored by LC–MS. The reaction was complete after 16 h. DMF was removed under vacuum. The product was purified by silica gel chromatography to afford a brown solid (3.3 mg, 25%): *R*_f_=0.6 (EtOAc/hexanes, 1:2); ^1^H NMR (400 MHz, CDCl_3_): *δ*=7.99 (dd, *J*=1.5, 8.3 Hz, 1 H), 7.34–7.29 (m, 3 H), 7.29–7.22 (m, 4 H), 7.07–7.01 (m, 1 H), 6.93–6.81 (m, 2 H), 6.79–6.71 (m, 1 H), 5.84 (s, 1 H), 5.11 (s, 2 H), 4.73 (d, *J*=5.4 Hz, 2 H), 4.31–4.25 (m, 2 H), 4.22 ppm (dd, *J*=3.1, 5.0 Hz, 2 H); ^13^C NMR (126 MHz, CDCl_3_): *δ*=159.8, 156.1, 150.2, 146.5, 144.6, 138.1, 128.9, 127.7, 124.9, 123.9, 121.6, 121.1, 119.4, 116.9, 116.8, 115.8, 112.6, 66.1, 56.5, 45.5, 42.2 ppm; HRMS-ESI: *m*/*z* [*M*+H]^+^ calcd for C_25_H_22_N_5_O_2_: 424.1773, found: 424.1783; HPLC purity: 96.0%.

***N***^**2**^,***N***^**4**^**-Diphenylquinazoline-2,4-diamine (9)**: 2-Chloro-*N*-phenylquinazolin-4-amine and aniline were reacted according to general procedure A to give **9** as a white solid (11.8 mg, 97%): ^1^H NMR (400 MHz, CDCl_3_): *δ*=7.79–7.71 (m, 6 H), 7.71–7.65 (m, 2 H), 7.48–7.42 (m, 2 H), 7.40 (br s, 1 H), 7.38–7.29 (m, 3 H), 7.26–7.20 (m, 1 H), 7.17 (br s, 1 H), 7.09–7.00 ppm (m, 1 H); ^13^C NMR (101 MHz, CDCl_3_): *δ*=158.2, 156.5, 152.0, 140.0, 138.3, 133.2, 129.0, 128.8, 126.8, 124.5, 122.7, 122.1, 122.1, 120.4, 119.5, 111.6 ppm; HRMS-ESI: *m*/*z* [*M*+H]^+^ calcd for C_20_H_17_N_4_: 313.1453, found: 313.1451; HPLC purity: 98.8%.

***N***^**2**^**-(3-Chlorophenyl)-*N***^**4**^**-(4-fluorobenzyl)quinazoline-2,4-diamine (12)**: 2-Chloro-*N*-(4-fluorobenzyl)quinazolin-4-amine and 3-chloroaniline were reacted according to general procedure A to give **12** as a white solid (13.0 mg, 99%): ^1^H NMR (400 MHz, CDCl_3_): *δ*=8.00 (t, *J*=2.0 Hz, 1 H), 7.65–7.57 (m, 2 H), 7.54 (d, *J*=8.2 Hz, 1 H), 7.44–7.38 (m, 1 H), 7.35 (dd, *J*=5.3, 8.7 Hz, 2 H), 7.22–7.14 (m, 2 H), 7.09 (br s, 1 H), 7.06–6.99 (m, 2 H), 6.93 (ddd, *J*=0.9, 2.0, 7.9 Hz, 1 H), 5.89 (br s, 1 H), 4.80 ppm (d, *J*=5.2 Hz, 2 H); ^13^C NMR (101 MHz, CDCl_3_): *δ*=163.6, 161.1, 160.1, 156.4, 151.3, 141.6, 134.4, 133.93, 133.90, 133.1, 130.0, 129.54, 129.46, 126.6, 122.7, 121.6, 120.7, 118.9, 116.9, 115.8, 115.6, 111.5, 44.6 ppm; HRMS-ESI: *m*/*z* [*M*+H]^+^ calcd for C_21_H_17_ClFN_4_: 379.1126, found: 379.1123; HPLC purity: 100%.

***N***^**4**^**-Benzyl-*N***^**2**^**-(4-methoxybenzyl)quinazoline-2,4-diamine (13)**: 2-Chloro-*N*-benzylquinazolin-4-amine and 4-methoxybenzyl amine were reacted according to general procedure A to give **13** as a white solid (22.0 mg, 80%): ^1^H NMR (400 MHz, [D_6_]DMSO): *δ*=8.44 (s, 1 H), 8.03 (d, *J*=7.3 Hz, 1 H), 7.55–7.43 (m, 1 H), 7.43–7.11 (m, 8 H), 7.04 (t, *J*=7.1 Hz, 2 H), 6.82 (br s, 2 H), 4.74 (d, *J*=4.7 Hz, 2 H), 4.43 (br s, 2 H), 3.70 ppm (s, 3 H); ^13^C NMR (101 MHz, [D_6_]DMSO): *δ*=159.9, 159.8, 159.3, 157.8, 139.9, 133.2, 132.2, 129.2, 128.5, 128.2, 127.3, 126.6, 122.7, 119.9, 113.5, 113.4, 54.9, 43.4, 43.3 ppm; HRMS-ESI: *m*/*z* [*M*+H]^+^ calcd for C_23_H_23_N_4_O: 371.1872, found: 371.1868; HPLC purity: 96.8%.

***N***^**2**^,***N***^**4**^**-Dibenzyl-8-methoxyquinazoline-2,4-diamine (14)**: To a suspension of 2,4-dichloro-8-methoxyquinazoline (50.0 mg, 0.22 mmol) in CH_3_CN (2 mL) was added benzylamine (0.12 mL, 1.1 mmol, 5 equiv). The mixture was heated at 180 °C for 1 h under microwave irradiation. The solvent was removed under vacuum, the residue suspended in EtOAc, washed with saturated NaHCO_3_, and the layers were separated. The organic layer was dried over MgSO_4_ and concentrated under vacuum. The residue was purified by silica gel chromatography to give the product as a white solid (14.9 mg, 18%): *R*_f_=0.5 (EtOAc); ^1^H NMR (400 MHz, CDCl_3_): *δ*=7.44–7.18 (m, 11 H), 7.11 (dd, *J*=1.6, 7.9 Hz, 1 H), 7.07–6.93 (m, 2 H), 5.86 (br s, 1 H), 5.49 (br s, 1 H), 4.78 (d, *J*=5.7 Hz, 2 H), 4.75 (d, *J*=5.7 Hz, 2 H), 3.99 ppm (s, 3 H); ^13^C NMR (101 MHz, CDCl_3_): *δ*=160.1, 159.3, 153.2, 144.2, 140.2, 138.7, 128.7, 128.4, 128.0, 127.5, 126.8, 120.4, 112.5, 111.2, 110.9, 55.9, 45.7, 45.2 ppm; HRMS-ESI: *m*/*z* [*M*+H]^+^ calcd for C_23_H_23_N_4_O: 371.1872, found: 371.1871; HPLC purity: 98.9%.

**2-(2*H*-Benzo[*b*][1,4]oxazin-4(3*H*)-yl)-*N*-benzylquinazolin-4-amine (15)**: 2-Chloro-*N*-benzylquinazolin-4-amine and 3,4-dihydro-2*H*-benzo[*b*][1,4]oxazine were reacted according to general procedure A to give **15** as a light-yellow oil (27.0 mg, 99%): ^1^H NMR (400 MHz, CDCl_3_): *δ*=8.12 (d, *J*=8.3 Hz, 1 H), 7.68–7.61 (m, 2 H), 7.58 (d, *J*=8.2 Hz, 1 H), 7.45–7.30 (m, 5 H), 7.25–7.12 (m, 1 H), 7.03–6.89 (m, 2 H), 6.88–6.77 (m, 1 H), 5.94 (s, 1 H), 4.84 (d, *J*=5.5 Hz, 2 H), 4.39 (dd, *J*=3.1, 5.1 Hz, 2 H), 4.34 ppm (dd, *J*=3.1, 5.0 Hz, 2 H); ^13^C NMR (101 MHz, CDCl_3_): *δ*=159.8, 156.7, 151.7, 146.3, 138.5, 132.8, 128.8, 128.1, 127.7, 127.6, 126.8, 124.7, 123.4, 122.3, 120.7, 119.4, 116.7, 111.3, 66.2, 45.3, 42.2 ppm; HRMS-ESI: *m*/*z* [*M*+H]^+^ calcd for C_23_H_21_N_4_O: 369.1715, found: 369.1716; HPLC purity: 99.0%.

**2-(2*H*-Benzo[*b*][1,4]oxazin-4(3*H*)-yl)-*N*-benzylthieno[3,2-*d*]pyrimidin-4-amine (17)**: *N*-Benzyl-2-chlorothieno[3,2-*d*]pyrimidin-4-amine and 3,4-dihydro-2*H*-benzo[*b*][1,4]oxazine were reacted according to general procedure A to give **17** as a white solid (8.2 mg, 41%): ^1^H NMR (500 MHz, [D_6_]DMSO): *δ*=8.39 (t, *J*=5.9 Hz, 1 H), 8.01 (d, *J*=5.3 Hz, 1 H), 7.86 (dd, *J*=1.5, 8.3 Hz, 1 H), 7.36–7.29 (m, 4 H), 7.28–7.21 (m, 1 H), 7.19 (d, *J*=5.3 Hz, 1 H), 6.90–6.84 (m, 1 H), 6.82 (dd, *J*=1.7, 8.1 Hz, 1 H), 6.68 (ddd, *J*=1.7, 7.1, 8.6 Hz, 1 H), 4.66 (d, *J*=5.9 Hz, 2 H), 4.23–4.16 (m, 2 H), 4.15–4.13 ppm (m, 2 H); ^13^C NMR (126 MHz, DMSO): *δ*=160.9, 157.7, 156.7, 145.6, 139.7, 133.0, 128.3, 128.2, 127.0, 126.6, 124.3, 123.7, 122.7, 119.1, 116.2, 107.6, 65.3, 43.4, 42.1 ppm; HRMS-ESI: *m*/*z* [*M*+H]^+^ calcd for C_21_H_19_N_4_OS: 375.1280, found: 375.1284; HPLC purity: 95.3%.

**2-(2-Amino-1*H*-benzo[*d*]imidazol-1-yl)-*N*-benzyl-5,6,7,8-tetrahydroquinazolin-4-amine (18)**: *N*-Benzyl-2-chloro-5,6,7,8-tetrahydroquinazolin-4-amine and 1*H*-benzo[*d*]imidazol-2-amine were reacted according to general procedure A to give **18** as a white solid (1.8 mg, 12%): ^1^H NMR (400 MHz, [D_6_]DMSO): *δ*=7.92 (d, *J*=7.4 Hz, 1 H), 7.84 (s, 1 H), 7.65 (s, 2 H), 7.40–7.29 (m, 4 H), 7.23 (s, 1 H), 7.11 (d, *J*=7.1 Hz, 1 H), 6.99 (t, *J*=7.0 Hz, 1 H), 6.79–6.72 (m, 1 H), 4.71 (d, *J*=6.0 Hz, 2 H), 2.70 (br s, 2 H), 2.49 (br s, 2 H), 1.83 ppm (br s, 4 H); ^13^C NMR (126 MHz, DMSO): *δ*=161.0, 160.8, 154.3, 154.1, 142.8, 139.3, 131.6, 128.3, 126.7, 126.4, 122.2, 118.6, 114.6, 114.3, 108.6, 44.1, 31.3, 21.9, 21.7, 21.6 ppm; HRMS-ESI: *m*/*z* [*M*+H]^+^ calcd for C_22_H_23_N_6_: 371.1984, found: 371.1989; HPLC purity: 81.2%.

**2-(2*H*-Benzo[*b*][1,4]oxazin-4(3*H*)-yl)-*N*-benzyl-5,6,7,8-tetrahydroquinazolin-4-amine (ML241)**: *N*-Benzyl-2-chloro-5,6,7,8-tetrahydroquinazolin-4-amine and 3,4-dihydro-2*H*-benzo[*b*][1,4]oxazine were reacted according to general procedure A to give ML241 as a white solid (9.0 mg, 66%): ^1^H NMR (400 MHz, CDCl_3_): *δ*=8.09–8.01 (m, 1 H), 7.42–7.30 (m, 5 H), 6.92–6.86 (m, 2 H), 6.80–6.73 (m, 1 H), 4.79 (s, 1 H), 4.69 (d, *J*=5.6 Hz, 2 H), 4.28 (dd, *J*=3.1, 5.0 Hz, 2 H), 4.23 (dd, *J*=3.1, 5.0 Hz, 2 H), 2.67 (t, *J*=5.7 Hz, 2 H), 2.32 (t, *J*=5.7 Hz, 2 H), 1.93–1.77 ppm (m, 4 H); ^13^C NMR (101 MHz, CDCl_3_): *δ*=162.5, 160.7, 157.3, 145.9, 139.5, 128.6, 128.5, 127.5, 127.3, 123.9, 122.6, 119.5, 116.6, 103.8, 65.9, 45.0, 42.2, 32.2, 22.6, 22.5, 21.9 ppm; HRMS-ESI: *m*/*z* [*M*+H]^+^ calcd for C_23_H_25_N_4_O: 373.2028, found: 373.2030; HPLC purity: 98.3%.

**2-(2-Amino-1*H*-benzo[*d*]imidazol-1-yl)-*N*-benzyl-8-methoxyquinazolin-4-amine (ML240)**: *N*-Benzyl-2-chloro-8-methoxyquinazolin-4-amine and 1*H*-benzo[*d*]imidazol-2-amine were reacted according to general procedure A to give ML240 as a light-yellow oil (14.6 mg, 55%): ^1^H NMR (400 MHz, [D_6_]DMSO): *δ*=9.33 (t, *J*=5.9 Hz, 1 H), 8.31–8.05 (m, 3 H), 7.93 (d, *J*=7.5 Hz, 1 H), 7.50–7.39 (m, 3 H), 7.39–7.30 (m, 3 H), 7.25 (t, *J*=7.3 Hz, 1 H), 7.15 (d, *J*=7.1 Hz, 1 H), 7.03 (td, *J*=1.2, 7.6 Hz, 1 H), 6.87–6.76 (m, 1 H), 4.92 (d, *J*=5.8 Hz, 2 H), 3.98 ppm (s, 3 H); ^13^C NMR (101 MHz, [D_6_]DMSO): *δ*=160.8, 154.7, 153.5, 153.4, 142.8, 140.0, 138.5, 131.5, 128.5, 126.9, 126.6, 124.9, 122.5, 118.7, 114.8, 114.6, 114.1, 113.0, 112.7, 56.1, 44.5 ppm; HRMS-ESI: *m*/*z* [*M*+H]^+^ calcd for C_23_H_21_N_6_O: 397.1777, found: 397.1776; HPLC purity: 100%.

***N***^**4**^**-Benzyl-8-methoxy-*N***^**2**^**-(1-methyl-1*H*-benzo[*d*]imidazol-2-yl)quinazoline-2,4-diamine (19)**: To a suspension of *N*-benzyl-2-chloro-8-methoxyquinazolin-4-amine (15.0 mg, 50 mmol) in CH_3_CN (1 mL) was added 1-methyl-1*H*-benzo[*d*]imidazol-2-amine (14.7 mg, 0.1 mmol, 2 equiv). The mixture was heated at 180 °C for 10 h under microwave irradiation. The solvent was removed under vacuum, the residue was suspended in EtOAc, washed with saturated NaHCO_3_, and the layers were separated. The organic layer was dried over MgSO_4_ and concentrated under vacuum. The residue was purified by reversed-phase preparative HPLC to give **19** as a white solid (3.9 mg, 19%): ^1^H NMR (500 MHz, [D_6_]DMSO): *δ*=9.36 (s, 1 H), 8.28 (s, 1 H), 8.24 (d, *J*=8.0 Hz, 1 H), 7.93 (d, *J*=7.9 Hz, 1 H), 7.51 (s, 1 H), 7.50–7.39 (m, 2 H), 7.35 (td, *J*=2.7, 9.5 Hz, 3 H), 7.25 (t, *J*=7.3 Hz, 1 H), 7.16 (d, *J*=5.4 Hz, 2 H), 6.93 (s, 1 H), 4.88 (d, *J*=5.7 Hz, 2 H), 3.99 (s, 3 H), 3.42 ppm (s, 3 H); HRMS-ESI: *m*/*z* [*M*+H]^+^ calcd for C_24_H_23_N_6_O: 411.1933, found: 411.1937; HPLC purity: 96.9%.

***N*****-Benzyl-2-(3,4-dihydroisoquinolin-2(1*H*)-yl)quinazolin-4-amine (20)**: 2-Chloro-*N*-benzylquinazolin-4-amine and 1,2,3,4-tetrahydroisoquinoline were reacted according to general procedure A to give **20** as a white solid (8.0 mg, 59%): ^1^H NMR (400 MHz, CDCl_3_): *δ*=7.45 (d, *J*=3.6 Hz, 2 H), 7.41 (d, *J*=8.2 Hz, 1 H), 7.35 (d, *J*=7.3 Hz, 2 H), 7.33–7.21 (m, 3 H), 7.16–7.03 (m, 4 H), 7.03–6.89 (m, 1 H), 5.73 (br s, 1 H), 4.95 (s, 2 H), 4.78 (d, *J*=5.4 Hz, 2 H), 4.07 (t, *J*=5.9 Hz, 2 H), 2.84 ppm (t, *J*=5.9 Hz, 2 H); ^13^C NMR (101 MHz, CDCl_3_): *δ*=159.6, 139.0, 135.5, 134.9, 132.6, 128.7, 128.6, 127.9, 127.5, 126.6, 126.03, 125.95, 120.9, 120.7, 110.3, 46.4, 45.3, 41.5, 29.2 ppm; HRMS-ESI: *m*/*z* [*M*+H]^+^ calcd for C_24_H_23_N_4_: 367.1923, found: 367.1921; HPLC purity: 83.1%.

**2-(2-Amino-5,6-dichloro-1*H*-benzo[*d*]imidazol-1-yl)-*N*-benzyl-8-methoxyquinazolin-4-amine (21)**: *N*-Benzyl-2-chloro-8-methoxyquinazolin-4-amine and 5,6-dichloro-1*H*-benzo[*d*]imidazol-2-amine were reacted according to general procedure A to give **21** as a white solid (7.0 mg, 30%): ^1^H NMR (500 MHz, [D_6_]DMSO): *δ*=9.42 (s, 1 H), 8.55 (s, 1 H), 8.53–8.34 (m, 2 H), 7.93 (d, *J*=7.7 Hz, 1 H), 7.52–7.41 (m, 3 H), 7.38 (d, *J*=7.5 Hz, 1 H), 7.33 (dd, *J*=5.8, 9.4 Hz, 3 H), 7.25 (d, *J*=7.4 Hz, 1 H), 4.90 (d, *J*=5.8 Hz, 2 H), 3.98 ppm (s, 3 H); ^13^C NMR (126 MHz, [D_6_]DMSO): *δ*=160.7, 156.3, 153.5, 152.8, 143.2, 139.7, 138.0, 131.1, 128.5, 127.0, 126.6, 125.3, 124.7, 120.0, 115.9, 115.3, 114.1, 113.1, 112.9, 56.1, 44.5 ppm; HRMS-ESI: *m*/*z* [*M*+H]^+^ calcd for C_23_H_19_Cl_2_N_6_O: 424.1773, found: 465.1028; HPLC purity: 95.6%.

**2-(2-Amino-5,6-dimethyl-1*H*-benzo[*d*]imidazol-1-yl)-*N*-benzyl-8-methoxyquinazolin-4-amine (22)**: *N*-Benzyl-2-chloro-8-methoxyquinazolin-4-amine and 5,6-dimethyl-1*H*-benzo[*d*]imidazol-2-amine were reacted according to general procedure A to give **22** as a white solid (13.0 mg, 61%): ^1^H NMR (400 MHz, [D_6_]DMSO): *δ*=9.27 (t, *J*=6.0 Hz, 1 H), 8.09 (s, 2 H), 8.07 (s, 1 H), 7.90 (d, *J*=7.5 Hz, 1 H), 7.47–7.39 (m, 3 H), 7.39–7.30 (m, 3 H), 7.25 (t, *J*=7.3 Hz, 1 H), 6.94 (s, 1 H), 4.98 (d, *J*=5.9 Hz, 2 H), 3.98 (s, 3 H), 2.19 (s, 3 H), 2.04 ppm (s, 3 H); ^13^C NMR (101 MHz, [D_6_]DMSO): *δ*=160.9, 154.2, 153.5, 140.9, 140.2, 138.5, 130.0, 129.7, 128.5, 126.9, 126.5, 126.2, 124.7, 115.7, 115.5, 114.1, 112.8, 112.7, 56.1, 44.4, 19.7 ppm; HRMS-ESI: *m*/*z* [*M*+H]^+^ calcd for C_25_H_25_N_6_O: 425.2090, found: 425.2090; HPLC purity: 94.4%.

**2-(2-Amino-5-chloro-1*H*-benzo[*d*]imidazol-1-yl)-*N*-benzyl-8-methoxyquinazolin-4-amine and 2-(2-Amino-6-chloro-1*H*-benzo[*d*]imidazol-1-yl)-*N*-benzyl-8-methoxyquinazolin-4-amine (∼1:0.93) (23)**: *N*-Benzyl-2-chloro-8-methoxyquinazolin-4-amine and 5-chloro-1*H*-benzo[*d*]imidazol-2-amine were reacted according to general procedure A to give **23**, a white solid, as a mixture of regioisomers (10.2 mg, 47%): ^1^H NMR (400 MHz, [D_6_]DMSO): *δ*=9.39 (d, *J*=7.1 Hz, 2 H), 8.50–8.19 (m, 5 H), 8.07 (d, *J*=8.6 Hz, 1 H), 7.94 (d, *J*=8.2 Hz, 2 H), 7.54–7.41 (m, 6 H), 7.41–7.30 (m, 6 H), 7.30–7.19 (m, 2 H), 7.19–7.11 (m, 2 H), 7.07 (dd, *J*=2.1, 8.3 Hz, 1 H), 6.79 (dd, *J*=2.1, 8.6 Hz, 1 H), 4.92 (d, *J*=5.9 Hz, 2 H), 4.89 (d, *J*=5.8 Hz, 2 H), 3.99 (s, 3 H), 3.98 ppm (s, 3 H); ^13^C NMR (126 MHz, [D_6_]DMSO): *δ*=160.8, 155.8, 155.4, 153.51, 153.49, 153.1, 144.2, 141.7, 139.9, 139.8, 138.4, 138.1, 132.2, 130.3, 128.5, 127.0, 126.9, 126.7, 126.6, 126.5, 125.13, 125.08, 122.6, 122.4, 118.0, 115.6, 115.4, 114.8, 114.1, 113.1, 113.0, 112.9, 112.8, 56.1, 44.6, 44.5 ppm; HRMS-ESI: *m*/*z* [*M*+H]^+^ calcd for C_23_H_20_ClN_6_O: 431.1387, found: 431.1400; HPLC purity: 97.8%.

**2-(2-Amino-5-bromo-1*H*-benzo[*d*]imidazol-1-yl)-*N*-benzyl-8-methoxyquinazolin-4-amine and 2-(2-Amino-6-bromo-1*H*-benzo[*d*]imidazol-1-yl)-*N*-benzyl-8-methoxyquinazolin-4-amine (∼1:0.73) (24)**: *N*-Benzyl-2-chloro-8-methoxyquinazolin-4-amine and 5-bromo-1*H*-benzo[*d*]imidazol-2-amine were reacted according to general procedure A to give **24**, a white solid, as a mixture of regioisomers (12 mg, 50%): ^1^H NMR (400 MHz, [D_6_]DMSO): *δ*=9.30 (d, *J*=4.1 Hz, 2 H), 8.53 (d, *J*=2.0 Hz, 1 H), 8.27 (d, *J*=15.2 Hz, 4 H), 7.95 (d, *J*=8.6 Hz, 1 H), 7.86 (d, *J*=8.4 Hz, 2 H), 7.38 (ddd, *J*=3.9, 7.2, 11.2 Hz, 6 H), 7.28 (dt, *J*=6.3, 14.9 Hz, 6 H), 7.23–7.09 (m, 4 H), 7.02 (d, *J*=8.3 Hz, 1 H), 6.83 (dd, *J*=2.0, 8.6 Hz, 1 H), 4.84 (d, *J*=5.9 Hz, 2 H), 4.81 (d, *J*=5.8 Hz, 2 H), 3.91 (s, 3 H), 3.90 ppm (s, 3 H); ^13^C NMR (126 MHz, [D_6_]DMSO): *δ*=160.8, 160.7, 155.6, 155.3, 153.5, 153.5, 153.1, 144.7, 142.2, 139.9, 139.8, 138.4, 138.1, 132.7, 130.7, 128.5, 128.5, 127.0, 126.9, 126.7, 126.5, 125.2, 125.1, 120.8, 117.5, 116.9, 116.2, 116.0, 114.7, 114.1, 113.04, 113.03, 112.8, 112.8, 110.3, 56.1, 44.6, 44.5 ppm; HRMS-ESI: *m*/*z* [*M*+H]^+^ calcd for C_23_H_20_BrN_6_O: 475.0882 and 477.0862 (^81^Br), found: 477.0894; HPLC purity: 95.5%.

**2-(2-Amino-5-methyl-1*H*-benzo[*d*]imidazol-1-yl)-*N*-benzyl-8-methoxyquinazolin-4-amine and 2-(2-Amino-6-methyl-1*H*-benzo[*d*]imidazol-1-yl)-*N*-benzyl-8-methoxyquinazolin-4-amine (∼1:1) (25)**: *N*-Benzyl-2-chloro-8-methoxyquinazolin-4-amine and 5-methyl-1*H*-benzo[*d*]imidazol-2-amine were reacted according to general procedure A to give **25**, a white solid, as a mixture of regioisomers (13.7 mg, 67%): ^1^H NMR (400 MHz, [D_6_]DMSO): *δ*=9.22 (t, *J*=5.8 Hz, 2 H), 8.13–7.97 (m, 5 H), 7.94 (d, *J*=8.1 Hz, 1 H), 7.84 (d, *J*=8.3 Hz, 2 H), 7.36 (td, *J*=3.8, 7.9 Hz, 6 H), 7.28 (td, *J*=5.1, 7.6 Hz, 6 H), 7.18 (t, *J*=7.2 Hz, 2 H), 6.94 (d, *J*=7.9 Hz, 1 H), 6.88 (s, 1 H), 6.76 (d, *J*=6.8 Hz, 1 H), 6.54 (d, *J*=7.1 Hz, 1 H), 4.90 (d, *J*=5.9 Hz, 2 H), 4.83 (d, *J*=5.8 Hz, 2 H), 3.90 (s, 3 H), 3.90 (s, 3 H), 2.24 (d, *J*=7.5 Hz, 3 H), 2.08 ppm (s, 3 H); ^13^C NMR (101 MHz, [D_6_]DMSO): *δ*=160.9, 160.8, 154.8, 154.5, 153.5, 153.5, 153.4, 143.0, 140.6, 140.1, 140.1, 138.5, 138.4, 131.7, 131.3, 129.5, 128.48, 128.45, 127.4, 126.92, 126.88, 126.6, 126.5, 124.8, 124.7, 123.3, 119.5, 115.3, 115.1, 114.4, 114.2, 114.1, 112.9, 112.8, 112.69, 112.67, 56.1, 44.5, 44.4, 21.2, 21.1 ppm; HRMS-ESI: *m*/*z* [*M*+H]^+^ calcd for C_24_H_23_N_6_O: 411.1933, found: 411.1959; HPLC purity: 93.2%.

**2-(2-Amino-5-methoxy-1*H*-benzo[*d*]imidazol-1-yl)-*N*-benzyl-8-methoxyquinazolin-4-amine and 2-(2-Amino-6-methoxy-1*H*-benzo[*d*]imidazol-1-yl)-*N*-benzyl-8-methoxyquinazolin-4-amine (∼1:1) (26)**: *N*-Benzyl-2-chloro-8-methoxyquinazolin-4-amine and 5-methoxy-1*H*-benzo[*d*]imidazol-2-amine were reacted according to general procedure A to give **26**, a white solid, as a mixture of regioisomers (10.2 mg, 48%): ^1^H NMR (400 MHz, [D_6_]DMSOO): *δ*=9.21 (d, *J*=4.2 Hz, 2 H), 8.11 (d, *J*=2.5 Hz, 3 H), 7.97 (d, *J*=8.8 Hz, 1 H), 7.91 (s, 2 H), 7.84 (dd, *J*=4.8, 7.4 Hz, 2 H), 7.36 (ddd, *J*=1.7, 5.9, 9.7 Hz, 6 H), 7.28 (q, *J*=7.5 Hz, 6 H), 7.19 (dd, *J*=3.4, 7.3 Hz, 2 H), 6.98 (d, *J*=8.5 Hz, 1 H), 6.65 (d, *J*=2.5 Hz, 1 H), 6.59 (dd, *J*=2.6, 8.5 Hz, 1 H), 6.33 (dd, *J*=2.6, 8.8 Hz, 1 H), 4.88 (d, *J*=5.8 Hz, 2 H), 4.83 (d, *J*=5.8 Hz, 2 H), 3.904 (s, 3 H), 3.900 (s, 3 H), 3.66 (s, 3 H), 3.47 ppm (s, 3 H); ^13^C NMR (101 MHz, [D_6_]DMSO): *δ*=160.8, 160.7, 155.9, 155.3, 154.1, 153.6, 153.5, 153.4, 153.3, 153.2, 143.9, 140.2, 140.1, 138.5, 138.4, 136.7, 128.5, 128.5, 127.1, 127.0, 126.7, 124.9, 124.7, 115.0, 114.7, 114.1, 112.90, 112.87, 112.73, 112.69, 109.9, 105.4, 100.9, 99.6, 56.1, 55.2, 55.1, 44.53, 44.47 ppm; HRMS-ESI: *m*/*z* [*M*+H]^+^ calcd for C_24_H_23_N_6_O_2_: 427.1882, found: 427.1910; HPLC purity: 90.2%.

**2-(2-Amino-4-fluoro-1*H*-benzo[*d*]imidazol-1-yl)-*N*-benzyl-8-methoxyquinazolin-4-amine (27)**: *N*-Benzyl-2-chloro-8-methoxyquinazolin-4-amine and 4-fluoro-1*H*-benzo[*d*]imidazol-2-amine were reacted according to general procedure A to give **27** as a white solid (5.3 mg, 51%): ^1^H NMR (500 MHz, [D_6_]DMSO): *δ*=9.37 (t, *J*=5.9 Hz, 1 H), 8.33 (s, 2 H), 8.02–7.96 (m, 1 H), 7.94 (d, *J*=7.6 Hz, 1 H), 7.47 (t, *J*=8.1 Hz, 1 H), 7.43 (d, *J*=7.2 Hz, 2 H), 7.40–7.32 (m, 3 H), 7.25 (t, *J*=7.3 Hz, 1 H), 6.88 (dd, *J*=8.2, 9.9 Hz, 1 H), 6.76 (td, *J*=5.2, 8.1 Hz, 1 H), 4.90 (d, *J*=5.8 Hz, 2 H), 3.98 ppm (s, 3 H); ^13^C NMR (126 MHz, [D_6_]DMSO): *δ*=160.8, 154.9, 153.5, 153.2, 149.5, 139.8, 138.4, 134.3, 134.2, 128.5, 126.9, 126.6, 125.2, 118.7, 118.6, 114.1, 113.1, 112.8, 111.3, 108.8, 108.6, 56.1, 44.5 ppm; HRMS-ESI: *m*/*z* [*M*+H]^+^ calcd for C_23_H_20_FN_6_O: 415.1683, found: 415.1709; HPLC purity: 98.8%.

**2-(2-Amino-7-fluoro-1*H*-benzo[*d*]imidazol-1-yl)-*N*-benzyl-8-methoxyquinazolin-4-amine (28)**: The regioisomer of **27** was isolated by reversed-phase preparative HPLC from the above reaction mixture to give **28** as a white solid (2.1 mg, 20%): ^1^H NMR (400 MHz, [D_6_]DMSO): *δ*=9.20 (s, 1 H), 7.91 (d, *J*=7.5 Hz, 1 H), 7.62 (s, 2 H), 7.49 (t, *J*=8.1 Hz, 1 H), 7.37 (d, *J*=7.3 Hz, 1 H), 7.35–7.25 (m, 4 H), 7.23 (d, *J*=7.1 Hz, 1 H), 7.13–7.02 (m, 2 H), 6.85–6.75 (m, 1 H), 4.85 (d, *J*=5.8 Hz, 2 H), 3.96 ppm (s, 3 H); ^13^C NMR (126 MHz, [D_6_]DMSOO): *δ*=160.8, 154.9, 153.5, 153.2, 149.5, 139.8, 138.4, 134.3, 134.2, 128.5, 126.9, 126.6, 125.2, 118.7, 118.6, 114.1, 113.1, 112.8, 111.3, 108.8, 108.6, 56.1, 44.5 ppm; HRMS-ESI: *m*/*z* [*M*+H]^+^ calcd for C_23_H_20_FN_6_O: 415.1683, found: 415.1709; HPLC purity: 96.8%.

**2-(2-Amino-4-methyl-1*H*-benzo[*d*]imidazol-1-yl)-*N*-benzyl-8-methoxyquinazolin-4-amine (29)**: *N*-Benzyl-2-chloro-8-methoxyquinazolin-4-amine and 4-methyl-1*H*-benzo[*d*]imidazol-2-amine were reacted according to general procedure A to give **29** as a white solid (5.4 mg, 53%): ^1^H NMR (400 MHz, [D_6_]DMSO): *δ*=9.23 (t, *J*=5.9 Hz, 1 H), 8.11 (s, 2 H), 7.94 (d, *J*=7.8 Hz, 1 H), 7.85 (d, *J*=7.4 Hz, 1 H), 7.42–7.33 (m, 3 H), 7.28 (t, *J*=7.6 Hz, 3 H), 7.17 (t, *J*=7.3 Hz, 1 H), 6.79 (d, *J*=7.3 Hz, 1 H), 6.64 (t, *J*=7.7 Hz, 1 H), 4.84 (d, *J*=5.8 Hz, 2 H), 3.91 (s, 3 H), 2.30 ppm (s, 3 H); ^13^C NMR (126 MHz, [D_6_]DMSO): *δ*=160.7, 154.2, 153.5, 153.5, 141.4, 140.0, 138.5, 130.9, 128.4, 126.9, 126.7, 124.8, 123.6, 123.2, 118.5, 114.1, 112.9, 112.7, 112.6, 56.1, 44.5, 16.4 ppm; HRMS-ESI: *m*/*z* [*M*+H]^+^ calcd for C_24_H_23_N_6_O: 411.1933, found: 411.1956; HPLC purity: 99.0%.

**2-(2-Amino-5-fluoro-1*H*-benzo[*d*]imidazol-1-yl)-*N*-benzyl-8-methoxyquinazolin-4-amine and 2-(2-Amino-6-fluoro-1*H*-benzo[*d*]imidazol-1-yl)-*N*-benzyl-8-methoxyquinazolin-4-amine (∼1.1:1) (30)**: *N*-Benzyl-2-chloro-8-methoxyquinazolin-4-amine and 5-fluoro-1*H*-benzo[*d*]imidazol-2-amine were reacted according to general procedure A to give **30**, a white solid, as a mixture of regioisomers (14.1 mg, 68%): ^1^H NMR (500 MHz, [D_6_]DMSO): *δ*=9.44–9.31 (m, 2 H), 8.34 (s, 2 H), 8.18 (s, 2 H), 8.13–8.04 (m, 2 H), 7.93 (d, *J*=7.6 Hz, 2 H), 7.51–7.40 (m, 6 H), 7.40–7.30 (m, 6 H), 7.25 (dt, *J*=5.4, 10.8 Hz, 2 H), 7.10 (dd, *J*=5.1, 8.5 Hz, 1 H), 6.96–6.83 (m, 2 H), 6.64–6.55 (m, 1 H), 4.89 (t, *J*=6.3 Hz, 4 H), 3.98 (s, 3 H), 3.97 ppm (s, 3 H); ^13^C NMR (126 MHz, [D_6_]DMSO): *δ*=160.72, 160.70, 159.9, 158.0, 157.2, 156.0, 155.4, 155.2, 153.53, 153.47, 153.13, 153.08, 144.1, 144.0, 139.90, 139.04, 138.38, 138.17, 131.5, 131.4, 128.51, 128.49, 128.0, 127.0, 126.9, 126.6, 125.1, 124.9, 115.1, 115.0, 114.44, 114.37, 114.1, 113.02, 113.00, 112.8, 112.7, 109.2, 109.0, 104.9, 104.7, 102.7, 102.4, 101.2, 101.0, 56.08, 56.06, 44.6, 44.5 ppm; HRMS-ESI: *m*/*z* [*M*+H]^+^ calcd for C_23_H_20_FN_6_O: 415.1683, found: 415.1689; HPLC purity: 98.8%.

**2-(2*H*-Benzo[*b*][1,4]oxazin-4(3*H*)-yl)-*N*-benzyl-5,6-dimethylpyrimidin-4-amine (31)**: *N*-Benzyl-2-chloro-5,6-dimethylpyrimidin-4-amine and 3,4-dihydro-2*H*-benzo[*b*][1,4]oxazine were reacted according to general procedure A to give **31** as a brown oil (6.5 mg, 46%): ^1^H NMR (400 MHz, CDCl_3_): *δ*=8.13–7.99 (m, 1 H), 7.42–7.30 (m, 5 H), 6.94–6.87 (m, 2 H), 6.81–6.74 (m, 1 H), 4.82 (s, 1 H), 4.69 (d, *J*=5.6 Hz, 2 H), 4.31–4.26 (m, 2 H), 4.29–4.23 (m, 2 H), 2.35 (s, 3 H), 2.01 ppm (s, 3 H); ^13^C NMR (101 MHz, CDCl_3_): *δ*=161.7, 160.9, 157.2, 145.9, 139.5, 128.6, 128.4, 127.5, 127.2, 124.0, 122.7, 119.5, 116.6, 101.8, 65.9, 45.3, 42.2, 22.2, 10.9 ppm; HRMS-ESI: *m*/*z* [*M*+H]^+^ calcd for C_21_H_23_N_4_O: 347.1872, found: 347.1872; HPLC purity: 100%.

**2-(2*H*-Benzo[*b*][1,4]oxazin-4(3*H*)-yl)-*N*-benzylpyrimidin-4-amine (32)**: *N*-Benzyl-2-chloropyrimidin-4-amine and 3,4-dihydro-2*H*-benzo[*b*][1,4]oxazine were reacted according to general procedure A to give **32** as a brown oil (20.0 mg, 69%): ^1^H NMR (400 MHz, CDCl_3_): *δ*=8.08–7.96 (m, 2 H), 7.45–7.30 (m, 5 H), 7.01–6.89 (m, 2 H), 6.89–6.78 (m, 1 H), 5.91 (d, *J*=5.8 Hz, 1 H), 5.13 (s, 1 H), 4.57 (d, *J*=5.0 Hz, 2 H), 4.35–4.27 (m, 2 H), 4.27–4.17 ppm (m, 2 H); ^13^C NMR (101 MHz, CDCl_3_): *δ*=162.6, 159.7, 156.2, 146.3, 138.5, 128.7, 127.8, 127.5, 127.4, 124.4, 123.6, 119.6, 116.8, 66.0, 45.3, 42.1 ppm; HRMS-ESI: *m*/*z* [*M*+H]^+^ calcd for C_19_H_19_N_4_O: 319.1559, found: 319.1555; HPLC purity: 100%.

**2-((2-(2*H*-Benzo[*b*][1,4]oxazin-4(3*H*)-yl)-4-(benzylamino)quinazolin-8-yl)oxy)ethanol (33)**: 2-(2*H*-Benzo[*b*][1,4]oxazin-4(3*H*)-yl)-4-(benzylamino)quinazolin-8-ol and 2-chloroethanol were reacted according to general procedure B to give **33** as a brown oil (3.7 mg, 33%): ^1^H NMR (400 MHz, CDCl_3_): *δ*=8.05 (d, *J*=7.3 Hz, 1 H), 7.39 (dd, *J*=5.9, 7.5 Hz, 6 H), 7.20 (d, *J*=6.7 Hz, 1 H), 7.12 (t, *J*=7.9 Hz, 1 H), 6.99 (d, *J*=6.9 Hz, 1 H), 6.97–6.91 (m, 1 H), 6.84 (t, *J*=7.6 Hz, 1 H), 4.84 (d, *J*=5.5 Hz, 2 H), 4.34 (s, 4 H), 4.29–4.18 (m, 2 H), 3.92–3.81 ppm (m, 2 H); ^13^C NMR (101 MHz, CDCl_3_): *δ*=159.8, 146.5, 138.2, 128.8, 127.8, 127.6, 127.4, 124.4, 124.1, 122.4, 119.5, 118.6, 117.0, 114.9, 112.2, 73.6, 66.1, 60.8, 45.4, 42.5 ppm; HRMS-ESI: *m*/*z* [*M*+H]^+^ calcd for C_25_H_25_N_4_O_3_: 429.1927, found: 429.1925; HPLC purity: 100%.

**2-(2*H*-Benzo[*b*][1,4]oxazin-4(3*H*)-yl)-*N*-benzyl-8-(2-methoxyethoxy)quinazolin-4-amine (34)**: 2-(2*H*-Benzo[*b*][1,4]oxazin-4(3*H*)-yl)-4-(benzylamino)quinazolin-8-ol and 1-chloro-2-methoxyethane were reacted according to general procedure B to give **34** as a brown oil (3.6 mg, 27%): ^1^H NMR (400 MHz, CDCl_3_): *δ*=8.28 (d, *J*=7.7 Hz, 1 H), 7.40–7.39 (m, 4 H), 7.37–7.32 (m, 1 H), 7.21–719 (m, 1 H), 7.13–7.09 (m, 2 H), 6.99–6.88 (m, 2 H), 6.83–6.79 (m, 1 H), 5.86 (br s, 1 H), 4.83 (d, *J*=5.4 Hz, 2 H), 4.45–4.38 (m, 2 H), 4.38–4.27 (m, 4 H), 3.97–3.88 (m, 2 H), 3.56 ppm (s, 3 H); ^13^C NMR (126 MHz, CDCl_3_): *δ*=159.7, 156.0, 153.2, 146.2, 143.9, 138.4, 128.8, 128.1, 127.7, 127.6, 124.5, 123.2, 121.9, 119.4, 116.6, 114.0, 113.0, 111.9, 71.1, 68.8, 66.1, 59.5, 45.4, 42.1, 29.7 ppm; HRMS-ESI: *m*/*z* [*M*+H]^+^ calcd for C_26_H_27_N_4_O_3_: 443.2083, found: 443.2088; HPLC purity: 98.0%.

**2-(2*H*-Benzo[*b*][1,4]oxazin-4(3*H*)-yl)-*N*-benzyl-8-(2-(diethylamino)ethoxy)quinazolin-4-amine (35)**: 2-(2*H*-Benzo[*b*][1,4]oxazin-4(3*H*)-yl)-4-(benzylamino)quinazolin-8-ol and 2-bromo-*N*,*N*-diethylethanamine hydrochloride were reacted according to general procedure B to give **35** as a brown oil (4.1 mg, 64%): ^1^H NMR (400 MHz, CDCl_3_): *δ*=7.98–7.79 (m, 1 H), 7.31–7.22 (m, 6 H), 7.05 (s, 2 H), 6.85 (s, 2 H), 6.70 (s, 1 H), 4.75 (d, *J*=5.5, 2 H), 4.58 (s, 2 H), 4.21 (s, 4 H), 3.48 (s, 2 H), 3.21 (d, *J*=7.3, 4 H), 1.32 ppm (t, *J*=7.3, 6 H); HRMS-ESI: *m*/*z* [*M*+H]^+^ calcd for C_29_H_34_N_5_O_2_: 484.2713 found: 484.2712; HPLC purity: 68.2%.

**2-(2*H*-Benzo[*b*][1,4]oxazin-4(3*H*)-yl)-*N*-benzyl-8-(4-methoxyphenyl)quinazolin-4-amine (36)**: A mixture of 2-(2*H*-benzo[*b*][1,4]oxazin-4(3*H*)-yl)-*N*-benzyl-8-bromoquinazolin-4-amine (12.0 mg, 26.8 mmol), palladium acetate (0.6 mg, 2.7 mmol), [1,1′-biphenyl]-2-yldicyclohexylphosphine (1.9 mg, 5.4 mmol), 4-methoxyphenylboronic acid (6.1 mg, 40.2 mmol) and potassium fluoride (4.7 mg, 80.5 mmol) in THF (0.5 mL) was heated at 50 °C for 16 h. The mixture was diluted with EtOAc and washed with H_2_O. The organic phase was concentrated and purified by silica gel chromatography to give **36** as a brown solid (12 mg, 94%): *R*_f_=0.5 (CH_2_Cl_2_/CH_3_OH, 1:9); ^1^H NMR (400 MHz, CDCl_3_): *δ*=8.17–8.09 (m, 1 H), 7.75–7.69 (m, 2 H), 7.68 (dd, *J*=1.4, 7.3 Hz, 1 H), 7.56 (dd, *J*=1.3, 8.2 Hz, 1 H), 7.40 (dd, *J*=1.9, 3.5 Hz, 4 H), 7.39–7.32 (m, 1 H), 7.28–7.21 (m, 1 H), 7.07–7.00 (m, 2 H), 6.92 (dtd, *J*=1.6, 8.1, 9.9 Hz, 2 H), 6.77–6.70 (m, 1 H), 5.94 (s, 1 H), 4.85 (t, *J*=5.3 Hz, 2 H), 4.33–4.25 (m, 4 H), 3.92 ppm (s, 3 H); ^13^C NMR (101 MHz, CDCl_3_): *δ*=160.1, 158.8, 156.1, 149.5, 146.2, 138.6, 137.6, 133.1, 131.9, 131.6, 128.8, 128.2, 127.7, 127.6, 125.1, 123.2, 121.9, 119.5, 119.4, 116.5, 113.2, 111.6, 66.1, 55.4, 45.5, 42.1 ppm; 89%): HRMS-ESI: *m*/*z* [*M*+H]^+^ calcd for C_30_H_27_N_4_O_2_: 475.2134, found: 475.2135; HPLC purity: 92.9%.

**2-(2*H*-Benzo[*b*][1,4]oxazin-4(3*H*)-yl)-*N*-benzyl-8-butoxyquinazolin-4-amine (37)**: *N*-Benzyl-8-butoxy-2-chloroquinazolin-4-amine and 3,4-dihydro-2*H*-benzo[*b*][1,4]oxazine were reacted according to general procedure A to give **37** as a brown oil (4.9 mg, 61%): ^1^H NMR (400 MHz, CDCl_3_): *δ*=8.37 (d, *J*=8.2 Hz, 1 H), 7.47–7.31 (m, 5 H), 7.20–7.07 (m, 2 H), 7.04 (dd, *J*=1.5, 7.5 Hz, 1 H), 7.00–6.89 (m, 2 H), 6.89–6.77 (m, 1 H), 5.85 (s, 1 H), 4.84 (d, *J*=5.4 Hz, 2 H), 4.48–4.38 (m, 2 H), 4.35–4.32 (m, 2 H), 4.15 (t, *J*=6.5 Hz, 2 H), 2.04–1.89 (m, 2 H), 1.72–1.62 (m, 2 H), 1.08 ppm (t, *J*=7.4 Hz, 3 H); ^13^C NMR (101 MHz, CDCl_3_): *δ*=159.7, 155.9, 153.6, 146.2, 143.7, 138.5, 128.8, 128.3, 127.7, 127.6, 124.4, 123.1, 121.9, 119.5, 116.6, 112.7, 112.1, 111.8, 68.6, 66.1, 45.4, 42.1, 31.4, 19.4, 14.0 ppm; HRMS-ESI: *m*/*z* [*M*+H]^+^ calcd for C_27_H_29_N_4_O_2_: 441.2291, found: 441.2296; HPLC purity: 100%.

**2-(2*H*-Benzo[*b*][1,4]oxazin-4(3*H*)-yl)-*N*-benzyl-8-methoxyquinazolin-4-amine (38)**: *N*-Benzyl-2-chloro-8-methoxyquinazolin-4-amine and 3,4-dihydro-2*H*-benzo[*b*][1,4]oxazine were reacted according to general procedure A to give **38** as a brown oil (7.1 mg, 53%): ^1^H NMR (400 MHz, CDCl_3_): *δ*=8.21–8.08 (m, 1 H), 7.46–7.31 (m, 5 H), 7.18 7.11 (m, 2 H), 7.05 (dd, *J*=1.6, 7.3 Hz, 1 H), 6.99–6.88 (m, 2 H), 6.80 (ddd, *J*=2.3, 6.5, 8.7 Hz, 1 H), 5.86 (s, 1 H), 4.83 (d, *J*=5.5 Hz, 2 H), 4.50–4.40 (m, 2 H), 4.40–4.29 (m, 2 H), 4.03 ppm (s, 3 H); ^13^C NMR (101 MHz, CDCl_3_): *δ*=159.7, 156.2, 153.9, 146.2, 143.6, 138.5, 128.8, 128.1, 127.8, 127.6, 124.3, 123.2, 121.9, 119.4, 116.7, 112.3, 111.8, 111.4, 66.2, 56.1, 45.4, 42.2 ppm; HRMS-ESI: *m*/*z* [*M*+H]^+^ calcd for C_24_H_23_N_4_O_2_: 399.1821, found: 399.1824; HPLC purity: 100%.

**2-(2*H*-Benzo[*b*][1,4]oxazin-4(3*H*)-yl)-*N*-(4-fluorobenzyl)-8-methoxyquinazolin-4-amine (40)**: 2-Chloro-*N*-(4-fluorobenzyl)-8-methoxyquinazolin-4-amine and 3,4-dihydro-2*H*-benzo[*b*][1,4]oxazine were reacted according to general procedure A to give **40** as a brown oil (2.9 mg, 22%): ^1^H NMR (400 MHz, CDCl_3_): *δ*=8.12–7.87 (m, 1 H), 7.25 (br s, 2 H), 7.14–7.02 (m, 2 H), 6.98–6.93 (m, 2 H), 6.84 (br s, 2 H), 6.78–6.56 (m, 1 H), 5.89–5.66 (m, 1 H), 4.69 (br s, 2 H), 4.32 (br s, 2 H), 4.24 (br s, 2 H), 3.92 ppm (s, 3 H); ^13^C NMR (126 MHz, CDCl_3_) *δ*=163.2, 161.2, 159.7, 134.2, 129.5, 124.4, 123.3, 122.0, 119.4, 116.7, 115.6, 115.5, 112.3, 111.7, 111.4, 66.2, 56.2, 44.6, 42.2, 29.7 ppm; HRMS-ESI: *m*/*z* [*M*+H]^+^ calcd for C_24_H_22_FN_4_O_2_: 417.1727, found: 417.1726; HPLC purity: 98.8%.

**2-(2*H*-Benzo[*b*][1,4]oxazin-4(3*H*)-yl)-8-methoxy-*N*-(thiophen-2-ylmethyl)quinazolin-4-amine (41)**: 2-Chloro-8-methoxy-*N*-(thiophen-2-ylmethyl)quinazolin-4-amine and 3,4-dihydro-2*H*-benzo[*b*][1,4]oxazine were reacted according to general procedure A to give **41** as a brown oil (15.9 mg, 80%): ^1^H NMR (400 MHz, CDCl_3_): *δ*=8.25 (dd, *J*=1.4, 8.2 Hz, 1 H), 7.27 (dd, *J*=1.3, 5.1 Hz, 1 H), 7.19–7.09 (m, 2 H), 7.08–7.02 (m, 2 H), 6.99 (ddd, *J*=2.5, 4.1, 8.1 Hz, 1 H), 6.95 (dd, *J*=1.8, 4.0 Hz, 1 H), 6.93–6.86 (m, 1 H), 5.90 (s, 1 H), 4.98 (d, *J*=5.1 Hz, 2 H), 4.47 (dd, *J*=3.6, 5.3 Hz, 2 H), 4.37 (dd, *J*=3.6, 5.3 Hz, 2 H), 4.02 ppm (s, 3 H); ^13^C NMR (101 MHz, CDCl_3_): *δ*=159.3, 156.1, 153.9, 146.2, 143.6, 141.2, 128.1, 126.9, 126.2, 125.4, 124.4, 123.3, 122.0, 119.5, 116.7, 112.4, 111.7, 111.5, 66.2, 56.1, 42.3, 40.2 ppm; HRMS-ESI: *m*/*z* [*M*+H]^+^ calcd for C_22_H_21_N_4_O_2_S: 405.1385, found: 405.1383; HPLC purity: 96.6%.

**2-(2*H*-Benzo[*b*][1,4]oxazin-4(3*H*)-yl)-*N*-(cyclohexylmethyl)-8-methoxyquinazolin-4-amine (42)**: 2-Chloro-*N*-(cyclohexylmethyl)-8-methoxyquinazolin-4-amine and 3,4-dihydro-2*H*-benzo[*b*][1,4]oxazine were reacted according to general procedure A to give **42** as a brown oil (13.6 mg, 69%): ^1^H NMR (400 MHz, CDCl_3_): *δ*=8.26 (dd, *J*=1.4, 8.1 Hz, 1 H), 7.20–7.09 (m, 2 H), 7.03 (dd, *J*=1.9, 7.1 Hz, 1 H), 7.01–6.86 (m, 3 H), 5.69 (s, 1 H), 4.46 (dd, *J*=3.6, 5.2 Hz, 2 H), 4.37 (dd, *J*=3.6, 5.2 Hz, 2 H), 4.02 (s, 3 H), 3.54–3.42 (m, 2 H), 187–1.71 (m, 6 H), 1.40–1.13 (m, 4 H), 1.04 ppm (q, *J*=12.0 Hz, 2 H); ^13^C NMR (101 MHz, CDCl_3_): *δ*=160.0, 156.4, 153.9, 146.2, 143.4, 128.2, 124.4, 123.1, 121.7, 119.3, 116.7, 112.2, 111.9, 111.2, 66.2, 56.1, 47.6, 42.2, 37.8, 31.1, 26.5, 25.9 ppm; HRMS-ESI: *m*/*z* [*M*+H]^+^ calcd for C_24_H_29_N_4_O_2_: 405.2291, found: 405.2289; HPLC purity: 95.0%.

**2-(((2-(2*H*-Benzo[*b*][1,4]oxazin-4(3*H*)-yl)-8-methoxyquinazolin-4-yl)amino)methyl)phenol (43)**: 2-(((2-Chloro-8-methoxyquinazolin-4-yl)amino)methyl)phenol and 3,4-dihydro-2*H*-benzo[*b*][1,4]oxazine were reacted according to general procedure A to give **43** as a brown solid (11.0 mg, 51%): ^1^H NMR (400 MHz, CDCl3): *δ*=8.91 (br s, 1 H), 7.97 (br s, 1 H), 7.14–7.04 (m, 3 H), 7.01 (t, *J*=7.9 Hz, 1 H), 6.93 (d, *J*=8.8 Hz, 1 H), 6.91–6.85 (m, 2 H), 6.82 (t, *J*=7.3 Hz, 1 H), 6.73 (t, *J*=7.2 Hz, 2 H), 4.68 (br s, 2 H), 4.30 (s, 4 H), 3.87 ppm (s, 3 H); ^13^C NMR (101 MHz, CDCl_3_): *δ*=159.9, 155.8, 146.3, 130.8, 129.9, 124.0, 122.8, 119.9, 119.7, 117.4, 117.2, 112.6, 112.1, 111.7, 66.0, 56.1, 43.1, 42.0 ppm; HRMS-ESI: *m*/*z* [*M*+H]^+^ calcd for C_24_H_23_N_4_O_3_: 415.1770, found: 415.1763; HPLC purity: 100%.

## Notes

As a service to our authors and readers, this journal provides supporting information supplied by the authors. Such materials are peer reviewed and may be re-organized for online delivery, but are not copy-edited or typeset. Technical support issues arising from supporting information (other than missing files) should be addressed to the authors.
