# Supplementary material for: Structure–Activity Relationship Study Reveals ML240 and ML241 as Potent and Selective Inhibitors of p97 ATPase
Source: ChemMedChem. 2013 Jan 11;8(2):297–312. doi: 10.1002/cmdc.201200520 (PMC3662613; doi:10.1002/cmdc.201200520)

CHEMMEDCHEM
Chemistry & Drug Discovery

Supporting Information

© Copyright Wiley-VCH Verlag GmbH & Co. KGaA, 69451 Weinheim, 2013

Structure–Activity Relationship Study Reveals ML240 and ML241 as Potent and Selective Inhibitors of p97 ATPase

Tsui-Fen Chou,*[a, c] Kelin Li,[b] Kevin J. Frankowski,[b] Frank J. Schoenen,[b] and Raymond J. Deshaies*[a]

cmdc_201200520_sm_miscellaneous_information.pdf
cmdc_201200520_sm_supporting_information.xls

Contents

Figure S1: ODD-Luc degradation western blot assays for select compounds S-3

Figure S2: Results from the NCI 60 cell line tumor growth screen for **ML240** S-4

Figure S3: Results from the NCI 60 cell line tumor growth screen for **ML241** S-5

Table S1: Commercial analogues of *N*-benzyl-2-(2-fluorophenyl)quinazolin-4-amine S-6

Table S2: Analogues of N^2, N^4-disubstitued quinazoline-2,4-diamine S-7

Table S3: Analogues of N^2, N^4-diphenylquinazoline-2,4-diamine S-9

Table S4: Analogues of N^4-benzyl-N^2-phenylquinazoline-2,4-diamine S-10

Table S5: Analogues of N^4-benzyl-N^2-(3-chlorophenyl)quinazoline-2,4-diamine S-11

Table S6: Analogues of N^2,N^4-dibenzylquinazoline-2,4-diamine (DBeQ) S-12

Table S7: Analogues of N^2,N^4-dibenzylquinazoline-2,4-diamine (DBeQ) S-13

Table S8: N^2 Constrained DBeQ analogues S-14

Table S9: Alternatives to the quinazoline scaffold analogues S-16

Table S10: Miscellaneous 8-methoxyquinazoline analogues S-17

Table S11: 3,4-Dihydro-1,4-benzoxazine analogues incorporating the quinazoline of **ML241** S-18

Table S12: Unabridged compound purity assessment via HPLC analysis S-20

Table S13: Aqueous solubility for select compounds S-25

Table S14: Kinase/ATPase profiling for **ML240** and **ML241** S-28

General experimental details S-37

General Procedure A: Representative protocol for the synthesis of quinazoline analogues, synthesis and characterization for **16**, S45-S56, S70-S125, S142-S147, S156-S160, **ML241**, S162, S165 and S169-S188) S-38

General Procedure B: Representative protocol for the synthesis of 8-*O*-alkylatedquinazoline analogues, synthesis and characterization for S148 (**9**), S150-S152 and S154 .... S-142

General Prococedure C: representative protocol for the one-pot synthesis of quinazoline analogues, synthesis and characterization for S129, S126-S128 and S130-S141 .... S-148

General procedure D: representative protocol for the synthesis of quinazoline analogues of less nucleophilic amines, synthesis and characterization for S166, S161, S163 (**31**), S164, S167 (**19**) and S168 .... S-165

General procedure E: representative protocol for the synthesis of quinazoline analogues via a Suzuki coupling route, synthesis and characterization for S153 (**36**) and S149 .... S-172

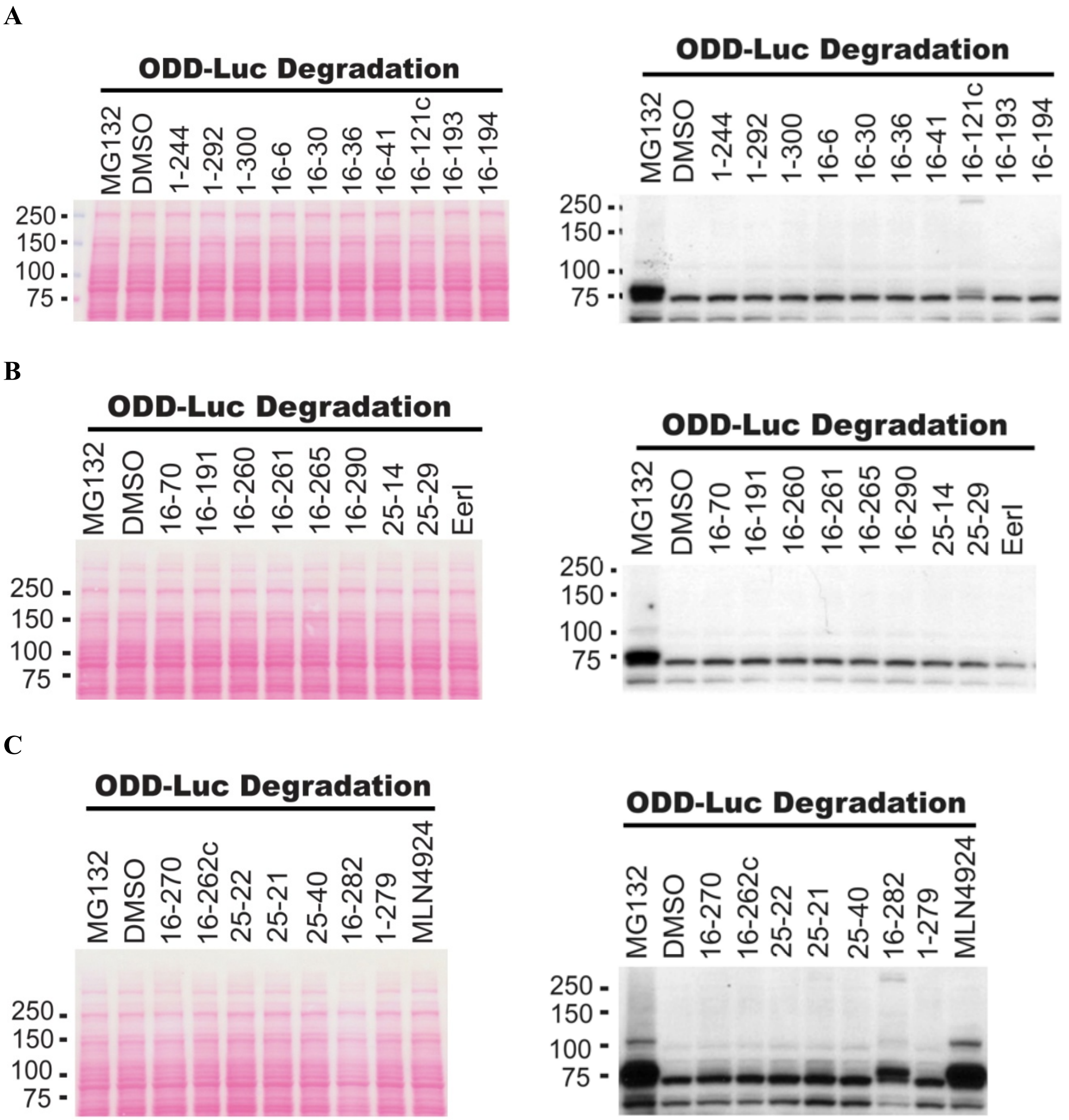

Figure S1. ODD-Luc degradation western blot assay. HeLa cells stably expressing the Ub^{G76V}-GFP and ODD-Luc reporters were grown in 6 well plates. Cells were treated with MG132 (4 **µ**M for 1 h) to accumulate reporter, washed, and exposed to the indicated test compounds plus CHX to initiate a chase. After 2h, cells were harvested and evaluated by immunoblotting for the remaining ODD-Luc (right panel). The Ponceau S-stained filters serve as loading controls (left panel). In the figure test compounds are identified using the “KU-SCC #”, where each two part identifier is prefixed with the letter code KSC. Compound structures can be found in the supplementary SAR Tables S1 to S11.

KUC107871N p97 probes

Developmental Therapeutics Program	NSC: D-759704 / 1	Conc: 1.00E-5 Molar	Test Date: Jun 20, 2011
One Dose Mean Graph	Experiment ID: 1106OS69		Report Date: Aug 02, 2011

Panel/Cell Line	Growth Percent
Leukemia	
HL-60(TB)	-73.88
MOLT-4	-68.71
Non-Small Cell Lung Cancer	
A549/ATCC	-87.93
EKVX	-82.83
HOP-62	-90.94
NCI-H226	-74.40
NCI-H23	-86.55
NCI-H322M	-96.90
NCI-H460	-71.80
NCI-H522	-74.45
Colon Cancer	
COLO 205	-95.26
HCC-2998	-93.99
HCT-116	-80.37
HCT-15	-82.60
HT29	-83.50
KM12	-94.70
SW-620	-67.40
CNS Cancer	
SF-268	-87.67
SF-295	-75.31
SF-539	-85.07
SNB-19	-79.35
SNB-75	-96.84
U251	-92.31
Melanoma	
LOX IMVI	-87.91
MALME-3M	-80.69
M14	-80.65
MDA-MB-435	-79.48
SK-MEL-2	-79.12
SK-MEL-28	-91.76
SK-MEL-5	-99.44
UACC-257	-93.81
UACC-62	-91.32
Ovarian Cancer	
IGROV1	-71.91
OVCAR-3	-99.70
OVCAR-4	-100.00
OVCAR-5	-97.46
OVCAR-8	-69.98
NCI/ADR-RES	-68.34
SK-OV-3	-99.41
Renal Cancer	
786-0	-89.35
A498	-95.45
ACHN	-98.20
CAKI-1	-88.11
SN12C	-72.64
TK-10	-95.73
UO-31	-89.93
Prostate Cancer	
PC-3	-82.99
DU-145	-98.93
Breast Cancer	
MCF7	-69.60
MDA-MB-231/ATCC	-87.34
HS 578T	-43.78
BT-549	-80.55
T-47D	-68.27
MDA-MB-468	-90.44
Mean	-84.54
Delta	15.46
Range	56.22

Mean Growth Percent - Growth Percent

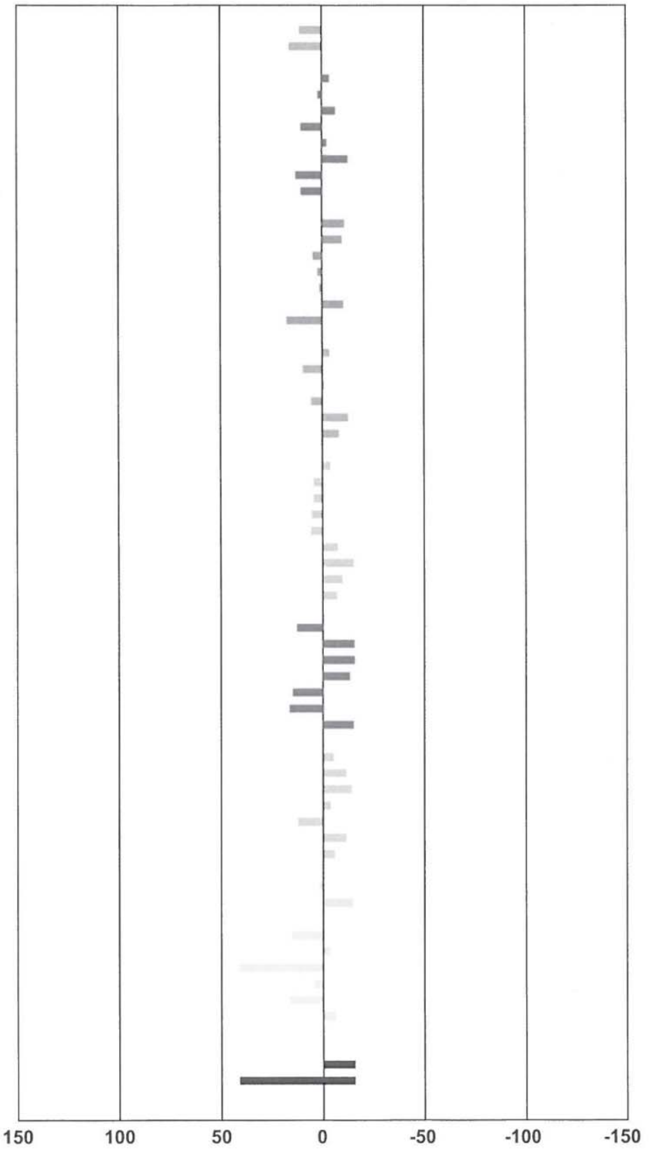

Figure S2: Full results from the NCI 60 cell line panel for **ML240**.

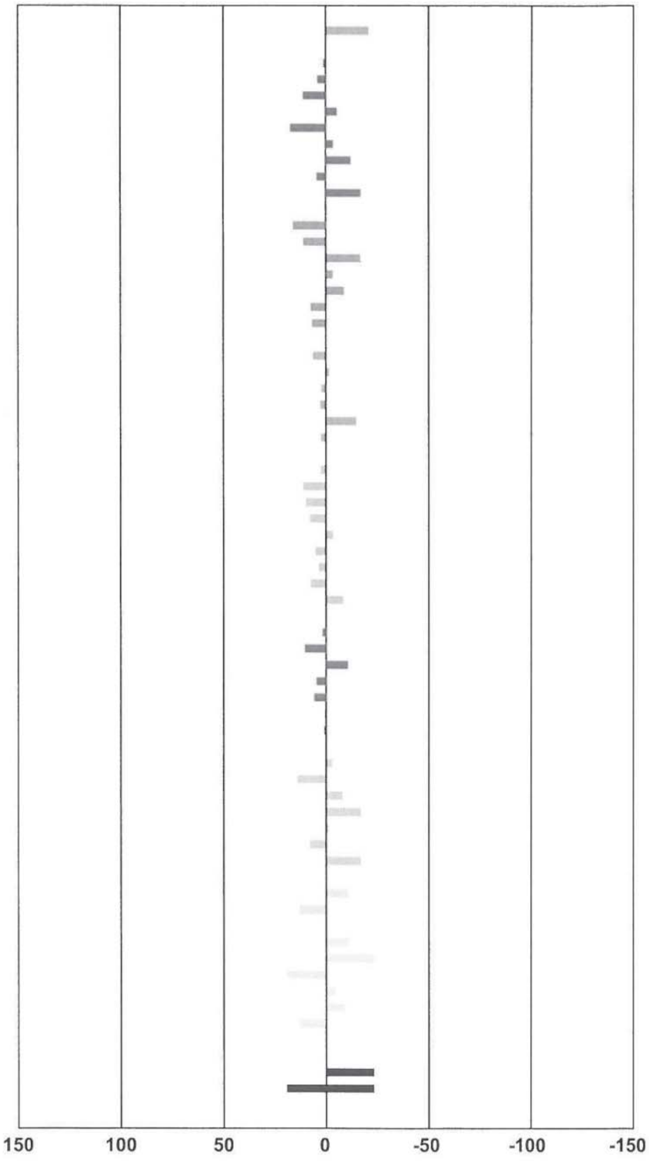

KUC 10 7881N P97 Probe

Developmental Therapeutics Program | NSC: D-759705 / 1 | Conc: 1.00E-5 Molar | Test Date: Jun 20, 2011

One Dose Mean Graph | Experiment ID: 1106OS69 | Report Date: Aug 02, 2011

Panel/Cell Line	Growth Percent
Leukemia	
MOLT-4	72.28
Non-Small Cell Lung Cancer	
A549/ATCC	93.61
EKVX	96.60
HOP-62	103.52
HOP-92	87.72
NCI-H226	109.83
NCI-H23	89.57
NCI-H322M	81.12
NCI-H460	96.94
NCI-H522	76.11
Colon Cancer	
COLO 205	108.58
HCC-2998	103.50
HCT-116	76.43
HCT-15	89.72
HT29	84.31
KM12	99.90
SW-620	99.21
CNS Cancer	
SF-268	98.76
SF-295	91.41
SF-539	94.66
SNB-19	95.15
SNB-75	78.43
U251	94.79
Melanoma	
LOX IMVI	95.09
MALME-3M	103.62
M14	102.23
MDA-MB-435	100.26
SK-MEL-2	89.82
SK-MEL-28	97.51
SK-MEL-5	95.85
UACC-257	100.04
UACC-62	84.89
Ovarian Cancer	
IGROV1	94.35
OVCAR-3	102.92
OVCAR-4	82.55
OVCAR-5	97.23
OVCAR-8	98.44
NCI/ADR-RES	93.13
SK-OV-3	93.52
Renal Cancer	
786-0	90.44
A498	106.48
ACHN	85.43
CAKI-1	76.28
SN12C	91.92
TK-10	100.29
UO-31	76.50
Prostate Cancer	
PC-3	82.68
DU-145	105.61
Breast Cancer	
MCF7	82.52
MDA-MB-231/ATCC	69.94
HS 578T	111.85
BT-549	88.69
T-47D	84.36
MDA-MB-468	105.49
Mean	92.82
Delta	22.88
Range	41.91

Figure S3: Full results from the NCI 60 cell line panel for **ML241**.

Table S1: Commercial analogues of *N*-benzyl-2-(2-fluorophenyl)quinazolin-4-amine

**compound identifiers**					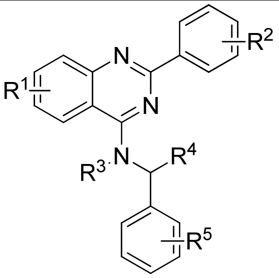					**p97 inhibition**	
**Cpd**	**CID**	**SID**	**KU SCC #**	*****	**R1**	**R2**	**R3**	**R4**	**R5**	**ATPase IC_{50} (μM)**	**Ub^{G76V}-GFP IC_{50} (μM)**
S1 **1**[#]	886813	87796231	KSC-1-150	P	H	2-F	H	H	H	3±0.8	9.0±1.3
S2 **2**[#]	1894007	87796228	KSC-1-146	P	H	2-Cl	H	H	H	1.2±0.6	8±3
S3 **3**[#]	2927831	87796230	KSC-1-149	P	H	3-NO_2	H	H	H	5.9±3	4.0±1.6
S4	780643	87796227	KSC-1-145	P	H	H	H	H	H	4.2±2.3	12±3
S5	4084712	87796265	KSC-1-226	P	H	3-Me	H	H	H	4.2±0.2	17±4
S6	1591117	87796273	KSC-1-236	P	H	4-Cl	H	H	H	5.5±1.9	20±3
S7	1187251	87796271	KSC-1-234	P	H	4-NO_2	H	H	H	11±6	19±7
S8	949742	87796266	KSC-1-227	P	H	4-OMe	H	H	H	1.6±0.3	15±2
S9	3395671	87796263	KSC-1-224	P	H	4-Me	H	H	H	5.7±0.7	13±2
S10	2452802	87796251	KSC-1-212	P	H	H	H	H	4-F	11±3	19±3
S11	20963125	87796258	KSC-1-219	P	H	4-OMe	H	H	4-F	7.8±0.4	9.8±3
S12	20963158	87796259	KSC-1-220	P	H	4-OMe	H	H	4-OMe	13±4	15±5
S13	20963177	87796260	KSC-1-221	P	H	4-OMe	H	H	4-Me	8±2	12±2
S14	20963187	87796261	KSC-1-222	P	H	4-OMe	H	H	4-Cl	8±2	9.3±1
S15	20963255	87796262	KSC-1-223	P	H	4-Cl	H	H	4-F	8±0.2	20±5
S16	4084711	87796264	KSC-1-225	P	H	3-Me	H	H	4-F	15±2	12±0.8
S17	2790952	87796274	KSC-1-237	P	H	4-Me	H	Me	H	7±4	17±4
S18	15992808	87796255	KSC-1-216	P	H	2-F	H	Me	H	>30	15±4
S19 **4**[#]	8074678	87796236	KSC-1-193	P	7-Cl	H	H	H	H	70±24	12±4
S20 **5**[#]	8080035	87796237	KSC-1-194	P	7-Cl	4-Me	H	H	H	39±13	24±5
S21	8080040	87796238	KSC-1-195	P	7-Cl	4-OMe	H	H	H	25±11	70±30

S22	2454628	87796252	KSC-1-213	P	H	H	CH2	H	2-CH2	>30	19±5
S23	2047240	87796235	KSC-1-147	P	H	4-Br	H	H	H	72±14	25±10
S24	2178012	87796229	KSC-1-148	P	H	2-NO_2	Me	H	H	25±11	11±6
S25	1819025	87796240	KSC-1-200	P	H	3,4-Di-Cl	H	H	H	7.1±0.4	37±6
S26	2452792	87796250	KSC-1-211	P	H	H	H	H	3-Cl	28±9	17±4
S27	2465033	87796253	KSC-1-214	P	H	H	H	H	2-Cl	30±10	11±3
S28	2456309	87796254	KSC-1-215	P	H	H	H	H	4-Cl	>30	15±3
S29	15993188	87796256	KSC-1-217	P	H	2-F	H	H	2-Cl	26±12	15±3
S30	15995431	87796257	KSC-1-218	P	H	2-F	Me	H	H	25±4	6.0±2.0
S31	1566144	87796272	KSC-1-235	P	H	4-NO_2	Me	H	H	8.4±4	28±4
S32	5051334	87796275	KSC-1-238	P	H	H	H	Me	H	27±14	13±1
S33	1895905	87796288	KSC-1-257	P	H	2,4-Di-Cl	H	H	H	>30	11±0.8
S34 **8**[#]	1553819	87796289	KSC-1-258	P	H	H	Me	H	H	49±17	17±5

* Designates whether a compound was purchased (P) or synthesized (S)
[#] Compound identifier used in the main text.

Table S2: Analogues of N^2, N^4-disubstitued quinazoline-2,4-diamine

**compound identifiers**					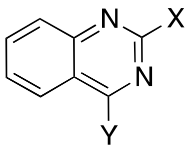	**p97 inhibition**	
**Cpd**	**CID**	**SID**	**KU SCC #**	*****	**structure**	**ATPase IC_{50} (μM)**	**Ub^{G76V}-GFP IC_{50} (μM)**
S35 **6**[#]	696840	87796233	KSC-1-152	P	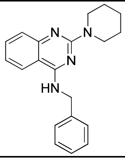	26±4	7.3±2
S36 **7**[#]	832282	87796232	KSC-1-151	P	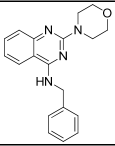	23±6	36±9
S37	2950240	87796276	KSC-1-240	P	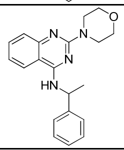	34±10	36±0.3

S38	832283	92093143	KSC-1-260	P	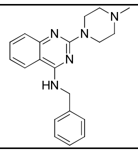	53±18	31±6
S39	1330669	87796248	KSC-1-208	P	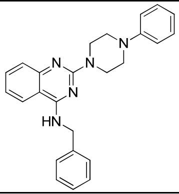	34±11	35±5
S40	1337599	92093137	KSC-1-203	P	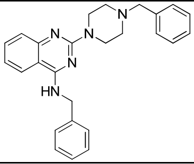	33±8	12±1
S41	1091839	92093140	KSC-1-242	P	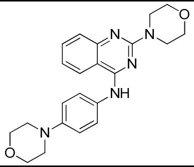	57±15	56±10
S42	2955641	87796277	KSC-1-241	P	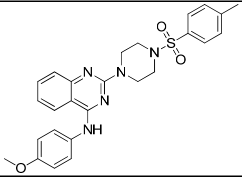	22±12	39±18
S43	5051334	87796275	KSC-1-239	P	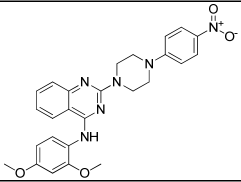	43±13	70±16
S44	2932797	92093142	KSC-1-254	P	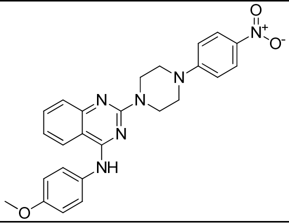	30±6	34±6
S45	46873814	99313590	KSC-16-190	S	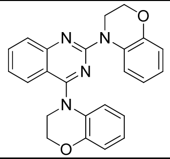	>30	8.1±1.8
S46	49830268	103904170	KSC-16-272	S	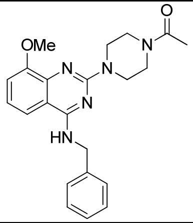	>30	>20

S47	49830259	103904173	KSC-16-277	S	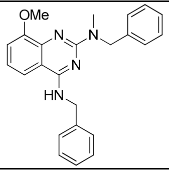	7±3	>20
S48	49852179	104221944	KSC-25-10	S	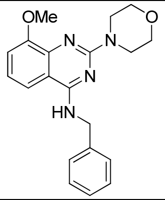	31±7	47±9
S49	49852178	104221946	KSC-25-16	S	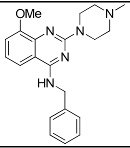	>30	45±18

* Designates whether a compound was purchased (P) or synthesized (S)
# Compound identifier used in the main text.

Table S3: Analogues of N^2, N^4-diphenylquinazoline-2,4-diamine

**compound identifiers**					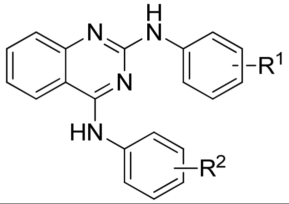		**p97 inhibition**	
**Cpd**	**CID**	**SID**	**KU SCC #**	*****	**R1**	**R2**	**ATPase IC_{50} (μM)**	**Ub^{G76V}-GFP IC_{50} (μM)**
S50	17324423	99239924	KSC-16-149	S	4-OMe	4-OMe	22±4	23±3
S51	46850879	99239936	KSC-16-163	S	3-Cl	H	34±16	12±2
S52	46850870	99239937	KSC-16-164	S	3-Cl	4-OMe	34±14	13±1
S53	46850872	99239940	KSC-16-174	S	3-Cl	4-Me	49±19	30±4
S54	46850878	99239942	KSC-16-176	S	3-Cl	4-Cl	117±5.8	21±2
S55	46850875	99239944	KSC-16-151	S	4-Me	4-Me	54±19	21±4
S56 **9**#	213847	99206554	KSC-16-146	S	H	H	2.6±0.8	7.2±1
S57	1878549	87796278	KSC-1-243	P	4-Cl	4-Cl	52±10	19±4

* Designates whether a compound was purchased (P) or synthesized (S)
# Compound identifier used in the main text.

Table S4 : Analogues of N^4-benzyl-N^2-phenylquinazoline-2,4-diamine

compound identifiers					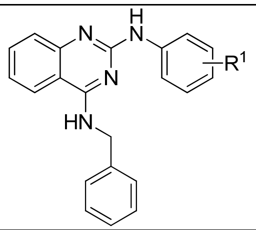	p97 inhibition	
**Cpd**	**CID**	**SID**	**KU SCC #**	*****	**R1**	**ATPase IC_{50} (μM)**	**$Ub^{G76V}GFP$ IC_{50} (μM)**
S58 **10**#	2909934	87796234	KSC-1-153	P	H	2.3±1	3.1±0.4
S59	929548	87796285	KSC-1-251	P	4-Me	2.2±0.4	6.3±1.4
S60	2351737	87796281	KSC-1-246	P	4-Cl	5.4±2	5.9±1.2
S61	797650	87796284	KSC-1-249	P	4-F	3.8±1.2	9.4±1
S62	1943389	87796280	KSC-1-245	P	4-Br	1.8±0.4	6.0±0.8
S63	1330474	92093141	KSC-1-250	P	4-OMe	3.8±0.5	4.5±1.1
S64	949445	87796286	KSC-1-252	P	2-Me	3.4±1.5	10±2
S65	886196	87796282	KSC-1-247	P	2-F	0.85±0.17	10±2
S66	1415819	87796283	KSC-1-248	P	2-OMe	2.2±0.9	11±3
S67	950033	87796287	KSC-1-253	P	3-Me	1.7±0.5	3.0±0.7
S68 **11**#	1633082	87796279	KSC-1-244	P	3-Cl	0.48±0.16	7.8±1.3
S69	1571079	87796290	KSC-1-259	P	3,4-di-Cl	8.1±2.6	12±2
S70	1330474	92252642	KSC-1-290	S	4-OMe	3.5±0.5	4.8±0.7
S71	45108365	92252645	KSC-1-293	S	3-Br	2.5±0.5	4.3±0.8
S72	39861404	92252643	KSC-1-291	S	3-OMe	2.5±0.2	4.3±0.7
S73	46850871	99239933	KSC-16-155	S	3-NO_2	5.2±0.5	19±2
S74 **44**#	11645888	92252644	KSC-1-292	S	3-F	1.6±0.1	2.7±0.4
S75	45108364	92252647	KSC-1-295	S	3-Cl-6-F	13±3	13±2
S76	45108364	92252647	KSC-1-294	S	3,5-di-Cl	8.2±0.3	13±1
S77	46224527	96079523	KSC-16-103	S	3-Cl-2-OMe	0.9±0.2	6.3±1.7
S78	46224522	96079524	KSC-16-104	S	3-F-2-Me	0.9±0.1	3.7±0.8
S79	46224524	96079525	KSC-16-105	S	3-F-5-Me	0.7±0.05	5.4±0.8

S80	46224529	96079526	KSC-16-106	S	3-F-2-OMe	1.7±0.5	12±3
S81	46224523	96079527	KSC-16-107	S	3-Cl-2-Me	0.7±0.1	5.5±1
S82	46224519	96079528	KSC-16-108	S	3-F-6-Me	0.8±0.1	5±1
S83	46224528	96079529	KSC-16-109	S	3-F-4-Me	0.9±0.2	5.2±1.2
S84	46224530	96079530	KSC-16-110	S	3-F-4-OMe	0.9±0.1	4.7±0.8
S85	46224526	96079531	KSC-16-112	S	3-Cl-6-Me	0.8±0.07	7±1.5
S86	46224518	96079532	KSC-16-113	S	3-Cl-6-OMe	1.1±0.2	16±6
S87	46829342	99206553	KSC-16-120	S	3-F-6-OMe	6.7±3	9.3±1
S88	45108363	92252640	KSC-1-288	S	2-Br	64±24	13±2
S89	2384230	92252641	KSC-1-289	S	2-Cl	69±25	13±2
S90	46829335	99239925	KSC-16-150	S	3-CF_3	50±16	21±1
S91	46850881	99239930	KSC-16-152	S	3-I	5.2±1.9	36±4
S92	46873821	99313588	KSC-16-188	S	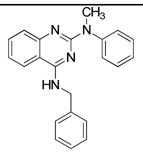	>30	9.2±1.2

* Designates whether a compound was purchased (P) or synthesized (S)
[#] Compound identifier used in the main text.

Table S5: Analogues of N^4-benzyl-N^2-(3-chlorophenyl)quinazoline-2,4-diamine

**compound identifiers**					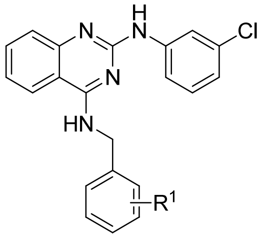	**p97 inhibition**	
**Cpd**	**CID**	**SID**	**KU SCC #**	*****	**R1**	**ATPase IC_{50} (μM)**	**$Ub^{G76V}GFP$ IC_{50} (μM)**
S93	46173070	96022083	KSC-16-72	S	4-Me	1.4±0.3	12±2
S94	46173066	96022090	KSC-16-89	S	4-Cl	2.8±0.9	7.6±2.4
S95 **12**[#]	46173057	96022093	KSC-16-98	S	4-F	1.5±0.3	5.7±1.3
S96	46173059	96022096	KSC-16-101	S	4-Br	7.4±2	10±3
S97	46173063	96022086	KSC-16-79	S	4-OMe	2.3±0.6	11±4

S98	46173069	96022080	KSC-16-63	S	2-Me	4.2±1.1	11±3
S99	46173062	96022087	KSC-16-84	S	2-Cl	4.7±1.4	18±5
S100	46173072	96022091	KSC-16-92	S	2-F	1.8±0.5	7.8±2.1
S101	46173058	96022094	KSC-16-99	S	2-Br	4±1.7	12±5
S102	46173067	96022084	KSC-16-75	S	2-OMe	1.6±0.4	11±2
S103	46173061	96022081	KSC-16-66	S	3-Me	2.3±0.6	9.7±2.7
S104	46173068	96022088	KSC-16-87	S	3-Cl	5±1.2	15±4
S105	46173060	96022092	KSC-16-95	S	3-F	1.2±0.2	6.3±1.7
S106	46173065	96022095	KSC-16-100	S	3-Br	3.7±0.9	9.9±2
S107	46173071	96022085	KSC-16-78	S	3-OMe	1±0.1	9.9±1.6

* Designates whether a compound was purchased (P) or synthesized (S)

[#] Compound identifier used in the main text.

Table S6: Analogues of N^2,N^4-dibenzylquinazoline-2,4-diamine (DBeQ)

**compound identifiers**					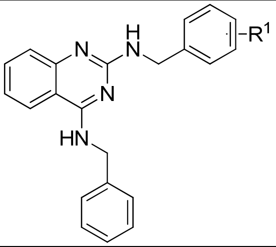	**p97 inhibition**	
**Cpd**	**CID**	**SID**	**KU SCC #**	*****	**R1**	**ATPase IC_{50} (μM)**	**$Ub^{G76V}GFP$ IC_{50} (μM)**
S108 **13**[#]	45382111	93374187	KSC-16-2	S	4-OMe	3±0.5	1.3±0.2
S109 **45**[#]	25144450	93374186	KSC-1-300	S	4-Me	3±0.3	1.5±0.4
S110	45382112	93374224	KSC-16-33	S	4-Cl	3.1±0.2	2.1±0.4
S111	45382121	93374227	KSC-16-38	S	4-F	2.6±0.2	3.4±0.5
S112	45382119	93374230	KSC-16-42	S	4-Br	4.3±0.9	3.9±0.3
S113	45382120	93374191	KSC-16-8	S	2-Me	2.7±0.5	1.9±0.4
S114	45382117	93374222	KSC-16-31	S	2-Cl	5.3±1.2	2.9±0.9

S115	45382108	93374225	KSC-16-35	S	2-F	2.9±0.4	2.8±0.4
S116	45382107	93374228	KSC-16-40	S	2-Br	2.0±0.3	3.8±0.7
S117	45382105	93374193	KSC-16-29	S	2-OMe	3.6±0.8	1.9±0.2
S118	45382113	93374192	KSC-16-28	S	3-Me	2.3±0.4	2.5±0.5
S119	45382114	93374223	KSC-16-32	S	3-Cl	2.7±0.1	2.5±0.4
S120<br>**46**#	45382109	93374190	KSC-16-6	S	4-CF_3	2.6±0.4	2.3±0.3
S121<br>**47**#	45382116	93374221	KSC-16-30	S	3-OMe	4.5±0.8	2.5±0.8
S122<br>**48**#	45382110	93374226	KSC-16-36	S	3-F	3.1±0.4	2.8±0.4
S123<br>**49**#	45382118	93374229	KSC-16-41	S	3-Br	3.7±0.4	2.8±0.4
S124	45382106	93374188	KSC-16-3	S	3,4-di-Cl	3±0.5	1.9±0.2
S125	45382115	93374189	KSC-16-4	S	4-Cl-3-CF_3	4.7±1	2.2±0.4

* Designates whether a compound was purchased (P) or synthesized (S)

# Compound identifier used in the main text.

Table S7: Analogues of N^2,N^4-dibenzylquinazoline-2,4-diamine (DBeQ)

**compound identifiers**					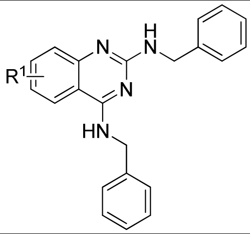	**p97 inhibition**	
**Cpd**	**CID**	**SID**	**KU SCC #**	*****	**R1**	**ATPase IC_{50} (μM)**	**Ub^{G76V}GFP IC_{50} (μM)**
S126	46224525	96079533	KSC-16-114	S	5-Cl	2.2±0.7	16±2
S127	46224521	96079534	KSC-16-115	S	5-F	1.2±0.3	13±2
S128	46224520	96079535	KSC-16-117	S	6-Cl	1.6±0.4	6.6±0.9
S129	5276745	96022097	KSC-16-102	S	6,7-di-OMe	4.4±1.8	8.8±1.5
S130<br>**14**#	46829340	99206522	KSC-16-121c	S	8-OMe	0.6±0.06	10±2
S131	46829333	99206552	KSC-16-118	S	7-Me	2.2±0.4	6.1±1.6

S132	46850874	99239928	KSC-16-160c	S	7-CF_3	9.1±2	19±1
S133	46850882	99239931	KSC-16-153	S	8-Br	3.3±0.9	30±2
S134	46850883	99239932	KSC-16-154	S	8-F	3.1±0.5	18±2
S135	46850884	99239934	KSC-16-156	S	7-F	2.6±0.5	6.1±0.8
S136	46850880	99239935	KSC-16-159	S	7-Cl	5.5±0.9	6.3±0.9
S137	46850885	99239938	KSC-16-166	S	7-OMe	5.7±1	42±6
S138	46850877	99239939	KSC-16-172	S	6-OMe	7.5±1.1	8.6±1.7
S139	46850873	99239941	KSC-16-175	S	6-F	4.7±0.4	8.2±1.5
S140	4685087	99239943	KSC-16-122	S	7-Br	22±4	10±2
S141	46850869	99239929	KSC-16-167C	S	7-CN	25±10	21±3

* Designates whether a compound was purchased (P) or synthesized (S)

[#] Compound identifier used in the main text.

Table S8: N^2 Constrained DBeQ analogues

**compound identifiers**					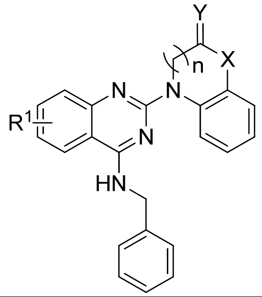				**p97 inhibition**	
**Cpd**	**CID**	**SID**	**KU SCC #**	*****	**R1**	**n**	**X**	**Y**	**ATPase IC_{50} (μM)**	**$Ub^{G76V}GFP$ IC_{50} (μM)**
S142	46829336	99239922	KSC-16-125	S	H	1	C	-H	4.4±1.8	10±2
S143	46829332	99239923	KSC-16-144	S	H	0	C	-H	2±0.5	11±3
S144 **15**[#]	46173064	96022082	KSC-16-70	S	H	1	O	-H	0.6±0.2	7±1.9
S145	46829338	99206557	KSC-16-147	S	H	1	NH	-H	0.4±0.05	6.3±1
S146	46873816	99313586	KSC-16-182	S	8-OMe	1	NH	-H	0.9±0.1	3.7±0.2
S147 **38**[#]	46873819	99313591	KSC-16-191	S	8-OMe	1	O	-H	0.2±0.02	3.3±0.4

S148	46931212	99437738	KSC-16-232	S	8-OH	1	O	-H	1.2±0.3	7.7±1.7
S149	49830264	103904180	KSC-16-255	S	8-Ph	1	O	-H	3.5±0.6	5±0.9
S150 **33**[#]	49830253	103904181	KSC-16-260	S	8-OCH_2CH_2OH	1	O	-H	0.17±0.05	3.8±0.8
S151 **34**[#]	49852181	104221957	KSC-25-29	S	8-OCH_2CH_2OMe	1	O	-H	0.6±0.03	6.5±0.7
S152 **35**[#]	49830265	103904183	KSC-16-265	S	8-$OCH_2CH_2NEt_2$	1	O	-H	0.4±0.08	5.3±0.6
S153 **36**[#]	49830267	103904184	KSC-16-268	S	8-p-OMePh	1	O	-H	1.1±0.1	9±1
S154 **37**[#]	49852171	104221958	KSC-25-30	S	8-*n*-Bu	1	O	-H	2.63±0.7	28±3
S155 **39**[#]	50918589	113284765	KSC-206-005	S	8-OCH_2CN	1	O	-H	0.4±0.08	7.7±0.7
S156	49852177	104221952	KSC-25-17	S	8-OMe	1	NMe	-H	3.1±0.5	7.8±0.7
S157	49852173	104221953	KSC-25-15c1	S	8-OMe	1	NCOMe	-H	2.7±0.6	8±1
S158 **ML240**[#]	49830258	103904169	KSC-16-270 (ML240)	S	8-OMe	0	NH	=NH	0.11±0.03	0.9±0.1
S159	49830270	103904172	KSC-16-262cc	S	8-OMe	0	O	=O	0.11±0.03	5±1
S160	49852176	104221943	KSC-25-3	S	8-OMe	0	N**	-H	3.3±1.1	78±45
S161 **18**[#]	50918580	113284753	KSC-25-31	S	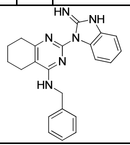				0.05±0.003	1.8±0.2

* Designates whether a compound was purchased (P) or synthesized (S)
** unsubstituted nitrogen of a benzimidazole moiety

[#] Compound identifier used in the main text.

Table S9: Alternatives to the quinazoline scaffold analogues

compound identifiers					structure	p97 inhibition	
**Cpd**	**CID**	**SID**	**KU SCC #**	*****	**Formulas X to XII**	**ATPase IC$_{50}$ (μM)**	**Ub^{G76V}GFP IC$_{50}$ (μM)**
**ML241**[#]	49830260	103904185	KSC-16-290 (ML241)	S	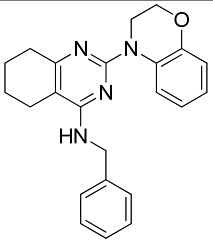	0.11±0.03	3.5±0.4
S162 **17**[#]	49852184	104221950	KSC-25-14	S	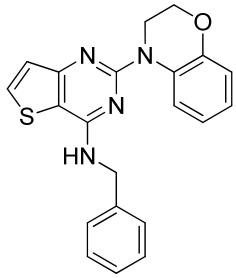	0.4±0.1	6.4±1
S163 **31**[#]	49852172	104221955	KSC-25-24	S	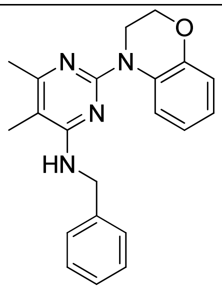	1.1±0.2	10±1
S164	50918581	113284766	KSC-25-32	S	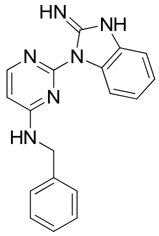	2.8±0.6	14±3
S165 **32**[#]	49852180	104221956	KSC-25-28	S	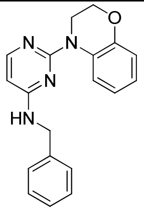	2.9±0.2	27±3

* Designates whether a compound was purchased (P) or synthesized (S)

[#] Compound identifier used in the main text.

Table S10: Miscellaneous 8-methoxyquinazoline analogues

compound identifiers					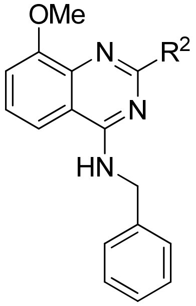	p97 inhibition	
Cpd	CID	SID	KU SCC #	*	R2	ATPase IC_{50} (μM)	$Ub^{G76V}GFP$ IC_{50} (μM)
S166	49852185	104221947	KSC-25-22	S	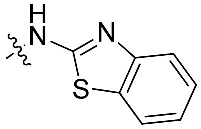	0.5±0.2	14±2
S167 **19**[#]	49852183	104221949	KSC-25-21	S	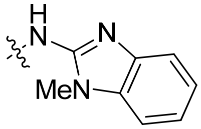	0.3±0.07	7.8±1.2
S168	49852174	104221945	KSC-25-12	S	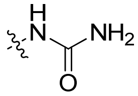	49±10	108±31
S169	49852175	104221954	KSC-25-19	S	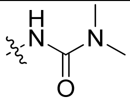	60±23	37±7
S170 **20**[#]	25144452	99313587	KSC-16-187	S	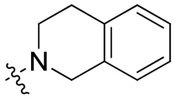	15±3	7±1.3
S171	46931213	99437735	KSC-16-203	S	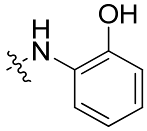	2.6±0.4	2.8±0.5
S172	49830269	103904171	KSC-16-273	S	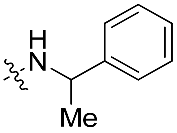	12±5	2.1±0.7
S173	49830255	103904174	KSC-16-278	S	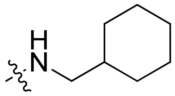	5.4±0.9	4.2±1

S174<br>**50**#	46873815	99313592	KSC-16-193	S	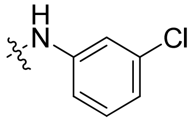	0.5±0.1	5.4±1
S175<br>**51**#	46873818	99313593	KSC-16-194	S	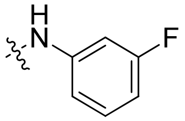	0.52±0.04	4.8±0.7
S176<br>**52**#	49830257	103904175	KSC-16-282	S	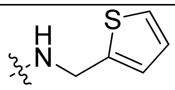	1.7±0.4	1.8±0.3
S177	49830266	103904176	KSC-16-283	S	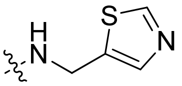	11±1	10±3
S178	46873813	99313594	KSC-16-196	S	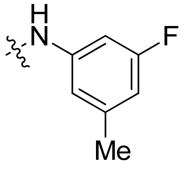	1.5±0.2	7.1±0.5

* Designates whether a compound was purchased (P) or synthesized (S)

# Compound identifier used in the main text.

Table S11: 3,4-Dihydro-1,4-benzoxazine analogues incorporating the quinazoline of **ML241**

compound identifiers					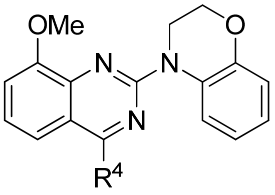	p97 inhibition	
**Cpd**	**CID**	**SID**	**KU SCC #**	*****	**R4**	**ATPase**<br>**IC_{50} (μM)**	**$Ub^{G76V}GFP$**<br>**IC_{50} (μM)**
S179<br>**40**#	49830256	103904182	KSC-16-261	S	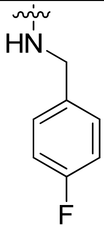	1.9±0.4	3.7±0.6

S180<br>**41**[#]	49830261	103904186	KSC-16-295	S	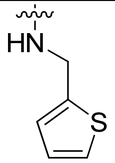	1.2±0.2	3±0.6
S181<br>**42**[#]	49830262	103904187	KSC-16-299	S	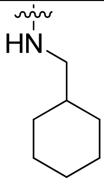	7.4±0.9	8.2±1
S182	46931214	99437736	KSC-16-219	S	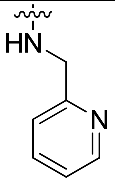	19±5	15±4
S183<br>**43**[#]	46931210	99437737	KSC-16-222	S	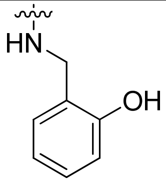	4.6±1	6±0.6
S184	46931211	99437740	KSC-16-235c2	S	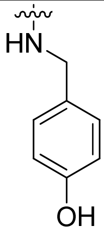	2.4±0.5	7.3±1
S185	46931215	99437739	KSC-16-227	S	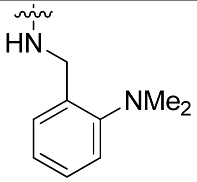	47±22	4.5±0.5
S186	49830271	103904178	KSC-16-243	S	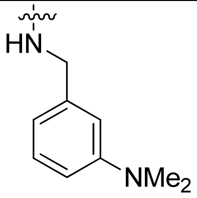	52±10	19±4

S187	49830254	103904179	KSC-16-251	S	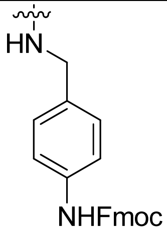	33±7	23±2
S188	49852170	104221951	KSC-25-6	S	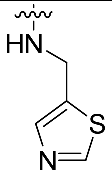	38±7	20±3
* Designates whether a compound was purchased (P) or synthesized (S)<br><br># Compound identifier used in the main text.							

Table S12: Unabridged compound purity assessment via HPLC analysis.

internal compound ID	compound number(s)	HPLC purity (%)[1]
KSC-1-150	**1, S1**	100.0
KSC-1-146	**2, S2**	100.0
KSC-1-149	**3, S3**	94.5
KSC-1-193	**4, S19**	99.2
KSC-1-194	**5, S20**	99.5
KSC-1-152	**6, S35**	92.0
KSC-1-151	**7, S36**	100.0
KSC-1-258	**8, S34**	100.0
KSC-16-146	**9, S56**	98.8
KSC-1-153	**10, S58**	99.1
KSC-1-244	**11, S68**	96.7
KSC-16-98	**12, S95**	100.0
KSC-16-2	**13, S108**	96.8
KSC-16-121c	**14, S130**	98.9
KSC-16-70	**15, S144**	99.0
KSC-206-026	**16**	96.1
KSC-25-14	**17, S162**	95.3
KSC-25-31	**18, S161**	81.2
KSC-16-290	**ML241**	98.3
KSC-16-270	**ML240, S158**	100.0
KSC-25-21	**19, S167**	96.9
KSC-16-187	**20, S170**	83.1
KSC-25-40	**21**	95.6
KSC-25-23	**22**	94.4
KSC-25-38	**23**	97.8

KSC-206-023	**24**	95.5
KSC-206-022	**25**	93.2
KSC-206-019	**26**	90.2
KSC-206-017	**27**	98.8
KSC-206-017-P2	**28**	96.8
KSC-206-015	**29**	99.0
KSC-206-007	**30**	98.8
KSC-25-24	**31, S163**	100.0
KSC-25-28	**32, S165**	100.0
KSC-16-260	**33, S150**	100.0
KSC-25-29	**34, S151**	98.0
KSC-16-265	**35, S152**	68.2
KSC-16-268	**36, S153**	92.9
KSC-25-30	**37, S154**	100.0
KSC-16-191	**38, S147**	100.0
KSC-206-005	**39, S155**	96.0
KSC-16-261	**40, S179**	98.8
KSC-16-295	**41,S180**	96.6
KSC-16-299	**42, S181**	95.0
KSC-16-222	**43, S183**	100.0
KSC-1-292	**44, S74**	100.0
KSC-1-300	**45, S109**	97.6
KSC-16-6	**46, S120**	99.0
KSC-16-30	**47, S121**	100.0
KSC-16-36	**48, S122**	100.0
KSC-16-41	**49, S123**	99.0
KSC-16-193	**50, S174**	98.9
KSC-16-194	**51, S175**	100.0
KSC-16-282	**52, S176**	100.0
KSC-1-145	**S4**	100.0
KSC-1-226	**S5**	100.0
KSC-1-236	**S6**	100.0
KSC-1-234	**S7**	94.8
KSC-1-227	**S8**	98.5
KSC-1-224	**S9**	100.0
KSC-1-212	**S10**	92.5
KSC-1-219	**S11**	96.8
KSC-1-220	**S12**	95.5
KSC-1-221	**S13**	97.5
KSC-1-222	**S14**	100.0
KSC-1-223	**S15**	98.1
KSC-1-225	**S16**	100.0
KSC-1-237	**S17**	98.9
KSC-1-216	**S18**	100.0
KSC-1-195	**S21**	99.3

KSC-1-213	**S22**	100.0
KSC-1-147	**S23**	99.1
KSC-1-148	**S24**	100.0
KSC-1-200	**S25**	100.0
KSC-1-211	**S26**	100.0
KSC-1-214	**S27**	97.7
KSC-1-215	**S28**	100.0
KSC-1-217	**S29**	100.0
KSC-1-218	**S30**	100.0
KSC-1-235	**S31**	100.0
KSC-1-238	**S32**	100.0
KSC-1-257	**S33**	96.6
KSC-1-240	**S37**	100.0
KSC-1-260	**S38**	100.0
KSC-1-208	**S39**	100.0
KSC-1-203	**S40**	100.0
KSC-1-242	**S41**	81.2
KSC-1-241	**S42**	98.2
KSC-1-239	**S43**	96.1
KSC-1-254	**S44**	96.5
KSC-16-190	**S45**	97.7
KSC-16-272	**S46**	100.0
KSC-16-277	**S47**	98.6
KSC-25-10	**S48**	100.0
KSC-25-16	**S49**	98.2
KSC-16-149	**S50**	96.2
KSC-16-163	**S51**	100.0
KSC-16-164	**S52**	100.0
KSC-16-174	**S53**	98.4
KSC-16-176	**S54**	97.8
KSC-16-151	**S55**	100.0
KSC-1-243	**S57**	100.0
KSC-1-251	**S59**	100.0
KSC-1-246	**S60**	100.0
KSC-1-249	**S61**	100.0
KSC-1-245	**S62**	97.7
KSC-1-250	**S63**	91.5
KSC-1-252	**S64**	100.0
KSC-1-247	**S65**	97.7
KSC-1-248	**S66**	96.1
KSC-1-253	**S67**	100.0
KSC-1-259	**S69**	100.0
KSC-1-290	**S70**	92.1
KSC-1-293	**S71**	98.5
KSC-1-291	**S72**	99.0

KSC-16-155	**S73**	100.0
KSC-1-295	**S75**	98.2
KSC-1-294	**S76**	98.6
KSC-16-103	**S77**	96.3
KSC-16-104	**S78**	100.0
KSC-16-105	**S79**	100.0
KSC-16-106	**S80**	97.6
KSC-16-107	**S81**	99.0
KSC-16-108	**S82**	100.0
KSC-16-109	**S83**	100.0
KSC-16-110	**S84**	100.0
KSC-16-112	**S85**	100.0
KSC-16-113	**S86**	96.4
KSC-16-120	**S87**	96.5
KSC-1-288	**S88**	100.0
KSC-1-289	**S89**	100.0
KSC-16-150	**S90**	100.0
KSC-16-152	**S91**	100.0
KSC-16-188	**S92**	100.0
KSC-16-72	**S93**	100.0
KSC-16-89	**S94**	100.0
KSC-16-101	**S96**	100.0
KSC-16-79	**S97**	100.0
KSC-16-63	**S98**	100.0
KSC-16-84	**S99**	100.0
KSC-16-92	**S100**	100.0
KSC-16-99	**S101**	100.0
KSC-16-75	**S102**	100.0
KSC-16-66	**S103**	100.0
KSC-16-87	**S104**	100.0
KSC-16-95	**S105**	100.0
KSC-16-100	**S106**	100.0
KSC-16-78	**S107**	100.0
KSC-16-38	**S111**	100.0
KSC-16-42	**S112**	98.1
KSC-16-8	**S113**	97.5
KSC-16-31	**S114**	98.9
KSC-16-35	**S115**	97.7
KSC-16-40	**S116**	100.0
KSC-16-29	**S117**	97.3
KSC-16-28	**S118**	97.2
KSC-16-32	**S119**	98.4
KSC-16-33	**S110**	98.4
KSC-16-3	**S124**	97.4
KSC-16-4	**S125**	98.6

KSC-16-114	**S126**	100.0
KSC-16-115	**S127**	99.0
KSC-16-117	**S128**	100.0
KSC-16-102	**S129**	100.0
KSC-16-118	**S131**	92.0
KSC-16-160c	**S132**	98.6
KSC-16-153	**S133**	100.0
KSC-16-154	**S134**	100.0
KSC-16-156	**S135**	100.0
KSC-16-159	**S136**	100.0
KSC-16-166	**S137**	100.0
KSC-16-172	**S138**	93.2
KSC-16-175	**S139**	98.0
KSC-16-122	**S140**	96.8
KSC-16-167c	**S141**	97.5
KSC-16-125	**S142**	96.1
KSC-16-144	**S143**	94.8
KSC-16-147	**S145**	60.6
KSC-16-182	**S146**	19.5
KSC-16-232	**S148**	100.0
KSC-16-255	**S149**	95.6
KSC-25-17	**S156**	75.0
KSC-25-15c1	**S157**	96.5
KSC-16-262cc	**S159**	93.9
KSC-25-3	**S160**	99.2
KSC-25-32	**S164**	84.4
KSC-25-22	**S166**	97.9
KSC-25-12	**S168**	98.8
KSC-25-19	**S169**	91.1
KSC-16-203	**S171**	100.0
KSC-16-273	**S172**	100.0
KSC-16-278	**S173**	93.5
KSC-16-283	**S177**	100.0
KSC-16-196	**S178**	100.0
KSC-16-219	**S182**	94.9
KSC-16-235c2	**S184**	95.6
KSC-16-227	**S185**	91.5
KSC-16-243	**S186**	81.3
KSC-16-251	**S187**	97.0
KSC-25-6	**S188**	98.4

[1] Compound purity was measured on the basis of peak integration (area under the curve) from UV/vis absorbance (at 214 nm), and compound identity was determined on the basis of mass analysis.

Table S13: Aqueous solubility for select compounds.

compound identifiers		structure	solubility[1] (μg/mL) pH = 5.0/6.2/7.4
**cpd**	**KU SCC #**		
S175 **51**[#]	KSC-16-294	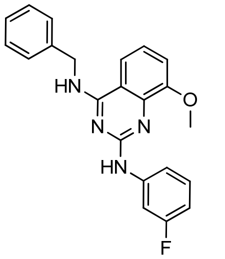	0.09/<0.01/<0.01
S84	KSC-16-110	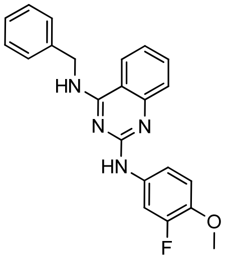	3.4/0.03/0.08
S79	KSC-16-105	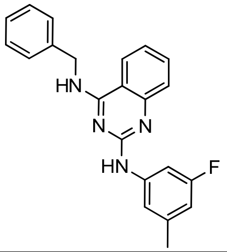	0.05/<0.01/<0.01
S82	KSC-16-108	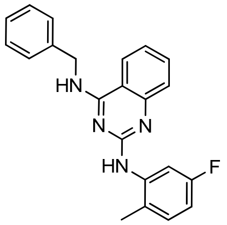	5.6/0.07/0.04
S68 **11**[#]	KSC-1-244	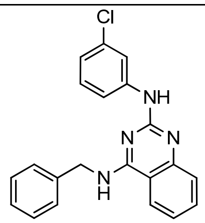	0.04/0.01/0.02
S144 **15**[#]	KSC-16-70	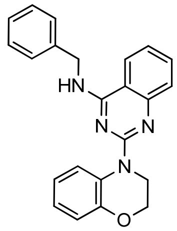	25.2/0.72/0.48

S-83	KSC-16-109	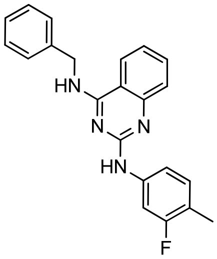	0.13/0.04/0.05
S78	KSC-16-104	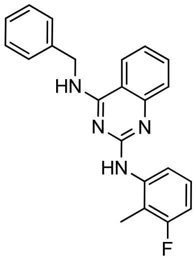	8.4/0.05/<0.01
S146	KSC-16-182	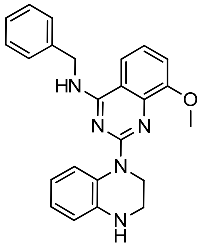	28.2/10.2/0.30
S174 **50**[#]	KSC-16-193	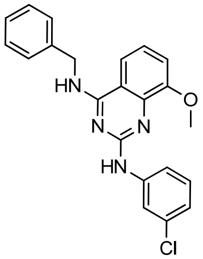	0.08/0.05/<0.01
S77	KSC-16-103	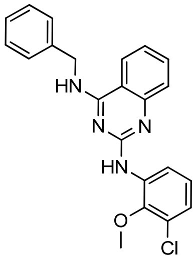	0.50/0.23/0.06
S130 **14**[#]	KSC-16-121c	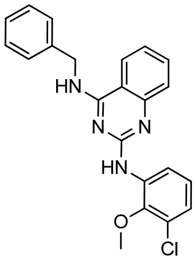	3.2/1.0/0.03

S145	KSC-16-147	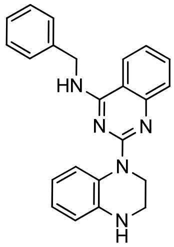	>36/18/0.02
S133	KSC-16-153	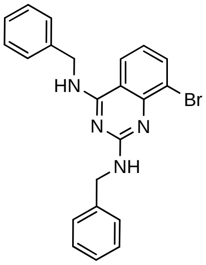	0.42/0.13/<0.01
S71	KSC-1-293	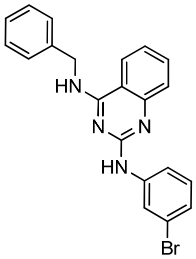	0.03/0.01/<0.01
S116	KSC-16-40	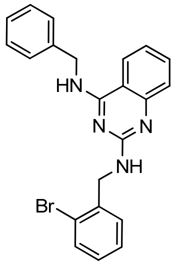	0.21/0.24/0.14
S123 **49**#	KSC-16-41	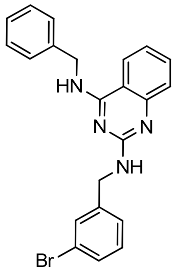	0.37/0.25/0.17
S106	KSC-16-100	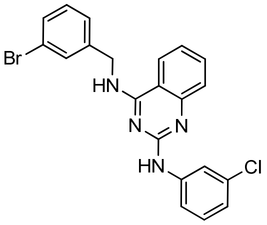	0.10/0.02/<0.01

S158<br>**ML240**[#]	KSC-16-270	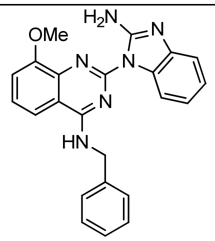	0.06/0.09/0.05
S159	KSC-16-262cc	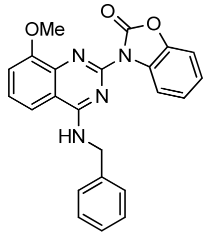	0.02/<0.01/<0.01
S150	KSC-16-260	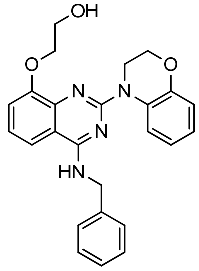	21.5/0.48/0.43
**ML241**[#]	KSC-16-290	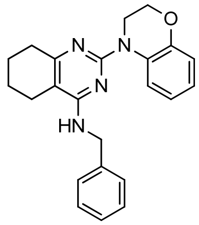	28/0.13/0.20

[1] in aqueous PION's buffer, pH's 5.0/6.2/7.4
[#] Compound identifier used in the main text.

Table S14: Kinase/ATPase profiling for **ML240** and **ML241**.

kinase	annotation	sequence	labeling site	% Inhibition (20 μM)	
				**ML240**	**ML241**
ABL,ARG	Proto-oncogene tyrosine-protein kinase ABL1 [Homo sapiens (Human)]	LMTGDTYTAHAGAkFPIK	Activation Loop	-12.6	-13.9
ABL,ARG	Proto-oncogene tyrosine-protein kinase ABL1 [Homo sapiens (Human)]	YSLTVAVkTLKEDTMEVEEFLK	Lys1	11.1	5.3
ACK	Activated CDC42 kinase 1 [Homo sapiens (Human)]	TVSVAVkCLKPDVLSQPEAMDDFIR	Lys1	18.9	-11.3
AGK	Acylglycerol kinase, mitochondrial n=2 Tax=Homo sapiens RepID=AGK_HUMAN	ATVFLNPAACkGK	ATP	7.5	5.2
AMPKa1	5'-AMP-activated protein kinase catalytic subunit alpha-1 [Homo sapiens (Human)]	IGHYILGDTLGVGTFGkVK	ATP Loop	8.3	11.5
AMPKa1,AMPKa2	5'-AMP-activated protein kinase catalytic subunit alpha-1 [Homo sapiens (Human)]	VAVkILNR	Lys1	-19.2	-12.5
AMPKa1,AMPKa2	5'-AMP-activated protein kinase catalytic subunit alpha-1 [Homo sapiens (Human)]	DLkPENVLLDAHMNAK	Lys2	-1.5	-0.9
AMPKa2	5'-AMP-activated protein kinase catalytic subunit alpha-2 [Homo sapiens (Human)]	IGHYVLGDTLGVGTFGkVK	ATP Loop	11.9	-6.2
ANPb	Atrial natriuretic peptide receptor B precursor	GMAFLHNSIISSHGSLkSSNCVVDSR	Lys2	5	-6.3

	[Homo sapiens (Human)]				
ARAF	A-Raf proto-oncogene serine/threonine-protein kinase [Homo sapiens (Human)]	DLkSNNIFLHEGLTVK	Lys2	-7.3	3
ATM	Serine-protein kinase ATM [Homo sapiens (Human)]	QLVkGRDDLRQDAVMQQVFQMCNTLLQR	ATP	-4.3	-8.3
ATR	Serine/threonine-protein kinase ATR [Homo sapiens (Human)]	FYIMMCkPK	ATP	-0.4	-7.4
ATR	Serine/threonine-protein kinase ATR [Homo sapiens (Human)]	kAGHHQTAYNALLNAGESR	Protein Kinase Domain	1.6	-7.8
AurA	Serine/threonine-protein kinase 6 [Homo sapiens (Human)]	FILALkVLFK	Lys1	10.3	-1.8
AurA	Serine/threonine-protein kinase 6 [Homo sapiens (Human)]	DIkPENLLLGSAGELK	Lys2	11.9	-1
AurB	Serine/threonine-protein kinase 12 [Homo sapiens (Human)]	SHFIVALkVLFK	Lys1	-32.8	-14.3
BARK1	Beta-adrenergic receptor kinase 1 n=1 Tax=Homo sapiens RepID=ARBK1_HUMAN	DLkPANILLDEHGHVR	Lys2	-14.5	-6.7
BCKDK	[3-methyl-2-oxobutanoate dehydrogenase [lipoamide]] kinase, mitochondrial precursor [Homo sapiens (Human)]	QLLDDHkDVVTLLAEGLR	Other	-9.9	-9.4
BRAF	B-Raf proto-oncogene serine/threonine-protein kinase [Homo sapiens (Human)]	DLkSNNIFLHEDLTVK	Lys2	10.5	14.3
CaMK1a	Calcium/calmodulin-dependent protein kinase type 1 [Homo sapiens (Human)]	LVAIkCIAK	Lys1	-2.1	1.2
CaMK1d	Calcium/calmodulin-dependent protein kinase type 1D [Homo sapiens (Human)]	LFAVkCIPK	Lys1	0	-2.2
CaMK2a,CaMK2b,CaMK2d,CaMK2g	Calcium/calmodulin-dependent protein kinase type II alpha chain [Homo sapiens (Human)]	DLkPENLLLASK	Lys2	5.7	-0.5
CaMK2b,CaMK2d	Calcium/calmodulin-dependent protein kinase type II beta chain [Homo sapiens (Human)]	LCTGHEYAAkIINTK	Lys1	-1.1	12.7
CaMK2d	Calcium/calmodulin-dependent protein kinase type II delta chain n=2 Tax=Euarchontoglires RepID=KCC2D_HUMAN	IPTGQEYAAkIINTK	Lys1	-14.3	-17.7
CaMK2g	Calcium/calmodulin-dependent protein kinase type II gamma chain [Homo sapiens (Human)]	KTSTQEYAAkIINTKK	Lys1	-8.6	-9.5
CaMK2g	Calcium/calmodulin-dependent protein kinase type II gamma chain [Homo sapiens (Human)]	TSTQEYAAkIINTK	Lys1	7.5	6.7
CaMKK1	Calcium/calmodulin-dependent protein kinase kinase 1 [Homo sapiens (Human)]	LAYNESEDRHYAMkVLSK	Lys1	-24.9	-7.1
CaMKK2	Calcium/calmodulin-dependent protein kinase kinase 2 [Homo sapiens (Human)]	LAYNENDNTYYAMkVLSK	Lys1	-5.8	0.1
CASK	Peripheral plasma membrane protein CASK n=2 Tax=Catarrhini RepID=CSKP_HUMAN	ETGQQFAVkIVDVAK	Lys1	5.7	-0.4
CCRK	Cell cycle-related kinase [Homo sapiens (Human)]	DLkPANLLISASGQLK	Lys2	2.1	4.4
CDC2	Cell division control protein 2 homolog [Homo sapiens (Human)]	DLkPQNLLIDDKGTIK	Lys2	-2.6	-6.5
CDK10	Cell division protein kinase 10 [Homo sapiens (Human)]	DLkVSNLLMTDK	Lys2	4.7	-7.4
CDK11,CDK8	Cell division protein kinase 8 [Homo sapiens (Human)]	DLkPANILVMGEGPER	Lys2	-2.3	-5.3
CDK2	Cell division protein kinase 2 [Homo sapiens (Human)]	LTGEVVALkK	Lys1	-9.5	-2.8
CDK2	Cell division protein kinase 2 [Homo sapiens (Human)]	NKLTGEVVALKk	Lys1	15.7	3.7
CDK2	Cell division protein kinase 2 [Homo sapiens (Human)]	DLkPQNLLINTEGAIK	Lys2	-0.8	-1.9
CDK5	Cell division protein kinase 5 [Homo sapiens (Human)]	ETHEIVALkR	Lys1	-20.3	-10.8
CDK5	Cell division protein kinase 5 [Homo sapiens (Human)]	NRETHEIVALkR	Lys1	-17.2	-18.9

CDK5	Cell division protein kinase 5 [Homo sapiens (Human)]	DLkPQNLLINR	Lys2	10.7	-5.6
CDK7	Cell division protein kinase 7 [Homo sapiens (Human)]	DLkPNNLLLDENGVLK	Lys2	2.5	-1.7
CDK9	Cell division protein kinase 9 [Homo sapiens (Human)]	DMkAANVLITR	Lys2	-14.5	-15.3
CHED	Cell division cycle 2-like protein kinase 5 [Homo sapiens (Human)]	DIkCSNILLNNRGQIK	Lys2	17.4	2.7
CHK1	Serine/threonine-protein kinase Chk1 [Homo sapiens (Human)]	LSkGDGLEFK	Other	-4.2	-3.2
CHK2	Serine/threonine-protein kinase Chk2 [Homo sapiens (Human)]	VAIkIISK	Lys1	-4.9	-5.4
CHK2	Serine/threonine-protein kinase Chk2 [Homo sapiens (Human)]	DLkPENVLLSSQEEDCLIK	Lys2	3.1	4.2
CK1a	Casein kinase I isoform alpha [Homo sapiens (Human)]	DIkPDNFLMGIGR	Lys2	4.9	-1.4
CK1d,CK1e	Casein kinase I isoform delta [Homo sapiens (Human)]	DVkPDNFLMGLGKK	Lys2	-1.5	-4.8
CK2a2	Casein kinase II subunit alpha' [Homo sapiens (Human)]	DVkPHNVMIDHQQK	Lys2	-11.7	-34.4
CLK2	Dual specificity protein kinase CLK2 [Homo sapiens (Human)]	LTHTDLkPENILFVNSDYELTYNLEK	Lys2	-12.9	-5.9
CRK7	Cell division cycle 2-related protein kinase 7 [Homo sapiens (Human)]	DIkCSNILLNNSGQIK	Lys2	-31.4	-22.3
CSK	Tyrosine-protein kinase CSK [Homo sapiens (Human)]	EASSTQDTGkLPVK	Activation Loop	-21.6	-22
CSK	Tyrosine-protein kinase CSK [Homo sapiens (Human)]	VSDFGLTkEASSTQDTGK	Activation Loop	-10.5	-1.8
CSK	Tyrosine-protein kinase CSK [Homo sapiens (Human)]	VSDFGLTkEASSTQDTGKLPVK	Activation Loop	-18	-8.1
DNAPK	DNA-dependent protein kinase catalytic subunit [Homo sapiens (Human)]	GHDEREHPFLVkGGEDLRQDQR	ATP	12.4	1.7
DNAPK	DNA-dependent protein kinase catalytic subunit [Homo sapiens (Human)]	kGGSWIQEINVAEK	ATP	79.1	45.3
eEF2K	Elongation factor 2 kinase [Homo sapiens (Human)]	YIkYNSNSGFVR	Protein Kinase Domain	-5.9	-1.9
EGFR	Epidermal growth factor receptor precursor [Homo sapiens (Human)]	ITDFGLAkLLGAEEK	Activation Loop	12.7	-3.9
EGFR	Epidermal growth factor receptor precursor [Homo sapiens (Human)]	LLGAEEKEYHAEGGkVPIK	Activation Loop	0.1	-3.2
EGFR	Epidermal growth factor receptor precursor [Homo sapiens (Human)]	IPVAIkELR	Lys1	9.7	-7.4
EphA1	Ephrin type-A receptor 1 precursor [Homo sapiens (Human)]	LLDDFDGTYETQGGkIPIR	Activation Loop	-22.2	-26.1
EphA2	Ephrin type-A receptor 2 precursor [Homo sapiens (Human)]	VLEDDPEATYTTSGGkIPIR	Activation Loop	-8.1	-9.6
EphA2	Ephrin type-A receptor 2 precursor [Homo sapiens (Human)]	EVPVAIkTLK	Lys1	-11.4	-5.6
EphA7	Ephrin type-A receptor 7 n=1 Tax=Homo sapiens RepID=EPHA7_HUMAN	VIEDDPEAVYTTTGGkIPVR	Activation Loop	2.9	12
EphB2	Ephrin type-B receptor 2 precursor [Homo sapiens (Human)]	FLEDDTSDPTYTSALGGkIPIR	Activation Loop	8.3	2.6
EphB4	Ephrin type-B receptor 4 precursor [Homo sapiens (Human)]	FLEENSSDPTYTSSLGGkIPIR	Activation Loop	-8.8	-4
Erk1,Erk2	Mitogen-activated protein kinase 3 [Homo sapiens (Human)]	DLkPSNLLINTTCDLK	Lys2	3.6	3.5
Erk1,Erk2	Mitogen-activated protein kinase 3 [Homo sapiens (Human)]	YIHSANVLHRDLkPSNLLINTTCDLK	Lys2	-12.2	8.6
Erk1,Erk2	Mitogen-activated protein kinase 1 [Homo sapiens]	YIHSANVLHRDLkPSNLLLNTTCDLK	Lys2	-10	5.3
Erk5	Mitogen-activated protein kinase 7 n=2 Tax=Homo sapiens RepID=MK07_HUMAN	DLkPSNLLVNENCELK	Lys2	-14.3	-6.4
FAK	Focal adhesion kinase 1 [Homo sapiens	YMEDSTYYkASK	Activation	-20.3	6.5

	(Human)]		Loop		
FAK	Focal adhesion kinase 1 [Homo sapiens (Human)]	CIGEGQFGDVHQGIYMSPENPALAVAIkTCK	Lys1	6.5	-2
FER	Proto-oncogene tyrosine-protein kinase FER n=2 Tax=Homo sapiens RepID=FER_HUMAN	QEDGGVYSSSGLkQIPIK	Activation Loop	-27.4	-20.1
FER	Proto-oncogene tyrosine-protein kinase FER n=2 Tax=Homo sapiens RepID=FER_HUMAN	TSVAVkTCKEDLPQELK	Lys1	4.8	-1.8
FRAP	FKBP12-rapamycin complex-associated protein [Homo sapiens (Human)]	IQSIAPSLQVITSkQRPR	ATP	22.6	-0.2
FYN	Proto-oncogene tyrosine-protein kinase Fyn n=2 Tax=Homo sapiens RepID=FYN_HUMAN	VAIkTLKPGTMSPESFLEEAQIMK	Lys1	-15.9	-18
FYN,SRC,YES	Proto-oncogene tyrosine-protein kinase Fyn n=2 Tax=Homo sapiens RepID=FYN_HUMAN	QGAkFPIKWTAPEAALYGR	Activation Loop	7.4	1.5
GCK	Mitogen-activated protein kinase kinase kinase kinase 2 n=1 Tax=Homo sapiens RepID=M4K2_HUMAN	DTVTSELAAVkIVK	Lys1	6.7	5
GCK	Mitogen-activated protein kinase kinase kinase kinase 2 n=1 Tax=Homo sapiens RepID=M4K2_HUMAN	DIkGANLLLTLQGDVK	Lys2	-34	-22.6
GCN2 domain2	Eukaryotic translation initiation factor 2-alpha kinase 4 [Homo sapiens (Human)]	LDGCCYAVkR	Lys1	-15.1	-13.9
GCN2 domain2	Eukaryotic translation initiation factor 2-alpha kinase 4 [Homo sapiens (Human)]	VQNKLDGCCYAVkR	Lys1	2.3	-5.4
GCN2 domain2	Eukaryotic translation initiation factor 2-alpha kinase 4 [Homo sapiens (Human)]	DLkPVNIFLDSDDHVK	Lys2	12.6	1
GSK3A	Glycogen synthase kinase-3 alpha [Homo sapiens (Human)]	DIkPQNLLVDPDTAVLK	Lys2	2	0
GSK3B	Glycogen synthase kinase-3 beta n=2 Tax=Homo sapiens RepID=GSK3B_HUMAN	DIkPQNLLLDPDTAVLK	Lys2	1	-3.9
HER2/ErbB2	Receptor tyrosine-protein kinase erbB-2 precursor [Homo sapiens (Human)]	GIWIPDGENVKIPVAIkVLR	Lys1	32.6	0.8
IKKa	Inhibitor of nuclear factor kappa-B kinase alpha subunit [Homo sapiens (Human)]	DLkPENIVLQDVGGK	Lys2	8.5	10.8
IKKb	Inhibitor of nuclear factor kappa B kinase beta subunit [Homo sapiens (Human)]	DLkPENIVLQQGEQR	Lys2	-13.4	-6.4
IKKe,TBK1	Inhibitor of nuclear factor kappa-B kinase epsilon subunit [Homo sapiens (Human)]	DIkPGNIMR	Lys2	-6.7	-9.7
ILK	Integrin-linked protein kinase 1 [Homo sapiens (Human)]	WQGNDIVVkVLK	Lys1	1.5	0.2
ILK	Integrin-linked protein kinase 1 [Homo sapiens (Human)]	ISMADVkFSFQCPGR	Protein Kinase Domain	-2.4	1.8
IRAK1	Interleukin-1 receptor-associated kinase 1 [Homo sapiens (Human)]	AIQFLHQDSPSLIHGDIkSSNVLLDER	Lys2	-2.2	-2.4
IRAK4	Interleukin-1 receptor-associated kinase 4 [Homo sapiens (Human)]	GYVNNTTVAVkK	Lys1	-14.3	22.5
IRAK4	Interleukin-1 receptor-associated kinase 4 [Homo sapiens (Human)]	DIkSANILLDEAFTAK	Lys2	-23.6	-7.7
IRE1	Endoribonuclease n=1 Tax=Homo sapiens RepID=ERN1_HUMAN	DLkPHNILISMPNAHGK	Lys2	-6.5	-3.3
ITPK1	Inositol-tetrakisphosphate 1-kinase [Homo sapiens (Human)]	ESIFFNSHNVSkPESSSVLTELDKIEGVFERPSDEVIR	Other	-7.7	4.8
JAK1 domain1	Tyrosine-protein kinase JAK1 n=1 Tax=Homo sapiens RepID=JAK1_HUMAN	QLASALSYLEDKDLVHGNVCTkNLLLAR	Other	91.8	7.1
JAK1 domain2	Tyrosine-protein kinase JAK1 n=1 Tax=Homo sapiens RepID=JAK1_HUMAN	IGDFGLTkAIETDKEYYTVK	Activation Loop	1.6	0.5
JAK1 domain2	Tyrosine-protein kinase JAK1 n=1 Tax=Homo sapiens RepID=JAK1_HUMAN	IGDFGLTkAIETDKEYYTVKDDR	Activation Loop	-10.7	4.1
JNK1,JNK2,JNK3	Mitogen-activated protein kinase 8 [Homo sapiens (Human)]	DLkPSNIVVK	Lys2	9.6	-7
KHS1	Mitogen-activated protein kinase kinase kinase kinase 5 [Homo sapiens (Human)]	NVHTGELAAVkIIK	Lys1	-6.4	-7.4
KHS2	Mitogen-activated protein kinase kinase kinase kinase 3 [Homo sapiens (Human)]	NVNTGELAAIkVIK	Lys1	4.8	3.3

LATS1	Serine/threonine-protein kinase LATS1 [Homo sapiens (Human)]	ALYATkTLR	Lys1	-30.5	-8
LATS2	Serine/threonine-protein kinase LATS2 n=1 Tax=Homo sapiens RepID=LATS2_HUMAN	VDTHALYAMkTLR	Lys1	11.4	6.1
LATS2	Serine/threonine-protein kinase LATS2 n=1 Tax=Homo sapiens RepID=LATS2_HUMAN	DIkPDNILIDLDGHIK	Lys2	-0.2	-3.7
LKB1	Serine/threonine-protein kinase 11 [Homo sapiens (Human)]	DIkPGNLLLTTGGTLK	Lys2	4.4	-3
LKB1	Serine/threonine-protein kinase 11 [Homo sapiens (Human)]	FPVCQAHGYFCQLIDGLEYLHSQGIVHkDIKPGNLLLTTGGTLK	Lys2	-8.8	25.7
LOK	Serine/threonine-protein kinase 10 [Homo sapiens (Human)]	NKETGALAAAkVIETK	Lys1	7.4	9.5
LOK	Serine/threonine-protein kinase 10 [Homo sapiens (Human)]	DLkAGNVLMTLEGDIR	Lys2	-9.3	-7.7
LYN	Tyrosine-protein kinase Lyn n=1 Tax=Homo sapiens RepID=LYN_HUMAN	VAVkTLKPGTMSVQAFLEEANLMK	Lys1	-9.1	-1.4
MAP2K1, MAP2K2	Dual specificity mitogen-activated protein kinase kinase 1 n=4 Tax=Eutheria RepID=MP2K1_HUMAN	DVkPSNILVNSR	Lys2	-17.1	-3.5
MAP2K3	Dual specificity mitogen-activated protein kinase kinase 3 [Homo sapiens (Human)]	MCDFGISGYLVDSVAkTMDAGCKPYMAPER	Activation Loop	0.7	12
MAP2K3	Dual specificity mitogen-activated protein kinase kinase 3 [Homo sapiens (Human)]	HAQSGTIMAVkR	Lys1	-4	-7.2
MAP2K3	Dual specificity mitogen-activated protein kinase kinase 3 [Homo sapiens (Human)]	DVkPSNVLINK	Lys2	-13.7	-10.5
MAP2K4	Dual specificity mitogen-activated protein kinase kinase 4 [Homo sapiens (Human)]	LCDFGISGQLVDSIAkTR	Activation Loop	-24.7	1.6
MAP2K4	Dual specificity mitogen-activated protein kinase kinase 4 [Homo sapiens (Human)]	MVHKPSGQIMAVkR	Lys1	-13	-16.1
MAP2K4	Dual specificity mitogen-activated protein kinase kinase 4 [Homo sapiens (Human)]	DIkPSNILLDR	Lys2	1.9	-5.2
MAP2K5	Dual specificity mitogen-activated protein kinase kinase 5 n=1 Tax=Homo sapiens RepID=MP2K5_HUMAN	DVkPSNMLVNTR	Lys2	6.7	-10.4
MAP2K6	Dual specificity mitogen-activated protein kinase kinase 6 [Homo sapiens (Human)]	MCDFGISGYLVDSVAkTIDAGCKPYMAPER	Activation Loop	2.4	3.6
MAP2K6	Dual specificity mitogen-activated protein kinase kinase 6 [Homo sapiens (Human)]	HVPSGQIMAVkR	Lys1	0.1	-8
MAP2K6	Dual specificity mitogen-activated protein kinase kinase 6 [Homo sapiens (Human)]	DVkPSNVLINALGQVK	Lys2	-11.4	-5.5
MAP3K2	Mitogen-activated protein kinase kinase kinase 2 n=3 Tax=Homo sapiens RepID=M3K2_HUMAN	ELAVkQVQFDPDSPETSKEVNALECEIQLLK	Lys1	-4.9	1.7
MAP3K2	Mitogen-activated protein kinase kinase kinase 2 n=3 Tax=Homo sapiens RepID=M3K2_HUMAN	VYLCYDVDTGRELAVkQVQFDPDSPETSKEVNALECEIQLLK	Lys1	3.1	13.4
MAP3K2, MAP3K3	Mitogen-activated protein kinase kinase kinase 2 n=3 Tax=Homo sapiens RepID=M3K2_HUMAN	DIkGANILR	Lys2	-11.3	-4.2
MAP3K3	Mitogen-activated protein kinase kinase kinase 3 n=2 Tax=Homo sapiens RepID=M3K3_HUMAN	ELASkQVQFDPDSPETSKEVSALECEIQLLK	Lys1	-12.9	-13.5
MAP3K4	Mitogen-activated protein kinase kinase kinase 4 [Homo sapiens (Human)]	VYTCISVDTGELMAMkEIR	Lys1	-6.6	1.4
MAP3K4	Mitogen-activated protein kinase kinase kinase 4 [Homo sapiens (Human)]	DIkGANIFLTSSGLIK	Lys2	-29.3	-7.7
MAP3K5	Mitogen-activated protein kinase kinase kinase 5 [Homo sapiens (Human)]	DIkGDNVLINTYSGVLK	Lys2	-7.6	-10.3
MAP3K5, MAP3K6, MAP3K7	Mitogen-activated protein kinase kinase kinase 5 [Homo sapiens (Human)]	IAIkEIPER	Lys1	-2.3	-2.8
MAP3K7,TAK1	Mitogen-activated protein kinase kinase kinase 7 [Homo sapiens (Human)]	DLkPPNLLLVAGGTVLK	Lys2	-6.3	-10.3
MAPKAPK	MAP kinase-activated protein kinase 3 [Homo	KYAVTDDYQLSkQVLGLGVNGK	ATP Loop	-7.4	-11.1

3	sapiens (Human)]				
MARK2	Serine/threonine-protein kinase MARK2 [Homo sapiens (Human)]	EVAVkIIDKTQLNSSSLQK	Lys1	-7.2	-11.2
MARK2,MARK3	MAP/microtubule affinity-regulating kinase 3 [Homo sapiens (Human)]	DLkAENLLLDADMNIK	Lys2	-26.2	-16.4
MARK3	MAP/microtubule affinity-regulating kinase 3 [Homo sapiens (Human)]	EVAIkIIDKTQLNPTSLQK	Lys1	-1	3
MARK3,MARK4	MAP/microtubule affinity-regulating kinase 3 [Homo sapiens (Human)]	EVAIkIIDK	Lys1	-5.4	-7.1
MAST1,MAST2	Microtubule-associated serine/threonine-protein kinase 2 [Homo sapiens (Human)]	DLkPDNLLITSMGHIK	Lys2	5	6.4
MAST2,MAST4	Microtubule-associated serine/threonine-protein kinase 2 [Homo sapiens (Human)]	DLkPDNLLVTSMGHIK	Lys2	-5.1	0.9
MAST3	Microtubule-associated serine/threonine-protein kinase 3 [Homo sapiens (Human)]	DLkPDNLLITSLGHIK	Lys2	-8.5	-6.1
MASTL	Microtubule-associated serine/threonine-protein kinase-like [Homo sapiens (Human)]	GAFGkVYLGQK	ATP Loop	-20.9	-27
MET	Hepatocyte growth factor receptor precursor [Homo sapiens (Human)]	DMYDkEYYSVHNK	Activation Loop	-27	-4.2
MET	Hepatocyte growth factor receptor precursor [Homo sapiens (Human)]	DMYDKEYYSVHNkTGAK	Activation Loop	-15	-7
MLK1	Mitogen-activated protein kinase kinase kinase 9 n=1 Tax=Homo sapiens RepID=M3K9_HUMAN	DLkSSNILILQK	Lys2	11.3	3.8
MLK2	Mitogen-activated protein kinase kinase kinase 10 n=1 Tax=Homo sapiens RepID=M3K10_HUMAN	DLkSINILILEAIENHNLADTVLK	Lys2	17.4	14.1
MLK3	Mitogen-activated protein kinase kinase kinase 11 [Homo sapiens (Human)]	EWHkTTQMSAAGTYAWMAPEVIK	Activation Loop	14.3	10.5
MLK3	Mitogen-activated protein kinase kinase kinase 11 [Homo sapiens (Human)]	DLkSNNILLLQPIESDDMEHK	Lys2	3.9	4.1
MLK4	Mitogen-activated protein kinase kinase kinase [Homo sapiens (Human)]	DLkSSNILLLEK	Lys2	14.6	-6
MLKL	CDNA FLJ34389 fis, clone HCHON2000087 [Homo sapiens (Human)]	APVAIkVFK	Lys1	-10.7	-9.3
MPSK1	Serine/threonine-protein kinase 16 n=1 Tax=Homo sapiens RepID=STK16_HUMAN	LGEGGFSYVDLVEGLHDGHFYALkR	Lys1	2.9	6
MPSK1	Serine/threonine-protein kinase 16 n=1 Tax=Homo sapiens RepID=STK16_HUMAN	DLkPTNILLGDEGQPVLMDLGSMNQACIHVEGSR	Lys2	-11.7	-1.7
MRCKb	Serine/threonine-protein kinase MRCK beta n=1 Tax=Homo sapiens RepID=MRCKB_HUMAN	DIkPDNVLLDVNGHIR	Lys2	-1.3	-0.5
MRCKb	Serine/threonine-protein kinase MRCK beta n=1 Tax=Homo sapiens RepID=MRCKB_HUMAN	NHHVHLYPWSSLDGAEGSFDIkLPETK	Other	1.2	5.1
MSK1 domain1	Ribosomal protein S6 kinase alpha 5 [Homo sapiens (Human)]	DIkLENILLDSNGHVVLTDFGLSK	Lys2	1	-0.8
MSK1 domain1	Ribosomal protein S6 kinase alpha 5 [Homo sapiens (Human)]	LGIIYRDIkLENILLDSNGHVVLTDFGLSK	Lys2	8.5	10.9
MSK1,MSK2 domain1	Ribosomal protein S6 kinase alpha 5 [Homo sapiens (Human)]	VLGTGAYGkVFLVR	ATP Loop	2.9	1.4
MSK2 domain1	Ribosomal protein S6 kinase alpha 4 [Homo sapiens (Human)]	DLkLENVLLDSEGHIVLTDFGLSK	Lys2	7.3	2.6
MST1	Serine/threonine-protein kinase 4 [Homo sapiens (Human)]	ETGQIVAIkQVPVESDLQEIIK	Lys1	6.2	1.2
MST1,MST2	Serine/threonine-protein kinase 4 [Homo sapiens (Human)]	DIkAGNILLNTEGHAK	Lys2	-3	-2.4
MST2	Serine/threonine-protein kinase 3 [Homo sapiens (Human)]	ESGQVVAIkQVPVESDLQEIIK	Lys1	-5.8	-3.6
MST2	Serine/threonine-protein kinase 3 [Homo sapiens (Human)]	ESGQVVAIkQVPVESDLQEIIKEISIMQQCDSPYVVK	Lys1	-32	-8.3
MST3	Serine/threonine-protein kinase 24 [Homo sapiens (Human)]	DIkAANVLLSEHGEVK	Lys2	-9.9	-7

MST4	Serine/threonine-protein kinase MST4 [Homo sapiens (Human)]	TQQVVAIkIIDLEEAEDEIEDIQQEITVLSQCDSSYVTK	Lys1	31.1	38.2
MST4,YSK1	Serine/threonine-protein kinase 25 [Homo sapiens (Human)]	DIkAANVLLSEQGDVK	Lys2	-30.2	-15.5
NDR1	Serine/threonine-protein kinase 38 [Homo sapiens (Human)]	DTGHVYAMkILR	Lys1	-9.4	-10.8
NDR2	Serine/threonine-protein kinase 38-like [Homo sapiens (Human)]	DTGHIYAMkILR	Lys1	4.2	-6.5
NDR2	Serine/threonine-protein kinase 38-like [Homo sapiens (Human)]	DIkPDNLLLDAK	Lys2	-11.8	-8
NEK1	Serine/threonine-protein kinase Nek1 [Homo sapiens (Human)]	DIkSQNIFLTK	Lys2	-13	-7.6
NEK3	Serine/threonine-protein kinase Nek3 [Homo sapiens (Human)]	SkNIFLTQNGK	Activation Loop	2.9	5.9
NEK4	Serine/threonine-protein kinase Nek4 [Homo sapiens (Human)]	DLkTQNVFLTR	Lys2	-7.4	-18.5
NEK6,NEK7	Serine/threonine-protein kinase Nek6 [Homo sapiens (Human)]	DIkPANVFITATGVVK	Lys2	6.1	-4.4
NEK7	Serine/threonine-protein kinase Nek7 [Homo sapiens (Human)]	AACLLDGVPVALkK	Lys1	-1	-6
NEK8	Serine/threonine-protein kinase Nek8 [Homo sapiens (Human)]	DLkTQNILLDK	Lys2	-12.2	-15.9
NEK9	Serine/threonine-protein kinase Nek9 n=1 Tax=Homo sapiens RepID=NEK9_HUMAN	LGDYGLAkK	Activation Loop	-22.1	-12.3
NEK9	Serine/threonine-protein kinase Nek9 n=1 Tax=Homo sapiens RepID=NEK9_HUMAN	TEDDSLVVWkEVDLTR	Lys1	-9.3	5
NEK9	Serine/threonine-protein kinase Nek9 n=1 Tax=Homo sapiens RepID=NEK9_HUMAN	DIkTLNIFLTK	Lys2	-5.8	1.1
OSR1	Serine/threonine-protein kinase OSR1 [Homo sapiens (Human)]	DVkAGNILLGEDGSVQIADFGVSAFLATGGDITR	Lys2	-6.6	-9
p38a	Mitogen-activated protein kinase 14 n=3 Tax=Eutheria RepID=MK14_HUMAN	DLkPSNLAVNEDCELK	Lys2	16.5	6.3
p38a	Mitogen-activated protein kinase 14 n=3 Tax=Eutheria RepID=MK14_HUMAN	QELNkTIWEVPER	Other	-2.1	-5.7
p38b	Mitogen-activated protein kinase 11 n=2 Tax=Homo sapiens RepID=MK11_HUMAN	QELNkTVWEVPQR	Other	-9.6	-9.7
p38d,p38g	Mitogen-activated protein kinase 12 [Homo sapiens (Human)]	DLkPGNLAVNEDCELK	Lys2	-11.1	2.8
p70S6K	Ribosomal protein S6 kinase 1 (EC 2.7.1.37) (S6K) (S6K1) (70 kDa ribosomal protein S6 kinase 1) (p70 S6 kinase alpha) (p70(S6K)-alpha) [Homo sapiens (Human)]	LTDFGLCkESIHDGTVTHTFCGTIEYMAPEILMR	Activation Loop	-15.5	-8.4
p70S6K	Ribosomal protein S6 kinase 1 (EC 2.7.1.37) (S6K) (S6K1) (70 kDa ribosomal protein S6 kinase 1) (p70 S6 kinase alpha) (p70(S6K)-alpha) [Homo sapiens (Human)]	DLkPENIMLNHQGHVK	Lys2	-6.9	-6.4
p70S6K,p70S6Kb	Ribosomal protein S6 kinase 1 (EC 2.7.1.37) (S6K) (S6K1) (70 kDa ribosomal protein S6 kinase 1) (p70 S6 kinase alpha) (p70(S6K)-alpha) [Homo sapiens (Human)]	GGYGkVFQVR	ATP Loop	-2.3	0.8
PCTAIRE1	Serine/threonine-protein kinase PCTAIRE-1 [Homo sapiens (Human)]	LTDNLVALkEIR	Lys1	-2.7	2.9
PCTAIRE2	Serine/threonine-protein kinase PCTAIRE-2 [Homo sapiens (Human)]	DLkPQNLLINEK	Lys2	-40.2	-12.4
PCTAIRE2, PCTAIRE3	Serine/threonine-protein kinase PCTAIRE-2 [Homo sapiens (Human)]	SKLTENLVALkEIR	Lys1	10.3	-1.8
PDHK1	[Pyruvate dehydrogenase [lipoamide]] kinase isozyme 1, mitochondrial precursor [Homo sapiens (Human)]	SPGQPIQVVYVPSHLYHMVFELFkNAMR	Other	31.4	16.9
PDK1	3-phosphoinositide-dependent protein kinase 1 [Homo sapiens (Human)]	EYAIkILEK	Lys1	-5.3	-11.8
PEK	Eukaryotic translation initiation factor 2-alpha kinase 3 precursor [Homo sapiens (Human)]	DLkPSNIFFTMDDVVK	Lys2	-7.4	-3.9
PFTAIRE1	Serine/threonine-protein kinase PFTAIRE-1	LVALkVIR	Lys1	8.2	-4.9

	n=1 Tax=Homo sapiens RepID=PFTK1_HUMAN				
PFTAIRE1	Serine/threonine-protein kinase PFTAIRE-1 n=1 Tax=Homo sapiens RepID=PFTK1_HUMAN	DLkPQNLLISDTGELK	Lys2	-0.6	-6.5
PHKg1	Phosphorylase b kinase gamma catalytic chain, skeletal muscle isoform n=2 Tax=Homo sapiens RepID=PHKG1_HUMAN	DLkPENILLDDNMNIK	Lys2	6.1	2
PHKg2	Phosphorylase b kinase gamma catalytic chain, testis/liver isoform [Homo sapiens (Human)]	ATGHEFAVkIMEVTAER	Lys1	4.6	-2.7
PI4KA,PI4 KAP2	Phosphatidylinositol 4-kinase alpha n=2 Tax=Homo sapiens RepID=PI4KA_HUMAN	SGTPMQSAAkAPYLAK	ATP	-7.4	-3.1
PI4KB	Phosphatidylinositol 4-kinase beta n=2 Tax=Homo sapiens RepID=PI4KB_HUMAN	VPHTQAVVLNSkDK	ATP	2	-16.5
PI4KB	Phosphatidylinositol 4-kinase beta n=2 Tax=Homo sapiens RepID=PI4KB_HUMAN	VPHTQAVVLNSkDKAPYLIYVEVLECENFD TTSVPAR	ATP	9.8	-5.8
PIK3C2B	Phosphatidylinositol-4-phosphate 3-kinase C2 domain-containing beta polypeptide [Homo sapiens]	VIFkCGDDLRQDMLTLQMIR	ATP	52.1	40.3
PIK3C3	Phosphatidylinositol 3-kinase catalytic subunit type 3 [Homo sapiens (Human)]	TEDGGKYPVIFkHGDDLR	ATP	1.8	-16.5
PIK3C3	Phosphatidylinositol 3-kinase catalytic subunit type 3 [Homo sapiens (Human)]	TEDGGKYPVIFkHGDDLRQDQLILQIISLM DK	ATP	13.7	14.3
PIK3CA	Phosphatidylinositol-4,5-bisphosphate 3-kinase catalytic subunit alpha isoform n=2 Tax=Homo sapiens RepID=PK3CA_HUMAN	RPLWLNWENPDIMSELLFQNNEIIFkNGD DLRQDMLTLQIIR	ATP	5.6	28.5
PIK3CB	Phosphatidylinositol-4,5-bisphosphate 3-kinase catalytic subunit beta isoform [Homo sapiens (Human)]	VFGEDSVGVIFkNGDDLR	ATP	5.6	-9
PIK3CB	Phosphatidylinositol-4,5-bisphosphate 3-kinase catalytic subunit beta isoform [Homo sapiens (Human)]	VFGEDSVGVIFkNGDDLRQDMLTLQMLR	ATP	0.9	6.7
PIP4K2A	Phosphatidylinositol-4-phosphate 5-kinase type II alpha (EC 2.7.1.68) (1-phosphatidylinositol-4-phosphate 5-kinase 2-alpha) (PtdIns(4)P-5- kinase isoform 2-alpha) (PIP5KII-alpha) (Diphosphoinositide kinase 2-alpha) (PtdIns(4)P-5-kinase B isoform) (PIP5KI	AKELPTLkDNDFINEGQK	ATP	0.5	-4.5
PIP4K2B	Phosphatidylinositol-4-phosphate 5-kinase type II beta (EC 2.7.1.68) (1-phosphatidylinositol-4-phosphate 5-kinase 2-beta) (PtdIns(4)P-5- kinase isoform 2-beta) [Homo sapiens (Human)]	DLPTFkDNDFLNEGQK	ATP	-4.7	15.9
PIP4K2C	Phosphatidylinositol-5-phosphate 4-kinase type-2 gamma n=1 Tax=Homo sapiens RepID=PI42C_HUMAN	ELPTLkDMDFLNK	ATP	-8.2	-6.7
PIP4K2C	Phosphatidylinositol-5-phosphate 4-kinase type-2 gamma n=1 Tax=Homo sapiens RepID=PI42C_HUMAN	TLVIkEVSSEDIADMHSNLSNYHQYIVK	ATP	-9.4	-10.9
PIP4K2C	Phosphatidylinositol-5-phosphate 4-kinase type-2 gamma n=1 Tax=Homo sapiens RepID=PI42C_HUMAN	VKELPTLkDMDFLNK	ATP	16	4.2
PIP5K3	FYVE finger-containing phosphoinositide kinase (EC 2.7.1.68) (1- phosphatidylinositol-4-phosphate 5-kinase) (Phosphatidylinositol-3- phosphate 5-kinase type III) (PIP5K) (PtdIns(4)P-5-kinase) [Homo sapiens (Human)]	GGkSGAAFYATEDDRFILK	ATP	96.6	-7.7
PITSLRE	PITSLRE serine/threonine-protein kinase CDC2L1 [Homo sapiens (Human)]	DLkTSNLLLSHAGILK	Lys2	4.6	-1.1
PKCa,PKC b	Protein kinase C alpha type n=2 Tax=Homo sapiens RepID=KPCA_HUMAN	DLkLDNVMLDSEGHIK	Lys2	-0.1	-1.9
PKCi	Protein kinase C iota type [Homo sapiens (Human)]	IYAMkVVK	Lys1	-13.3	-0.4
PKCz	Protein kinase C zeta type [Homo sapiens (Human)]	NDQIYAMkVVK	Lys1	19.7	17.3
PKD1	Serine/threonine-protein kinase D1 n=1	DVAIkIIDK	Lys1	-20.7	-8.8

	Tax=Homo sapiens RepID=KPCD1_HUMAN				
PKD1,PKD2	Serine/threonine-protein kinase D1 n=1 Tax=Homo sapiens RepID=KPCD1_HUMAN	NIVHCDLkPENVLLASADPFPQVK	Lys2	-10.6	5.2
PKD2	Protein kinase D2 [Homo sapiens (Human)]	DVAVkVIDK	Lys1	-4.3	-7.2
PKD3	Protein kinase D3 [Homo sapiens (Human)]	NIVHCDLkPENVLLASAEPFPQVK	Lys2	-8.9	2.8
PKN1	Protein kinase N1 [Homo sapiens (Human)]	VLLSEFRPSGELFAIkALK	Lys1	0.9	1.6
PKN2	Protein kinase N2 [Homo sapiens (Human)]	DLkLDNLLLDTEGFVK	Lys2	-3.6	9.1
PKR	Interferon-induced, double-stranded RNA-activated protein kinase [Homo sapiens (Human)]	DLkPSNIFLVDTK	Lys2	-0.3	-5.4
PLK1	Serine/threonine-protein kinase PLK1 [Homo sapiens (Human)]	CFEISDADTKEVFAGkIVPK	Lys1	-11.4	-1.5
PLK1	Serine/threonine-protein kinase PLK1 [Homo sapiens (Human)]	DLkLGNLFLNEDLEVK	Lys2	-15.2	-7.2
PRP4	Serine/threonine-protein kinase PRP4 homolog [Homo sapiens (Human)]	CNILHADIkPDNILVNESK	Lys2	-1.8	-6.7
PRPK	TP53-regulating kinase [Homo sapiens (Human)]	FLSGLELVkQGAEAR	ATP Loop	3.2	0.3
RIPK2	Receptor-interacting serine/threonine-protein kinase 2 [Homo sapiens (Human)]	VQVAVkHLHIHTPLLDSER	Lys1	7.6	0
RIPK2	Receptor-interacting serine/threonine-protein kinase 2 [Homo sapiens (Human)]	ILHEIALGVNYLHNMTPPLLHHDLkTQNILLDNEFHVK	Lys2	8.8	5.3
ROCK1	Rho-associated protein kinase 1 [Homo sapiens (Human)]	kLQLELNQER	Other	2.2	3.9
ROCK1,ROCK2	Rho-associated protein kinase 2 [Homo sapiens (Human)]	DVkPDNMLLDK	Lys2	-22.5	-16.1
RON	Macrophage-stimulating protein receptor precursor (EC 2.7.1.112) (MSP receptor) (p185-Ron) (CDw136 antigen) [Contains: Macrophage- stimulating protein receptor alpha chain; Macrophage-stimulating protein receptor beta chain] [Homo sapiens (Human)]	IQCAIkSLSR	Lys1	-13.2	-10.5
RSK1 domain2	Ribosomal protein S6 kinase alpha 1 [Homo sapiens (Human)]	DLkPSNILYVDESGNPECLR	Lys2	1	-6.9
RSK1,RSK2 domain1	Ribosomal protein S6 kinase alpha 1 [Homo sapiens (Human)]	DLkPENILLDEEGHIKLTDFGLSK	Lys2	-1	2.1
RSK1,RSK2,RSK3 domain1	Ribosomal protein S6 kinase alpha 1 [Homo sapiens (Human)]	DLkPENILLDEEGHIK	Lys2	8.4	-2
RSK1,RSK3	Ribosomal protein S6 kinase alpha 1 [Homo sapiens (Human)]	SkRDPSEEIEILLR	Other	2.1	1.5
RSK1,RSK4 domain1	Ribosomal protein S6 kinase alpha 1 [Homo sapiens (Human)]	VLGQGSFGkVFLVR	ATP Loop	9	-10.3
RSK2 domain1	Ribosomal protein S6 kinase alpha 3 [Homo sapiens (Human)]	VLGQGSFGkVFLVK	ATP Loop	34.2	3.3
RSK2 domain2	Ribosomal protein S6 kinase alpha 3 [Homo sapiens (Human)]	DLkPSNILYVDESGNPESIR	Lys2	-3.8	-9.6
RSK2 domain2	Ribosomal protein S6 kinase alpha 3 [Homo sapiens (Human)]	SkRDPTEEIEILLR	Protein Kinase Domain	13.3	8.5
RSK4 domain1	Ribosomal protein S6 kinase alpha 6 [Homo sapiens (Human)]	DLkPENILLDEIGHIK	Lys2	52.8	0.9
RSKL1 domain1	Ribosomal protein S6 kinase delta-1 n=2 Tax=Homo sapiens RepID=KS6C1_HUMAN	VLGVIDkVLLVMDTR	Protein Kinase Domain	1.1	-2.6
SGK3	Serine/threonine-protein kinase Sgk3 [Homo sapiens (Human)]	FYAVkVLQK	Lys1	-7.6	-9
SLK	CTCL tumor antigen se20-9 [Homo sapiens (Human)]	ETSVLAAAkVIDTK	Lys1	-11.1	-1.8
SLK	CTCL tumor antigen se20-9 [Homo sapiens (Human)]	DLkAGNILFTLDGDIK	Lys2	-9.2	-8.1
SMG1	Serine/threonine-protein kinase SMG1 n=4 Tax=Homo sapiens RepID=SMG1_HUMAN	DTVTIHSVGGTITILPTKTkPK	ATP	-5.7	-7.5
SNRK	SNF-related serine/threonine-protein kinase	DLkPENVVFFEK	Lys2	1.2	0.3

	[Homo sapiens (Human)]				
SRC	Proto-oncogene tyrosine-protein kinase Src n=1 Tax=Homo sapiens RepID=SRC_HUMAN	VAIkTLKPGTMSPEAFLQEAQVMK	Lys1	-15.5	-13.5
SRPK1	Serine/threonine-protein kinase SRPK1 n=1 Tax=Homo sapiens RepID=SRPK1_HUMAN	IIHTDIkPENILLSVNEQYIR	Lys2	5.7	4.3
SRPK1,SRPK2	Serine/threonine-protein kinase SRPK1 n=1 Tax=Homo sapiens RepID=SRPK1_HUMAN	FVAMkVVK	Lys1	-61.9	-6.3
STLK5	STE20-related adaptor protein [Homo sapiens (Human)]	YSVkVLPWLSPEVLQQNLQGYDAK	Activation Loop	5.3	10.7
STLK5	STE20-related adaptor protein [Homo sapiens (Human)]	SVkASHILISVDGK	Lys2	-32.7	-5.5
STLK6	Serine/threonine-protein kinase ALS2CR2 [Homo sapiens (Human)]	HTPTGTLVTIkITNLENCNEER	Lys1	0.6	-13.2
STLK6	Serine/threonine-protein kinase ALS2CR2 [Homo sapiens (Human)]	SIkASHILISGDGLVTLSGLSHLHSLVK	Lys2	-11.4	4
TAO1,TAO3	Serine/threonine-protein kinase TAO3 [Homo sapiens (Human)]	DIkAGNILLTEPGQVK	Lys2	-7	-11.1
TAO2	Serine/threonine-protein kinase TAO2 n=2 Tax=Homo sapiens RepID=TAOK2_HUMAN	DVkAGNILLSEPGLVK	Lys2	-6.8	-2.6
TBK1	Serine/threonine-protein kinase TBK1 [Homo sapiens (Human)]	TGDLFAIkVFNNISFLRPVDVQMR	Lys1	-11.4	3.3
TLK1	Serine/threonine-protein kinase tousled-like 1 [Homo sapiens (Human)]	YAAVkIHQLNK	Lys1	-28.7	-24.9
TLK1,TLK2	Serine/threonine-protein kinase tousled-like 1 [Homo sapiens (Human)]	YLNEIKPPIIHYDLkPGNILLVDGTACGEIK	Lys2	0.3	-4.7
TLK2	Serine/threonine-protein kinase tousled-like 2 [Homo sapiens (Human)]	YVAVkIHQLNK	Lys1	-7.5	-6.8
ULK1	Serine/threonine-protein kinase ULK1 [Homo sapiens]	DLkPQNILLSNPAGR	Lys2	-8.3	-9
ULK3	Unc-51-like kinase 3 [Homo sapiens (Human)]	EVVAIKCVAk	Lys1	-11.6	0.5
ULK3	Unc-51-like kinase 3 [Homo sapiens (Human)]	NISHLDLkPQNILLSSLEKPHLK	Lys2	-11.5	9.4
Wnk1,Wnk2,Wnk3	Serine/threonine-protein kinase WNK2 [Homo sapiens (Human)]	DLkCDNIFITGPTGSVK	Lys2	7.4	-9
Wnk1,Wnk2,Wnk4	Serine/threonine-protein kinase WNK2 [Homo sapiens (Human)]	IGDLGLATLkR	Activation Loop	-5.2	-12.8
Wnk4	Serine/threonine-protein kinase WNK4 [Homo sapiens (Human)]	DLkCDNVFITGPTGSVK	Lys2	-16.7	-7.1
YANK3	PKE protein kinase [Homo sapiens (Human)]	DVkPDNILLDER	Lys2	13.6	-7.6
YES	Proto-oncogene tyrosine-protein kinase Yes n=1 Tax=Homo sapiens RepID=YES_HUMAN	VAIkTLKPGTMMPEAFLQEAQIMK	Lys1	-2.4	-4.3
YSK1	Serine/threonine-protein kinase 25 [Homo sapiens (Human)]	EVVAIkIIDLEEAEDEIEDIQQEITVLSQCDSPYITR	Lys1	3.5	34.6
ZAK	Mitogen-activated protein kinase kinase kinase MLT [Homo sapiens (Human)]	WISQDKEVAVKk	Lys1	-3.3	-6.3
ZC1/HGK	Mitogen-activated protein kinase kinase kinase kinase 4 [Homo sapiens (Human)]	TGQLAAIkVMDVTEDEEEEIKLEINMLK	Lys1	17.4	43.7
ZC1/HGK	Mitogen-activated protein kinase kinase kinase kinase 4 [Homo sapiens (Human)]	TGQLAAIkVMDVTEDEEEEIKLEINMLKK	Lys1	11.1	-8.3
ZC1/HGK,ZC2/TNIK,ZC3/MINK	Mitogen-activated protein kinase kinase kinase kinase 4 [Homo sapiens (Human)]	DIkGQNVLLTENAEVK	Lys2	-11.4	-15.8
ZC2/TNIK	TRAF2 and NCK-interacting kinase [Homo sapiens (Human)]	TGQLAAIkVMDVTGDEEEEIKQEINMLKK	Lys1	3.5	-8

General experimental section

All reagents were used as received. Acetonitrile, DCM, toluene and THF were purified using the Innovative Technology PureSolv solvent purification system. The ^{1}H and ^{13}C spectra were recorded on a Bruker Avance 400 or 500 MHz spectrometer. Chemical shifts are reported in parts per million and were referenced to residual proton solvent signals. Flash column chromatography separations were performed

using the Teledyne Isco CombiFlash Rf using RediSep Rf silica gel columns. TLC was performed on Analtech UNIPLATE silica gel GHLF plates (gypsum inorganic hard layer with fluorescence). TLC plates were developed using iodine vapor. Automated preparative RP HPLC purification was performed using an Agilent 1200 Mass-Directed Fractionation system (Prep Pump G1361 with gradient extension, Make-up pump G1311A, pH modification pump G1311A, HTS PAL autosampler, UV-DAD detection G1315D, Fraction Collector G1364B, and Agilent 6120 quadrapole spectrometer G6120A). The preparative chromatography conditions included a Waters X-Bridge C18 column (19 × 150 mm, 5 um, with 19 × 10-mm guard column), elution with a water and acetonitrile gradient, which increases 20% in acetonitrile content over 4 min at a flow rate of 20 mL/min (modified to pH 9.8 through addition of NH_4OH by auxiliary pump), and sample dilution in DMSO. The preparative gradient, triggering thresholds, and UV wavelength were selected according to the analytical RP HPLC analysis of each crude sample. The analytical method used an Agilent 1200 RRLC system with UV detection (Agilent 1200 DAD SL) and mass detection (Agilent 6224 TOF). The analytical method conditions included a Waters Aquity BEH C18 column (2.1 × 50 mm, 1.7 um) and elution with a linear gradient of 5% acetonitrile in pH 9.8 buffered aqueous ammonium formate to 100% acetonitrile at 0.4 mL/min flow rate. Compound purity was measured on the basis of peak integration (area under the curve) from UV/vis absorbance (at 214 nm), and compound identity was determined on the basis of mass analysis.

Compounds S1-S44 and S57-S69 were purchased from commercial vendors. All synthetic compounds were synthesized by one of four general synthetic methods (A to D) described below. The yield, purity and characterization of compounds synthesized by each method are compiled immediately following each method.

General Procedure A: Representative protocol for the synthesis of quinazoline analogues, synthesis and characterization for **16**, S45-S56, S70-S125, S142-S147, S156-S160, ML241, S162, S165, S169-S188.

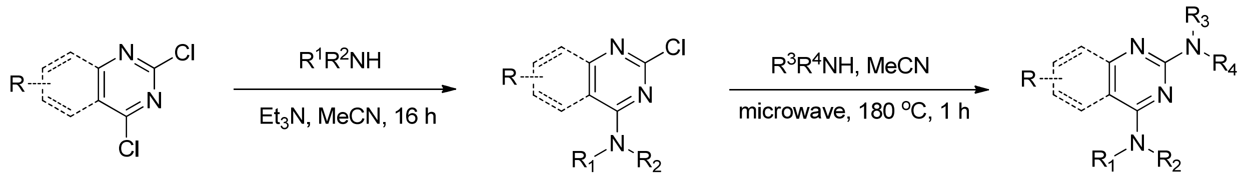

Scheme S1: General synthesis of 2,4-diaminoquinazolines

Representative procedure for the synthesis of quinazoline analogues:

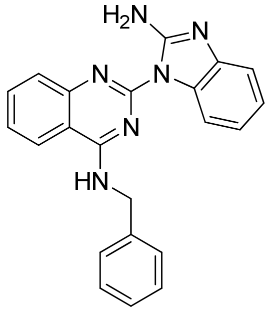

KSC-206-026

***N*-Benzyl-2-chloroquinazolin-4-amine**. 2,4-dichloroquinazoline (2.4 g, 12.3 mmol) was suspended in 20 mL of THF. Triethylamine (2.1 mL, 14.7 mmol) was added, followed by the addition of benzylamine (1.4 mL, 12.9 mmol). The mixture was stirred at room temperature for 16 h. The mixture was diluted with EtOAc, filtered, and the filtrate was concentrated. The residue was purified by silica gel chromatography (EtOAc:hexanes = 1:3, Rf = 0.5) to give a colorless solid (1.6 g, 47%). ^{1}H NMR (400MHz, $CDCl_3$) δ 7.87–7.73 (m, 2H), 7.68 (d, *J* = 8.2 Hz, 1H), 7.54–7.32 (m, 6H), 6.10 (s, 1H), 4.90 (d, *J* = 5.3 Hz, 2H).

2-(2-Amino-*1H*-benzo[*d*]imidazol-1-yl)-*N*-benzylquinazolin-4-amine (16). A suspension of *N*-benzyl-2-chloroquinazolin-4-amine (0.015 g, 0.056 mmol) and *1H*-benzo[d]imidazol-2-amine (0.015 g, 0.111 mmol) in Acetonitrile (1 ml) was heated in microwave at 180 °C for 1 h. Solvent was removed and the crude sample was purified via reverse phase preparative HPLC. Yield: 6.8 mg, 33%.^{1}H NMR (400 MHz, DMSO) δ 9.42 (t, *J* = 5.8 Hz, 1H), 8.41 (d, *J* = 8.1 Hz, 1H), 8.12 (d, *J* = 7.5 Hz, 1H), 7.94 – 7.79 (m, 4H), 7.54 – 7.50 (m, 1H), 7.45 (d, *J* = 7.1 Hz, 2H), 7.37 (t, *J* = 7.6 Hz, 2H), 7.27 (t, *J* = 7.3 Hz, 1H), 7.15 (d, *J* = 7.1 Hz, 1H), 7.03 (td, *J* = 1.2, 7.6 Hz, 1H), 6.88 – 6.76 (m, 1H), 4.91 (d, *J* = 5.8 Hz, 2H). ^{13}C NMR (126 MHz, DMSO) δ 160.7, 154.5, 154.1, 148.9, 142.8, 138.4, 133.7, 131.7, 128.5, 127.0, 126.6, 125.1, 123.0, 122.5, 118.7, 114.8, 112.5, 44.4. HRMS (m/z): calcd for $C_{22}H_{19}N_6$ (M+H) 367.1671; found 367.1685.

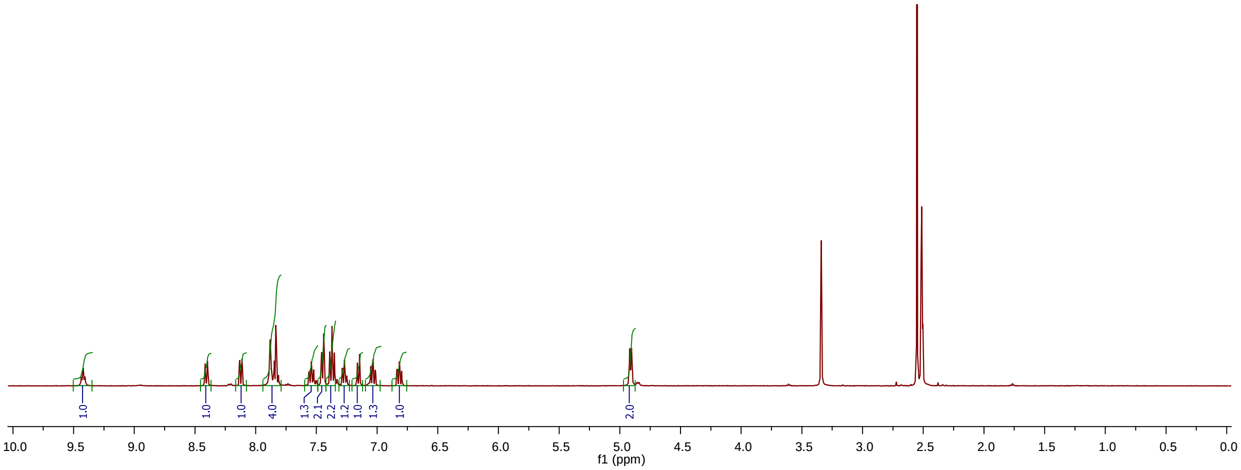

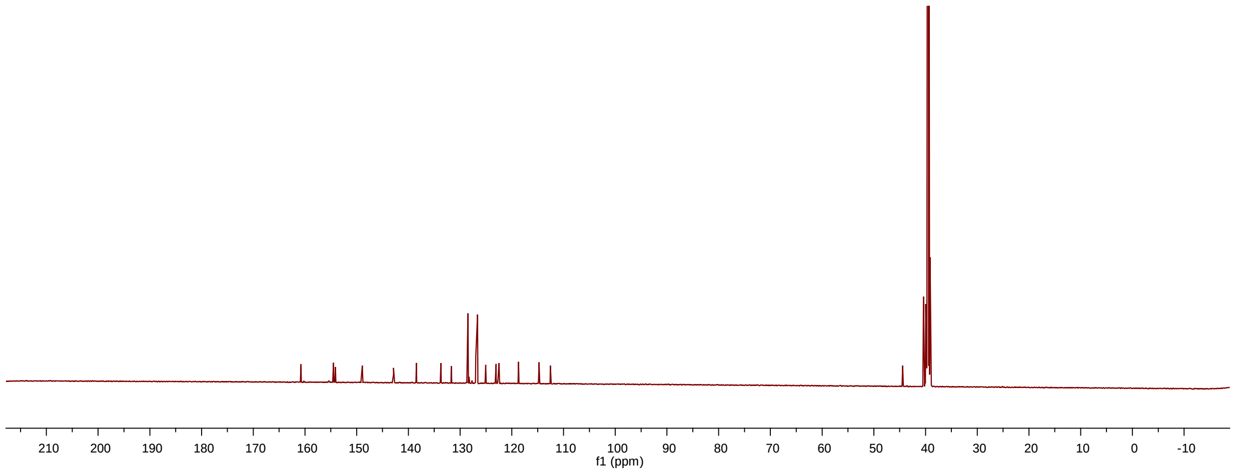

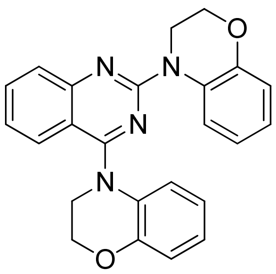

KSC-16-190

4,4'-(Quinazoline-2,4-diyl)bis(3,4-dihydro-*2H*-benzo[*b*][1,4]oxazine) (S45). Yield: 10.4 mg, 33%. ^{1}H NMR (400 MHz, $CDCl_3$) δ 8.03 (dd, *J* = 1.5, 8.2 Hz, 1H), 7.66 (dd, *J* = 0.8, 8.4 Hz, 1H), 7.59 (dd, *J* = 0.8, 8.5 Hz, 1H), 7.53 (ddd, *J* = 1.4, 6.7, 8.4 Hz, 1H), 7.00 (ddd, *J* = 1.4, 6.7, 8.2 Hz, 1H), 6.96 – 6.75 (m, 5H), 6.71 (d, *J* = 8.1 Hz, 1H), 6.67 – 6.53 (m, 1H), 4.48 – 4.36 (m, 2H), 4.36 – 4.22 (m, 4H), 4.07 – 3.95 (m, 2H). ^{13}C NMR (101 MHz, $CDCl_3$) δ 161.4, 156.4, 154.0, 146.4, 145.7, 133.3, 129.7, 127.7, 127.0, 126.2, 124.3, 124.0, 123.8, 122.2, 120.4, 120.0, 119.5, 117.3, 117.0, 113.7, 66.5, 66.1, 45.6, 42.3. HRMS (m/z): calcd for $C_{24}H_{21}N_4O_2$ (M+H) 397.1665; found 397.1664.

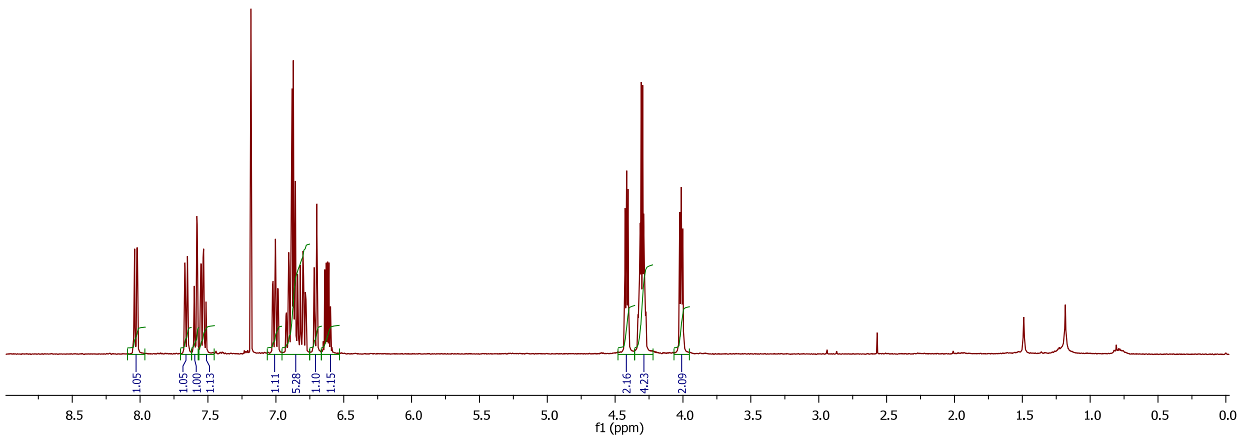

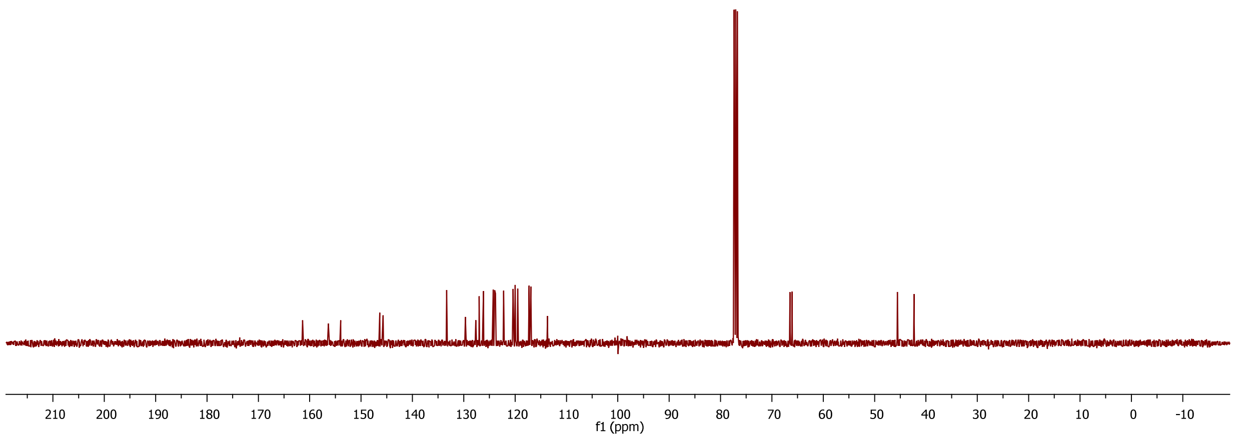

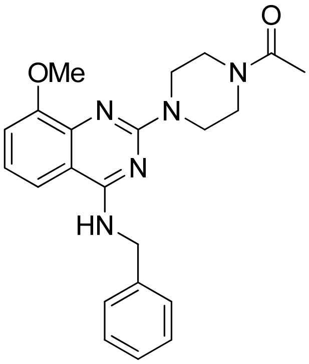

KSC-16-272

1-(4-(4-(Benzylamino)-8-methoxyquinazolin-2-yl)piperazin-1-yl)ethanone (S46). Yield: 20.6 mg, 79%. ^{1}H NMR (400 MHz, $CDCl_3$) δ 7.43 – 7.34 (m, 4H), 7.34 – 7.29 (m, 1H), 7.16 (dd, *J* = 1.3, 8.2 Hz, 1H), 7.05 (t, *J* = 7.9 Hz, 1H), 6.99 (dd, *J* = 1.2, 7.8 Hz, 1H), 5.97 (t, *J* = 5.3 Hz, 1H), 4.81 (d, *J* = 5.4 Hz, 2H), 3.99 (s, 3H), 3.98 – 3.94 (m, 2H), 3.91 (dd, *J* = 4.3, 6.3 Hz, 2H), 3.74 – 3.63 (m, 2H), 3.58 – 3.46 (m, 2H), 2.15 (s, 3H). ^{13}C NMR (101 MHz, $CDCl_3$) δ 169.2, 159.8, 158.4, 153.4, 143.9, 138.7, 128.7, 127.8, 127.5, 121.0, 112.6, 111.2, 111.0, 56.1, 46.4, 45.4, 44.0, 43.8, 41.6, 21.5. HRMS (m/z): calcd for $C_{22}H_{26}N_5O_2$ (M+H) 392.2087; found 392.2084.

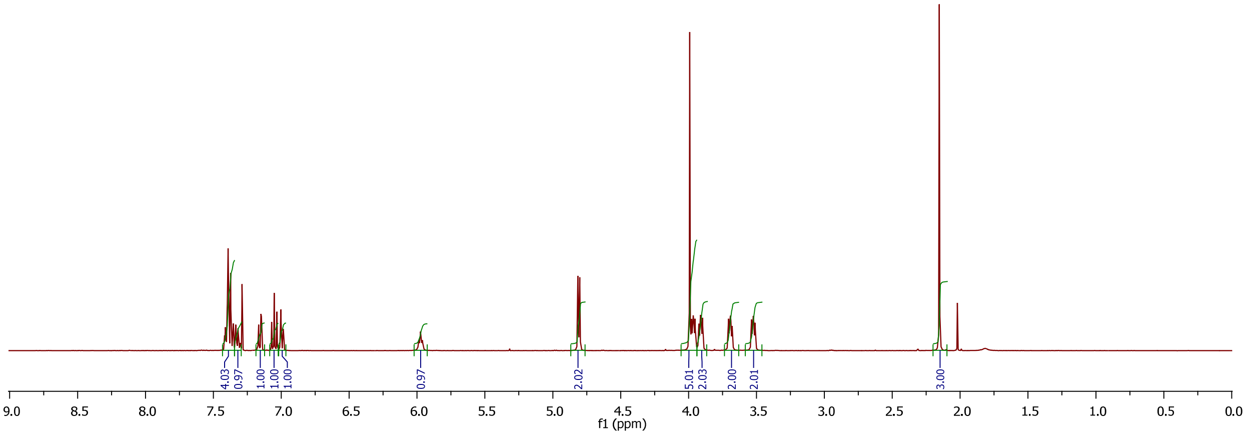

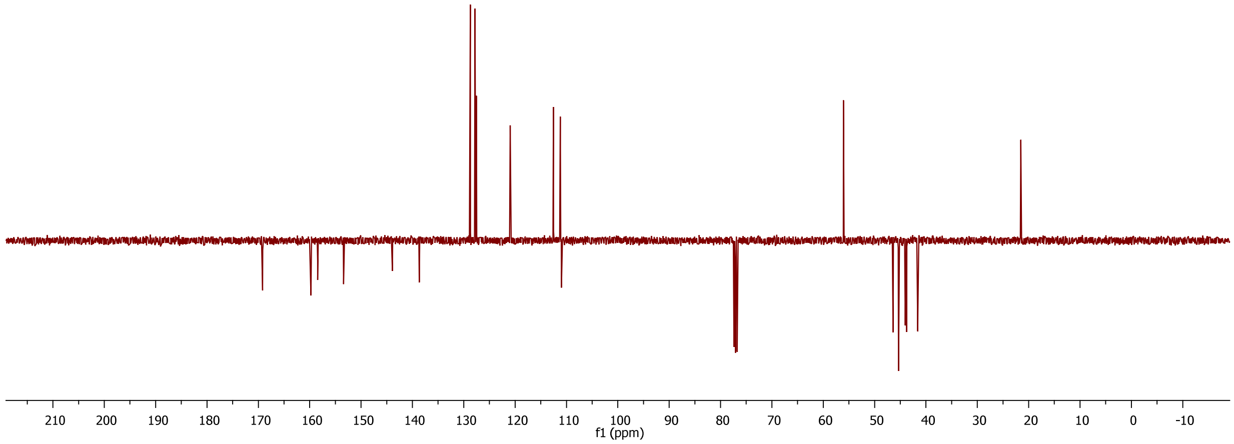

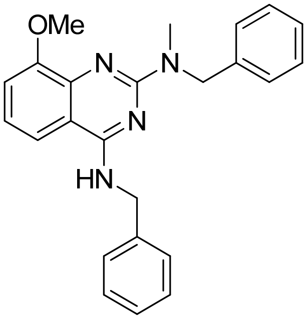

KSC-16-277

***N*²,*N*⁴-Dibenzyl-8-methoxy-*N*²-methylquinazoline-2,4-diamine (S47)**. Yield: 21.8 mg, 85%. ^{1}H NMR (400 MHz, $CDCl_3$) δ 7.40 – 7.19 (m, 10H), 7.12 (dd, *J* = 2.0, 7.3 Hz, 1H), 7.07 – 6.93 (m, 2H), 5.81 (s, 1H), 5.02 (s, 2H), 4.75 (d, *J* = 5.5 Hz, 2H), 4.00 (s, 3H), 3.28 (s, 3H). ^{13}C NMR (101 MHz, $CDCl_3$) δ 159.6, 159.1, 153.4, 139.4, 138.9, 128.7, 128.3, 127.9, 127.5, 127.4, 126.7, 120.1, 112.5, 111.1, 110.6, 56.1, 52.5, 45.2, 34.7. HRMS (m/z): calcd for $C_{24}H_{25}N_4O$ (M+H) 385.2028; found 385.2024.

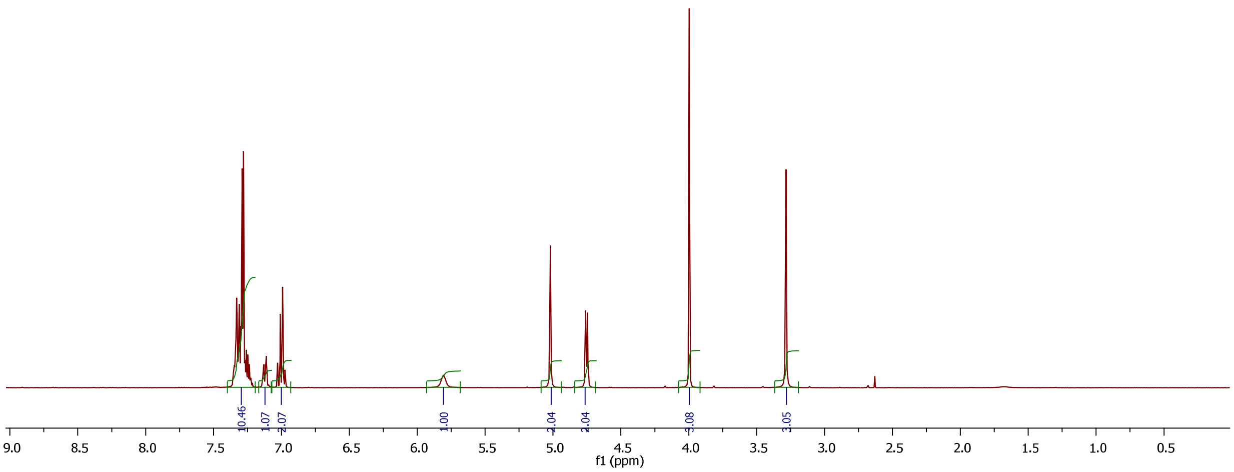

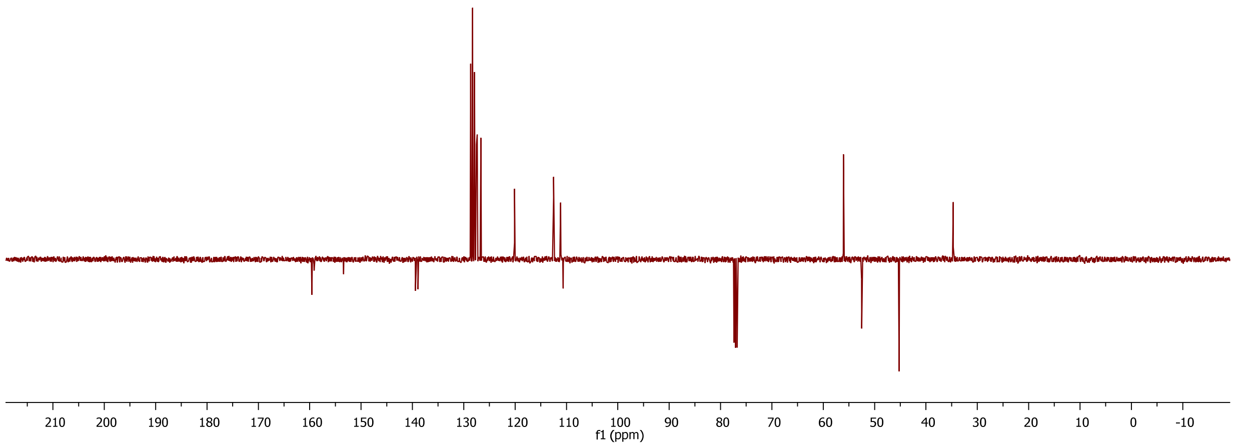

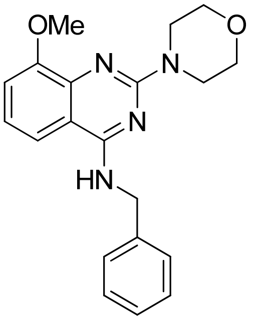

KSC-25-10

***N*-Benzyl-8-methoxy-2-morpholinoquinazolin-4-amine (S48)**. Yield: 14.2 mg, 81%. ^{1}H NMR (400 MHz, DMSO) δ 8.52 (t, *J* = 5.7 Hz, 1H), 7.63 (dd, *J* = 1.9, 7.6 Hz, 1H), 7.41 – 7.35 (m, 2H), 7.35 – 7.28 (m, 2H), 7.26 – 7.18 (m, 1H), 7.08 – 6.98 (m, 2H), 4.70 (d, *J* = 5.8 Hz, 2H), 3.83 (s, 3H), 3.74 – 3.66 (m, 4H), 3.64 – 3.54 (m, 4H). ^{13}C NMR (101 MHz, DMSO) δ 159.7, 157.9, 152.9, 143.4, 140.0, 128.2, 127.3, 126.6, 120.3, 114.2, 112.0, 111.0, 66.1, 55.5, 44.2, 43.7. HRMS (m/z): calcd for $C_{20}H_{23}N_4O_2$ (M+H) 351.1821; found 351.1814.

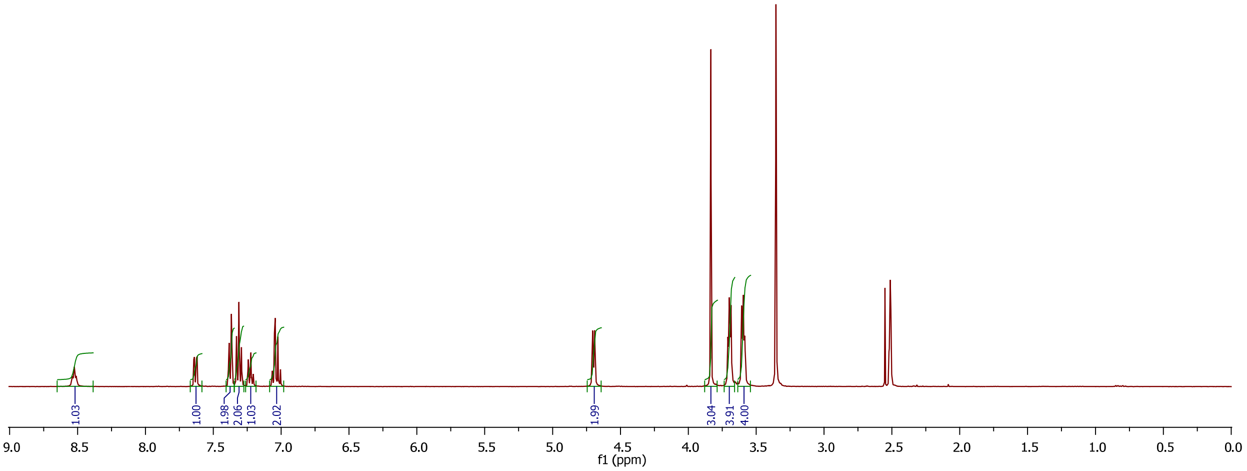

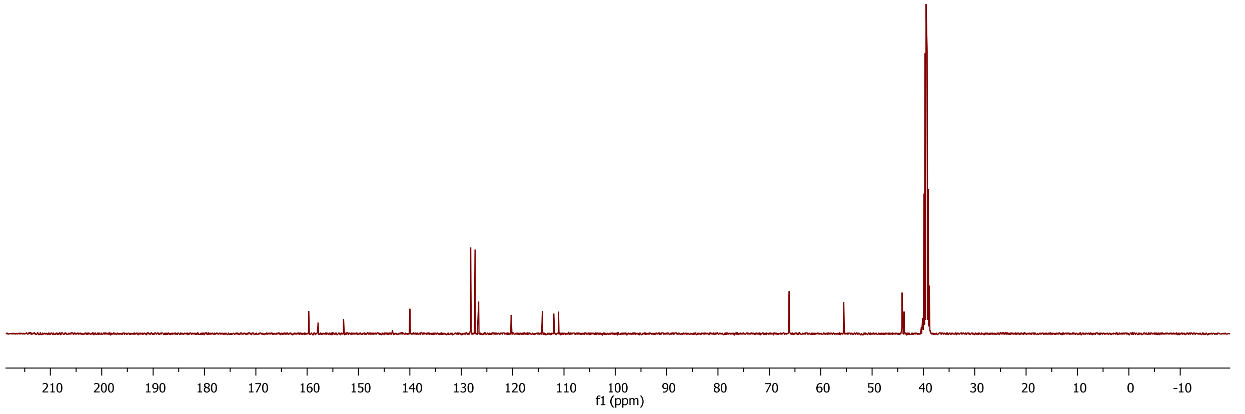

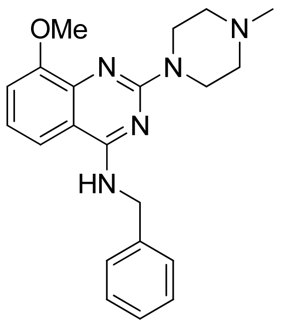

KSC-25-16

***N*-Benzyl-8-methoxy-2-(4-methylpiperazin-1-yl)quinazolin-4-amine (S49)**. Yield: 12.0 mg, 66%. ^{1}H NMR (500 MHz, DMSO) δ 8.47 (t, *J* = 5.9 Hz, 1H), 7.60 (dd, *J* = 1.4, 8.0 Hz, 1H), 7.37 (d, *J* = 7.1 Hz, 2H), 7.30 (dd, *J* = 4.8, 10.3 Hz, 2H), 7.21 (t, *J* = 7.3 Hz, 1H), 7.03 (dd, *J* = 1.3, 7.8 Hz, 1H), 6.99 (t, *J* = 7.8 Hz, 1H), 4.68 (d, *J* = 5.8 Hz, 2H), 3.82 (s, 3H), 3.71 (s, 4H), 2.27 (t, *J* = 4.9 Hz, 4H), 2.17 (s, 3H). ^{13}C NMR (126 MHz, DMSO) δ 159.6, 157.8, 152.8, 143.6, 140.1, 128.1, 127.3, 126.6, 120.0, 114.1, 111.8, 110.8, 55.5, 54.6, 45.9, 43.7, 43.3. HRMS (m/z): calcd for $C_{21}H_{26}N_5O$ (M+H) 364.2137; found 364.2142.

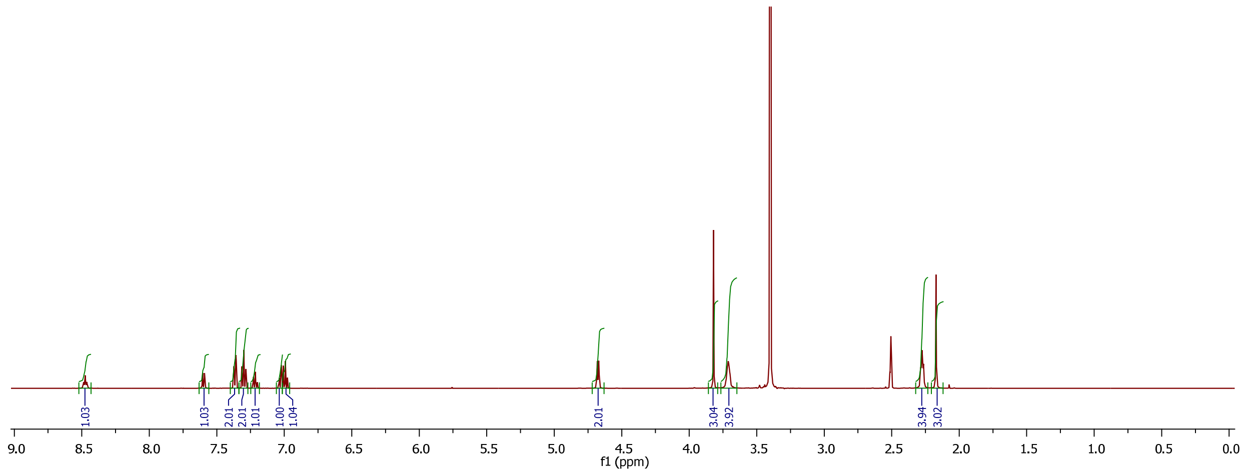

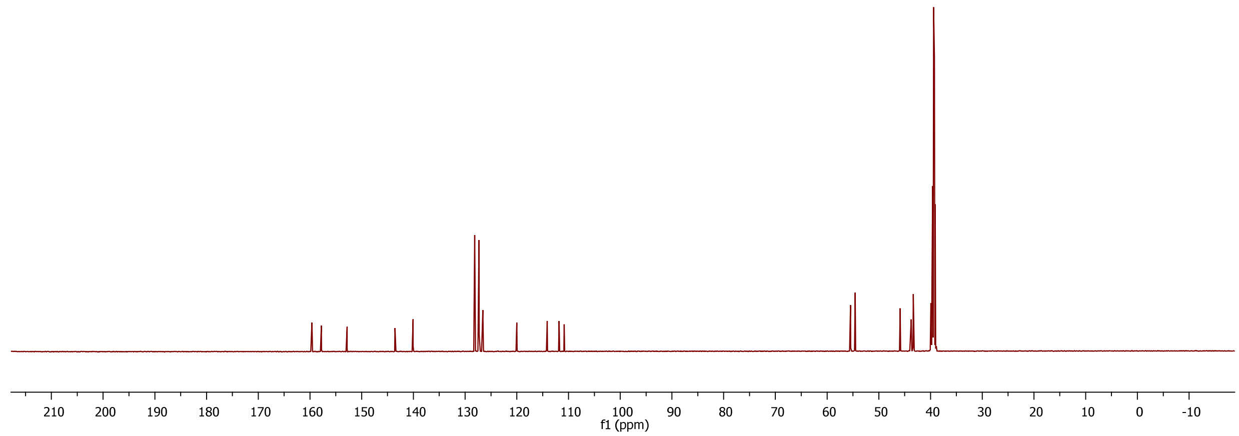

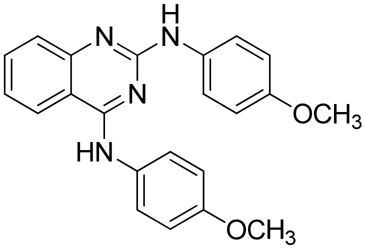

KSC-16-149

***N*[2],*N*[4]-Bis(4-methoxyphenyl)quinazoline-2,4-diamine (S50)**. Yield: 11.9 mg, 91%. ^{1}H NMR (400 MHz, $CDCl_3$) δ 7.71 (d, *J* = 8.0 Hz, 1H), 7.68 – 7.52 (m, 6H), 7.29 (s, br. 1H), 7.24 (ddd, *J* = 1.7, 6.5, 8.2 Hz, 1H), 7.08 – 6.92 (m, 3H), 6.93 – 6.83 (m, 2H), 3.87 (s, 3H), 3.83 (s, 3H). ^{13}C NMR (101 MHz, $CDCl_3$) δ 158.4, 156.9, 156.7, 155.1, 152.0, 133.3, 133.0, 131.3, 126.5, 124.2, 122.2, 121.5, 120.5, 114.2, 114.0, 111.4, 55.58, 55.56. HRMS (m/z): calcd for $C_{22}H_{21}N_4O_2$ (M+H) 373.1665; found 373.1659.

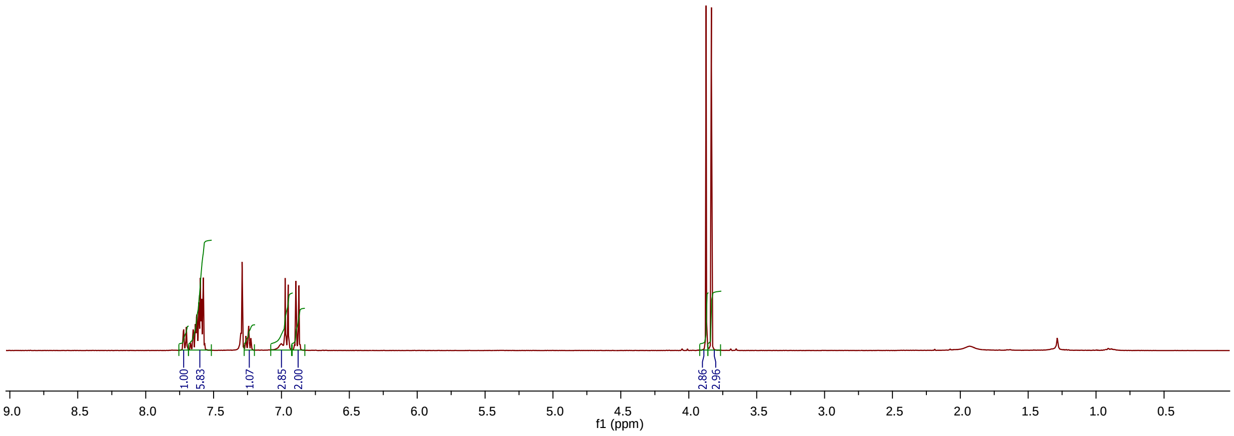

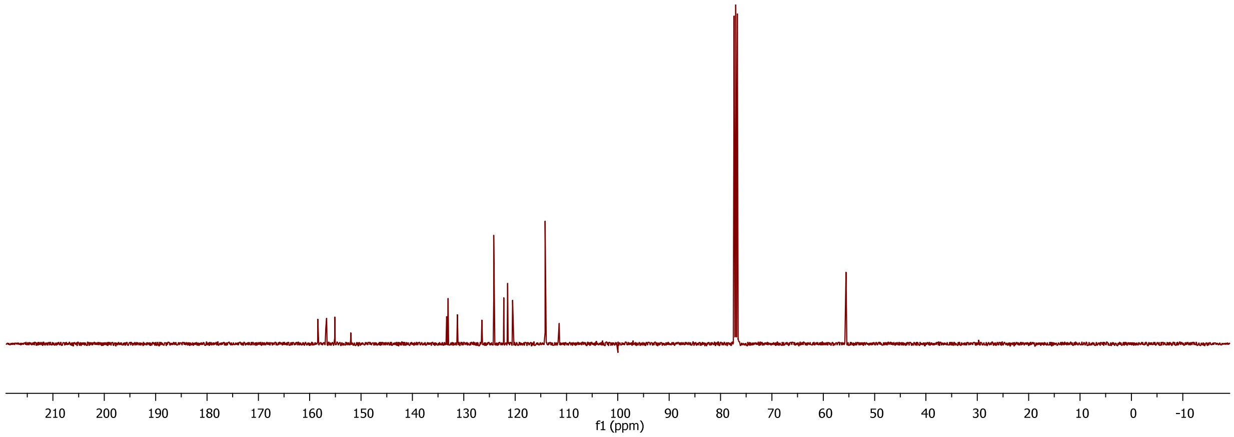

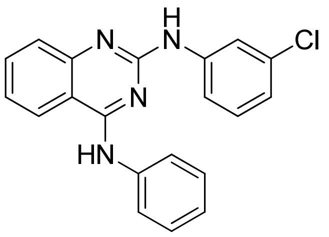

KSC-16-163

***N*^{2}-(3-Chlorophenyl)-*N*^{4}-phenylquinazoline-2,4-diamine (S51)**. Yield: 13.0 mg, 96%. ^{1}H NMR (400 MHz, $CDCl_3$) δ 7.88 (t, *J* = 2.0 Hz, 1H), 7.71 – 7.53 (m, 5H), 7.37 (dd, *J* = 5.2, 10.7 Hz, 3H), 7.31 (s, br. 1H), 7.22 (ddd, *J* = 2.9, 5.3, 8.2 Hz, 1H), 7.17 – 7.00 (m, 3H), 6.89 (ddd, *J* = 0.9, 2.0, 7.9 Hz, 1H). ^{13}C NMR (101 MHz, $CDCl_3$) δ 158.2, 156.1, 151.8, 141.3, 138.1, 134.5, 133.3, 129.7, 129.2, 126.9, 124.7, 123.1, 122.1, 121.9, 120.5, 119.1, 117.1, 111.7. HRMS (m/z): calcd for $C_{20}H_{16}ClN_4$ (M+H) 347.1063; found 347.1061.

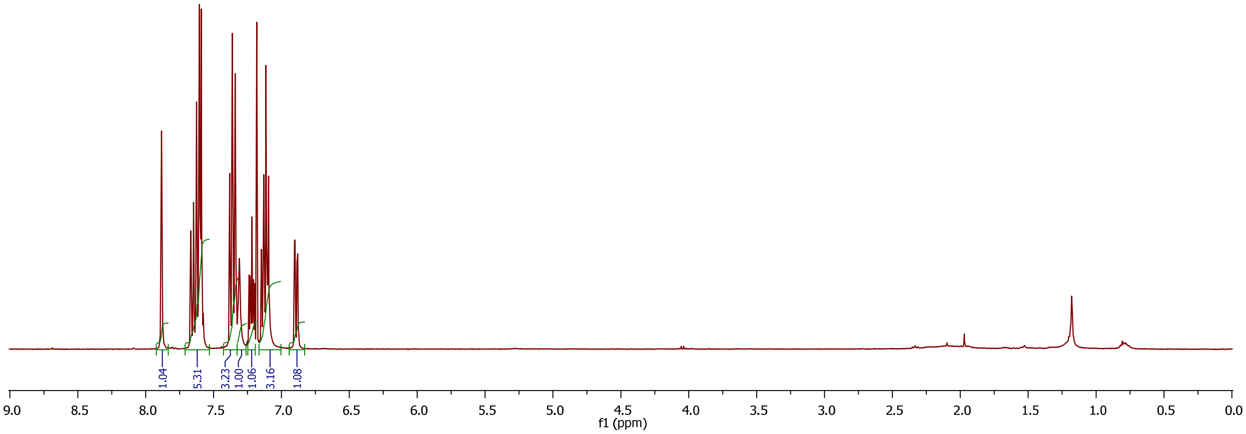

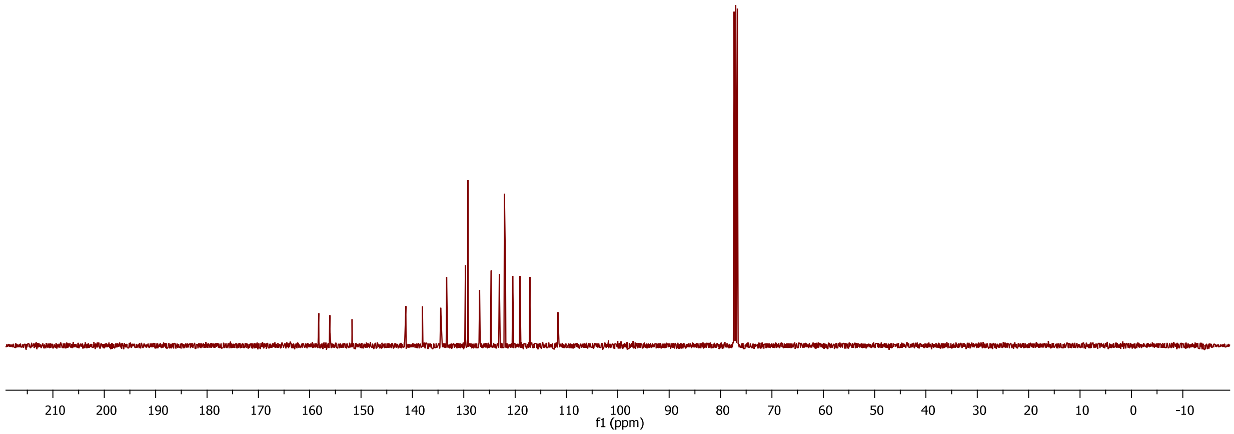

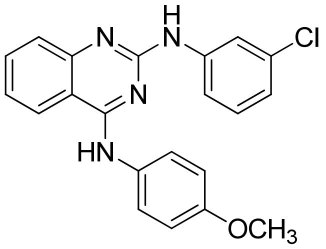

KSC-16-164

***N*[2]-(3-Chlorophenyl)-*N*[4]-(4-methoxyphenyl)quinazoline-2,4-diamine (S52)**. Yield: 13.0 mg, 99%. ^{1}H NMR (400 MHz, $CDCl_3$) δ ^{1}H NMR (400 MHz, CDCl3) δ 7.85 (t, J = 2.0 Hz, 1H), 7.63 (d, J = 8.0 Hz, 1H), 7.61 – 7.52 (m, 2H), 7.49 – 7.39 (m, 2H), 7.31 – 7.11 (m, 4H), 7.08 (t, J = 8.1 Hz, 1H), 6.94 – 6.80 (m, 3H), 3.77 (s, 3H). ^{13}C NMR (101 MHz, $CDCl_3$) δ 158.8, 157.1, 156.2, 151.6, 141.4, 134.4, 133.3, 130.8, 129.6, 126.7, 124.6, 122.9, 121.7, 120.6, 118.9, 117.0, 114.4, 111.6, 55.6. HRMS (m/z): calcd for $C_{21}H_{18}ClN_4O$ (M+H) 377.1169; found 377.1169.

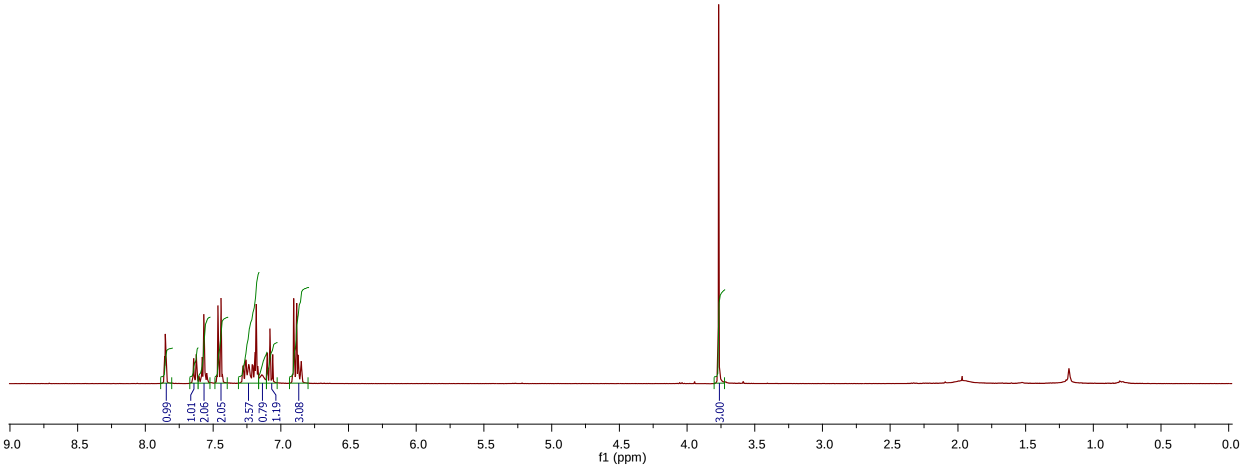

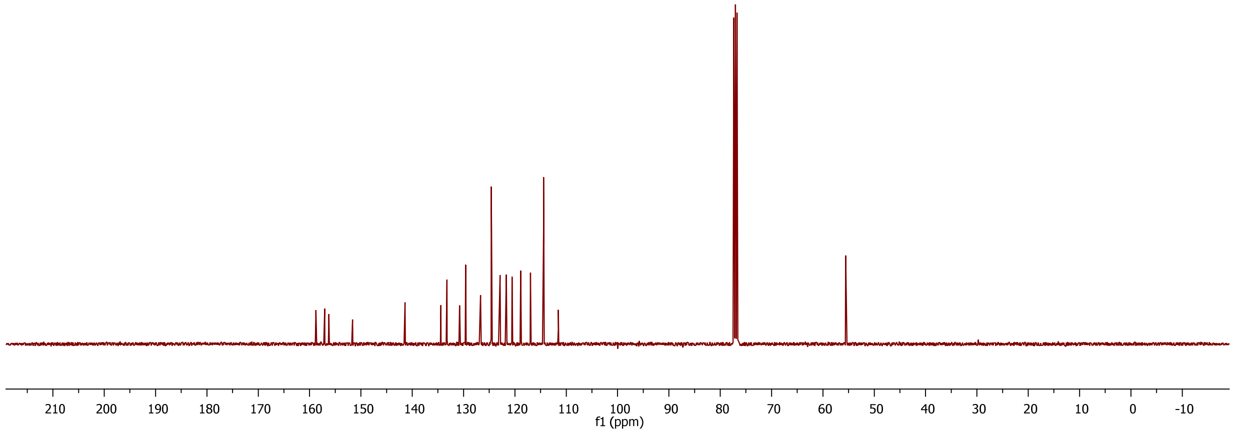

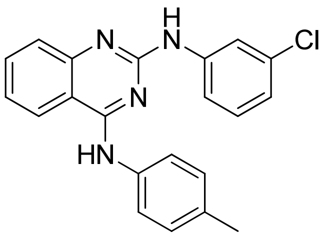

KSC-16-174

***N*^{2}-(3-Chlorophenyl)-*N*^{4}-(*p*-tolyl)quinazoline-2,4-diamine (S53)**. Yield: 5.7 mg, 69%. ^{1}H NMR (400 MHz, $CDCl_3$) δ 7.89 (t, *J* = 2.0 Hz, 1H), 7.66 (d, *J* = 8.1 Hz, 1H), 7.60 (dd, *J* = 1.6, 5.1 Hz, 2H), 7.47 (d, *J* = 8.4 Hz, 2H), 7.38 – 7.30 (m, 1H), 7.22 (ddd, *J* = 4.7, 7.4, 8.2 Hz, 2H), 7.16 (s, br. 2H), 7.12 (t, *J* = 8.1 Hz, 2H), 6.89 (ddd, *J* = 0.9, 2.0, 7.9 Hz, 1H), 2.32 (s, 3H). ^{13}C NMR (101 MHz, $CDCl_3$) δ 158.5, 156.1, 151.7, 141.4, 135.3, 134.6, 134.5, 133.3, 129.7, 129.6, 126.8, 123.0, 122.5, 121.8, 120.5, 119.0, 117.0, 111.7, 21.0. HRMS (m/z): calcd for $C_{21}H_{18}ClN_4$ (M+H) 361.1220; found 361.1228.

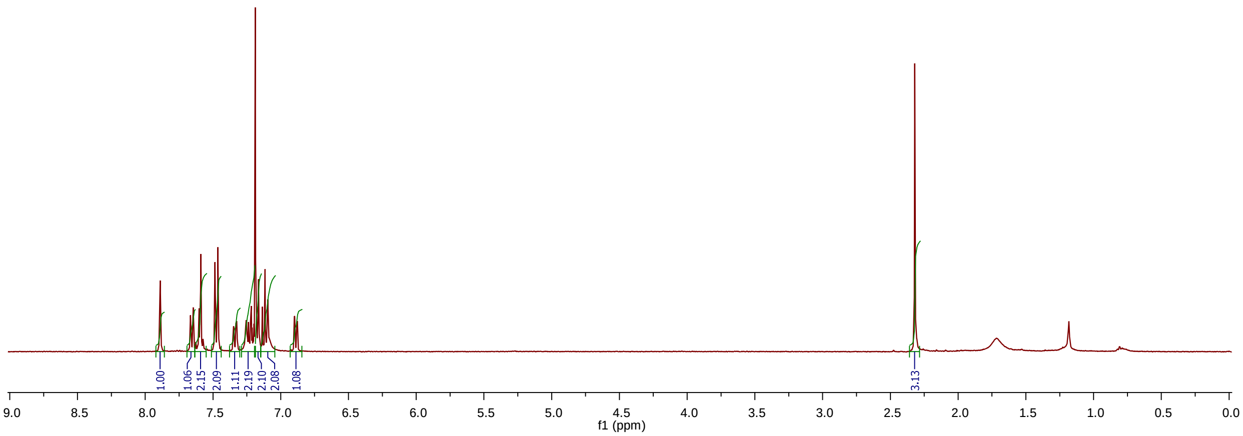

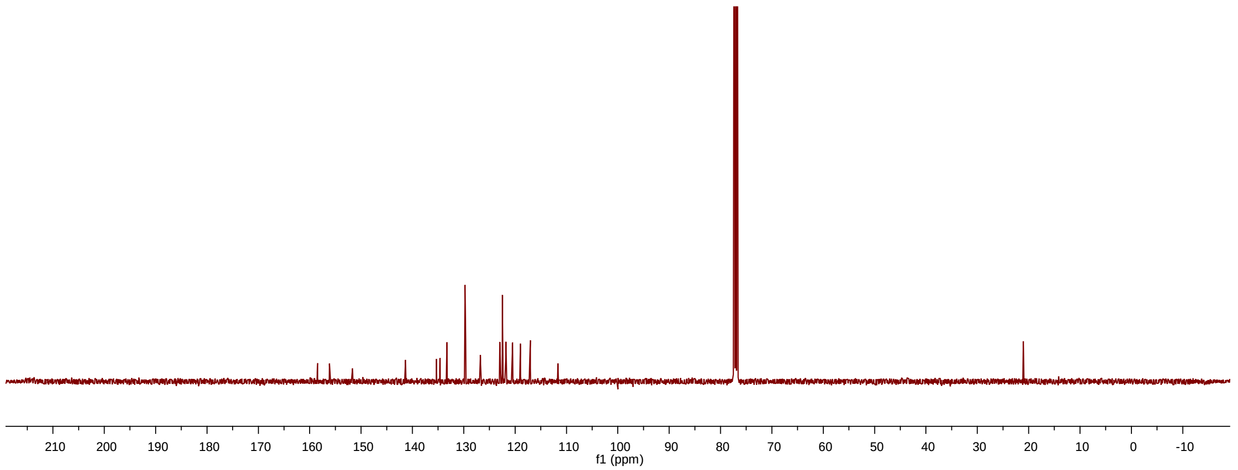

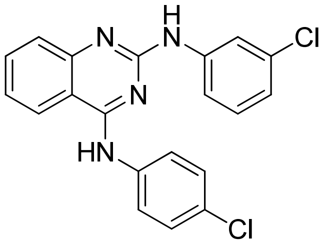

KSC-16-176

***N*²-(3-Chlorophenyl)-*N*⁴-(4-chlorophenyl)quinazoline-2,4-diamine (S54)**. Yield: 13.0 mg, 99%. ^{1}H NMR (400 MHz, $CDCl_3$) δ 7.97 (t, *J* = 2.0 Hz, 1H), 7.78 – 7.61 (m, 5H), 7.46 – 7.36 (m, 4H), 7.32 (ddd, *J* = 2.1, 6.1, 8.2 Hz, 1H), 7.23 (t, *J* = 8.0 Hz, 2H), 7.01 (ddd, *J* = 0.9, 2.0, 7.9 Hz, 1H). ^{13}C NMR (101 MHz, $CDCl_3$) δ 158.1, 156.0, 151.8, 141.2, 136.6, 134.5, 133.5, 129.7, 129.7, 129.2, 126.9, 123.4, 123.2, 122.1, 120.4, 119.2, 117.2, 111.5. HRMS (m/z): calcd for $C_{20}H_{15}Cl_2N_4$ (M+H) 381.0674; found 381.0668.

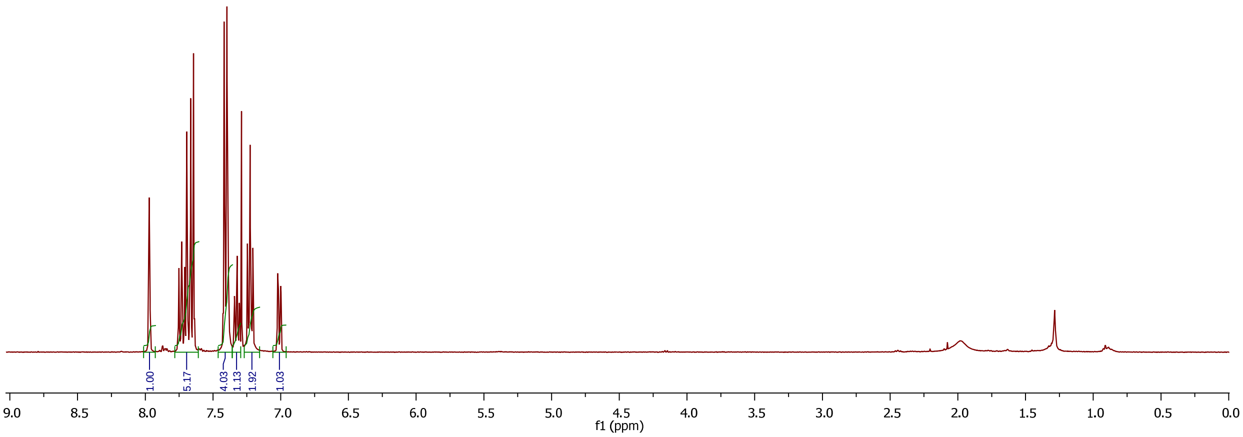

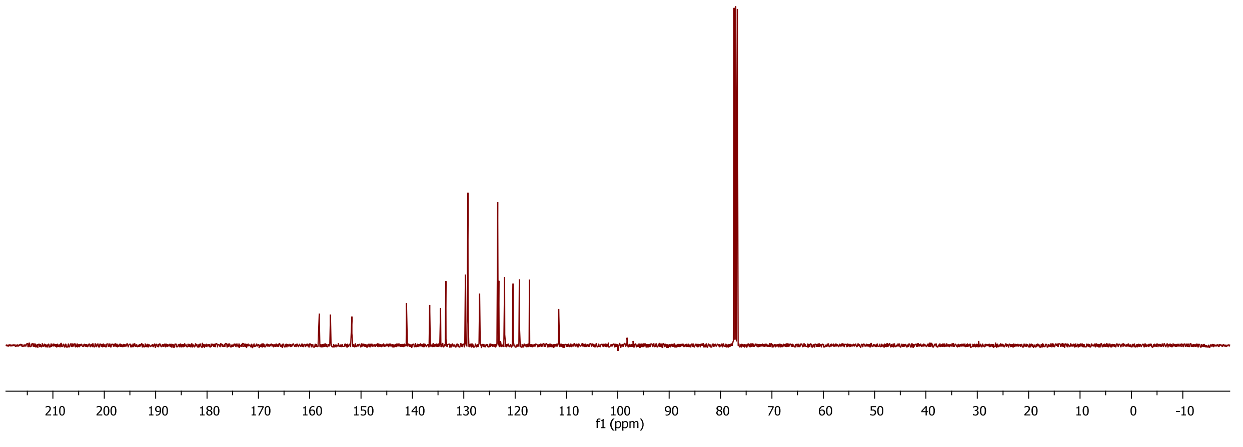

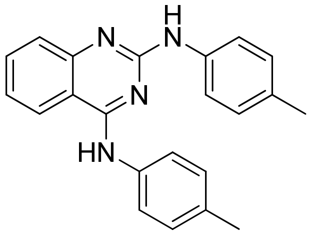

KSC-16-151

***N*[2],*N*[4]-Di-*p*-tolylquinazoline-2,4-diamine (S55)**. Yield: 37.8 mg, 44%. ^{1}H NMR (400 MHz, $CDCl_3$) δ 7.70 (t, *J* = 8.0 Hz, 1H), 7.66 (dd, *J* = 3.8, 4.5 Hz, 2H), 7.64 – 7.56 (m, 4H), 7.39 (s, br. 1H), 7.28 – 7.19 (m, 3H), 7.14 (d, *J* = 8.1 Hz, 2H), 7.08 (s, br. 1H), 2.42 (s, 3H), 2.37 (s, 3H). ^{13}C NMR (101 MHz, $CDCl_3$) δ 158.3, 156.8, 152.1, 137.6, 135.7, 134.1, 133.0, 131.5, 129.5, 129.3, 126.7, 122.3, 122.3, 120.6, 119.7, 111.6, 21.0, 20.9. HRMS (m/z): calcd for $C_{22}H_{21}N_4$ (M+H) 341.1766; found 341.1759.

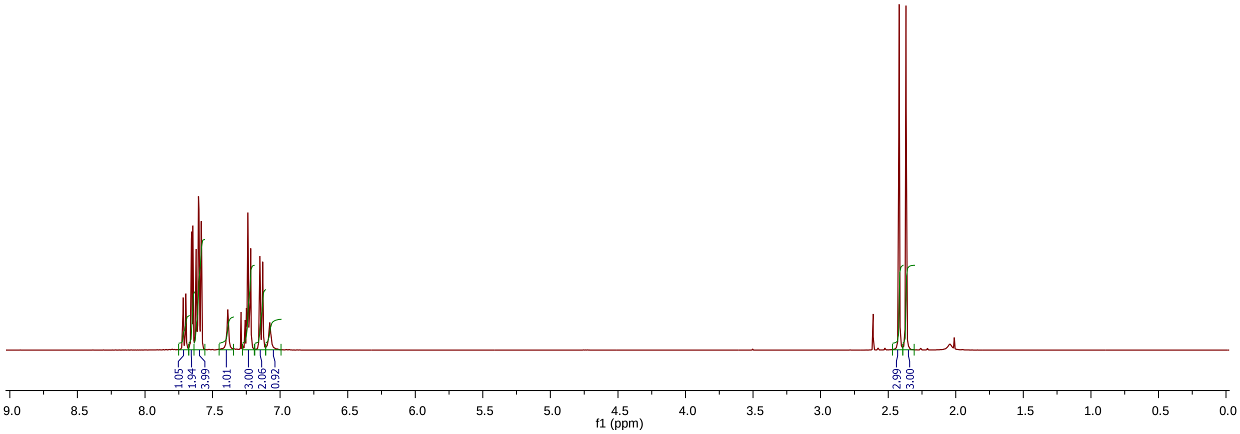

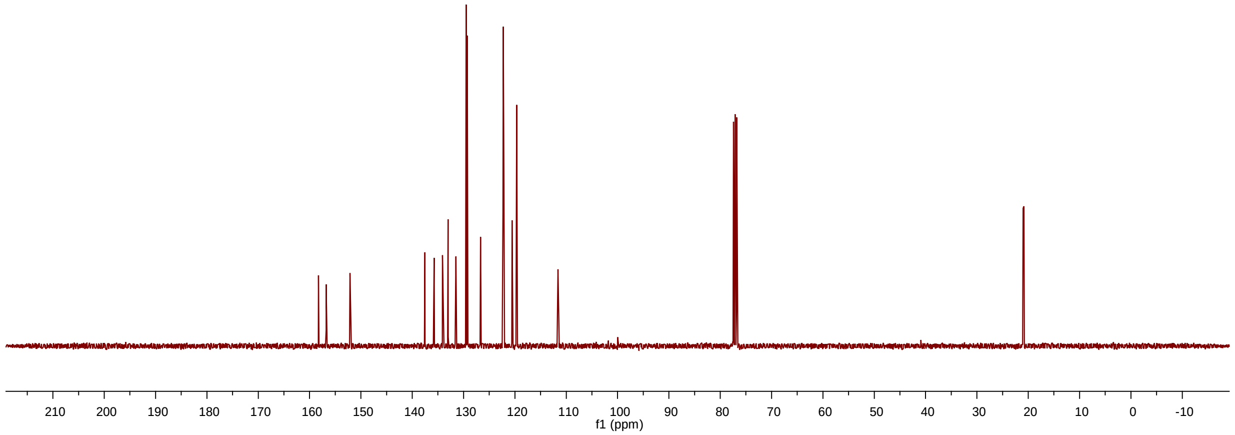

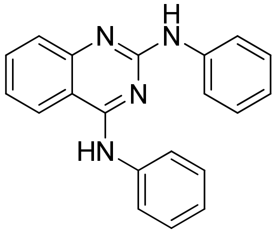

KSC-16-146

***N^2^,N^4^*-Diphenylquinazoline-2,4-diamine (S56)**. Yield: 11.8 mg, 97%. ^{1}H NMR (400 MHz, $CDCl_3$) δ 7.79 – 7.71 (m, 6H), 7.71 – 7.65 (m, 2H), 7.48 – 7.42 (m, 2H), 7.40 (s, br. 1H), 7.38 – 7.29 (m, 3H), 7.26 – 7.20 (m, 1H), 7.17 (s, br. 1H), 7.09 – 7.00 (m, 1H). ^{13}C NMR (101 MHz, $CDCl_3$) δ 158.2, 156.5, 152.0, 140.0, 138.3, 133.2, 129.0, 128.8, 126.8, 124.5, 122.7, 122.1, 122.1, 120.4, 119.5, 111.6. HRMS (m/z): calcd for $C_{20}H_{17}N_4$ (M+H) 313.1453; found 313.1451.

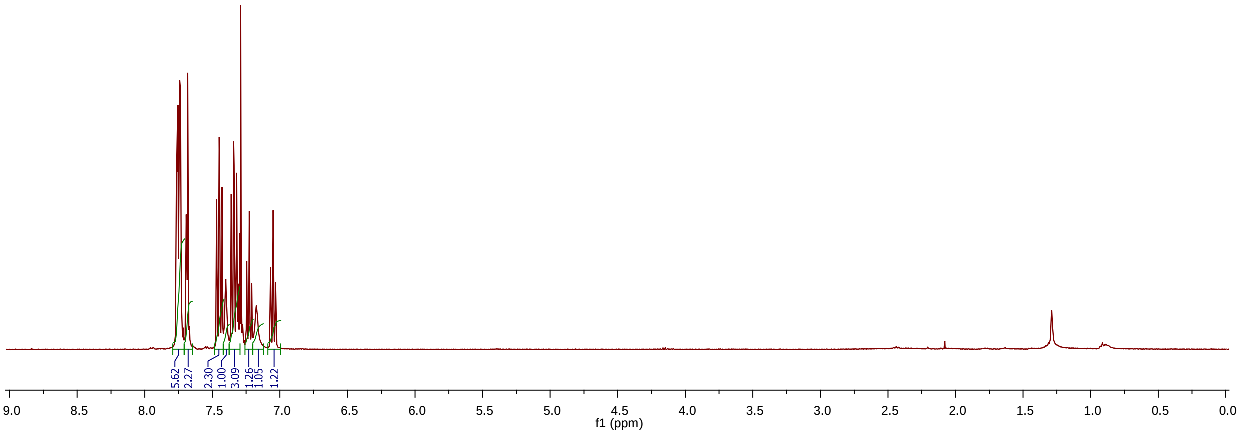

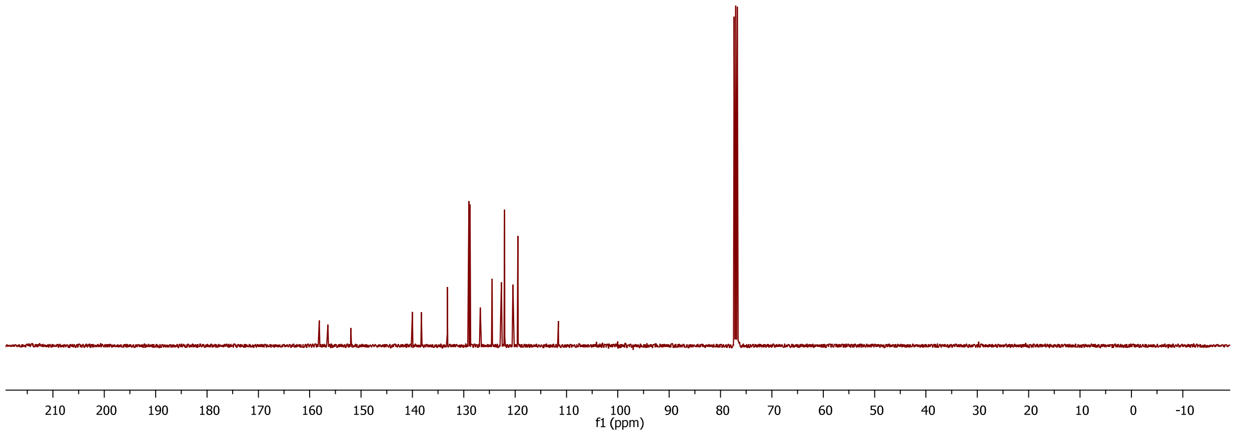

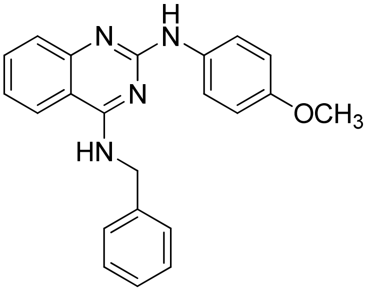

KSC-1-290

***N*[4]-Benzyl-*N*[2]-(4-methoxyphenyl)quinazoline-2,4-diamine (S70)**. Yield: 32.0 mg, 48%. ^{1}H NMR (400 MHz, $CDCl_3$) δ 7.66 – 7.58 (m, 4H), 7.56 (d, *J* = 8.2 Hz, 1H), 7.46 – 7.39 (m, 4H), 7.37 – 7.33 (m, 1H), 7.16 (ddd, *J* = 2.3, 5.8, 8.2 Hz, 1H), 7.02 (s, br. 1H), 6.93 – 6.81 (m, 2H), 5.95 (s, br. 1H), 4.84 (d, *J* = 5.3 Hz, 2H), 3.82 (s, 3H). ^{13}C NMR (101 MHz, $CDCl_3$) δ 160.1, 157.2, 154.9, 151.8, 138.4, 133.6, 132.9, 128.8, 127.9, 127.6, 126.3, 121.9, 121.2, 120.7, 114.0, 111.4, 55.6, 45.3. HRMS (m/z): calcd for $C_{22}H_{21}N_4O$ (M+H) 357.1715; found 357.1711.

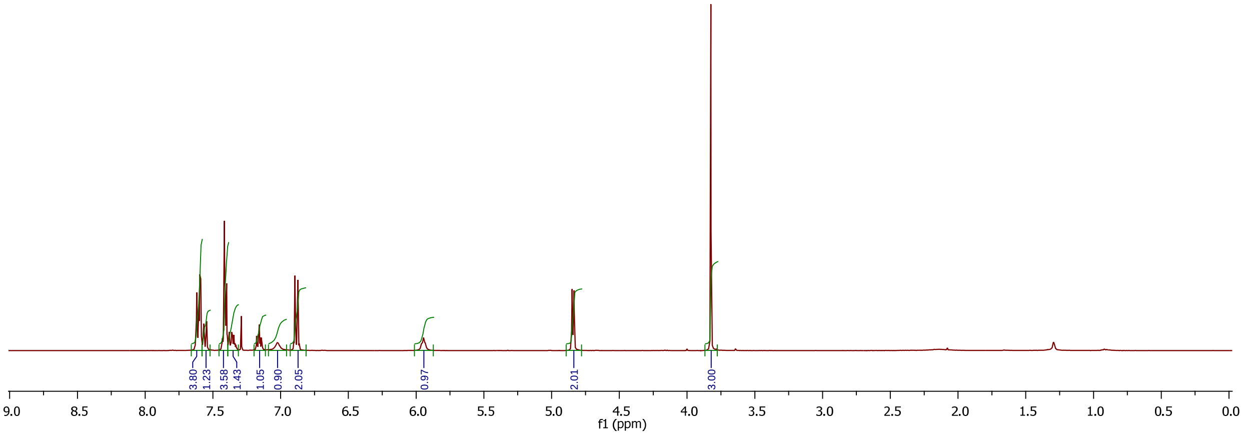

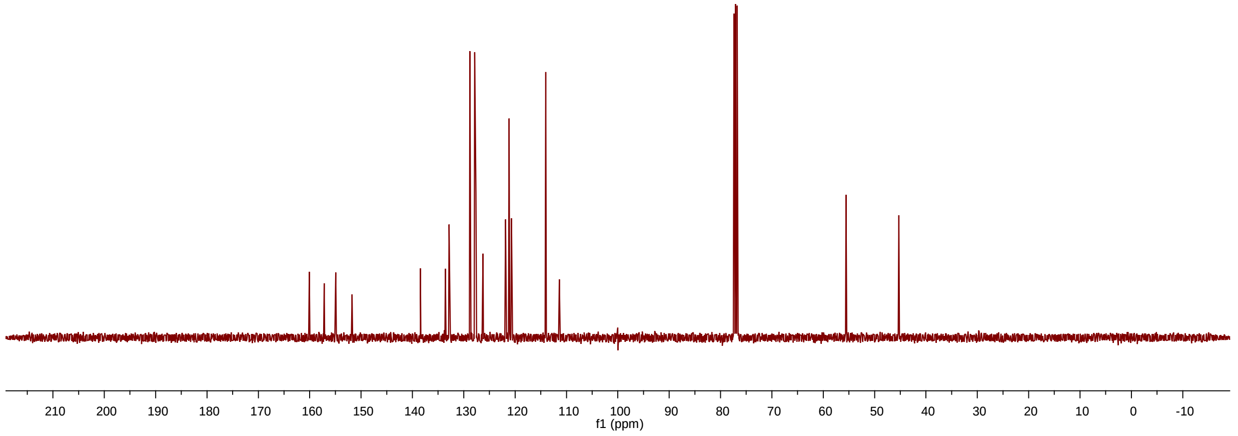

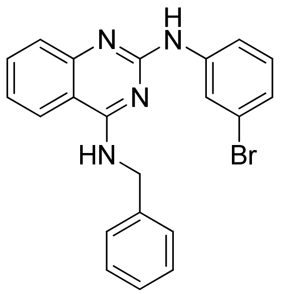

KSC-1-293

***N*[4]-Benzyl-*N*[2]-(3-bromophenyl)quinazoline-2,4-diamine (S71)**. Yield: 47.0 mg, 63%. ^{1}H NMR (400 MHz, $CDCl_3$) δ 8.21 (t, *J* = 1.9 Hz, 1H), 7.64 (t, *J* = 6.5 Hz, 2H), 7.58 (d, *J* = 8.2 Hz, 1H), 7.55 – 7.49 (m, 1H), 7.48 – 7.33 (m, 5H), 7.27 – 7.06 (m, 4H), 5.93 (t, *J* = 5.4 Hz, 1H), 4.88 (d, *J* = 5.4 Hz, 2H). ^{13}C NMR (101 MHz, $CDCl_3$) δ 160.1, 156.5, 151.4, 141.8, 138.2, 133.0, 130.0, 128.9, 127.9, 127.8, 126.6, 124.4, 122.6, 122.6, 121.7, 120.7, 117.3, 111.6, 45.4. HRMS (m/z): calcd for $C_{21}H_{18}BrN_4$ (M+H) 405.0715 and 407.0694; found 407.0691.

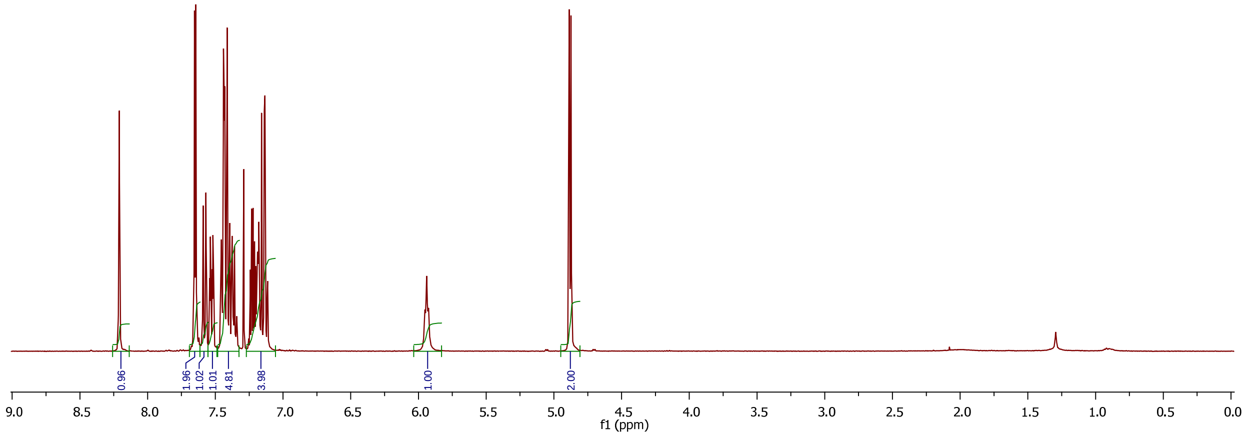

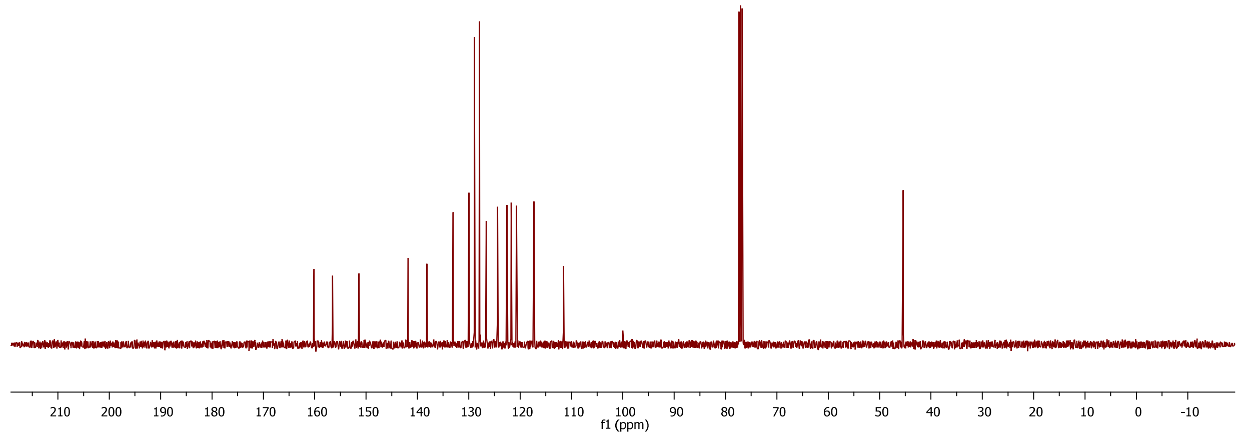

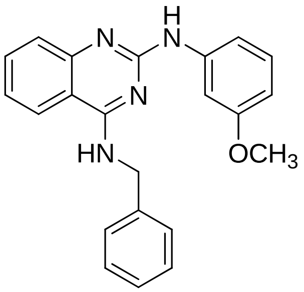

KSC-1-291

***N*[4]-Benzyl-*N*[2]-(3-methoxyphenyl)quinazoline-2,4-diamine (S72)**. Yield: 64.0 mg, 97%. ^{1}H NMR (400 MHz, $CDCl_3$) δ 7.70 – 7.59 (m, 3H), 7.56 (d, J = 8.0 Hz, 1H), 7.46 – 7.30 (m, 6H), 7.25 – 7.13 (m, 3H), 6.59 (ddd, J = 1.6, 2.4, 7.6 Hz, 1H), 6.01 (t, J = 5.3 Hz, 1H), 4.87 (d, J = 5.4 Hz, 2H), 3.81 (s, 3H). ^{13}C NMR (101 MHz, $CDCl_3$) δ 160.2, 160.2, 156.9, 151.6, 141.7, 138.4, 132.9, 129.4, 128.9, 127.9, 127.7, 126.5, 122.3, 120.8, 111.50, 111.47, 107.5, 104.7, 55.2, 45.3. HRMS (m/z): calcd for $C_{22}H_{21}N_4O$ (M+H) 357.1715; found 357.1715.

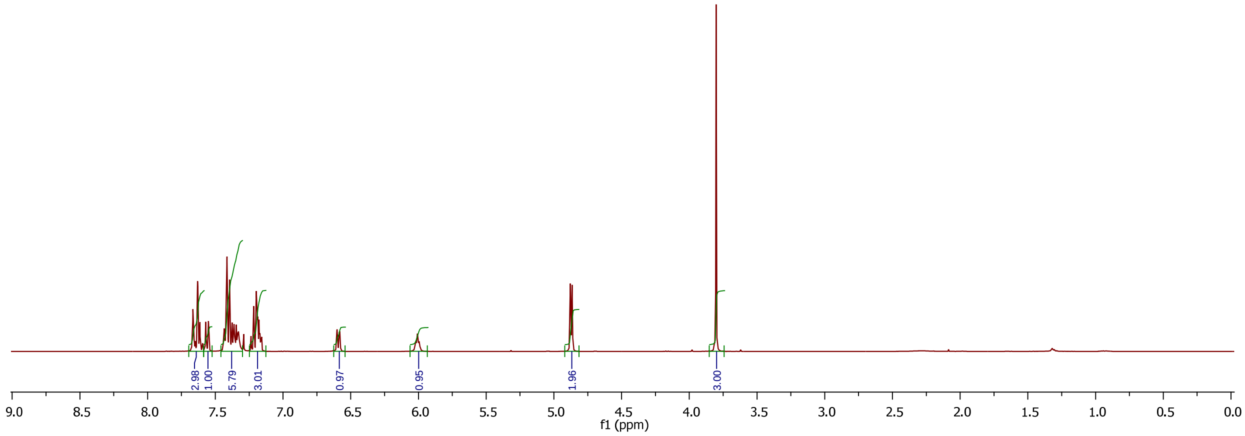

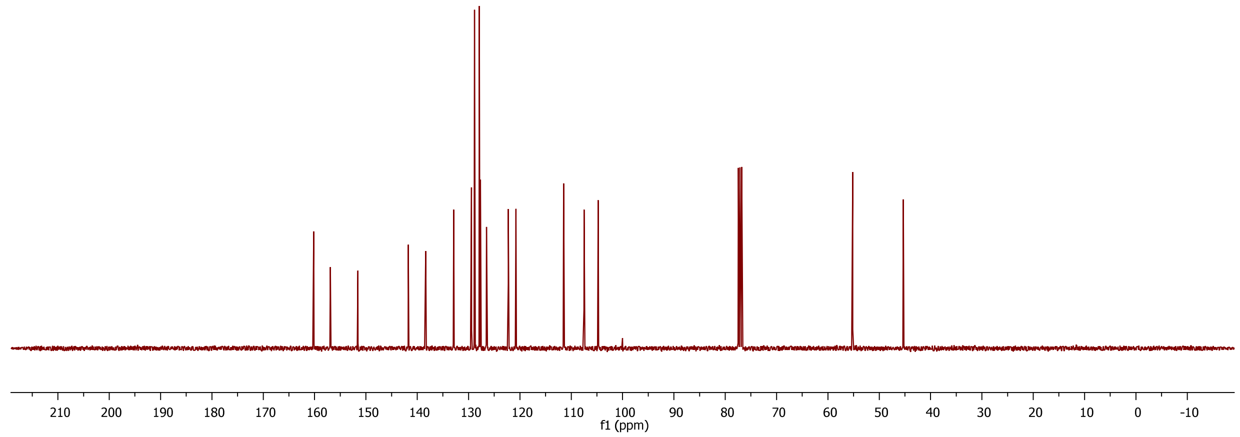

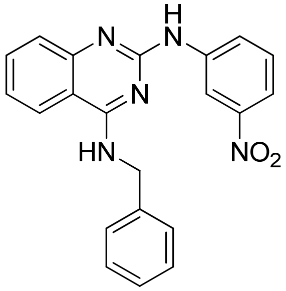

KSC-16-155

***N*^{4}-Benzyl-*N*^{2}-(3-nitrophenyl)quinazoline-2,4-diamine (S73)**. Yield: 11.0 mg, 80%. ^{1}H NMR (400 MHz, CDCl$_3$) δ 9.03 (t, *J* = 2.2 Hz, 1H), 7.82 – 7.68 (m, 2H), 7.62 (d, *J* = 3.6 Hz, 2H), 7.56 (d, *J* = 8.2 Hz, 1H), 7.45 (s, br. 1H), 7.42 – 7.26 (m, 6H), 7.20 (dd, *J* = 3.9, 8.0 Hz, 1H), 5.94 (s, 1H), 4.87 (d, *J* = 5.2 Hz, 2H). ^{13}C NMR (101 MHz, CDCl$_3$) δ 160.2, 156.3, 151.0, 148.8, 141.6, 137.9, 133.3, 129.3, 128.9, 127.9, 127.9, 126.6, 124.1, 123.0, 120.7, 116.1, 113.4, 111.6, 45.6, 29.7. HRMS (m/z): calcd for $C_{21}H_{18}N_5O_2$ (M+H) 372.1460; found 372.1462.

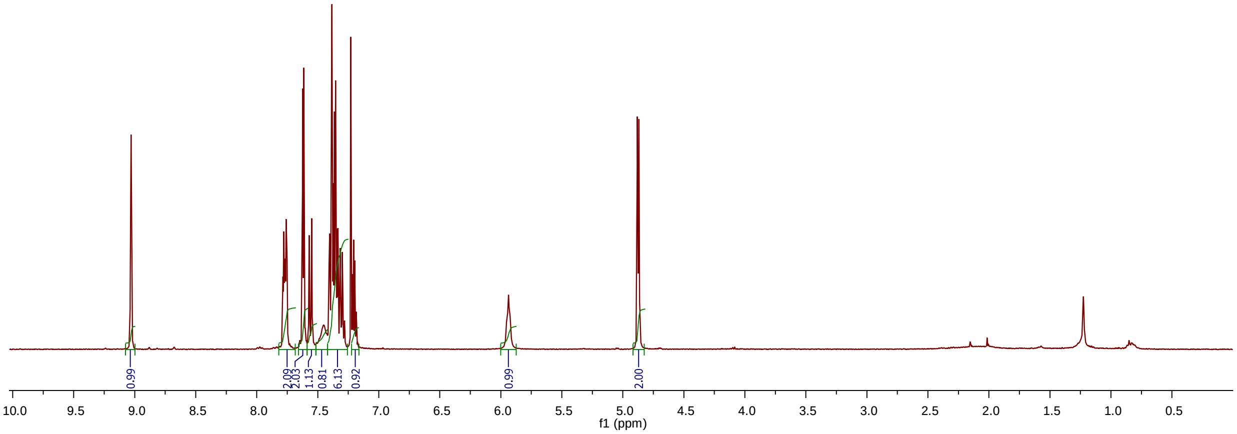

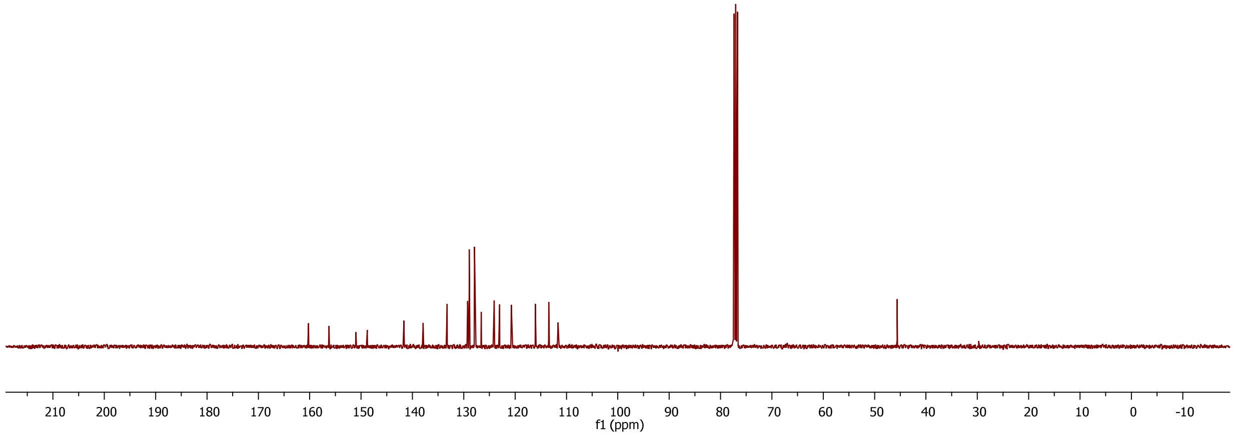

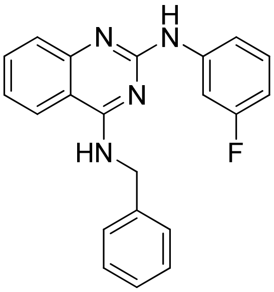

KSC-1-292

***N*[4]-Benzyl-*N*[2]-(3-fluorophenyl)quinazoline-2,4-diamine (S74 or 44)**. Yield: 58.0 mg, 91%. ^{1}H NMR (400 MHz, $CDCl_3$) δ 8.00 – 7.87 (m, 1H), 7.71 – 7.61 (m, 2H), 7.58 (d, *J* = 8.1, 1H), 7.50 – 7.33 (m, 5H), 7.29 – 7.12 (m, 4H), 6.78 – 6.62 (m, 1H), 5.97 (s, br. 1H), 4.87 (d, *J* = 5.3 Hz, 2H). ^{13}C NMR (101 MHz, $CDCl_3$) δ 164.5, 162.1, 160.1, 156.5, 151.4, 142.2, 142.1, 138.2, 133.0, 129.7, 129.6, 128.89, 127.87, 127.8, 126.6, 122.6, 120.7, 114.12, 114.09, 111.6, 108.2, 108.0, 106.2, 105.9, 45.4. HRMS (m/z): calcd for $C_{21}H_{18}FN_4$ (M+H) 345.1515; found 345.1510.

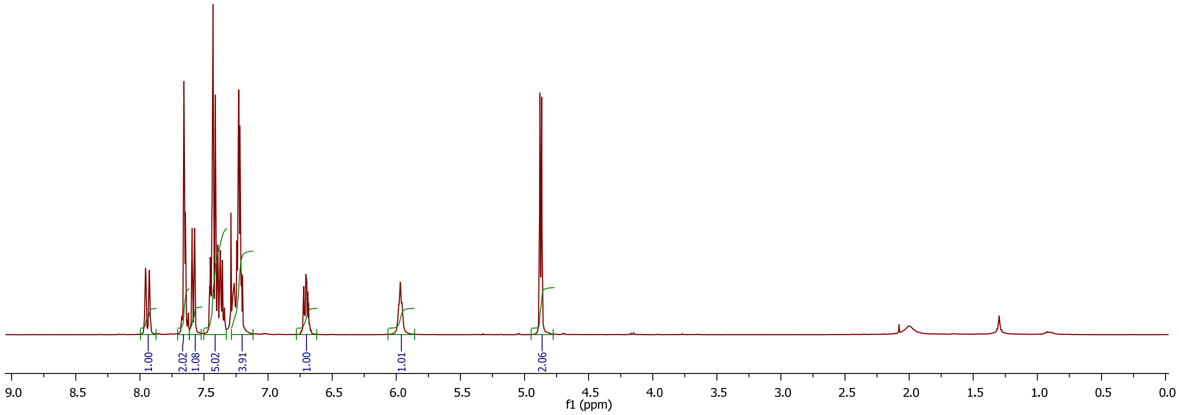

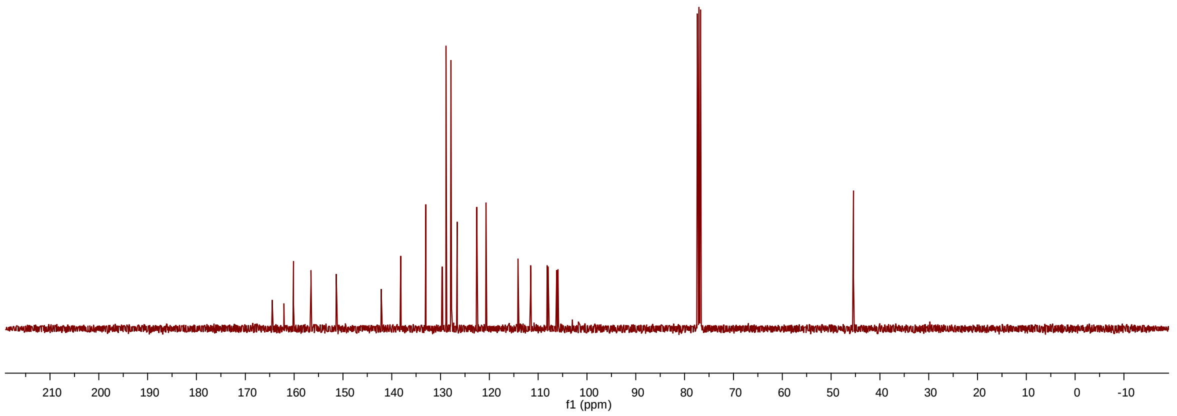

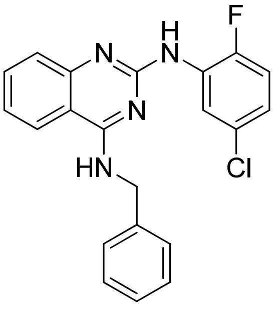

KSC-1-295

***N*^{4}-Benzyl-*N*^{2}-(5-chloro-2-fluorophenyl)quinazoline-2,4-diamine (S75)**. Yield: 58.0 mg, 83%. ^{1}H NMR (400 MHz, $CDCl_3$) δ 9.00 (dd, *J* = 2.6, 7.3 Hz, 1H), 7.77 – 7.62 (m, 2H), 7.59 (d, *J* = 8.0 Hz, 1H), 7.52 – 7.32 (m, 5H), 7.25 – 7.23 (m, 1H), 7.21 (s, br. 1H), 7.03 (dd, *J* = 8.7, 11.0 Hz, 1H), 6.89 (ddd, *J* = 2.6, 4.3, 8.6 Hz, 1H), 5.93 (s, br. 1H), 4.88 (t, *J* = 7.5 Hz, 2H). ^{13}C NMR (101 MHz, $CDCl_3$) δ 160.1, 156.1, 151.7, 151.2, 149.3, 138.1, 133.1, 130.1, 130.0, 129.4, 129.3, 128.9, 128.0, 127.8, 126.8, 123.0, 120.7, 120.53, 120.45, 120.0, 115.3, 115.1, 111.7, 45.5. HRMS (m/z): calcd for $C_{21}H_{17}ClFN_4$ (M+H) 379.1126; found 379.1119.

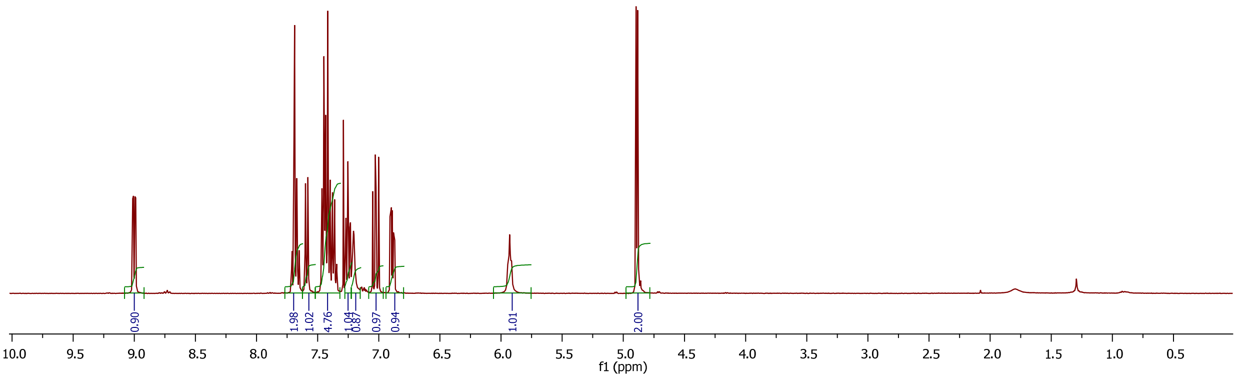

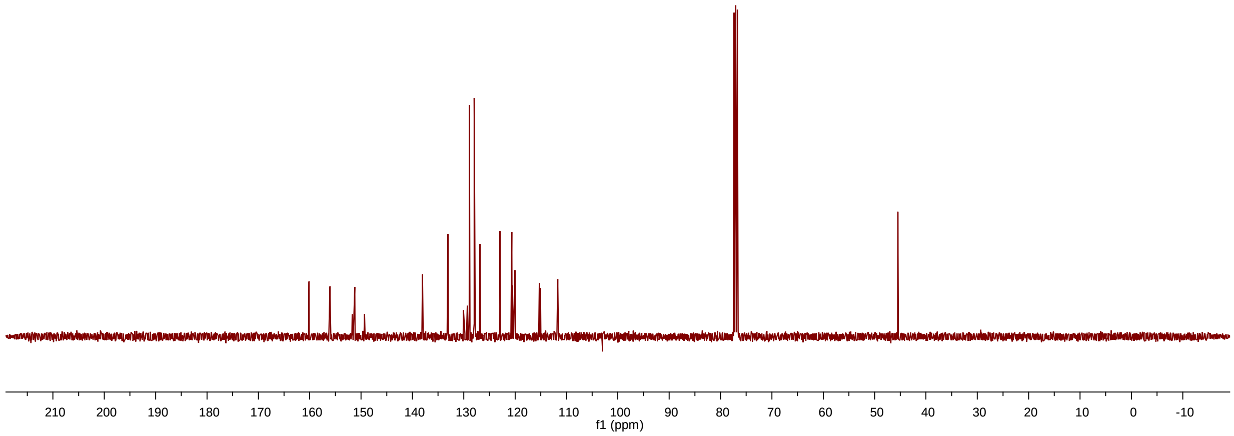

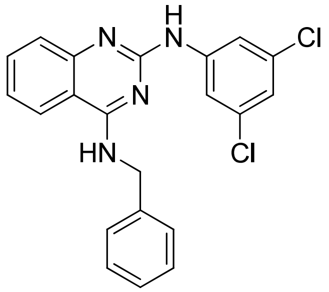

KSC-1-294

***N*[4]-Benzyl-*N*[2]-(3,5-dichlorophenyl)quinazoline-2,4-diamine (S76)**. Yield: 73.0 mg, 100%. ^{1}H NMR (400 MHz, $CDCl_3$) δ 7.75 (d, *J* = 1.8 Hz, 2H), 7.69 – 7.61 (m, 2H), 7.58 (d, *J* = 8.2 Hz, 1H), 7.52 – 7.31 (m, 6H), 7.28 – 7.18 (m, 1H), 6.96 (t, *J* = 1.8 Hz, 1H), 6.00 (t, *J* = 5.3 Hz, 1H), 4.86 (d, *J* = 5.4 Hz, 2H). ^{13}C NMR (101 MHz, $CDCl_3$) δ 160.2, 156.3, 151.1, 142.4, 138.0, 134.8, 133.2, 128.9, 127.9, 127.8, 126.6, 122.9, 121.1, 120.8, 116.9, 111.6, 45.4. HRMS (m/z): calcd for $C_{21}H_{17}Cl_2N_4$ (M+H) 395.0830; found 395.0924.

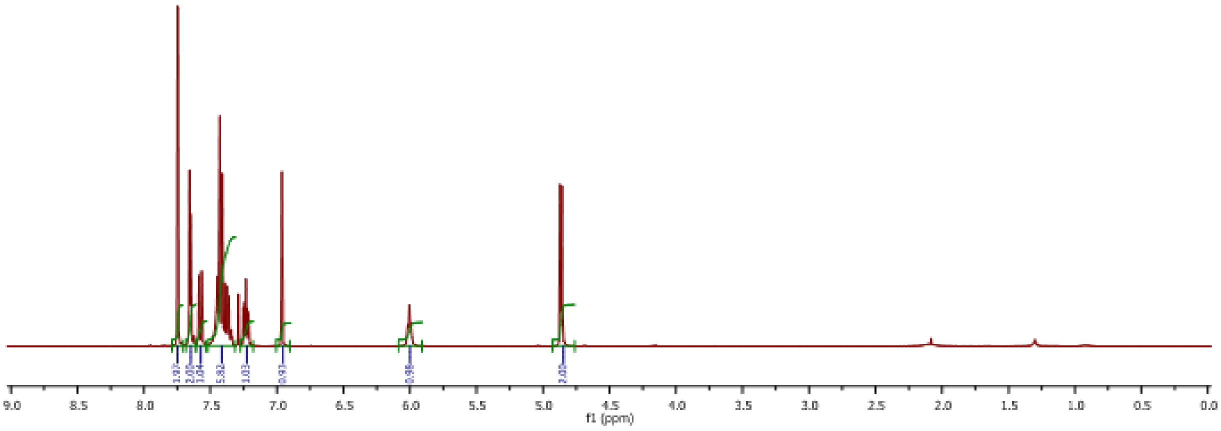

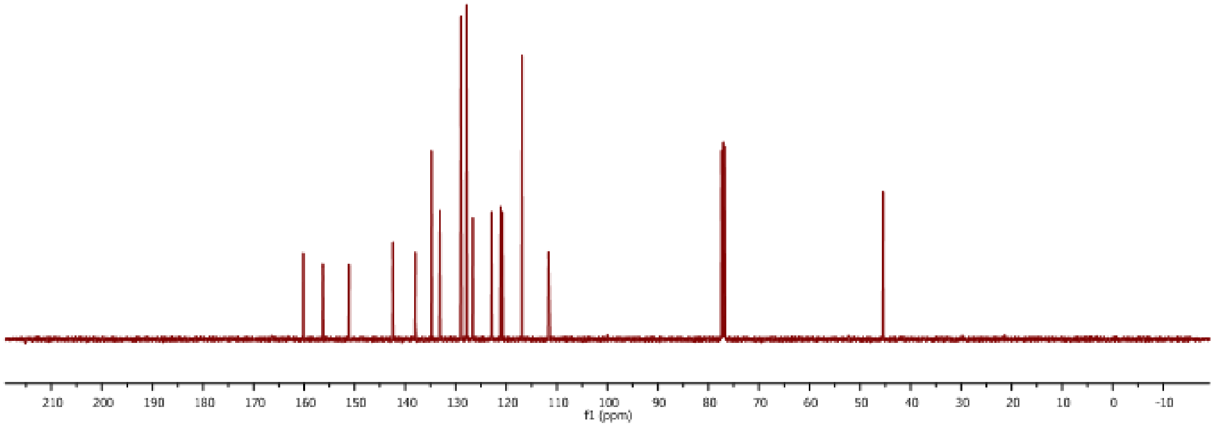

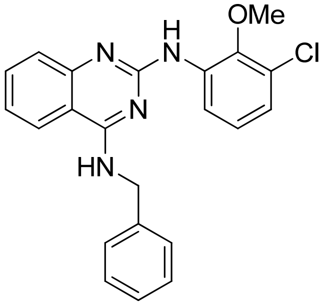

KSC-16-103

***N*^{4}-Benzyl-*N*^{2}-(3-chloro-2-methoxyphenyl)quinazoline-2,4-diamine (S77)**. Yield: 6.2 mg, 43%. ^{1}H NMR (400 MHz, $CDCl_3$) δ 8.60 (s, 1H), 7.61 (dd, *J* = 1.2, 4.6 Hz, 2H), 7.55 (d, *J* = 8.1 Hz, 1H), 7.47 – 7.28 (m, 5H), 7.23 – 7.10 (m, 1H), 6.99 (t, *J* = 8.1 Hz, 1H), 6.93 (dd, *J* = 1.7, 8.1 Hz, 1H), 5.92 (s, br. 1H), 4.85 (d, *J* = 5.3 Hz, 2H), 3.91 (s, 3H). ^{13}C NMR (101 MHz, $CDCl_3$) δ 160.1, 156.4, 144.6, 138.2, 135.4, 133.1, 128.9, 127.9, 127.8, 126.9, 126.3, 124.8, 122.7, 122.2, 120.7, 117.9, 111.5, 60.7, 45.5. HRMS (m/z): calcd for $C_{22}H_{20}ClN_4O$ (M+H) 391.1326; found 391.1324.

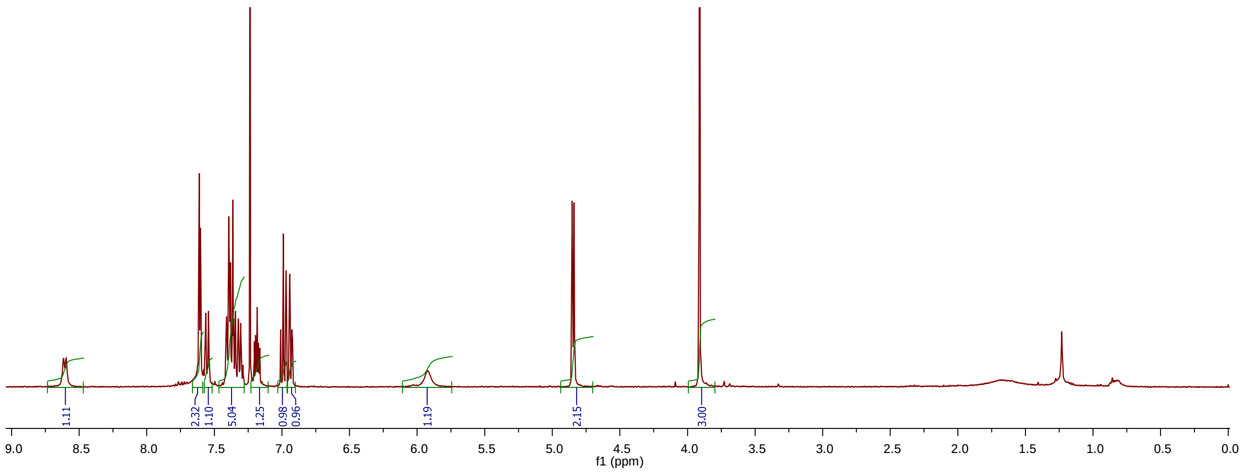

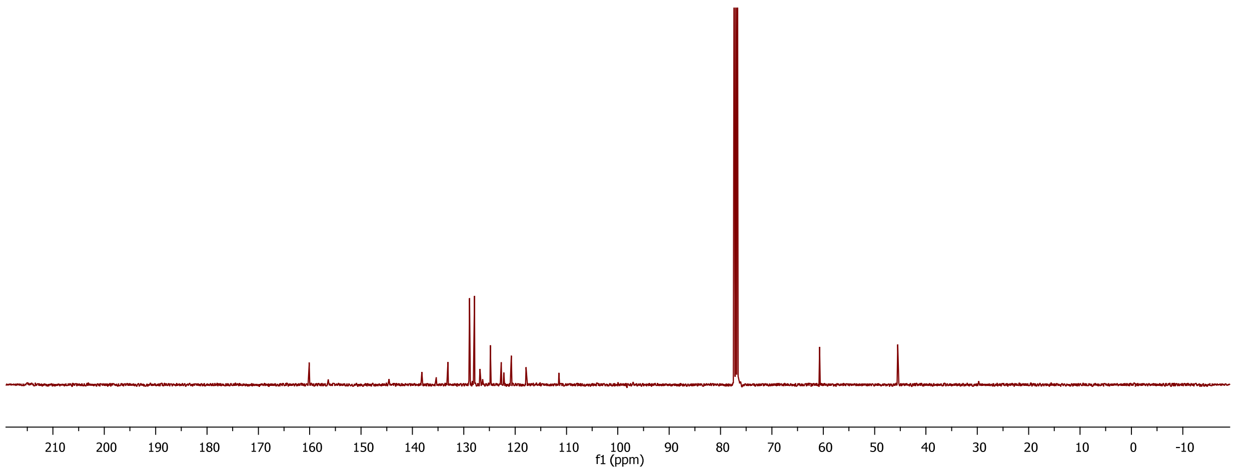

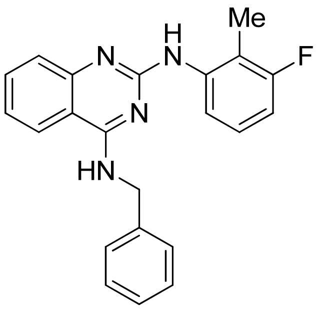

KSC-16-104

***N*^4-Benzyl-*N*^2-(3-fluoro-2-methylphenyl)quinazoline-2,4-diamine (S78)**. Yield: 13.0 mg, 98%. ^{1}H NMR (400 MHz, $CDCl_3$) δ 8.07 (d, *J* = 8.3 Hz, 1H), 7.66 – 7.58 (m, 3H), 7.46 – 7.31 (m, 5H), 7.21 (ddd, *J* = 1.8, 6.3, 8.2 Hz, 1H), 7.15 (dd, *J* = 8.0, 15.0 Hz, 1H), 6.88 (s, br. 1H), 6.79 (t, *J* = 8.5 Hz, 1H), 5.97 (s, br. 1H), 4.84 (d, *J* = 5.2 Hz, 2H), 2.27 (d, *J* = 1.7 Hz, 3H). ^{13}C NMR (101 MHz, $CDCl_3$) δ 162.4, 160.2, 160.0, 157.1, 151.5, 139.9, 139.9, 138.3, 133.0, 128.8, 127.9, 127.7, 126.5, 126.43, 126.36, 122.4, 120.7, 116.89, 116.86, 115.1, 115.0, 111.5, 109.3, 109.0, 45.3, 9.32, 9.26. HRMS (m/z): calcd for $C_{22}H_{20}FN_4$ (M+H) 359.1672; found 359.1677.

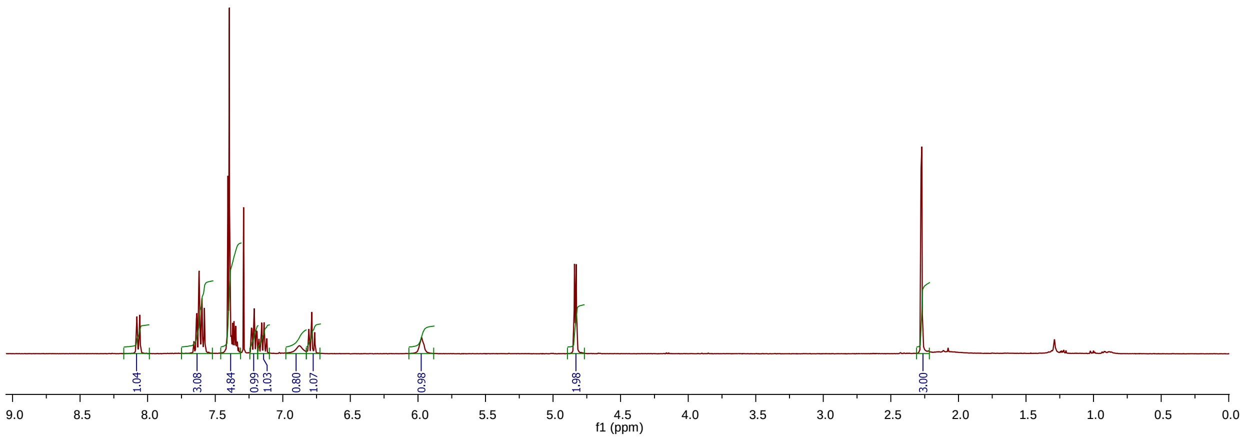

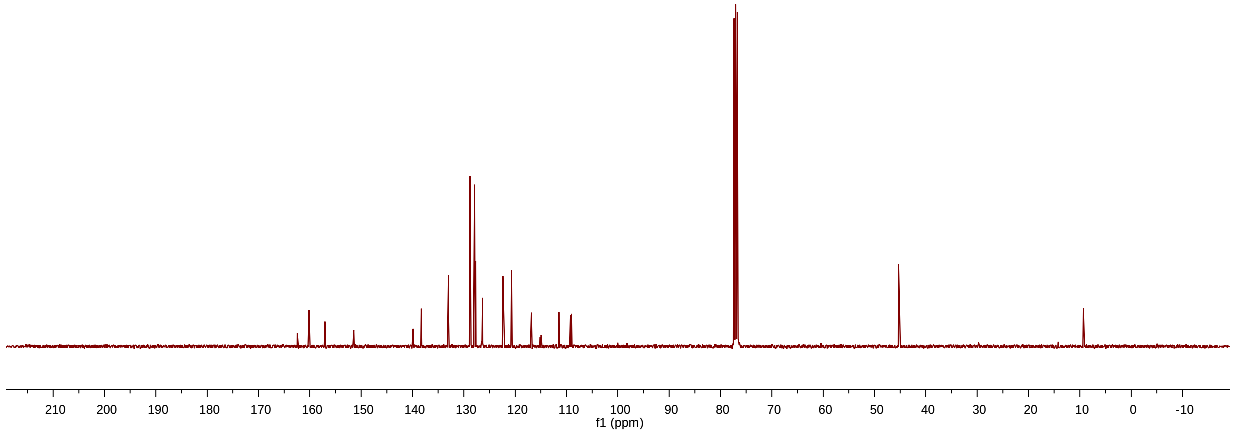

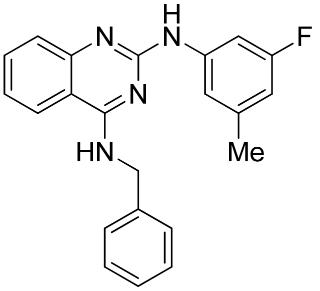

KSC-16-105

***N*^4-Benzyl-*N*^2-(3-fluoro-5-methylphenyl)quinazoline-2,4-diamine (S79)**. Yield: 13.0 mg, 98%. ^{1}H NMR (400 MHz, $CDCl_3$) δ 7.64 (d, J = 11.6 Hz, 1H), 7.54 (d, J = 3.5 Hz, 2H), 7.47 (d, J = 8.1 Hz, 1H), 7.39 – 7.21 (m, 5H), 7.15 – 6.99 (m, 2H), 6.94 (s, 1H), 6.43 (d, J = 9.3 Hz, 1H), 5.85 (s, br. 1H), 4.77 (d, J = 5.0 Hz, 2H), 2.22 (s, 3H). ^{13}C NMR (101 MHz, $CDCl_3$) δ 164.4, 162.0, 160.1, 156.5, 151.3, 141.7, 141.6, 140.1, 140.0, 138.2, 133.0, 128.9, 127.9, 127.8, 126.5, 122.5, 120.7, 114.74, 114.72, 111.5, 109.1, 108.9, 103.4, 103.2, 103.0, 45.4, 21.58, 21.56. HRMS (m/z): calcd for $C_{22}H_{20}FN_4$ (M+H) 359.1672; found 359.1675.

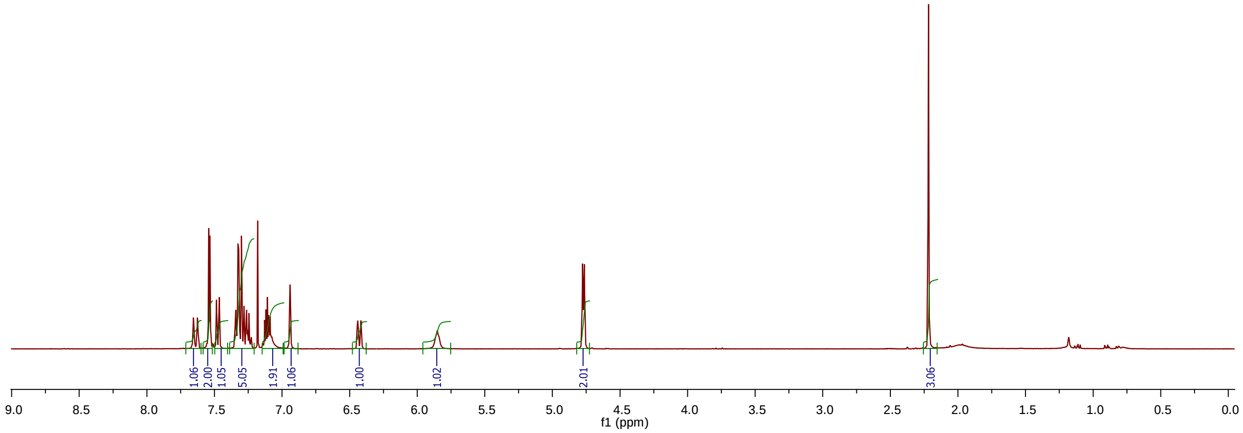

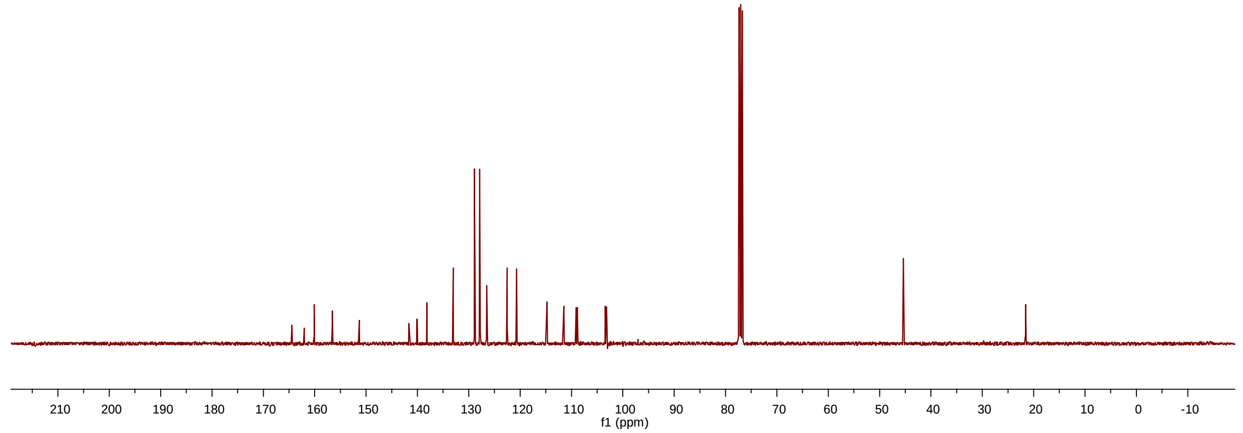

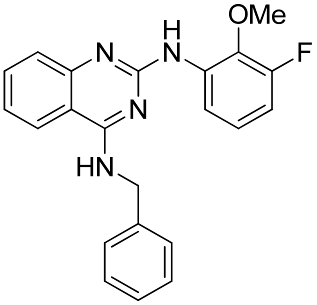

KSC-16-106

***N*[4]-Benzyl-*N*[2]-(3-fluoro-2-methoxyphenyl)quinazoline-2,4-diamine (S80)**. Yield: 12.4 mg, 89%. ^{1}H NMR (400 MHz, $CDCl_3$) δ 8.44 (d, *J* = 8.4 Hz, 1H), 7.72 – 7.41 (m, 4H), 7.41 – 7.21 (m, 5H), 7.12 (ddd, *J* = 2.8, 5.3, 8.2 Hz, 1H), 6.89 (td, *J* = 6.0, 8.4 Hz, 1H), 6.62 (ddd, *J* = 1.5, 8.4, 11.2 Hz, 1H), 5.85 (s, br. 1H), 4.80 (d, *J* = 5.3 Hz, 2H), 3.92 (d, *J* = 1.6 Hz, 3H). ^{13}C NMR (101 MHz, $CDCl_3$) δ 160.1, 156.5, 156.3, 153.9, 151.2, 138.2, 136.0, 135.8, 134.9, 134.9, 133.0, 128.9, 127.9, 127.8, 126.5, 123.5, 123.4, 122.6, 120.7, 114.3, 111.5, 108.8, 108.6, 61.5, 61.4, 45.5. HRMS (m/z): calcd for $C_{22}H_{20}FN_4O$ (M+H) 375.1621; found 375.1624.

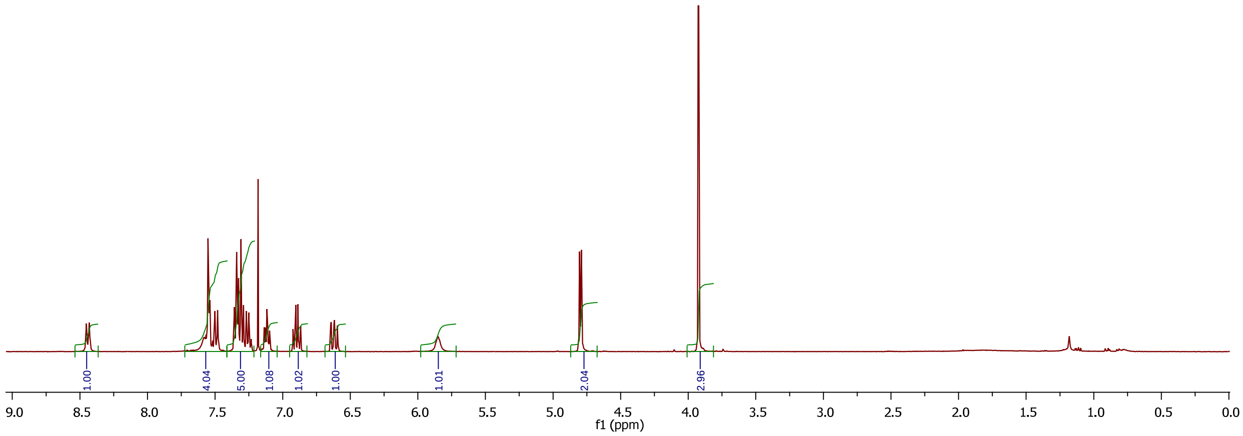

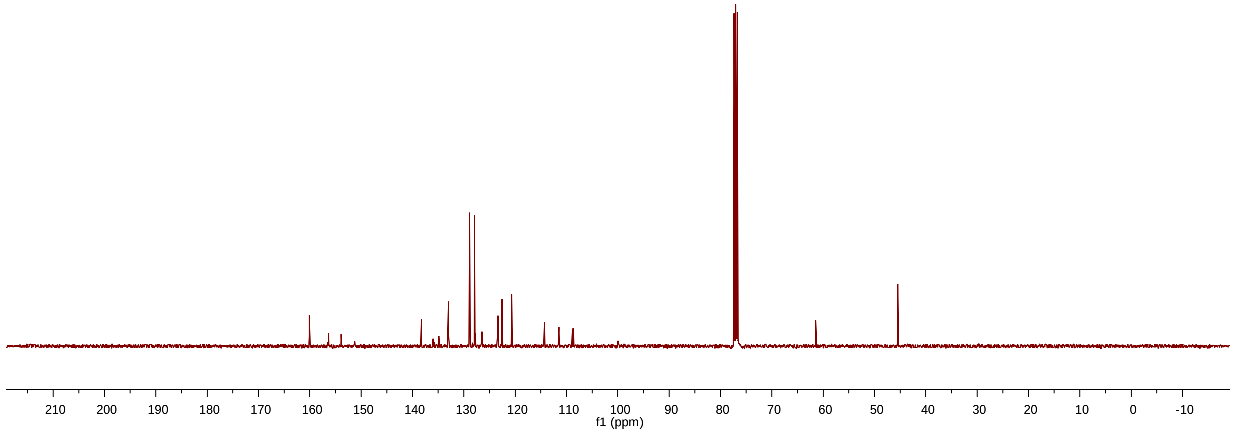

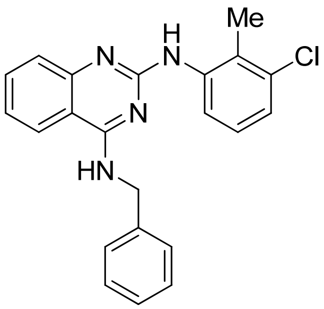

KSC-16-107

***N*[4]-Benzyl-*N*[2]-(3-chloro-2-methylphenyl)quinazoline-2,4-diamine (S81)**. Yield: 11.7 mg, 84%. ^{1}H NMR (400 MHz, $CDCl_3$) δ 8.02 (dd, *J* = 2.6, 6.7 Hz, 1H), 7.60 – 7.53 (m, 3H), 7.41 – 7.26 (m, 5H), 7.16 (ddd, *J* = 1.5, 6.6, 8.2 Hz, 1H), 7.12 – 7.01 (m, 2H), 7.00 – 6.71 (s, br. 1H), 5.92 (s, br. 1H), 4.76 (d, *J* = 5.2 Hz, 2H), 2.38 (s, 3H). ^{13}C NMR (101 MHz, $CDCl_3$) δ 160.2, 157.1, 151.1, 139.5, 138.2, 134.5, 133.1, 128.8, 127.9, 127.7, 126.9, 126.5, 126.1, 123.9, 122.4, 120.8, 120.7, 111.4, 45.3, 14.8. HRMS (m/z): calcd for $C_{22}H_{20}ClN_4$ (M+H) 375.1376; found 375.1378.

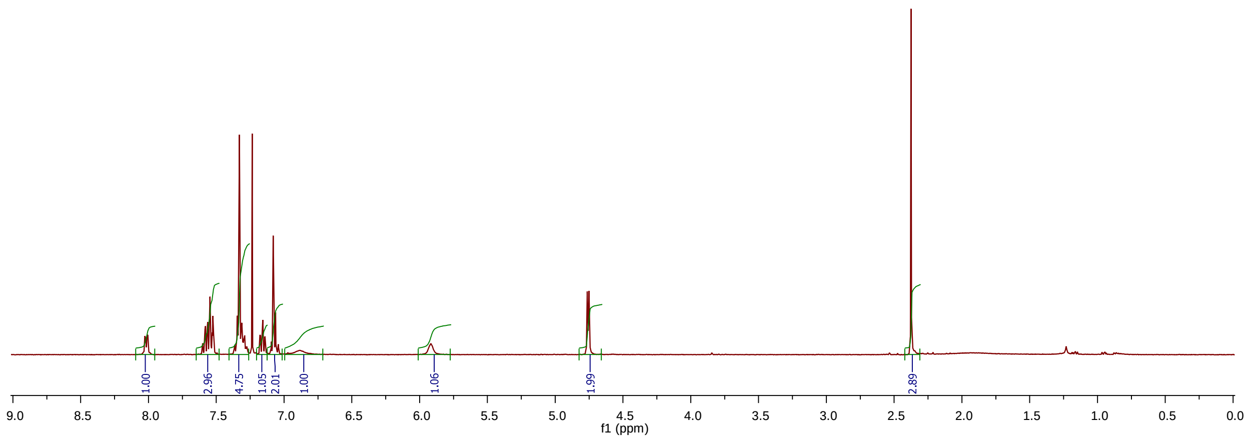

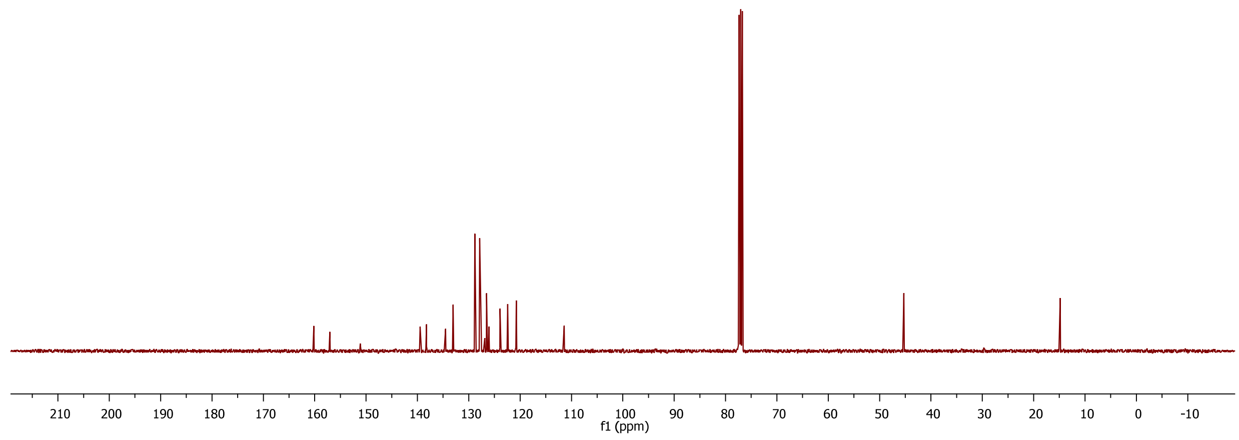

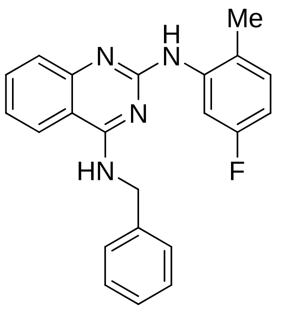

KSC-16-108

***N*[4]-Benzyl-*N*[2]-(5-fluoro-2-methylphenyl)quinazoline-2,4-diamine (S82)**. Yield: 12.7 mg, 96%. ^{1}H NMR (400 MHz, $CDCl_3$) δ 8.30 (dd, *J* = 2.6, 12.1 Hz, 1H), 7.54 (d, *J* = 3.6 Hz, 2H), 7.49 (d, *J* = 8.2 Hz, 1H), 7.39 – 7.20 (m, 5H), 7.11 (dt, *J* = 4.1, 8.2 Hz, 1H), 7.06 – 6.94 (m, 1H), 6.83 (s, br. 1H), 6.56 (td, *J* = 2.7, 8.2 Hz, 1H), 5.87 (s, br. 1H), 4.76 (d, *J* = 5.2 Hz, 2H), 2.23 (s, 3H). ^{13}C NMR (101 MHz, $CDCl_3$) δ 162.8, 160.5, 160.1, 156.6, 151.1, 139.6, 139.4, 138.1, 133.1, 130.7, 130.6, 128.9, 128.0, 127.8, 126.4, 122.6, 121.3, 120.7, 111.5, 108.3, 108.0, 107.4, 107.1, 45.4, 17.5. HRMS (m/z): calcd for $C_{22}H_{20}FN_4$ (M+H) 359.1672; found 359.1678.

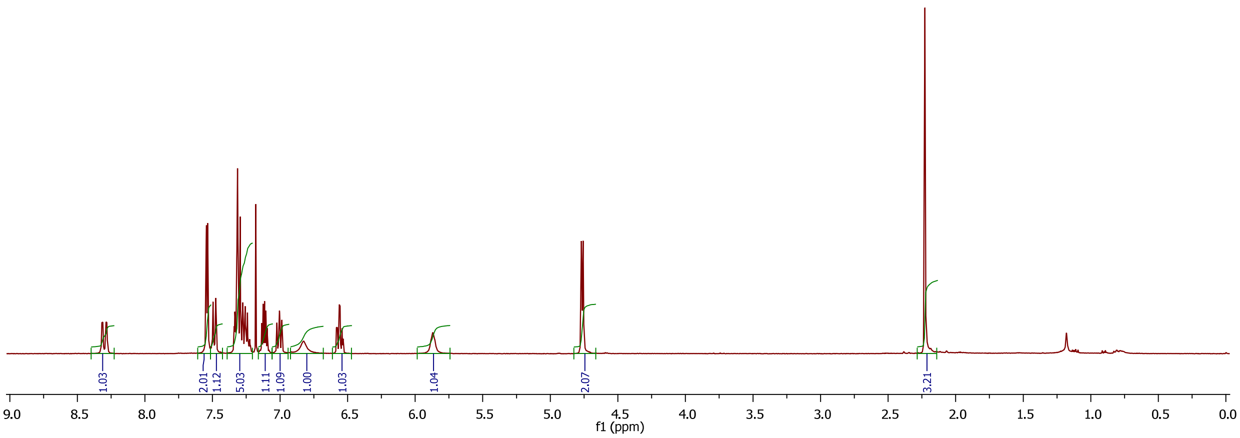

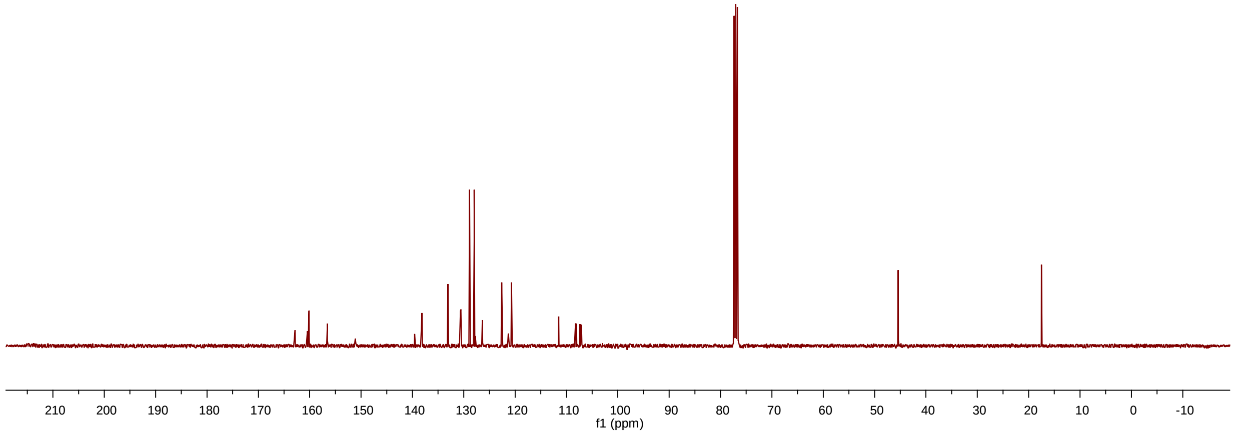

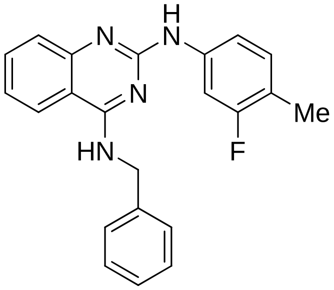

KSC-16-109

N^4-Benzyl-N^2-(3-fluoro-4-methylphenyl)quinazoline-2,4-diamine (S83). Yield: 13.0 mg, 98%. ^{1}H NMR (400 MHz, $CDCl_3$) δ 7.78 (dd, J = 2.1, 12.4, 1H), 7.64 – 7.55 (m, 2H), 7.51 (d, J = 8.1, 1H), 7.43 – 7.25 (m, 5H), 7.20 – 7.12 (m, 1H), 7.12 – 6.92 (m, 3H), 5.87 (s, br. 1H), 4.81 (d, J = 5.2 Hz, 2H), 2.20 (d, J = 1.6 Hz, 3H). ^{13}C NMR (101 MHz, $CDCl_3$) δ 162.5, 160.11, 160.08, 156.6, 151.4, 139.6, 139.5, 138.2, 133.0, 131.02, 130.95, 128.9, 127.9, 127.7, 126.5, 122.4, 120.7, 117.6, 117.4, 114.14, 114.11, 111.5, 106.3, 106.1, 45.4, 14.02, 13.99. HRMS (m/z): calcd for $C_{22}H_{20}FN_4$ (M+H) 359.1672; found 359.1673.

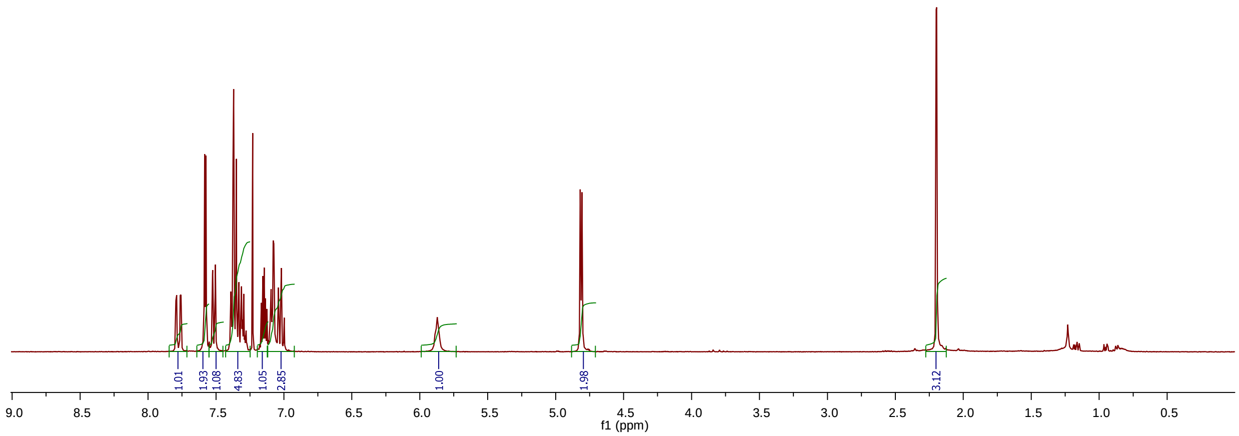

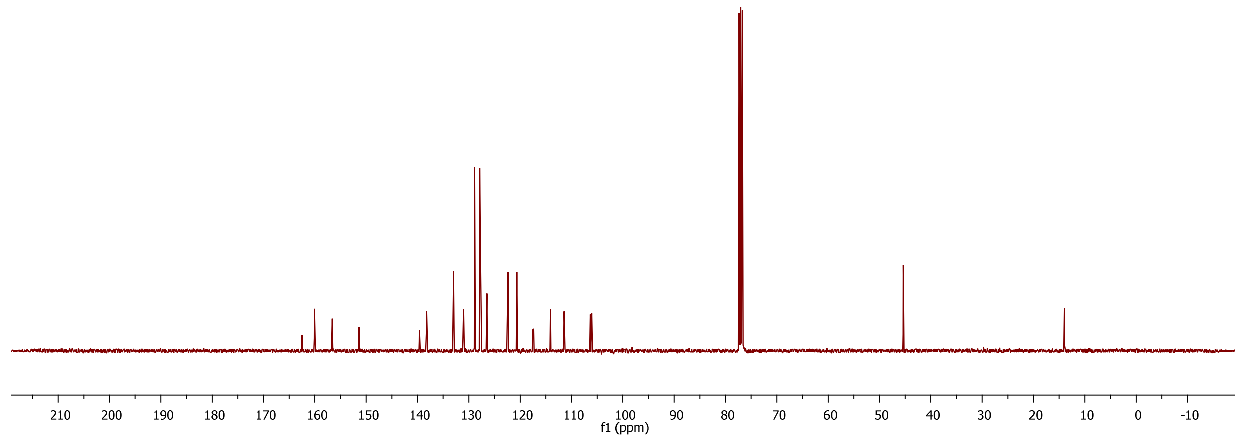

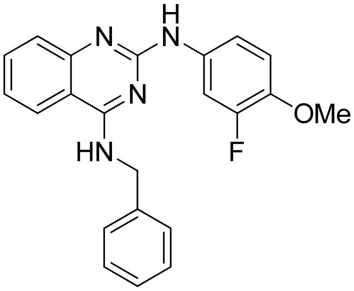

KSC-16-110

***N*^{4}-Benzyl-*N*^{2}-(3-fluoro-4-methoxyphenyl)quinazoline-2,4-diamine (S84)**. Yield: 13.0 mg, 94%. ^{1}H NMR (400 MHz, $CDCl_3$) δ 7.76 (dd, J = 2.6, 13.8 Hz, 1H), 7.59 – 7.41 (m, 3H), 7.39 – 7.20 (m, 5H), 7.16 – 7.02 (m, 2H), 6.95 (s, br. 1H), 6.81 (t, J = 9.1 Hz, 1H), 5.83 (s, br. 1H), 4.75 (d, J = 5.2 Hz, 2H), 3.79 (s, 3H). ^{13}C NMR (101 MHz, $CDCl_3$) δ 160.1, 156.7, 153.6, 151.4, 151.2, 142.6, 142.4, 138.2, 134.4, 134.3, 133.0, 128.9, 127.9, 127.7, 126.4, 122.3, 120.7, 114.4, 114.4, 114.1, 114.1, 111.5, 108.5, 108.3, 56.9, 45.3. HRMS (m/z): calcd for $C_{22}H_{20}FN_4O$ (M+H) 375.1621; found 375.1623.

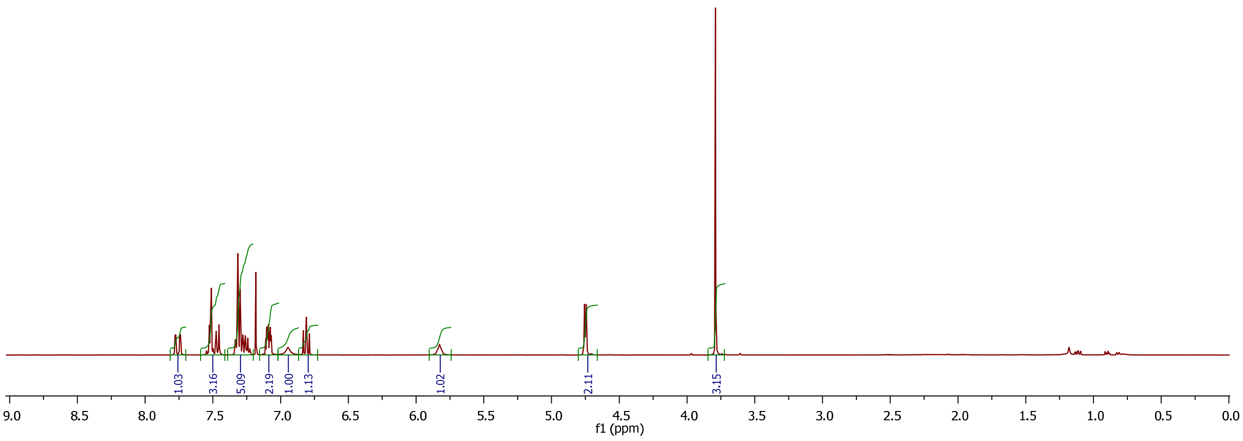

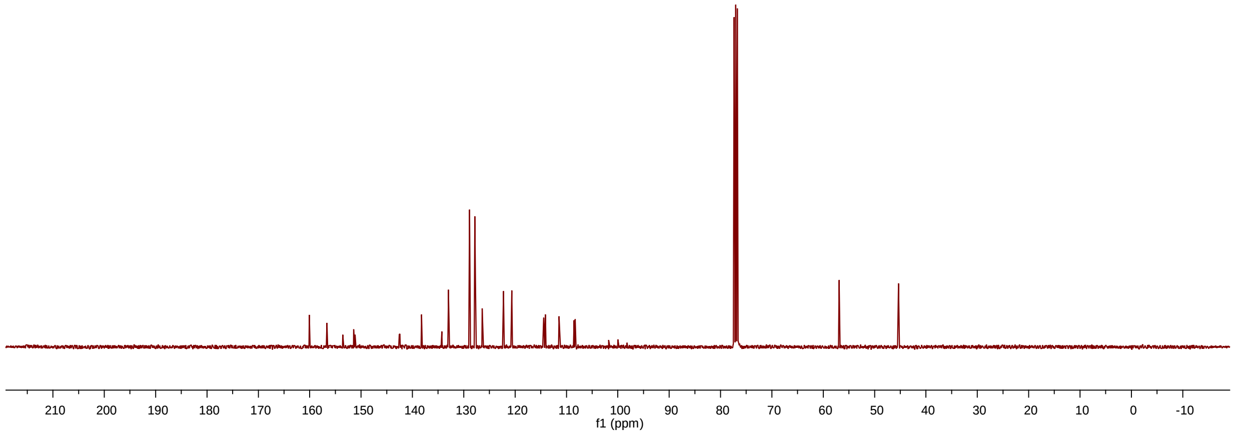

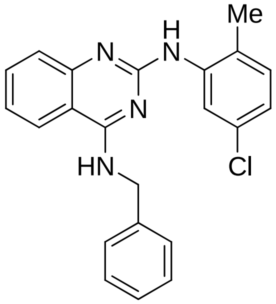

KSC-16-112

***N*[4]-Benzyl-*N*[2]-(5-chloro-2-methylphenyl)quinazoline-2,4-diamine (S85)**. Yield: 10.6 mg, 76%. ^{1}H NMR (400 MHz, $CDCl_3$) δ 8.48 (d, *J* = 2.1 Hz, 1H), 7.61 – 7.44 (m, 3H), 7.38 – 7.21 (m, 5H), 7.12 (ddd, *J* = 2.8, 5.3, 8.2 Hz, 1H), 7.01 (d, *J* = 8.1 Hz, 1H), 6.85 (dd, *J* = 2.2, 8.0 Hz, 2H), 5.88 (s, br. 1H), 4.77 (d, *J* = 5.1 Hz, 2H), 2.24 (s, 3H). ^{13}C NMR (101 MHz, $CDCl_3$) δ 160.2, 156.6, 139.3, 138.1, 133.1, 131.8, 131.0, 128.9, 128.0, 127.8, 126.2, 124.9, 122.6, 121.9, 120.7, 120.5, 111.5, 45.4, 17.7. HRMS (m/z): calcd for $C_{22}H_{20}ClN_4$ (M+H) 375.1376; found 375.1379.

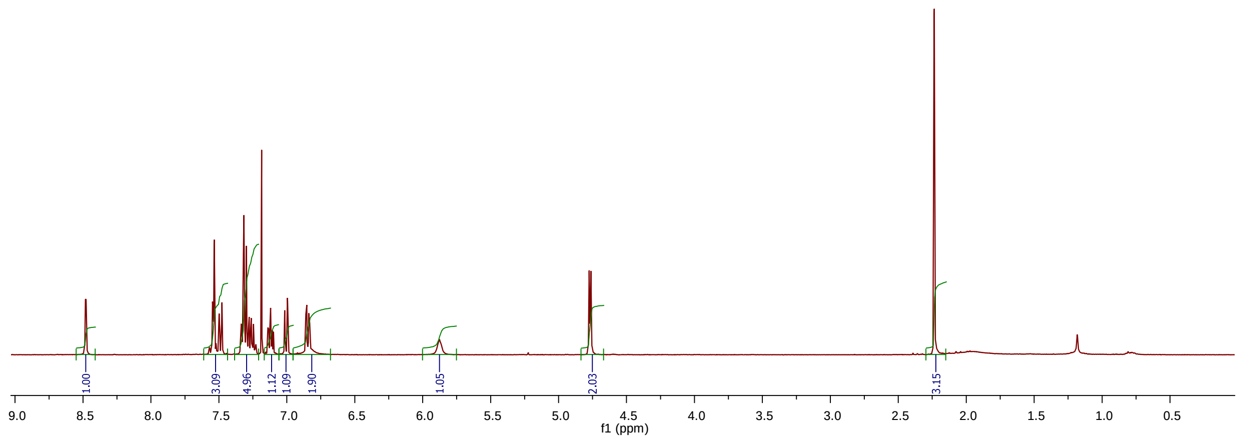

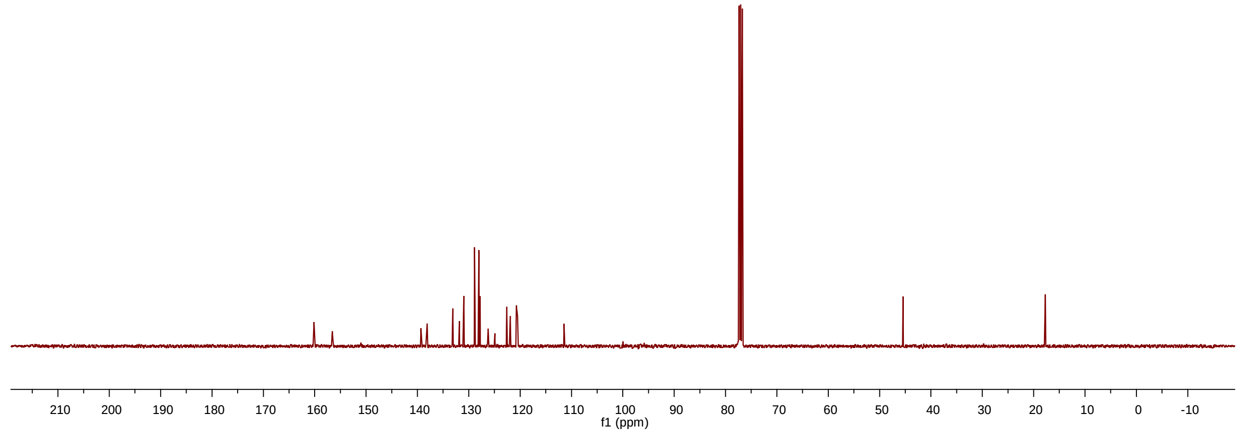

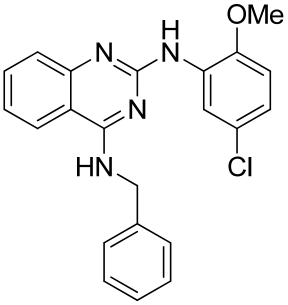

KSC-16-113

***N*^{4}-Benzyl-*N*^{2}-(5-chloro-2-methoxyphenyl)quinazoline-2,4-diamine (S86)**. Yield: 11.0 mg, 76%. ^{1}H NMR (400 MHz, $CDCl_3$) δ 8.83 (s, 1H), 7.75 – 7.41 (m, 4H), 7.41 – 7.21 (m, 5H), 7.11 (ddd, *J* = 1.7, 6.5, 8.2 Hz, 1H), 6.80 (dd, *J* = 2.6, 8.6 Hz, 1H), 6.69 (d, *J* = 8.6 Hz, 1H), 5.87 (s, br. 1H), 4.81 (d, *J* = 5.3 Hz, 2H), 3.81 (s, 3H). ^{13}C NMR (101 MHz, $CDCl_3$) δ 160.1, 156.3, 146.3, 138.2, 133.0, 131.0, 128.9, 128.0, 127.8, 126.4, 125.9, 122.6, 120.7, 120.2, 118.5, 111.4, 110.4, 56.0, 45.5. HRMS (m/z): calcd for $C_{22}H_{20}ClN_4O$ (M+H) 391.1326; found 391.1324.

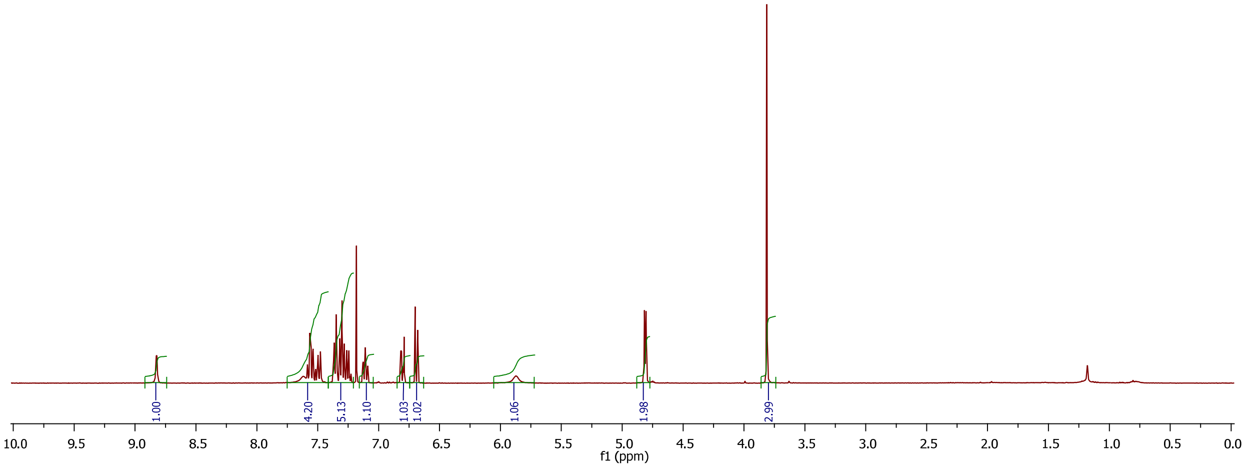

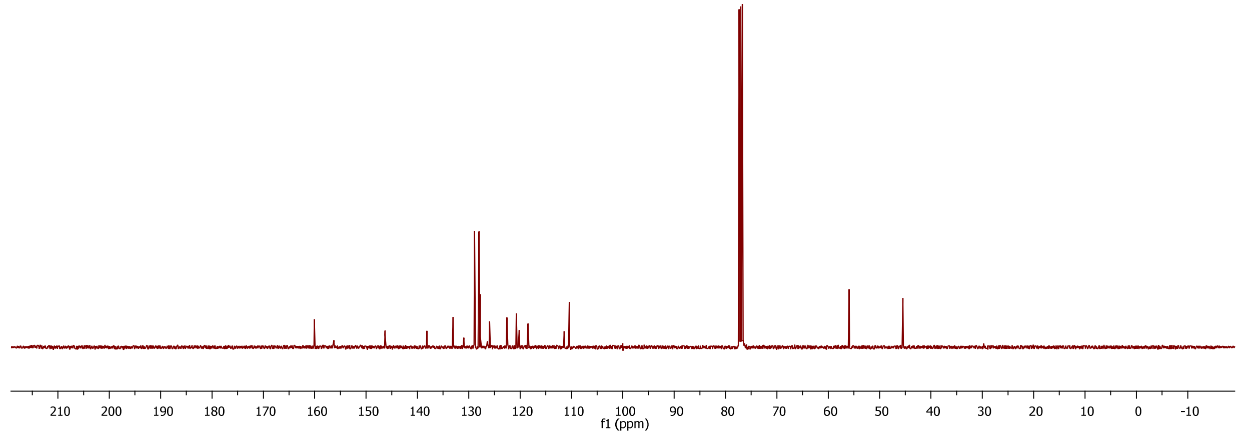

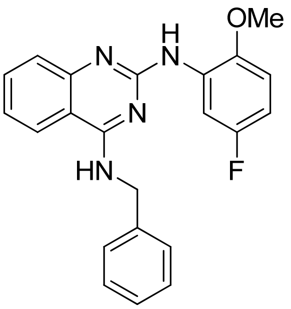

KSC-16-120

***N*^{4}-Benzyl-*N*^{2}-(5-fluoro-2-methoxyphenyl)quinazoline-2,4-diamine (S87)**. Yield: 13.0 mg, 94%. ^{1}H NMR (400 MHz, $CDCl_3$) δ 8.75 – 8.57 (m, 1H), 7.71 – 7.52 (m, 3H), 7.40 (qdd, *J* = 5.5, 8.0, 13.6 Hz, 6H), 7.18 (ddd, *J* = 3.3, 4.8, 8.2 Hz, 1H), 6.76 – 6.60 (m, 2H), 5.91 (s, br. 1H), 4.88 (d, *J* = 5.4 Hz, 2H), 3.91 (s, 3H). ^{13}C NMR (101 MHz, $CDCl_3$) δ 160.0, 158.9, 156.8, 156.5, 151.6, 148.8, 148.7, 138.4, 132.9, 128.8, 127.9, 127.7, 126.5, 126.14, 126.11, 122.1, 120.7, 119.3, 119.2, 111.4, 106.4, 106.1, 98.6, 98.4, 55.9, 45.4. HRMS (m/z): calcd for $C_{22}H_{20}FN_4O$ (M+H) 375.1621; found 375.1622.

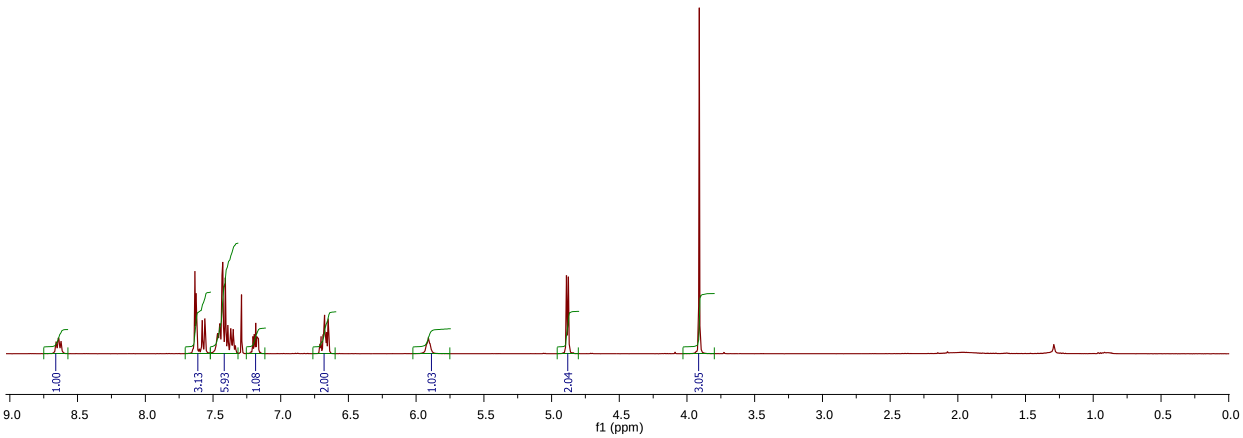

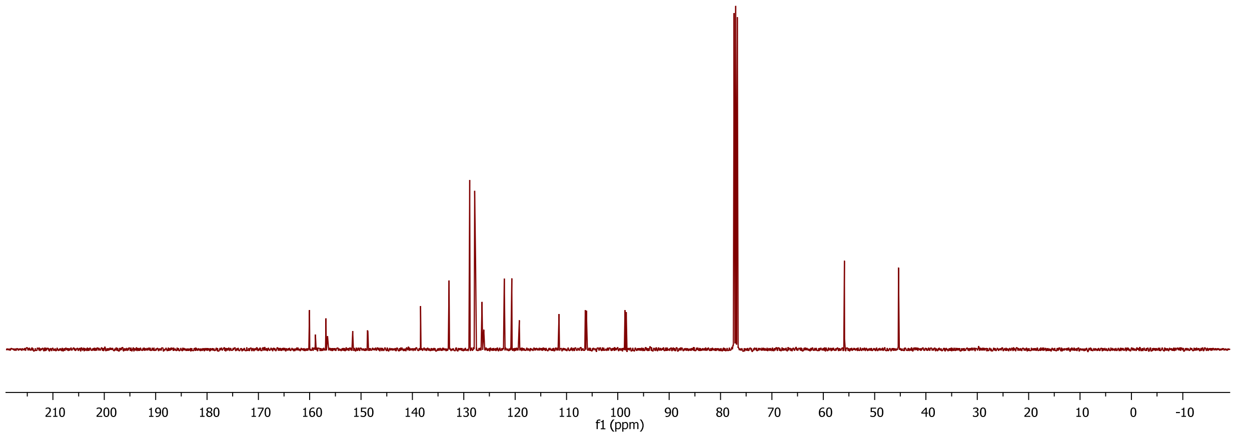

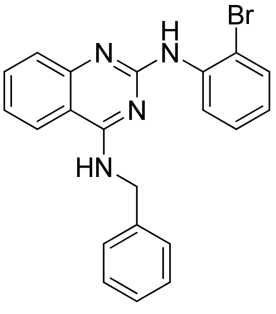

KSC-1-288

***N*[4]-Benzyl-*N*[2]-(2-bromophenyl)quinazoline-2,4-diamine (S88)**. Yield: 18.0 mg, 60%. ^{1}H NMR (400 MHz, $CDCl_3$) δ 8.67 (dd, J = 1.5, 8.3 Hz, 1H), 7.59 – 7.51 (m, 2H), 7.51 – 7.44 (m, 2H), 7.41 (s, br. 1H), 7.37 – 7.28 (m, 4H), 7.28 – 7.19 (m, 2H), 7.13 (ddd, J = 2.3, 5.8, 8.2 Hz, 1H), 6.81 – 6.73 (m, 1H), 5.81 (s, br. 1H), 4.78 (d, J = 5.4 Hz, 2H). ^{13}C NMR (101 MHz, $CDCl_3$) δ 160.1, 156.5, 151.4, 138.3, 138.1, 132.9, 132.2, 128.9, 128.0, 127.9, 127.7, 126.7, 122.7, 122.4, 120.7, 120.6, 112.8, 111.7, 45.4. HRMS (m/z): calcd for $C_{21}H_{18}BrN_4$ (M+H) 405.0715 and 407.0694; found 407.0688.

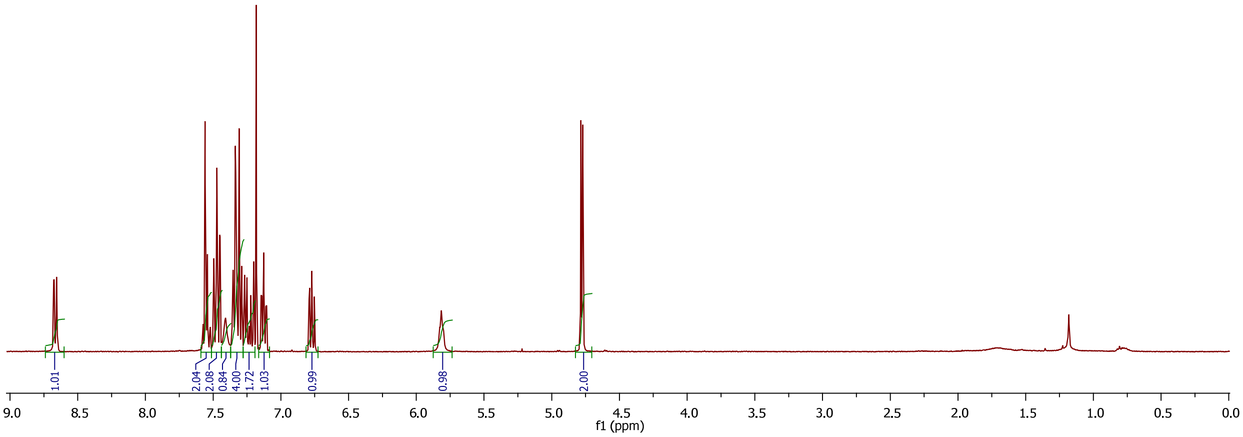

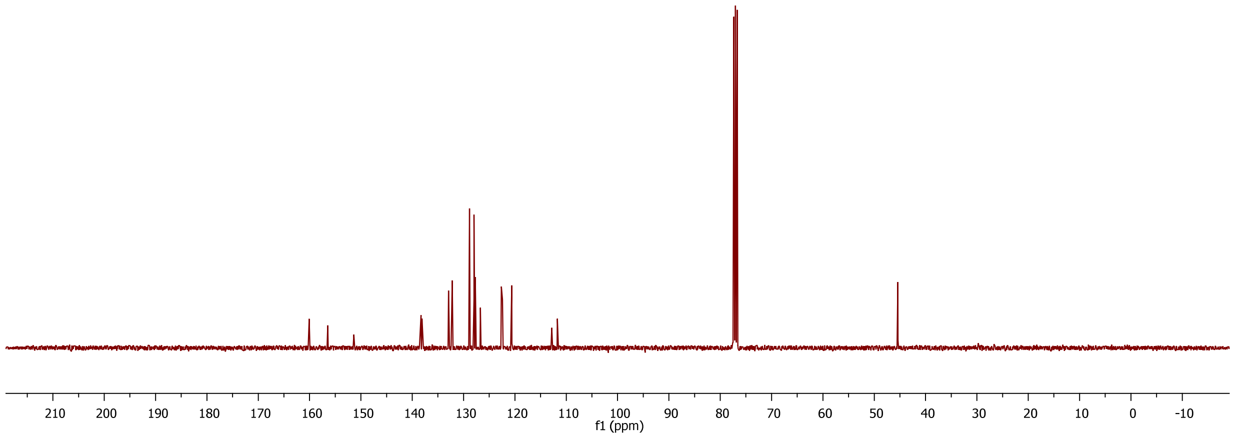

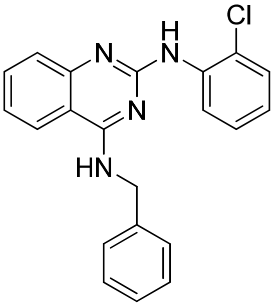

KSC-1-289

***N^4*-Benzyl-*N^2*-(2-chlorophenyl)quinazoline-2,4-diamine (S89)**. Yield: 39.0 mg, 86%. ^{1}H NMR (400 MHz, $CDCl_3$) δ 8.82 (dd, *J* = 1.5, 8.4 Hz, 1H), 7.72 – 7.62 (m, 2H), 7.59 (d, *J* = 8.1 Hz, 1H), 7.51 (s, br. 1H), 7.48 – 7.32 (m, 6H), 7.28 – 7.20 (m, 2H), 6.98 – 6.90 (m, 1H), 5.91 (s, br. 1H), 4.89 (d, *J* = 5.4 Hz, 2H). ^{13}C NMR (101 MHz, $CDCl_3$) δ 160.1, 156.4, 151.4, 138.3, 137.0, 132.9, 128.93, 128.87, 128.0, 127.7, 127.3, 126.8, 122.7, 121.9, 121.8, 120.6, 120.3, 111.7, 45.4. HRMS (m/z): calcd for $C_{21}H_{18}ClN_4$ (M+H) 361.1220; found 361.1213.

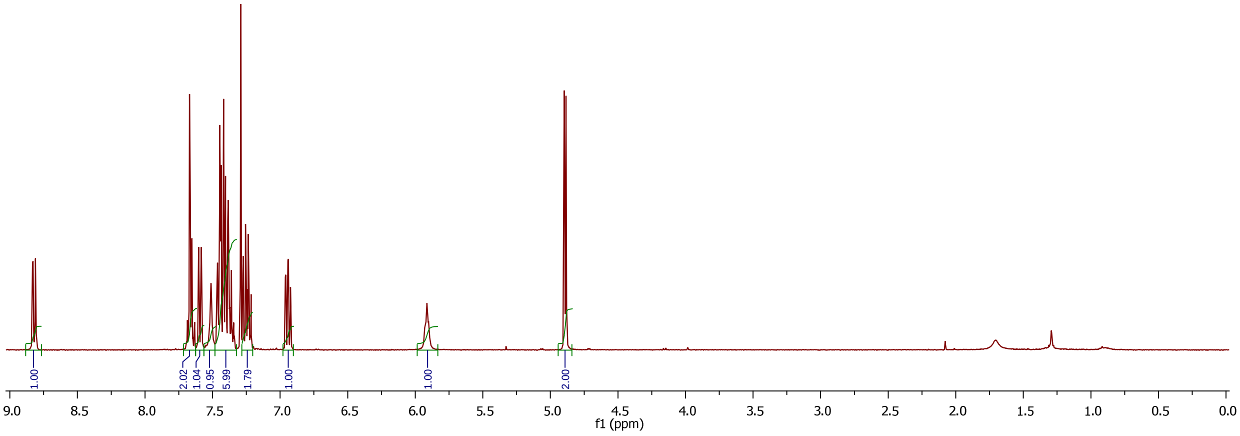

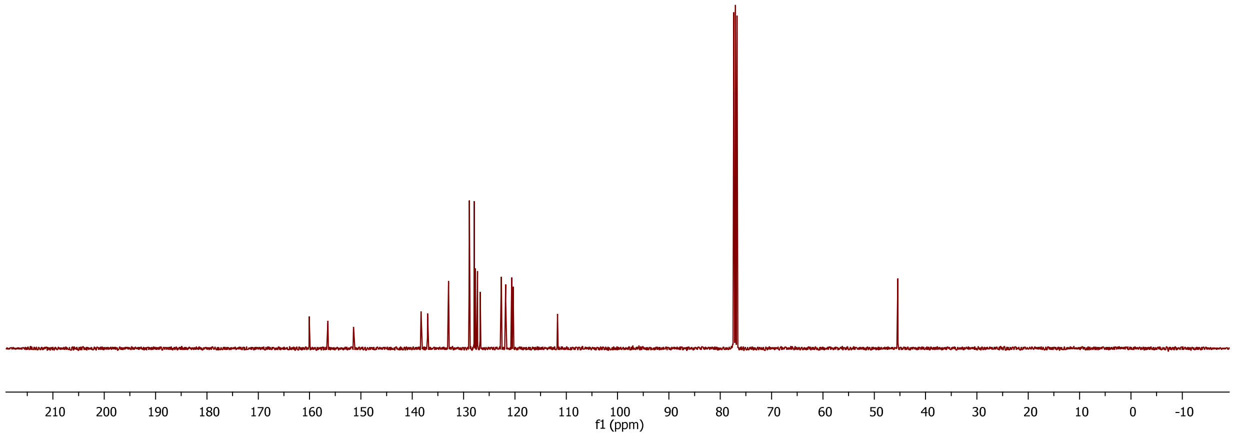

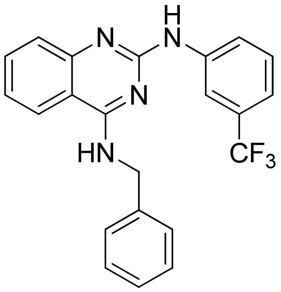

KSC-16-150

***N*[4]-Benzyl-*N*[2]-(3-(trifluoromethyl)phenyl)quinazoline-2,4-diamine (S90)**. Yield: 14.6 mg, 100%. ^{1}H NMR (400 MHz, $CDCl_3$) δ 8.34 (s, 1H), 7.77 (d, J = 8.2 Hz, 1H), 7.71 – 7.54 (m, 3H), 7.54 – 7.31 (m, 7H), 7.24 (ddd, J = 2.4, 5.0, 8.2 Hz, 2H), 5.95 (s, br. 1H), 4.89 (d, J = 5.3 Hz, 2H). ^{13}C NMR (101 MHz, $CDCl_3$) δ 160.2, 156.5, 151.3, 140.9, 138.0, 133.1, 131.5, 131.2, 130.9, 130.5, 129.1, 128.9, 127.9, 127.8, 126.5, 125.7, 123.0, 122.7, 121.7, 120.7, 118.1, 118.04, 118.00, 117.97, 115.7, 115.66, 115.62, 115.58, 111.6, 45.5. HRMS (m/z): calcd for $C_{22}H_{18}F_3N_4$ (M+H) 395.1484; found 395.1481.

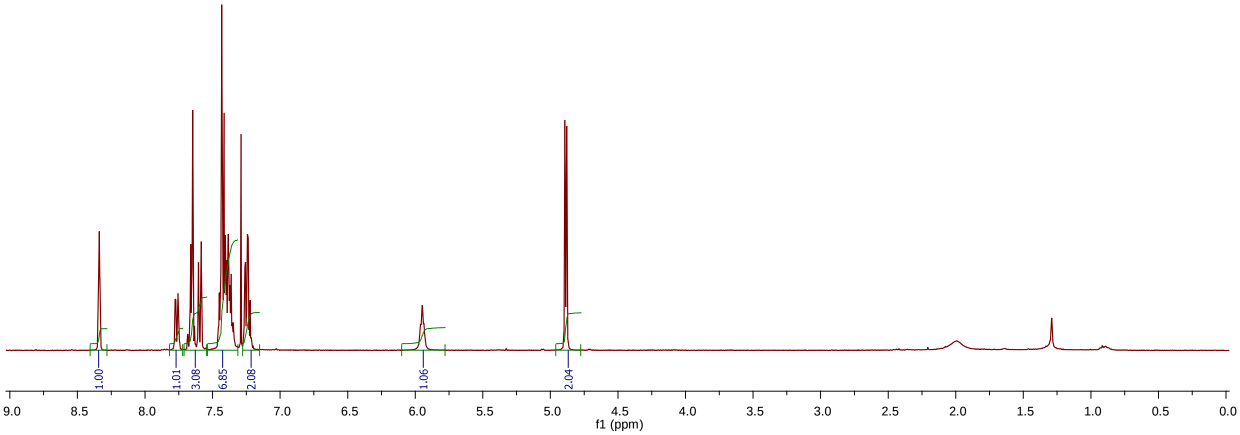

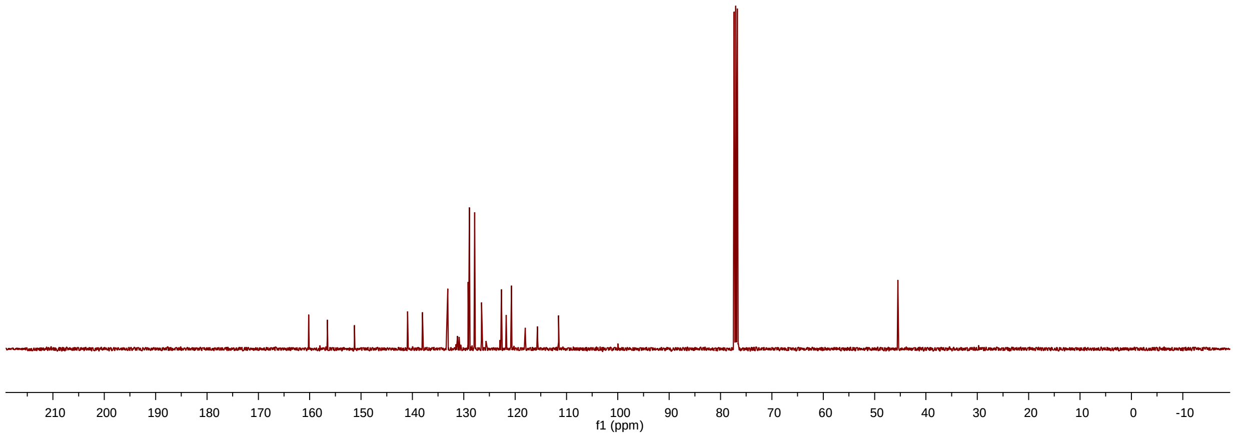

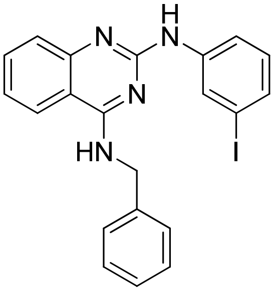

KSC-16-152

***N*[4]-Benzyl-*N*[2]-(3-iodophenyl)quinazoline-2,4-diamine (S91)**. Yield: 16.0 mg, 95%. ^{1}H NMR (400 MHz, $CDCl_3$) δ 8.38 (t, *J* = 1.9 Hz, 1H), 7.62 (tdd, *J* = 4.1, 5.9, 9.2 Hz, 4H), 7.50 – 7.31 (m, 6H), 7.22 (ddd, *J* = 3.0, 5.2, 8.2 Hz, 2H), 7.02 (t, *J* = 8.0 Hz, 1H), 5.95 (s, 1H), 4.88 (d, *J* = 5.3 Hz, 2H). ^{13}C NMR (101 MHz, $CDCl_3$) δ 160.1, 156.5, 151.3, 141.6, 138.1, 133.1, 130.5, 130.2, 128.9, 127.9, 127.8, 127.7, 126.5, 122.6, 120.7, 118.1, 111.5, 94.3, 45.5. HRMS (m/z): calcd for $C_{21}H_{18}IN_4$ (M+H) 453.0576; found 453.0571.

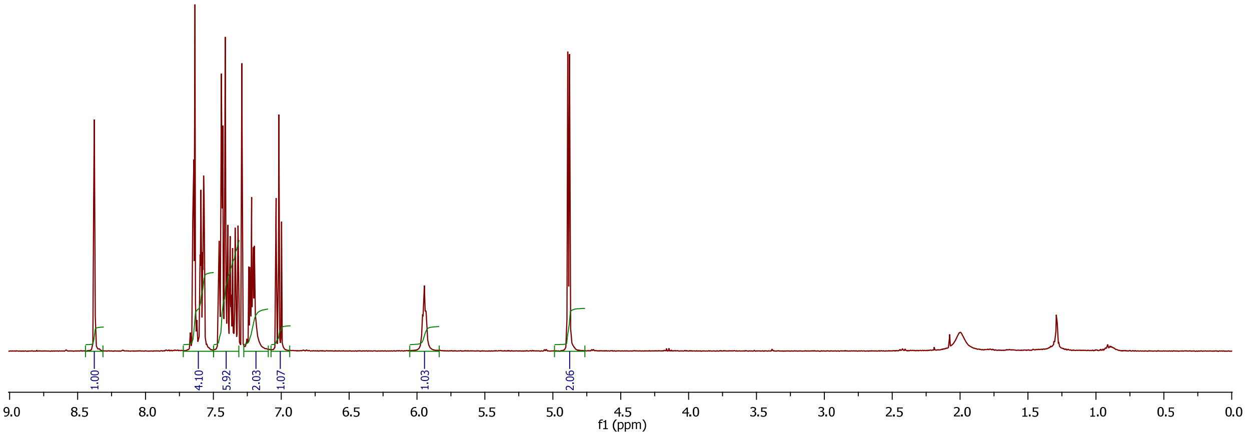

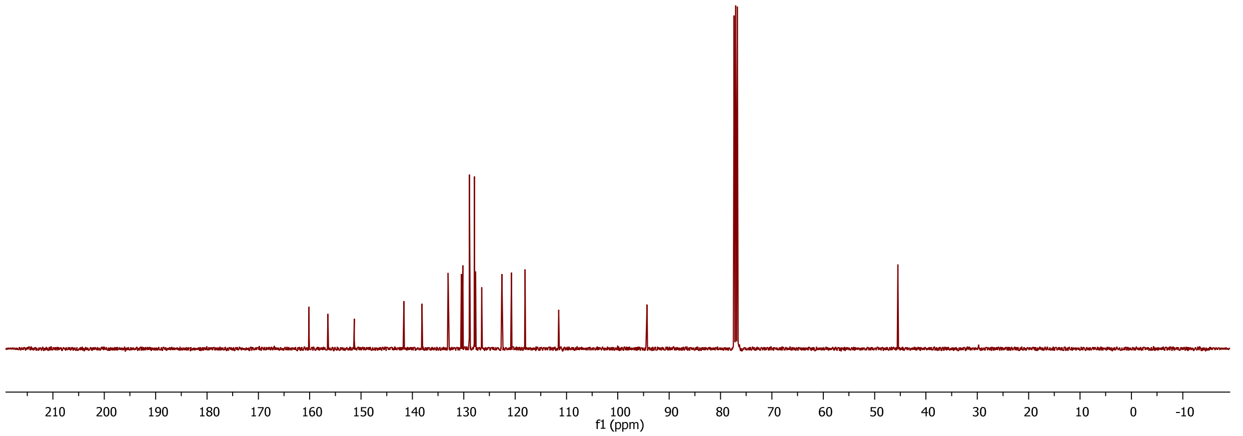

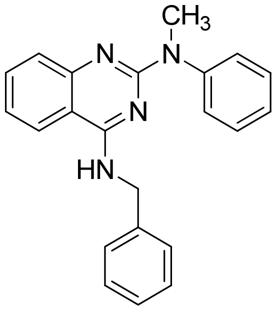

KSC-16-188

***N*[4]-Benzyl-*N*[2]-methyl-*N*[2]-phenylquinazoline-2,4-diamine (S92)**. Yield: 7.7 mg, 61%. ^{1}H NMR (400 MHz, $CDCl_3$) δ 7.36 (dd, J = 1.3, 4.6 Hz, 2H), 7.31 (t, J = 7.8 Hz, 1H), 7.26 – 7.09 (m, 7H), 7.05 – 7.00 (m, 2H), 7.00 – 6.94 (m, 1H), 6.90 (ddd, J = 3.3, 4.9, 8.2 Hz, 1H), 5.58 (s, 1H), 4.38 (d, J = 5.6 Hz, 2H), 3.45 (s, 3H). ^{13}C NMR (101 MHz, $CDCl_3$) δ 159.2, 158.9, 152.2, 146.3, 138.9, 132.5, 128.6, 128.4, 128.0, 127.3, 126.6, 126.5, 124.6, 121.3, 120.6, 110.9, 45.0, 38.2. HRMS (m/z): calcd for $C_{22}H_{21}N_4$ (M+H) 341.1766; found 341.1766.

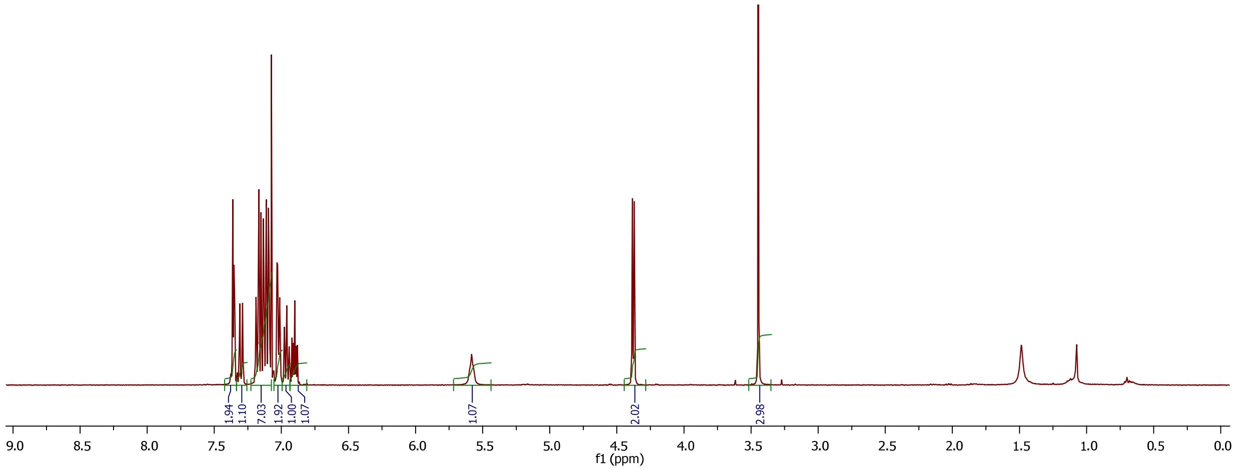

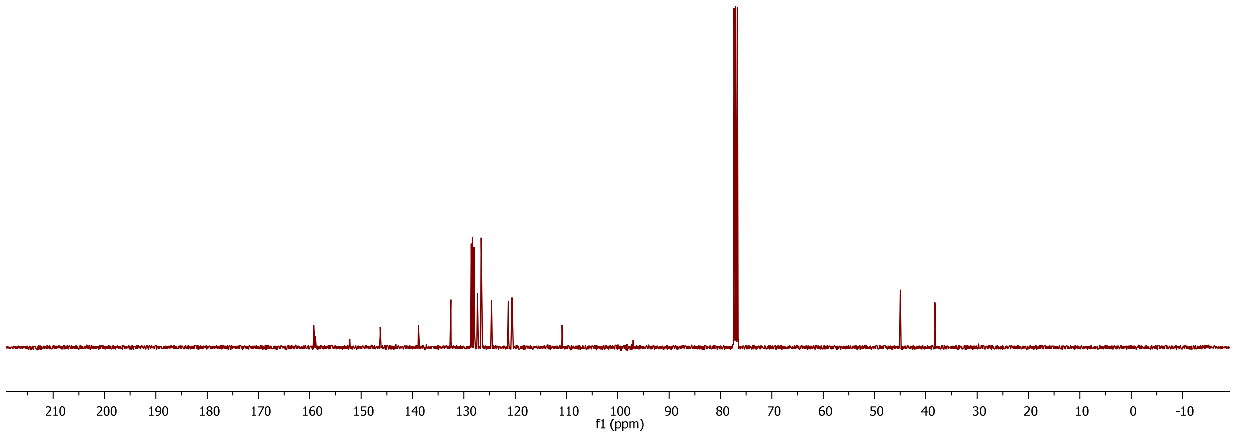

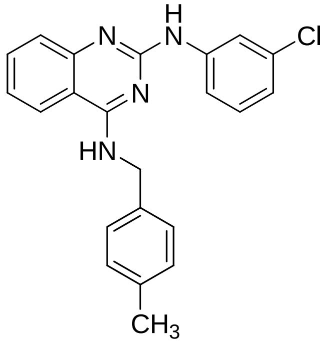

KSC-16-72

***N*^{2}-(3-Chlorophenyl)-*N*^{4}-(4-methylbenzyl)quinazoline-2,4-diamine (S93)**. Yield: 13.0 mg, 98%. ^{1}H NMR (400 MHz, $CDCl_3$) δ 8.08 (t, *J* = 2.0 Hz, 1H), 7.65 (d, *J* = 3.5 Hz, 2H), 7.57 (d, *J* = 8.2 Hz, 1H), 7.53 – 7.46 (m, 1H), 7.34 (d, *J* = 8.0 Hz, 2H), 7.25 – 7.20 (m, 4H), 7.06 (s, br. 1H), 6.98 (ddd, *J* = 0.9, 2.0, 7.9 Hz, 1H), 5.86 (s, br. 1H), 4.83 (d, *J* = 5.3 Hz, 2H), 2.40 (s, 3H). ^{13}C NMR (101 MHz, $CDCl_3$) δ 160.1, 156.5, 151.3, 141.7, 137.6, 135.1, 134.4, 133.0, 129.7, 129.6, 127.9, 126.6, 122.6, 121.5, 120.7, 118.8, 116.8, 111.6, 45.2, 21.2. HRMS (m/z): calcd for $C_{22}H_{20}ClN_4$ (M+H) 375.1376; found 375.1376.

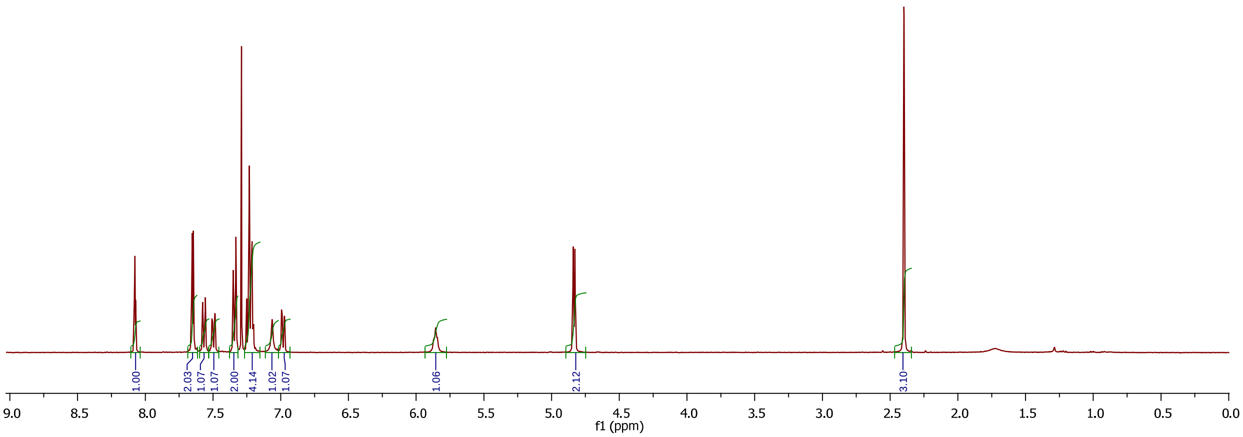

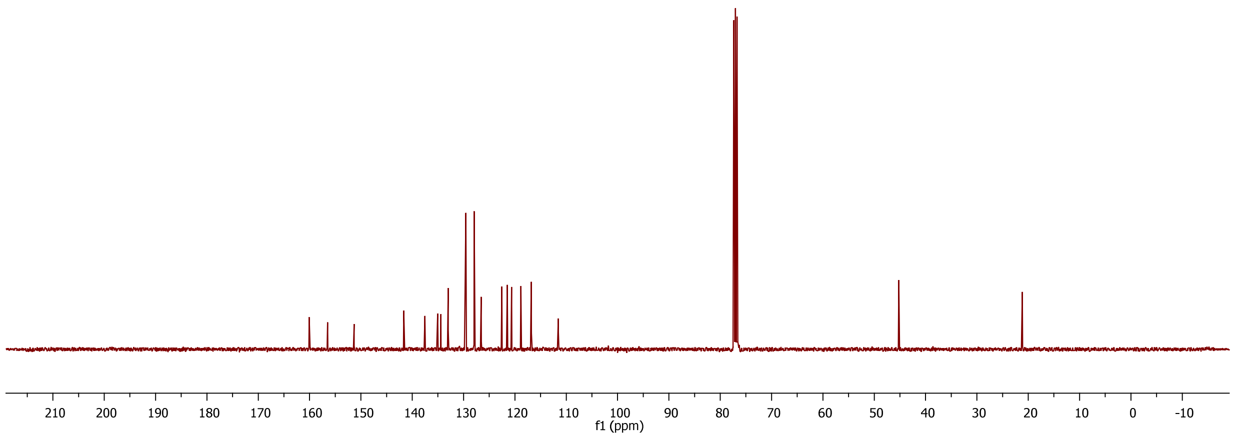

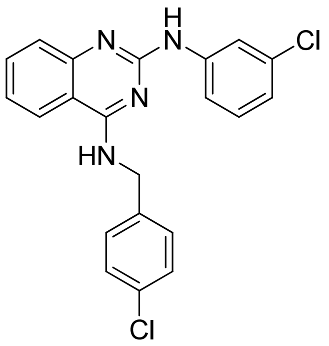

KSC-16-89

***N*^{4}-(4-Chlorobenzyl)-*N*^{2}-(3-chlorophenyl)quinazoline-2,4-diamine (S94)**. Yield: 13.0 mg, 100%. ^{1}H NMR (400 MHz, $CDCl_3$) δ 8.00 (t, *J* = 2.0 Hz, 1H), 7.63 – 7.61 (m, 2H), 7.55 (d, *J* = 8.2, 1H), 7.45 – 7.38 (m, 1H), 7.33 (s, 4H), 7.22 – 7.19 (m, 1H), 7.17 (d, *J* = 8.1 Hz, 1H), 7.03 (s, br. 1H), 6.94 (dd, *J* = 1.1, 7.9 Hz, 1H), 5.90 (s, br. 1H), 4.82 (d, *J* = 5.6 Hz, 2H). ^{13}C NMR (101 MHz, $CDCl_3$) δ 160.1, 156.4, 151.3, 141.5, 136.7, 134.4, 133.5, 133.2, 129.7, 129.1, 129.0, 126.6, 122.7, 121.6, 120.6, 118.9, 116.9, 111.4, 44.6. HRMS (m/z): calcd for $C_{21}H_{17}Cl_2N_4$ (M+H) 395.0830; found 395.0826.

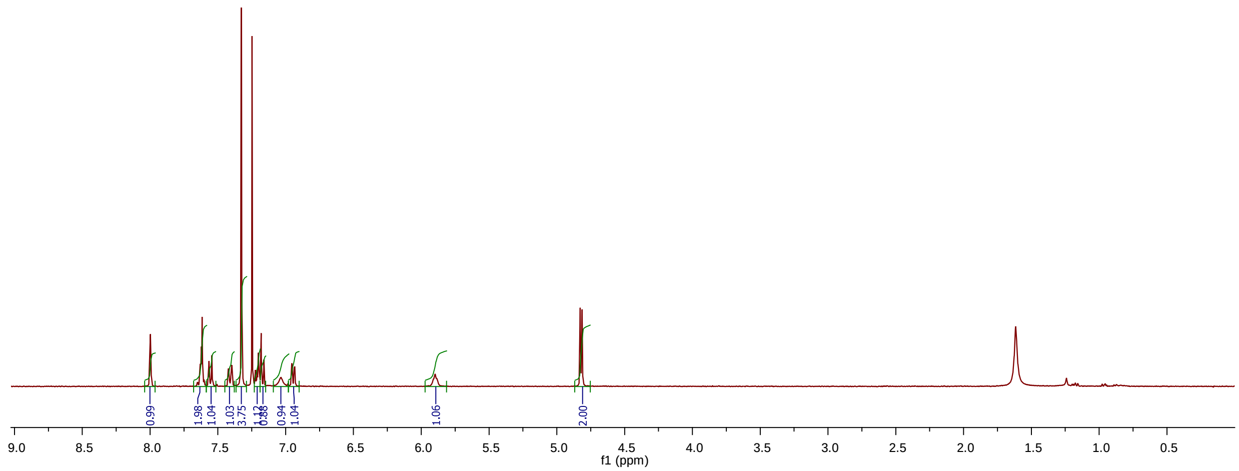

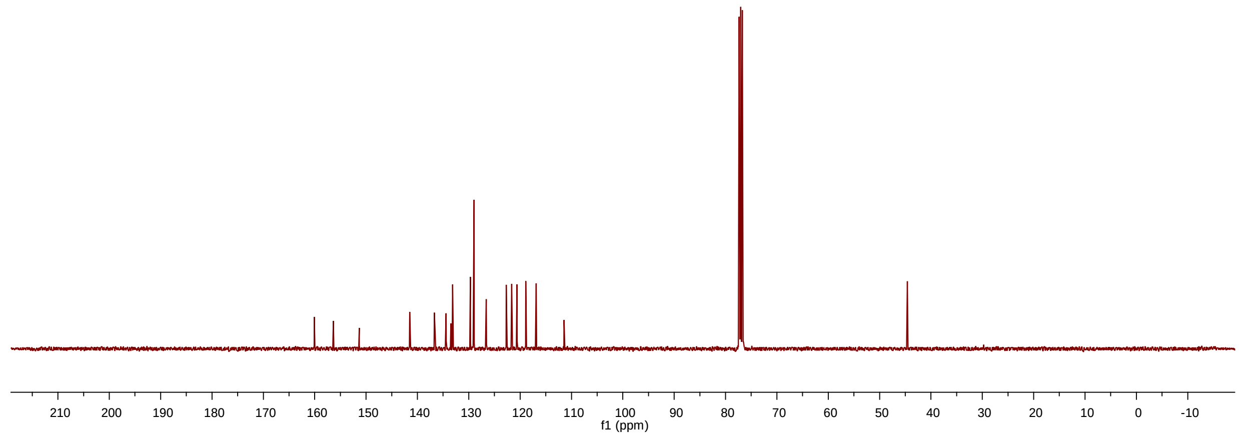

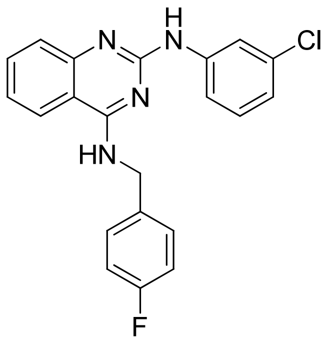

KSC-16-98

N[2]**-(3-Chlorophenyl)-*N***[4]**-(4-fluorobenzyl)quinazoline-2,4-diamine (S95 or 12)**. Yield: 13.0 mg, 99%. ^{1}H NMR (400 MHz, CDCl3) δ 8.00 (t, J = 2.0 Hz, 1H), 7.65 – 7.57 (m, 2H), 7.54 (d, J = 8.2 Hz, 1H), 7.44 – 7.38 (m, 1H), 7.35 (dd, J = 5.3, 8.7 Hz, 2H), 7.22 – 7.14 (m, 2H), 7.09 (s, br. 1H), 7.06 – 6.99 (m, 2H), 6.93 (ddd, J = 0.9, 2.0, 7.9 Hz, 1H), 5.89 (s, br. 1H), 4.80 (d, J = 5.2 Hz, 2H). ^{13}C NMR (101 MHz, CDCl3) δ 163.6, 161.1, 160.1, 156.4, 151.3, 141.6, 134.4, 133.93, 133.90, 133.1, 130.0, 129.54, 129.46, 126.6, 122.7, 121.6, 120.7, 118.9, 116.9, 115.8, 115.6, 111.5, 44.6. HRMS (m/z): calcd for $C_{21}H_{17}ClFN_4$ (M+H) 379.1126; found 379.1123.

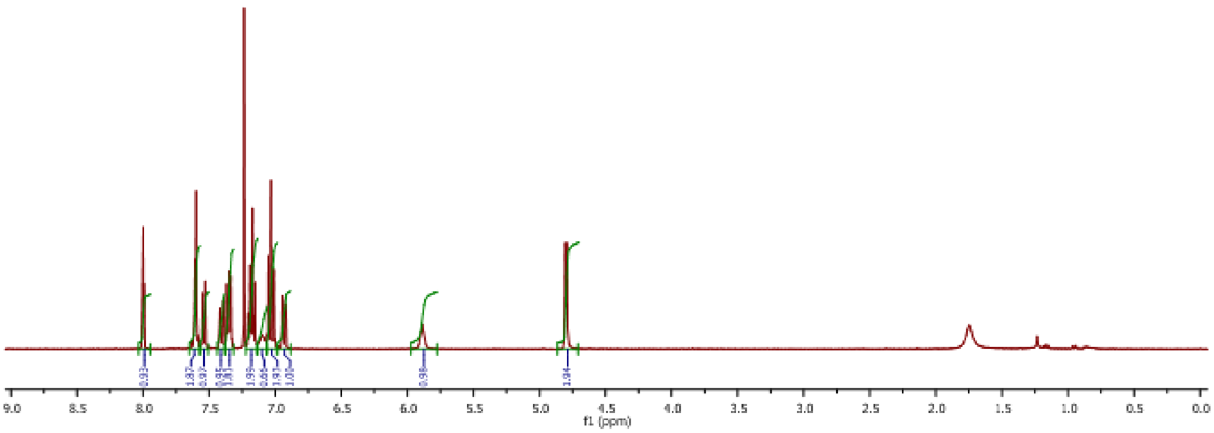

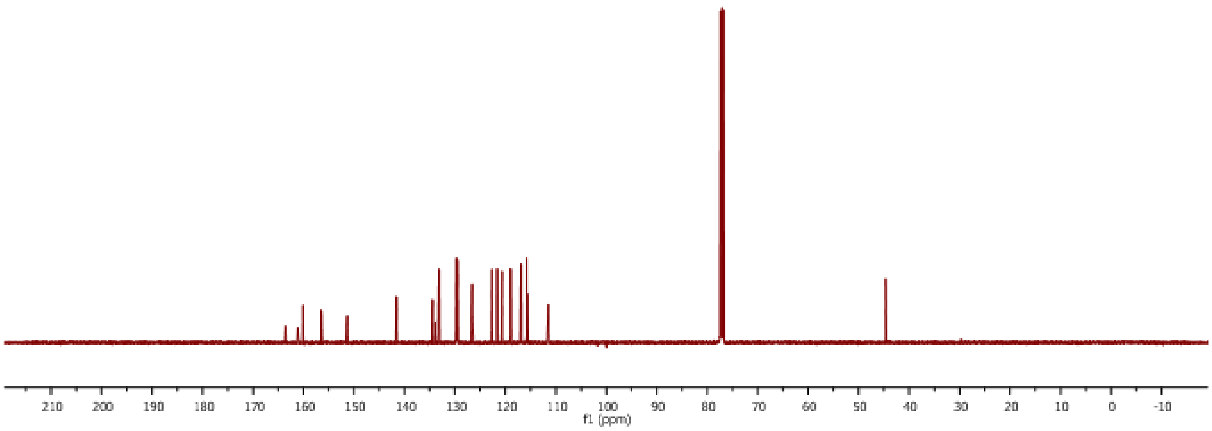

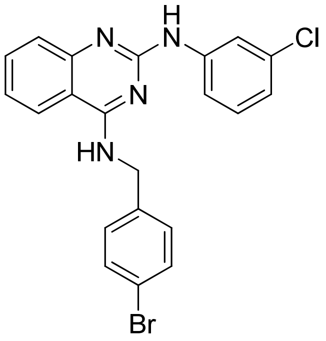

KSC-16-101

***N*[4]-(4-Bromobenzyl)-*N*[2]-(3-chlorophenyl)quinazoline-2,4-diamine (S96)**. Yield: 12.5 mg, 99%. ^{1}H NMR (400 MHz, $CDCl_3$) δ 8.04 (t, *J* = 2.0 Hz, 1H), 7.72 – 7.63 (m, 2H), 7.60 (d, *J* = 8.2 Hz, 1H), 7.57 – 7.48 (m, 2H), 7.48 – 7.40 (m, 1H), 7.31 (d, *J* = 8.5 Hz, 2H), 7.27 – 7.20 (m, 2H), 7.05 (s, br. 1H), 7.02 – 6.90 (m, 1H), 5.94 (s, br. 1H), 4.85 (d, *J* = 5.6 Hz, 2H). ^{13}C NMR (101 MHz, $CDCl_3$) δ 160.1, 156.4, 151.4, 141.5, 137.3, 134.4, 133.2, 131.9, 129.7, 129.4, 126.7, 122.7, 121.6, 121.5, 120.6, 118.9, 116.9, 111.4, 44.6. HRMS (m/z): calcd for $C_{21}H_{17}BrClN_4$ (M+H) 439.0325 and 441.0305; found 441.0296.

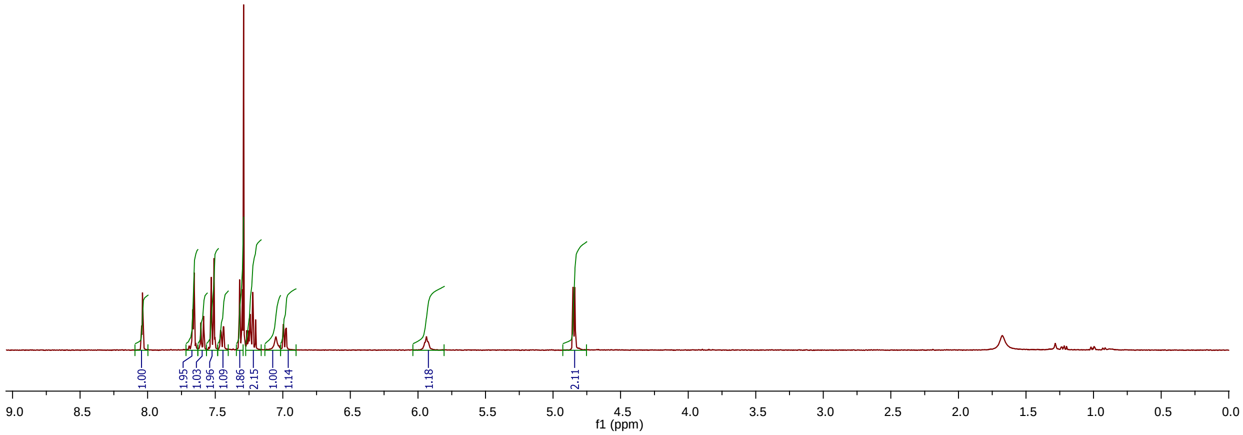

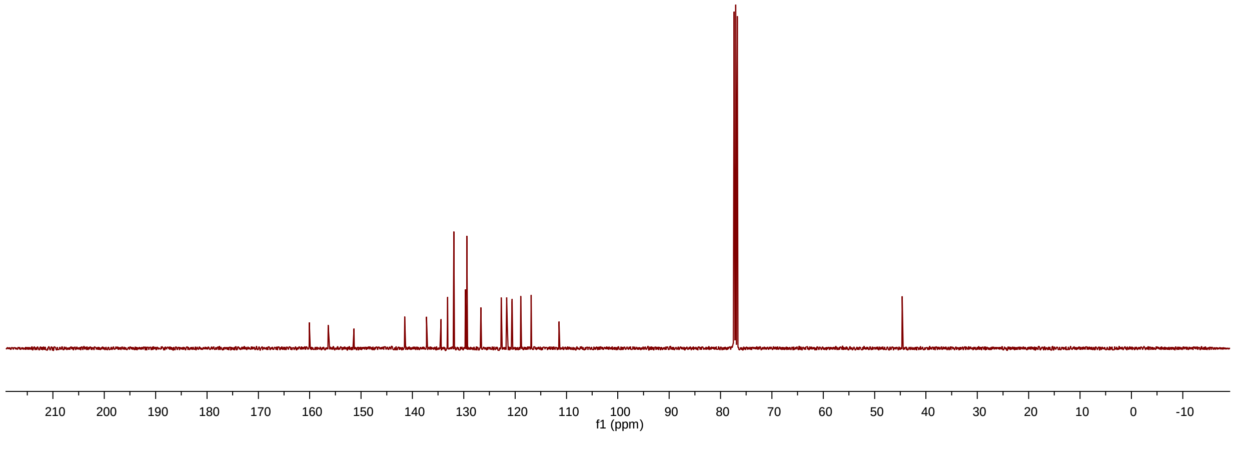

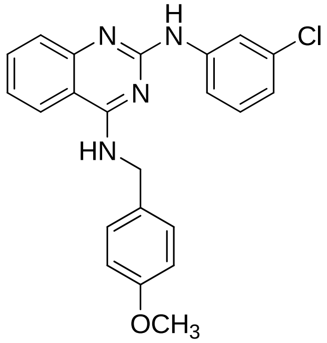

KSC-16-79

***N*[2]-(3-Chlorophenyl)-*N*[4]-(4-methoxybenzyl)quinazoline-2,4-diamine (S97)**. Yield: 11.3 mg, 87%. ^{1}H NMR (400 MHz, $CDCl_3$) δ 8.09 (t, *J* = 2.1 Hz, 1H), 7.65 (d, *J* = 3.5 Hz, 2H), 7.56 (d, *J* = 8.2 Hz, 1H), 7.53 – 7.47 (m, 1H), 7.38 (d, *J* = 8.8 Hz, 2H), 7.23 (dt, *J* = 6.0, 8.2 Hz, 2H), 7.03 (s, br. 1H), 6.99 (dd, *J* = 1.1, 7.9 Hz, 1H), 6.97 – 6.92 (m, 2H), 5.82 (s, br. 1H), 4.81 (d, *J* = 5.2 Hz, 2H), 3.85 (s, 3H). ^{13}C NMR (101 MHz, $CDCl_3$) δ 160.0, 159.3, 156.4, 151.2, 141.6, 134.4, 133.0, 130.1, 129.7, 129.3, 126.5, 122.6, 121.6, 120.7, 118.9, 116.9, 114.3, 111.5, 55.4, 45.0. HRMS (m/z): calcd for $C_{22}H_{20}ClN_4O$ (M+H) 391.1326; found 391.1326.

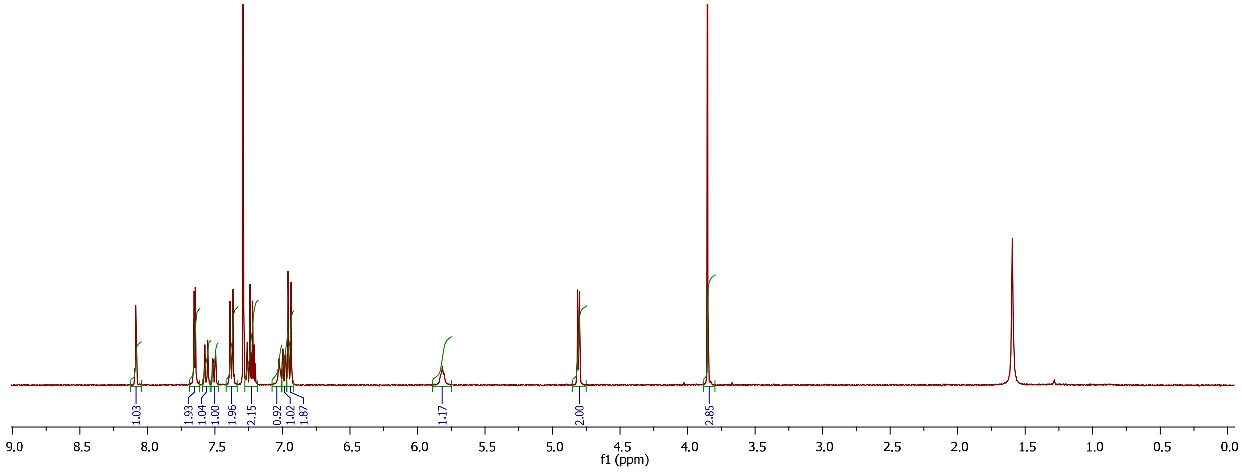

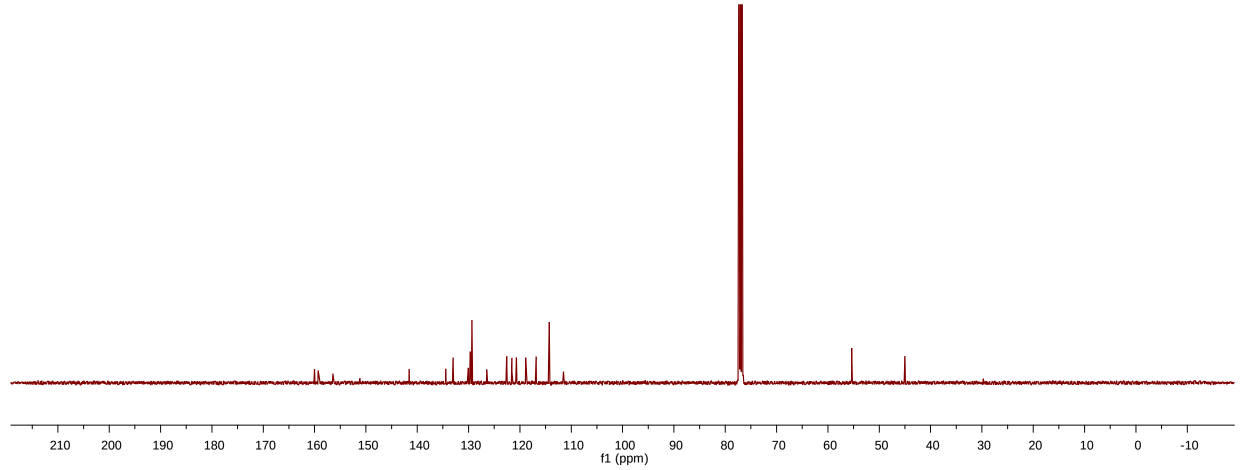

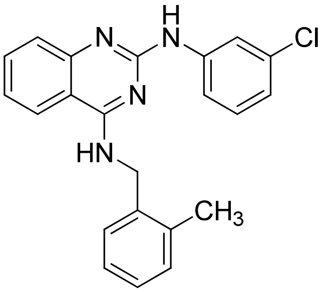

KSC-16-63

***N*^2^-(3-Chlorophenyl)-*N*^4^-(2-methylbenzyl)quinazoline-2,4-diamine (S98)**. Yield: 12.8 mg, 97%. ^{1}H NMR (400 MHz, $CDCl_3$) δ 8.07 (t, *J* = 2.0 Hz, 1H), 7.72 – 7.61 (m, 2H), 7.57 (d, *J* = 8.1 Hz, 1H), 7.52 – 7.42 (m, 1H), 7.38 (d, *J* = 7.3 Hz, 1H), 7.32 – 7.30 (m, 2H), 7.28 – 7.13 (m, 4H), 7.04 – 6.90 (m, 1H), 5.74 (s, br. 1H), 4.85 (d, *J* = 5.0 Hz, 2H), 2.44 (s, 3H). ^{13}C NMR (101 MHz, $CDCl_3$) δ 160.1, 156.5, 151.3, 141.7, 136.8, 135.7, 134.4, 133.0, 130.7, 129.7, 128.6, 128.1, 126.6, 126.4, 122.6, 121.5, 120.7, 118.9, 116.8, 111.5, 43.7, 19.2. HRMS (m/z): calcd for $C_{22}H_{20}ClN_4$ (M+H) 375.1376; found 375.1376.

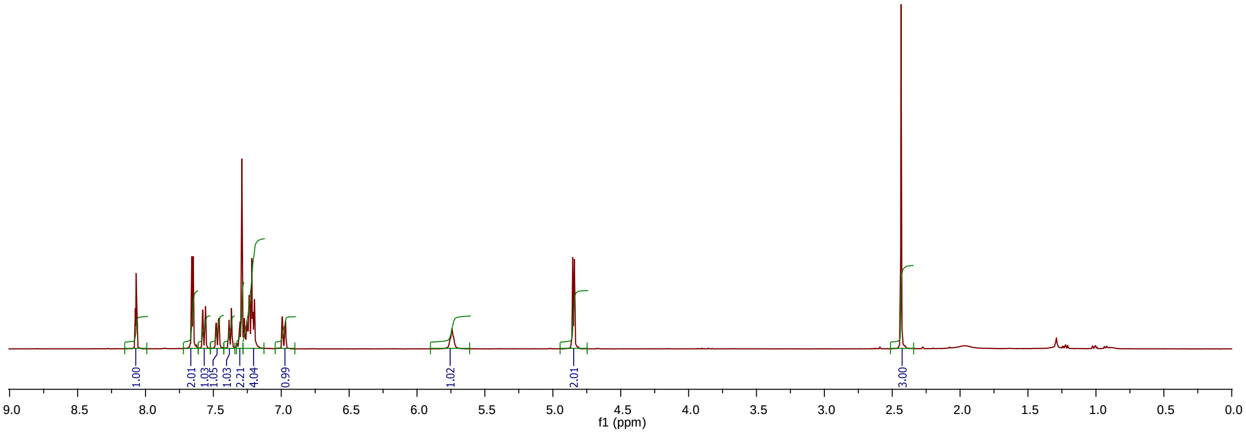

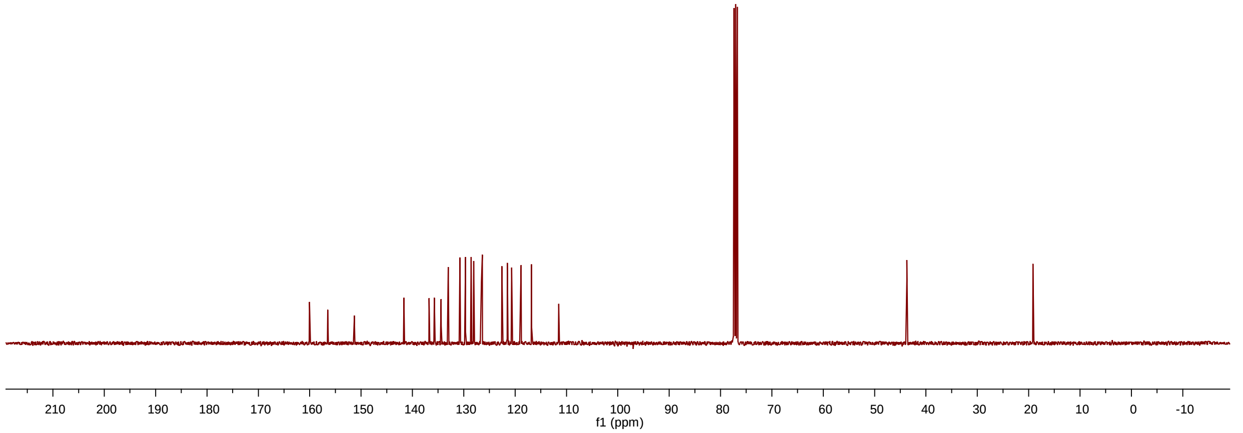

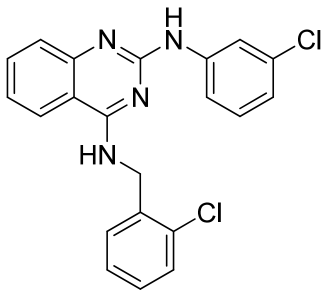

KSC-16-84

***N*[4]-(2-Chlorobenzyl)-*N*[2]-(3-chlorophenyl)quinazoline-2,4-diamine (S99)**. Yield: 13.0 mg, 100%. ^{1}H NMR (400 MHz, CDCl3) δ 8.02 (t, *J* = 2.1 Hz, 1H), 7.71 – 7.57 (m, 3H), 7.53 – 7.40 (m, 3H), 7.29 – 7.18 (m, 4H), 7.14 – 6.93 (m, 2H), 6.12 (s, br. 1H), 4.98 (d, *J* = 5.8 Hz, 2H). ^{13}C NMR (101 MHz, $CDCl_3$) δ 160.1, 156.4, 151.4, 141.6, 135.5, 134.4, 133.8, 133.1, 129.9, 129.72, 129.69, 129.0, 127.1, 126.6, 122.7, 121.6, 120.7, 118.9, 116.9, 111.6, 43.2. HRMS (m/z): calcd for $C_{21}H_{17}Cl_2N_4$ (M+H) 395.0830; found 395.0826.

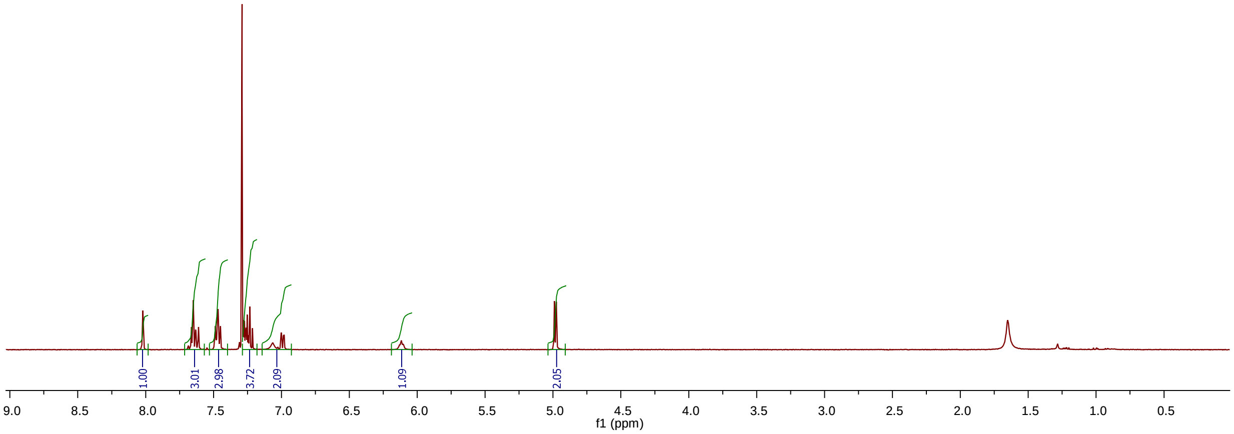

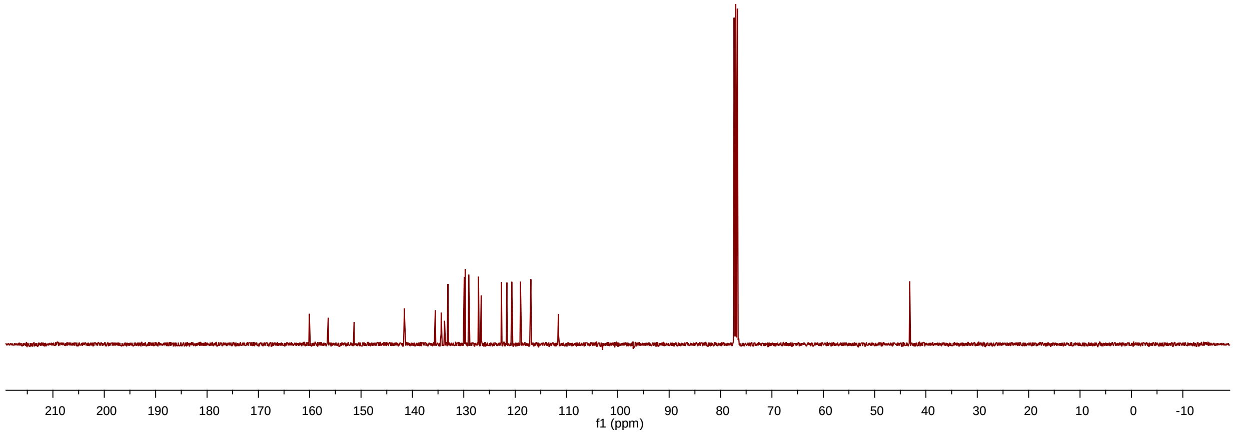

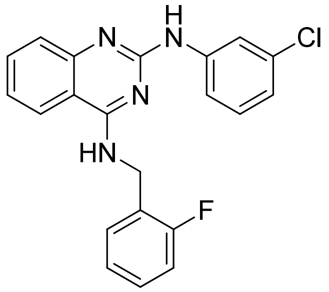

KSC-16-92

***N*[2]-(3-Chlorophenyl)-*N*[4]-(2-fluorobenzyl)quinazoline-2,4-diamine (S100)**. Yield: 12.4 mg, 94%. ^{1}H NMR (400 MHz, $CDCl_3$) δ 8.05 (t, J = 2.0 Hz, 1H), 7.71 – 7.57 (m, 3H), 7.53 – 7.40 (m, 2H), 7.38 – 7.30 (m, 1H), 7.28 – 7.20 (m, 2H), 7.20 – 7.07 (m, 3H), 7.04 – 6.96 (m, 1H), 6.02 (s, br. 1H), 4.95 (d, J = 5.6 Hz, 2H). ^{13}C NMR (101 MHz, $CDCl_3$) δ 162.5, 160.1, 160.0, 156.4, 151.3, 141.6, 134.4, 133.1, 130.2, 130.1, 129.7, 129.5, 129.4, 126.6, 125.2, 125.1, 124.41, 124.38, 122.6, 121.6, 120.7, 119.0, 116.9, 115.7, 115.5, 111.6, 39.4, 39.3. HRMS (m/z): calcd for $C_{21}H_{17}ClFN_4$ (M+H) 379.1126; found 379.1127.

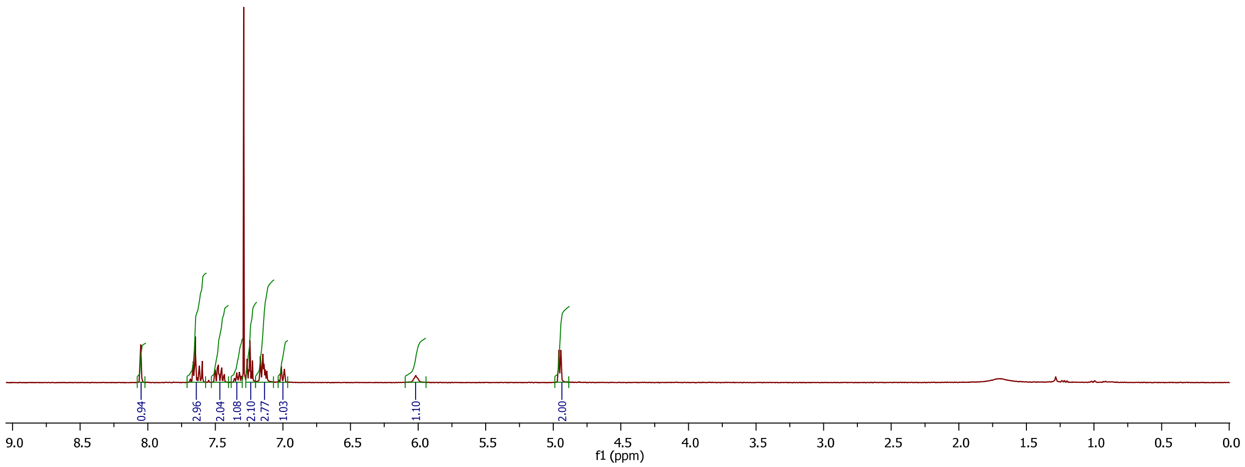

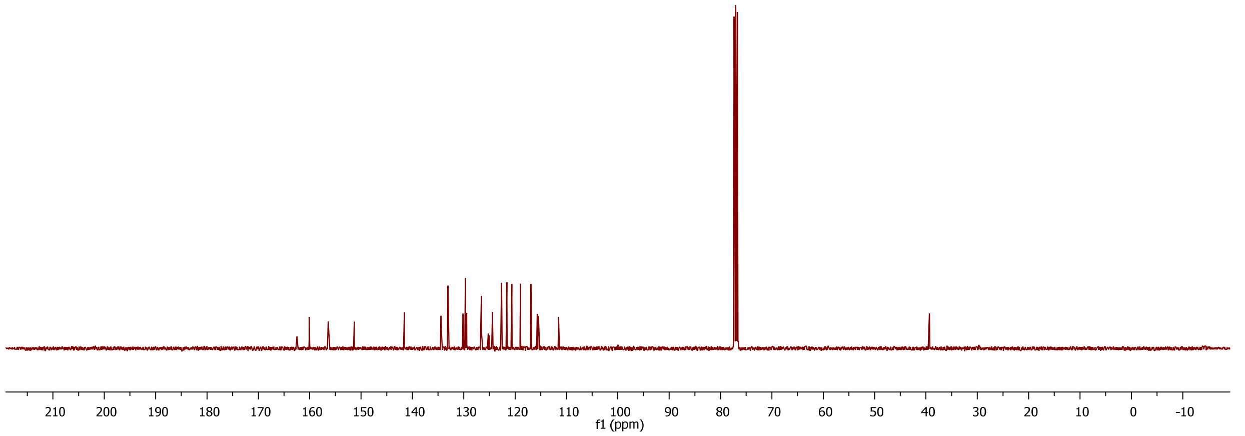

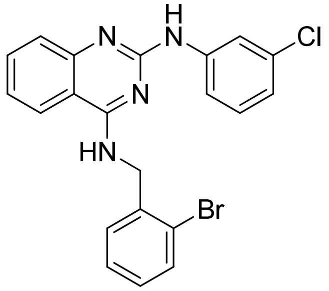

KSC-16-99

***N*[4]-(2-Bromobenzyl)-*N*[2]-(3-chlorophenyl)quinazoline-2,4-diamine (S101)**. Yield: 12.3 mg, 98%. ^{1}H NMR (400 MHz, $CDCl_3$) δ 8.01 (t, *J* = 2.0 Hz, 1H), 7.67 – 7.62 (m, 4H), 7.49 – 7.45 (m, 2H), 7.34 – 7.30 (m, 1H), 7.28 – 7.13 (m, 4H), 7.04 – 6.95 (m, 1H), 6.19 (s, br. 1H), 4.96 (d, *J* = 5.7 Hz, 2H). ^{13}C NMR (101 MHz, $CDCl_3$) δ 160.0, 156.3, 151.2, 141.5, 137.1, 134.4, 133.1, 133.0, 130.1, 129.7, 129.3, 127.8, 126.5, 123.8, 122.7, 121.7, 120.7, 119.0, 117.0, 111.5, 45.5. HRMS (m/z): calcd for $C_{21}H_{17}BrClN_4$ (M+H) 439.0325 and 441.0305; found 441.0298.

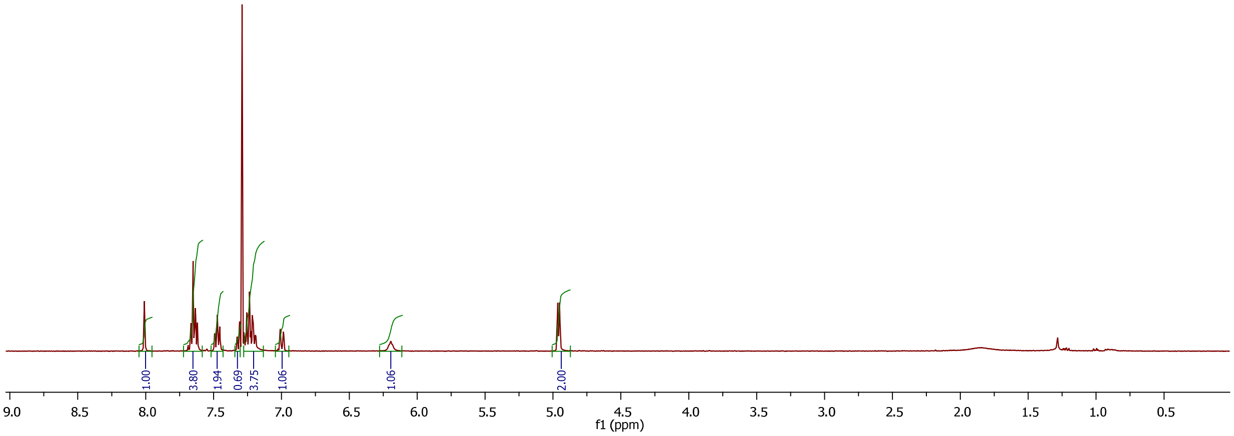

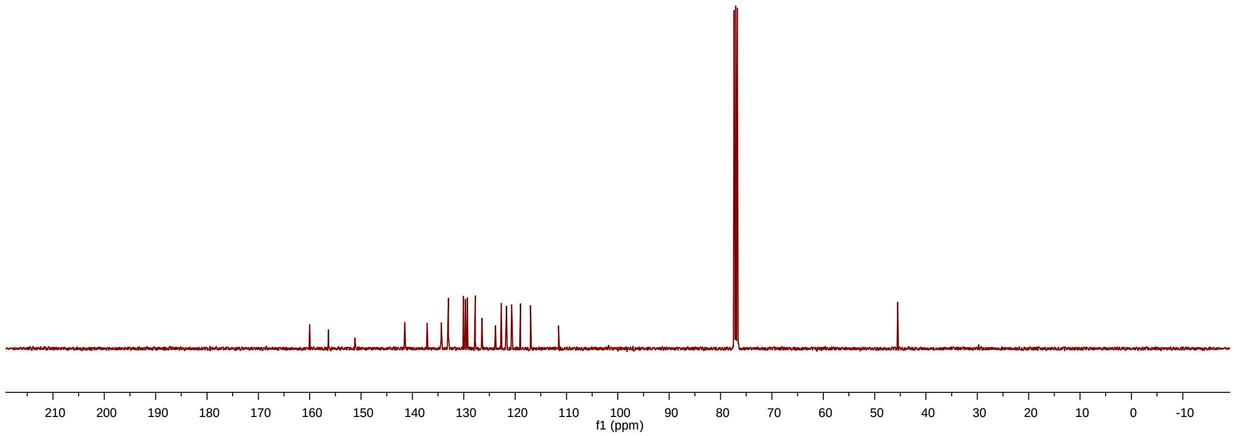

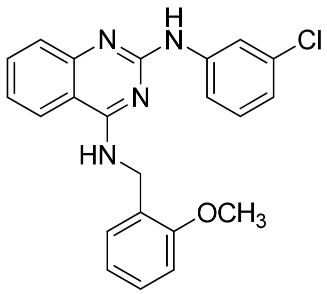

KSC-16-75

***N*[2]-(3-Chlorophenyl)-*N*[4]-(2-methoxybenzyl)quinazoline-2,4-diamine (S102)**. Yield: 13.0 mg, 100%. ^{1}H NMR (400 MHz, $CDCl_3$) δ 8.12 (t, *J* = 2.1 Hz, 1H), 7.68 – 7.59 (m, 2H), 7.56 (d, *J* = 8.2 Hz, 1H), 7.48 (ddd, *J* = 0.9, 2.1, 8.2 Hz, 1H), 7.41 (dd, *J* = 1.7, 7.6 Hz, 1H), 7.38 – 7.30 (m, 1H), 7.27 – 7.18 (m, 2H), 7.16 (s, br. 1H), 7.04 – 6.91 (m, 3H), 6.25 (s, br. 1H), 4.89 (d, *J* = 5.6 Hz, 2H), 3.96 (s, 3H). ^{13}C NMR (101 MHz, $CDCl_3$) δ 160.1, 157.8, 156.6, 151.3, 141.8, 134.4, 132.8, 129.9, 129.6, 129.1, 126.5, 126.1, 122.4, 121.4, 120.8, 120.7, 118.9, 116.9, 111.8, 110.5, 55.5, 41.4. HRMS (m/z): calcd for $C_{22}H_{20}ClN_4O$ (M+H) 391.1326; found 391.1327.

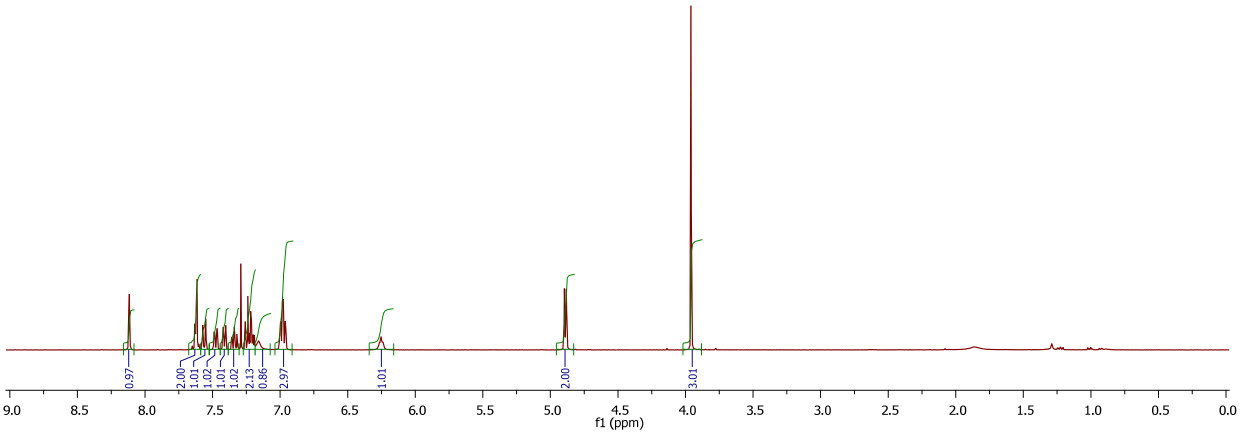

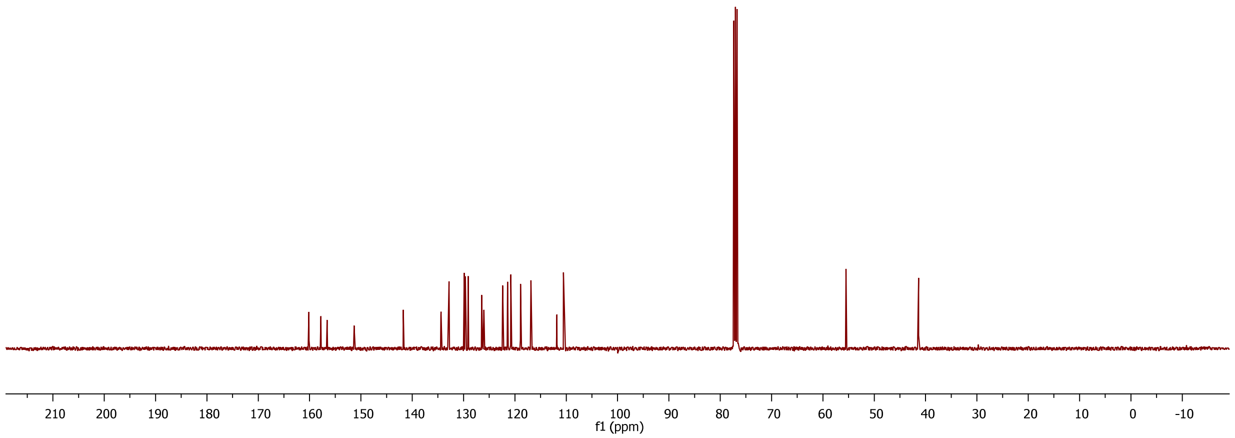

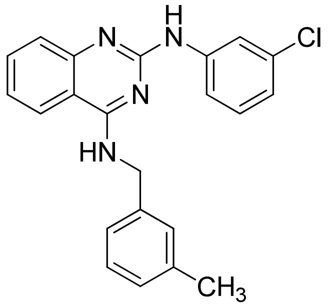

KSC-16-66

***N*²-(3-Chlorophenyl)-*N*⁴-(3-methylbenzyl)quinazoline-2,4-diamine (S103)**. Yield: 13.0 mg, 98%. ^{1}H NMR (400 MHz, $CDCl_3$) δ 8.07 (t, *J* = 2.1 Hz, 1H), 7.65 (d, *J* = 3.5 Hz, 2H), 7.58 (d, *J* = 8.2 Hz, 1H), 7.49 (ddd, *J* = 0.9, 2.1 Hz, 8.2, 1H), 7.34 – 7.29 (m, 1H), 7.28 – 7.08 (m, 6H), 6.98 (ddd, *J* = 0.9, 2.0, 7.9 Hz, 1H), 5.90 (s, br. 1H), 4.84 (d, *J* = 5.2 Hz, 2H), 2.40 (s, 3H). ^{13}C NMR (101 MHz, $CDCl_3$) δ 160.1, 156.5, 151.3, 141.6, 138.7, 138.0, 134.4, 133.0, 129.7, 128.8, 128.7, 128.5, 126.6, 125.0, 122.6, 121.5, 120.7, 118.8, 116.8, 111.6, 45.4, 21.4. HRMS (m/z): calcd for $C_{22}H_{20}ClN_4$ (M+H) 375.1376; found 375.1379.

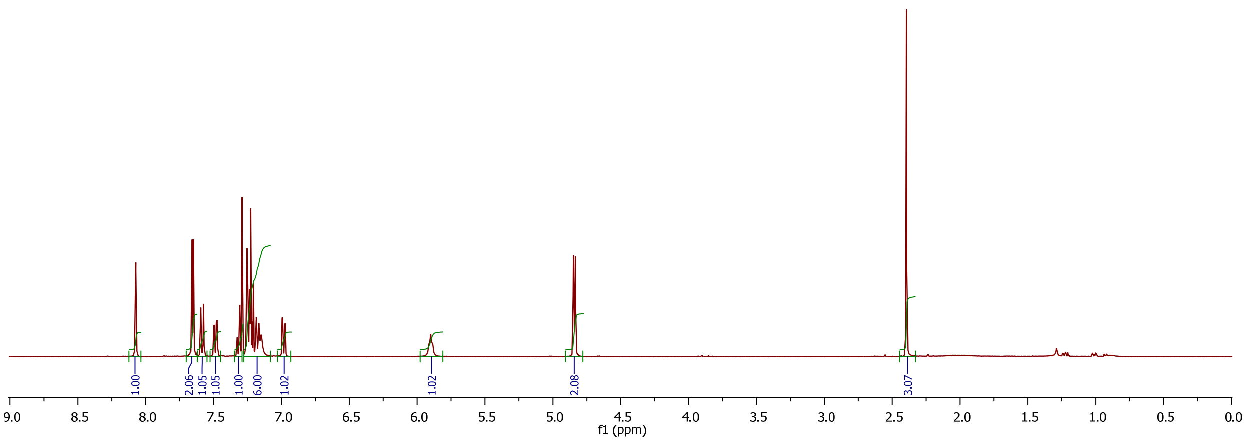

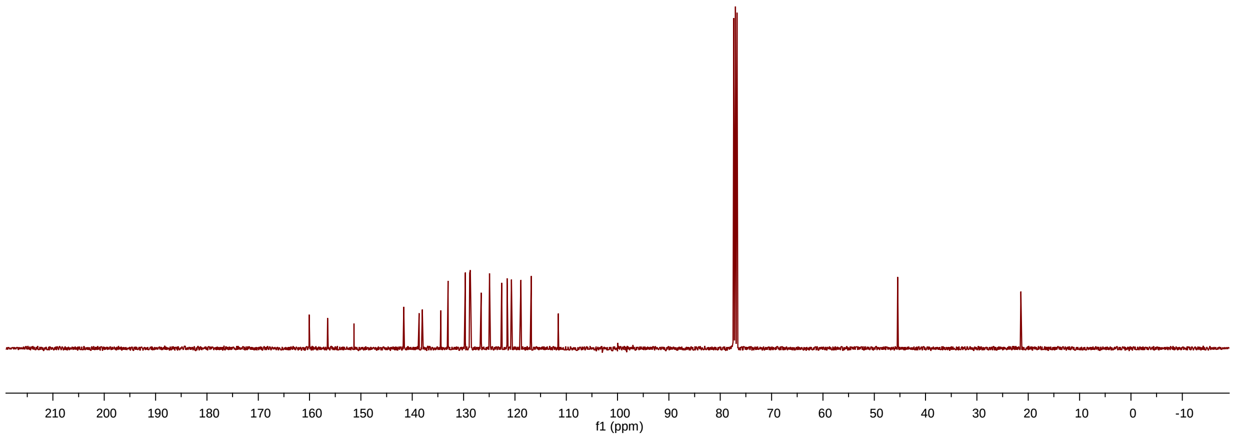

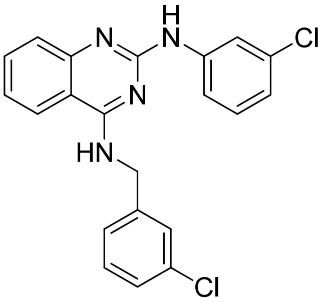

KSC-16-87

N

N^4-(3-Chlorobenzyl)-N^2-(3-chlorophenyl)quinazoline-2,4-diamine (S104). Yield: 13.0 mg 100%. ^{1}H NMR (400 MHz, $CDCl_3$) δ 8.02 (t, *J* = 2.0 Hz, 1H), 7.73 – 7.57 (m, 3H), 7.49 – 7.39 (m, 2H), 7.33 – 7.32 (m, 3H), 7.29 – 7.18 (m, 2H), 7.12 (s, br. 1H), 7.05 – 6.93 (m, 1H), 5.98 (s, br. 1H), 4.88 (d, *J* = 5.5 Hz, 2H). ^{13}C NMR (101 MHz, $CDCl_3$) δ 160.1, 156.3, 151.2, 141.4, 140.3, 134.7, 134.4, 133.2, 130.1, 129.7, 127.8, 127.7, 126.5, 125.8, 122.7, 121.7, 120.7, 119.0, 117.0, 111.4, 44.7. HRMS (m/z): calcd for $C_{21}H_{17}Cl_2N_4$ (M+H) 395.0830; found 395.0826.

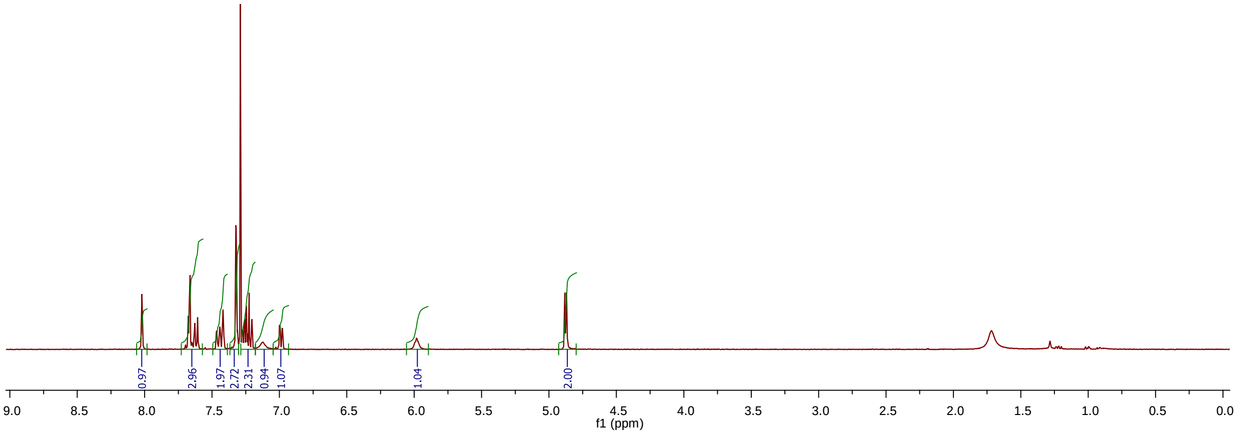

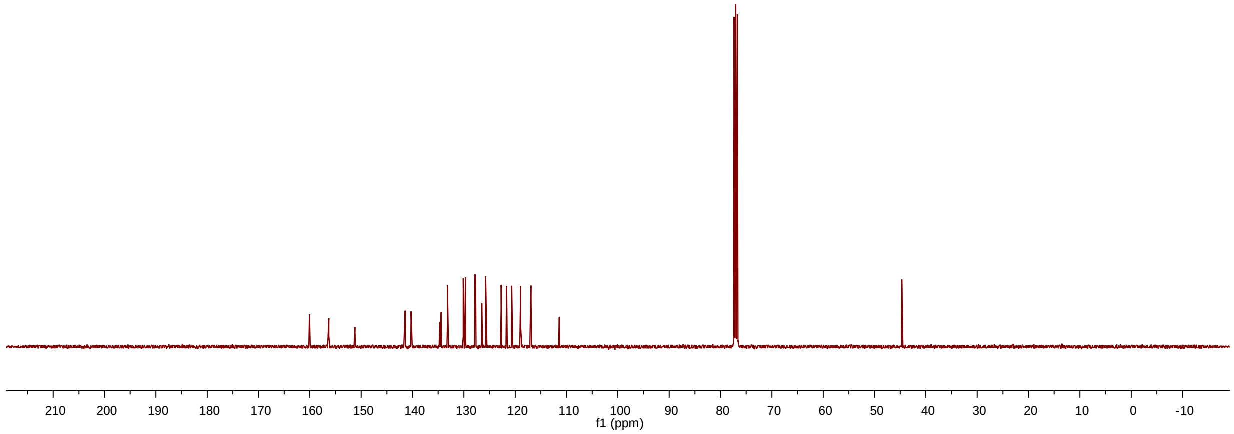

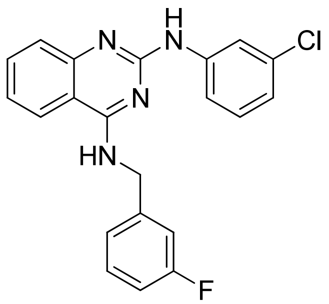

KSC-16-95

***N*[2]-(3-Chlorophenyl)-*N*[4]-(3-fluorobenzyl)quinazoline-2,4-diamine (S105)**. Yield: 13.0 mg, 99%. ^{1}H NMR (400 MHz, $CDCl_3$) δ 8.02 (t, J = 2.0 Hz, 1H), 7.71 – 7.64 (m, 2H), 7.62 (d, J = 8.2 Hz, 1H), 7.46 (d, J = 8.2 Hz, 1H), 7.41 – 7.33 (m, 1H), 7.28 – 7.23 (m, 1H), 7.21 (d, J = 8.0 Hz, 2H), 7.15 – 7.12 (m, 2H), 7.04 (t, J = 8.2 Hz, 1H), 6.99 (dd, J = 1.1, 7.9 Hz, 1H), 6.00 (s, br. 1H), 4.90 (d, J = 5.4 Hz, 2H). ^{13}C NMR (101 MHz, $CDCl_3$) δ 164.3, 161.9, 160.1, 156.4, 151.3, 141.5, 140.9, 140.8, 134.4, 133.2, 130.4, 130.3, 123.0, 126.6, 123.2, 123.1, 122.7, 121.6, 120.6, 118.9, 116.9, 114.66, 114.65, 114.5, 114.4, 111.4, 44.73, 44.71. HRMS (m/z): calcd for $C_{21}H_{17}ClFN_4$ (M+H) 379.1126; found 379.1125.

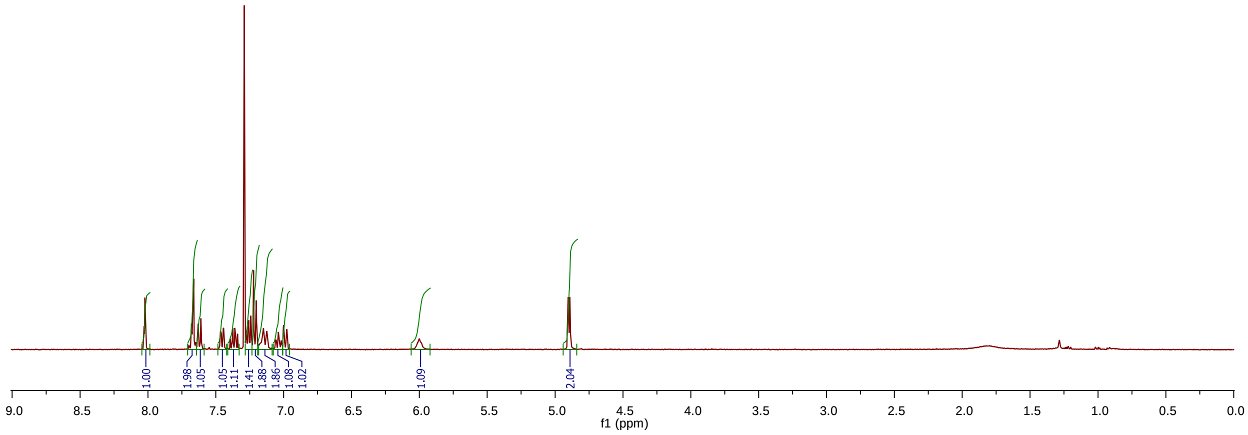

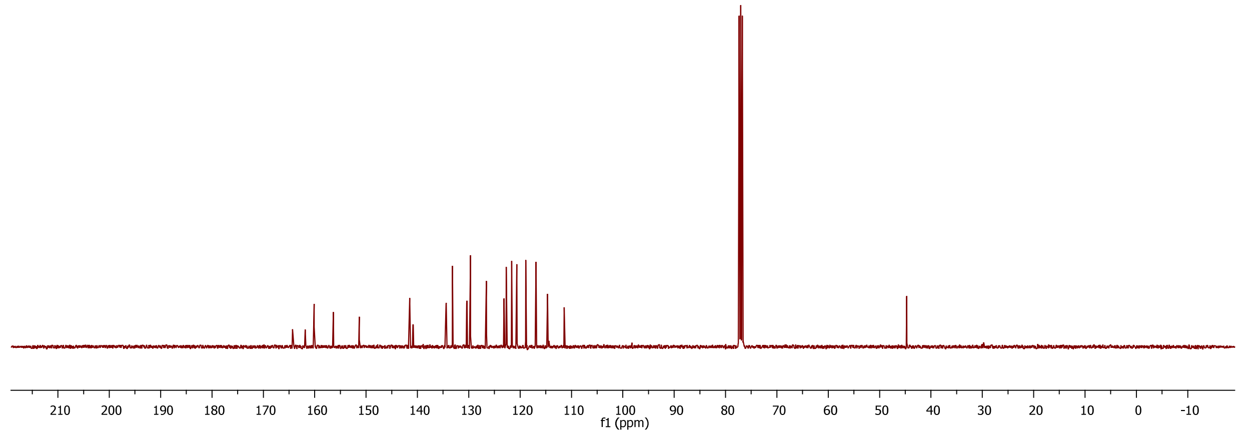

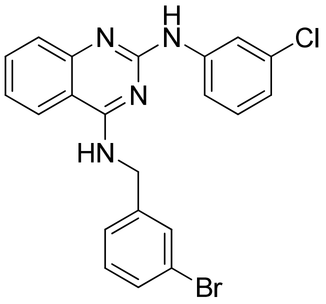

KSC-16-100

***N*^4-(3-Bromobenzyl)-*N*^2-(3-chlorophenyl)quinazoline-2,4-diamine (S106)**. Yield: 12.3 mg, 98%. ^{1}H NMR (400 MHz, $CDCl_3$) δ 8.01 (s, 1H), 7.68 – 7.62 (m, 3H), 7.58 (s, 1H), 7.47 (t, *J* = 9.1 Hz, 2H), 7.36 (d, *J* = 7.7 Hz, 1H), 7.28 – 7.20 (m, 3H), 7.00 (d, *J* = 9.0 Hz, 1H), 6.04 (s, br. 1H), 4.87 (d, *J* = 5.4 Hz, 2H). ^{13}C NMR (101 MHz, $CDCl_3$) δ 160.1, 156.3, 151.2, 141.4, 140.6, 134.4, 133.2, 130.8, 130.7, 130.4, 129.7, 126.5, 126.2, 122.9, 122.7, 121.7, 120.7, 118.9, 116.9, 111.4, 44.6. HRMS (m/z): calcd for $C_{21}H_{17}BrClN_4$ (M+H) 439.0325 and 441.0305; found 441.0299.

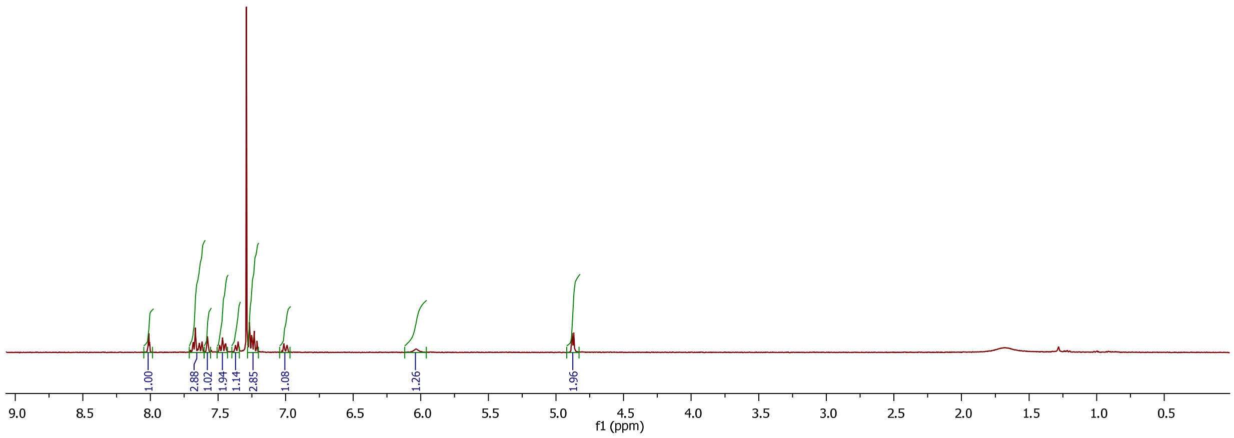

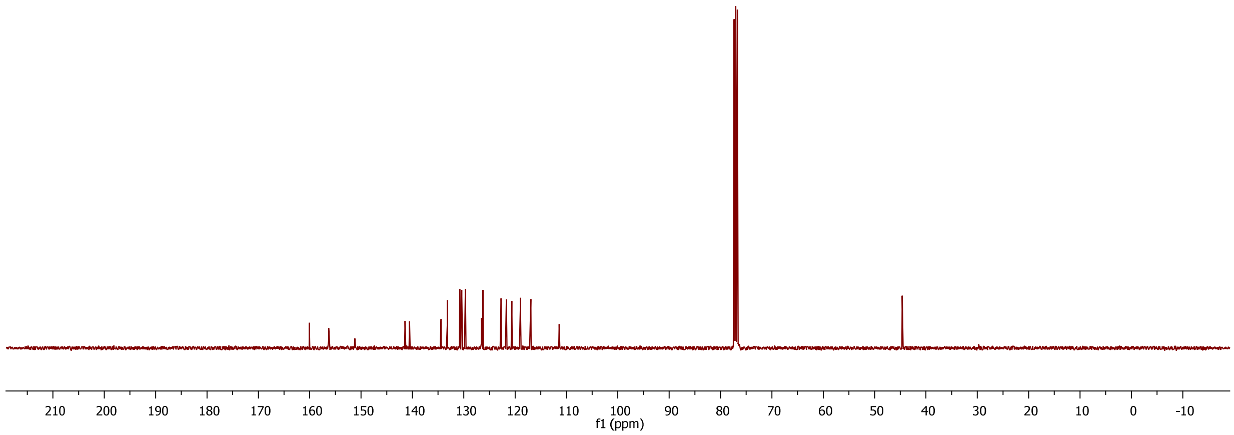

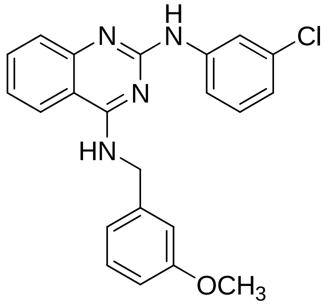

KSC-16-78

N^{2}**-(3-Chlorophenyl)-*N***^{4}**-(3-methoxybenzyl)quinazoline-2,4-diamine (S107)**. Yield: 13.0 mg, 100%. ^{1}H NMR (400 MHz, $CDCl_3$) δ 8.06 (t, *J* = 2.1 Hz, 1H), 7.70 – 7.62 (m, 2H), 7.59 (d, *J* = 8.2 Hz, 1H), 7.48 (ddd, *J* = 0.9, 2.1, 8.2 Hz, 1H), 7.37 – 7.30 (m, 1H), 7.24 – 7.20 (m, 2H), 7.15 (s, br. 1H), 7.03 (d, *J* = 7.5 Hz, 1H), 7.00 – 6.93 (m, 2H), 6.89 (dd, *J* = 1.9, 8.2 Hz, 1H), 5.93 (s, br. 1H), 4.85 (d, *J* = 5.3 Hz, 2H), 3.82 (s, 3H). ^{13}C NMR (101 MHz, $CDCl_3$) δ 160.1, 160.0, 156.5, 151.3, 141.6, 139.8, 134.4, 133.0, 130.0, 129.7, 126.6, 122.6, 121.5, 120.7, 120.0, 118.8, 116.8, 113.6, 113.0, 111.5, 55.3, 45.4. HRMS (m/z): calcd for $C_{22}H_{20}ClN_4O$ (M+H) 391.1326; found 391.1326.

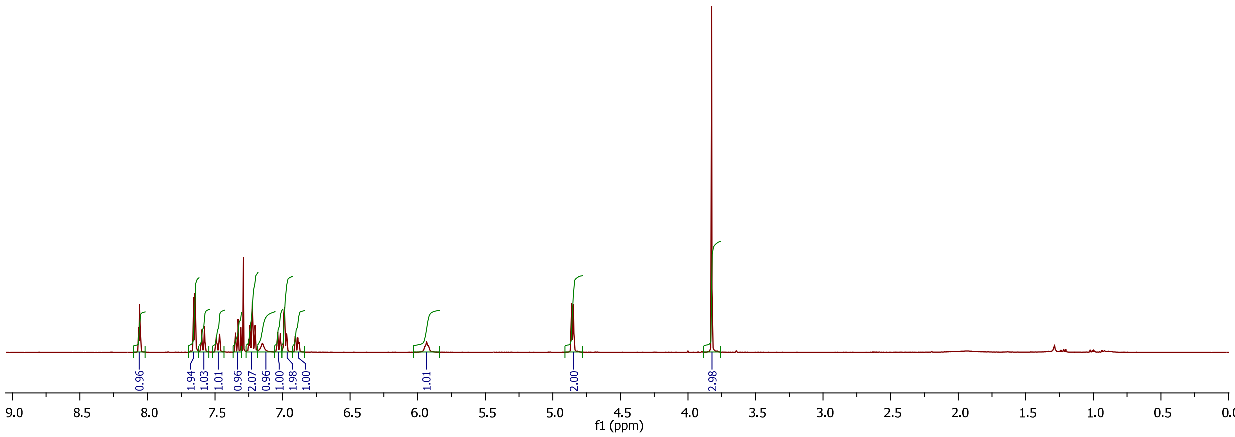

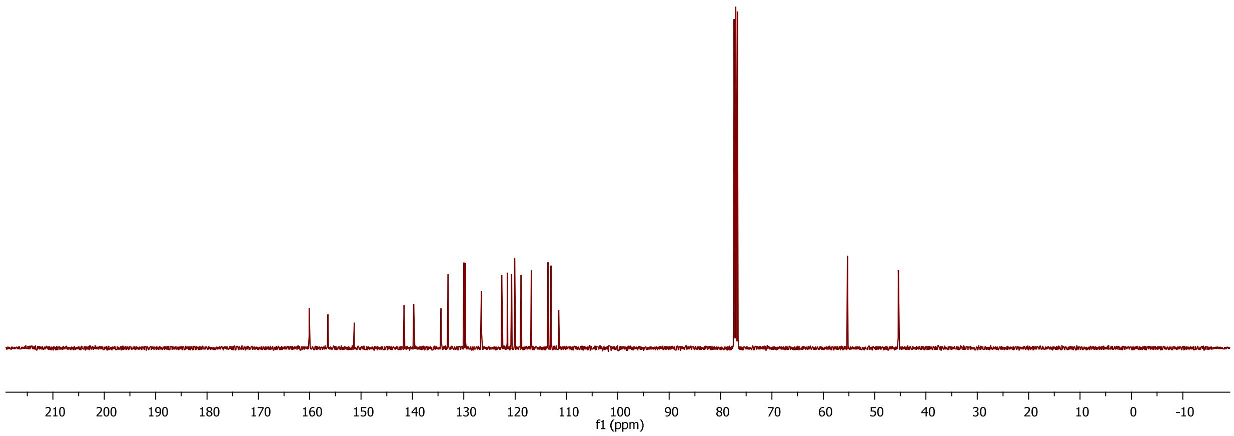

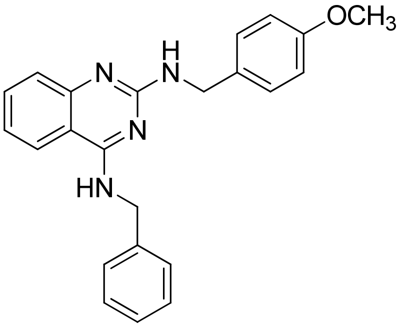

KSC-16-2

N[***4***]**-Benzyl-*****N***[***2***]**-(4-methoxybenzyl)quinazoline-2,4-diamine (S108 or 13)**. Yield: 22.0 mg, 80%. ^{1}H NMR (400 MHz, DMSO) δ 8.44 (s, 1H), 8.03 (d, J = 7.3 Hz, 1H), 7.55 – 7.43 (m, 1H), 7.43 – 7.11 (m, 8H), 7.04 (t, J = 7.1 Hz, 2H), 6.82 (s, br. 2H), 4.74 (d, J = 4.7 Hz, 2H), 4.43 (s, br. 2H), 3.70 (s, 3H). ^{13}C NMR (101 MHz, DMSO) δ 159.9, 159.8, 159.3, 157.8, 139.9, 133.2, 132.2, 129.2, 128.5, 128.2, 127.3, 126.6, 122.7, 119.9, 113.5, 113.4, 54.9, 43.4, 43.3. HRMS (m/z): calcd for $C_{23}H_{23}N_4O$ (M+H) 371.1872; found 371.1868.

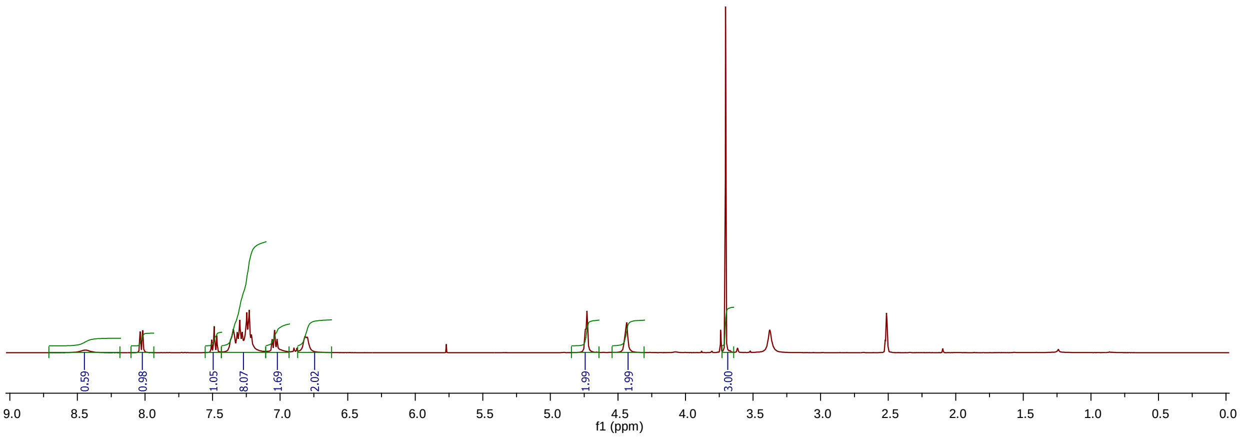

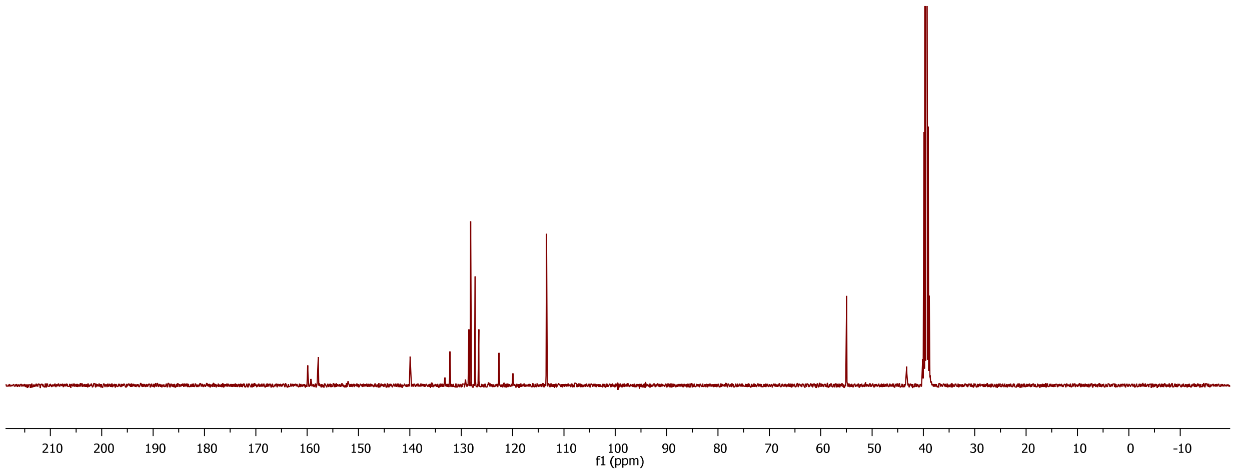

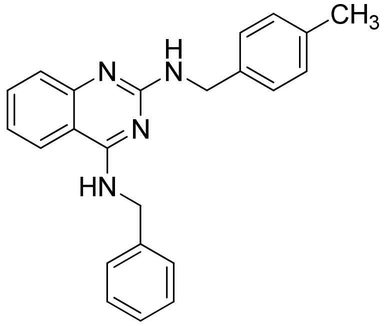

KSC-1-300

***N*[4]-Benzyl-*N*[2]-(4-methylbenzyl)quinazoline-2,4-diamine (S109 or 45)**. Yield: 24.0 mg, 91%. ^{1}H NMR (400 MHz, $CDCl_3$) δ 7.61 – 7.44 (m, 3H), 7.43 – 7.22 (m, 7H), 7.14 (d, *J* = 7.8 Hz, 2H), 7.12 – 7.03 (m, 1H), 5.92 (s, br. 1H), 5.32 (s, br. 1H), 4.80 (d, *J* = 5.4 Hz, 2H), 4.70 (d, *J* = 5.1 Hz, 2H), 2.36 (s, 3H). ^{13}C NMR (101 MHz, $CDCl_3$) δ 160.1, 159.5, 152.2, 138.7, 137.0, 136.6, 132.7, 129.2, 128.7, 128.0, 127.6, 127.5, 125.6, 121.1, 120.8, 111.0, 45.4, 45.1, 21.1. HRMS (m/z): calcd for $C_{23}H_{23}N_4$ (M+H) 355.1923; found 355.1924.

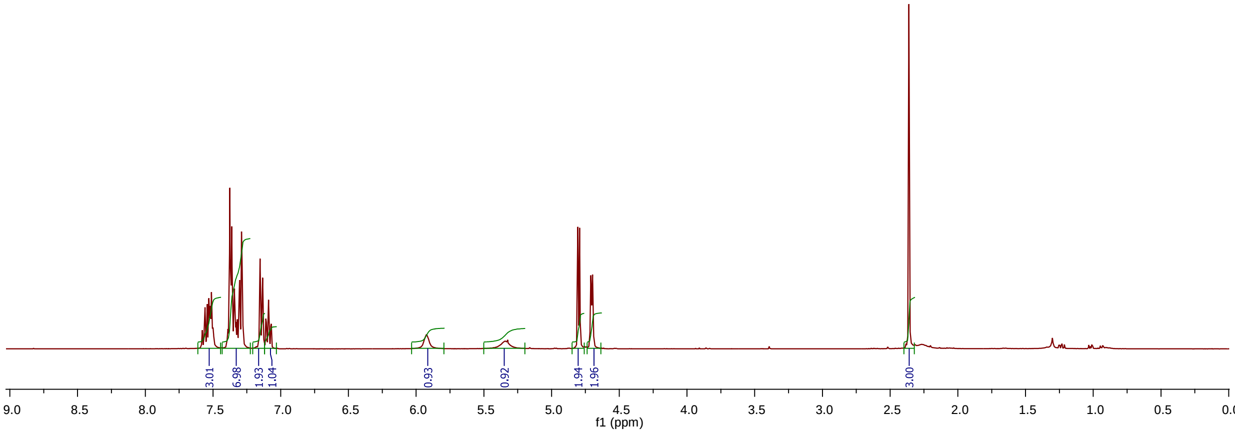

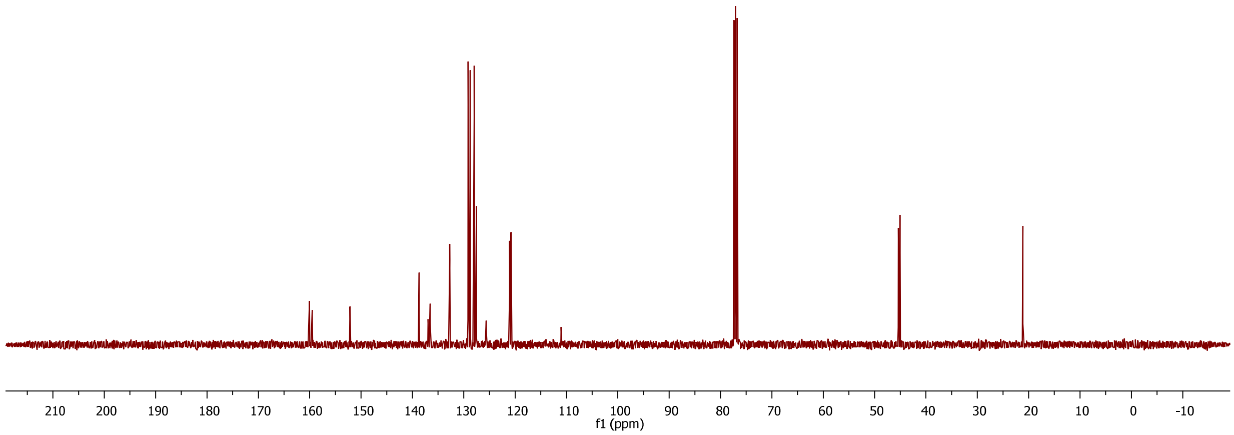

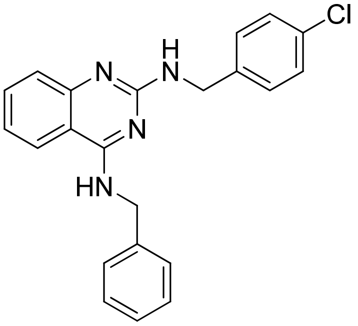

KSC-16-33

***N^4*-Benzyl-*N^2*-(4-chlorobenzyl)quinazoline-2,4-diamine (S110)**. Yield: 27.0 mg, 97%. ^{1}H NMR (400 MHz, $CDCl_3$) δ 7.61 – 7.55 (m, 1H), 7.51 (t, *J* = 8.1, 2H), 7.41 – 7.30 (m, 7H), 7.29 – 7.277 (m, 2H), 7.14 – 7.10 (m, 1H), 5.83 (s, br. 1H), 5.33 (s, br. 1H), 4.79 (d, *J* = 5.5 Hz, 2H), 4.71 (d, *J* = 6.0 Hz, 2H). HRMS (m/z): calcd for $C_{22}H_{20}ClN_4$ (M+H) 375.1376; found 375.1369.

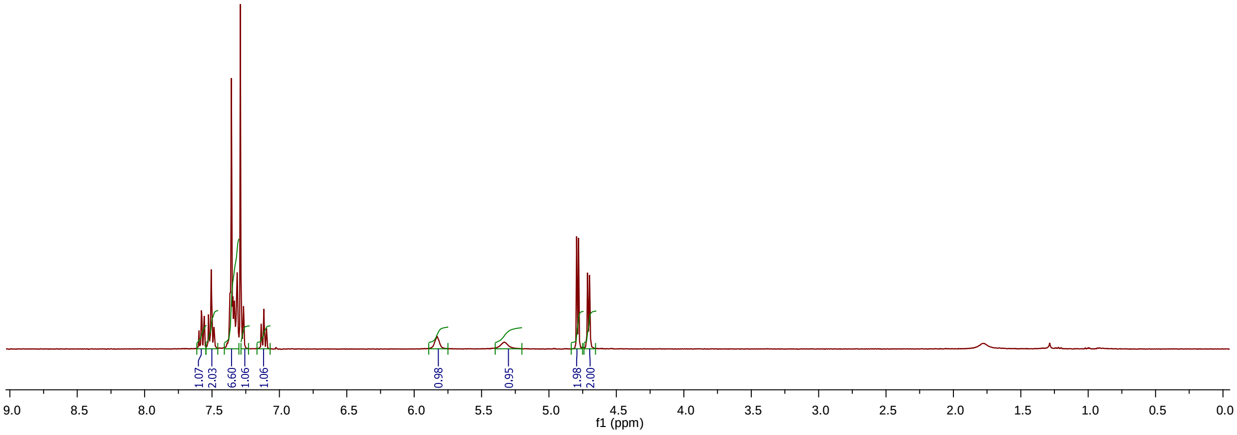

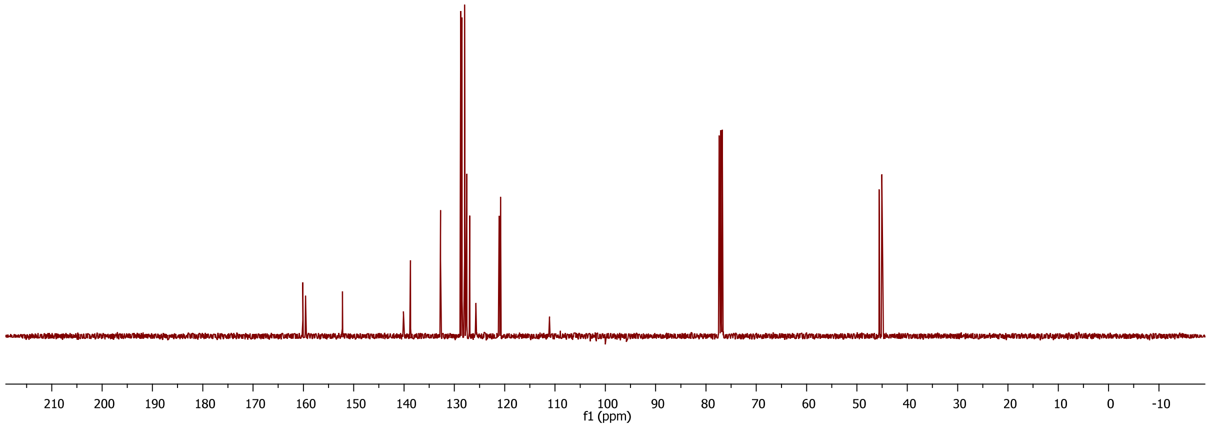

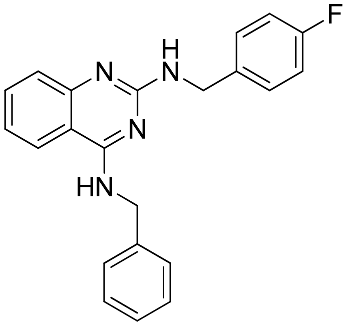

KSC-16-38

***N*[4]-Benzyl-*N*[2]-(4-fluorobenzyl)quinazoline-2,4-diamine (S111)**. Yield: 26.5 mg, 99%. ^{1}H NMR (400 MHz, $CDCl_3$) δ 7.58 (ddd, *J* = 1.4, 6.9, 8.2 Hz, 1H), 7.51 (t, *J* = 8.3 Hz, 2H), 7.43 – 7.30 (m, 7H), 7.11 (ddd, *J* = 1.3, 6.9, 8.2 Hz, 1H), 7.00 (t, *J* = 8.7 Hz, 2H), 5.86 (s, br. 1H), 5.33 (s, br. 1H), 4.80 (d, *J* = 5.5 Hz, 2H), 4.70 (d, *J* = 5.9 Hz, 2H). ^{13}C NMR (101 MHz, $CDCl_3$) δ 163.1, 160.7, 160.1, 159.4, 152.1, 138.6, 135.9, 132.8, 129.2, 129.1, 128.8, 127.9, 127.6, 125.8, 121.2, 120.9, 115.3, 115.1, 111.0, 45.1, 44.8. HRMS (m/z): calcd for $C_{22}H_{20}FN_4$ (M+H) 359.1672; found 359.1672.

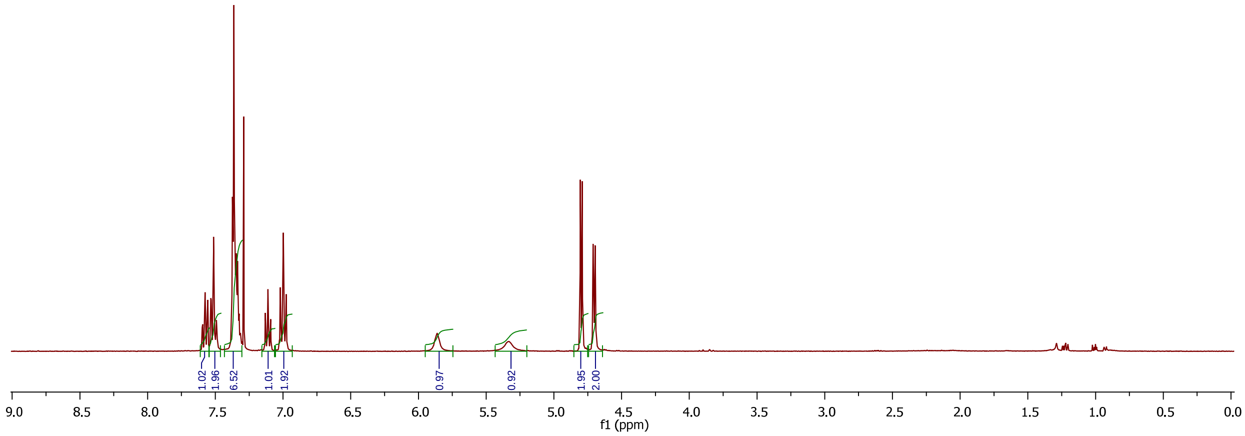

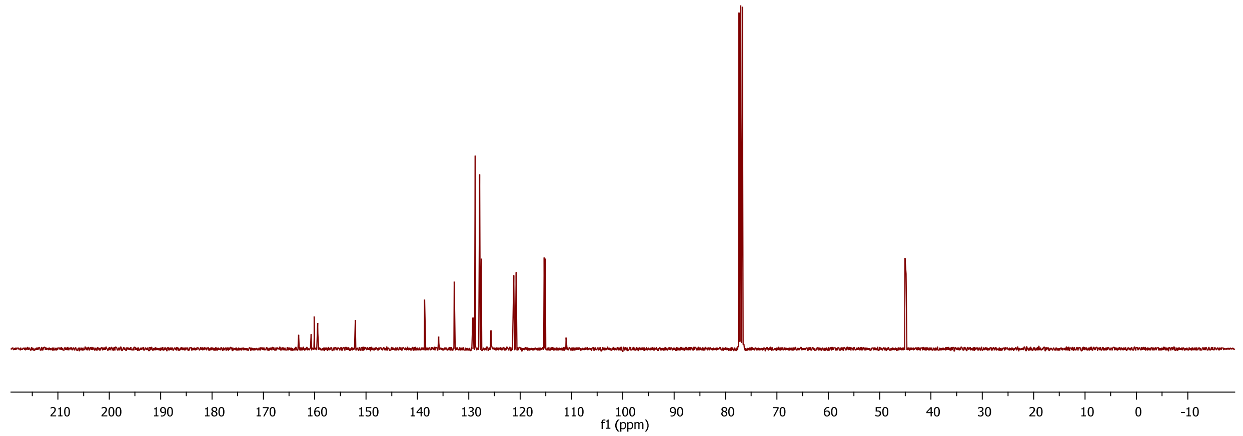

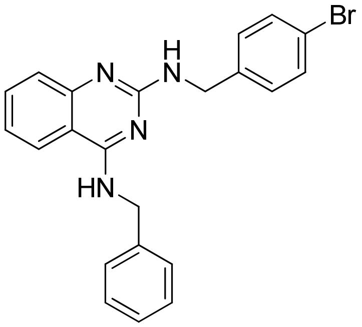

KSC-16-42

***N*[4]-Benzyl-*N*[2]-(4-bromobenzyl)quinazoline-2,4-diamine (S112)**. Yield: 31.0 mg, 100%. ^{1}H NMR (400 MHz, CDCl3) δ 7.62 – 7.54 (m, 1H), 7.50 (t, *J* = 7.6 Hz, 2H), 7.43 (d, *J* = 8.4 Hz, 2H), 7.37 – 7.33 (m, 5H), 7.27 (d, *J* = 8.3 Hz, 2H), 7.12 (t, *J* = 8.1 Hz, 1H), 5.80 (s, br. 1H), 5.28 (s, br. 1H), 4.79 (d, *J* = 5.5 Hz, 2H), 4.69 (d, *J* = 6.1 Hz, 2H). ^{13}C NMR (101 MHz, $CDCl_3$) δ 160.1, 159.4, 152.1, 139.3, 138.5, 132.8, 131.5, 129.2, 128.8, 127.9, 127.6, 125.8, 121.3, 120.7, 120.7, 111.1, 45.1, 44.9. HRMS (m/z): calcd for $C_{22}H_{20}BrN_4$ (M+H) 419.0871 and 421.0851; found 421.0846.

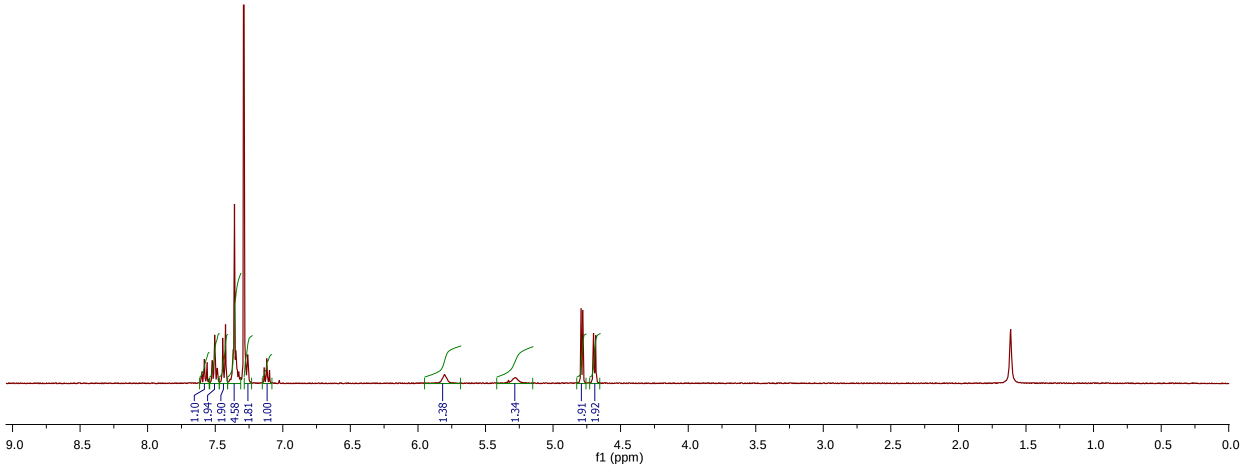

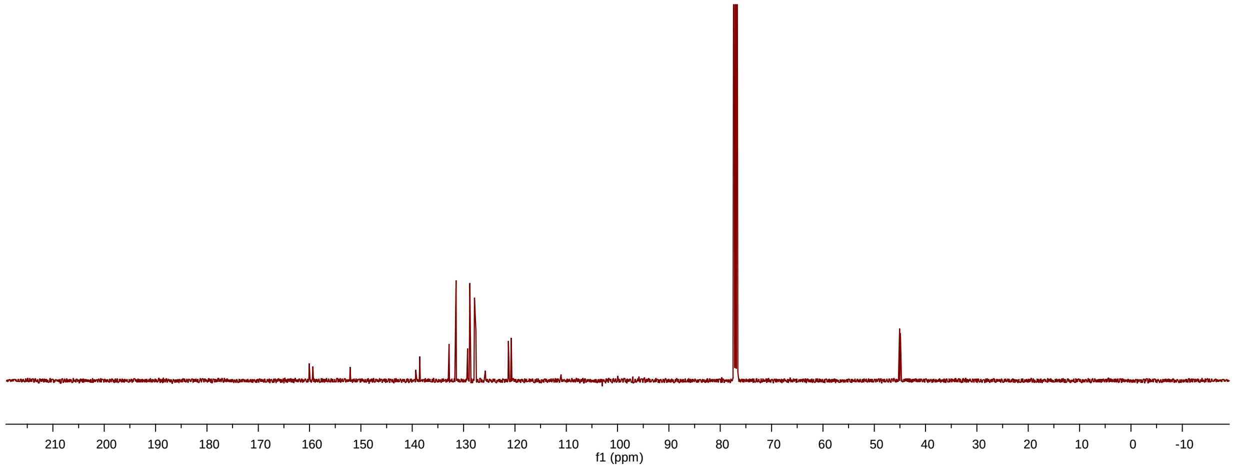

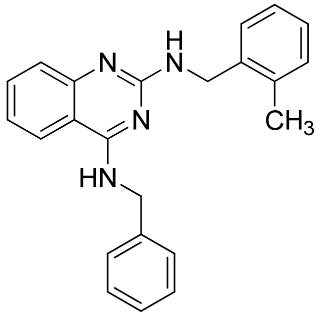

KSC-16-8

***N*[4]-Benzyl-*N*[2]-(2-methylbenzyl)quinazoline-2,4-diamine (S113)**. Yield: 25.0 mg, 95%. ^{1}H NMR (400 MHz, $CDCl_3$) δ 7.59 – 7.52 (m, 3H), 7.43 – 7.30 (m, 6H), 7.25 – 7.13 (m, 3H), 7.13 – 7.04 (m, 1H), 5.93 (s, br. 1H), 5.19 (s, br. 1H), 4.80 (d, J = 5.4 Hz, 2H), 4.72 (d, J = 5.0 Hz, 2H), 2.40 (s, 3H). ^{13}C NMR (101 MHz, $CDCl_3$) δ 160.1, 159.4, 152.2, 138.7, 137.6, 136.4, 132.8, 130.3, 128.8, 128.3, 128.0, 127.6, 127.2, 126.0, 125.7, 121.1, 120.8, 111.1, 45.1, 43.7, 19.1. HRMS (m/z): calcd for $C_{23}H_{23}N_4$ (M+H) 355.1923; found 355.1920.

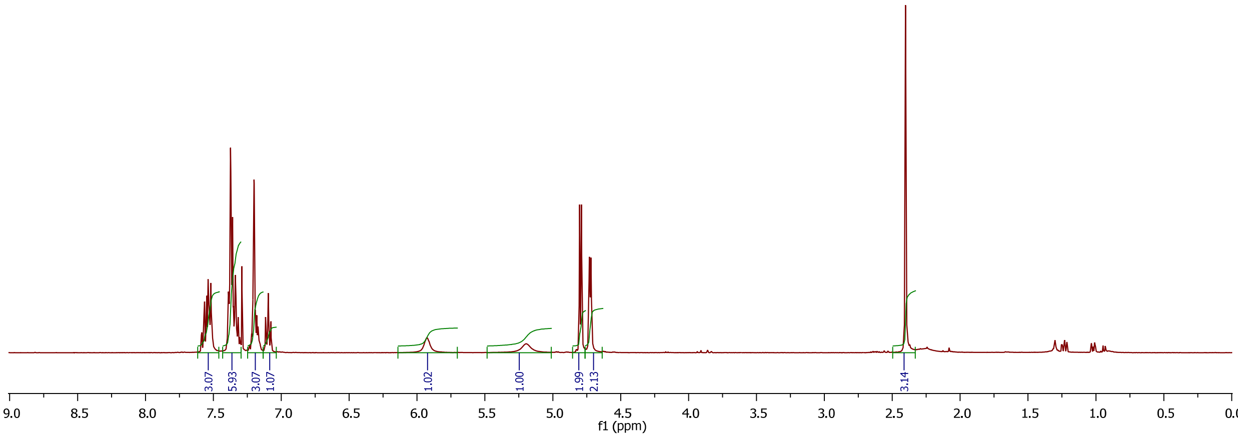

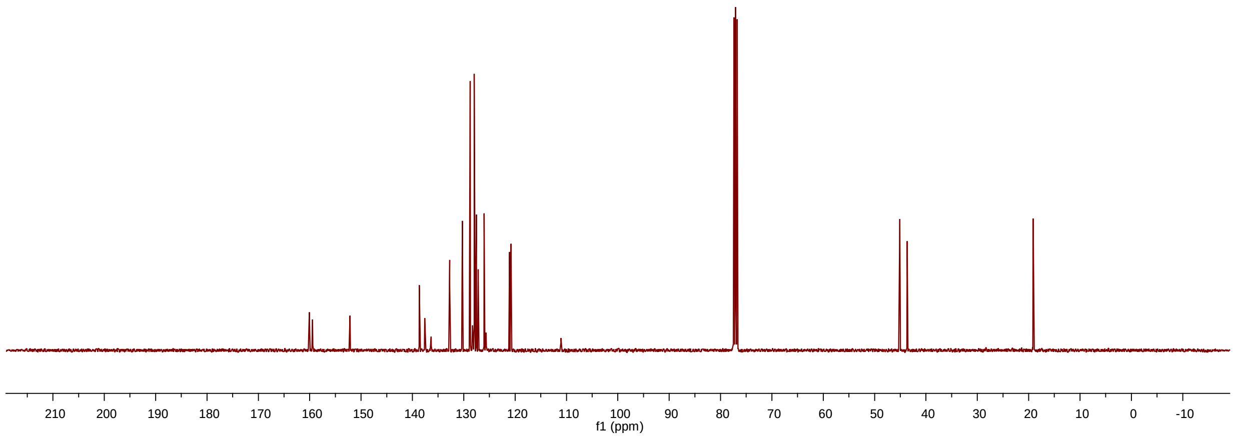

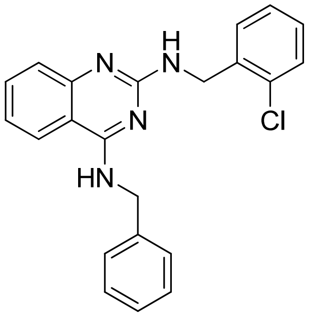

KSC-16-31

***N*[4]-Benzyl-*N*[2]-(2-chlorobenzyl)quinazoline-2,4-diamine (S114)**. Yield: 27.0 mg, 97%. ^{1}H NMR (400 MHz, $CDCl_3$) δ 7.62 – 7.54 (m, 1H), 7.50 (dd, J = 4.1, 7.7 Hz, 3H), 7.42 – 7.30 (m, 6H), 7.24 – 7.14 (m, 2H), 7.10 (ddd, J = 1.2, 6.8, 8.1 Hz, 1H), 5.83 (s, br. 1H), 5.44 (s, br. 1H), 4.84 (d, J = 6.3 Hz, 2H), 4.80 (d, J = 5.5 Hz, 2H). ^{13}C NMR (101 MHz, $CDCl_3$) δ 160.1, 159.4, 152.2, 138.6, 137.6, 133.4, 132.8, 129.6, 129.3, 128.8, 128.2, 127.9, 127.5, 126.8, 125.8, 121.2, 120.7, 111.0, 45.1, 43.2. HRMS (m/z): calcd for $C_{22}H_{20}ClN_4$ (M+H) 375.1376; found 375.1376.

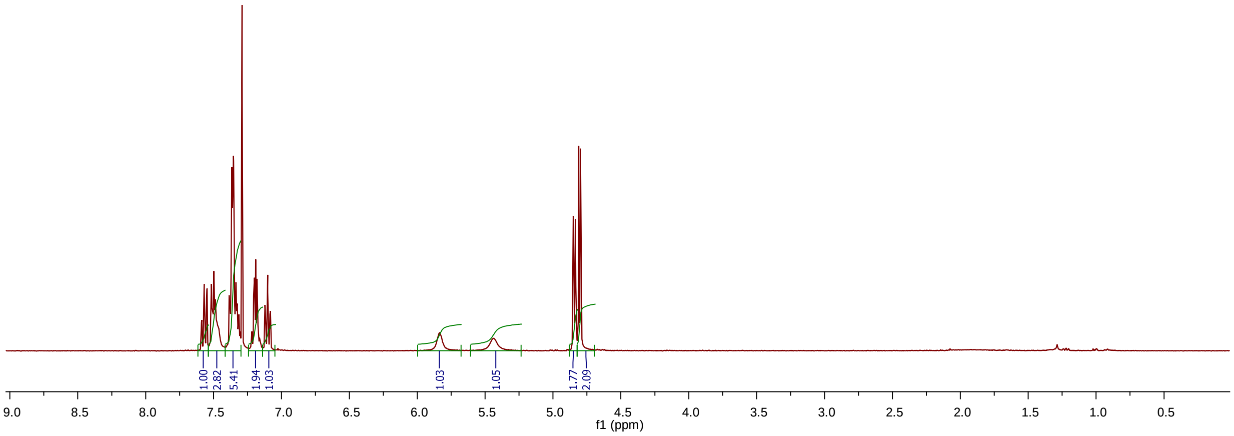

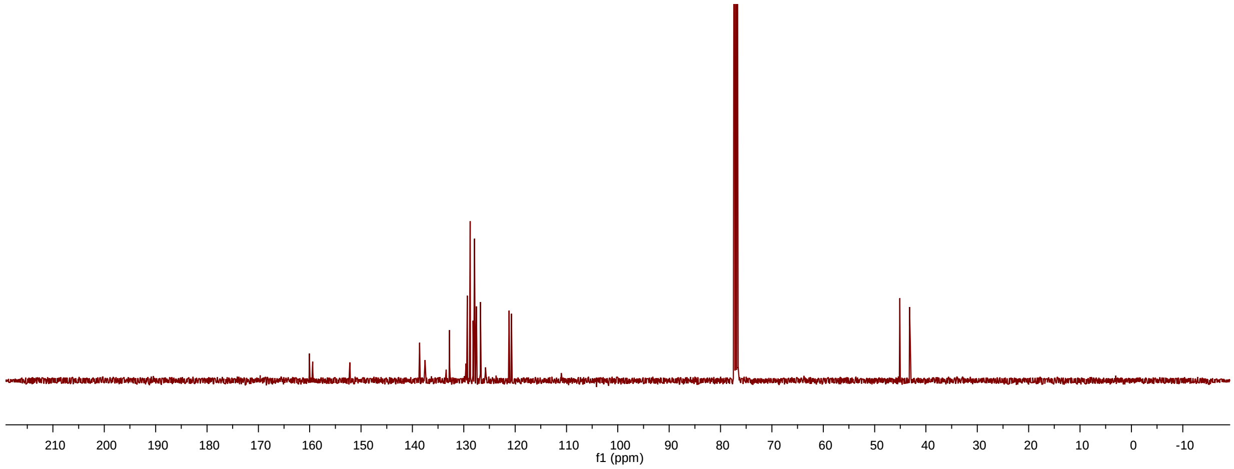

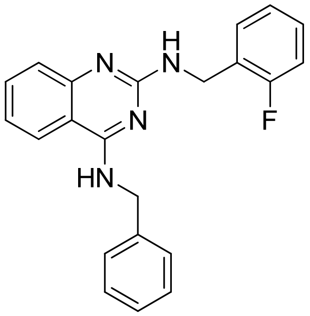

KSC-16-35

***N*[4]-Benzyl-*N*[2]-(2-fluorobenzyl)quinazoline-2,4-diamine (S115)**. Yield: 26.5 mg, 99%. ^{1}H NMR (400 MHz, $CDCl_3$) δ 7.51 – 7.43 (m, 1H), 7.40 (d, *J* = 7.9 Hz, 2H), 7.33 (s, 1H), 7.30 – 7.20 (m, 5H), 7.17 – 7.07 (m, 1H), 7.05 – 6.88 (m, 3H), 5.73 (s, br. 1H), 5.27 (s, br. 1H), 4.72 – 4.69 (m, 4H). ^{13}C NMR (101 MHz, CDCl3) δ 162.3, 160.1, 159.8, 159.4, 152.2, 138.6, 132.8, 129.9, 128.8, 128.6, 128.5, 127.9, 127.6, 127.2, 127.0, 125.7, 124.0, 124.0, 121.2, 120.7, 115.3, 115.0, 111.0, 45.1, 39.24, 39.20. HRMS (m/z): calcd for $C_{22}H_{20}FN_4$ (M+H) 359.1672; found 359.1672.

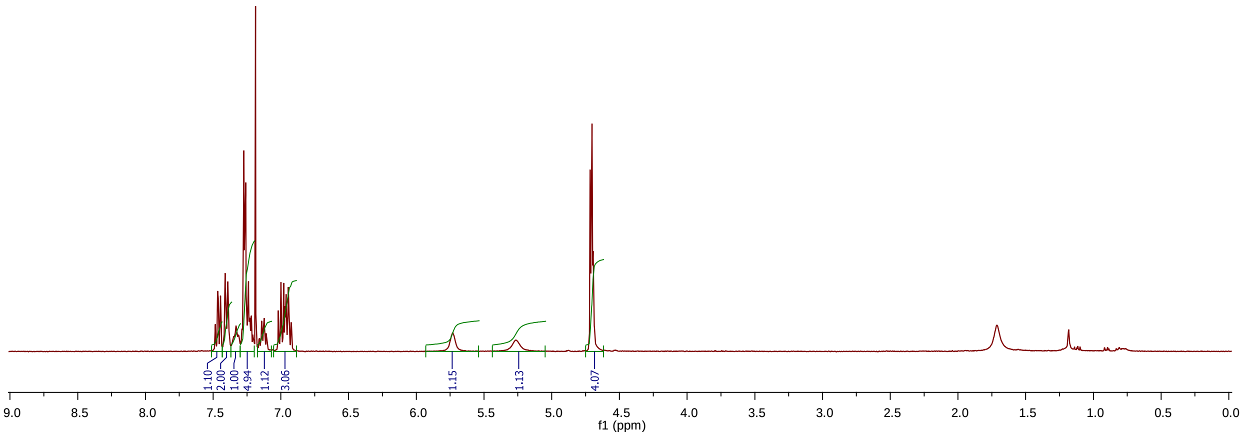

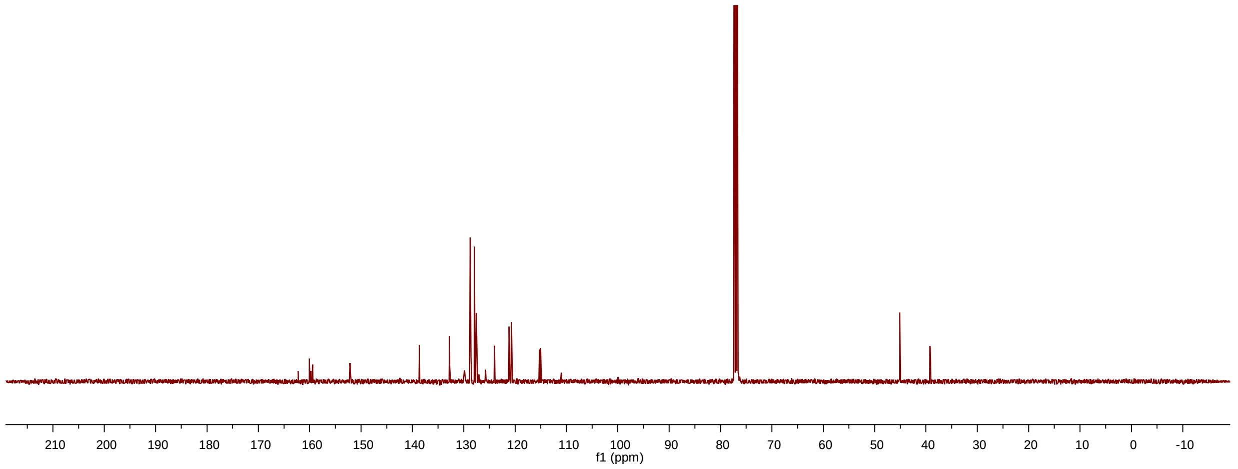

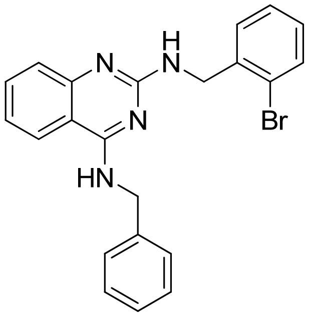

KSC-16-40

***N*[4]-Benzyl-*N*[2]-(2-bromobenzyl)quinazoline-2,4-diamine (S116)**. Yield: 31.0 mg, 99%. ^{1}H NMR (400 MHz, $CDCl_3$) δ 7.58 (dd, *J* = 4.9, 11.7 Hz, 2H), 7.51 – 7.46 (m, 3H), 7.36 – 7.31 (m, 5H), 7.23 (t, *J* = 7.3 Hz, 1H), 7.16 – 7.05 (m, 2H), 5.82 (s, br. 1H), 5.47 (s, br. 1H), 4.82 (d, *J* = 6.4 Hz, 2H), 4.81 (d, *J* = 6.4 Hz, 2H). ^{13}C NMR (101 MHz, $CDCl_3$) δ 160.1, 159.4, 152.2, 139.2, 138.6, 132.8, 132.6, 129.7, 128.8, 128.5, 127.9, 127.5, 127.4, 125.7, 123.5, 121.2, 120.7, 111.0, 45.6, 45.1. HRMS (m/z): calcd for $C_{22}H_{20}BrN_4$ (M+H) 419.0871 and 421.0851; found 419.0866.

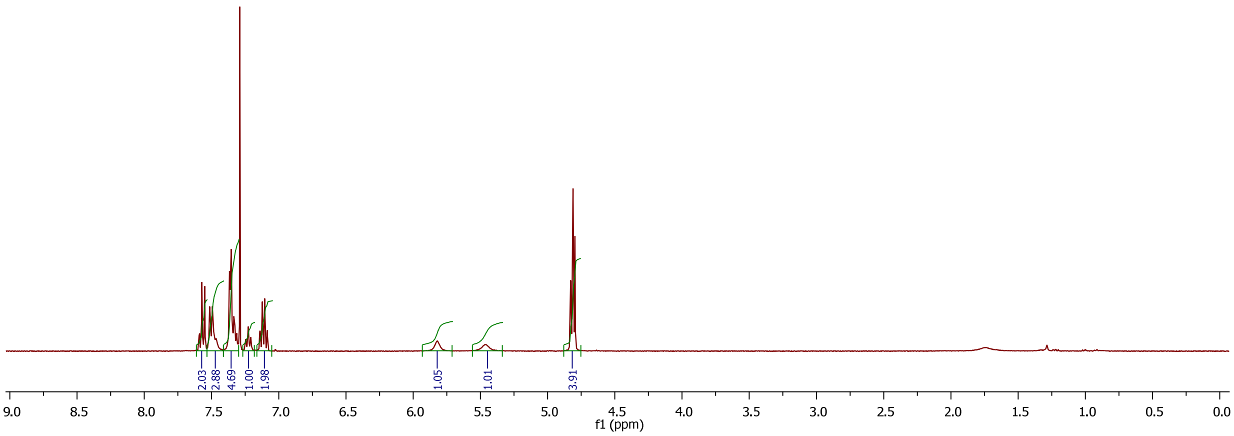

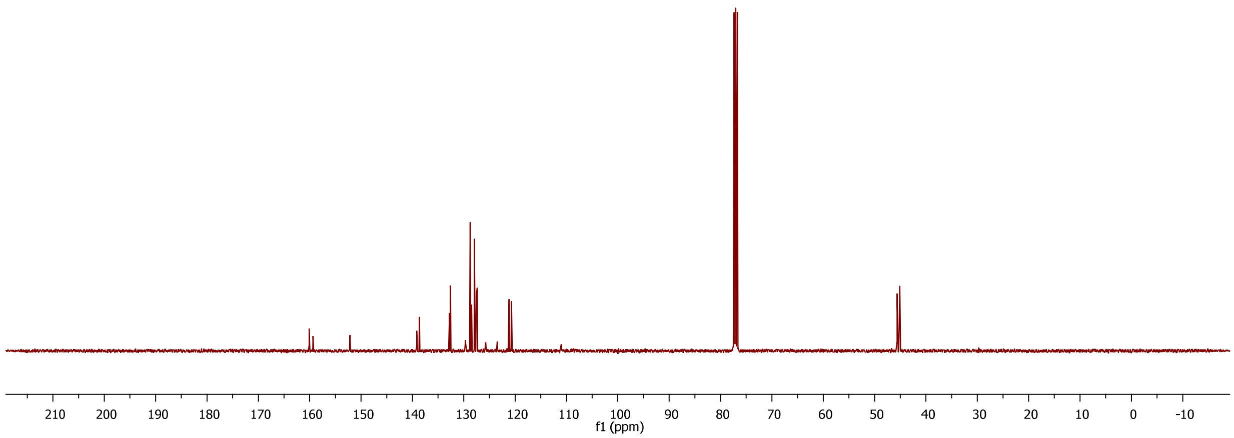

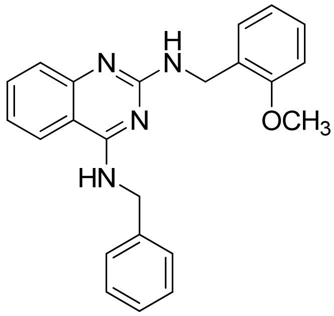

KSC-16-29

***N*[4]-Benzyl-*N*[2]-(2-methoxybenzyl)quinazoline-2,4-diamine (S117)**. Yield: 26.4 mg, 96%. ^{1}H NMR (400 MHz, $CDCl_3$) δ 7.61 – 7.43 (m, 3H), 7.43 – 7.30 (m, 6H), 7.28 – 7.20 (m, 1H), 7.11 – 7.01 (m, 1H), 6.95 – 6.81 (m, 2H), 5.93 (s, br. 1H), 5.48 (s, br. 1H), 4.84 (d, J = 5.4 Hz, 2H), 4.75 (d, J = 6.0 Hz, 2H), 3.86 (s, 3H). ^{13}C NMR (101 MHz, CDCl3) δ 160.1, 159.7, 157.6, 152.3, 138.8, 132.6, 129.4, 128.7, 128.2, 128.1, 128.0, 127.5, 125.6, 120.8, 120.8, 120.4, 110.9, 110.1, 55.3, 45.1, 41.1. HRMS (m/z): calcd for $C_{23}H_{23}N_4O$ (M+H) 371.1872; found 371.1869.

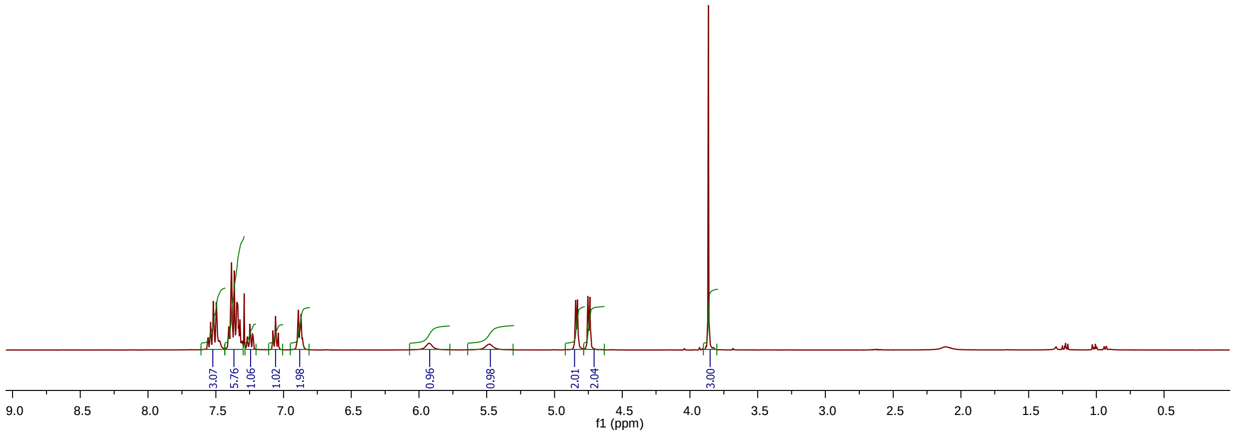

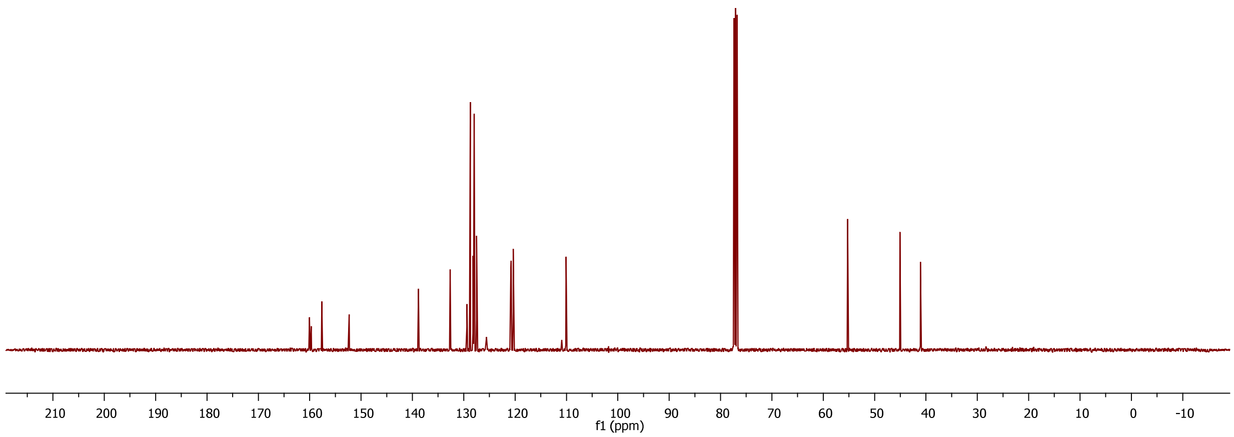

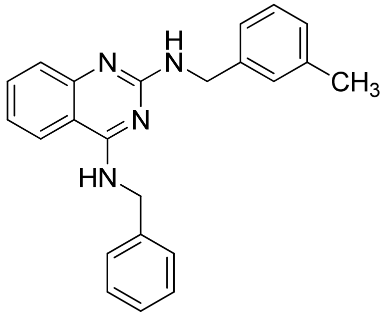

KSC-16-28

***N*[4]-Benzyl-*N*[2]-(3-methylbenzyl)quinazoline-2,4-diamine (S118)**. Yield: 26.0 mg, 99%. ^{1}H NMR (400 MHz, CDCl3) δ 7.62 – 7.46 (m, 3H), 7.43 – 7.30 (m, 5H), 7.27 – 7.15 (m, 3H), 7.09 (ddd, J = 1.4, 6.7, 8.1 Hz, 2H), 5.92 (s, br. 1H), 5.35 (s, br. 1H), 4.80 (d, J = 5.5 Hz, 2H), 4.72 (d, J = 5.1 Hz, 2H), 2.35 (s, 3H). ^{13}C NMR (101 MHz, $CDCl_3$) δ 160.1, 159.5, 152.2, 140.0, 138.7, 138.1, 132.8, 128.7, 128.4, 128.0, 127.7, 127.6, 125.7, 124.7, 121.1, 120.8, 111.1, 45.6, 45.1, 21.4. HRMS (m/z): calcd for $C_{23}H_{23}N_4$ (M+H) 355.1923; found 355.1922.

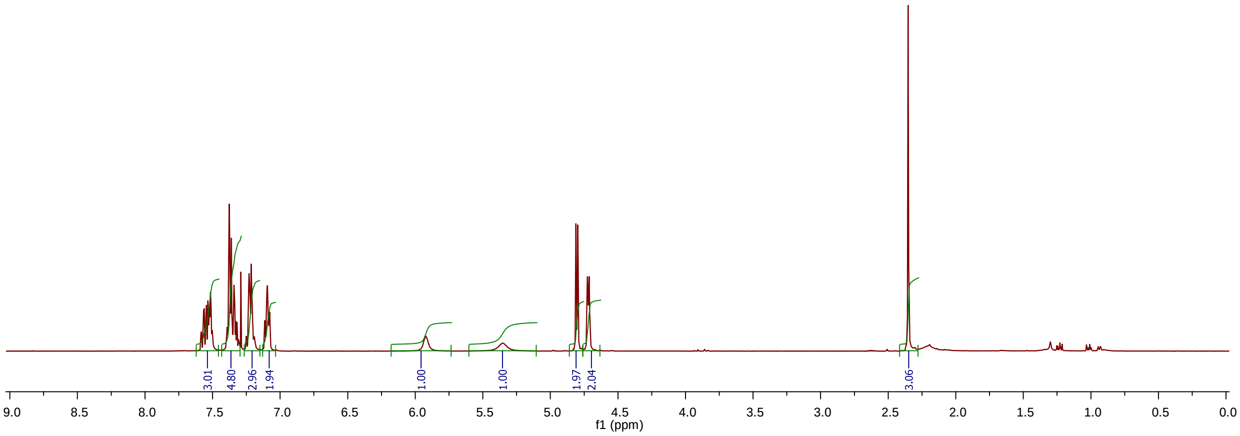

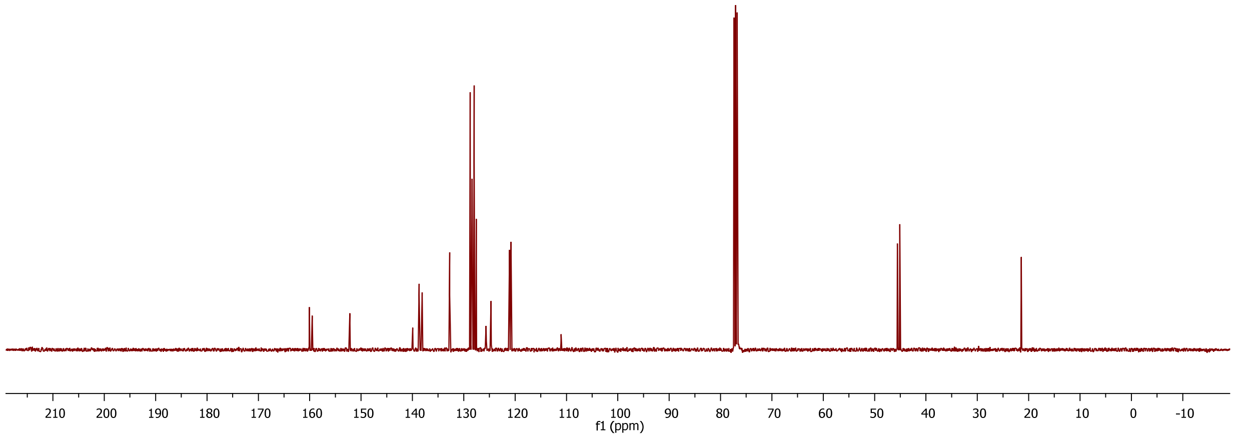

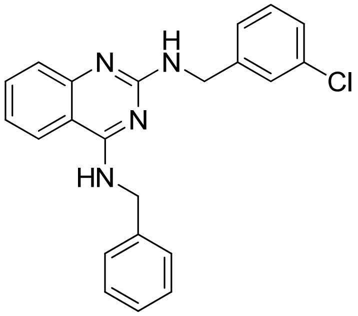

KSC-16-32

***N*[4]-Benzyl-*N*[2]-(3-chlorobenzyl)quinazoline-2,4-diamine (S119)**. Yield: 26.4 mg, 95%. ^{1}H NMR (400 MHz, $CDCl_3$) δ 7.52 – 7.44 (m, 1H), 7.41 (t, J = 8.0 Hz, 2H), 7.35 – 7.20 (m, 6H), 7.18 – 7.09 (m, 3H), 7.06 – 6.98 (m, 1H), 5.75 (s, br. 1H), 5.28 (s, br. 1H), 4.68 (d, J = 5.5 Hz, 2H), 4.62 (d, J = 6.1 Hz, 2H). ^{13}C NMR (101 MHz, $CDCl_3$) δ 160.1, 159.4, 152.1, 142.5, 138.6, 134.3, 132.8, 129.7, 128.8, 127.9, 127.6, 127.1, 125.7, 125.6, 121.3, 120.8, 111.1, 45.1, 45.0. HRMS (m/z): calcd for $C_{22}H_{20}ClN_4$ (M+H) 375.1376; found 375.1375.

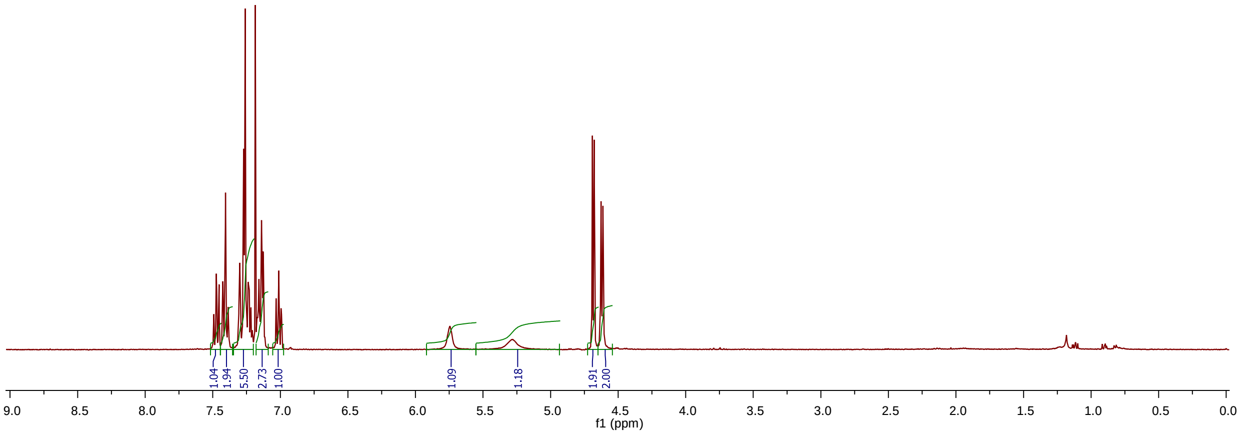

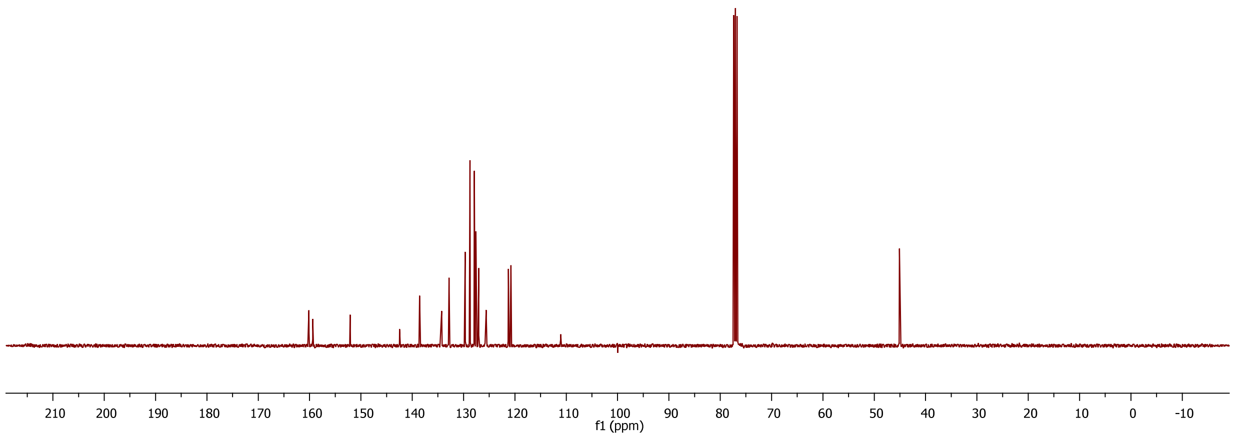

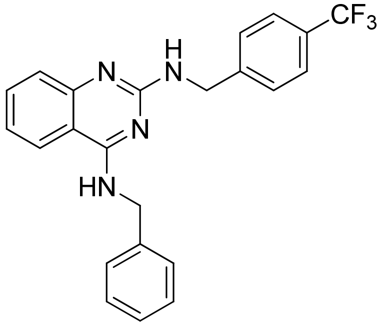

KSC-16-6

***N⁴*-Benzyl-*N²*-(4-(trifluoromethyl)benzyl)quinazoline-2,4-diamine (S120 or 46)**. Yield: 30.0 mg, 99%. ^{1}H NMR (400 MHz, $CDCl_3$) δ 7.59 – 7.48 (m, 7H), 7.33 – 7.31 (m, 5H), 7.11 (ddd, *J* = 1.3, 6.9, 8.2 Hz, 1H), 5.92 (s, br. 1H), 5.52 (s, br. 1H), 4.79 (d, *J* = 6.2 Hz, 2H), 4.77 (d, *J* = 5.5 Hz, 2H). ^{13}C NMR (101 MHz, $CDCl_3$) δ 160.1, 159.4, 152.1, 144.6, 138.5, 132.9, 129.2, 128.9, 128.8, 127.8, 127.6, 125.7, 125.6, 125.4, 125.34, 125.30, 125.26, 122.9, 121.4, 120.8, 111.1, 45.05. HRMS (m/z): calcd for $C_{23}H_{20}F_3N_4$ (M+H) 409.1640; found 409.1637.

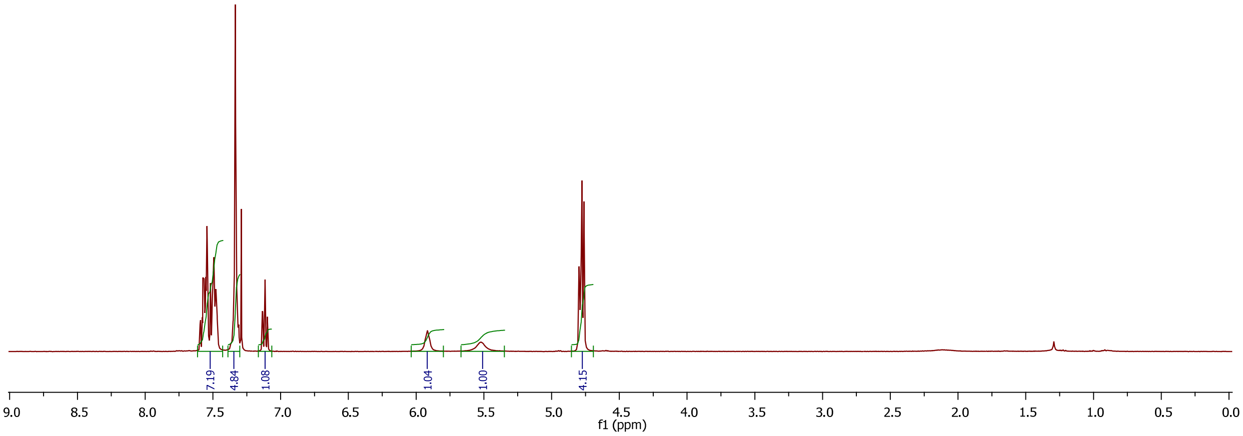

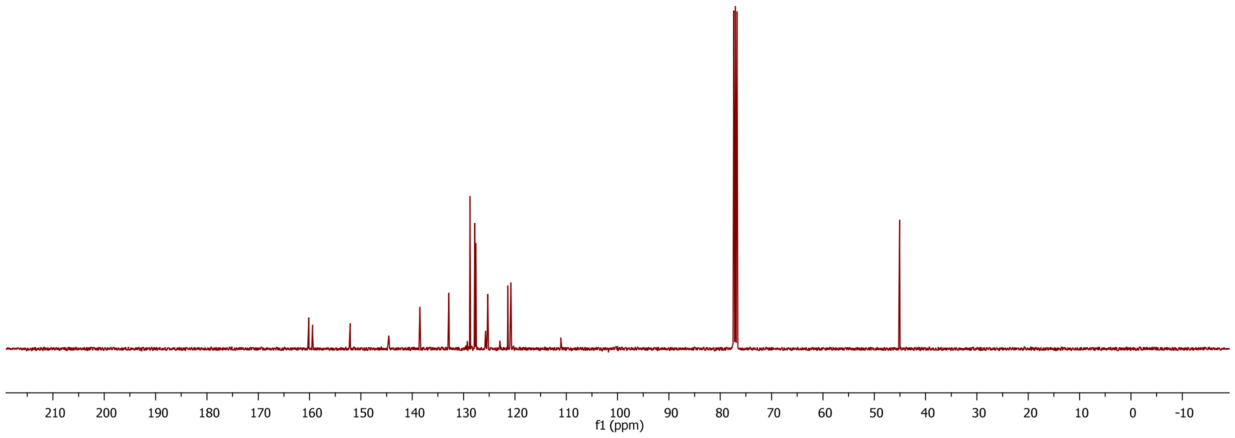

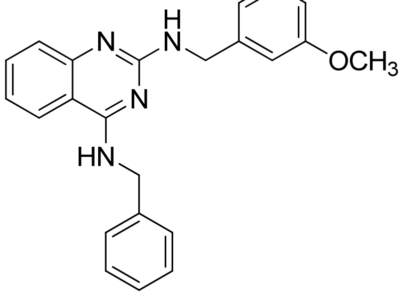

KSC-16-30

N^{4}**-Benzyl-*N***^{2}**-(3-methoxybenzyl)quinazoline-2,4-diamine (S121 or 47)**. Yield: 27.0 mg, 98%. ^{1}H NMR (400 MHz, $CDCl_3$) δ 7.63 – 7.45 (m, 3H), 7.43 – 7.30 (m, 5H), 7.25 (t, J = 7.8 Hz, 1H), 7.09 (ddd, J = 1.3, 6.8, 8.1 Hz, 1H), 6.99 (d, J = 8.1 Hz, 2H), 6.82 (dd, J = 1.9, 8.2 Hz, 1H), 5.89 (s, br. 1H), 5.36 (s, br. 1H), 4.80 (d, J = 5.5 Hz, 2H), 4.73 (d, J = 5.1 Hz, 2H), 3.80 (s, 3H). ^{13}C NMR (101 MHz, $CDCl_3$) δ 160.1, 159.8, 159.5, 152.2, 141.8, 138.7, 132.8, 129.5, 128.7, 128.0, 127.6, 125.7, 121.1, 120.8, 119.9, 113.1, 112.6, 111.1, 55.2, 45.6, 45.1. HRMS (m/z): calcd for $C_{23}H_{23}N_4O$ (M+H) 371.1872; found 371.1870.

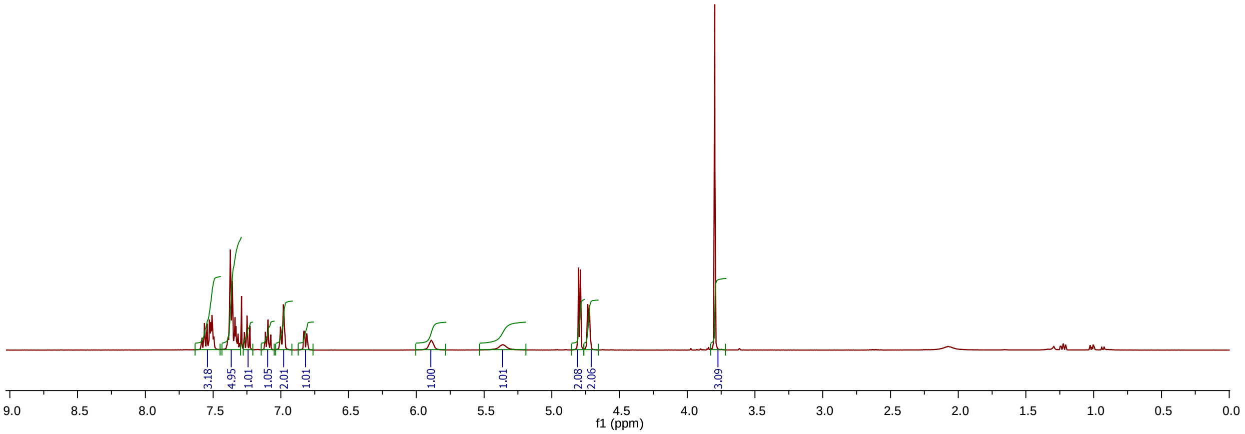

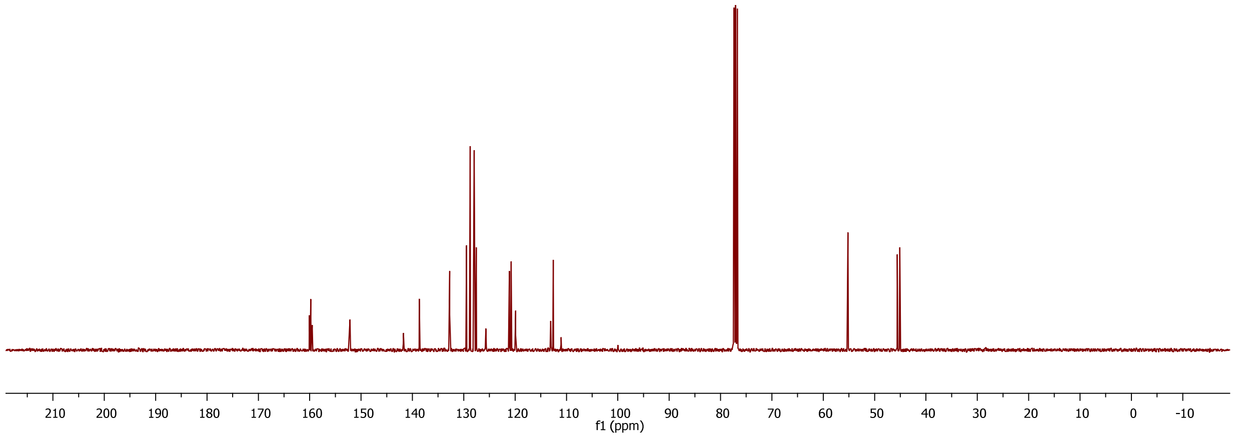

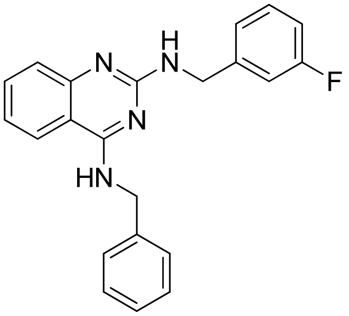

KSC-16-36

***N*[4]-Benzyl-*N*[2]-(3-fluorobenzyl)quinazoline-2,4-diamine (S122 or 48)**. Yield: 25.0 mg, 94%. ^{1}H NMR (400 MHz, $CDCl_3$) δ 7.57 (ddd, *J* = 1.3, 6.8, 8.2 Hz, 1H), 7.51 (dd, *J* = 8.3, 11.7 Hz, 2H), 7.42 – 7.30 (m, 5H), 7.29 – 7.23 (m, 1H), 7.20 – 7.04 (m, 3H), 6.94 (td, *J* = 1.9, 8.3 Hz, 1H), 5.92 (s, br. 1H), 5.44 (s, br. 1H), 4.78 (d, *J* = 5.5 Hz, 2H), 4.74 (d, *J* = 5.9 Hz, 2H). ^{13}C NMR (101 MHz, $CDCl_3$) δ 164.2, 161.8, 160.1, 159.4, 152.1, 143.1, 143.0, 138.6, 132.8, 129.9, 129.8, 128.8, 127.9, 127.6, 125.7, 123.0, 121.3, 120.8, 114.4, 114.2, 113.8, 113.6, 111.1, 45.1, 45.02, 45.00. HRMS (m/z): calcd for $C_{22}H_{20}FN_4$ (M+H) 359.1672; found 359.1671.

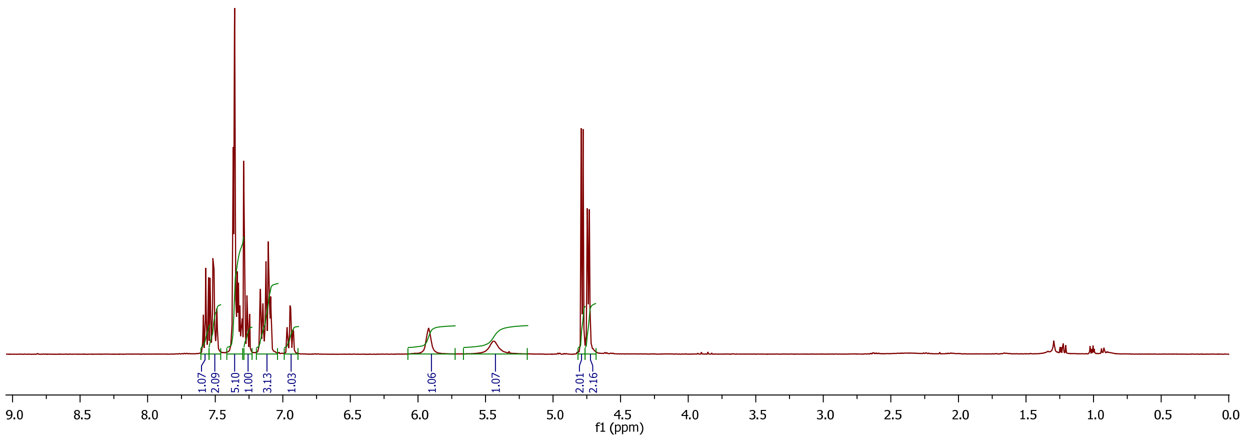

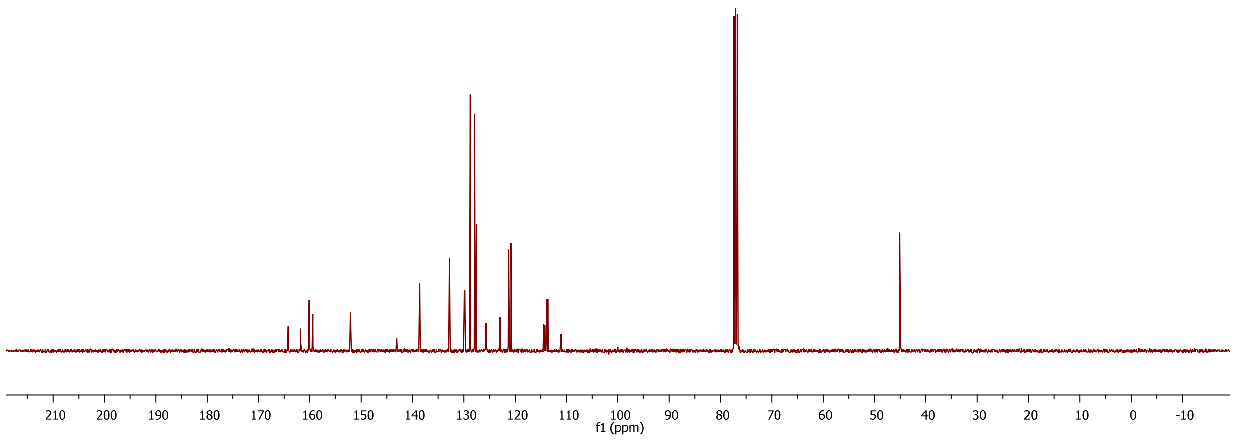

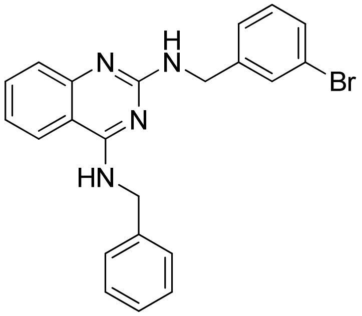

KSC-16-41

***N*[4]-Benzyl-*N*[2]-(3-bromobenzyl)quinazoline-2,4-diamine (S123 or 49)**. Yield: 29.4 mg, 95%. ^{1}H NMR (400 MHz, $CDCl_3$) δ 7.62 – 7.54 (m, 2H), 7.51 (t, *J* = 7.4 Hz, 2H), 7.43 – 7.30 (m, 7H), 7.18 (t, *J* = 7.8 Hz, 1H), 7.15 – 7.07 (m, 1H), 5.82 (s, br. 1H), 5.33 (s, br. 1H), 4.79 (d, *J* = 5.4 Hz, 2H), 4.72 (d, *J* = 6.0 Hz, 2H). ^{13}C NMR (101 MHz, $CDCl_3$) δ 160.1, 159.4, 152.1, 142.8, 138.6, 132.8, 130.5, 130.0, 128.8, 127.9, 127.6, 126.1, 125.7, 122.5, 121.3, 120.8, 111.1, 45.1, 44.9. HRMS (m/z): calcd for $C_{22}H_{20}BrN_4$ (M+H) 419.0871 and 421.0851; found 419.0866.

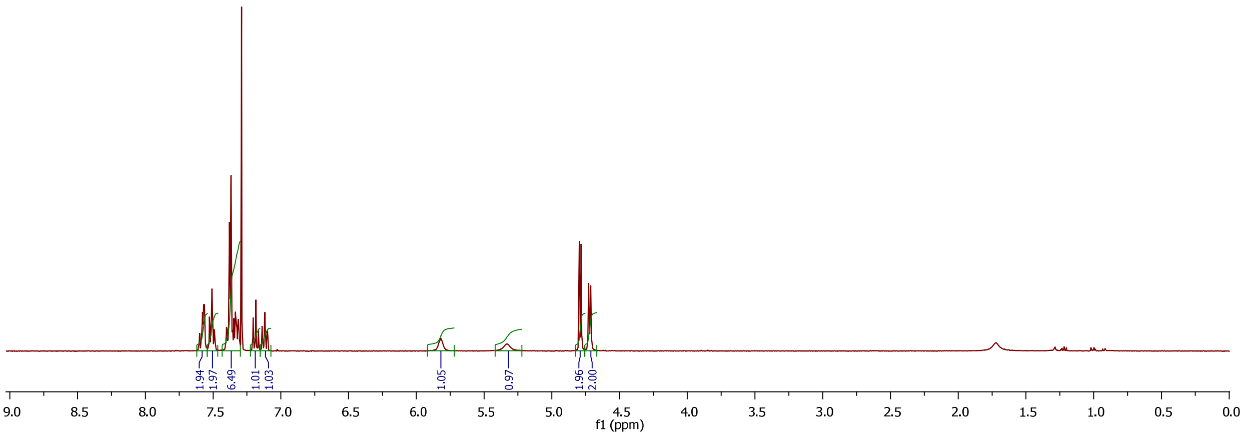

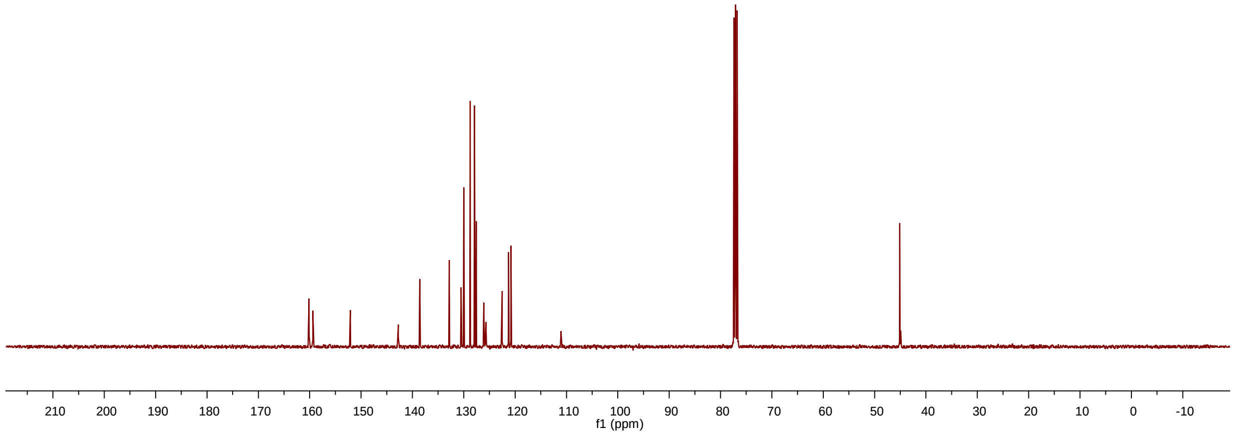

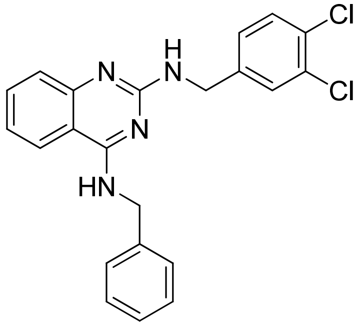

KSC-16-3

***N*[4]-Benzyl-*N*[2]-(3,4-dichlorobenzyl)quinazoline-2,4-diamine (S124)**. Yield: 30.0 mg, 99%. ^{1}H NMR (400 MHz, $CDCl_3$) δ 7.57 (ddd, *J* = 1.4, 6.9, 8.3 Hz, 1H), 7.54 – 7.47 (m, 3H), 7.40 – 7.30 (m, 6H), 7.18 (d, *J* = 8.1 Hz, 1H), 7.11 (ddd, *J* = 1.3, 6.9, 8.2 Hz, 1H), 5.97 (s, br. 1H), 5.59 (s, br. 1H), 4.77 (d, *J* = 5.5 Hz, 2H), 4.66 (d, *J* = 6.0 Hz, 2H). ^{13}C NMR (101 MHz, $CDCl_3$) δ 160.2, 159.3, 152.0, 140.9, 138.5, 132.9, 132.3, 130.6, 130.3, 129.3, 128.8, 127.8, 127.6, 126.8, 125.7, 121.4, 120.8, 111.1, 45.1, 44.4. HRMS (m/z): calcd for $C_{22}H_{19}Cl_2N_4$ (M+H) 409.0987; found 409.0980.

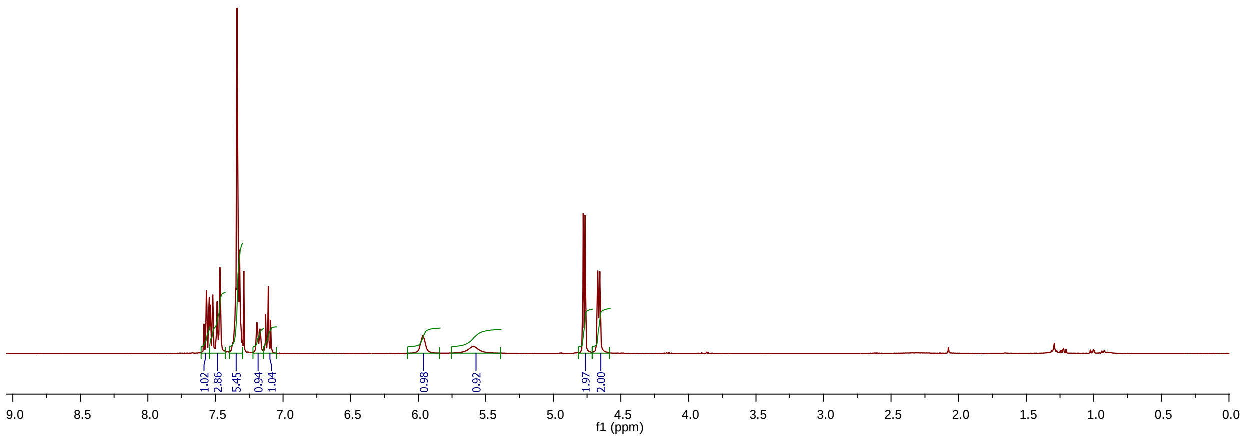

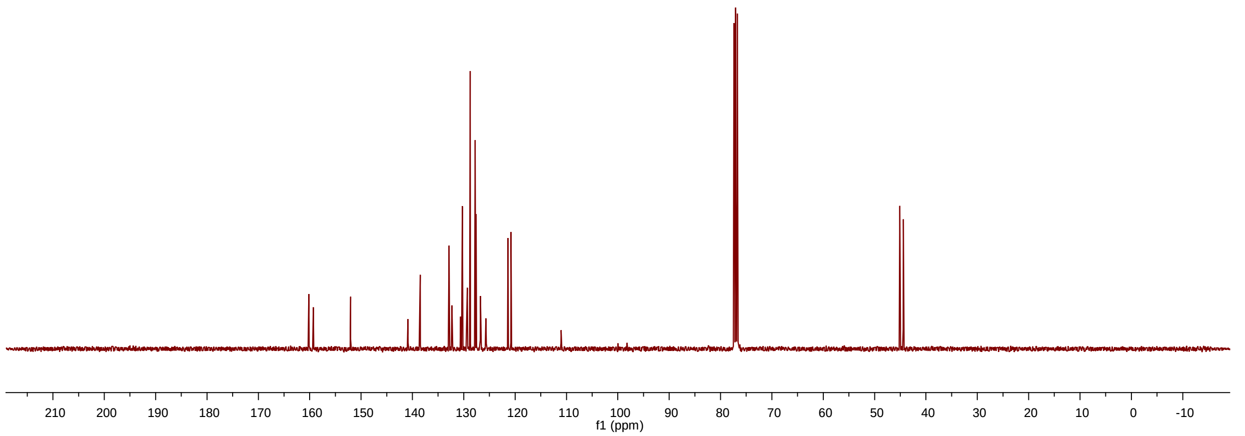

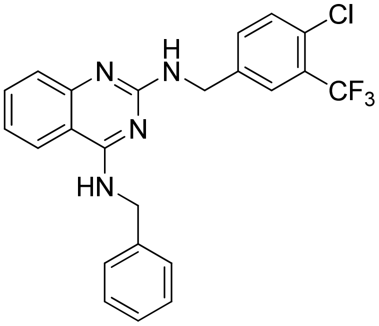

KSC-16-4

***N*[4]-Benzyl-*N*[2]-(4-chloro-3-(trifluoromethyl)benzyl)quinazoline-2,4-diamine (S125)**. Yield: 32.0 mg, 97%. ^{1}H NMR (400 MHz, $CDCl_3$) δ 7.61 (s, 1H), 7.46 (ddd, J = 1.4, 6.9, 8.3 Hz, 1H), 7.42 (d, J = 8.3 Hz, 1H), 7.39 – 7.32 (m, 2H), 7.32 – 7.19 (m, 6H), 7.01 (ddd, J = 1.2, 6.9, 8.2 Hz, 1H), 5.83 (s, br. 1H), 5.50 (s, br. 1H), 4.65 (d, J = 5.5 Hz, 2H), 4.61 (d, J = 5.5 Hz, 2H). ^{13}C NMR (101 MHz, $CDCl_3$) δ 160.2, 159.3, 152.0, 139.8, 138.4, 132.9, 131.8, 131.4, 130.4, 128.8, 128.3, 128.0, 127.7, 127.6, 126.62, 126.58, 125.7, 124.3, 121.6, 121.5, 120.8, 111.1, 45.1, 44.6. HRMS (m/z): calcd for $C_{23}H_{19}ClF_3N_4$ (M+H) 443.1250; found 443.1246.

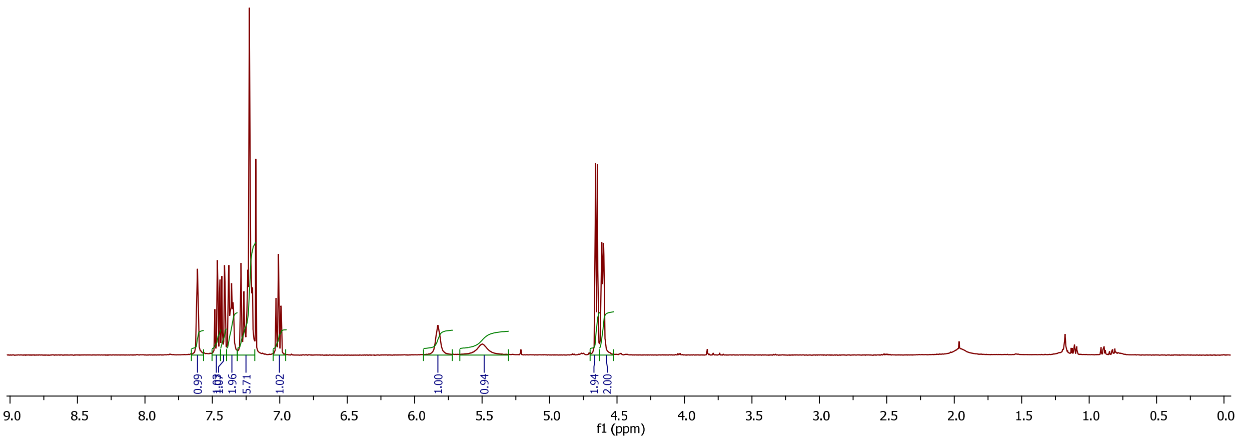

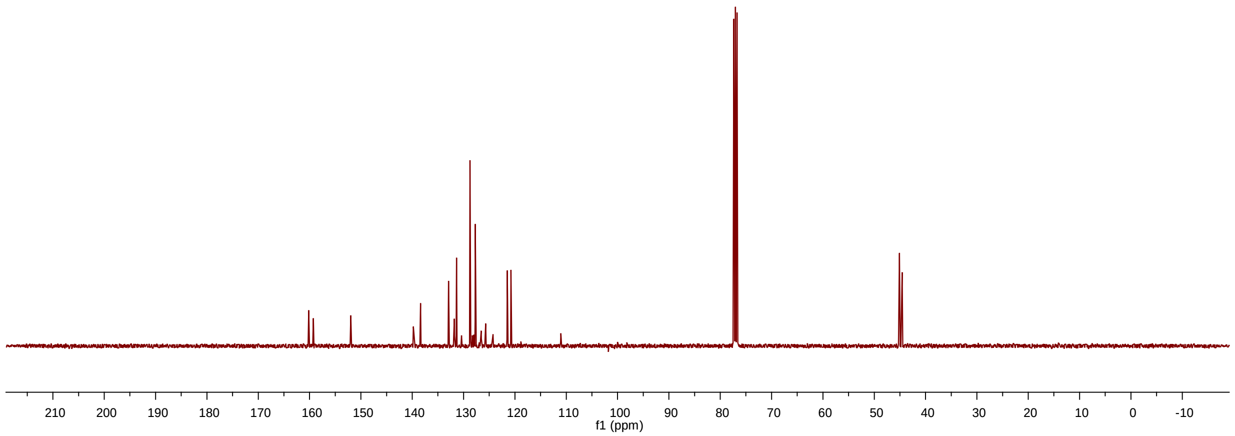

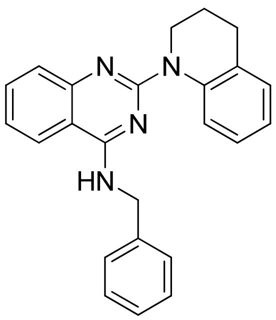

KSC-16-125

***N*-Benzyl-2-(3,4-dihydroquinolin-1(*2H*)-yl)quinazolin-4-amine (S142)**. Yield: 12.4 mg, 91%. ^{1}H NMR (400 MHz, $CDCl_3$) δ 7.76 (d, *J* = 7.2 Hz, 1H), 7.50 (dd, *J* = 1.0, 4.5 Hz, 2H), 7.46 (d, *J* = 8.2 Hz, 1H), 7.33 – 7.20 (m, 5H), 7.10 – 7.00 (m, 2H), 6.94 (dd, *J* = 4.1, 11.4 Hz, 1H), 6.87 (td, *J* = 1.2, 7.3 Hz, 1H), 5.73 (s, br. 1H), 4.69 (d, *J* = 5.5 Hz, 2H), 4.23 – 4.03 (m, 2H), 2.75 (t, *J* = 6.6 Hz, 2H), 1.94 (dt, *J* = 6.6, 12.7 Hz, 2H). ^{13}C NMR (101 MHz, $CDCl_3$) δ 159.5, 158.1, 152.0, 140.5, 138.8, 132.6, 130.3, 128.7, 128.4, 127.8, 127.5, 126.8, 125.1, 124.9, 122.3, 121.8, 120.6, 111.3, 45.2, 45.1, 27.7, 24.1. HRMS (m/z): calcd for $C_{24}H_{23}N_4$ (M+H) 367.1923; found 367.1917.

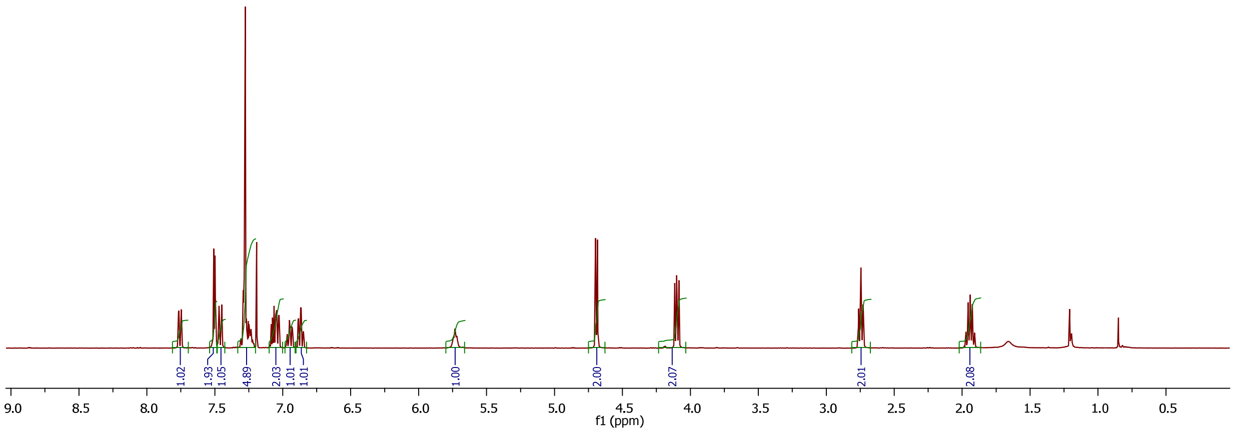

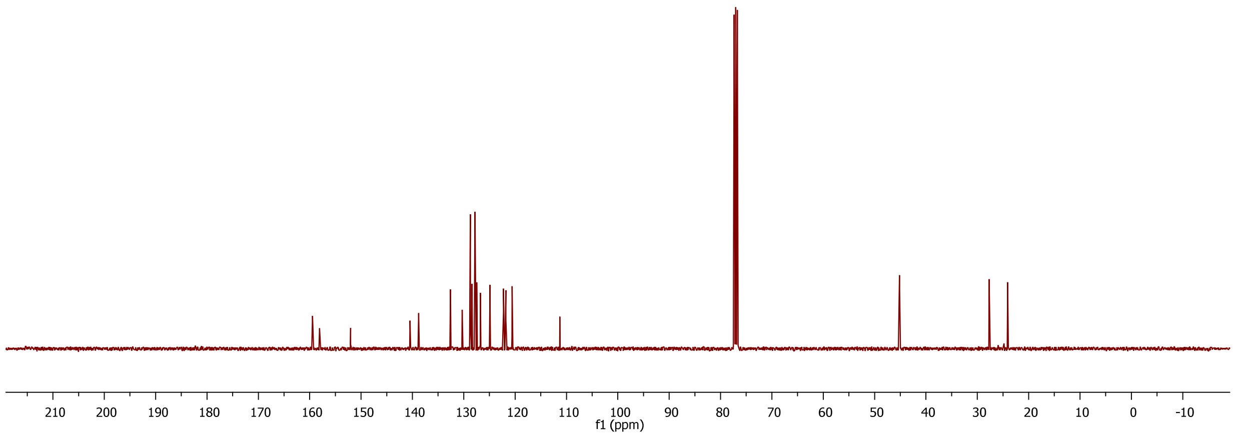

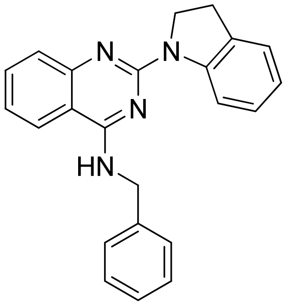

KSC-16-144

***N*-Benzyl-2-(indolin-1-yl)quinazolin-4-amine (S143)**. Yield: 13.0 mg, 99%. ^{1}H NMR (400 MHz, $CDCl_3$) δ 8.50 (d, *J* = 8.0 Hz, 1H), 7.76 – 7.53 (m, 3H), 7.52 – 7.32 (m, 5H), 7.23 – 7.11 (m, 3H), 6.91 (t, *J* = 7.4 Hz, 1H), 5.90 (s, 1H), 4.94 (d, *J* = 5.4 Hz, 2H), 4.45 – 4.28 (m, 2H), 3.19 (t, *J* = 8.7 Hz, 2H). ^{13}C NMR (101 MHz, $CDCl_3$) δ 159.7, 156.6, 151.9, 144.4, 138.6, 132.7, 132.4, 128.8, 127.9, 127.6, 127.1, 126.7, 124.5, 121.8, 120.8, 120.7, 115.4, 110.9, 49.0, 45.7, 27.3. HRMS (m/z): calcd for $C_{23}H_{21}N_4$ (M+H) 353.1766; found 353.1761.

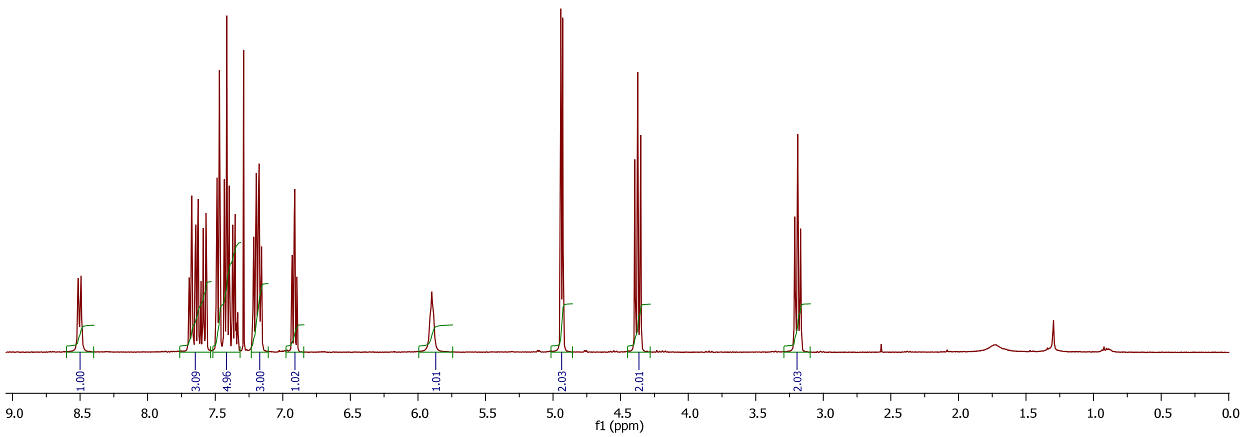

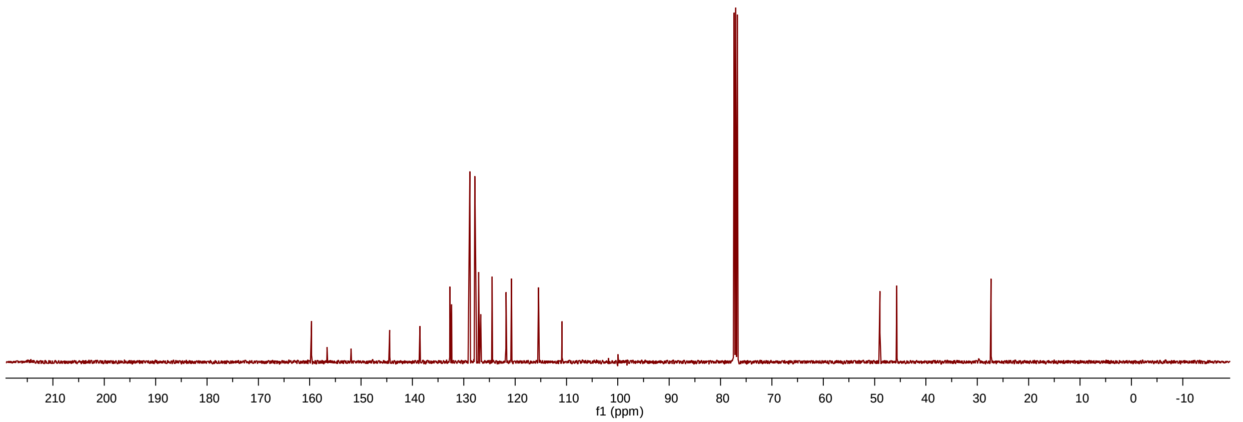

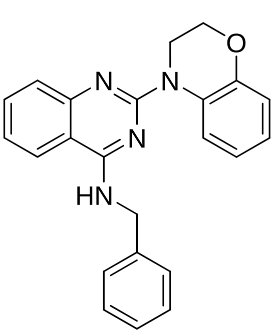

KSC-16-70

2-(*2H*-Benzo[*b*][1,4]oxazin-4(*3H*)-yl)-N-benzylquinazolin-4-amine (S144 or 15). Yield: 27.0 mg, 99%. ^{1}H NMR (400 MHz, $CDCl_3$) δ 8.12 (d, *J* = 8.3 Hz, 1H), 7.68 – 7.61 (m, 2H), 7.58 (d, *J* = 8.2 Hz, 1H), 7.45 – 7.30 (m, 5H), 7.25 – 7.12 (m, 1H), 7.03 – 6.89 (m, 2H), 6.88 – 6.77 (m, 1H), 5.94 (s, 1H), 4.84 (d, *J* = 5.5 Hz, 2H), 4.39 (dd, *J* = 3.1, 5.1 Hz, 2H), 4.34 (dd, *J* = 3.1, 5.0 Hz, 2H). ^{13}C NMR (101 MHz, $CDCl_3$) δ 159.8, 156.7, 151.7, 146.3, 138.5, 132.8, 128.8, 128.1, 127.7, 127.6, 126.8, 124.7, 123.4, 122.3, 120.7, 119.4, 116.7, 111.3, 66.2, 45.3, 42.2. HRMS (m/z): calcd for $C_{23}H_{21}N_4O$ (M+H) 369.1715; found 369.1716.

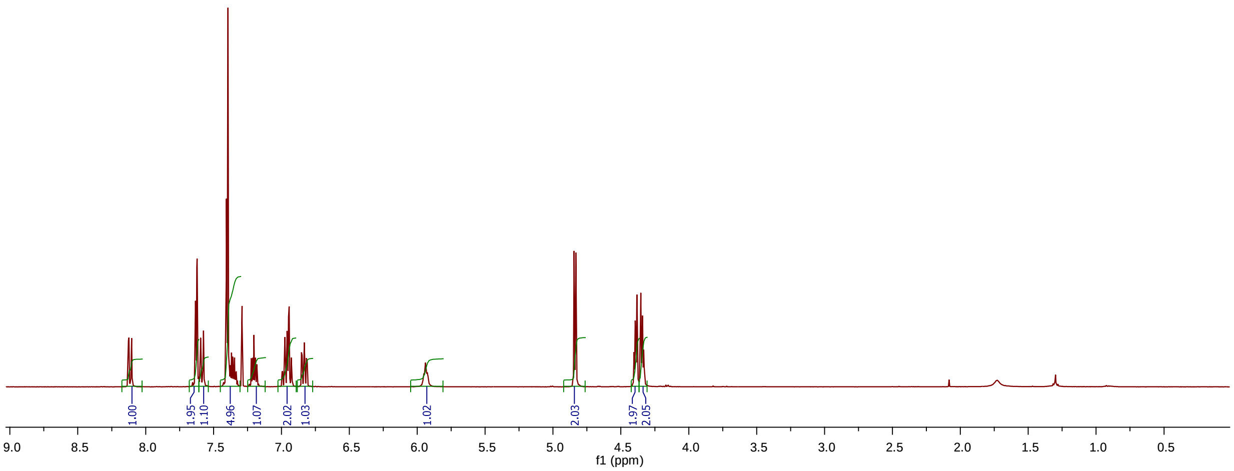

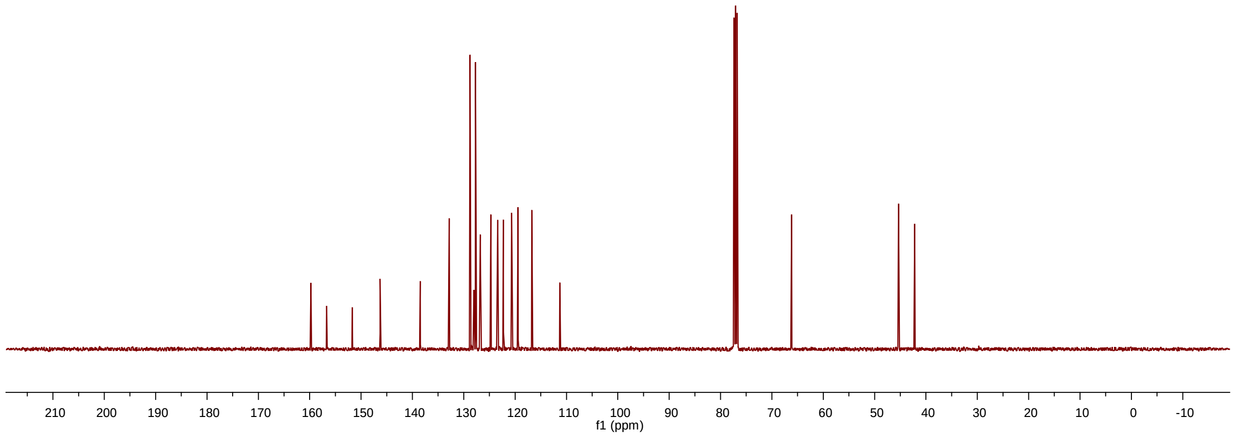

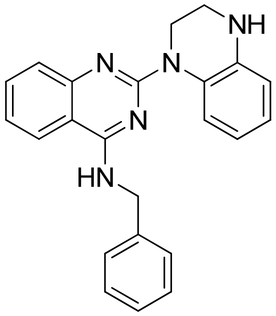

KSC-16-147

***N*-Benzyl-2-(3,4-dihydroquinoxalin-1(*2H*)-yl)quinazolin-4-amine (S145)**. Yield: 13.5 mg, 99%. ^{1}H NMR (400 MHz, $CDCl_3$) δ 7.82 (d, *J* = 7.8 Hz, 1H), 7.57 (dd, *J* = 6.2, 13.7 Hz, 3H), 7.45 – 7.31 (m, 5H), 7.21 – 7.09 (m, 1H), 6.94 – 6.84 (m, 1H), 6.62 (dd, *J* = 4.4, 12.2 Hz, 2H), 5.82 (s, br. 1H), 4.81 (d, *J* = 5.5 Hz, 2H), 4.40 – 4.25 (m, 2H), 3.55 – 3.45 (m, 2H). ^{13}C NMR (101 MHz, $CDCl_3$) δ 159.6, 157.2, 152.0, 138.7, 137.2, 132.6, 128.7, 127.8, 127.5, 126.7, 126.6, 125.7, 123.8, 121.7, 120.6, 116.1, 114.6, 111.2, 45.2, 42.4, 41.5. HRMS (m/z): calcd for $C_{23}H_{22}N_5$ (M+H) 368.1875; found 368.1874.

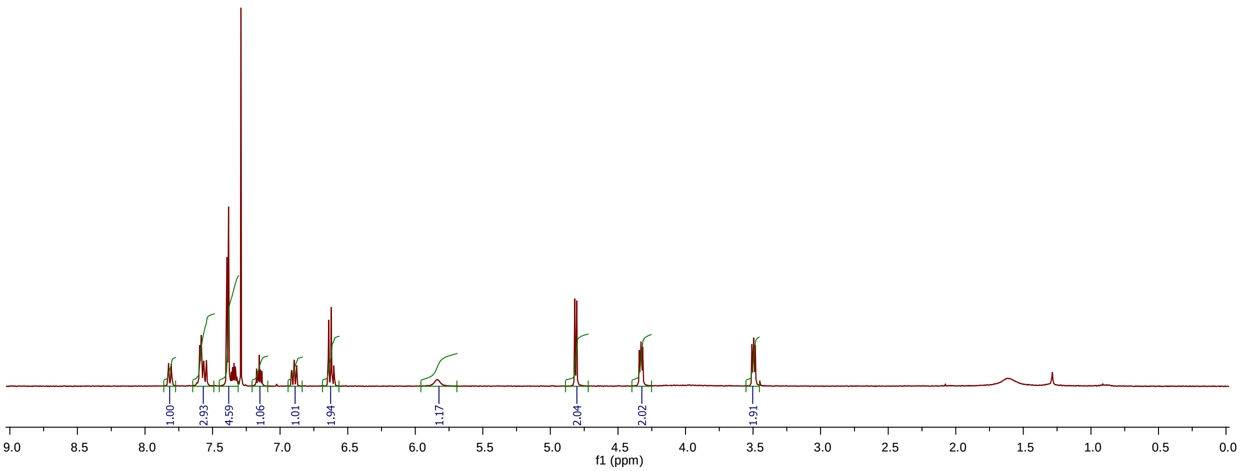

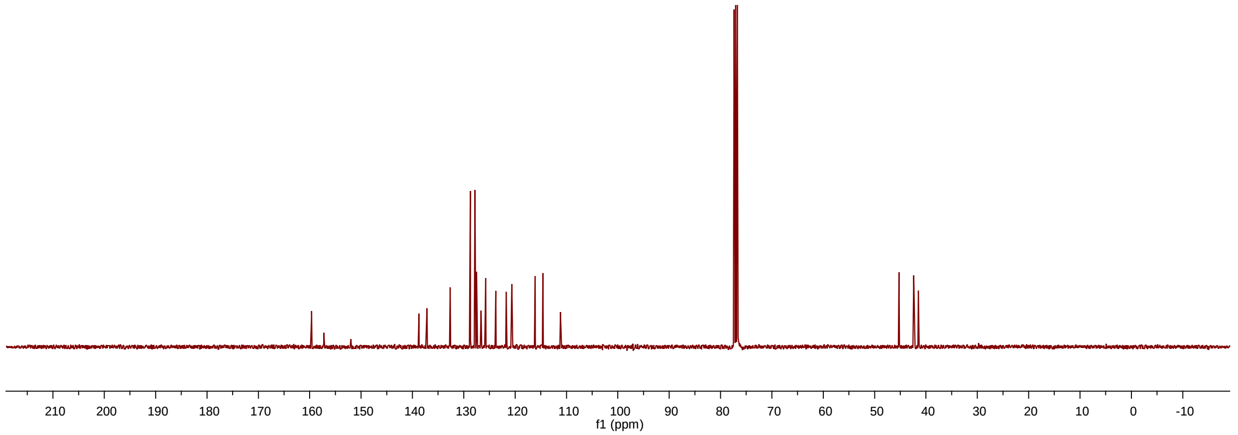

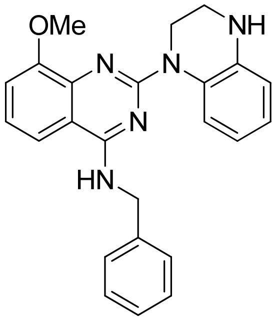

KSC-16-182

_N_-Benzyl-2-(3,4-dihydroquinoxalin-1(_2H_)-yl)-8-methoxyquinazolin-4-amine (S146). Yield: 5.6 mg, 42%. ^{1}H NMR (400 MHz, $CDCl_3$) δ 7.72 (d, J = 7.8 Hz, 1H), 7.37 – 7.21 (m, 5H), 7.04 (d, J = 7.8 Hz, 1H), 6.98 (t, J = 7.8 Hz, 1H), 6.92 (d, J = 7.7 Hz, 1H), 6.77 (t, J = 6.9 Hz, 1H), 6.59 – 6.44 (m, 2H), 5.68 (s, 1H), 4.69 (d, J = 5.4 Hz, 2H), 4.33 – 4.24 (m, 2H), 3.92 (s, 3H), 3.48 – 3.37 (m, 2H). ^{13}C NMR (101 MHz, $CDCl_3$) δ 159.6, 156.8, 153.8, 138.7, 137.0, 128.7, 127.9, 127.5, 126.7, 125.2, 123.5, 121.3, 118.8, 116.1, 114.5, 112.4, 111.7, 111.3, 56.2, 45.3, 42.5, 41.5. HRMS (m/z): calcd for $C_{24}H_{24}N_5O$ (M+H) 398.1981; found 398.1974.

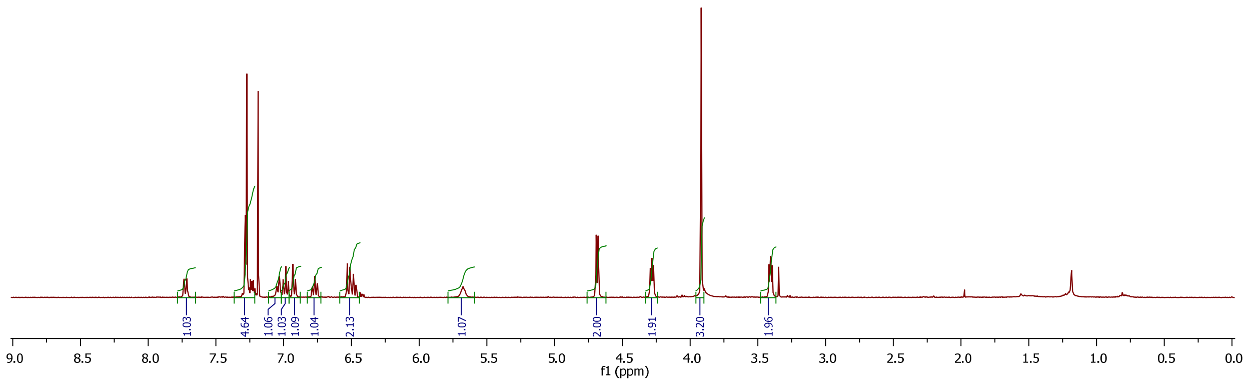

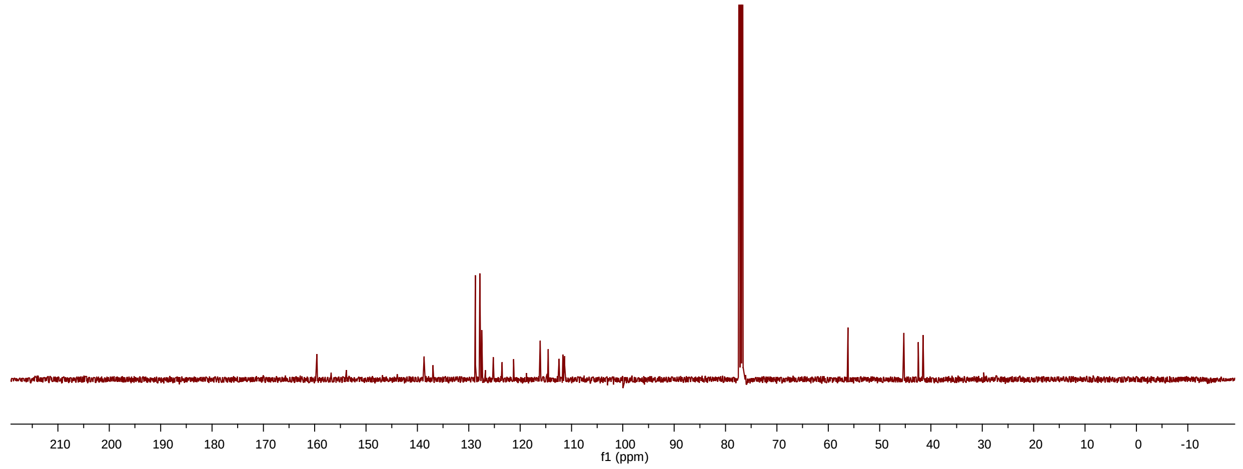

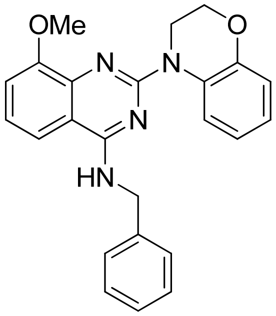

KSC-16-191

2-(*2H*-Benzo[*b*][1,4]oxazin-4(*3H*)-yl)-*N*-benzyl-8-methoxyquinazolin-4-amine (S147 or 38). Yield: 7.1 mg, 53%. ^{1}H NMR (400 MHz, $CDCl_3$) δ 8.21 – 8.08 (m, 1H), 7.46 – 7.31 (m, 5H), 7.18 7.11 (m, 2H), 7.05 (dd, *J* = 1.6, 7.3 Hz, 1H), 6.99 – 6.88 (m, 2H), 6.80 (ddd, *J* = 2.3, 6.5, 8.7 Hz, 1H), 5.86 (s, 1H), 4.83 (d, *J* = 5.5 Hz, 2H), 4.50 – 4.40 (m, 2H), 4.40 – 4.29 (m, 2H), 4.03 (s, 3H). ^{13}C NMR (101 MHz, $CDCl_3$) δ 159.7, 156.2, 153.9, 146.2, 143.6, 138.5, 128.8, 128.1, 127.8, 127.6, 124.3, 123.2, 121.9, 119.4, 116.7, 112.3, 111.8, 111.4, 66.2, 56.1, 45.4, 42.2. HRMS (m/z): calcd for $C_{24}H_{23}N_4O_2$ (M+H) 399.1821; found 399.1824.

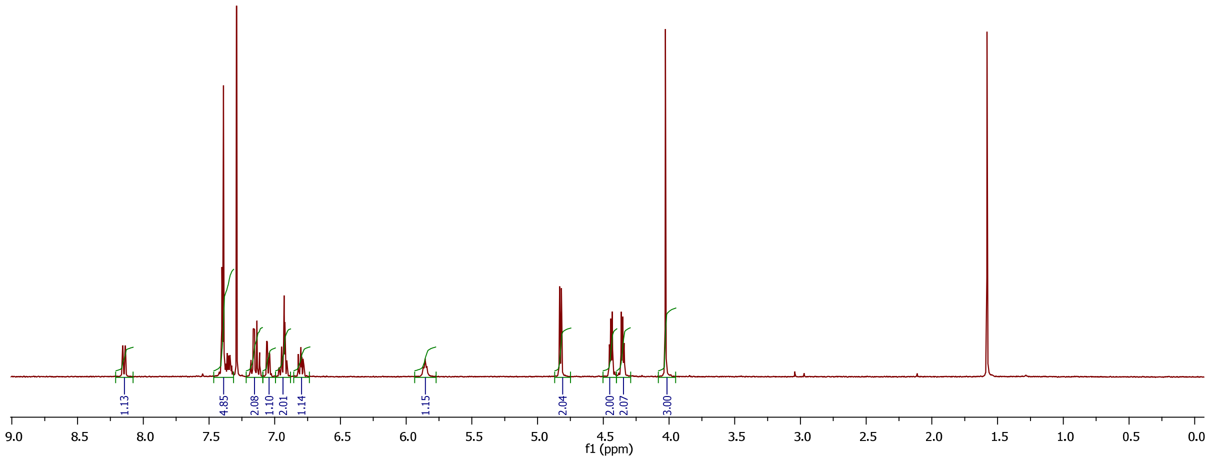

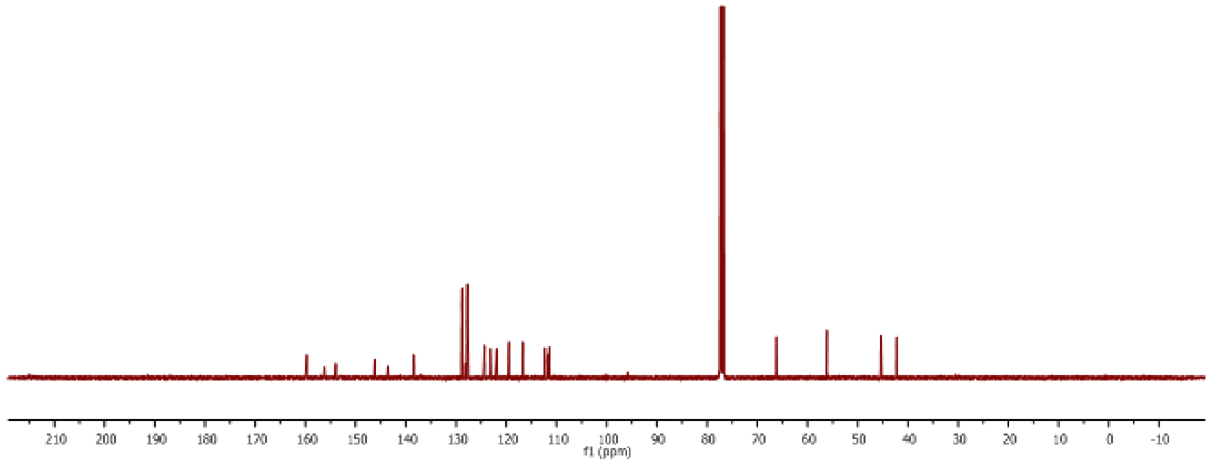

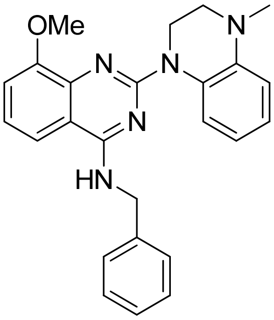

KSC-25-17

***N*-Benzyl-8-methoxy-2-(4-methyl-3,4-dihydroquinoxalin-1(2*H*)-yl)quinazolin-4-amine (S156)**. Yield: 10.0 mg, 73%. ^{1}H NMR (400 MHz, $CDCl_3$) δ 7.77 (dd, *J* = 1.4, 8.1 Hz, 1H), 7.44 – 7.30 (m, 5H), 7.14 (d, *J* = 6.9 Hz, 1H), 7.08 (t, *J* = 7.9 Hz, 1H), 7.05 – 7.01 (m, 1H), 6.96 (dd, *J* = 4.2, 11.2 Hz, 1H), 6.73 (dd, *J* = 1.3, 8.2 Hz, 1H), 6.58 (t, *J* = 7.6 Hz, 1H), 5.77 (s, 1H), 4.77 (d, *J* = 5.4 Hz, 2H), 4.47 – 4.36 (m, 2H), 4.02 (s, 3H), 3.49 – 3.38 (m, 2H), 2.99 (s, 3H). ^{13}C NMR (101 MHz, $CDCl_3$) δ 159.5, 156.8, 153.8, 143.9, 139.4, 138.7, 128.7, 127.9, 127.5, 127.1, 124.6, 123.7, 121.2, 115.3, 112.4, 111.7, 111.3, 111.1, 56.2, 51.0, 45.3, 41.8, 38.7. HRMS (m/z): calcd for $C_{25}H_{26}N_5O$ (M+H) 412.2137; found 412.2148.

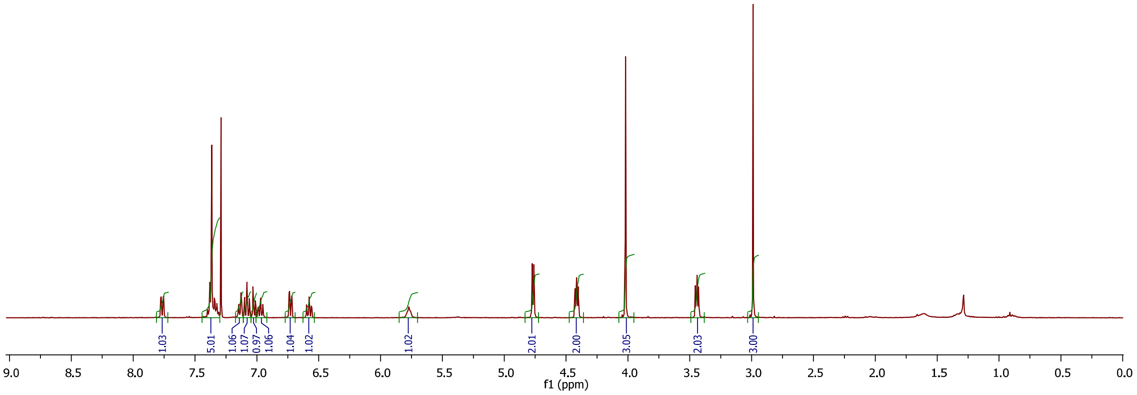

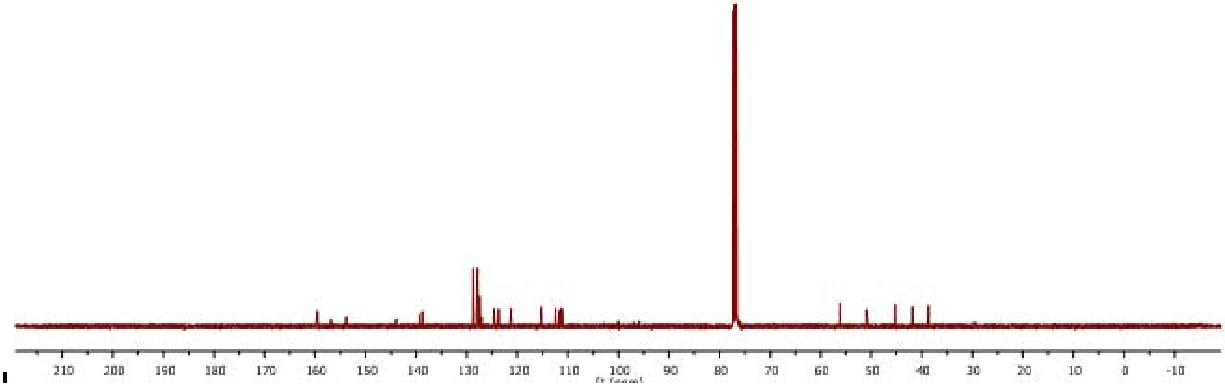

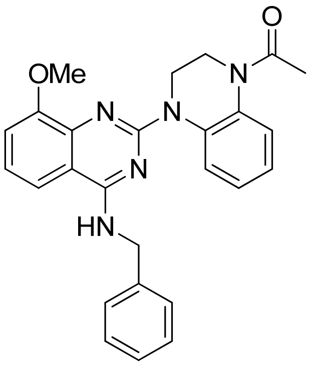

KSC-25-15c1

1-(4-(4-(Benzylamino)-8-methoxyquinazolin-2-yl)-3,4-dihydroquinoxalin-1(*2H*)-yl)ethanone (S157). Yield: 8.2 mg, 37%. ^{1}H NMR (400 MHz, $CDCl_3$) δ 7.34 – 7.21 (m, 5H), 7.15 – 6.87 (m, 6H), 5.98 – 5.73 (m, 1H), 4.70 (d, *J* = 5.4 Hz, 2H), 4.19 (s, br. 2H), 3.96 (s, br. 2H), 3.92 (s, 3H), 2.18 (s, 3H). HRMS (m/z): calcd for $C_{26}H_{26}N_5O_2$ (M+H) 440.2087; found 440.2096.

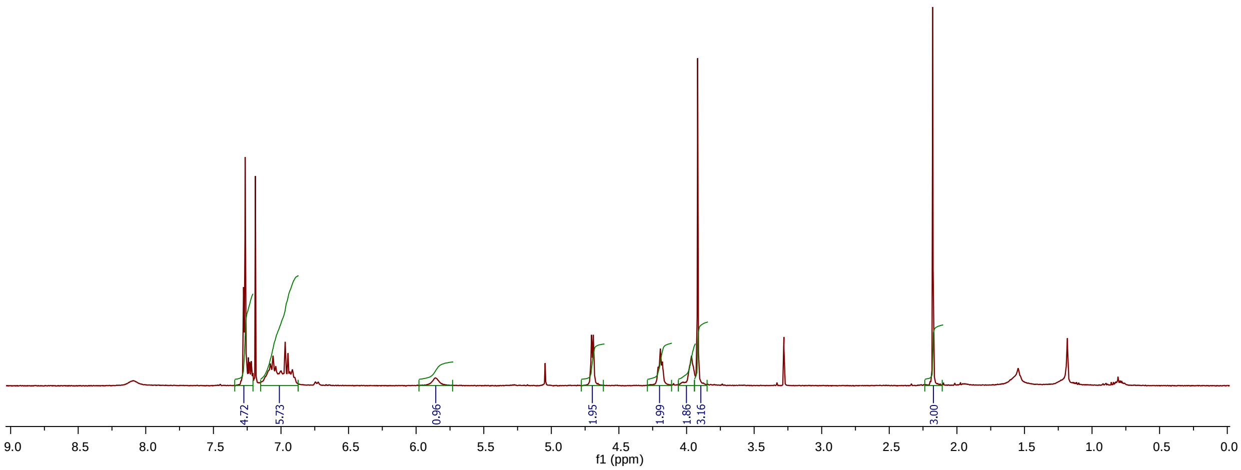

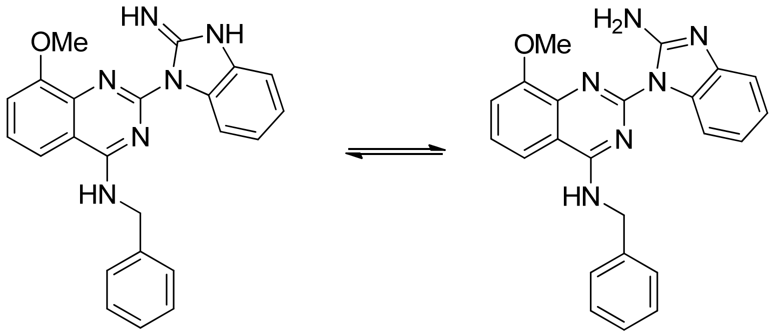

KSC-16-270 (probe)

2-(2-Amino-*1H*-benzo[*d*]imidazol-1-yl)-*N*-benzyl-8-methoxyquinazolin-4-amine (S158 or ML240). Yield: 14.6 mg, 55%. ^{1}H NMR (400 MHz, DMSO) δ 9.33 (t, *J* = 5.9 Hz, 1H), 8.31 – 8.05 (m, 3H), 7.93 (d, *J* = 7.5 Hz, 1H), 7.50 – 7.39 (m, 3H), 7.39 – 7.30 (m, 3H), 7.25 (t, *J* = 7.3 Hz, 1H), 7.15 (d, *J* = 7.1 Hz, 1H), 7.03 (td, *J* = 1.2, 7.6 Hz, 1H), 6.87 – 6.76 (m, 1H), 4.92 (d, *J* = 5.8 Hz, 2H), 3.98 (s, 3H). ^{13}C NMR (101 MHz, DMSO) δ 160.8, 154.7, 153.5, 153.4, 142.8, 140.0, 138.5, 131.5, 128.5, 126.9, 126.6, 124.9, 122.5, 118.7, 114.8, 114.6, 114.1, 113.0, 112.7, 56.1, 44.5. HRMS (m/z): calcd for $C_{23}H_{21}N_6O$ (M+H) 397.1777; found 397.1776.

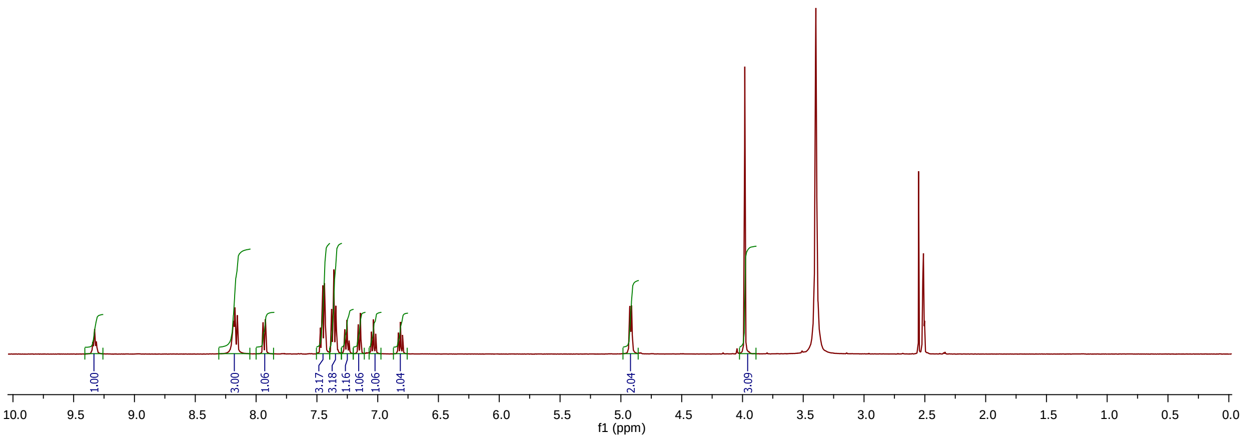

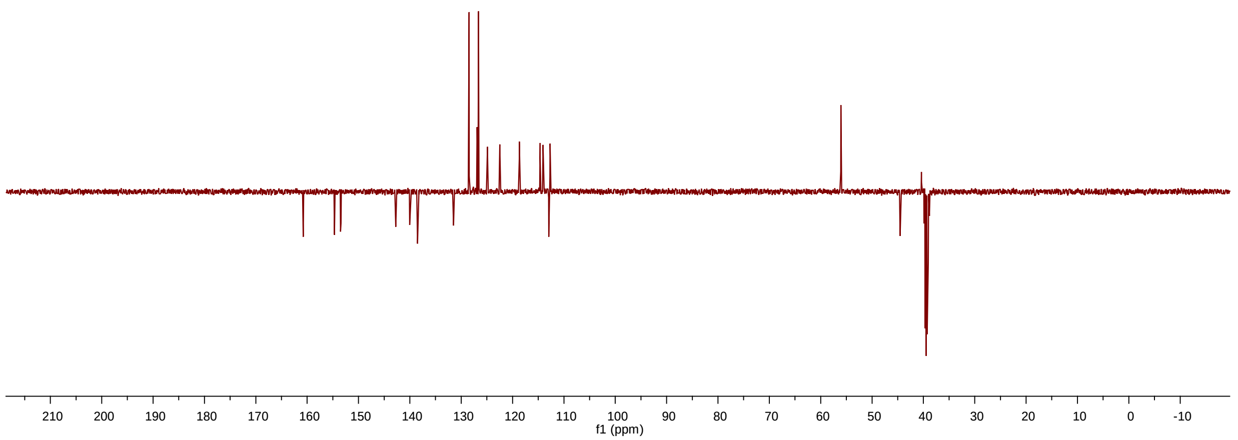

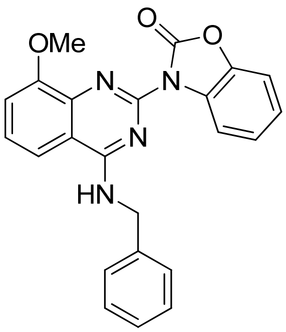

KSC-16-262

3-(4-(Benzylamino)-8-methoxyquinazolin-2-yl)benzo[*d*]oxazol-2(*3H*)-one (S159). Yield: 6.6 mg, 25%. ^{1}H NMR (400 MHz, DMSO) δ 9.22 (t, *J* = 6.0 Hz, 1H), 7.91 (d, *J* = 7.4 Hz, 1H), 7.83 (d, *J* = 7.9 Hz, 1H), 7.50 (t, *J* = 8.1 Hz, 1H), 7.45 (d, *J* = 7.0 Hz, 2H), 7.40 (d, *J* = 7.9 Hz, 1H), 7.38 – 7.31 (m, 3H), 7.27 (t, *J* = 7.3 Hz, 1H), 7.22 (t, *J* = 7.8 Hz, 1H), 7.15 (t, *J* = 7.8 Hz, 1H), 4.86 (d, *J* = 5.9 Hz, 2H), 3.98 (s, 3H). ^{13}C NMR (101 MHz, DMSO) δ 160.8, 154.3, 150.7, 150.1, 141.6, 141.2, 139.1, 129.3, 128.4, 127.4, 126.9, 125.8, 123.8, 123.4, 114.1, 114.0, 113.8, 113.0, 109.5, 56.0, 43.9. HRMS (m/z): calcd for $C_{23}H_{19}N_4O_3$ (M+H) 399.1457; found 399.1451.

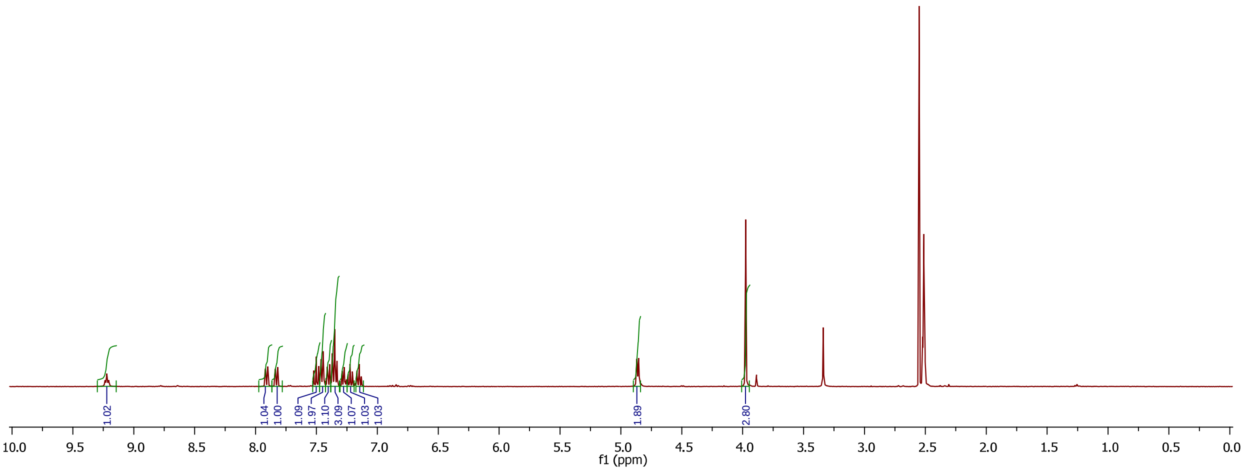

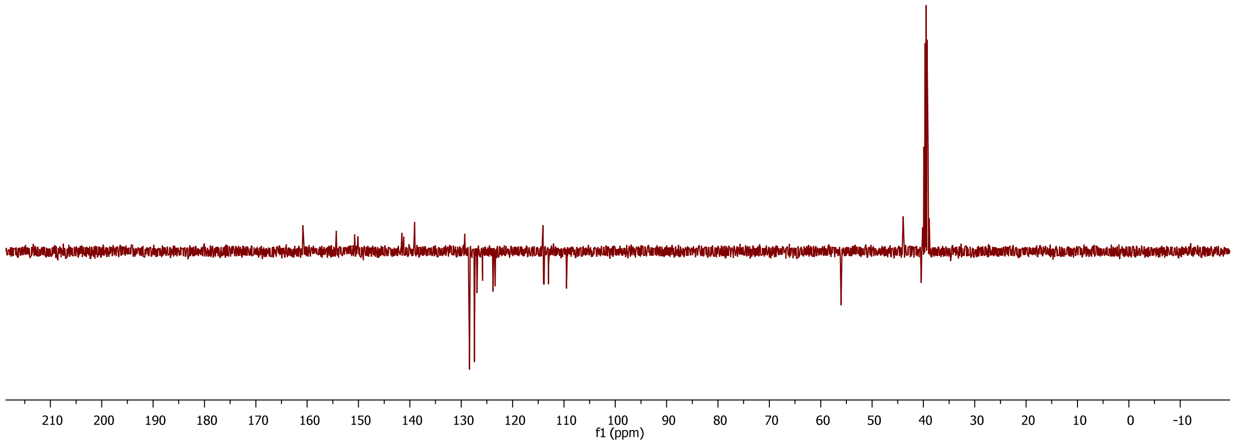

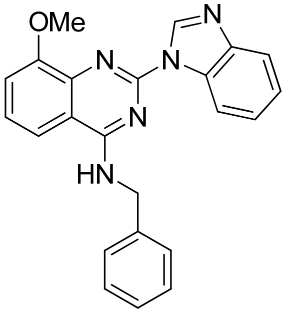

KSC-25-3

2-(*1H*-Benzo[*d*]imidazol-1-yl)-*N*-benzyl-8-methoxyquinazolin-4-amine (S160). Yield: 3.7 mg, 19%. ^{1}H NMR (500 MHz, DMSO) δ 9.27 (s, 1H), 9.08 (s, 1H), 8.72 (d, *J* = 7.5 Hz, 1H), 7.91 (d, *J* = 7.4 Hz, 1H), 7.74 (d, *J* = 7.4 Hz, 1H), 7.53 – 7.42 (m, 3H), 7.39 – 7.30 (m, 5H), 7.25 (d, *J* = 7.4 Hz, 1H), 4.92 (d, *J* = 5.8 Hz, 2H), 4.01 (s, 3H). ^{13}C NMR (126 MHz, DMSO) δ 160.6, 154.3, 151.5, 144.5, 142.1, 141.2, 139.2, 131.8, 128.4, 127.2, 126.9, 125.1, 124.0, 123.1, 119.6, 115.9, 114.14, 114.12, 113.0, 56.1, 44.3. HRMS (m/z): calcd for $C_{23}H_{20}N_5O$ (M+H) 382.1668; found 382.1658.

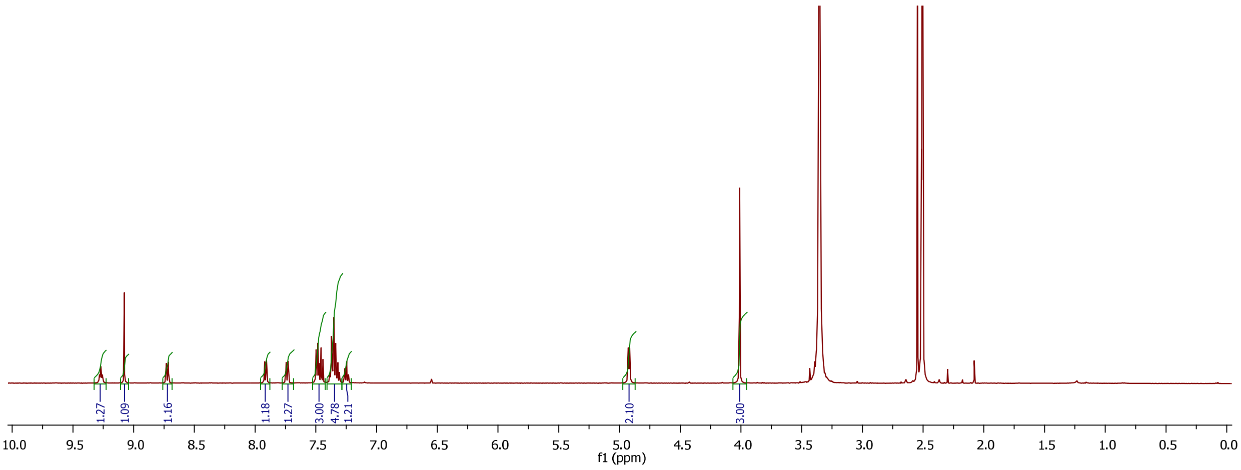

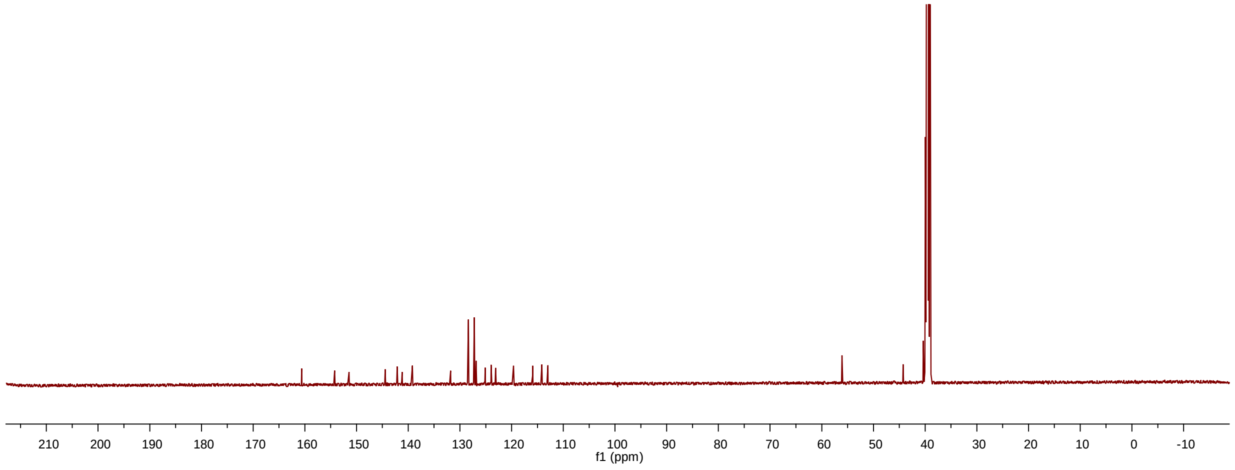

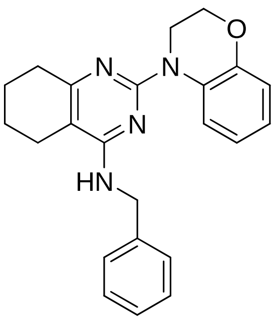

KSC-16-290

2-(*2H*-Benzo[*b*][1,4]oxazin-4(*3H*)-yl)-*N*-benzyl-5,6,7,8-tetrahydroquinazolin-4-amine (ML241). Yield: 9.0 mg, 66%. ^{1}H NMR (400 MHz, $CDCl_3$) δ 8.09 – 8.01 (m, 1H), 7.42 – 7.30 (m, 5H), 6.92 – 6.86 (m, 2H), 6.80 – 6.73 (m, 1H), 4.79 (s, 1H), 4.69 (d, *J* = 5.6 Hz, 2H), 4.28 (dd, *J* = 3.1, 5.0 Hz, 2H), 4.23 (dd, *J* = 3.1, 5.0 Hz, 2H), 2.67 (t, *J* = 5.7 Hz, 2H), 2.32 (t, *J* = 5.7 Hz, 2H), 1.93 – 1.77 (m, 4H). ^{13}C NMR (101 MHz, $CDCl_3$) δ 162.5, 160.7, 157.3, 145.9, 139.5, 128.6, 128.5, 127.5, 127.3, 123.9, 122.6, 119.5, 116.6, 103.8, 65.9, 45.0, 42.2, 32.2, 22.6, 22.5, 21.9. HRMS (m/z): calcd for $C_{23}H_{25}N_4O$ (M+H) 373.2028; found 373.2030.

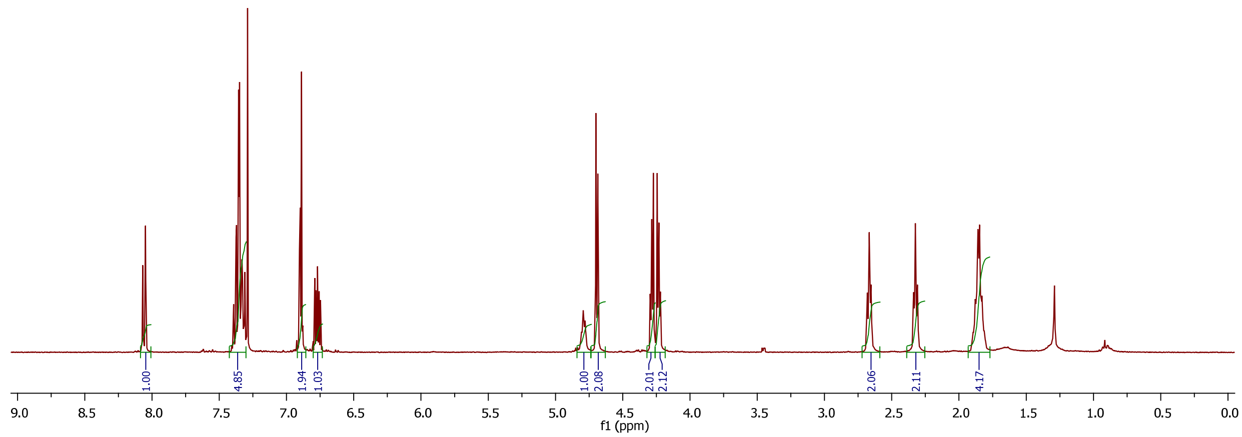

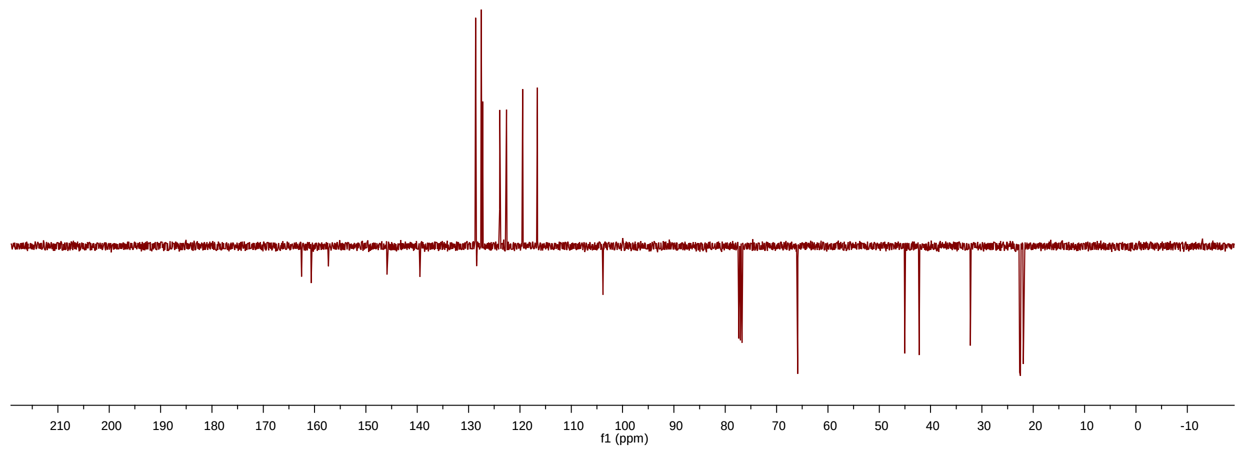

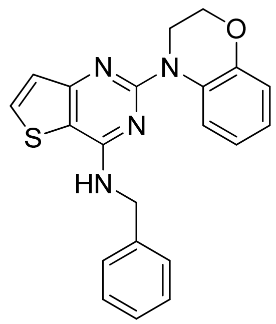

KSC-25-14

2-(*2H*-Benzo[*b*][1,4]oxazin-4(*3H*)-yl)-N-benzylthieno[3,2-*d*]pyrimidin-4-amine (S162 or 17). Yield: 8.2 mg, 41%. ^{1}H NMR (500 MHz, DMSO) δ 8.39 (t, *J* = 5.9 Hz, 1H), 8.01 (d, *J* = 5.3 Hz, 1H), 7.86 (dd, *J* = 1.5, 8.3 Hz, 1H), 7.36 – 7.29 (m, 4H), 7.28 – 7.21 (m, 1H), 7.19 (d, *J* = 5.3 Hz, 1H), 6.90 – 6.84 (m, 1H), 6.82 (dd, *J* = 1.7, 8.1 Hz, 1H), 6.68 (ddd, *J* = 1.7, 7.1, 8.6 Hz, 1H), 4.66 (d, *J* = 5.9 Hz, 2H), 4.23 – 4.16 (m, 2H), 4.15 – 4.13 (m, 2H). ^{13}C NMR (126 MHz, DMSO) δ 160.9, 157.7, 156.7, 145.6, 139.7, 133.0, 128.3, 128.2, 127.0, 126.6, 124.3, 123.7, 122.7, 119.1, 116.2, 107.6, 65.3, 43.4, 42.1. HRMS (m/z): calcd for $C_{21}H_{19}N_4OS$ (M+H) 375.1280; found 375.1284.

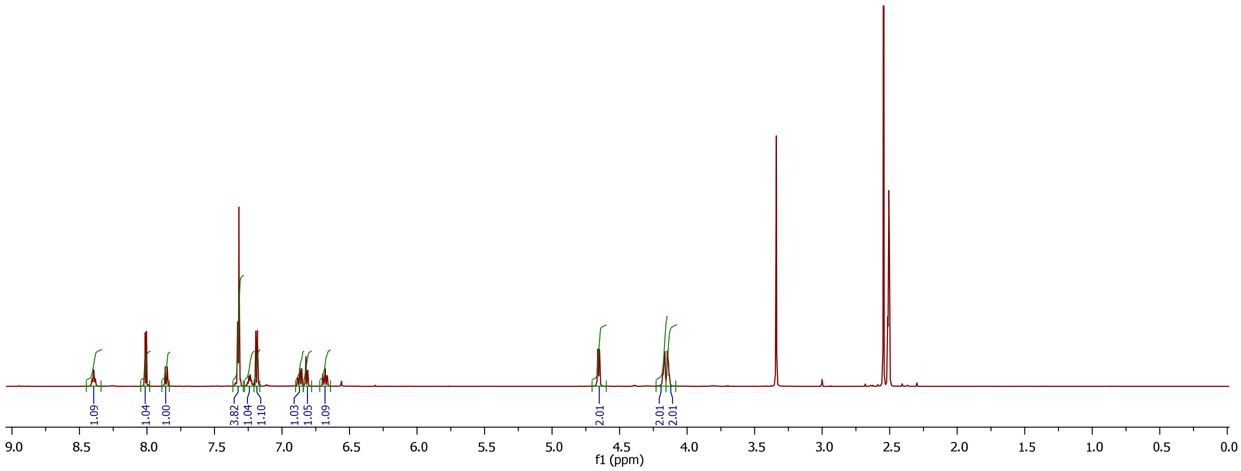

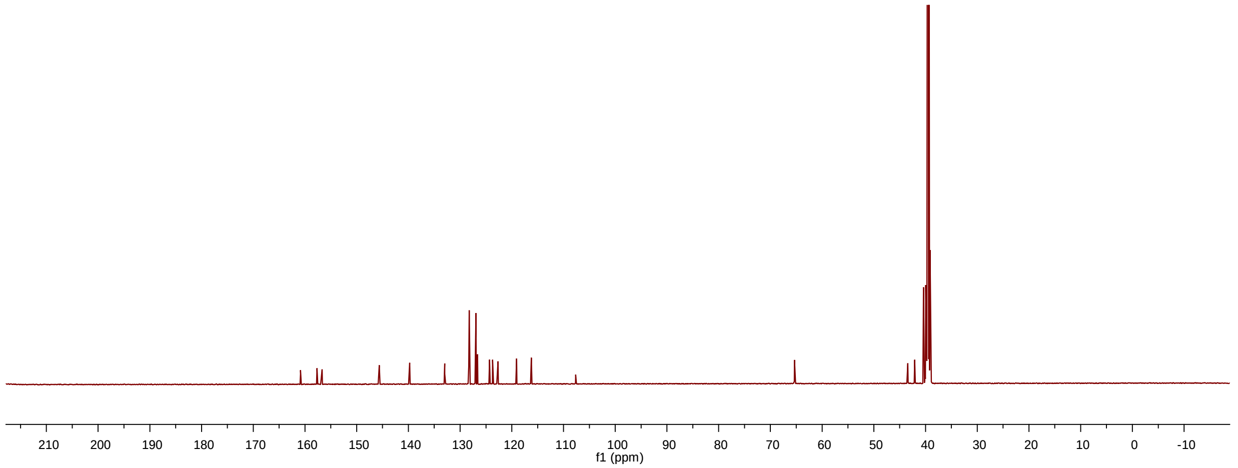

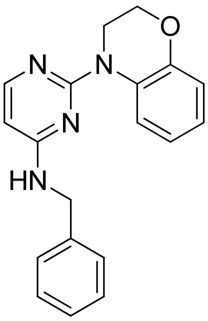

KSC-25-28

2-(*2H*-Benzo[*b*][1,4]oxazin-4(*3H*)-yl)-*N*-benzylpyrimidin-4-amine (S165 or 32). Yield: 20.0 mg, 69%. ^{1}H NMR (400 MHz, $CDCl_3$) δ 8.08 – 7.96 (m, 2H), 7.45 – 7.30 (m, 5H), 7.01 – 6.89 (m, 2H), 6.89 – 6.78 (m, 1H), 5.91 (d, *J* = 5.8 Hz, 1H), 5.13 (s, 1H), 4.57 (d, *J* = 5.0 Hz, 2H), 4.35 – 4.27 (m, 2H), 4.27 – 4.17 (m, 2H). ^{13}C NMR (101 MHz, $CDCl_3$) δ 162.6, 159.7, 156.2, 146.3, 138.5, 128.7, 127.8, 127.5, 127.4, 124.4, 123.6, 119.6, 116.8, 66.0, 45.3, 42.1. HRMS (m/z): calcd for $C_{19}H_{19}N_4O$ (M+H) 319.1559; found 319.1555.

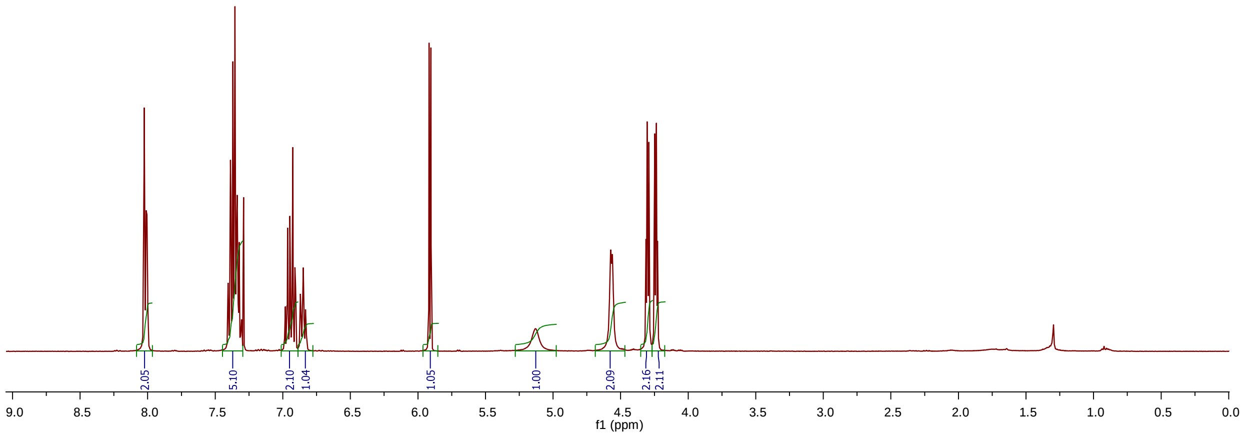

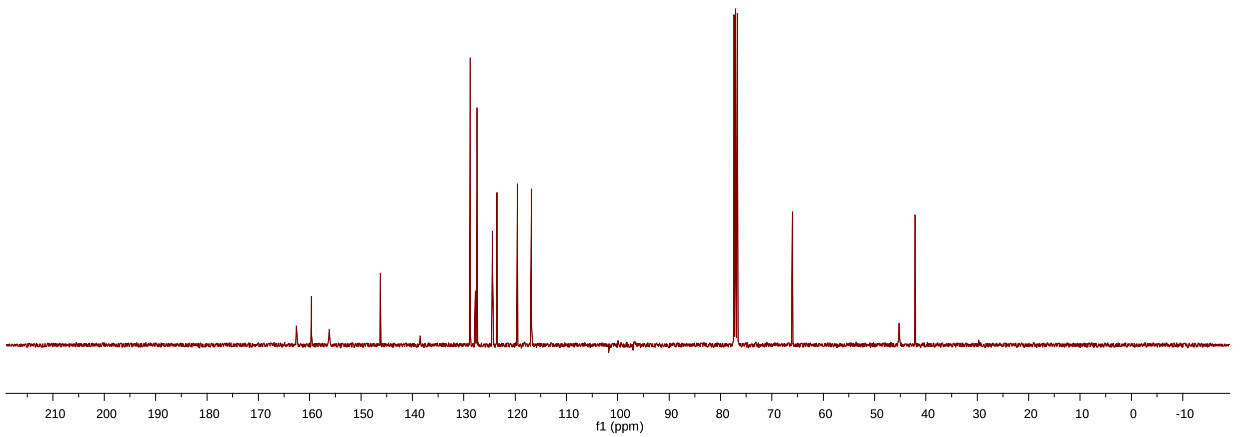

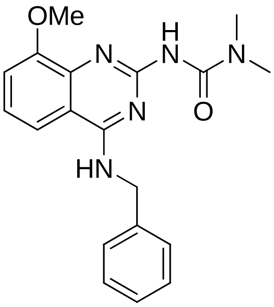

KSC-25-19

3-(4-(Benzylamino)-8-methoxyquinazolin-2-yl)-1,1-dimethylurea (S169). Yield: 12.3 mg, 70%. ^{1}H NMR (400 MHz, $CDCl_3$) δ 7.46 – 7.41 (m, 2H), 7.41 – 7.35 (m, 2H), 7.33 (dt, *J* = 2.2, 5.6 Hz, 1H), 7.10 (dd, *J* = 2.2, 7.2 Hz, 1H), 7.01 – 6.93 (m, 2H), 5.78 (s, 1H), 4.84 (d, *J* = 5.4 Hz, 2H), 3.99 (s, 3H), 3.29 (s, 6H). ^{13}C NMR (101 MHz, $CDCl_3$) δ 159.5, 159.3, 153.2, 144.3, 139.0, 128.7, 128.0, 127.4, 119.9, 112.5, 110.9, 110.2, 56.0, 45.2, 37.0. HRMS not detected because the product was not stable under LC conditions.

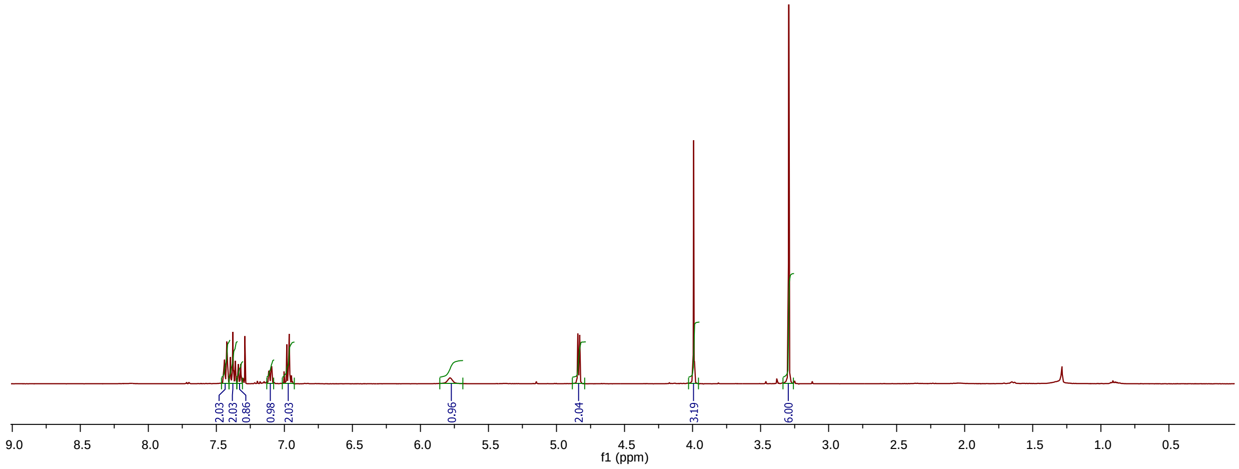

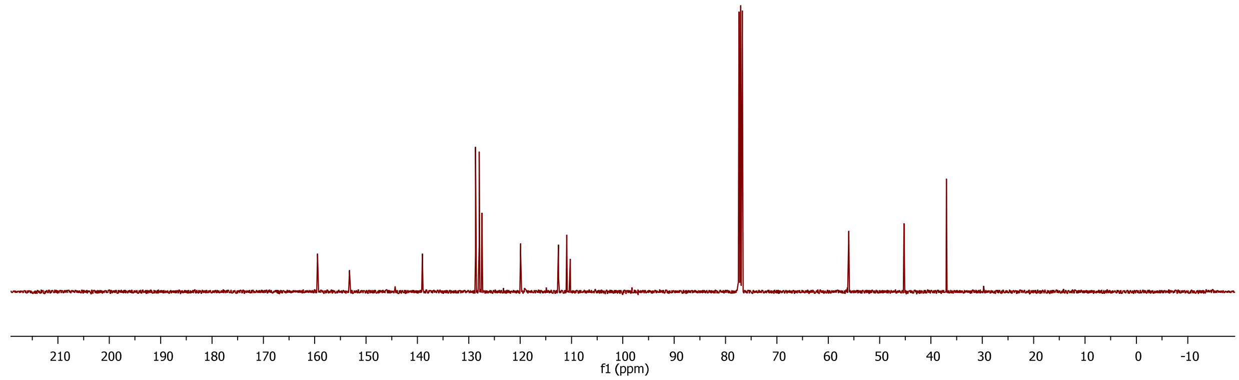

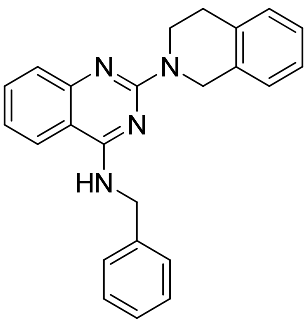

KSC-16-187

***N*-Benzyl-2-(3,4-dihydroisoquinolin-2(*1H*)-yl)quinazolin-4-amine (S170 or 20)**. Yield: 8.0 mg, 59%. ^{1}H NMR (400 MHz, $CDCl_3$) δ 7.45 (d, *J* = 3.6 Hz, 2H), 7.41 (d, *J* = 8.2 Hz, 1H), 7.35 (d, *J* = 7.3 Hz, 2H), 7.33 – 7.21 (m, 3H), 7.16 – 7.03 (m, 4H), 7.03 – 6.89 (m, 1H), 5.73 (s, br. 1H), 4.95 (s, 2H), 4.78 (d, *J* = 5.4 Hz, 2H), 4.07 (t, *J* = 5.9 Hz, 2H), 2.84 (t, *J* = 5.9 Hz, 2H). ^{13}C NMR (101 MHz, $CDCl_3$) δ 159.6, 139.0, 135.5, 134.9, 132.6, 128.7, 128.6, 127.9, 127.5, 126.6, 126.03, 125.95, 120.9, 120.7, 110.3, 46.4, 45.3, 41.5, 29.2. HRMS (m/z): calcd for $C_{24}H_{23}N_4$ (M+H) 367.1923; found 367.1921.

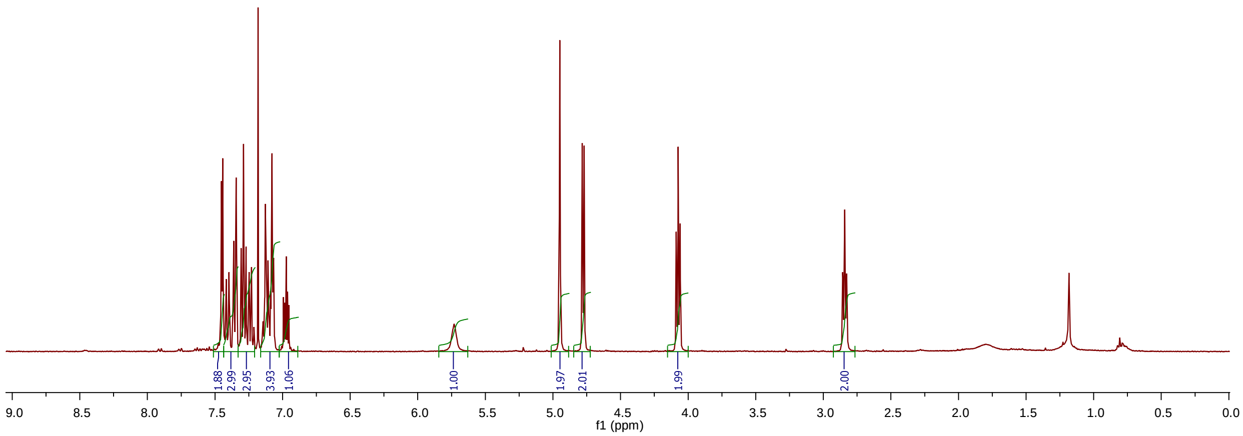

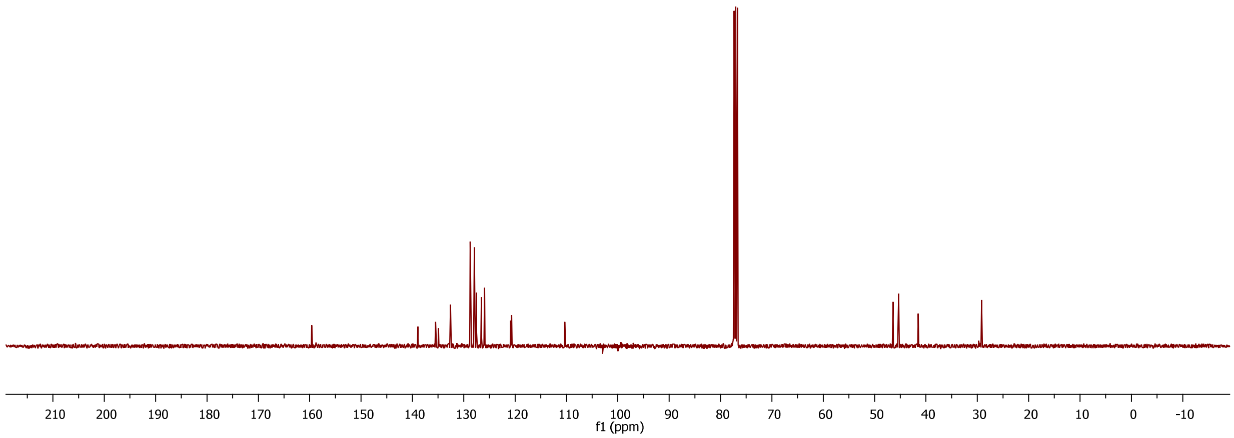

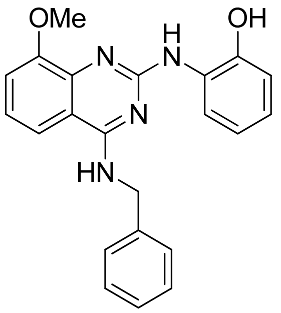

KSC-16-203

2-((4-(Benzylamino)-8-methoxyquinazolin-2-yl)amino)phenol (S171). Yield: 21.3 mg, 86%. ^{1}H NMR (400 MHz, $CDCl_3$) δ 7.47 – 7.31 (m, 5H), 7.16 – 7.10 (m, 3H), 7.10 – 7.06 (m, 1H), 7.03 (td, *J* = 2.0, 6.8 Hz, 1H), 6.94 (d, *J* = 6.6 Hz, 2H), 6.87 – 6.78 (m, 1H), 6.08 (s, br. 1H), 4.83 (d, *J* = 5.4 Hz, 2H), 3.99 (s, 3H). ^{13}C NMR (101 MHz, $CDCl_3$) δ 160.3, 156.5, 153.2, 149.3, 137.9, 128.9, 128.3, 128.0, 127.8, 125.3, 122.5, 121.8, 120.9, 119.6, 112.1, 111.9, 111.2, 56.1, 45.4. HRMS (m/z): calcd for $C_{22}H_{21}N_4O_2$ (M+H) 373.1665; found 373.1657.

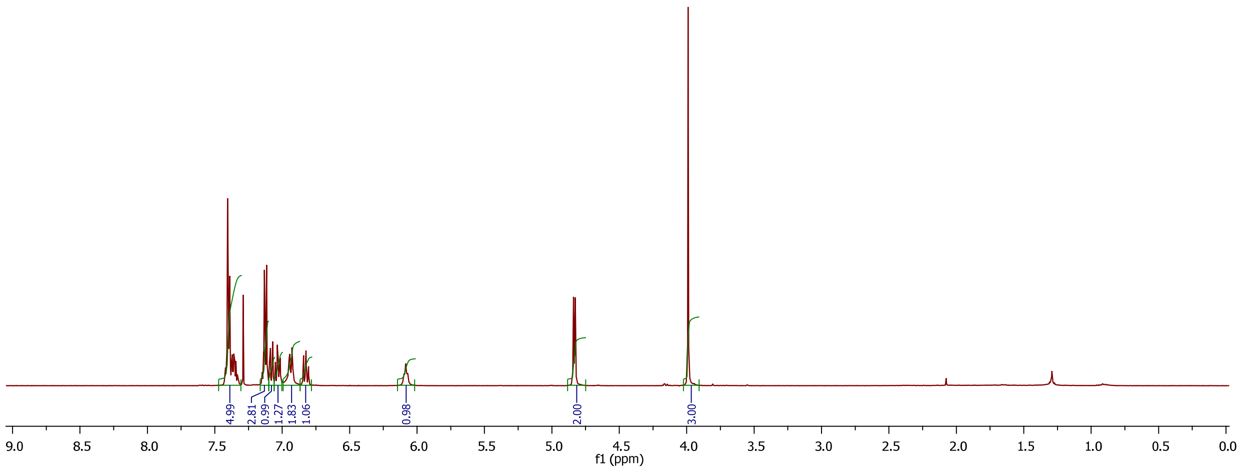

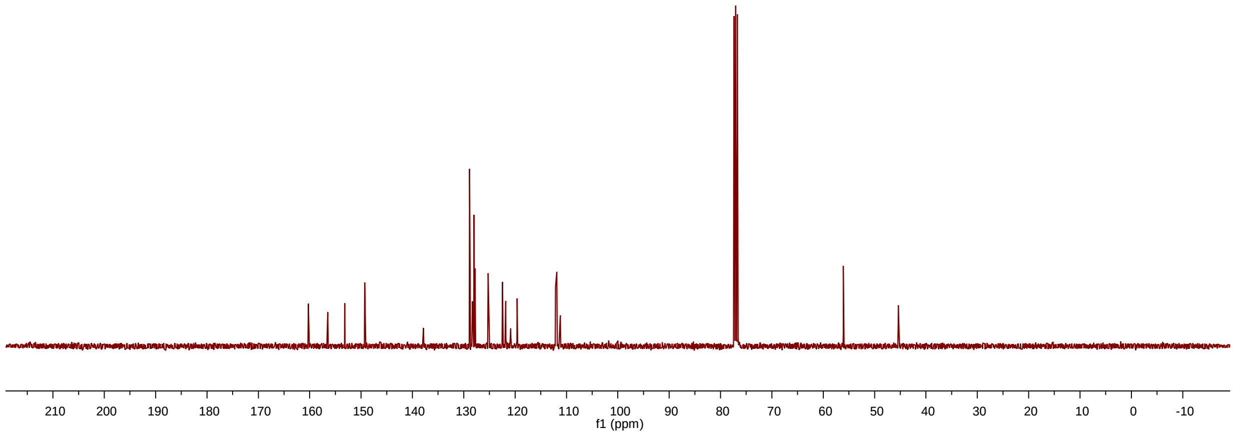

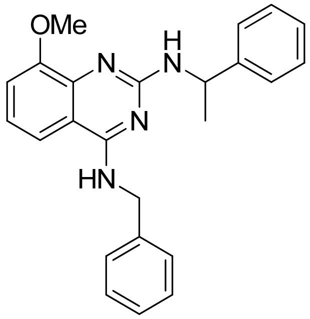

KSC-16-273

***N*[4]-Benzyl-8-methoxy-*N*[2]-(1-phenylethyl)quinazoline-2,4-diamine (S172)**. Yield: 20.5 mg, 80%. ^{1}H NMR (400 MHz, $CDCl_3$) δ 7.39 (d, *J* = 7.2 Hz, 2H), 7.36 – 7.24 (m, 7H), 7.23 – 7.16 (m, 1H), 7.04 (dd, *J* = 2.2, 7.4 Hz, 1H), 6.96 (td, *J* = 4.3, 7.5 Hz, 2H), 5.77 (s, 1H), 5.52 (s, 1H), 5.33 – 5.16 (m, 1H), 4.69 (dd, *J* = 9.4, 11.0 Hz, 2H), 3.99 (s, 3H), 1.53 (d, *J* = 6.9 Hz, 3H). ^{13}C NMR (101 MHz, CDCl3) δ 160.0, 158.7, 153.1, 145.9, 144.3, 138.8, 128.7, 128.3, 128.0, 127.4, 126.5, 126.0, 120.2, 112.5, 110.9, 110.7, 55.9, 51.1, 45.1, 23.5. HRMS (m/z): calcd for $C_{24}H_{25}N_4O$ (M+H) 385.2028; found 385.2018.

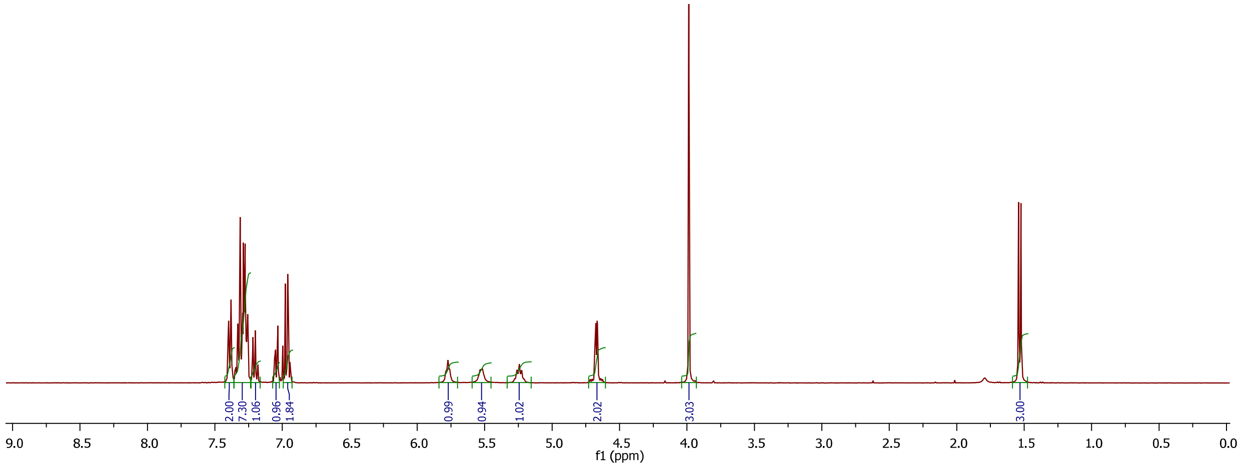

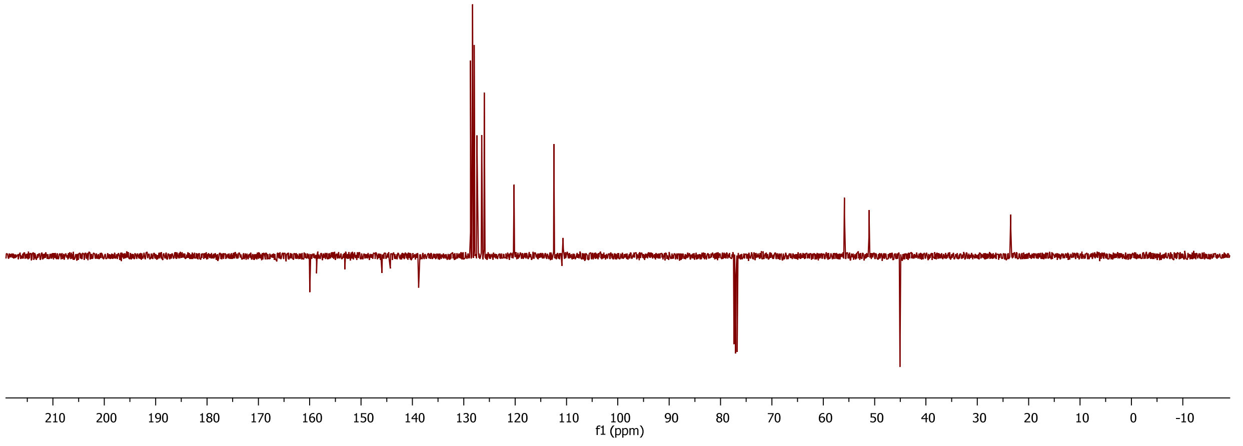

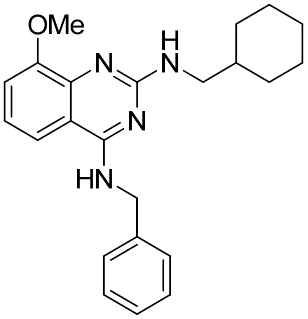

KSC-16-278

***N*[4]-Benzyl-*N*[2]-(cyclohexylmethyl)-8-methoxyquinazoline-2,4-diamine (S173)**. Yield: 19.5 mg, 78%. ^{1}H NMR (400 MHz, DMSO) δ 8.58 – 7.91 (m, 1H), 7.50 (d, *J* = 7.2 Hz, 1H), 7.28 (d, *J* = 7.1 Hz, 2H), 7.22 (t, *J* = 7.5 Hz, 2H), 7.14 (t, *J* = 7.2 Hz, 1H), 6.91 (d, *J* = 7.5 Hz, 1H), 6.85 (t, *J* = 7.9 Hz, 1H), 6.79 – 6.12 (m, 1H), 4.63 (d, *J* = 5.7 Hz, 2H), 3.73 (s, 3H), 3.02 (s, 2H), 1.53 (s, br. 5H), 1.42 – 1.21 (m, 1H), 1.00 (s, br., 3H), 0.72 (s, br., 2H). ^{13}C NMR (101 MHz, DMSO) δ 159.8, 159.1, 152.6, 144.1, 140.2, 128.1, 127.1, 126.5, 119.0, 114.3, 111.0, 55.2, 47.0, 43.5, 37.8, 30.5, 26.2, 25.5. HRMS (m/z): calcd for $C_{23}H_{29}N_4O$ (M+H) 377.2341; found 377.2338.

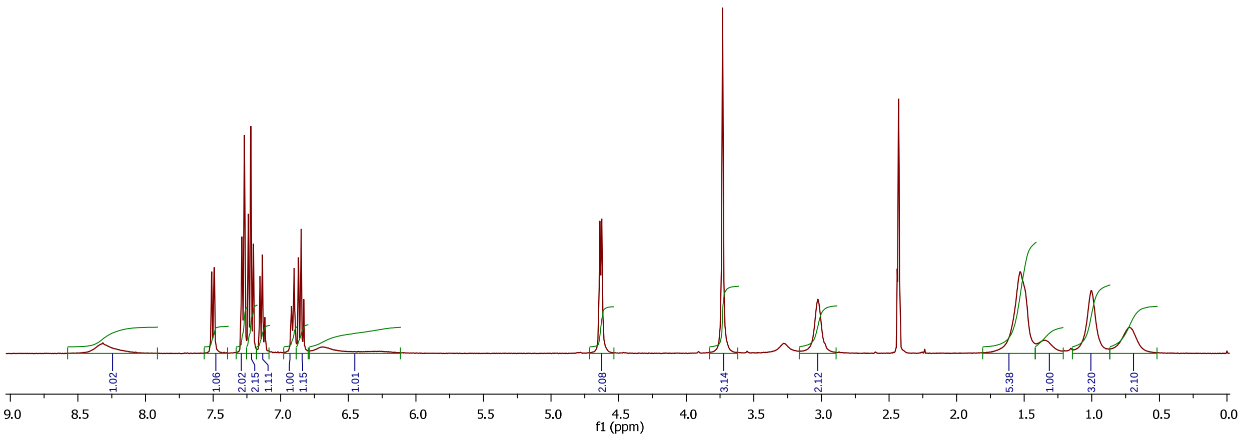

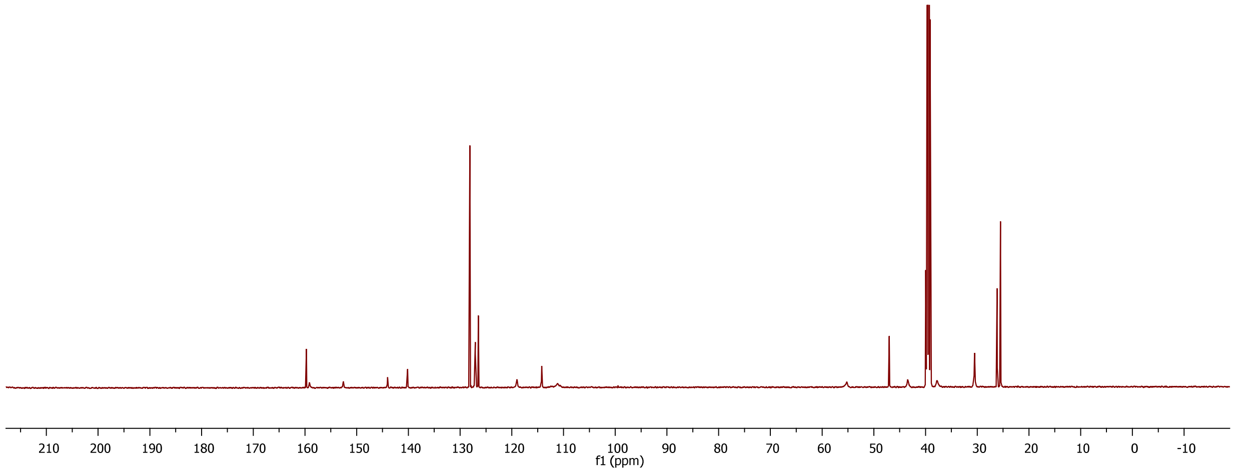

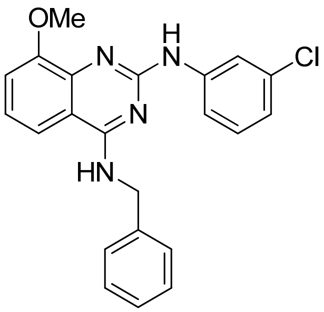

KSC-16-193

***N*[4]-Benzyl-*N*[2]-(3-chlorophenyl)-8-methoxyquinazoline-2,4-diamine (S174 or 50)**. Yield: 9.6 mg, 74%. ^{1}H NMR (400 MHz, $CDCl_3$) δ 8.03 (t, *J* = 2.1 Hz, 1H), 7.51 – 7.33 (m, 6H), 7.28 (d, *J* = 11.5 Hz, 1H), 7.23 – 7.12 (m, 3H), 7.07 (dd, *J* = 1.9, 7.1 Hz, 1H), 6.96 (ddd, *J* = 0.9, 2.0, 7.9 Hz, 1H), 5.94 (s, br. 1H), 4.90 (d, *J* = 5.1 Hz, 2H), 4.05 (s, 3H). ^{13}C NMR (101 MHz, $CDCl_3$) δ 160.2, 156.3, 153.7, 141.7, 138.0, 134.4, 129.7, 128.9, 127.9, 127.8, 122.1, 121.4, 118.8, 116.7, 112.4, 111.8, 111.3, 56.1, 45.7. HRMS (m/z): calcd for $C_{22}H_{20}ClN_4O$ (M+H) 391.1326; found 391.1325.

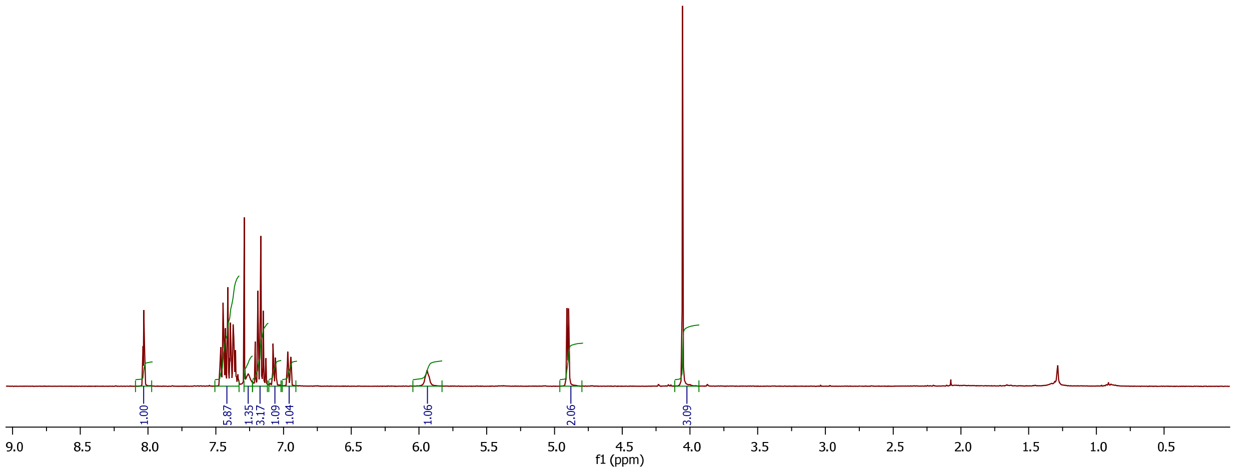

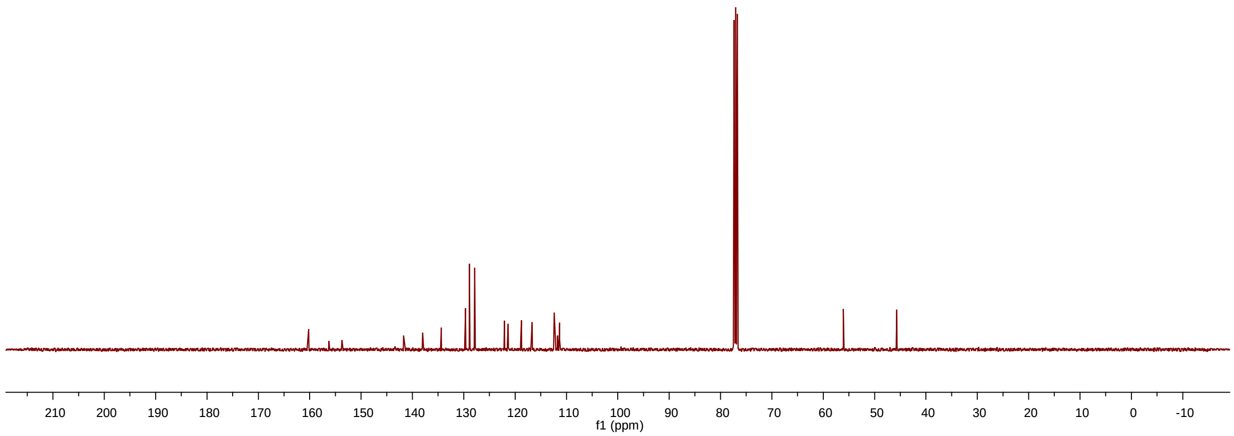

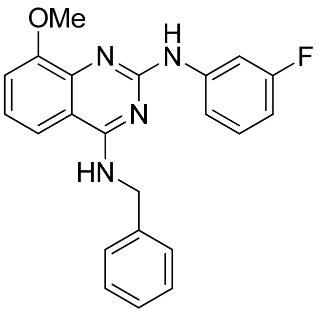

KSC-16-194

***N*[4]-Benzyl-*N*[2]-(3-fluorophenyl)-8-methoxyquinazoline-2,4-diamine (S175 or 51)**. Yield: 12.2 mg, 98%. ^{1}H NMR (400 MHz, $CDCl_3$) δ 7.73 (dt, *J* = 2.1, 11.8 Hz, 1H), 7.42 – 7.20 (m, 6H), 7.15 – 7.00 (m, 4H), 6.96 (dd, *J* = 1.7, 7.3 Hz, 1H), 6.64 – 6.51 (m, 1H), 5.86 (s, 1H), 4.79 (d, *J* = 5.1 Hz, 2H), 3.95 (s, 3H). ^{13}C NMR (101 MHz, CDCl3) δ 164.5, 162.1, 160.2, 156.3, 153.7, 143.3, 142.2, 142.1, 138.0, 129.7, 129.6, 128.9, 127.9, 127.8, 122.1, 114.0, 114.0, 112.4, 111.8, 111.3, 108.1, 107.8, 106.1, 105.8, 56.1, 45.7. HRMS (m/z): calcd for $C_{22}H_{20}FN_4O$ (M+H) 375.1621; found 375.1626.

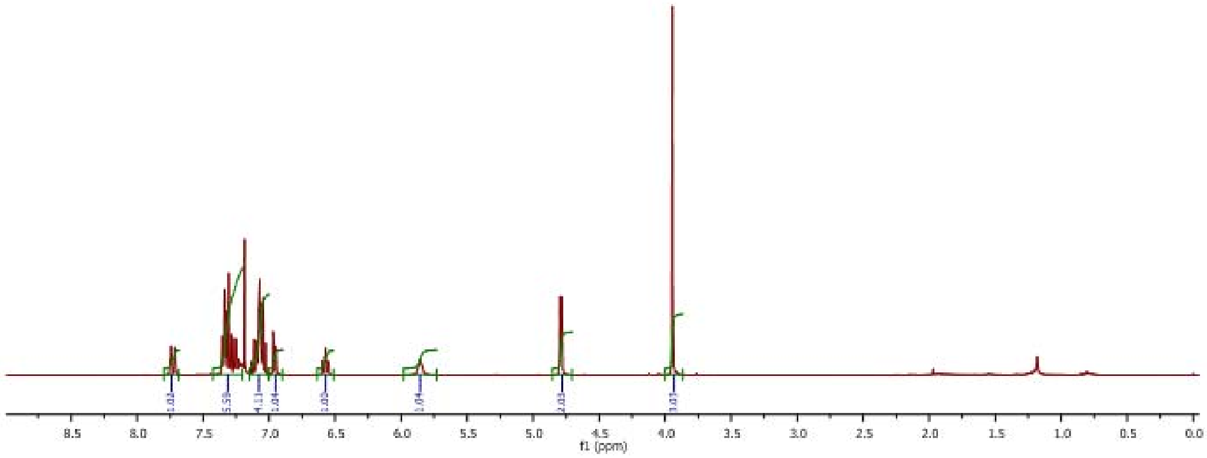

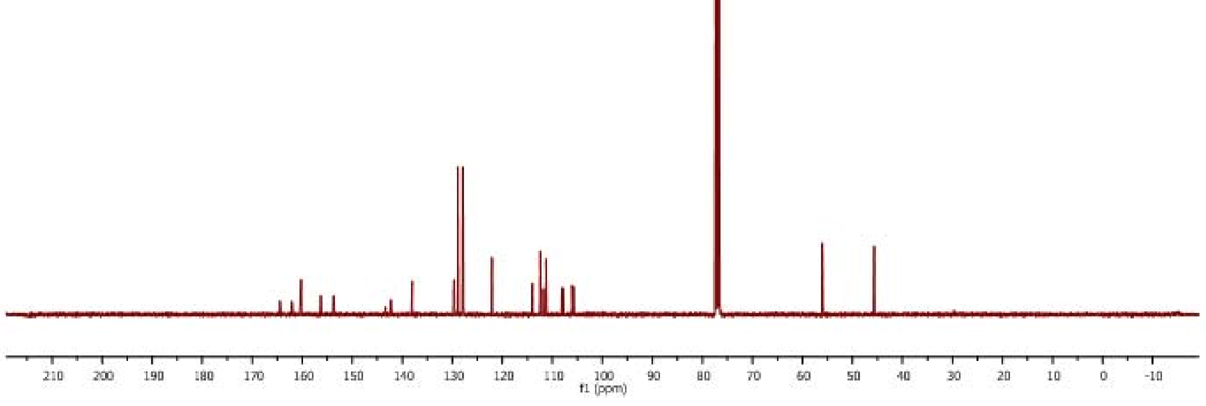

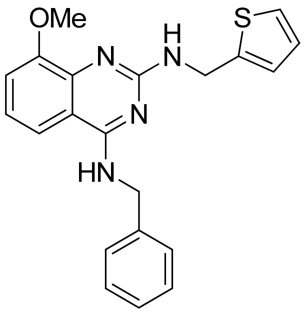

KSC-16-282

***N*⁴-Benzyl-8-methoxy-*N*²-(thiophen-2-ylmethyl)quinazoline-2,4-diamine (S176 or 52)**. Yield: 17.9 mg, 71%. ^{1}H NMR (400 MHz, DMSO) δ 8.33 (s, 1H), 7.53 (dd, *J* = 1.3, 8.0 Hz, 1H), 7.27 (d, *J* = 7.2 Hz, 2H), 7.24 – 7.16 (m, 3H), 7.13 (dd, *J* = 6.0, 8.3 Hz, 1H), 6.98 – 6.83 (m, 3H), 6.83 – 6.72 (m, 1H), 4.66 (d, *J* = 5.8 Hz, 2H), 4.57 (d, *J* = 5.7 Hz, 2H), 3.74 (s, 3H). ^{13}C NMR (101 MHz, DMSO) δ 159.9, 158.4, 152.8, 144.6, 143.8, 140.0, 128.2, 127.3, 126.6, 126.4, 124.7, 124.2, 119.7, 114.2, 111.7, 111.3, 55.4, 45.1, 43.4. HRMS (m/z): calcd for $C_{21}H_{21}N_4OS$ (M+H) 377.1436; found 377.1433.

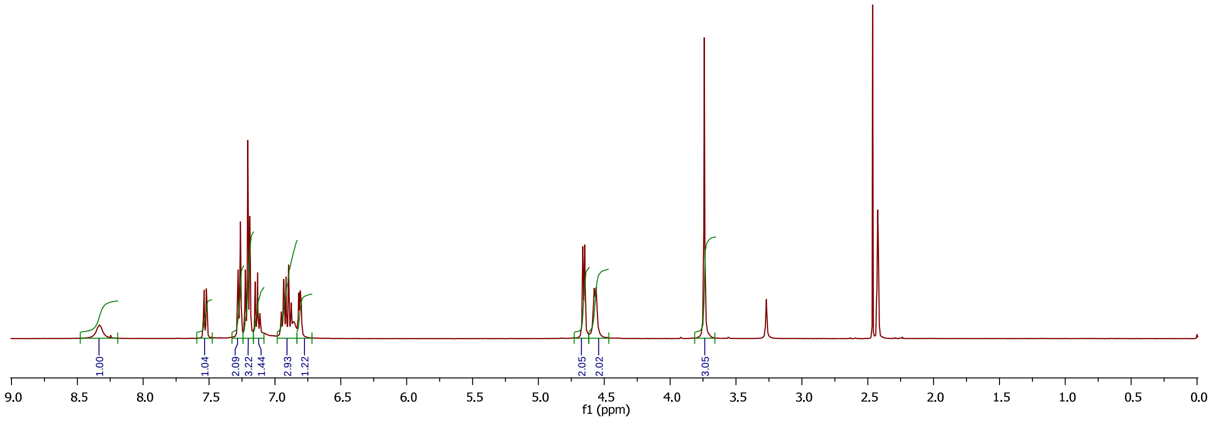

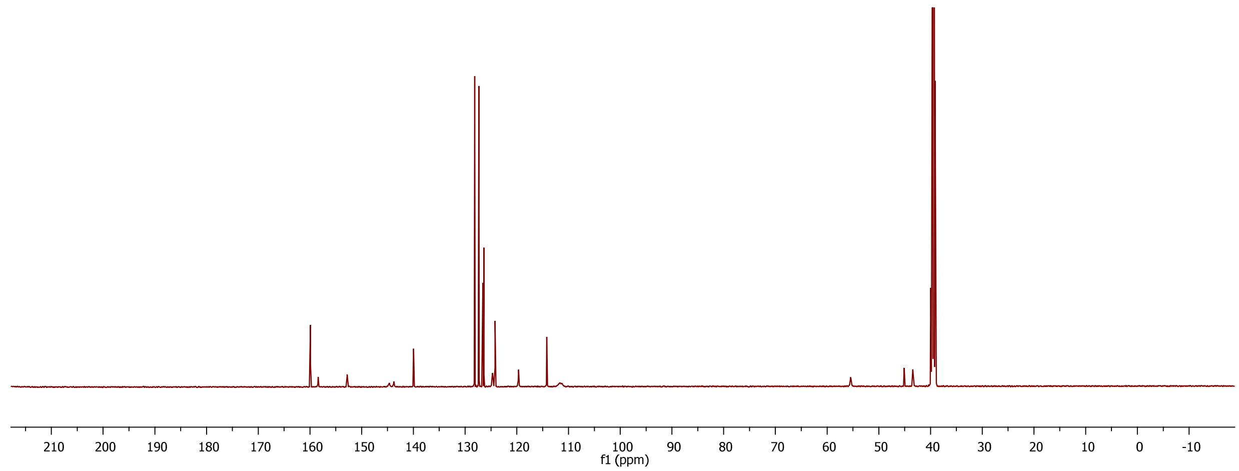

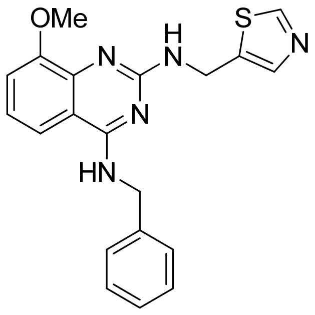

KSC-16-283

***N*^{4}-Benzyl-8-methoxy-*N*^{2}-(thiazol-5-ylmethyl)quinazoline-2,4-diamine (S177)**. Yield: 18.7 mg, 74%. ^{1}H NMR (400 MHz, DMSO) δ 8.86 (s, 1H), 8.45 (s, 1H), 7.75 (s, 1H), 7.63 (d, *J* = 7.9 Hz, 1H), 7.43 – 7.11 (m, 6H), 7.02 (d, *J* = 13.8 Hz, 2H), 4.83 – 4.55 (m, 4H), 3.84 (s, 3H). ^{13}C NMR (101 MHz, DMSO) δ 160.0, 158.2, 153.3, 152.9, 143.6, 140.8, 139.9, 128.2, 127.2, 126.6, 119.9, 114.2, 111.9, 111.4, 55.5, 43.4, 36.6. HRMS (m/z): calcd for $C_{20}H_{20}N_5OS$ (M+H) 378.1389; found 378.1384.

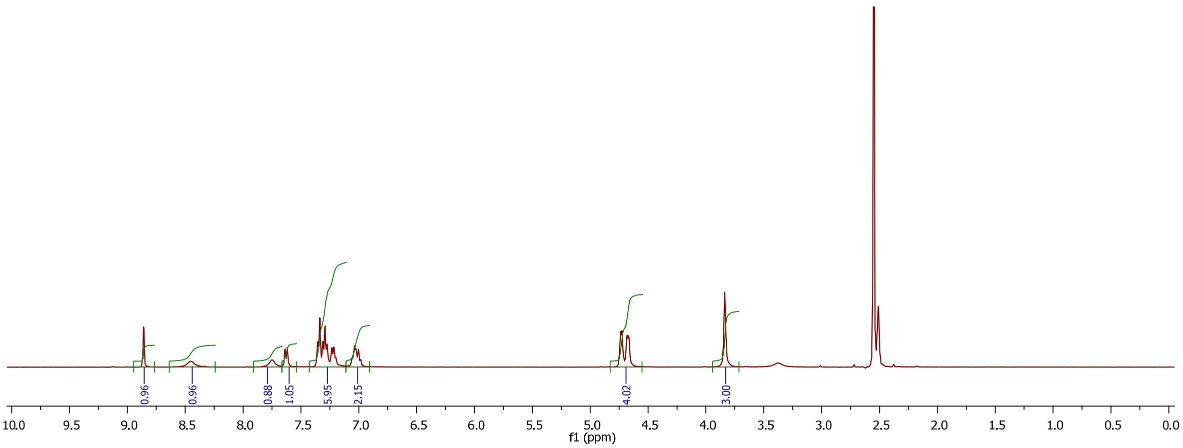

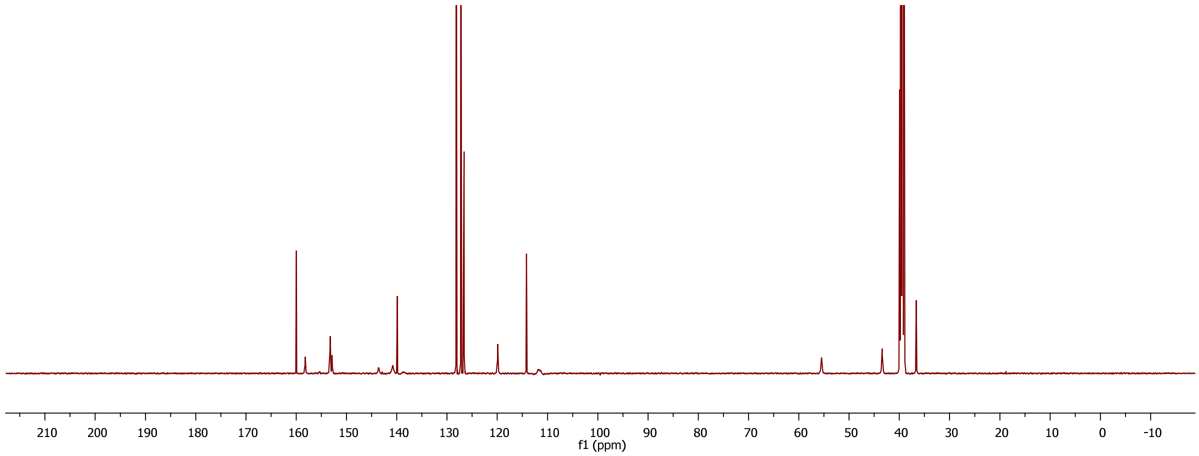

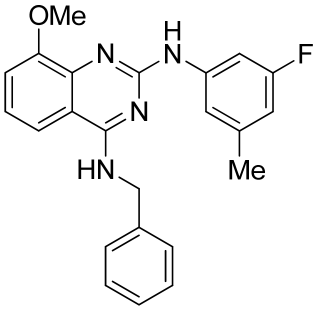

KSC-16-196

***N*[4]-Benzyl-*N*[2]-(3-fluoro-5-methylphenyl)-8-methoxyquinazoline-2,4-diamine (S178)**. Yield: 11.1 mg, 86%. ^{1}H NMR (400 MHz, $CDCl_3$) δ 7.53 (d, *J* = 11.7 Hz, 1H), 7.41 – 7.22 (m, 5H), 7.12 – 7.00 (m, 2H), 7.00 – 6.89 (m, 2H), 6.41 (d, *J* = 9.3 Hz, 1H), 5.84 (s, 1H), 4.80 (d, *J* = 5.1 Hz, 2H), 3.95 (s, 3H), 2.20 (s, 3H). ^{13}C NMR (101 MHz, CDCl3) δ 164.4, 162.0, 160.2, 156.4, 153.7, 141.8, 141.7, 140.1, 140.0, 138.0, 128.9, 127.9, 127.8, 122.0, 114.6, 112.4, 111.7, 111.3, 109.0, 108.7, 103.3, 103.0, 56.1, 45.7, 21.58, 21.56. HRMS (m/z): calcd for $C_{23}H_{22}FN_4O$ (M+H) 389.1778; found 389.1781.

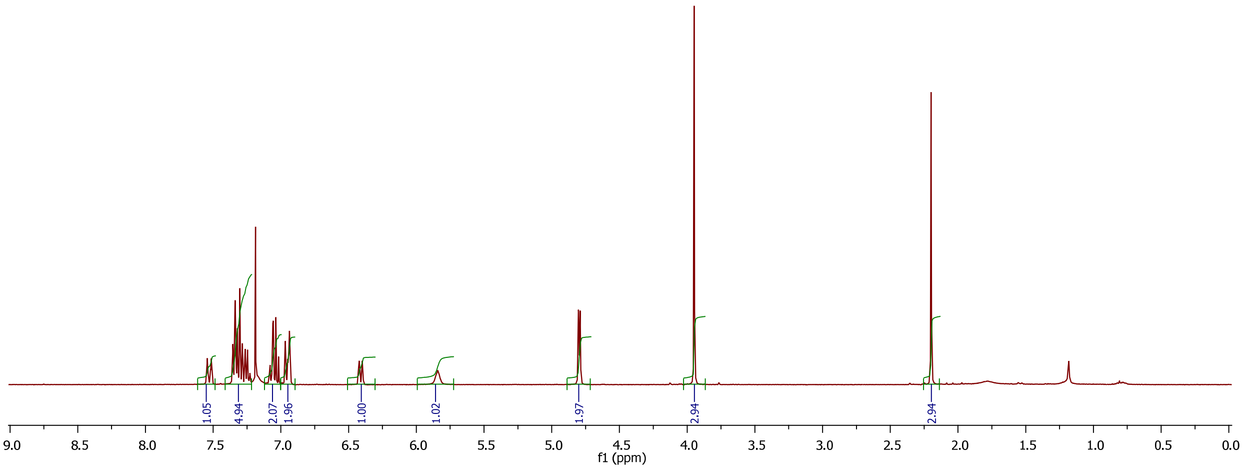

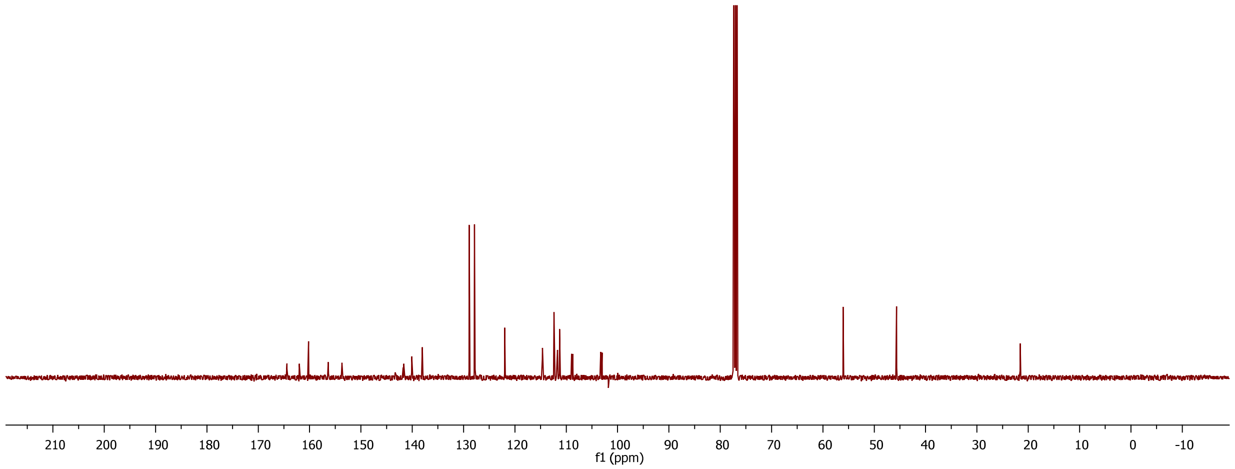

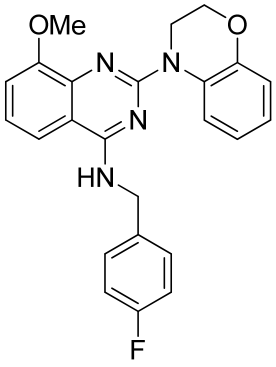

KSC-16-261

2-(*2H*-Benzo[*b*][1,4]oxazin-4(*3H*)-yl)-*N*-(4-fluorobenzyl)-8-methoxyquinazolin-4-amine (S179 or 40). Yield: 2.9 mg, 22%. ^{1}H NMR (400 MHz, $CDCl_3$) δ 8.12 – 7.87 (m, 1H), 7.25 (s, br. 2H), 7.14 – 7.02 (m, 2H), 6.98 – 6.93 (m, 2H), 6.84 (s, br. 2H), 6.78 – 6.56 (m, 1H), 5.89 – 5.66 (m, 1H), 4.69 (s, br. 2H), 4.32 (s, br. 2H), 4.24 (s, br. 2H), 3.92 (s, 3H). ^{13}C NMR (126 MHz, $CDCl_3$) δ 163.2, 161.2, 159.7, 134.2, 129.5, 124.4, 123.3, 122.0, 119.4, 116.7, 115.6, 115.5, 112.3, 111.7, 111.4, 66.2, 56.2, 44.6, 42.2, 29.7. HRMS (m/z): calcd for $C_{24}H_{22}FN_4O_2$ (M+H) 417.1727; found 417.1726.

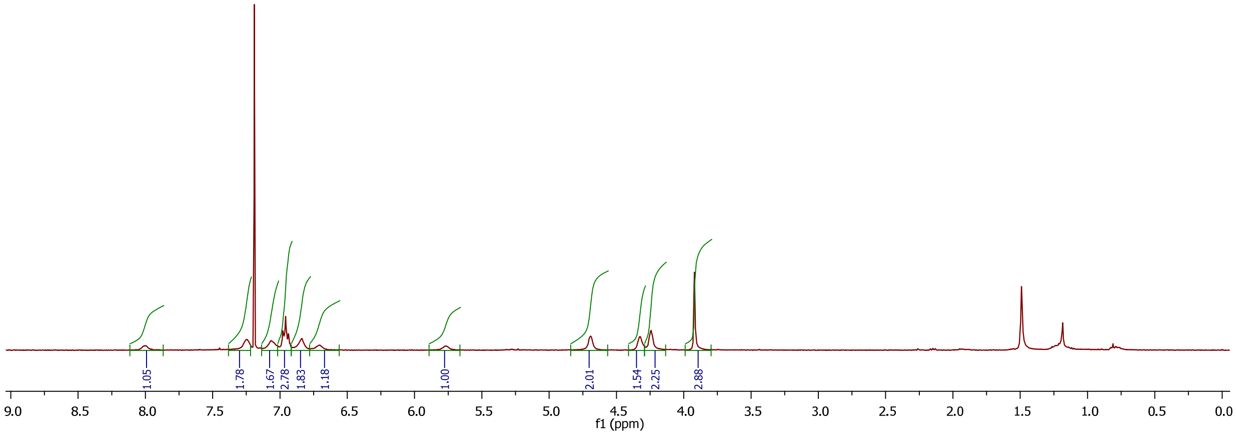

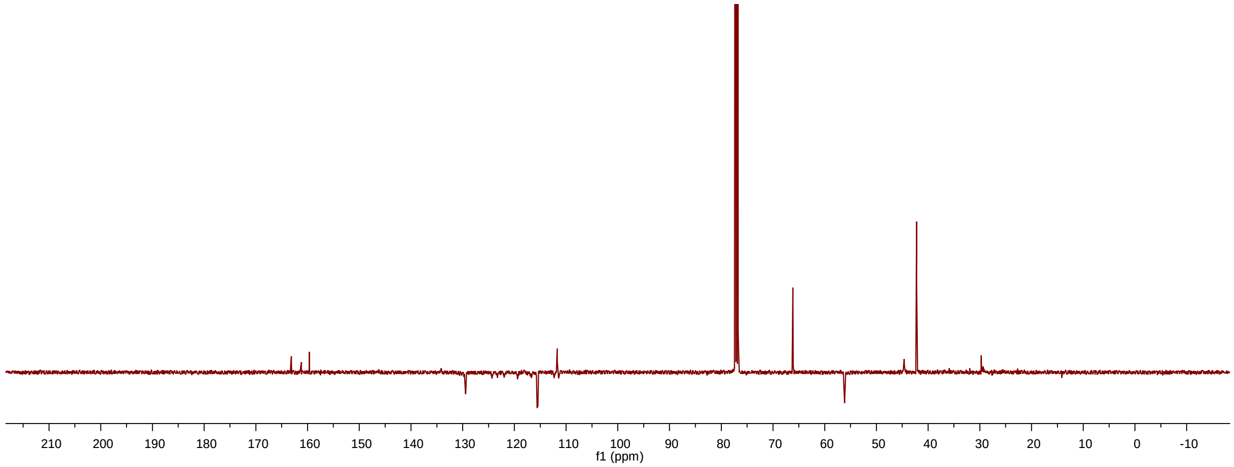

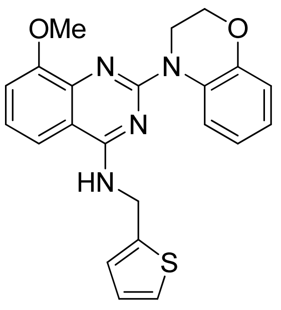

KSC-16-295

2-(*2H*-Benzo[*b*][1,4]oxazin-4(*3H*)-yl)-8-methoxy-*N*-(thiophen-2-ylmethyl)quinazolin-4-amine (S180 or 41). Yield: 15.9 mg, 80%. ^{1}H NMR (400 MHz, $CDCl_3$) δ 8.25 (dd, *J* = 1.4, 8.2 Hz, 1H), 7.27 (dd, *J* = 1.3, 5.1 Hz, 1H), 7.19 – 7.09 (m, 2H), 7.08 – 7.02 (m, 2H), 6.99 (ddd, *J* = 2.5, 4.1, 8.1 Hz, 1H), 6.95 (dd, *J* = 1.8, 4.0 Hz, 1H), 6.93 – 6.86 (m, 1H), 5.90 (s, 1H), 4.98 (d, *J* = 5.1 Hz, 2H), 4.47 (dd, *J* = 3.6, 5.3 Hz, 2H), 4.37 (dd, *J* = 3.6, 5.3 Hz, 2H), 4.02 (s, 3H). ^{13}C NMR (101 MHz, $CDCl_3$) δ 159.3, 156.1, 153.9, 146.2, 143.6, 141.2, 128.1, 126.9, 126.2, 125.4, 124.4, 123.3, 122.0, 119.5, 116.7, 112.4, 111.7, 111.5, 66.2, 56.1, 42.3, 40.2. HRMS (m/z): calcd for $C_{22}H_{21}N_4O_2S$ (M+H) 405.1385; found 405.1383.

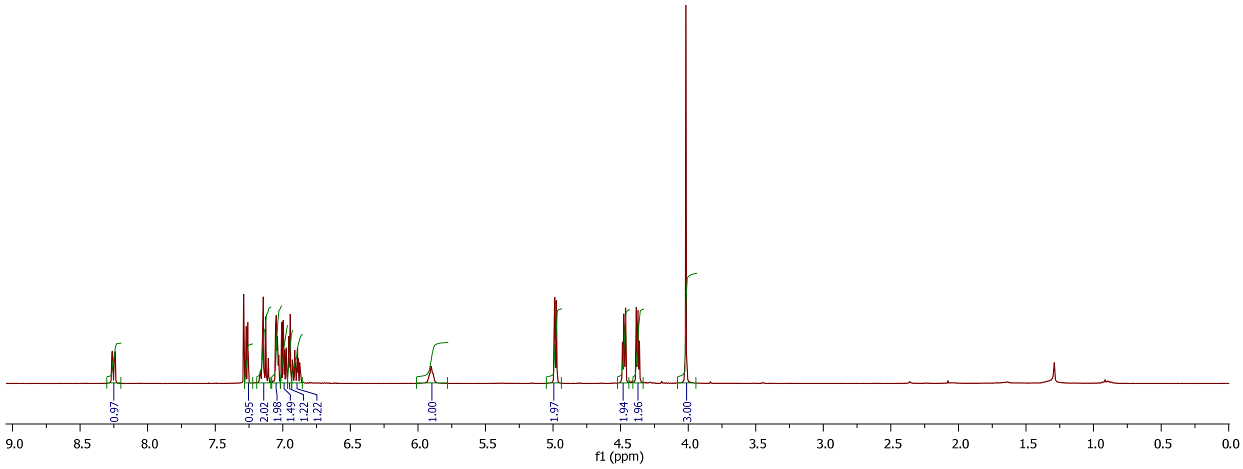

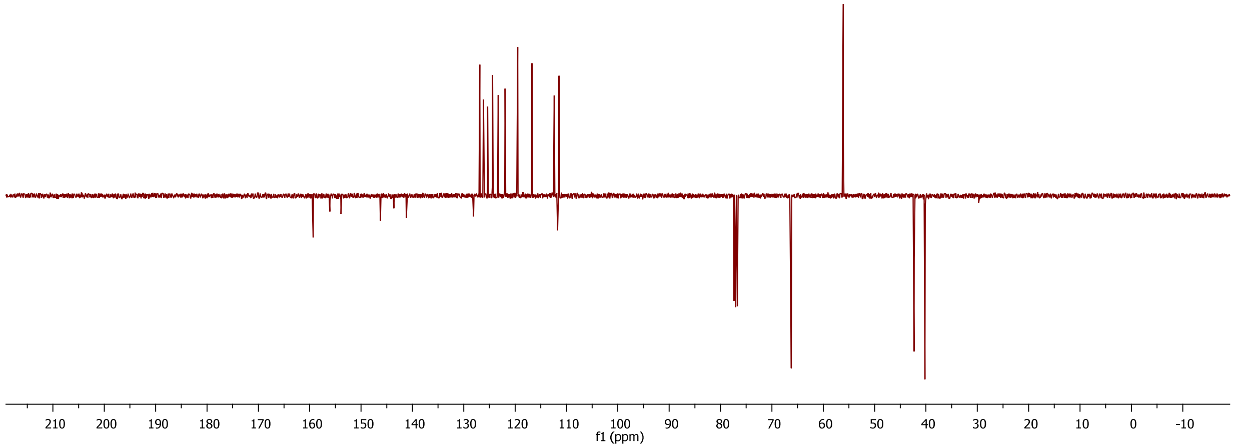

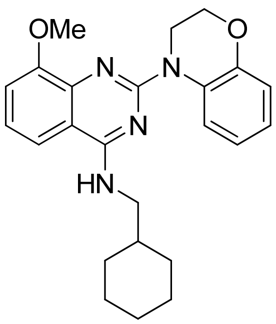

KSC-16-299

2-(*2H*-Benzo[*b*][1,4]oxazin-4(*3H*)-yl)-*N*-(cyclohexylmethyl)-8-methoxyquinazolin-4-amine (S181 or 42). Yield: 13.6 mg, 69%. ^{1}H NMR (400 MHz, $CDCl_3$) δ 8.26 (dd, *J* = 1.4, 8.1 Hz, 1H), 7.20 – 7.09 (m, 2H), 7.03 (dd, *J* = 1.9, 7.1 Hz, 1H), 7.01 – 6.86 (m, 3H), 5.69 (s, 1H), 4.46 (dd, *J* = 3.6, 5.2 Hz, 2H), 4.37 (dd, *J* = 3.6, 5.2 Hz, 2H), 4.02 (s, 3H), 3.54 – 3.42 (m, 2H), 187 – 1.71 (m, 6H), 1.40 – 1.13 (m, 4H), 1.04 (q, *J* = 12.0 Hz, 2H). ^{13}C NMR (101 MHz, $CDCl_3$) δ 160.0, 156.4, 153.9, 146.2, 143.4, 128.2, 124.4, 123.1, 121.7, 119.3, 116.7, 112.2, 111.9, 111.2, 66.2, 56.1, 47.6, 42.2, 37.8, 31.1, 26.5, 25.9. HRMS (m/z): calcd for $C_{24}H_{29}N_4O_2$ (M+H) 405.2291; found 405.2289.

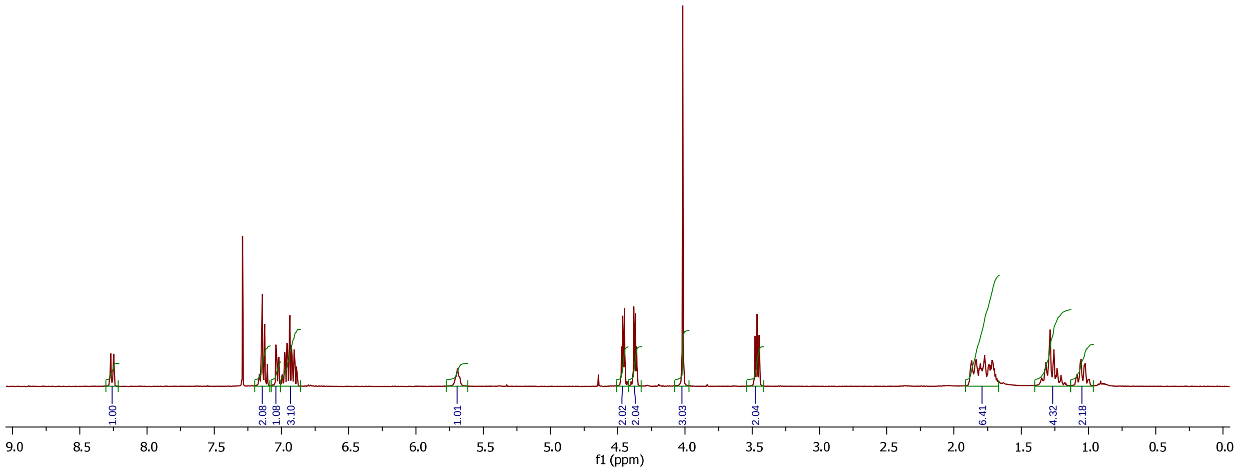

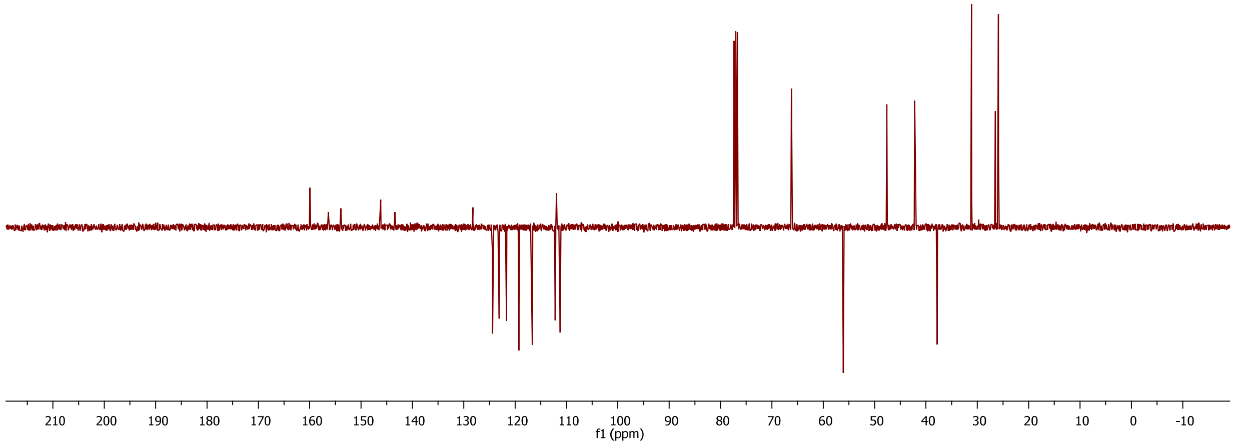

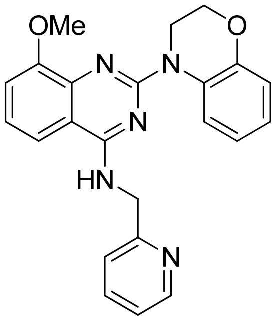

KSC-16-219

2-(*2H*-Benzo[*b*][1,4]oxazin-4(*3H*)-yl)-8-methoxy-*N*-(pyridin-2-ylmethyl)quinazolin-4-amine (S182). Yield: 7.4 mg, 56%. ^{1}H NMR (400 MHz, $CDCl_3$) δ 8.53 (s, 1H), 8.10 (s, 1H), 7.59 (td, *J* = 1.7, 7.7 Hz, 1H), 7.32 (s, br. 1H), 7.22 (d, *J* = 7.8 Hz, 1H), 7.18 – 7.12 (m, 1H), 7.09 (t, *J* = 8.0 Hz, 1H), 6.96 (d, *J* = 7.1 Hz, 1H), 6.93 – 6.77 (m, 3H), 4.79 (d, *J* = 4.6 Hz, 2H), 4.39 – 4.32 (m, 2H), 4.28 – 4.22 (m, 2H), 3.92 (s, 3H). ^{13}C NMR (101 MHz, CDCl3) δ 159.7, 156.6, 149.0, 146.3, 136.7, 124.2, 123.3, 122.4, 122.2, 119.4, 116.8, 112.2, 122.0, 111.5, 66.2, 56.1, 45.9, 42.3. HRMS (m/z): calcd for $C_{23}H_{22}N_5O_2$ (M+H) 400.1773; found 400.1768.

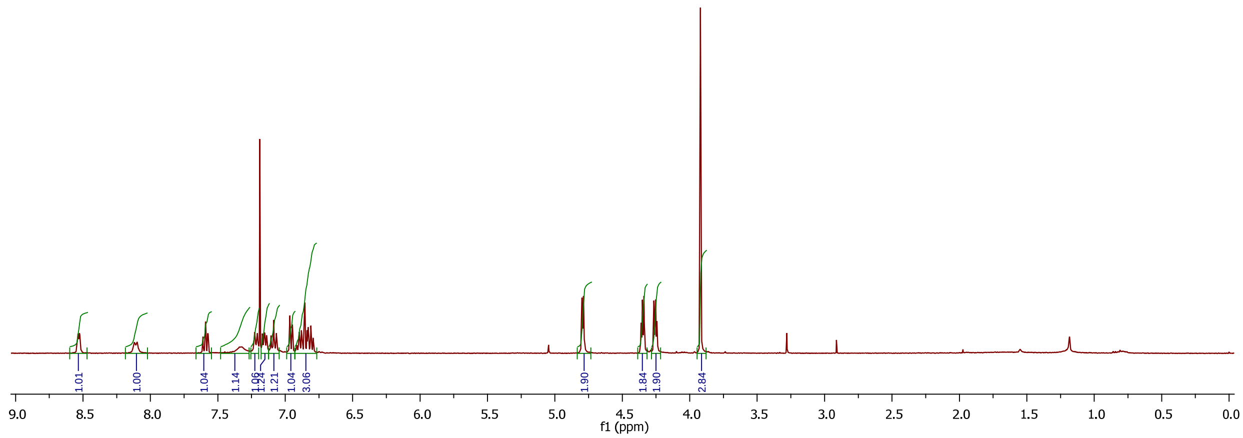

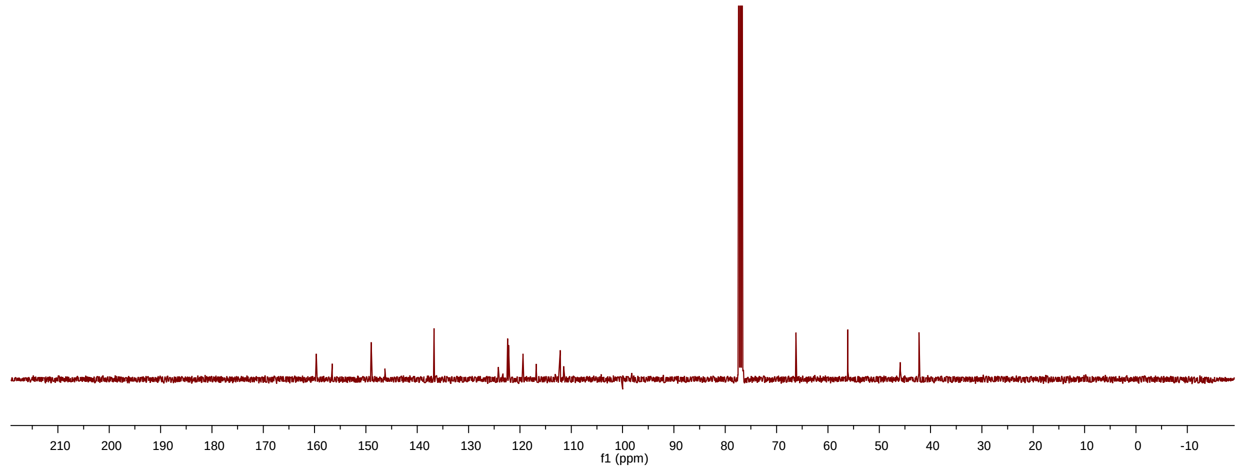

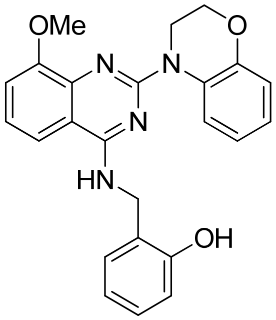

KSC-16-222

2-(((2-(*2H*-Benzo[*b*][1,4]oxazin-4(*3H*)-yl)-8-methoxyquinazolin-4-yl)amino)methyl)phenol (S183 or 43). Yield: 11.0 mg, 51%. ^{1}H NMR (400 MHz, CDCl3) δ 8.91 (s, br. 1H), 7.97 (s, br. 1H), 7.14 – 7.04 (m, 3H), 7.01 (t, *J* = 7.9 Hz, 1H), 6.93 (d, *J* = 8.8 Hz, 1H), 6.91 – 6.85 (m, 2H), 6.82 (t, *J* = 7.3 Hz, 1H), 6.73 (t, *J* = 7.2 Hz, 2H), 4.68 (s, br. 2H), 4.30 (s, 4H), 3.87 (s, 3H). ^{13}C NMR (101 MHz, $CDCl_3$) δ 159.9, 155.8, 146.3, 130.8, 129.9, 124.0, 122.8, 119.9, 119.7, 117.4, 117.2, 112.6, 112.1, 111.7, 66.0, 56.1, 43.1, 42.0. HRMS (m/z): calcd for $C_{24}H_{23}N_4O_3$ (M+H) 415.1770; found 415.1763.

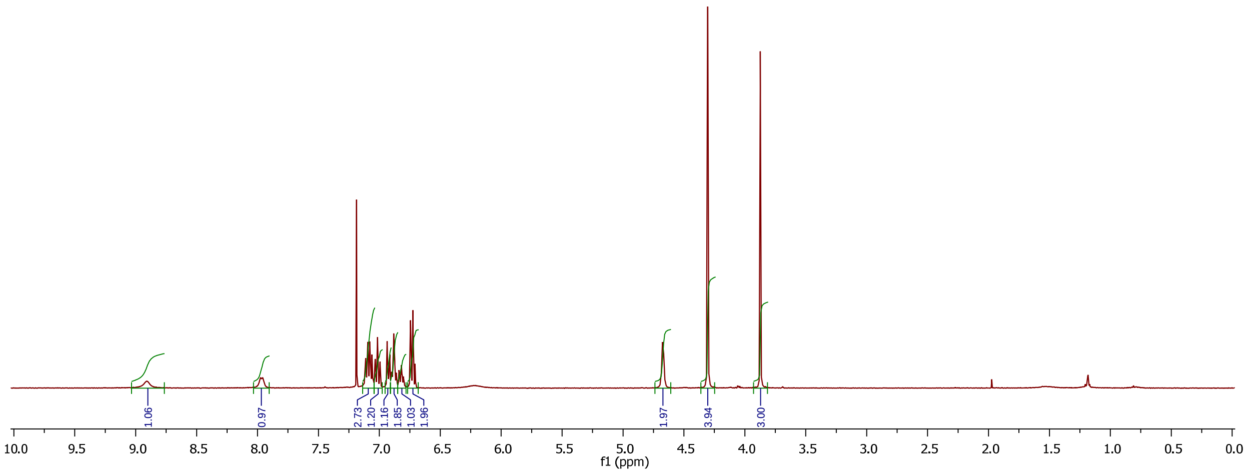

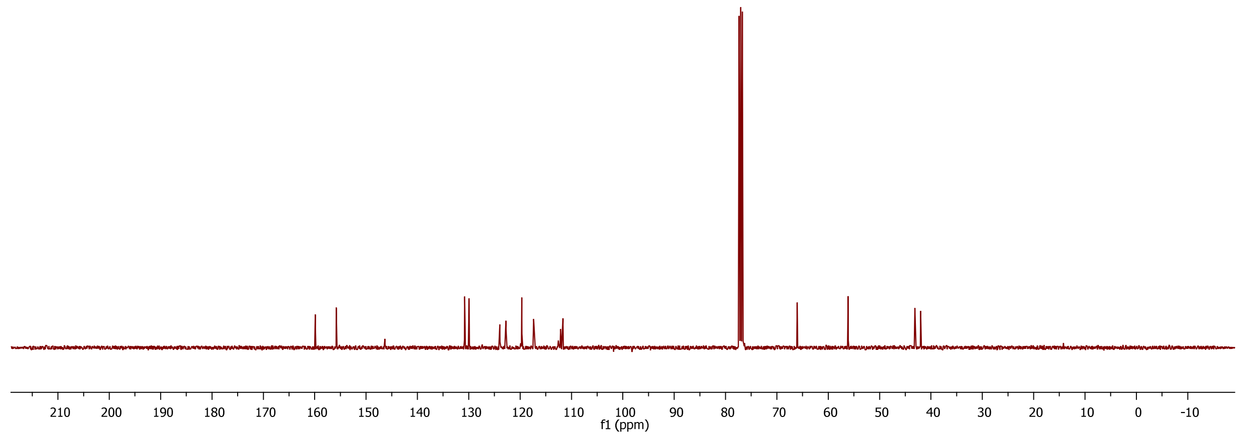

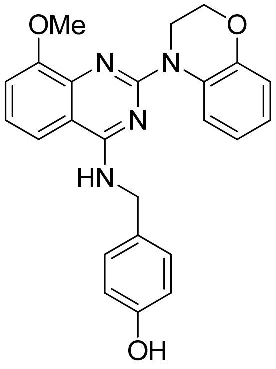

KSC-16-235

4-(((2-(*2H*-Benzo[*b*][1,4]oxazin-4(*3H*)-yl)-8-methoxyquinazolin-4-yl)amino)methyl)phenol (S184). Yield: 7.0 mg, 36%. ^{1}H NMR (400 MHz, $CDCl_3$) δ 8.01 (s, 1H), 7.13 – 7.11 (m, 3H), 7.03 (t, *J* = 8.0 Hz, 1H), 6.94 (d, *J* = 8.6 Hz, 1H), 6.91 – 6.78 (m, 2H), 6.72 (d, *J* = 8.4 Hz, 3H), 4.62 (d, *J* = 5.0 Hz, 2H), 4.31 (d, *J* = 4.5 Hz, 2H), 4.23 (d, *J* = 4.6 Hz, 2H), 3.90 (s, 3H). ^{13}C NMR (101 MHz, $CDCl_3$) δ 159.6, 155.3, 146.3, 130.2, 129.3, 124.2, 123.5, 122.1, 119.5, 116.8, 115.6, 112.7, 111.8, 111.6, 66.2, 56.1, 44.9, 42.3. HRMS (m/z): calcd for $C_{24}H_{23}N_4O_3$ (M+H) 415.1770; found 415.1760.

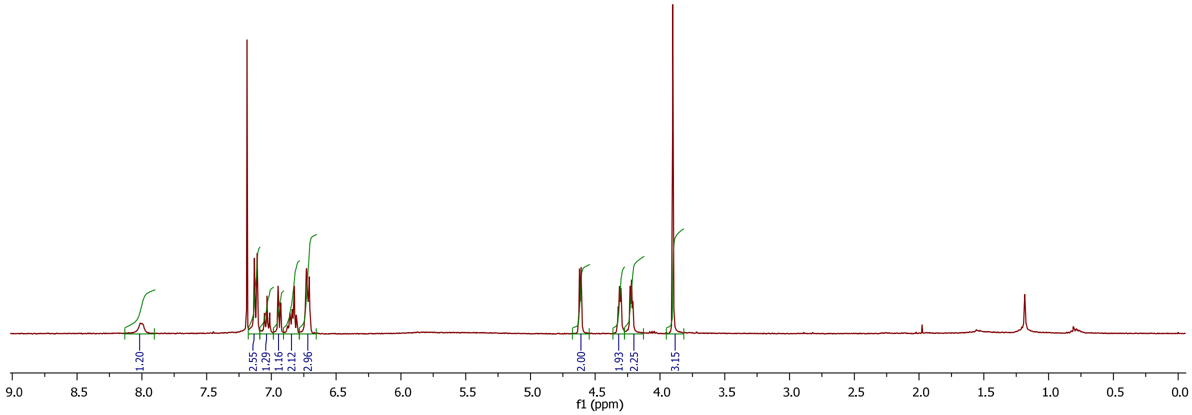

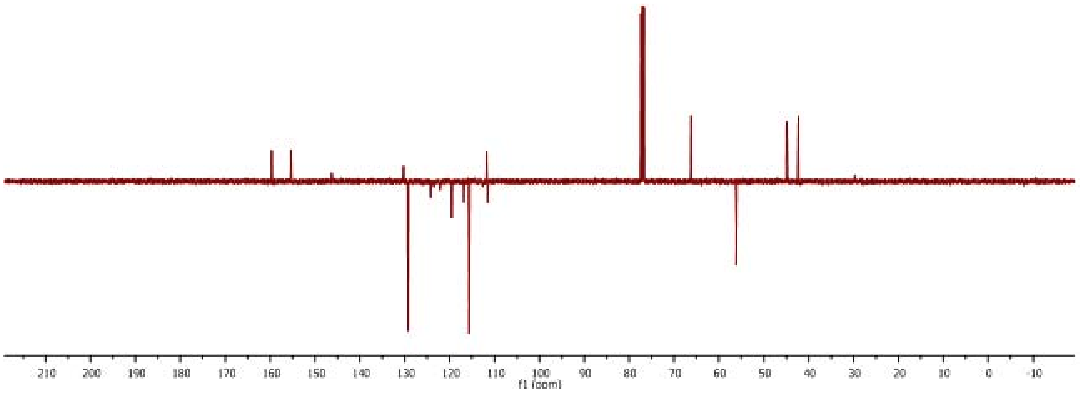

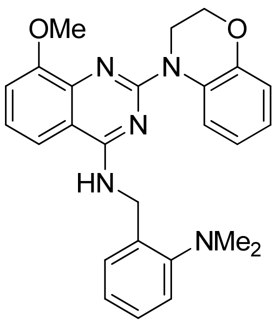

KSC-16-227

2-(*2H*-Benzo[*b*][1,4]oxazin-4(*3H*)-yl)-*N*-(2-(dimethylamino)benzyl)-8-methoxyquinazolin-4-amine (S185). Yield: 4.6 mg, 36%. ^{1}H NMR (400 MHz, $CDCl_3$) δ 8.16 (s, 1H), 7.31 (s, br. 1H), 7.21 (d, *J* = 7.2 Hz, 2H), 7.14 (d, *J* = 7.5 Hz, 1H), 7.04 – 6.97 (m, 3H), 6.94 – 6.91 (m, 1H), 6.89 – 6.85 (m, 2H), 6.84 – 6.74 (m, 1H), 4.82 (d, *J* = 5.1 Hz, 2H), 4.40 – 4.36 (m, 2H), 4.29 – 4.27 (m, 2H), 3.92 (s, 3H), 2.71 (s, 6H). ^{13}C NMR (126 MHz, $CDCl_3$) δ 159.8, 132.2, 129.6, 128.4, 124.4, 124.0, 121.8, 120.0, 119.3, 116.7, 112.4, 112.3, 111.2, 66.2, 56.1, 44.7, 43.4, 42.2. HRMS (m/z): calcd for $C_{26}H_{28}N_5O_2$ (M+H) 442.2243; found 442.2233.

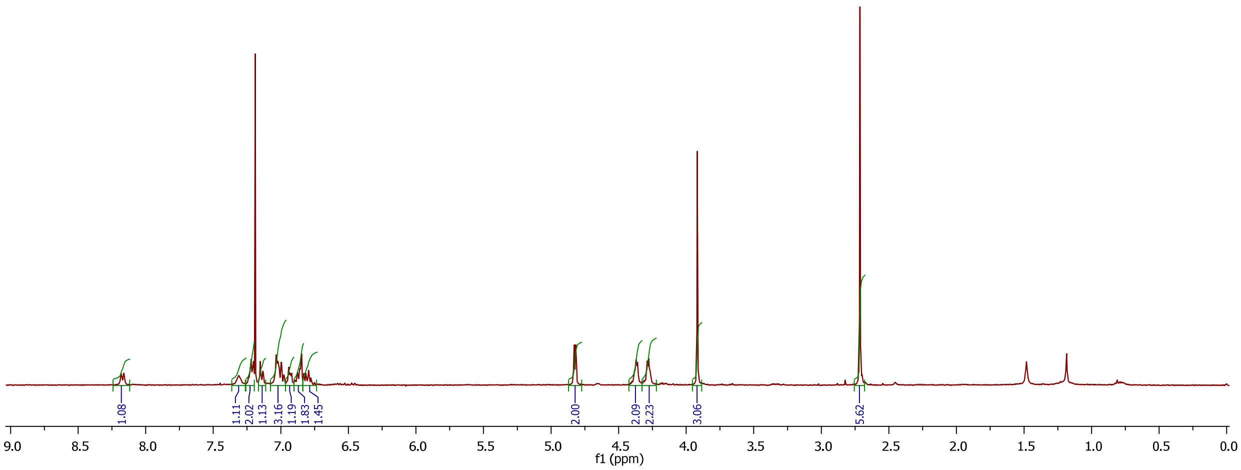

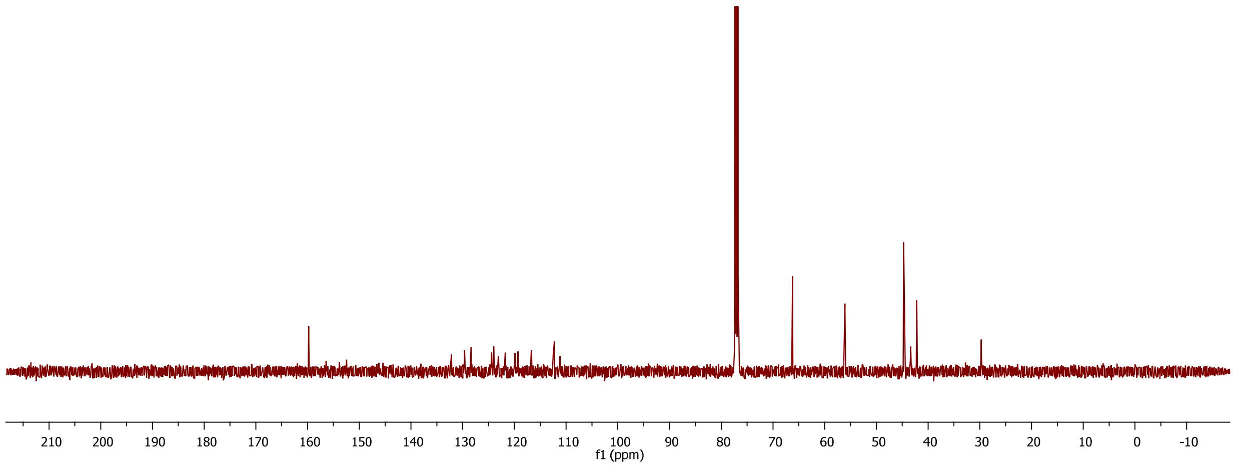

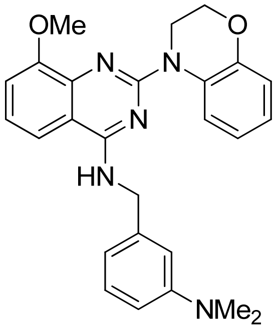

KSC-16-243

2-(*2H*-Benzo[*b*][1,4]oxazin-4(*3H*)-yl)-*N*-(3-(dimethylamino)benzyl)-8-methoxyquinazolin-4-amine (S186). Yield: 5.7 mg, 44%. ^{1}H NMR (400 MHz, $CDCl_3$) δ 8.39 – 8.15 (m, 1H), 7.26 (s, 1H), 7.14 (s, 2H), 7.05 (s, 1H), 6.94 (s, 2H), 6.90 – 6.82 (m, 1H), 6.76 (d, *J* = 7.1 Hz, 2H), 6.71 (d, *J* = 8.0 Hz, 1H), 5.93 – 5.72 (m, 1H), 4.77 (s, br. 2H), 4.46 (s, br. 2H), 4.36 (s, br. 2H), 4.02 (s, 3H), 2.95 (s, 6H). ^{13}C NMR (101 MHz, $CDCl_3$) δ 159.7, 151.0, 139.2, 129.5, 124.4, 123.1, 121.8, 119.5, 116.7, 116.0, 112.4, 112.2, 111.4, 111.8, 111.8, 66.2, 56.1, 46.2, 42.2, 40.6. HRMS (m/z): calcd for $C_{26}H_{28}N_5O_2$ (M+H) 442.2243; found 442.2239.

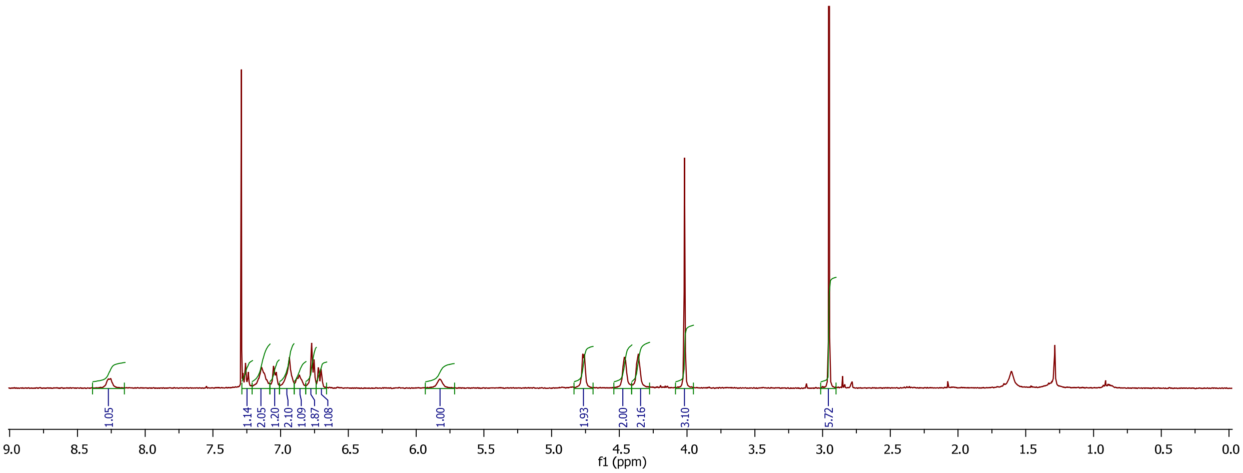

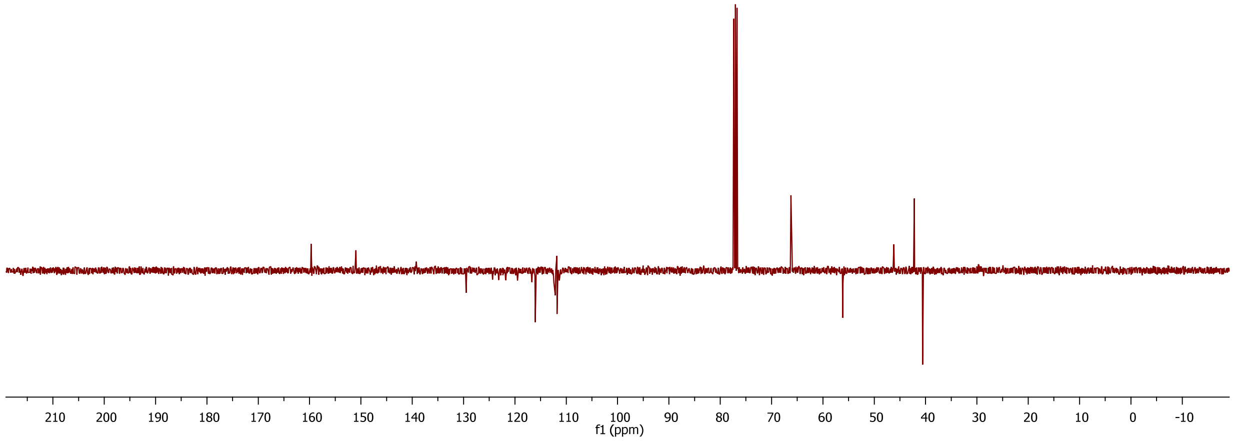

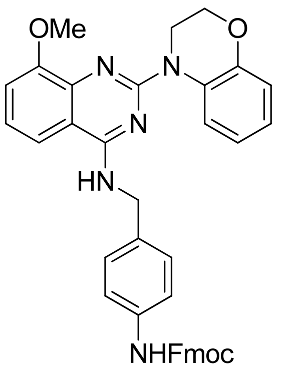

KSC-16-251

(*9H*-Fluoren-9-yl)methyl (4-(((2-(*2H*-benzo[*b*][1,4]oxazin-4(*3H*)-yl)-8-methoxyquinazolin-4-yl)amino)methyl)phenyl)carbamate (S187). Yield: 5.4 mg, 23%. ^{1}H NMR (400 MHz, $CDCl_3$) δ 8.24 – 8.06 (m, 1H), 7.81 (d, *J* = 7.5 Hz, 2H), 7.64 (d, *J* = 7.5 Hz, 2H), 7.44 (t, *J* = 7.4 Hz, 2H), 7.41 – 7.30 (m, 5H), 7.23 – 7.09 (m, 2H), 7.06 (s, 1H), 6.94 (s, 2H), 6.88 – 6.78 (m, 1H), 6.78 – 6.61 (m, 1H), 5.99 – 5.71 (m, 1H), 4.77 (s, br. 2H), 4.58 (d, *J* = 6.5 Hz, 2H), 4.42 (s, br. 2H), 4.34 – 4.28 (m, 3H), 4.02 (s, 3H). ^{13}C NMR (126 MHz, $CDCl_3$) δ 159.7, 153.4, 143.7, 141.4, 137.0, 128.6, 127.8, 127.6, 127.2, 124.9, 120.1, 119.0, 111.8, 66.9, 66.2, 56.2, 47.1, 44.9, 42.2, 29.7. HRMS (m/z): calcd for $C_{39}H_{34}N_5O_4$ (M+H) 636.2611; found 636.2613.

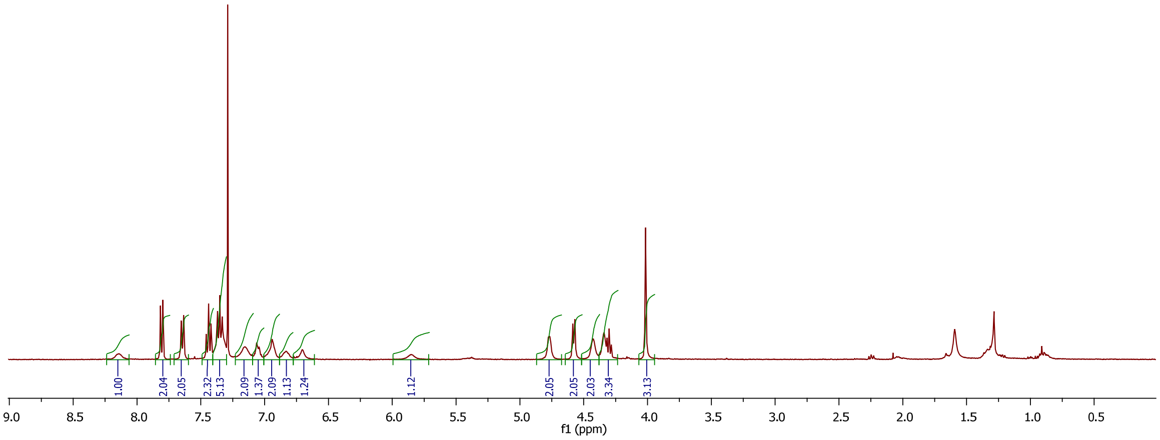

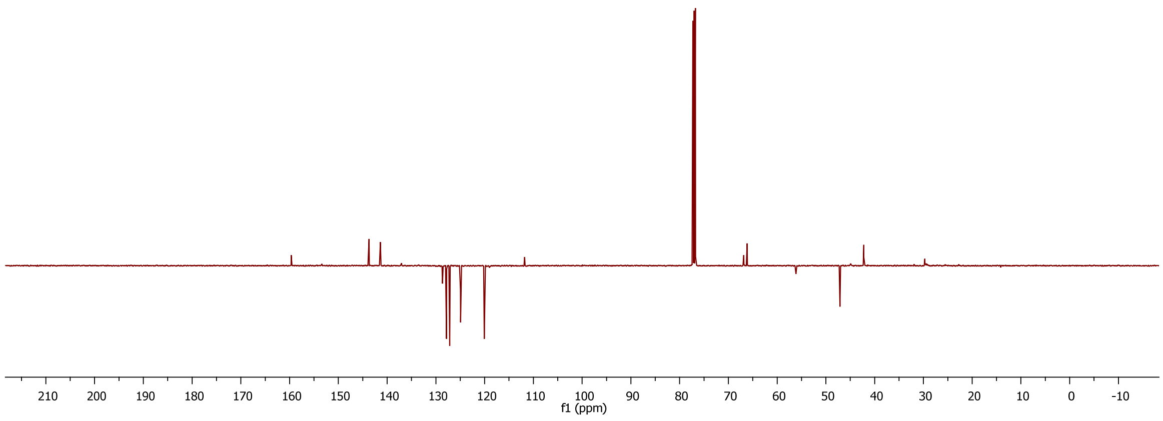

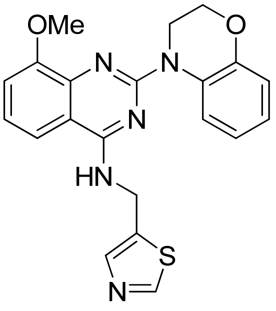

KSC-25-6

2-(*2H*-Benzo[*b*][1,4]oxazin-4(*3H*)-yl)-8-methoxy-*N*-(thiazol-5-ylmethyl)quinazolin-4-amine (S188). Yield: 10.1 mg, 76%. ^{1}H NMR (400 MHz, $CDCl_3$) δ 8.71 (s, 1H), 8.29 – 8.01 (m, 1H), 7.82 (s, 1H), 7.16 (s, 2H), 7.06 (d, *J* = 8.6 Hz, 1H), 6.96 – 6.84 (m, 3H), 6.28 – 5.93 (m, 1H), 5.03 (d, *J* = 5.5 Hz, 2H), 4.53 – 4.42 (m, 2H), 4.42 – 4.29 (m, 2H), 4.01 (s, 3H). ^{13}C NMR (126 MHz, $CDCl_3$) δ 159.3, 156.0, 153.6, 146.4, 143.7, 142.1, 135.7, 127.8, 124.4, 123.6, 122.2, 119.5, 116.9, 113.9, 112.4, 111.7, 66.3, 56.1, 42.4, 37.3. HRMS (m/z): calcd for $C_{21}H_{20}N_5O_2S$ (M+H) 406.1338; found 406.1341.

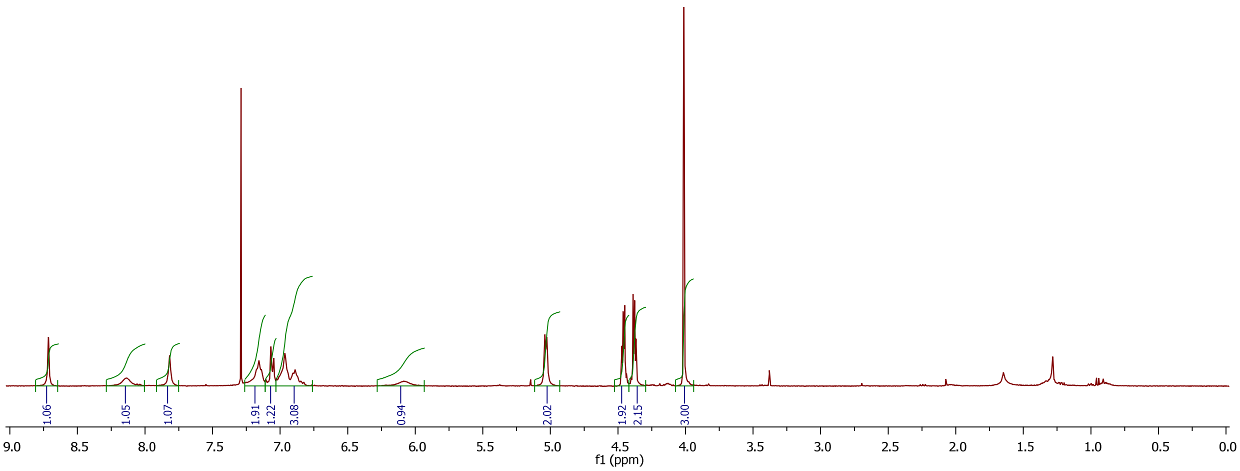

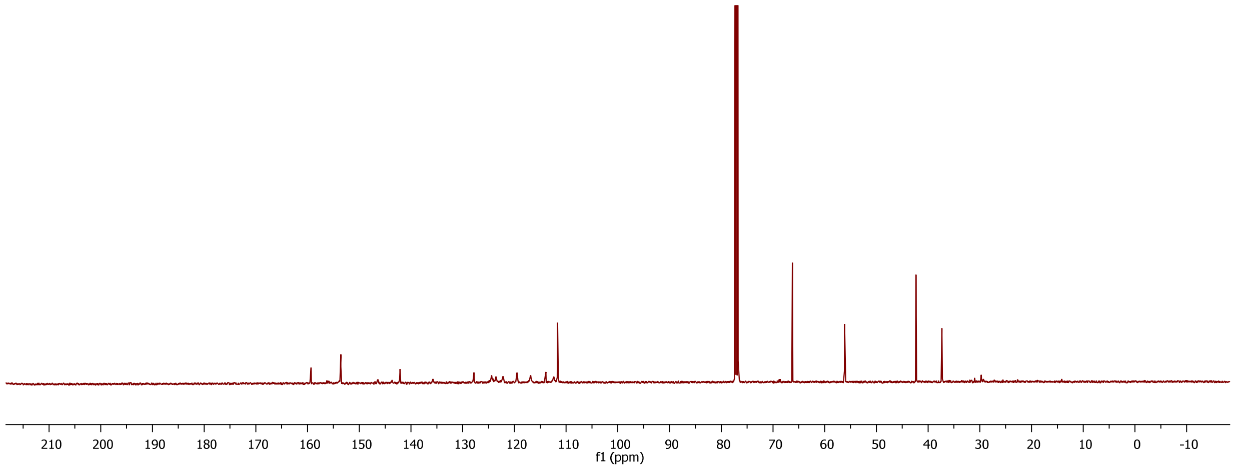

General procedure B: representative protocol for the synthesis of 8-*O*-alkylatedquinazoline analogues, synthesis and characterization for S148 (**9**), S150-S152, S154.

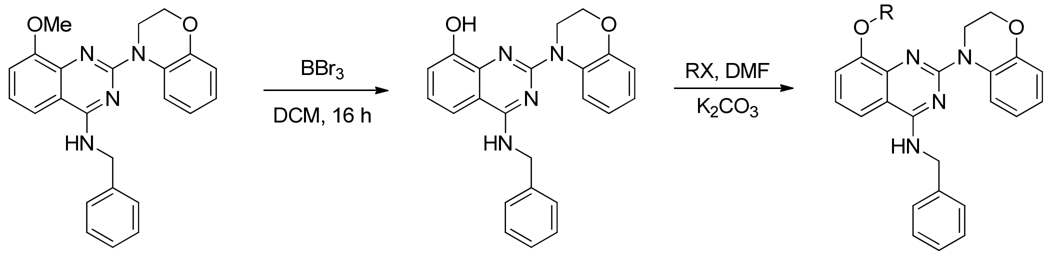

Scheme S2. Synthesis of 8-*O*-alkylated quinazolines

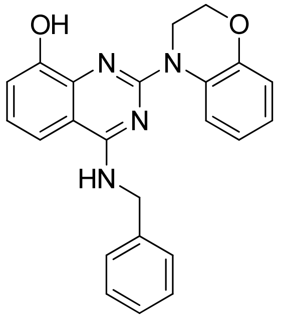

2-(*2H*-Benzo[*b*][1,4]oxazin-4(*3H*)-yl)-4-(benzylamino)quinazolin-8-ol (S148). To a solution of 2-(*2H*-benzo[*b*][1,4]oxazin-4(*3H*)-yl)-*N*-benzyl-8-methoxyquinazolin-4-amine (2.6 mg, 6.5 μmol) in 0.5 mL of DCM at 0 ºC, was added BBr_3 (39 μL, 39 μmol, 1M in DCM). The mixture was stirred at room temperature for 16 h. The reaction was quenched with slow addition of water. After extraction with DCM, the organic layer was dried over MgSO4, evaporated under vacuum to give the product. Yield: 19.0 mg, 62%. ^{1}H NMR (400 MHz, $CDCl_3$) δ 7.88 (s, 1H), 7.30 (d, *J* = 4.6 Hz, 4H), 7.28 – 7.21 (m, 1H), 7.09 - 7.06 (m, 2H), 7.02 – 6.88 (m, 2H), 6.86 (d, *J* = 6.5 Hz, 1H), 6.79 – 6.74 (m, 1H), 4.76 (d, *J* = 5.4 Hz, 2H), 4.23 (s, 4H). HRMS (m/z): calcd for $C_{23}H_{21}N_4O_2$ (M+H) 385.1665; found 385.1657.

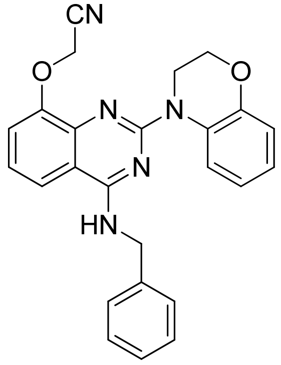

KSC-206-005

2-((2-(*2H*-Benzo[*b*][1,4]oxazin-4(*3H*)-yl)-4-(benzylamino)quinazolin-8-yl)oxy)acetonitrile (S155 or 39). To a suspension of 2-(*2H*-benzo[*b*][1,4]oxazin-4(*3H*)-yl)-4-(benzylamino)quinazolin-8-ol (0.012 g, 0.031 mmol) and K_2CO_3 (0.013 g, 0.094 mmol) in 1 mL of DMF, was added 2-chloroacetonitrile (3.0 µl, 0.047 mmol). The mixture was stirred at 80 °C for 16 h. The reaction was monitored by LC-MS. The reaction was complete after 16 h. DMF was removed under vacuum. The product was purified by SGC (EtOAc/hexanes = 1:2, Rf = 0.6). Yield: 3.3 mg, 25%. ^{1}H NMR (400 MHz, $CDCl_3$) δ 7.99 (dd, *J* = 1.5, 8.3 Hz, 1H), 7.34 – 7.29 (m, 3H), 7.29 – 7.22 (m, 4H), 7.07 – 7.01 (m, 1H), 6.93 – 6.81 (m, 2H), 6.79 – 6.71 (m, 1H), 5.84 (s, 1H), 5.11 (s, 2H), 4.73 (d, *J* = 5.4 Hz, 2H), 4.31 – 4.25 (m, 2H), 4.22 (dd, *J* = 3.1, 5.0 Hz, 2H). ^{13}C NMR (126 MHz, $CDCl_3$) δ 159.8, 156.1, 150.2, 146.5, 144.6, 138.1, 128.9, 127.7, 124.9, 123.9, 121.6, 121.1, 119.4, 116.9, 116.8, 115.8, 112.6, 66.1, 56.5, 45.5, 42.2. HRMS (m/z): calcd for $C_{25}H_{22}N_5O_2$ (M+H) 424.1773; found 424.1783.

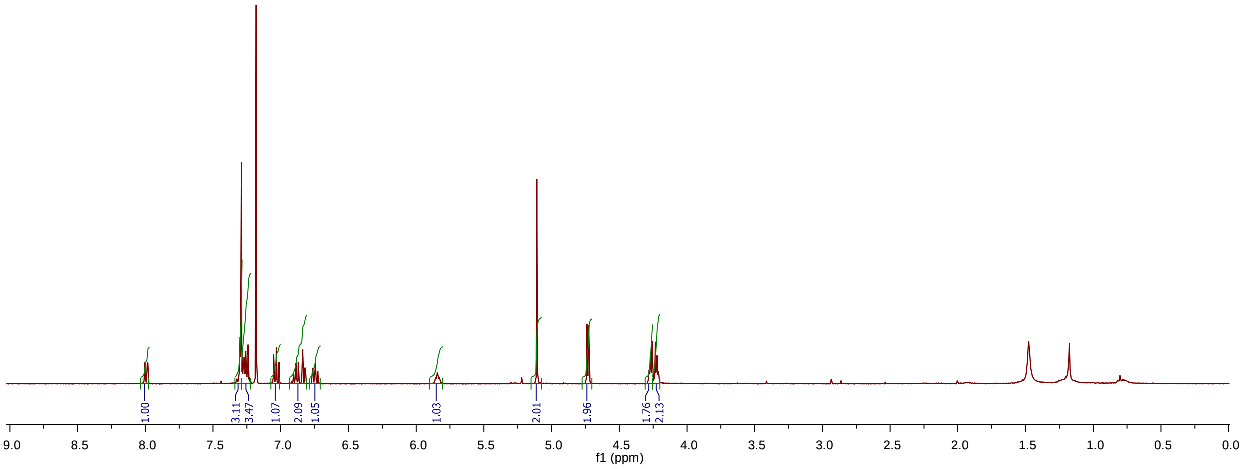

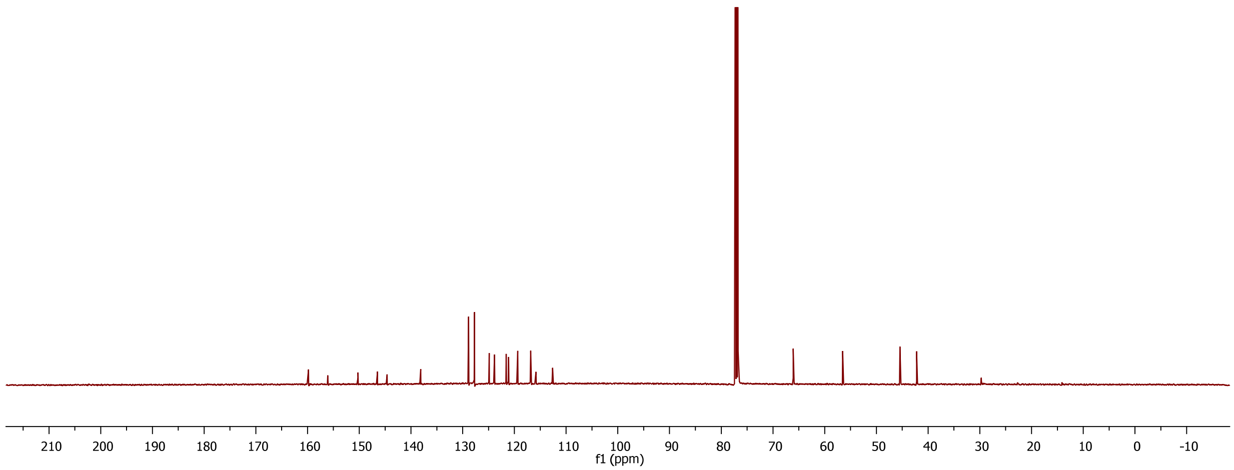

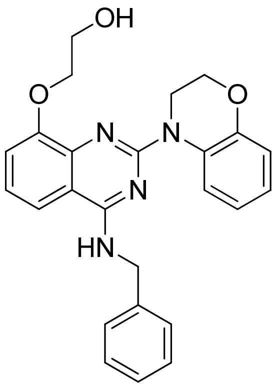

KSC-16-260

2-((2-(*2H*-Benzo[*b*][1,4]oxazin-4(*3H*)-yl)-4-(benzylamino)quinazolin-8-yl)oxy)ethanol (S150 or 33). Yield: 3.7 mg, 33%. ^{1}H NMR (400 MHz, $CDCl_3$) δ 8.05 (d, *J* = 7.3 Hz, 1H), 7.39 (dd, *J* = 5.9, 7.5 Hz, 6H), 7.20 (d, *J* = 6.7 Hz, 1H), 7.12 (t, *J* = 7.9 Hz, 1H), 6.99 (d, *J* = 6.9 Hz, 1H), 6.97 – 6.91 (m, 1H), 6.84 (t, *J* = 7.6 Hz, 1H), 4.84 (d, *J* = 5.5 Hz, 2H), 4.34 (s, 4H), 4.29 – 4.18 (m, 2H), 3.92 – 3.81 (m, 2H). ^{13}C NMR (101 MHz, $CDCl_3$) δ 159.8, 146.5, 138.2, 128.8, 127.8, 127.6, 127.4, 124.4, 124.1, 122.4, 119.5, 118.6, 117.0, 114.9, 112.2, 73.6, 66.1, 60.8, 45.4, 42.5. HRMS (m/z): calcd for $C_{25}H_{25}N_4O_3$ (M+H) 429.1927; found 429.1925.

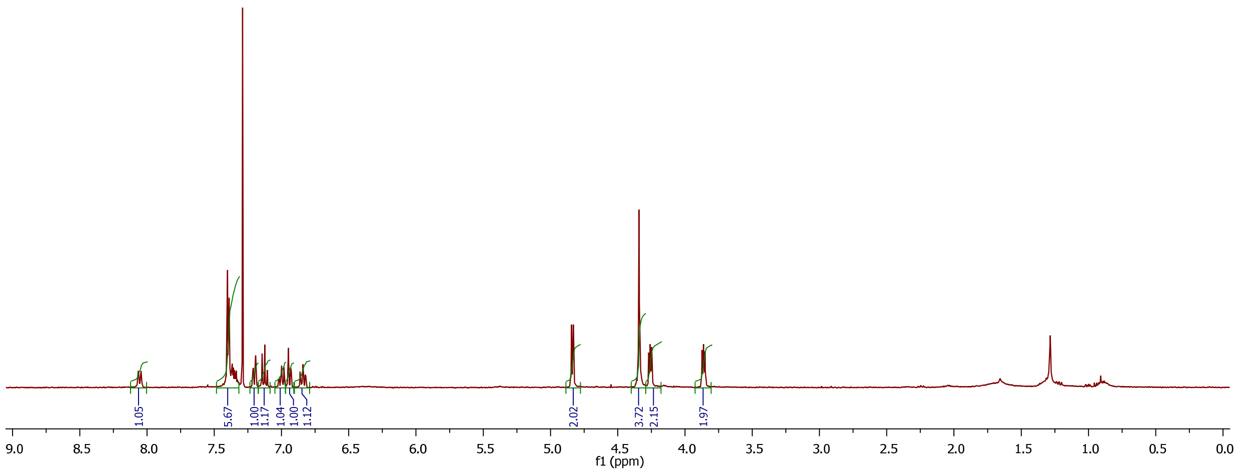

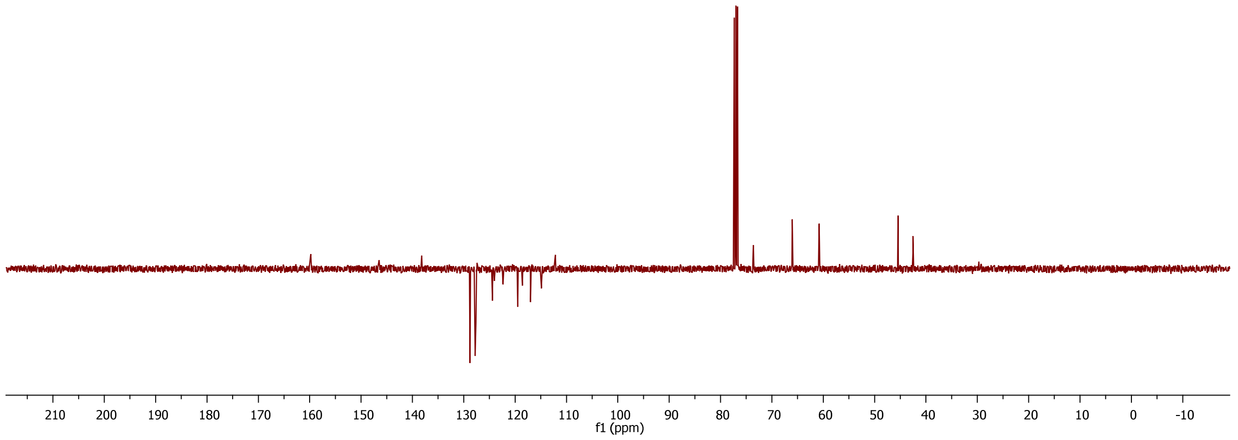

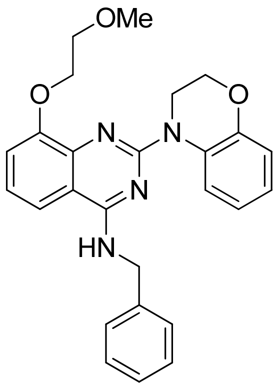

KSC-25-29

2-(*2H*-Benzo[*b*][1,4]oxazin-4(*3H*)-yl)-*N*-benzyl-8-(2-methoxyethoxy)quinazolin-4-amine (S151 or 34). Yield: 3.6 mg, 27%. ^{1}H NMR (400 MHz, $CDCl_3$) δ 8.28 (d, J = 7.7 Hz, 1H), 7.40 - 7.39 (m, 4H), 7.37 – 7.32 (m, 1H), 7.21 - 719 (m, 1H), 7.13 – 7.09 (m, 2H), 6.99 – 6.88 (m, 2H), 6.83 – 6.79 (m, 1H), 5.86 (s, br. 1H), 4.83 (d, J = 5.4 Hz, 2H), 4.45 – 4.38 (m, 2H), 4.38 – 4.27 (m, 4H), 3.97 – 3.88 (m, 2H), 3.56 (s, 3H). ^{13}C NMR (126 MHz, $CDCl_3$) δ 159.7, 156.0, 153.2, 146.2, 143.9, 138.4, 128.8, 128.1, 127.7, 127.6, 124.5, 123.2, 121.9, 119.4, 116.6, 114.0, 113.0, 111.9, 71.1, 68.8, 66.1, 59.5, 45.4, 42.1, 29.7. HRMS (m/z): calcd for $C_{26}H_{27}N_4O_3$ (M+H) 443.2083; found 443.2088.

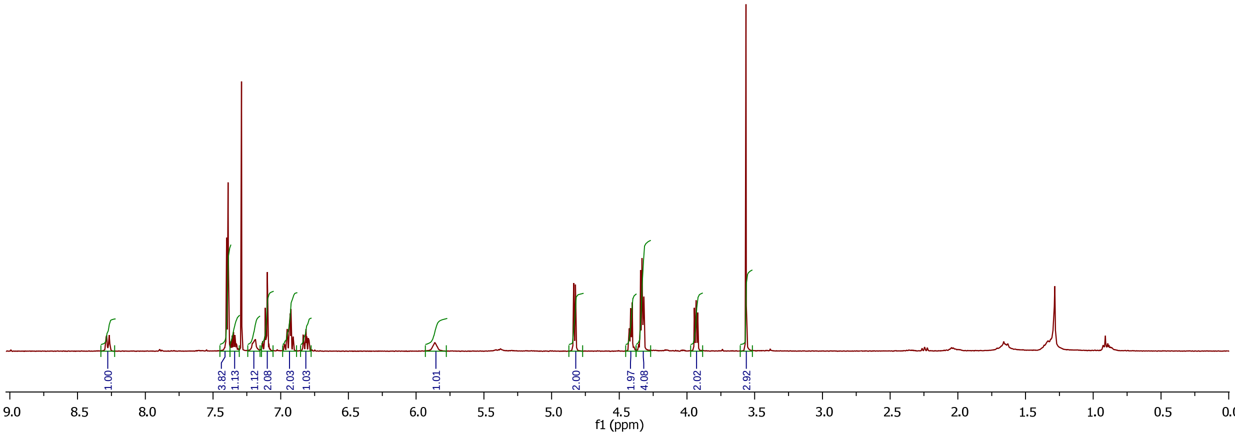

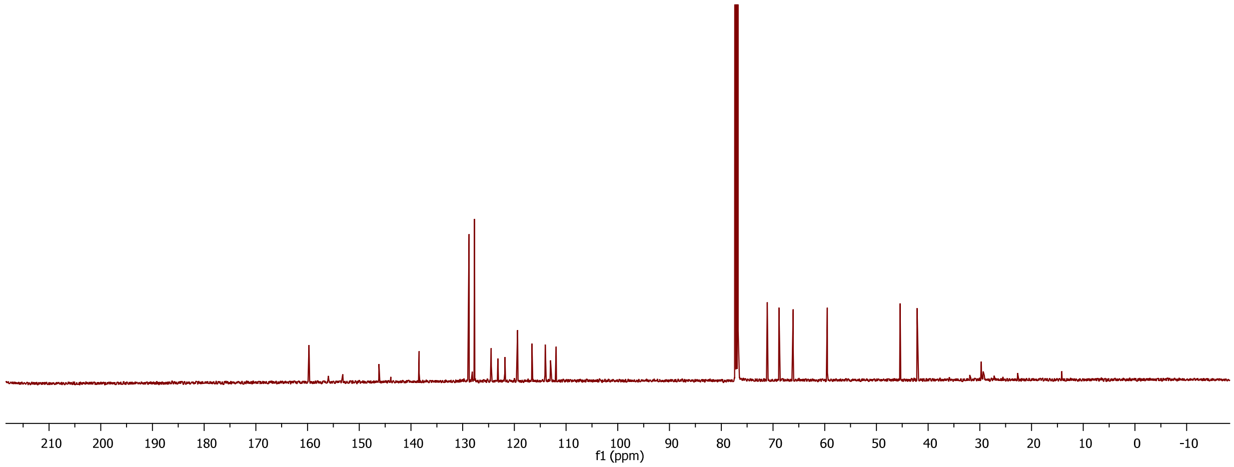

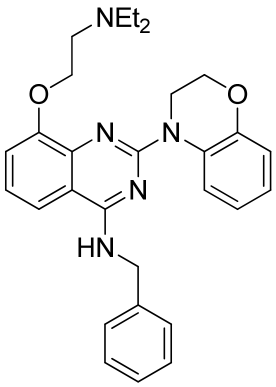

KSC-16-265

2-(*2H*-Benzo[*b*][1,4]oxazin-4(*3H*)-yl)-*N*-benzyl-8-(2-(diethylamino)ethoxy)quinazolin-4-amine (S152 or 35). Yield: 4.1 mg, 64%. ^{1}H NMR (400 MHz, $CDCl_3$) δ 7.98 – 7.79 (m, 1H), 7.31 – 7.22 (m, 6H), 7.05 (s, 2H), 6.85 (s, 2H), 6.70 (s, 1H), 4.75 (d, *J* = 5.5, 2H), 4.58 (s, 2H), 4.21 (s, 4H), 3.48 (s, 2H), 3.21 (d, *J* = 7.3, 4H), 1.32 (t, *J* = 7.3, 6H). HRMS (m/z): calcd for $C_{29}H_{34}N_5O_2$ (M+H) 484.2713; found 484.2712.

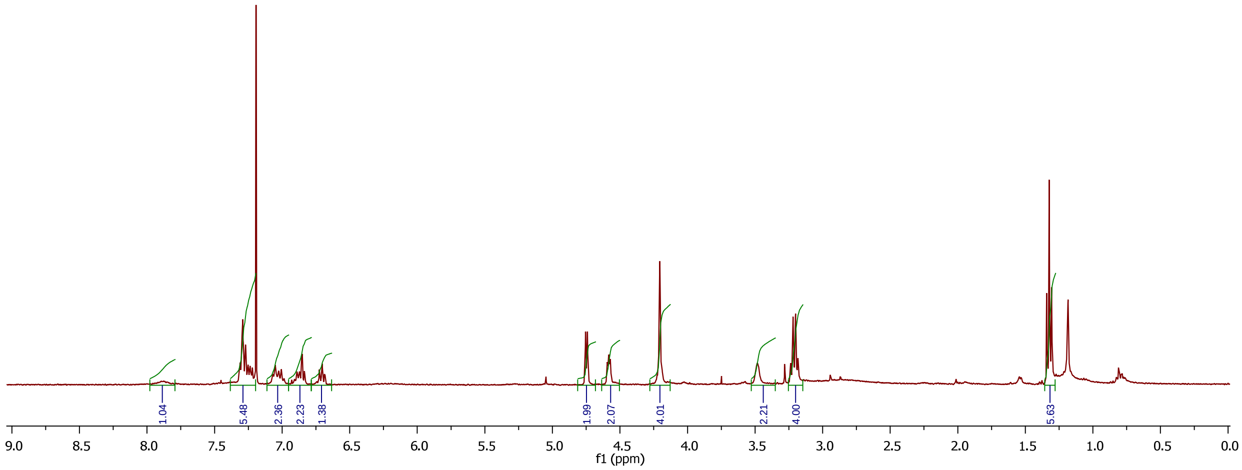

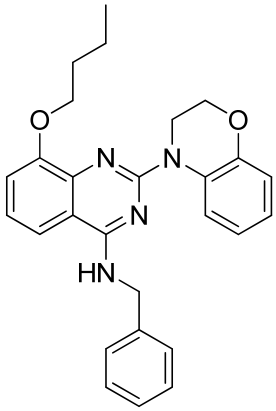

KSC-25-30

2-(*2H*-Benzo[*b*][1,4]oxazin-4(*3H*)-yl)-*N*-benzyl-8-butoxyquinazolin-4-amine (S154 or 37). Yield: 4.9 mg, 61%. ^{1}H NMR (400 MHz, $CDCl_3$) δ 8.37 (d, *J* = 8.2 Hz, 1H), 7.47 – 7.31 (m, 5H), 7.20 – 7.07 (m, 2H), 7.04 (dd, *J* = 1.5, 7.5 Hz, 1H), 7.00 – 6.89 (m, 2H), 6.89 – 6.77 (m, 1H), 5.85 (s, 1H), 4.84 (d, *J* = 5.4 Hz, 2H), 4.48 – 4.38 (m, 2H), 4.35 – 4.32 (m, 2H), 4.15 (t, *J* = 6.5 Hz, 2H), 2.04 – 1.89 (m, 2H), 1.72 – 1.62 (m, 2H), 1.08 (t, *J* = 7.4 Hz, 3H). ^{13}C NMR (101 MHz, $CDCl_3$) δ 159.7, 155.9, 153.6, 146.2, 143.7, 138.5, 128.8, 128.3, 127.7, 127.6, 124.4, 123.1, 121.9, 119.5, 116.6, 112.7, 112.1, 111.8, 68.6, 66.1, 45.4, 42.1, 31.4, 19.4, 14.0. HRMS (m/z): calcd for $C_{27}H_{29}N_4O_2$ (M+H) 441.2291; found 441.2296.

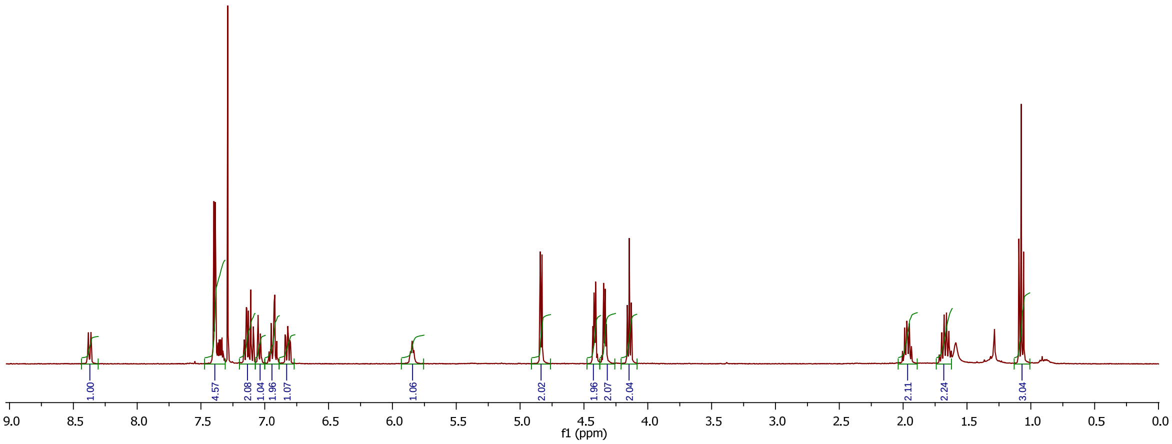

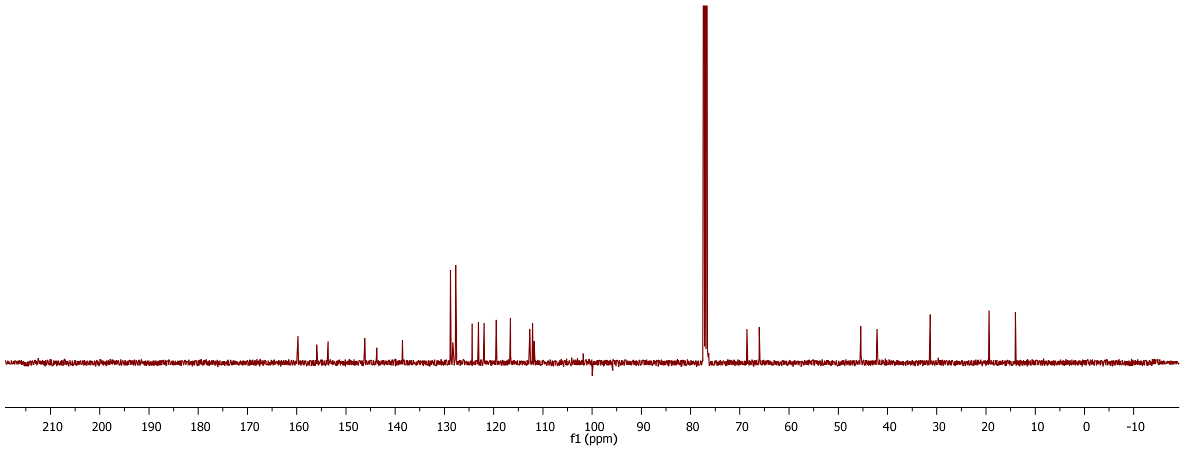

General Procecedure C: representative protocol for the one-pot synthesis of quinazoline analogues, synthesis and characterization for S129, S126-S128, S130-S141.

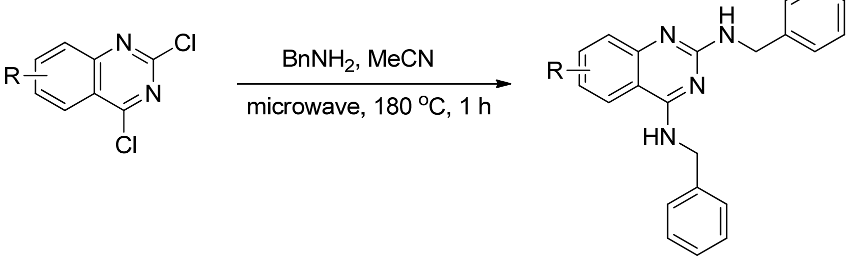

Scheme S3. Quinazoline synthesis via the one-pot protocol.

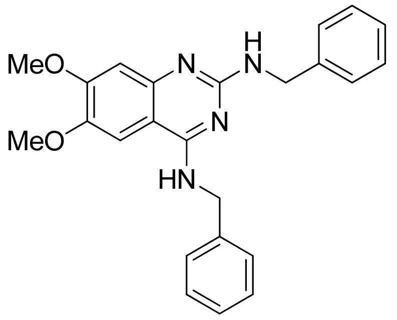

KSC-16-102

***N*²,*N*⁴-Dibenzyl-6,7-dimethoxyquinazoline-2,4-diamine (S129)**. To a suspension of 2,4-dichloro-6,7-dimethoxyquinazoline (50.0 mg, 0.19 mmol) in CH_3CN (2 mL) was added benzylamine (0.11 mL, 0.96 mmol, 5 equiv.). The mixture was heated to 180 °C for 1 h under microwave irradiation. The solvent was removed under vacuum, the residue was suspended in EtOAc, washed with saturated $NaHCO_3$, and the layers were separated. The organic layer was dried over $MgSO_4$ and concentrated under vacuum. The residue was purified by SGC (EtOAc, Rf = 0.5) to give the product (66.0 mg, 85%). ^{1}H NMR (400 MHz, $CDCl_3$) δ 7.46 – 7.16 (m, 10H), 6.94 (s, 1H), 6.89 (s, 1H), 6.31 (s, 1H), 5.41 (s, 1H), 4.80 (d, J = 5.5 Hz, 2H), 4.70 (d, J = 5.7 Hz, 2H), 3.87 (s, 3H), 3.80 (s, 3H). ^{13}C NMR (101 MHz, $CDCl_3$) δ 159.3, 159.0, 154.6, 148.5, 145.6, 140.1, 139.1, 128.6, 128.5, 128.1, 127.5, 127.4, 126.9, 105.3, 103.8, 101.1, 56.1, 56.0, 45.6, 45.0. HRMS (m/z): calcd for $C_{24}H_{25}N_4O_2$ (M+H) 401.1978; found 401.1978.

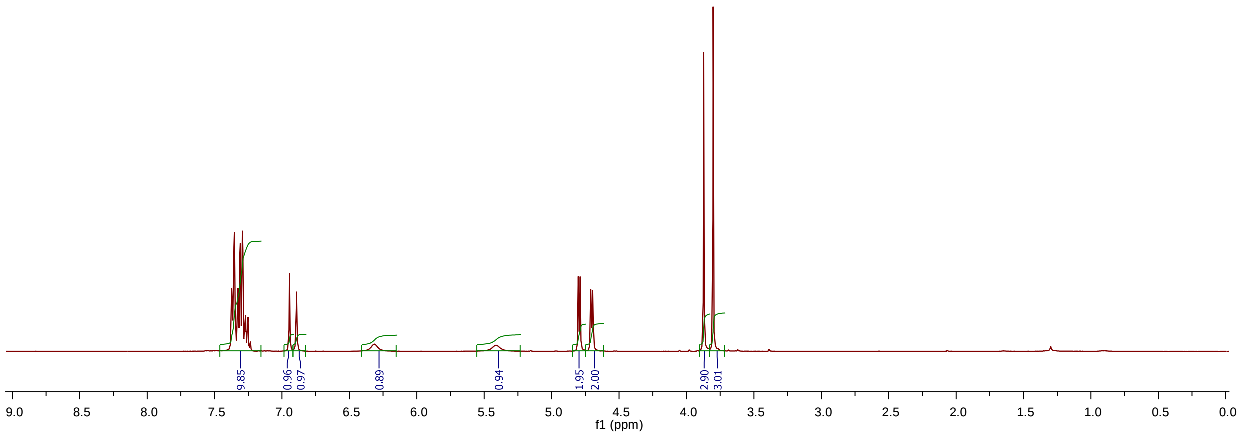
9.85
0.96
0.97
0.89
0.94
1.95
2.00
2.90
3.01
9.0 8.5 8.0 7.5 7.0 6.5 6.0 5.5 5.0 4.5 4.0 3.5 3.0 2.5 2.0 1.5 1.0 0.5 0.0
f1 (ppm)

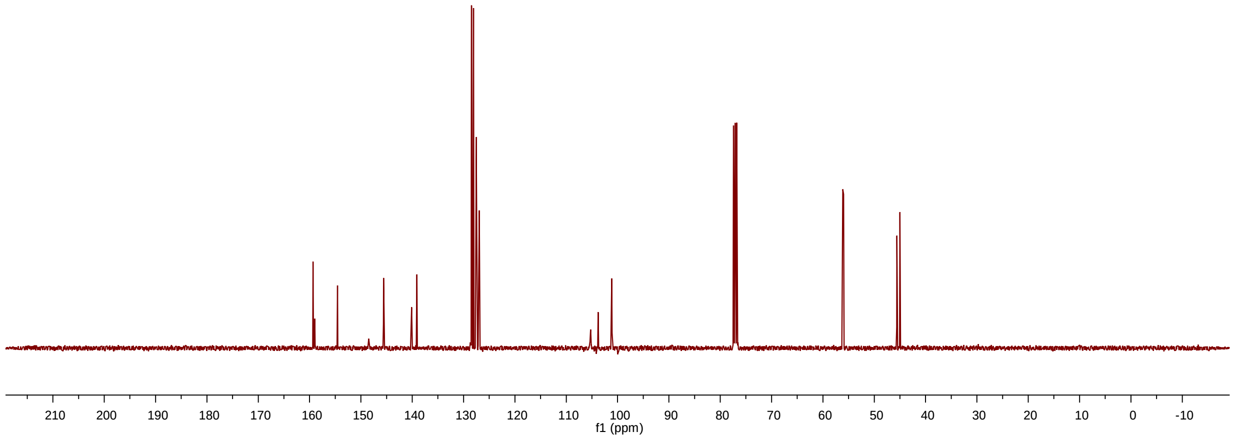
210 200 190 180 170 160 150 140 130 120 110 100 90 80 70 60 50 40 30 20 10 0 -10
f1 (ppm)

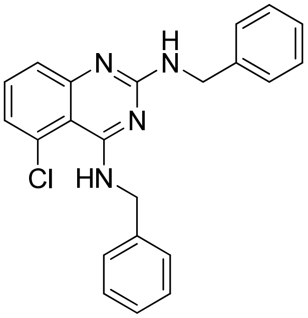

KSC-16-114

***N*^{2},*N*^{4}-Dibenzyl-5-chloroquinazoline-2,4-diamine (S126)**. Yield: 66.0 mg, 82%. ^{1}H NMR (400 MHz, $CDCl_3$) δ 7.93 (s, br. 1H), 7.51 – 7.21 (m, 12H), 7.06 (dd, *J* = 2.5, 6.3 Hz, 1H), 5.60 (s, br. 1H), 4.78 (d, *J* = 5.2 Hz, 2H), 4.72 (d, *J* = 6.0 Hz, 2H). ^{13}C NMR (101 MHz, CDCl3) δ 159.6, 159.1, 155.2, 140.0, 138.5, 131.9, 129.1, 128.7, 128.5, 127.7, 127.6, 127.4, 127.0, 125.3, 123.3, 109.1, 45.6, 45.4. HRMS (m/z): calcd for $C_{22}H_{20}ClN_4$ (M+H) 375.1376; found 375.1377.

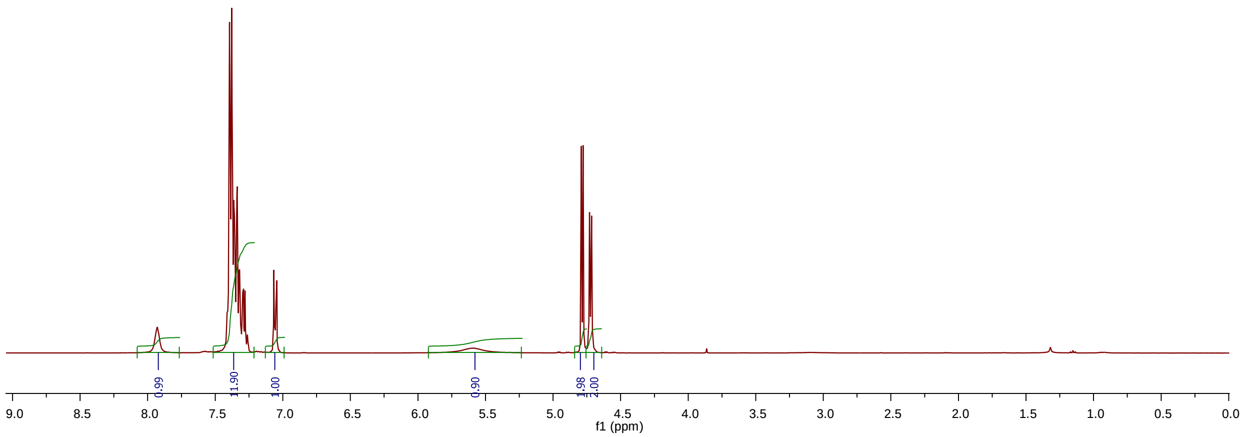

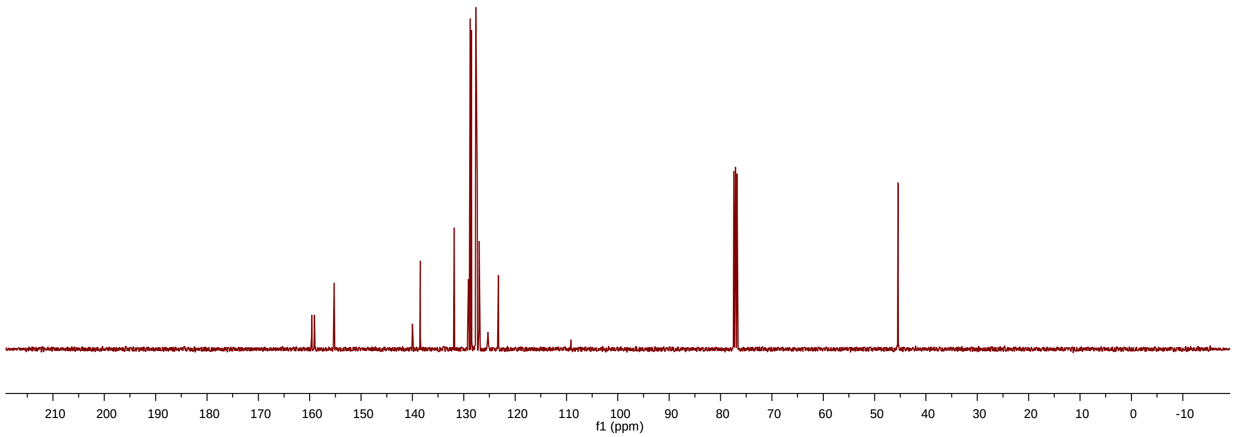

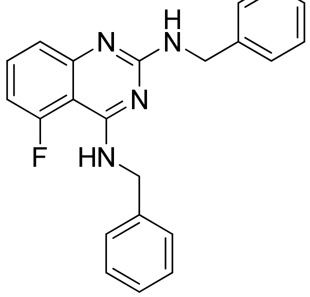

KSC-16-115

N^2,N^4-Dibenzyl-5-fluoroquinazoline-2,4-diamine (S127). Yield: 12.8 mg, 39%. ^{1}H NMR (400 MHz, $CDCl_3$) δ 7.30 (dd, *J* = 8.2, 14.8 Hz, 1H), 7.22 – 7.17 (m , 8H), 7.12 – 7.08 (m, 2H), 6.82 – 6.63 (m, 1H), 6.58 (dd, *J* = 7.9, 12.7 Hz, 1H), 4.69 – 4.61 (m, 2H), 4.57 (d, *J* = 5.9 Hz, 2H). ^{13}C NMR (101 MHz, CDCl3) δ 161.2, 159.8, 159.0, 158.7, 154.5, 139.8, 138.5, 132.5, 132.4, 128.7, 128.5, 127.7, 127.6, 127.5, 127.1, 121.3, 106.3, 106.1, 45.5, 45.0. HRMS (m/z): calcd for $C_{22}H_{20}FN_4$ (M+H) 359.1672; found 359.1676.

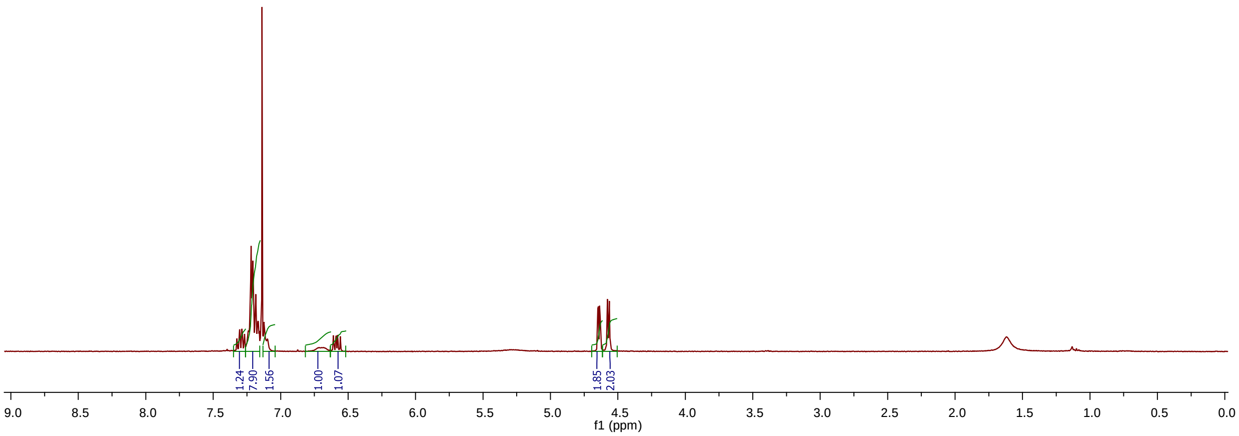

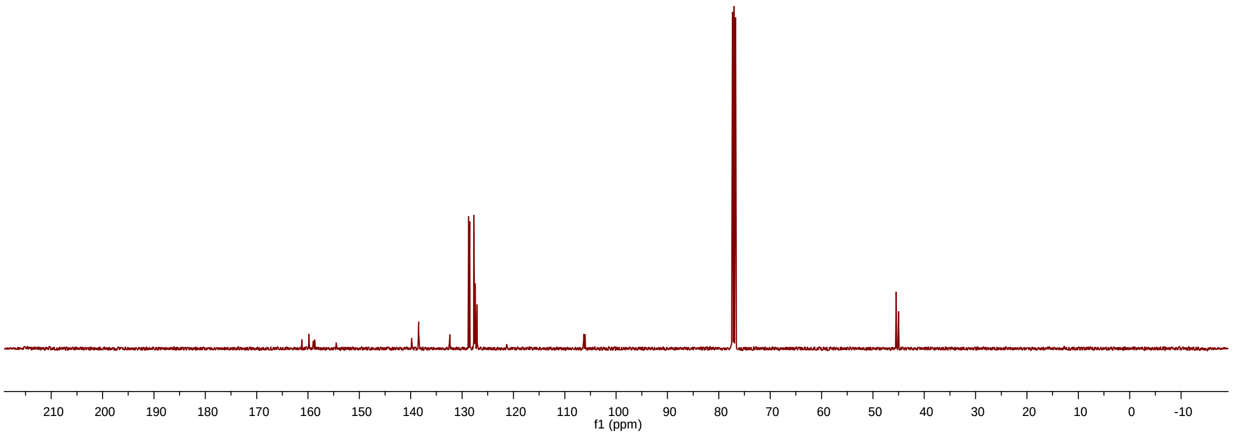

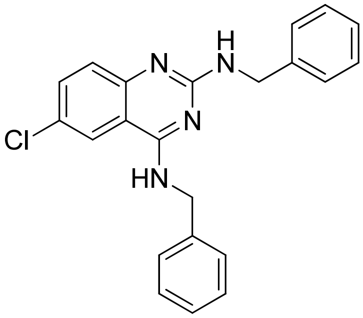

KSC-16-117

***N*^{2},*N*^{4}-Dibenzyl-6-chloroquinazoline-2,4-diamine (S128)**. Yield: 56.0 mg, 70%. ^{1}H NMR (400 MHz, $CDCl_3$) δ 7.55 – 7.21 (m, 13H), 5.78 (s, br. 1H), 5.40 (s, br. 1H), 4.77 (d, *J* = 5.4 Hz, 2H), 4.73 (d, *J* = 5.9 Hz, 2H). ^{13}C NMR (101 MHz, $CDCl_3$) δ 159.5, 159.2, 150.7, 139.8, 138.3, 133.2, 128.8, 128.5, 128.0, 127.7, 127.6, 127.2, 127.1, 126.0, 120.3, 45.6, 45.2. HRMS (m/z): calcd for $C_{22}H_{20}ClN_4$ (M+H) 375.1376; found 375.1376.

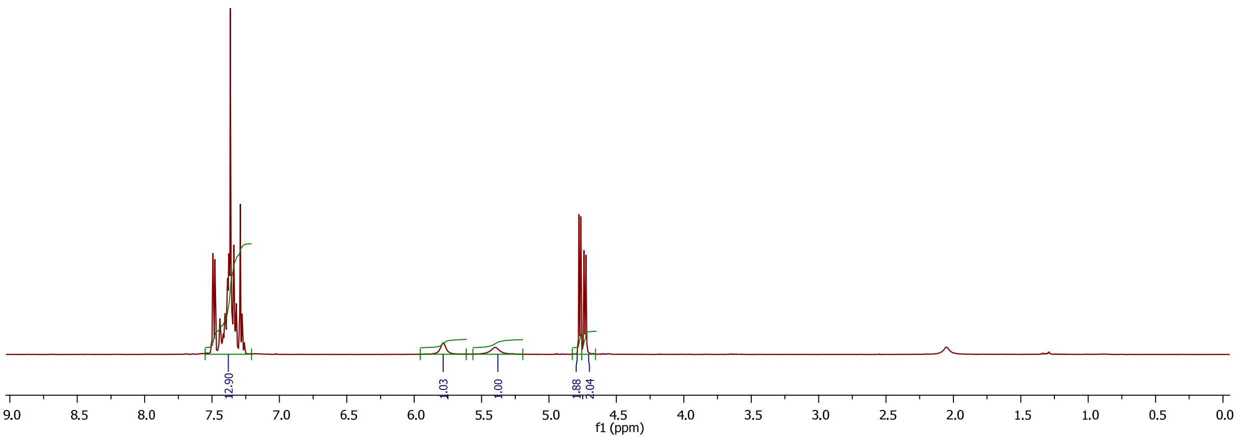

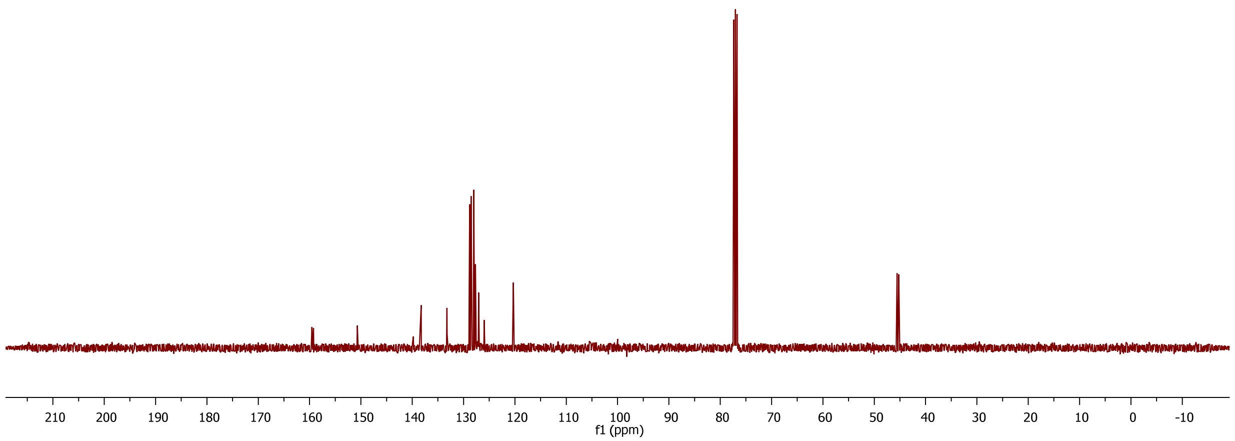

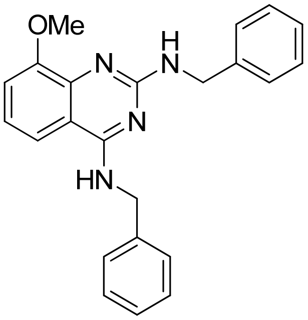

KSC-16-121

***N*[2],*N*[4]-Dibenzyl-8-methoxyquinazoline-2,4-diamine (S130 or 14)**. Yield: 14.9 mg, 18%. ^{1}H NMR (400 MHz, $CDCl_3$) δ 7.44 – 7.18 (m, 11H), 7.11 (dd, *J* = 1.6, 7.9 Hz, 1H), 7.07 – 6.93 (m, 2H), 5.86 (s, br. 1H), 5.49 (s, br. 1H),), 4.78 (d, *J* = 5.7 Hz, 2H), 4.75 (d, *J* = 5.7 Hz, 2H), 3.99 (s, 3H). ^{13}C NMR (101 MHz, $CDCl_3$) δ 160.1, 159.3, 153.2, 144.2, 140.2, 138.7, 128.7, 128.4, 128.0, 127.5, 126.8, 120.4, 112.5, 111.2, 110.9, 55.9, 45.7, 45.2. HRMS (m/z): calcd for $C_{23}H_{23}N_4O$ (M+H) 371.1872; found 371.1871.

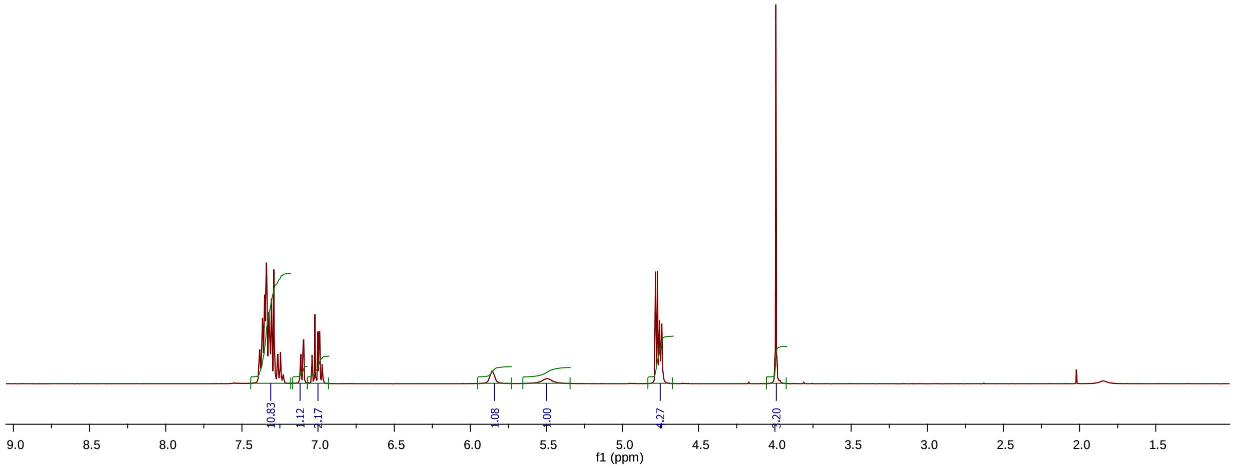

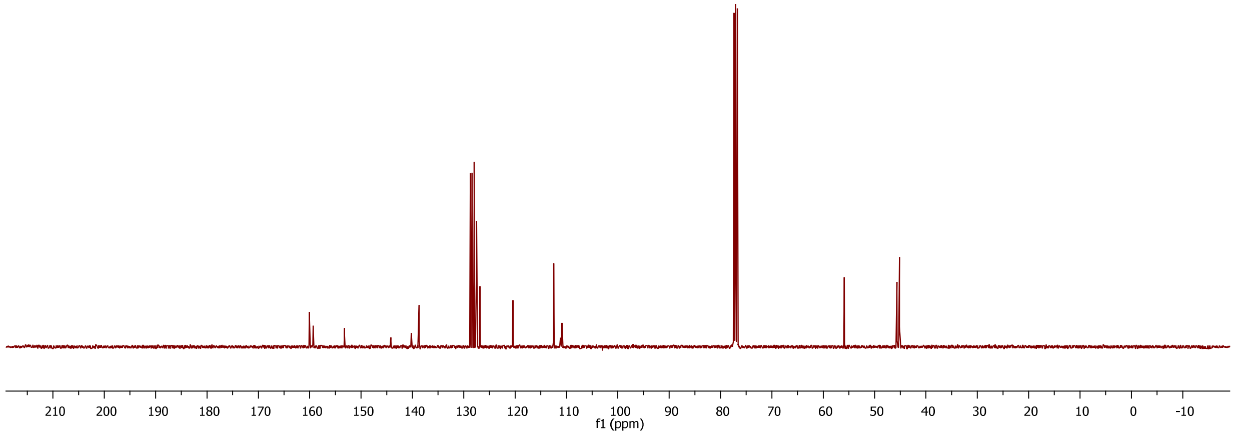

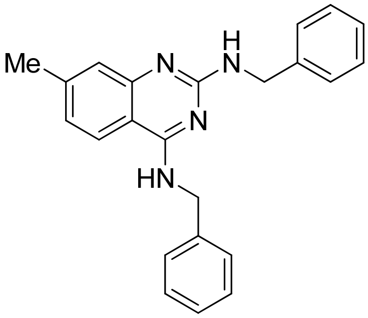

KSC-16-118

***N*[2],*N*[4]-Dibenzyl-7-methylquinazoline-2,4-diamine (S131)**. Yield: 83.0 mg, 99%. ^{1}H NMR (400 MHz, $CDCl_3$) δ 7.47 – 7.19 (m, 12H), 6.93 (dd, *J* = 1.4, 8.3 Hz, 1H), 5.75 (s, br. 1H), 5.28 (s, br. 1H), 4.79 (d, *J* = 5.5 Hz, 2H), 4.75 (d, *J* = 4.5 Hz, 2H), 2.44 (s, 3H). ^{13}C NMR (101 MHz, $CDCl_3$) δ 156.0, 159.7, 152.4, 143.3, 140.2, 138.8, 128.7, 128.5, 128.0, 127.6, 127.5, 126.9, 125.2, 123.0, 120.6, 108.9, 45.6, 45.0, 21.8. HRMS (m/z): calcd for $C_{23}H_{23}N_4$ (M+H) 355.1923; found 355.1924.

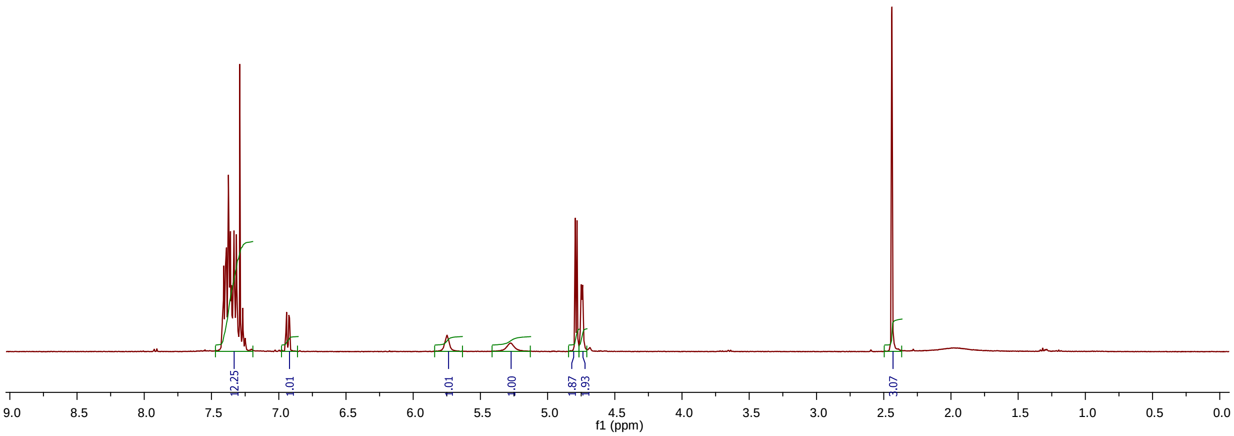

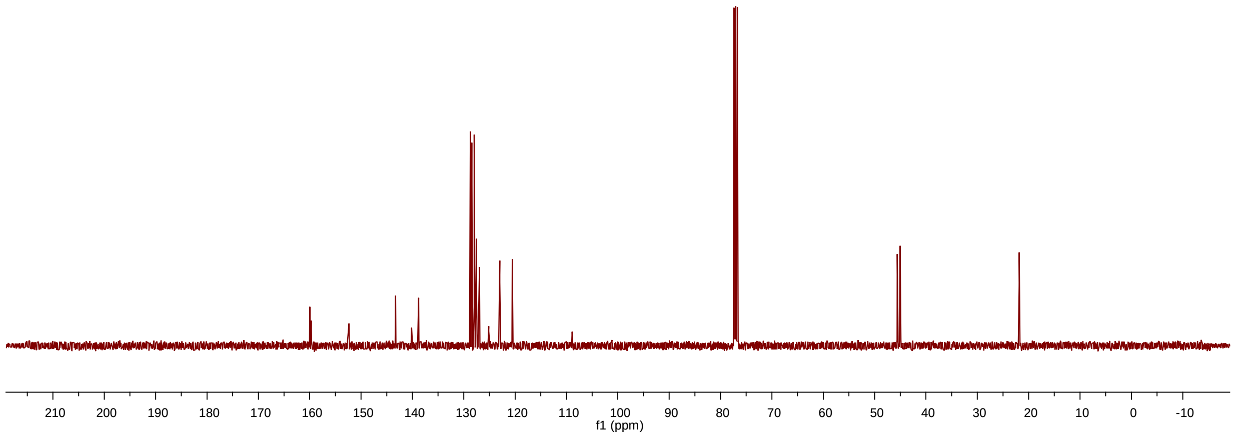

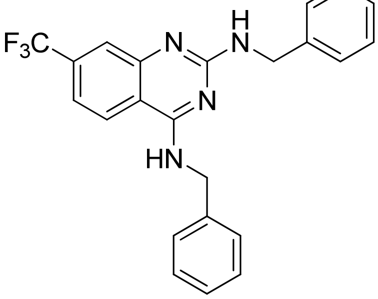

KSC-16-160c

***N*[2],*N*[4]-Dibenzyl-7-(trifluoromethyl)quinazoline-2,4-diamine (S132)**. Yield: 75.0 mg, 98%. ^{1}H NMR (400 MHz, $CDCl_3$) δ 7.78 (s, 1H), 7.60 (d, *J* = 8.5 Hz, 1H), 7.39 – 7.33 (m, 9H), 7.32 – 7.21 (m, 2H), 5.91 (s, br. 1H), 5.51 (s, br. 1H), 4.80 (d, *J* = 5.4 Hz, 2H), 4.75 (d, *J* = 5.9 Hz, 2H). ^{13}C NMR (101 MHz, $CDCl_3$) δ 160.0, 159.7, 152.0, 139.6, 138.2, 134.8, 134.5, 134.2, 133.9, 128.8, 128.6, 128.0, 127.8, 127.6, 127.1, 125.2, 123.2, 122.5, 121.9, 119.7, 116.64, 116.61, 112.8, 45.5, 45.2.HRMS (m/z): calcd for $C_{23}H_{20}F_3N_4$ (M+H) 409.1640; found 409.1642.

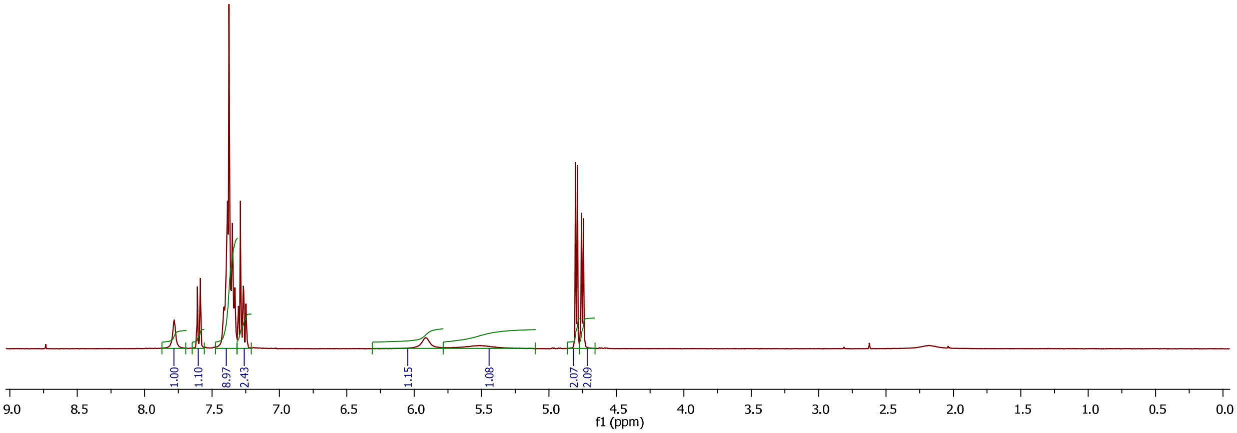

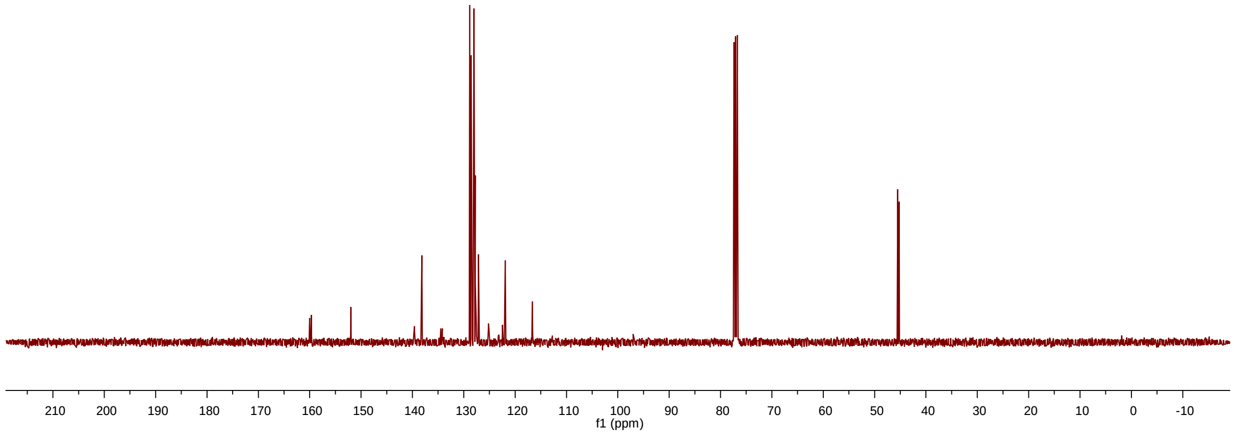

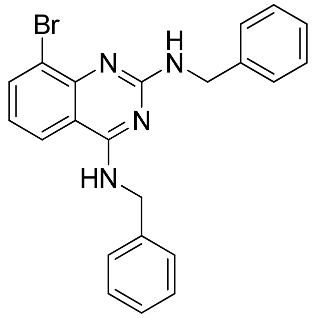

KSC-16-153

***N*²,*N*⁴-Dibenzyl-8-bromoquinazoline-2,4-diamine (S133)**. Yield: 75.0 mg, 99%. ^{1}H NMR (400 MHz, $CDCl_3$) δ 7.86 (dd, *J* = 1.1, 7.6 Hz, 1H), 7.68 – 7.19 (m, 11H), 6.90 (t, *J* = 7.8 Hz, 1H), 5.89 (s, br. 1H), 5.74 – 5.16 (m, 1H), 4.77 (d, *J* = 5.4 Hz, 4H). ^{13}C NMR (101 MHz, $CDCl_3$) δ 160.3, 159.8, 149.9, 139.9, 138.4, 136.1, 128.8, 128.4, 128.0, 127.6, 127.0, 120.9, 120.4, 45.7, 45.3. HRMS (m/z): calcd for $C_{22}H_{20}BrN_4$ (M+H) 419.0871 and 421.0851; found 421.0848.

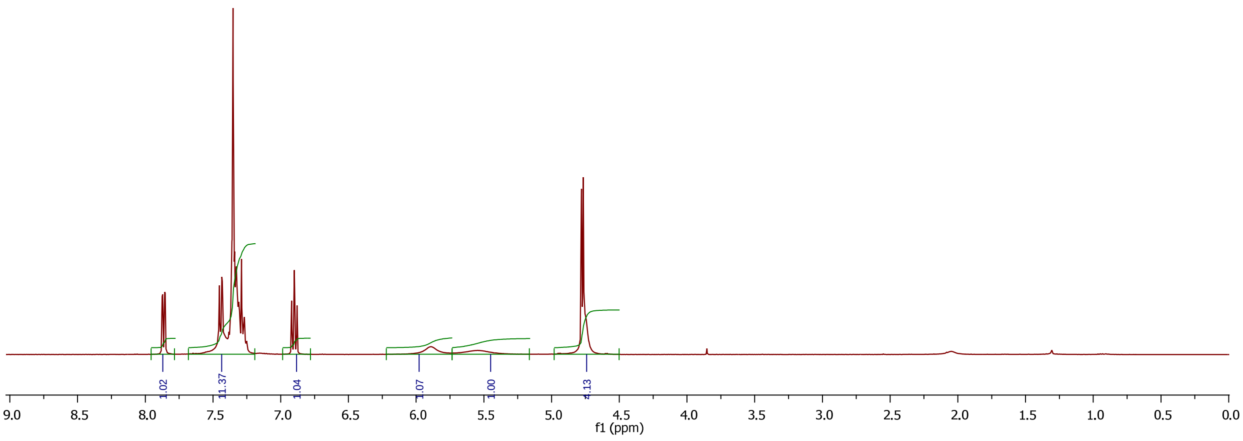

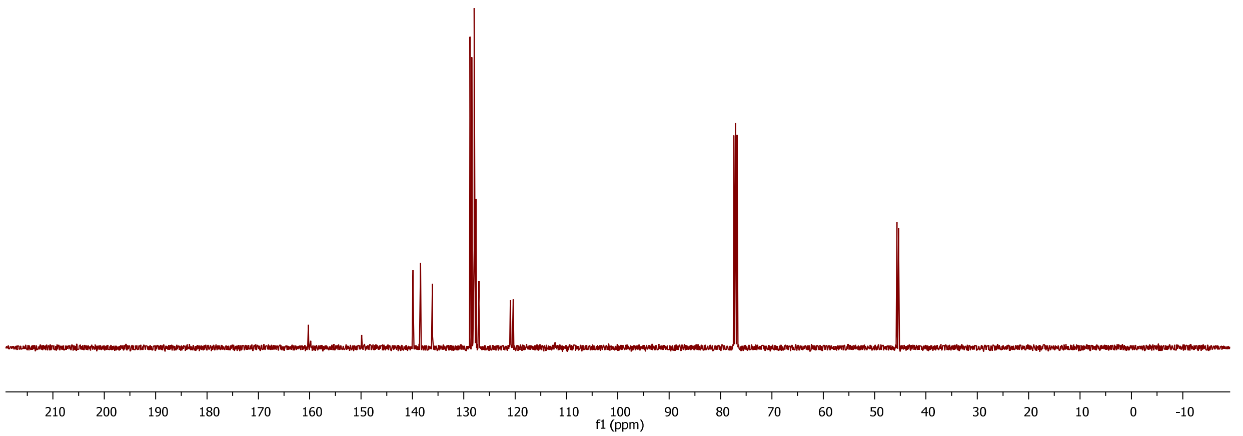

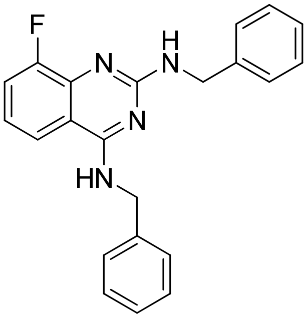

KSC-16-154

***N^2,N^4*-Dibenzyl-8-fluoroquinazoline-2,4-diamine (S134)**. Yield: 70.0 mg, 85%. ^{1}H NMR (400 MHz, $CDCl_3$) δ 7.47 – 7.12 (m, 12H), 6.95 (td, *J* = 4.9, 8.0 Hz, 1H), 6.15 (s, br. 1H), 5.68 (s, br. 1H), 4.77 (d, *J* = 5.5 Hz, 2H), 4.74 (d, *J* = 5.7 Hz, 2H). ^{13}C NMR (101 MHz, $CDCl_3$) δ 159.7, 159.6, 157.4, 154.9, 142.7, 142.6, 140.0, 138.5, 128.7, 128.5, 128.0, 127.6, 127.0, 119.8, 119.7, 117.0, 116.9, 116.6, 116.6, 112.8, 45.6, 45.1. . HRMS (m/z): calcd for $C_{22}H_{20}FN_4$ (M+H) 359.1672; found 359.1675.

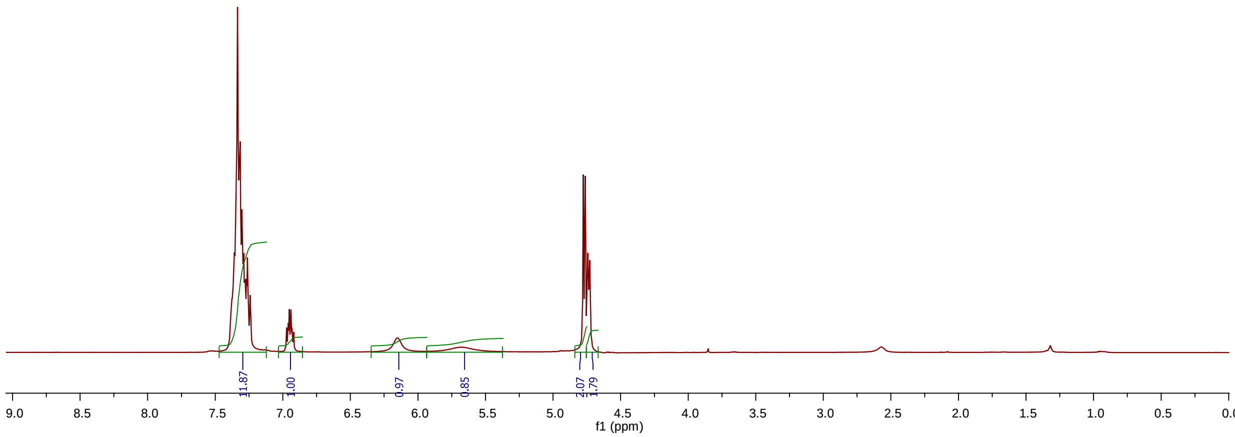

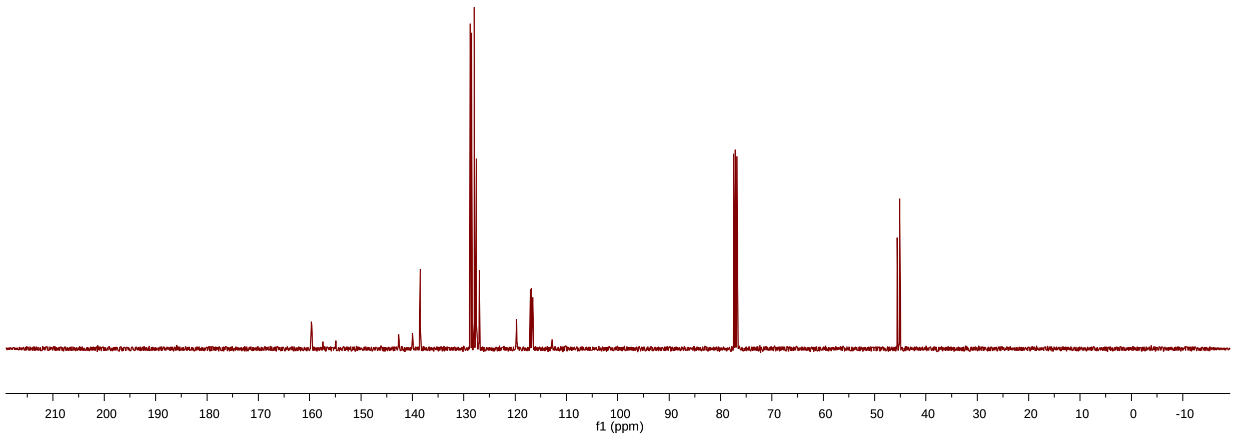

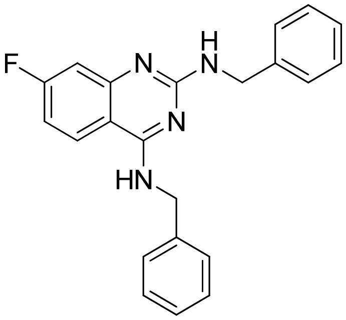

KSC-16-156

***N*[2],*N*[4]-Dibenzyl-7-fluoroquinazoline-2,4-diamine (S135)**. Yield: 37.0 mg, 45%. ^{1}H NMR (400 MHz, $CDCl_3$) δ 7.49 (dd, *J* = 5.9, 9.0 Hz, 1H), 7.46 – 7.31 (m, 9H), 7.30 – 7.23 (m, 1H), 7.12 (d, *J* = 10.3 Hz, 1H), 6.82 (td, *J* = 2.5, 8.6 Hz, 1H), 5.81 (s, br. 1H), 5.41 (s, br. 1H), 4.78 (d, *J* = 5.4 Hz, 2H), 4.73 (d, *J* = 5.9 Hz, 2H). ^{13}C NMR (101 MHz, $CDCl_3$) δ 167.0, 164.5, 160.1, 159.8, 154.5, 154.3, 139.8, 138.5, 128.8, 128.5, 128.0, 127.6, 127.1, 123.1, 123.0, 110.4, 110.1, 45.5, 45.1. HRMS (m/z): calcd for $C_{22}H_{20}FN_4$ (M+H) 359.1672; found 359.1674.

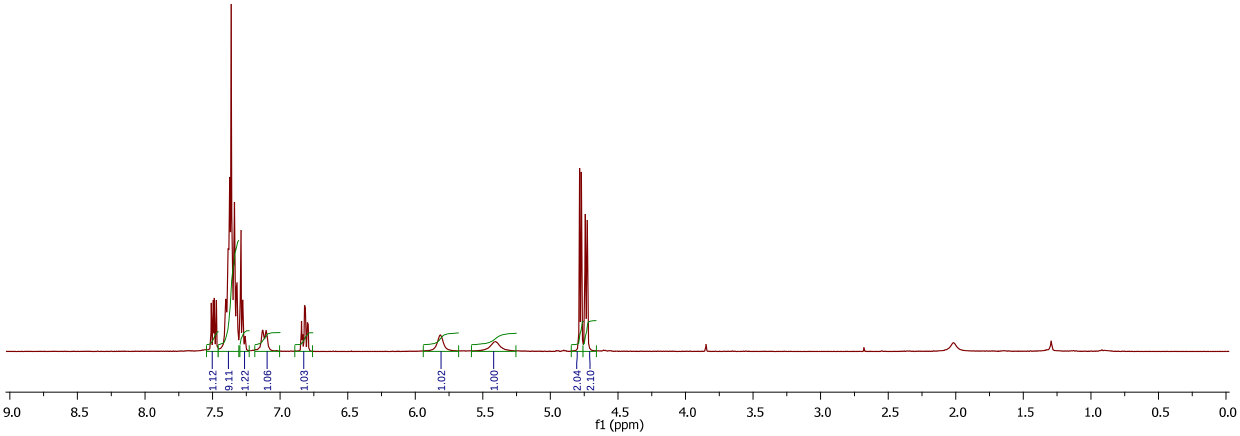

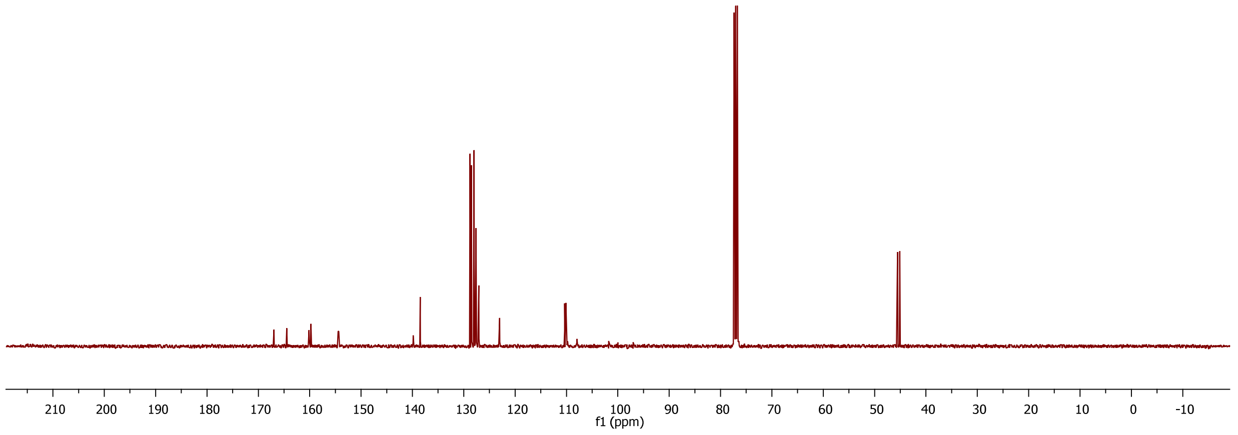

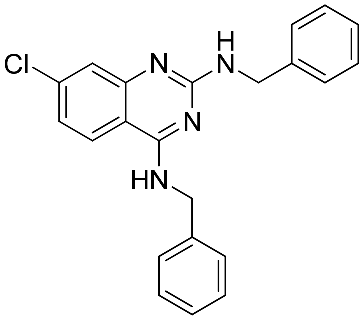

KSC-16-159

***N*^{2},*N*^{4}-Dibenzyl-7-chloroquinazoline-2,4-diamine (S136)**. Yield: 74.0 mg, 92%. ^{1}H NMR (400 MHz, $CDCl_3$) δ 7.54 (s, br. 1H), 7.50 – 7.37 (m, 10H), 7.32 (d, *J* = 7.0 Hz, 1H), 7.08 (dd, *J* = 2.1, 8.7 Hz, 1H), 5.82 (s, br. 1H), 5.42 (s, br. 1H), 4.83 (d, *J* = 5.4 Hz, 2H), 4.79 (d, *J* = 5.9 Hz, 2H). ^{13}C NMR (101 MHz, $CDCl_3$) δ 160.1, 159.8, 153.3, 139.8, 138.7, 138.4, 128.8, 128.5, 128.0, 127.7, 127.6, 127.1, 124.8, 122.2, 121.6, 109.5, 45.5, 45.1. HRMS (m/z): calcd for $C_{22}H_{20}ClN_4$ (M+H) 375.1376; found 375.1377.

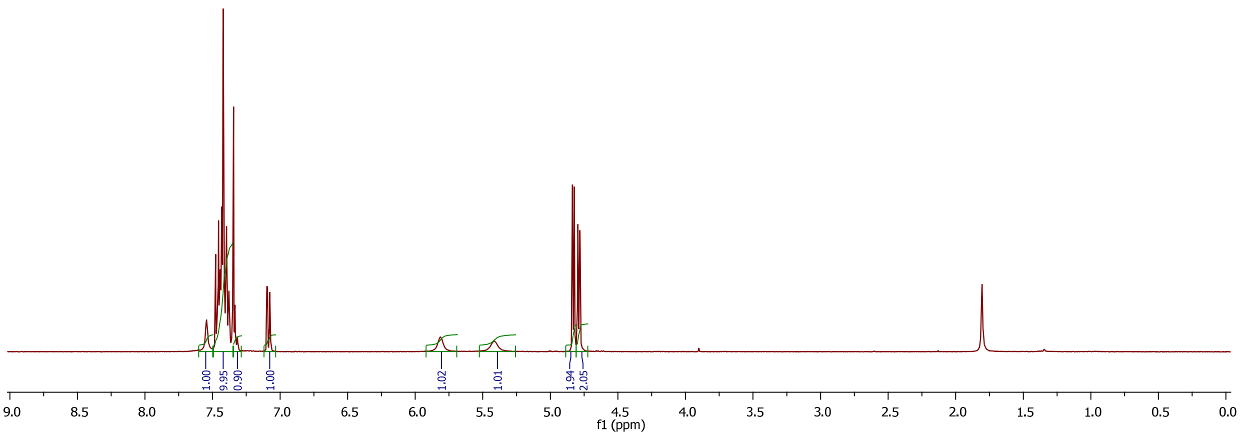

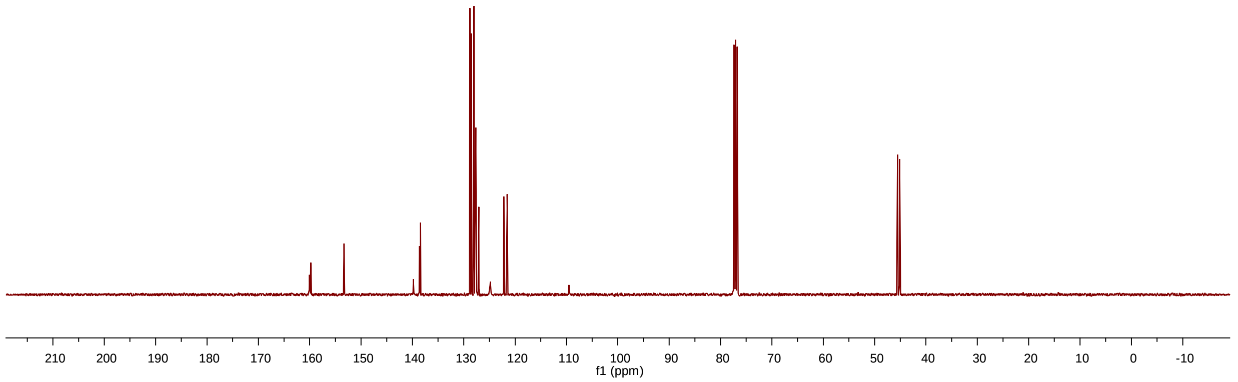

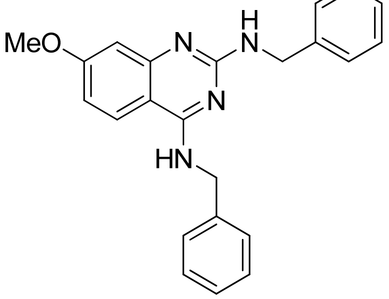

KSC-16-166

***N*[2],*N*[4]-Dibenzyl-7-methoxyquinazoline-2,4-diamine (S137)**. Yield: 47.0 mg, 58%. ^{1}H NMR (400 MHz, $CDCl_3$) δ 7.33 – 7.08 (m, 11H), 6.75 (s, 1H), 6.57 (dd, *J* = 2.5, 8.9 Hz, 1H), 5.72 (s, br. 1H), 5.32 (s, br. 1H), 4.64 (d, *J* = 5.5 Hz, 2H), 4.61 (d, *J* = 5.8 Hz, 2H), 3.72 (s, 3H). ^{13}C NMR (101 MHz, CDCl3) δ 163.4, 160.1, 159.9, 154.5, 140.1, 138.9, 128.7, 128.5, 128.0, 127.6, 127.5, 127.0, 122.3, 112.7, 105.2, 104.9, 55.4, 45.6, 45.0. HRMS (m/z): calcd for $C_{23}H_{23}N_4O$ (M+H) 371.1872; found 371.1872.

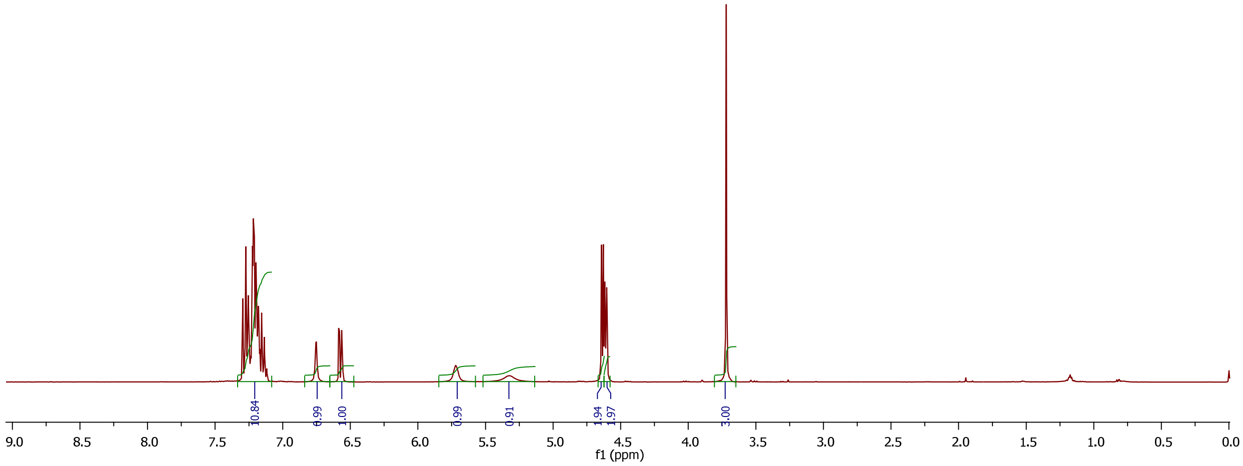

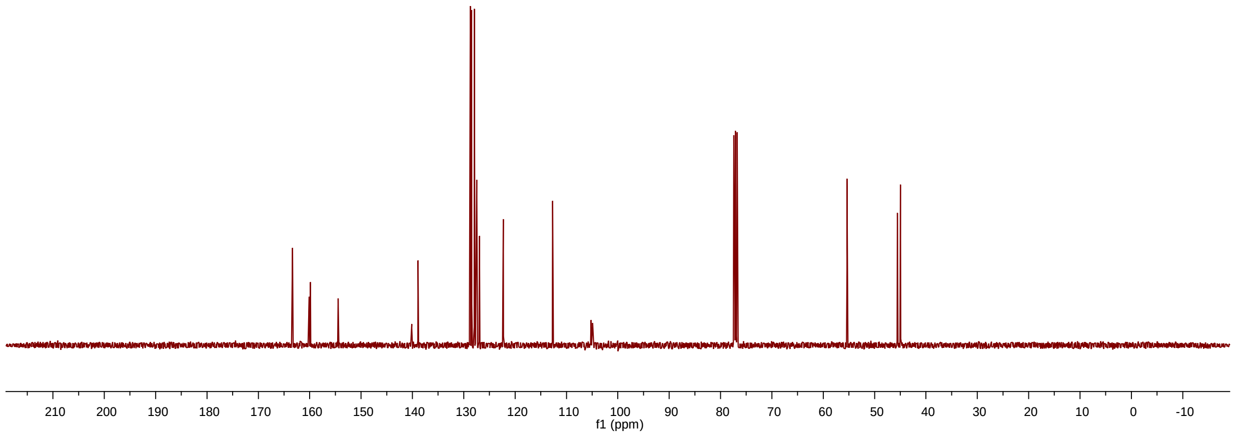

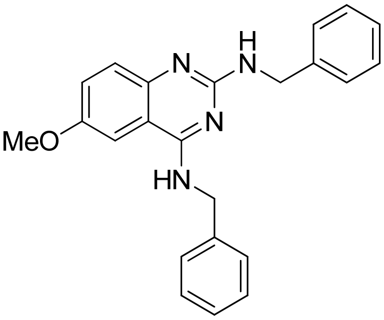

KSC-16-172

***N*²,*N*⁴-Dibenzyl-6-methoxyquinazoline-2,4-diamine (S138)**. Yield: 22.5 mg, 46%. ^{1}H NMR (400 MHz, $CDCl_3$) δ 7.36 (d, J = 9.1 Hz, 1H), 7.32 – 7.08 (m, 11H), 6.77 (d, J = 2.7 Hz, 1H), 5.80 (s, 1H), 5.17 (s, 1H), 4.69 (d, J = 5.4 Hz, 2H), 4.61 (d, J = 5.4 Hz, 2H), 3.70 (s, 3H). ^{13}C NMR (101 MHz, $CDCl_3$) δ 159.6, 158.6, 154.3, 147.3, 140.2, 138.9, 128.7, 128.4, 128.0, 127.6, 127.5, 127.0, 126.9, 123.4, 110.8, 101.4, 55.7, 45.7, 45.1. HRMS (m/z): calcd for $C_{23}H_{23}N_4O$ (M+H) 371.1872; found 371.1874.

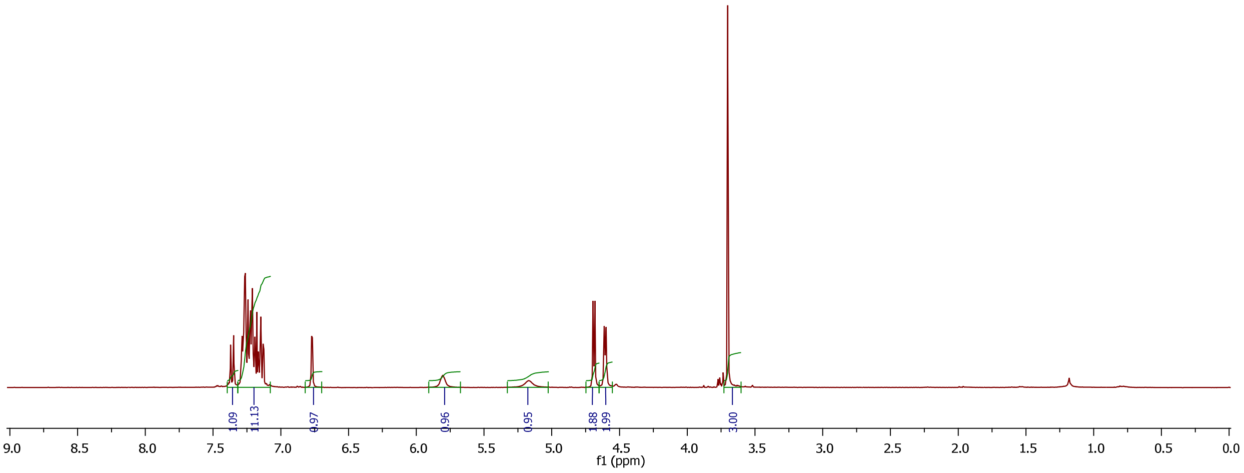

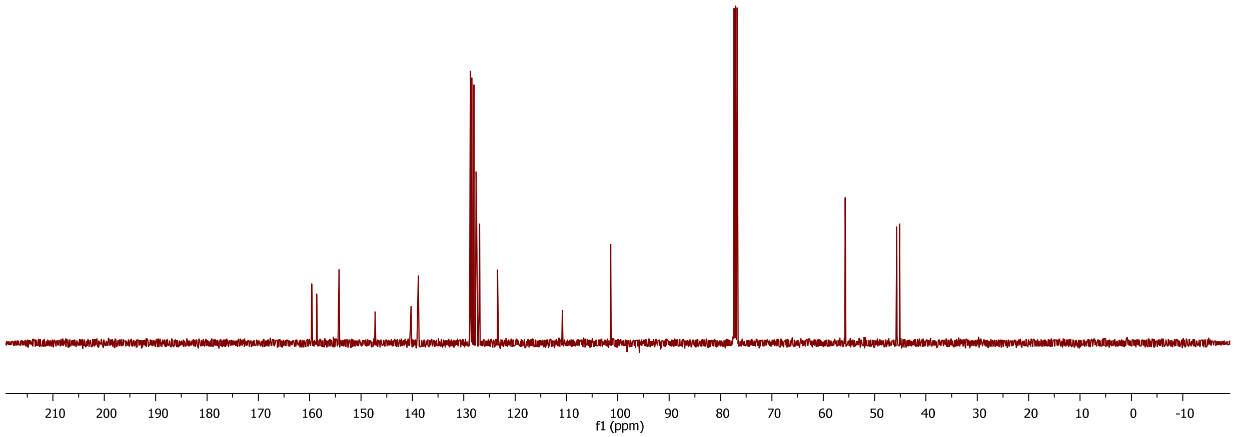

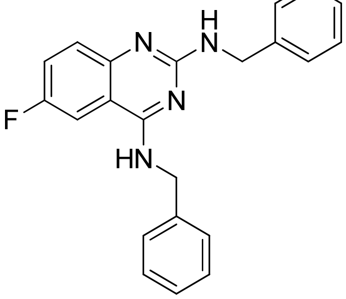

KSC-16-175

***N^2,N^4*-Dibenzyl-6-fluoroquinazoline-2,4-diamine (S139)**. Yield: 12 mg, 24%. ^{1}H NMR (400 MHz, $CDCl_3$) δ 7.49 (dd, *J* = 5.2, 9.1 Hz, 1H), 7.43 – 7.30 (m, 10H), 7.27 (d, *J* = 7.2 Hz, 1H), 7.16 (dd, *J* = 2.7, 9.0 Hz, 1H), 5.70 (s, br. 1H), 5.40 (s, br. 1H), 4.78 (d, *J* = 5.4 Hz, 2H), 4.73 (d, *J* = 5.7 Hz, 2H). ^{13}C NMR (101 MHz, $CDCl_3$) δ 159.7, 159.6, 159.2, 158.4, 156.0, 148.9, 140.0, 138.4, 128.8, 128.5, 128.0, 127.7, 127.6, 127.0, 122.0, 121.8, 110.7, 110.6, 105.5, 105.3, 103.0, 45.6, 45.2. HRMS (m/z): calcd for $C_{22}H_{20}FN_4$ (M+H) 359.1672; found 359.1672.

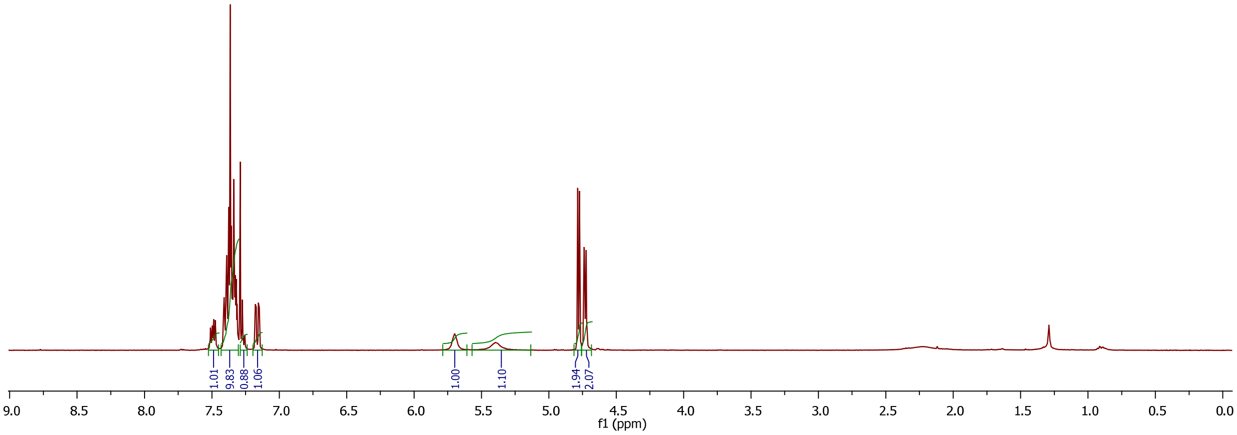

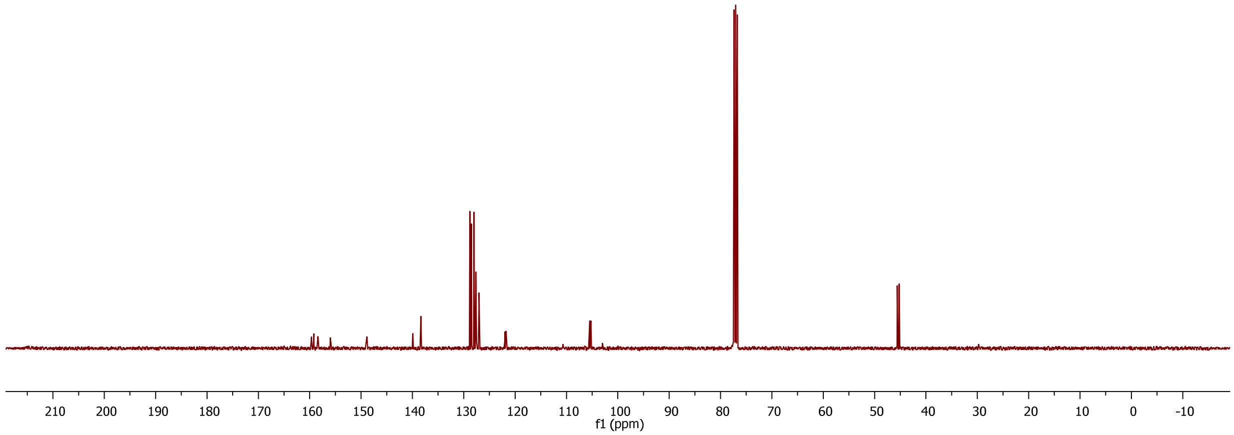

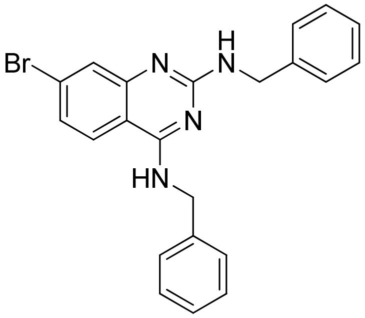

KSC-16-122

***N*[2],*N*[4]-Dibenzyl-7-bromoquinazoline-2,4-diamine (S140)**. Yield: 12.7 mg, 17%. ^{1}H NMR (400 MHz, $CDCl_3$) δ 7.67 (s, br. 1H), 7.47 – 7.32 (m, 10H), 7.31 – 7.25 (m, 1H), 7.17 (dd, *J* = 1.9, 8.6, 1H), 5.78 (s, br. 1H), 5.37 (s, br. 1H), 4.77 (d, *J* = 5.4 Hz, 2H), 4.73 (d, *J* = 5.9 Hz, 2H). ^{13}C NMR (101 MHz, $CDCl_3$) δ 159.9, 159.9, 153.5, 139.8, 138.3, 128.8, 128.5, 128.0, 127.7, 127.6, 127.2, 127.1, 124.2, 122.1, 45.5, 45.2. HRMS (m/z): calcd for $C_{22}H_{20}BrN_4$ (M+H) 419.0871 and 421.0851; found 421.0845.

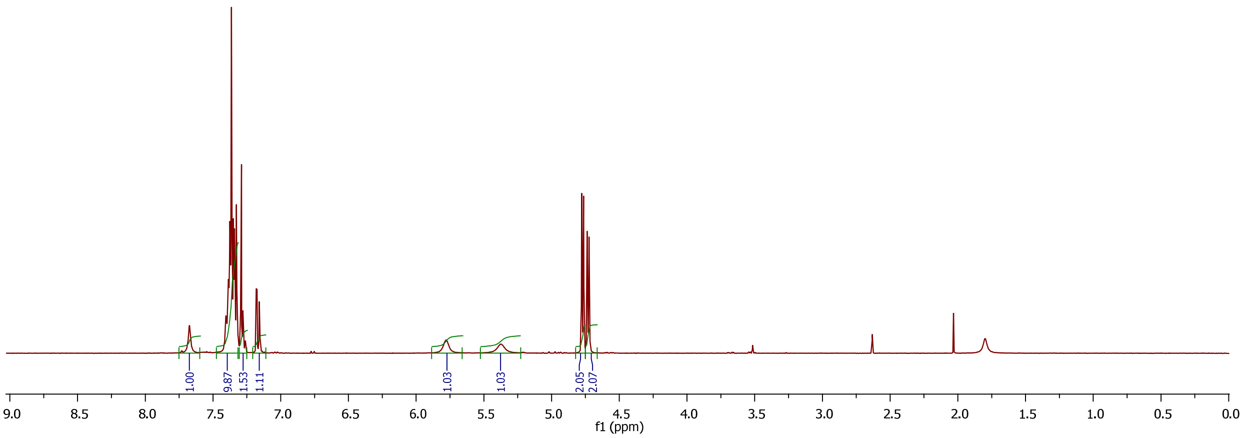

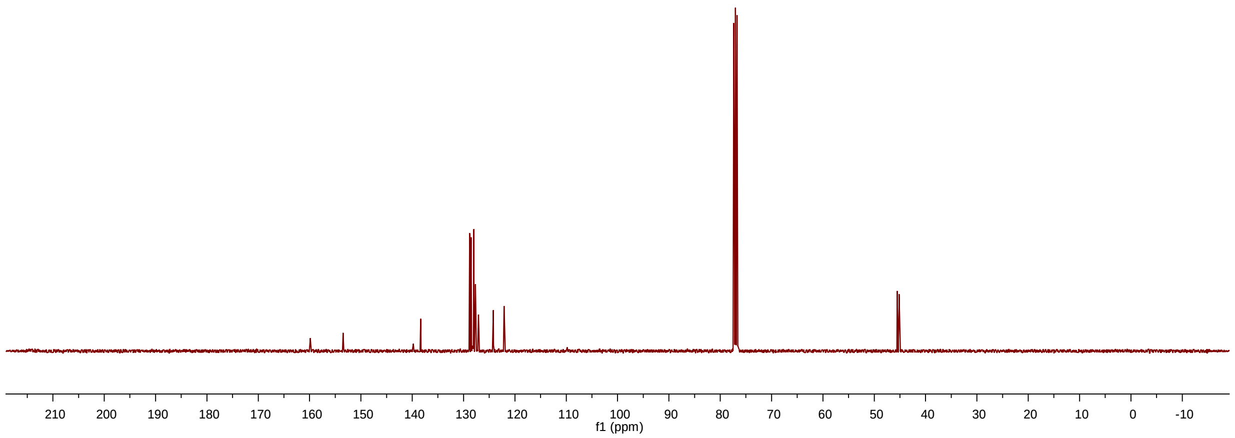

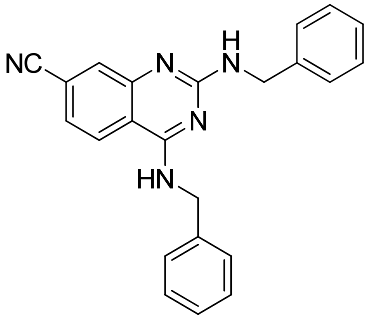

KSC-16-167c

2,4-Bis(benzylamino)quinazoline-7-carbonitrile (S141). Yield: 12.3 mg, 15%. ^{1}H NMR (400 MHz, $CDCl_3$) δ 7.79 (s, br. 1H), 7.55 (d, *J* = 8.3 Hz, 1H), 7.47 – 7.30 (m, 10H), 7.24 (dd, *J* = 1.6, 8.3 Hz, 1H), 5.83 (s, 1H), 5.55 – 5.32 (m, 1H), 4.78 (d, *J* = 5.4 Hz, 2H), 4.74 (d, *J* = 6.0 Hz, 2H). ^{13}C NMR (101 MHz, $CDCl_3$) δ 159.9, 159.5, 139.4, 137.9, 128.9, 128.6, 128.0, 127.9, 127.6, 127.2, 122.2, 118.4, 116.1, 45.5, 45.4. HRMS (m/z): calcd for $C_{23}H_{20}N_5$ (M+H) 366.1719; found 366.1719.

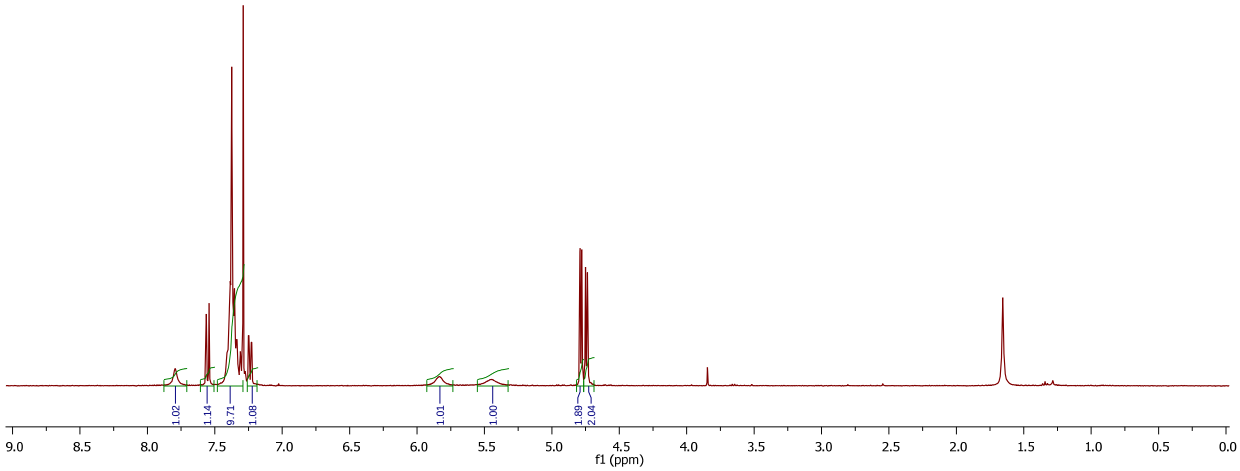

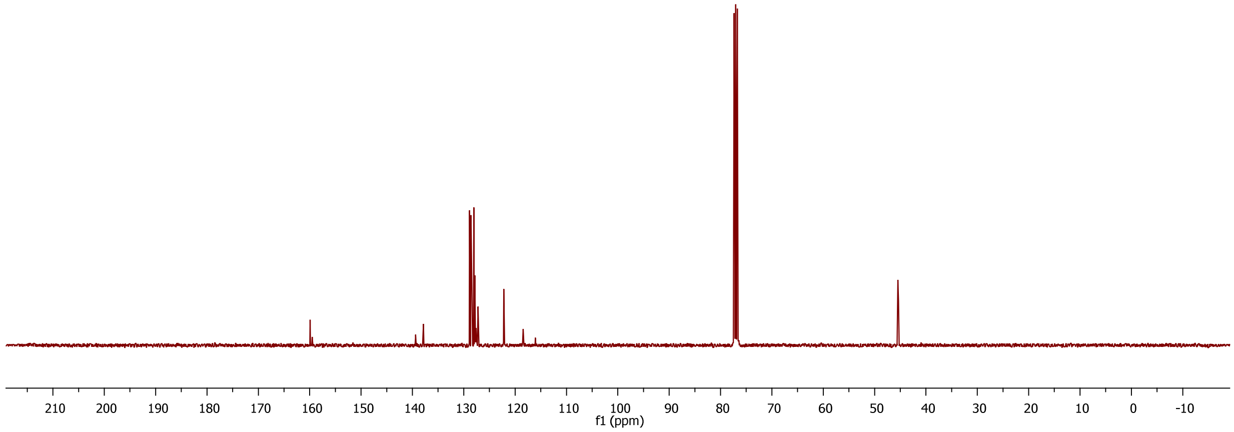

General procedure D: representative protocol for the synthesis of quinazoline analogues of less nucleophilic amines, synthesis and characterization for S166, S161, S163 (**31**), S164, S167 (**19**), S168.

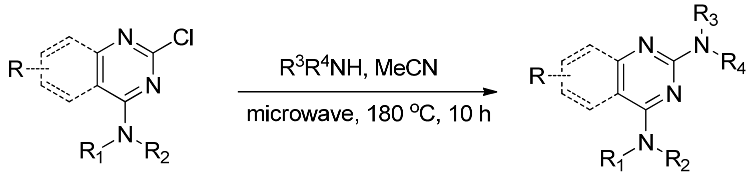

Scheme S4: Synthesis of quinazoline analogues of less nucleophilic amines

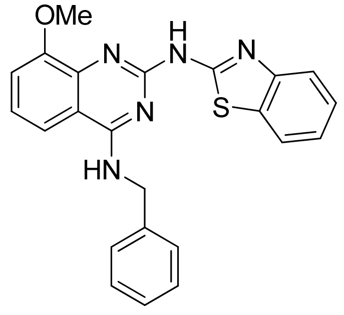

KSC-25-22

***N*[2]-(Benzo[*d*]thiazol-2-yl)-*N*[4]-benzyl-8-methoxyquinazoline-2,4-diamine (S166)**. To a suspension of N-benzyl-2-chloro-8-methoxyquinazolin-4-amine (15.0 mg, 50 µmol) in CH_3CN (1 mL) was added 2-aminobenzo[*d*]thiazole (15.0 mg, 0.1 mmol, 2 equiv.). The mixture was heated to 180 °C for 10 h under microwave irradiation. The solvent was removed under vacuum, the residue was suspended in EtOAc, washed with saturated $NaHCO_3$, and the layers were separated. The organic layer was dried over $MgSO_4$ and concentrated under vacuum. The residue was purified by reverse phase preparative HPLC to give the product (7.2 mg, 35%). ^{1}H NMR (400 MHz, DMSO) δ 11.43 (s, br. 1H), 8.91 (s, 1H), 7.91 – 7.75 (m, 2H), 7.62 (d, *J* = 7.8 Hz, 1H), 7.48 (d, *J* = 7.2 Hz, 2H), 7.40 – 7.30 (m, 3H), 7.30 – 7.11 (m, 4H), 4.94 (d, *J* = 5.6 Hz, 2H), 3.96 (s, 3H). ^{13}C NMR (126 MHz, DMSO) δ 160.3, 160.2, 153.5, 153.3, 149.7, 141.8, 139.3, 132.4, 128.3, 127.5, 126.8, 125.5, 122.9, 121.9, 120.8, 119.2, 117.7, 114.1, 112.5, 55.8, 44.1. HRMS (m/z): calcd for $C_{23}H_{20}N_5OS$ (M+H) 414.1389; found 414.1390.

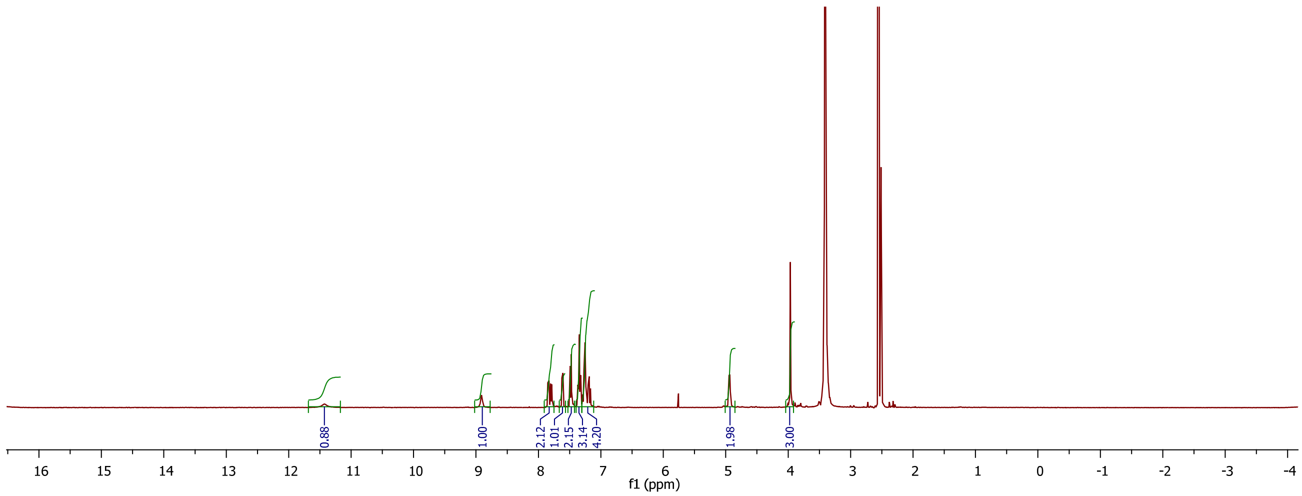

0.88
1.00
2.12
1.01
2.15
3.14
4.20
1.98
3.00
16 15 14 13 12 11 10 9 8 7 6 5 4 3 2 1 0 -1 -2 -3 -4
f1 (ppm)

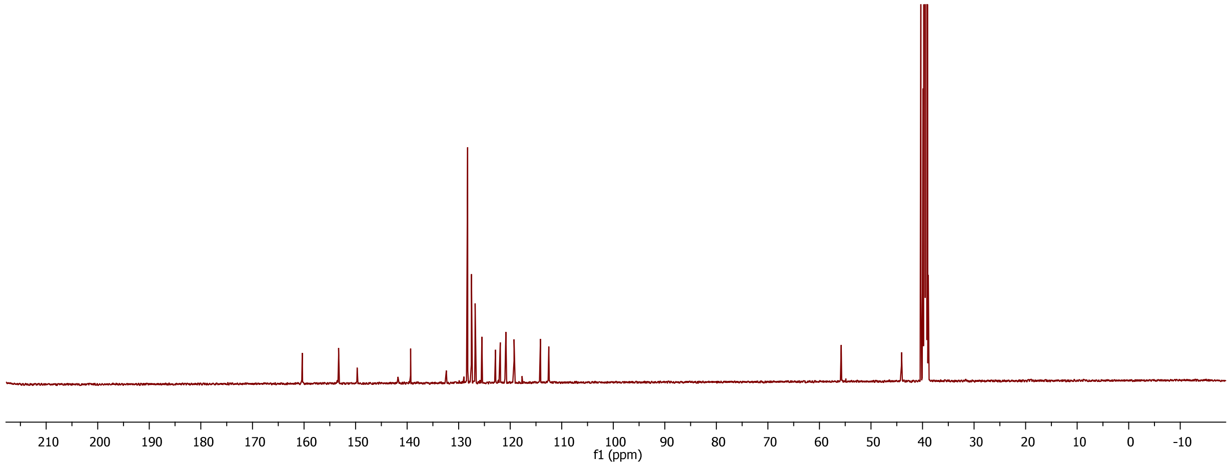

210 200 190 180 170 160 150 140 130 120 110 100 90 80 70 60 50 40 30 20 10 0 -10
f1 (ppm)

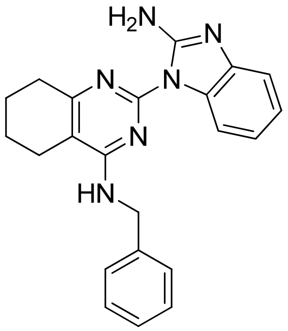

KSC-25-31

2-(2-Amino-*1H*-benzo[*d*]imidazol-1-yl)-*N*-benzyl-5,6,7,8-tetrahydroquinazolin-4-amine (S161, 18). Yield: 1.8 mg, 12%. ^{1}H NMR (400 MHz, DMSO) δ 7.92 (d, *J* = 7.4 Hz, 1H), 7.84 (s, 1H), 7.65 (s, 2H), 7.40 – 7.29 (m, 4H), 7.23 (s, 1H), 7.11 (d, *J* = 7.1 Hz, 1H), 6.99 (t, *J* = 7.0 Hz, 1H), 6.79 – 6.72 (m, 1H), 4.71 (d, *J* = 6.0 Hz, 2H), 2.70 (s, br. 2H), 2.49 (s, br. 2H), 1.83 (s, br. 4H). ^{13}C NMR (126 MHz, DMSO) δ 161.0, 160.8, 154.3, 154.1, 142.8, 139.3, 131.6, 128.3, 126.7, 126.4, 122.2, 118.6, 114.6, 114.3, 108.6, 44.1, 31.3, 21.9, 21.7, 21.6. HRMS (m/z): calcd for $C_{22}H_{23}N_6$ (M+H) 371.1984; found 371.1989.

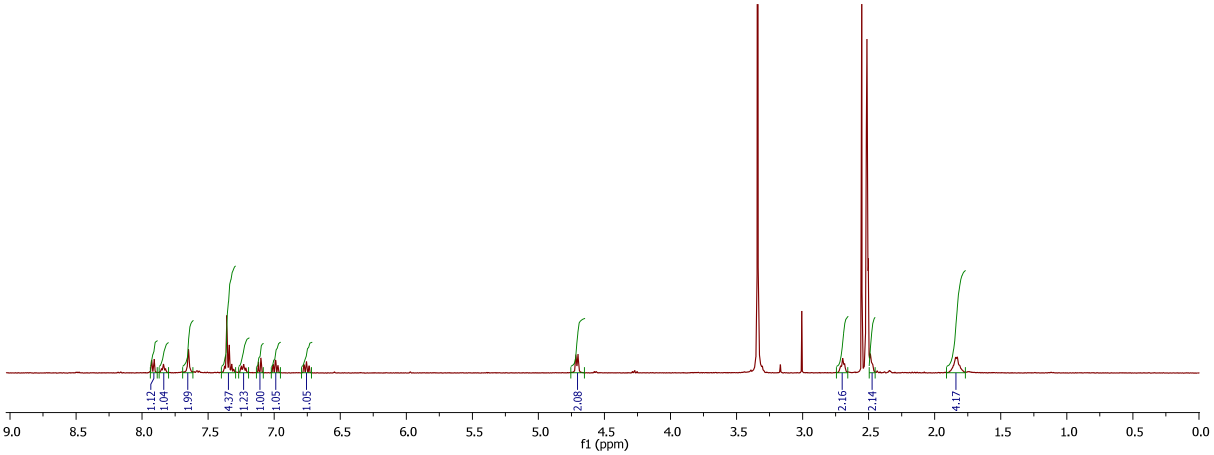

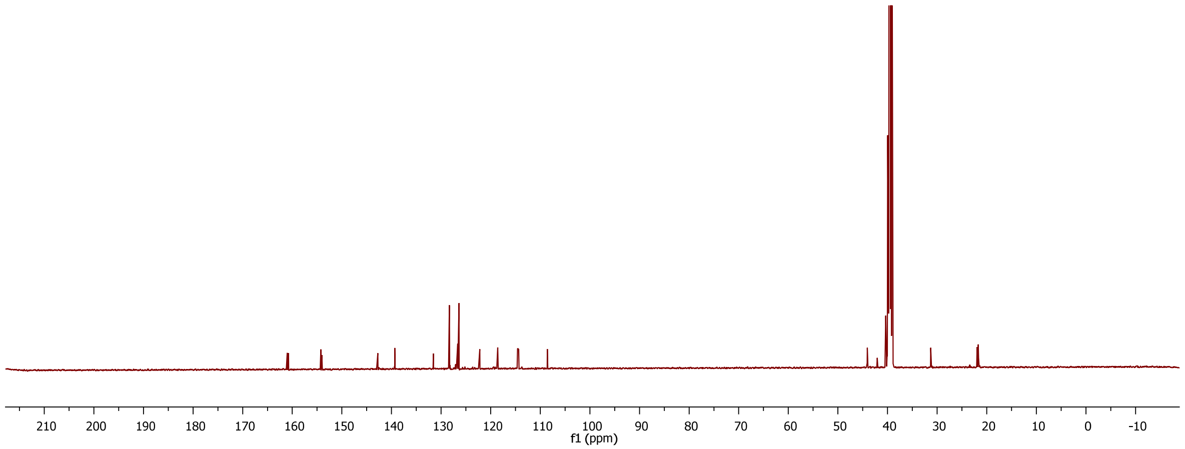

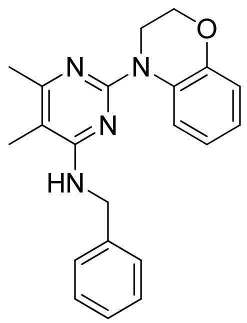

KSC-25-24

2-(*2H*-Benzo[*b*][1,4]oxazin-4(*3H*)-yl)-*N*-benzyl-5,6-dimethylpyrimidin-4-amine (S163 or 31). (Reaction time = 6 h) Yield: 6.5 mg, 46%. ^{1}H NMR (400 MHz, $CDCl_3$) δ 8.13 – 7.99 (m, 1H), 7.42 – 7.30 (m, 5H), 6.94 – 6.87 (m, 2H), 6.81 – 6.74 (m, 1H), 4.82 (s, 1H), 4.69 (d, *J* = 5.6 Hz, 2H), 4.31 – 4.26 (m, 2H), 4.29 – 4.23 (m, 2H), 2.35 (s, 3H), 2.01 (s, 3H). ^{13}C NMR (101 MHz, $CDCl_3$) δ 161.7, 160.9, 157.2, 145.9, 139.5, 128.6, 128.4, 127.5, 127.2, 124.0, 122.7, 119.5, 116.6, 101.8, 65.9, 45.3, 42.2, 22.2, 10.9. HRMS (m/z): calcd for $C_{21}H_{23}N_4O$ (M+H) 347.1872; found 347.1872.

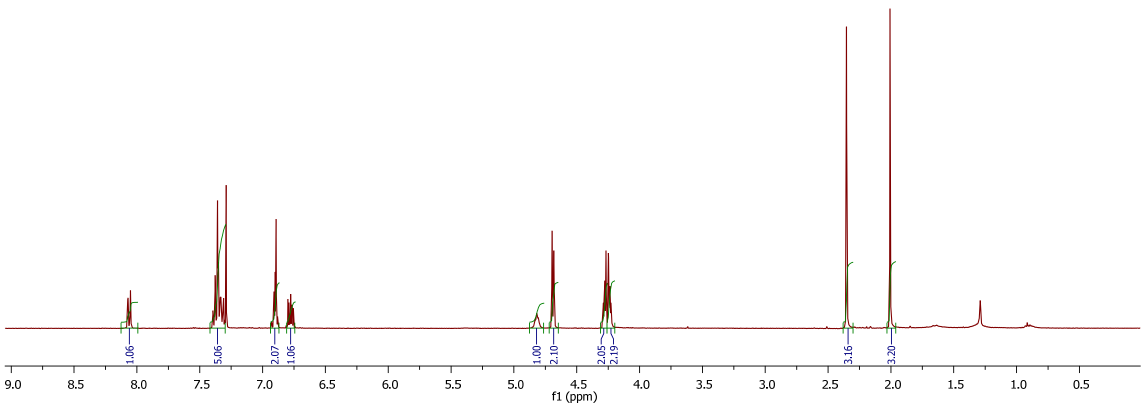

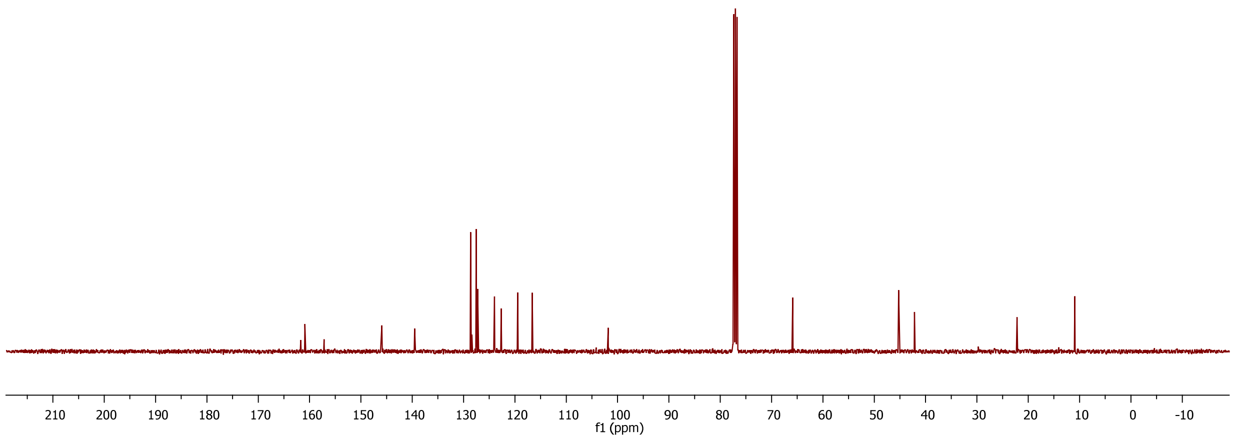

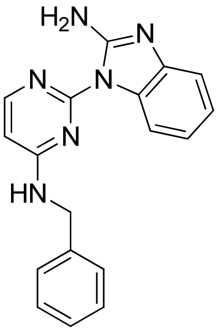

KSC-25-32

1-(4-(Benzylamino)pyrimidin-2-yl)-*1H*-benzo[*d*]imidazol-2-amine (S164). Yield: 11.7 mg, 48%. ^{1}H NMR (400 MHz, DMSO) δ 8.51 (s, 1H), 8.16 (s, 1H), 8.05 (s, 1H), 7.75 (s, 2H), 7.41 – 7.35 (m, 4H), 7.28 (s, 1H), 7.15 (d, *J* = 7.6 Hz, 1H), 7.05 – 7.02 (m, 1H), 6.83 (t, *J* = 7.8 Hz, 1H), 6.51 (d, *J* = 5.8 Hz, 2H), 4.66 (s, 2H). ^{13}C NMR (101 MHz, DMSO) δ 162.6, 156.9, 154.4, 153.8, 143.0, 138.7, 131.5, 128.5, 127.0, 122.6, 118.8, 114.7, 114.6, 102.5, 101.3, 43.9. HRMS (m/z): calcd for $C_{18}H_{17}N_6$ (M+H) 317.1515; found 317.1520.

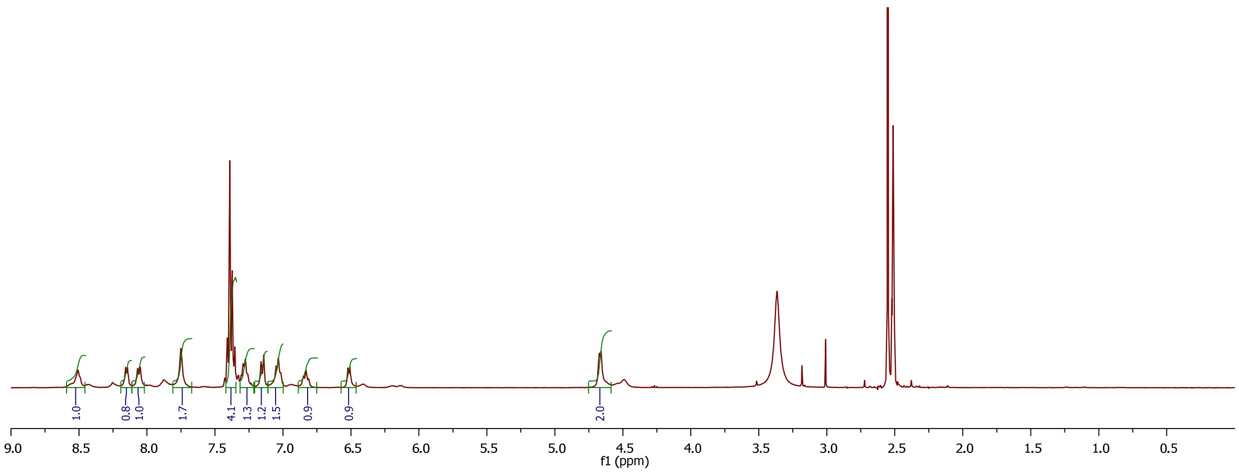

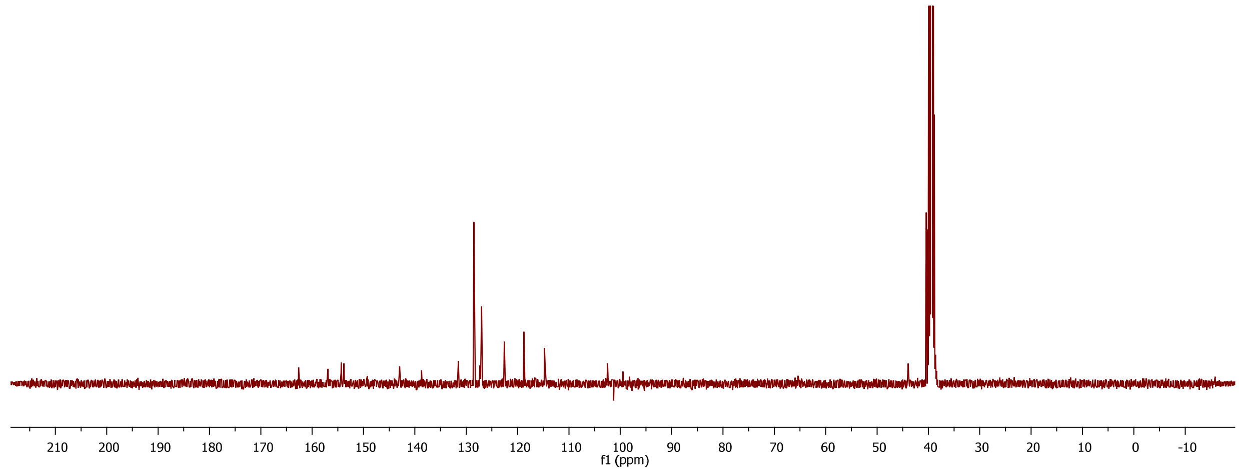

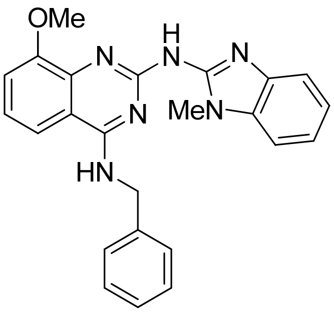

KSC-25-21

***N*[4]-Benzyl-8-methoxy-*N*[2]-(1-methyl-*1H*-benzo[*d*]imidazol-2-yl)quinazoline-2,4-diamine (S167 or 19)**. Yield: 3.9 mg, 19%. ^{1}H NMR (500 MHz, DMSO) δ 9.36 (s, 1H), 8.28 (s, 1H), 8.24 (d, *J* = 8.0 Hz, 1H), 7.93 (d, *J* = 7.9 Hz, 1H), 7.51 (s, 1H), 7.50 – 7.39 (m, 2H), 7.35 (td, *J* = 2.7, 9.5 Hz, 3H), 7.25 (t, *J* = 7.3 Hz, 1H), 7.16 (d, *J* = 5.4 Hz, 2H), 6.93 (s, 1H), 4.88 (d, *J* = 5.7 Hz, 2H), 3.99 (s, 3H), 3.42 (s, 3H). HRMS (m/z): calcd for $C_{24}H_{23}N_6O$ (M+H) 411.1933; found 411.1937.

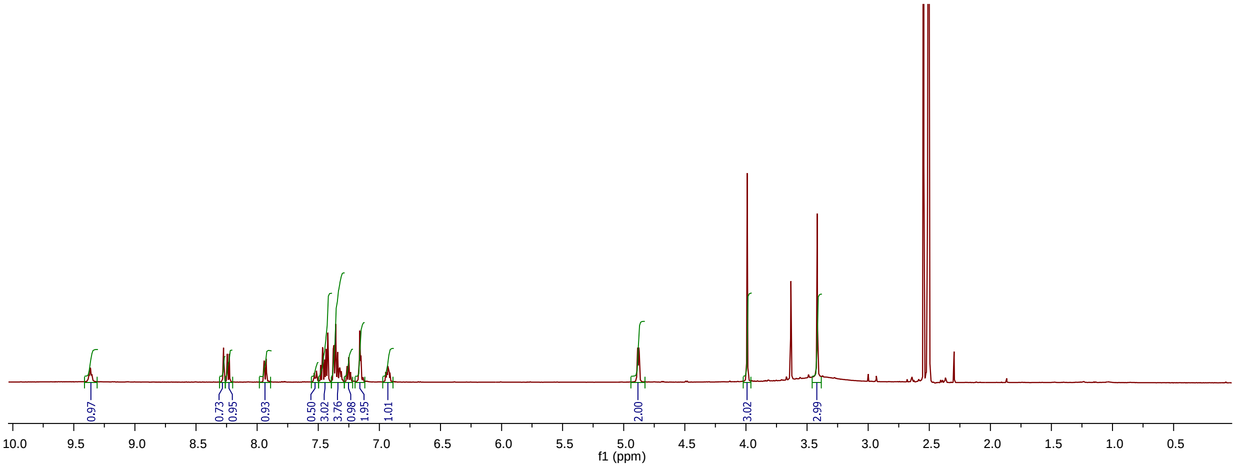

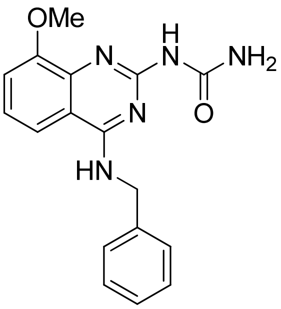

KSC-25-12

1-(4-(Benzylamino)-8-methoxyquinazolin-2-yl)urea (S168). (Reaction time = 3 h) Yield: 4.7 mg, 47%. ^{1}H NMR (500 MHz, DMSO) δ 8.32 (d, *J* = 7.1 Hz, 1H), 7.58 (dd, *J* = 1.3, 8.2 Hz, 1H), 7.36 (d, *J* = 7.0 Hz, 2H), 7.31 (dd, *J* = 4.9, 10.3 Hz, 2H), 7.23 (t, *J* = 7.2 Hz, 1H), 7.01 (d, *J* = 6.7 Hz, 1H), 6.96 (t, *J* = 7.9 Hz, 1H), 6.13 (s, 2H), 4.73 (d, *J* = 5.9 Hz, 2H), 3.81 (s, 3H). ^{13}C NMR (126 MHz, DMSO) δ 160.2, 159.8, 152.6, 143.6, 139.9, 128.2, 127.9, 127.3, 126.6, 119.5, 114.0, 111.3, 111.0, 55.2, 43.1. HRMS (m/z): calcd for $C_{17}H_{18}N_5O_2$ (M+H) 324.1460; found 324.1449.

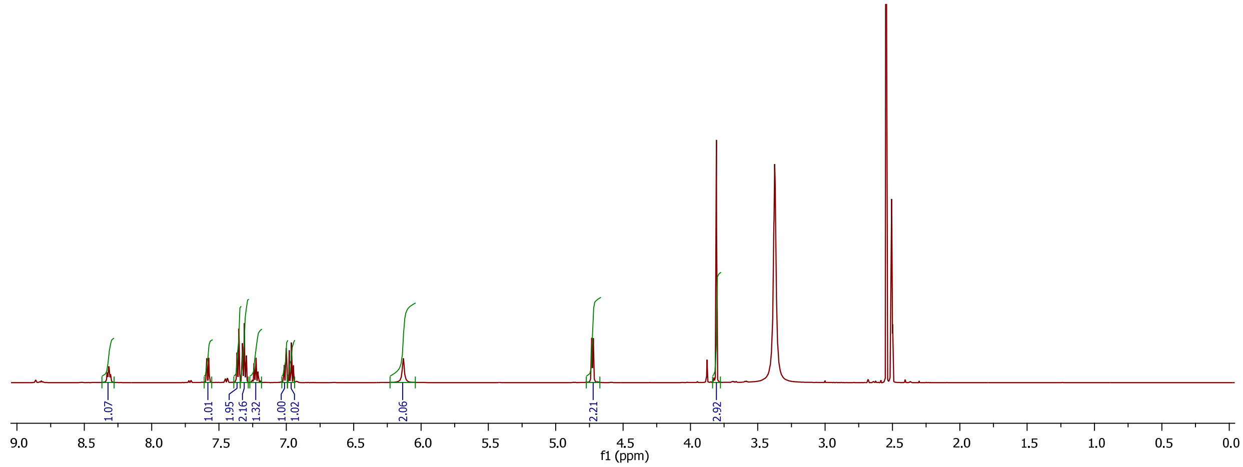

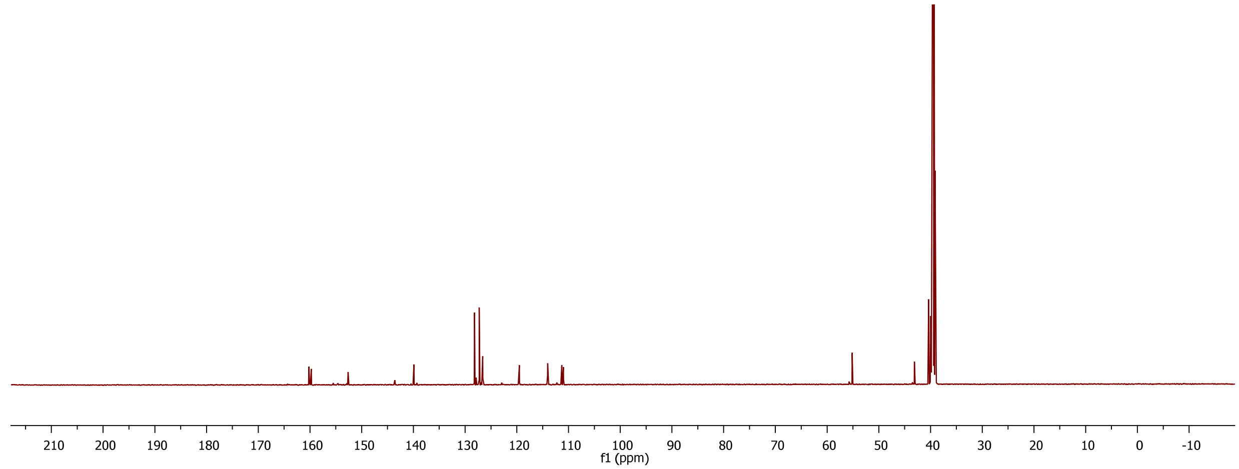

General procedure E: representative protocol for the synthesis of quinazoline analogues via a Suzuki coupling route, synthesis and characterization for S153 (**36**) and S149.

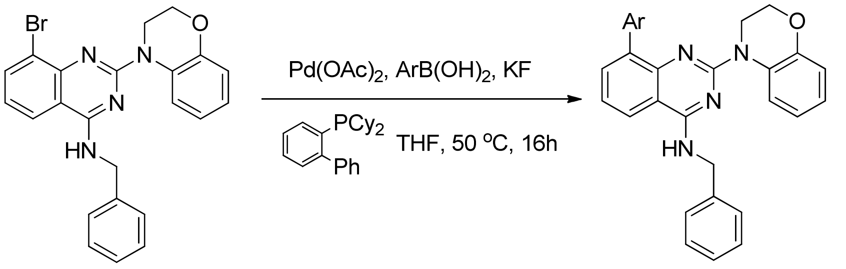

Scheme S5. Derivitization of quinazoline analogues via a Suzuki coupling route.

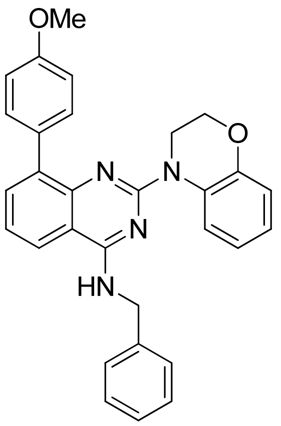

KSC-16-268

2-(*2H*- Benzo[*b*][1,4]oxazin-4(*3H*)-yl)-N-benzyl-8-(4-methoxyphenyl)quinazolin-4-amine (S153 or 36). A mixture of 2-(*2H*-benzo[*b*][1,4]oxazin-4(*3H*)-yl)-N-benzyl-8-bromoquinazolin-4-amine (12.0 mg, 26.8 μmol), palladium acetate (0.6 mg, 2.7 μmol), [1,1'-biphenyl]-2-yldicyclohexylphosphine (1.9 mg, 5.4 μmol), 4-methoxyphenylboronic acid (6.1 mg, 40.2 μmol), potassium fluoride (4.7 mg, 80.5 μmol) in 0.5 mL of THF was heated at 50 ºC for 16 h. The mixture was diluted with EtOAc and washed with water. The orgainc phase was concentrated and purified by SGC (DCM/MeOH = 1:9, Rf = 0.5). Yield: 12 mg, 94%. ^{1}H NMR (400 MHz, $CDCl_3$) δ 8.17 – 8.09 (m, 1H), 7.75 – 7.69 (m, 2H), 7.68 (dd, *J* = 1.4, 7.3 Hz, 1H), 7.56 (dd, *J* = 1.3, 8.2 Hz, 1H), 7.40 (dd, *J* = 1.9, 3.5 Hz, 4H), 7.39 – 7.32 (m, 1H), 7.28 – 7.21 (m, 1H), 7.07 – 7.00 (m, 2H), 6.92 (dtd, *J* = 1.6, 8.1, 9.9 Hz, 2H), 6.77 – 6.70 (m, 1H), 5.94 (s, 1H), 4.85 (t, *J* = 5.3 Hz, 2H), 4.33 – 4.25 (m, 4H), 3.92 (s, 3H). ^{13}C NMR (101 MHz, $CDCl_3$) δ 160.1, 158.8, 156.1, 149.5, 146.2, 138.6, 137.6, 133.1, 131.9, 131.6, 128.8, 128.2, 127.7, 127.6, 125.1, 123.2, 121.9, 119.5, 119.4, 116.5, 113.2, 111.6, 66.1, 55.4, 45.5, 42.1. HRMS (m/z): calcd for $C_{30}H_{27}N_4O_2$ (M+H) 475.2134; found 475.2135.

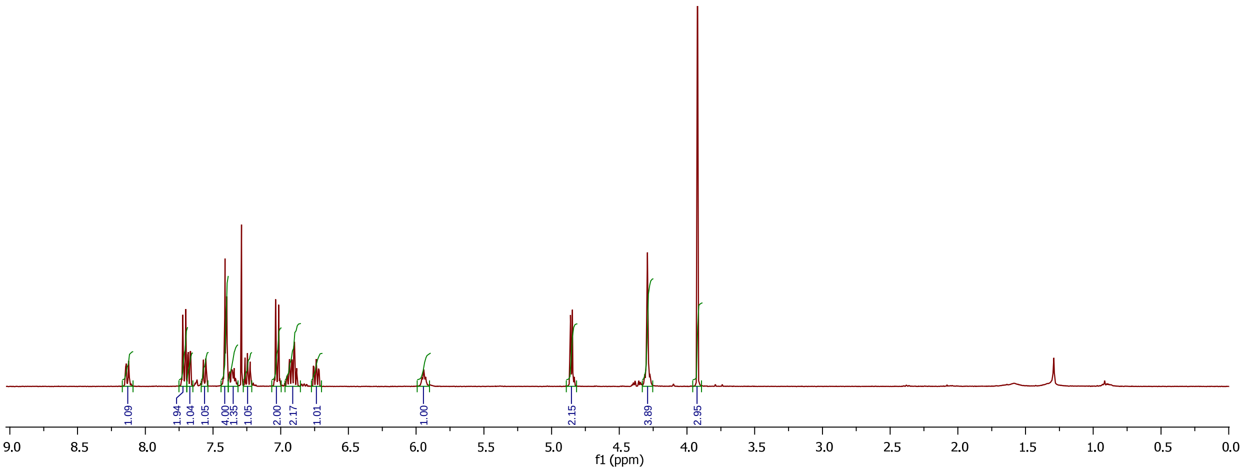
1.09
1.94
1.04
1.05
4.00
1.35
1.05
2.00
2.17
1.01
1.00
2.15
3.89
2.95
9.0 8.5 8.0 7.5 7.0 6.5 6.0 5.5 5.0 4.5 4.0 3.5 3.0 2.5 2.0 1.5 1.0 0.5 0.0
f1 (ppm)

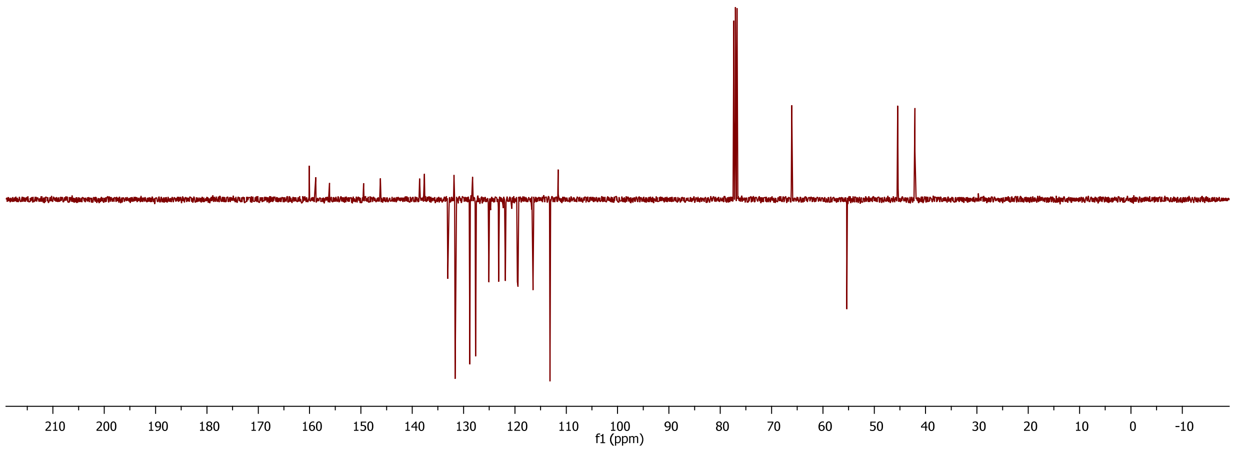
210 200 190 180 170 160 150 140 130 120 110 100 90 80 70 60 50 40 30 20 10 0 -10
f1 (ppm)

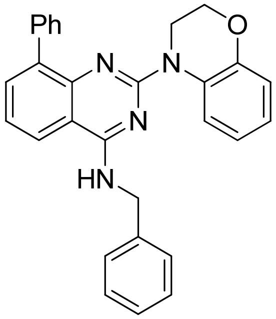

KSC-16-255

2-(*2H*-Benzo[*b*][1,4]oxazin-4(*3H*)-yl)-N-benzyl-8-phenylquinazolin-4-amine (S149). Yield: 3.9 mg, 33%. ^{1}H NMR (400 MHz, $CDCl_3$) δ 8.03 (d, *J* = 7.0 Hz, 1H), 7.69 – 7.55 (m, 3H), 7.50 (d, *J* = 7.0 Hz, 1H), 7.38 (t, *J* = 7.4 Hz, 2H), 7.34 – 7.22 (m, 5H), 7.18 – 7.11 (m, 1H), 6.79 (dt, *J* = 6.6, 8.1 Hz, 2H), 6.62 (dd, *J* = 4.3, 10.9 Hz, 1H), 5.86 (s, 1H), 4.76 (d, *J* = 5.4 Hz, 2H), 4.18 (s, 4H). ^{13}C NMR (126 MHz, CDCl3) δ 160.0, 156.1, 149.5, 146.2, 139.5, 138.5, 138.1, 133.5, 130.5, 128.8, 128.2, 127.7, 127.7, 127.6, 126.9, 125.0, 123.2, 121.8, 120.0, 119.4, 116.4, 111.6, 66.0, 45.5, 42.0. HRMS (m/z): calcd for $C_{29}H_{25}N_4O$ (M+H) 445.2028; found 445.2025.

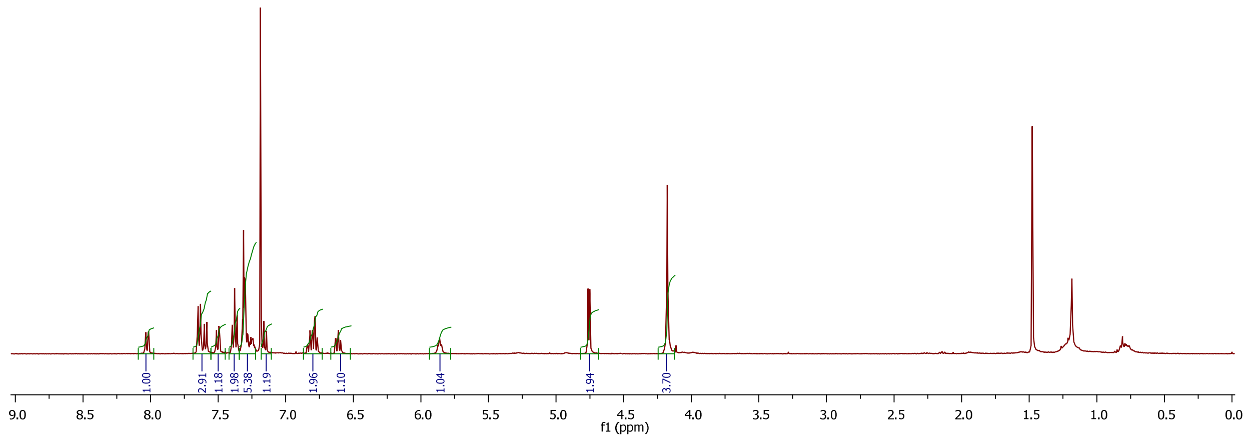

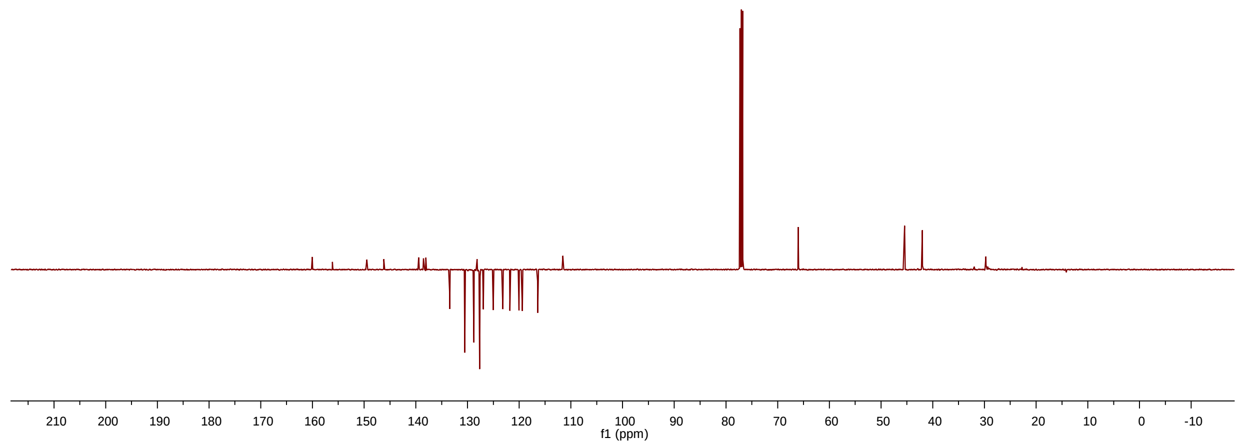

Supplement: Supplementary file 1 [file cmdc0008-0297-sd1.pdf]
